# Factors Associated with Psychiatric Comorbidity in Depression Patients in Primary Health Care in Chile

**DOI:** 10.1155/2018/1701978

**Published:** 2018-10-01

**Authors:** Alfredo Cancino, Marcelo Leiva-Bianchi, Carlos Serrano, Soledad Ballesteros-Teuber, Cristian Cáceres, Verónica Vitriol

**Affiliations:** ^1^Medicine School, Universidad de Talca, Talca, Chile; ^2^Communal Mental Health Program, Primary Health Care Department, Municipality of Curicó, Curicó, Chile; ^3^Faculty of Psychology, University of Talca, Talca, Chile; ^4^Hospital San Juan de Dios, Curicó, Chile

## Abstract

**Objective:**

To identify the clinical and psychosocial factors associated with psychiatric comorbidity in patients consulting for depression in Primary Health Care (PHC) in Chile.

**Methods:**

394 patients with a diagnosis of major depression being treated in a Chilean PHC were evaluated using a sociodemographic and clinical interview, the mini-international neuropsychiatric interview (MINI), a childhood trauma events (CTEs) screening, the intimate partner violence (IPV) scale, the Life Experiences Survey (LES), and the Hamilton Depression Rating Scale (HDRS).

**Results:**

Positive correlations were established between higher number of psychiatric comorbidities and severity of depressive symptoms (r = 0.358), frequency of CTEs (r = 0.228), frequency of IPV events (r = 0.218), frequency of recent stressful life events (r = 0.188), number of previous depressive episodes (r = 0.340), and duration of these (r = 0.120). Inverse correlations were determined with age at the time of the first consultation (r = -0.168), age of onset of depression (r = -0.320), and number of medical comorbidities (r = -0.140). Of all associated factors, early age of the first depressive episode, CTEs antecedents, and recent stressful life events explain 13.6% of total variability in psychiatric comorbidities.

**Conclusions:**

A higher prevalence of psychiatric comorbidity among subjects seeking help for depression in Chilean PHCs is associated with early onset of depression, clinical severity, chronicity, and interpersonal adversity experienced since childhood.

## 1. Introduction

Major depression is an important public health problem [1, 2]. Worldwide, it ranks fourth among the most disabling diseases [1].

Epidemiological studies conducted in the general population [3–7] show that, among depressed patients, comorbid anxiety disorders are found in 50% of cases [2, 4, 6, 7]. Compared with those who only have depression, patients with anxious-depression comorbidity develop more severe symptoms and greater chronicity, have a worse prognosis, and require different pharmacological and psychological therapeutic approaches [8].

The comorbidity of depression with other psychiatric disorders, mainly with anxiety disorders, remains a field that requires further investigation [9]. The categorization of both diagnoses has been questioned as separate nosological entities, due to the high comorbidities observed in clinical practice and genetic studies [6, 10]. Winokur and other authors used the term “primary-secondary depression” to differentiate the clinical manifestations and the antidepressant response in patients with pure depression from patients with comorbid depression [11]. The importance of anxiety in depression is now recognized in the DSM-V [12–14], but current therapeutic guidelines for depression still do not provide a specific indication for recognition and treatment in those patients who present with such comorbidity [9].

In most countries, primary health care (PHC) is the front line for the diagnosis and treatment of depression [15]. At this level of care, the frequency of comorbid anxiety disorders in depressed patients can be up to 75% [16, 17]. However, general practitioners (GPs) frequently do not recognize anxiety in patients with depressive symptoms [16, 18], a fact that is associated with a worse prognosis of depression, more chronicity, higher risk of suicide, and higher health costs [17–20].

In Chile, as in the world, major depression constitutes a complex public health problem [21]. Epidemiological studies conducted in the general population show that the lifetime prevalence of a major depressive episode is around 9% [22] and the presence of depressive symptoms during the last year reaches 17.2% [21].

During the last decades, Chilean health authorities have instituted a specific program for the treatment of depression in public services [23]. According to this, 90% of depressed Chilean patients are treated in PHC by GPs and psychosocial teams [23, 24]. However, in this country, despite the time of implementation of this PHC program [21, 23, 25], the evidence on the clinical recognition and the influence of psychiatric comorbidity on symptomatological severity, therapeutic response, and illness course is still scarce [21, 25].

Recent studies, carried out in a sample of depressed patients in PHC in the south-central zone of Chile [26, 27], found 89,1% had psychiatric comorbidities, most of which corresponded to the anxious spectrum. In this sample, the psychiatric comorbidity was the factor that, independent of other factors (childhood trauma, domestic violence, life stressor events, early onset of depression, and more frequency of depressive episodes), predicted the greatest clinical severity at the time of the consultation [26] and it also turned out to be the factor most associated with lower remission rates at twelve months [28].

Evidence on the characteristics associated with the comorbidity of depression with anxiety has been obtained, mainly, from clinical samples or epidemiological studies carried out in North America, Europe, and Asia [3–7]. However, there is a lack of evidence on the importance of the different factors to explain this comorbidity, specifically in PHC and in samples from Latin American countries.

Studying the above could have practical and theoretical implications. In PHC, in practical terms, it could help to guide GPs in the search for a subgroup of depressed patients that require a differential approach. Theoretically, it could contribute to provide evidence that eventually help to understand the complex phenomenon of comorbidity in patients with depression.

The objective of this research is to identify which factors are the most important associated with greater psychiatric comorbidity at the time of consultation in Chilean PHC depressed patients.

## 2. Methods

### 2.1. Design

A cross-sectional quantitative study was conducted. The research was approved by the Bioethics Committees of the University of Talca and the Maule regional Health Service.

### 2.2. Sample

Between February and September 2014, 2978 patients older than 15 years old entered treatment for depression in 8 PHC urban clinics in the cities of Talca and Curicó, Maule region, Chile. During the first consultation, after determining their suitability to sign an informed consent, each GP invited their patients to participate in the study.

Exclusion criteria were organic cognitive disability (such as mental weakness or clues of dementia), sensory impairment (such as hearing loss), and direct referral to psychiatry service due to current severe suicide attempt and/or suspicion of bipolarity and/or psychosis upon entering.

440 patients without excluding criteria were accepted to participate in the research and after signing an informed consent, they were interviewed by a team of psychiatrists and psychologists, who proceeded to confirm the diagnosis of depressive episode based on the tenth version of the International Classification of Diseases (ICD-10) criteria [29].

MDD was confirmed in only 394 patients. The rest of them continued receiving treatment in a PHC Mental Health unit, according to their diagnosis.

Therefore, patients with a confirmed diagnosis of MDD were evaluated by the research team using the following instruments:

1. Semistructured clinical interview, designed by the research team, which compiled (a) sociodemographic background: age of consultation, marital status, schooling, and current work activity; (b) clinical backgrounds on depressive illness: age of first depressive episode, number of depressive episodes, time of evolution of the current episode, time of evolution of the longest episode, history of attempted suicide, history of previous treatment, and history of hospitalization; (c) chronic biomedical pathologies currently under control: hypertension, diabetes, coronary pathology, arthrosis, chronic pain, cancer, Parkinson's disease, thyroid dysfunction, and so forth.
2. Mini-International Neuropsychiatric Interview (MINI), for diagnosis of the main psychiatric disorders listed in the ICD-10 and in the fourth edition of the Diagnostic and Statistical Manual of Mental Disorders (DSM-IV) [30].
3. Screening for CTEs, [31]: this screening delves memories before 15 years about traumatic separation from the patient's caregiver for more than a month, alcohol or drug abuse by a family member, physical abuse and physical injury associated with physical abuse, domestic violence between parents or caregivers, and sexual abuse by a relative and / or nonrelative. This instrument has been validated in Chile and has been applied in previous investigations [32, 33].
4. Life Experiences Survey (LES): this is a 47-item scale that investigates life changes experienced during the previous six months, with both positive and negative meanings. A Spanish translation was used. For the purposes of this research, only stressful life events with negative connotations were considered [34, 35].
5. Questionnaire for IPV, including 12 questions that inquire about having been a victim of violence perpetrated by the intimate partner during life, taking into account the World Health Organization (WHO) definitions for physical, psychological, and sexual violence. This instrument has been used in previous studies in Chile [36].
6. The 17-item Hamilton Depression Rating Scale(HDRS): this was used to determine the severity of the depressive symptoms [37].

### 2.3. Statistical Data Analysis

The statistical data analysis was performed using the fourteenth version of the Statistical Package for the Social Sciences (SPSS) program.

Univariate descriptive statistics for sociodemographic and clinical characteristics of the sample were made.

To determine the association between comorbidities and other continuous variables, bivariate Pearson correlations were performed.

Finally, to determine the highest comorbidity from the variables that would be significant, a multiple linear regression model (successive steps method) was used.

The level of significance was 0.05.

## 3. Results

### 3.1. Sociodemographic and Clinical Characteristics

**Table 1** shows the sociodemographic and clinical characteristics of the sample. Most of the individuals were women, in their middle age, and without a paid job and half of them were living as a couple. The average of onset of the first episode was in the third decade of life. The majority of patients presented chronic and recurrent symptoms and one-third of them with a history of attempted suicide.

### 3.2. Adverse Biographical Events

82% of the sample reported at least one CTE. Any type of childhood sexual abuse was remembered by 33.1% of sample. One or more recent stressful life events were present in 92.6% of the patients and 58.1% of the studied subjects reported having suffered some type of IPV event at some time throughout their lives.

### 3.3. Biomedical and Psychiatric Comorbidities

55% of the patients presented with at least one biomedical pathology (21.4% only one, 18.9% two, and 33.9% three or more).

Regarding psychiatric comorbidities, 89.1% of the sample had some comorbid disorder (20.6% only one, 20.3% two disorders, 19.5% three, and 28.6% more than 3 comorbidities). The distribution of the different psychiatric comorbidities can be seen in**Table 2**.

### 3.4. Associations between Variables

As observed in**Table 3**, positive correlations were established between a greater number of psychiatric comorbidities and severity of depression according to the HDRS (r = 0.36, p <0.01), number of depressive episodes (r = 0.27, p < 0.01), duration of the longest episode (r = 0.12, p <0.05), number of CTEs (r = 0.23, p <0.01), the sum of IPV events (r = 0.22, p <0.01), and the number of recent stressful life events (r = 0.19, p <0.01). On the other hand, inverse correlations were established with age at time of first consultation for depression (r = -0.17, p <0.01), age at time of the first depressive episode (r = - 0.32, p <0.01), and total number of comorbid medical illnesses (r = -0.14, p<0.05) (**Table 3**).

It is important to note that biomedical comorbidity was significantly associated with older age at time of first consultation for depression (r = 0.59, p <0.01) and older age at the time of the first episode (r = 0.36 p <0.01) (**Table 3**).

Finally, using the linear regression analysis (**Table 4**), taking into account only the significant variables that in the simple analysis were correlated with the psychiatric comorbidity, it was established that the youngest age at the time of the first episode, the number of CTEs, and the number of recent stressful events were the most significant factors to best explain the psychiatric comorbidity. In a first model, the youngest age at the time of the first episode explained a large part of the variability (10.4%). A second model, which includes the previous one plus the number of CTEs, explained a total of 12.2% of the variability. Finally, a third model, which includes the previous two plus the total of recent stressful life events, explained a total of 13.6% of the variability. Based on the latter model, it is possible to predict a greater number of psychiatric comorbidities by means of the following formula:

Total psychiatric comorbidities = 2,875 - 0,028 *∗* (age at the first depressive episode) + 0,117 *∗* (total CTEs) + 0,052 *∗* (total recent life stressful events).

From the previous equation it follows that, in those people who have been diagnosed with depression, when the values of the independent variables (age at first depressive episode, traumatic events in childhood, and recent stressful life events) have a value equal to 0, the total psychiatric comorbidity score will be close to 2,875. For each unit that increases the age of onset of the first depressive episode, the psychiatric comorbidity decreases by 0.028 points, while, for each unit that increases the number of traumatic events in childhood, the psychiatric comorbidity will increase 0.117 points and for each unit that increases the number of recent vital events, the psychiatric comorbidity will increase 0.052 points.

## 4. Discussion

The high prevalence of psychiatric comorbidities found in this study is consistent with data from other studies, carried out in similar clinical samples [18, 38, 39]. This result was also associated with a younger age at the onset of depression, a greater number of depressive symptoms, and greater chronicity. These results, obtained from a Latino population, are consistent with current literature [7, 40–42].

Of all the factors analyzed in this study, the earliest age of onset of depressive symptoms was the best predictor of the increase in psychiatric comorbidity. This finding, which is consistent with the current literature [40, 41], was also observed by Davidson and Turnbull in the 80s. In their report, these authors observed that, in comparison with noncomorbid depression, depression secondary to anxiety presented not only an earlier onset of the illness, but also greater severity, chronicity, and less response to tricyclic treatment and electroconvulsive therapy [43]. Other authors have found evidence that atypical depression, or depression with atypical features as it has been known in the DSM-IV, is associated with higher rates social anxiety disorder, earlier age of onset, and higher severity of the illness when compared to nonatypical depression [44].

The evidence that anxiety-depression comorbidity implies a different profile of depressive patients is now included in the DSMV [13, 14]. Current research shows that these patients need a more intensive serotonergic treatment [8]. Considering that anxious comorbidity is underdiagnosed in patients with depression [16, 17], our results give more evidence on the importance of performing an active investigation of anxious symptomatology in PHC depressed patients, mainly when depressive manifestations start early. Evidence shows that social anxiety begins earlier in life, followed by generalized anxiety and panic disorders, and finally by posttraumatic stress disorder [45].

Additionally, in this sample, the highest psychiatric comorbidity was associated with all the forms of interpersonal biographical trauma. In this sample we found a high frequency of CTEs, especially sexual abuse [26, 27, 46]. These backgrounds were the second factor—after younger age at the time of the first depressive episode—that predicted greatest psychiatric comorbidity in this sample. On the other hand, according to the results of this study, the antecedents of intimate partner violence (IPV) and recent stressful life events are factors associated with depression comorbidity that should also be considered.

Regarding the impact of history of CTEs on depression [7, 42, 46], the findings of this study are in the same line of current literature [47–49]. According to the evidence accumulated during the last 30 years [46–49], CTEs constitute a vulnerability factor for development of adult psychopathology such as depression [50]. In addition, patients affected by depression with CTEs often develop complex clinical manifestations, which are characterized by the early onset of depression, presence of psychiatric comorbidity, recurrent MDD, increased suicide risk, and poor response to treatments validated for depression [51–54]. Most of these clinical characteristics were also associated with greater psychiatric comorbidity in this sample.

The clinical complexity presented by patients with adult psychopathology and history of CTEs is an expression of neurobiological changes in epigenetics, neuro-immuno-endocrine systems and neurotransmission, and specific toxicity in brain areas, as a consequence of the exposure to damaging stressful experiences during a window of great susceptibility in the development of the mental system [46–49].

In this sense, according to our data and the current knowledge, it is possible to posit that the association between anxiety-depression comorbidity and early onset of depression may be associated with history of CTEs, which in turn is based on vulnerability to stress. In these patients this vulnerability may favor first the development of anxious symptoms followed by the onset of a depressive disorder.

As said above, for several authors, patients with depression and history of CTEs constitute a different profile of depressive patients who should be recognized in clinical practice [48, 49]. However, as psychiatric comorbidities, traumatic biographies are not routinely investigated when subjects consult for depression [55, 56] and the recognition of the deleterious consequences of child maltreatment is not even incorporated in psychiatric nosology [56].

Based on our data and other studies [6, 46, 51], we posit that psychiatric comorbidity and CTEs are factors that could be present in the same subtype of depressive patients. Additionally, our research team has also found associations between specific CTEs and specific comorbid anxiety disorders in depressed patients: psychological abuse was associated with social phobia, and sexual abuse was associated with posttraumatic stress disorder and panic disorder [27]. Taking into account the above, psychiatric comorbidity and CTEs are factors that should be inquired in PHC depressed patients, especially when depressive manifestations start early. These patients may require differentiated pharmacological treatment and a differentiated integral psychological clinical approach [8].

Another important finding in our study was the fact that, in this sample, we were unable to establish a relationship between biomedical pathology and childhood trauma history. This result was unexpected considering the Adverse Childhood Study (ACES) findings [57–59] and could be explained considering that the instrument used in this research only considered some of the childhood adverse events investigated in the ACES. The lack of association between CTEs and biomedical comorbidities in this sample can also explain the result of this study with respect to the inversely significant relationship between psychiatric comorbidity and biomedical comorbidity.

Among the limitations of this work is the fact that the instrument used to diagnose psychiatric comorbidities punctually investigated current and nonhistorical diagnoses, and it excluded diagnoses such as borderline personality disorder and somatoform disorders. On the other hand, the CTEs were retrospectively investigated and, as already noted, only some adverse events were considered. Childhood negligence must be incorporated in future studies [38].

Among the strengths of this study, we can point out that the diagnostic instruments were administered by professionals with years of experience in mental health, which allowed them to access very confidential information and to perform a comprehensive and standardized evaluation of the different psychiatric comorbidities.

In summary, as the available evidence shows, our results confirm that the high prevalence of psychiatric comorbidity in patients who consult for depression in PHC is associated with a subgroup of patients whose depression occurs with greater severity, earlier onset of depressive illness, chronicity, and exposure to multiple interpersonal stressors since childhood.

Taking into account the fact that current therapeutic guidelines for depression still do not provide a specific indication for recognition and treatment in those patients who present comorbidities [9] and considering that psychiatric comorbidities and psychosocial factors associated are often underdiagnosed and not adequately treated in the PHC [16, 17], this study gives more evidence on the importance to incorporate new strategies, aimed at the recognition and adequate treatment of comorbidities and the factors associated in depressed patients consulting in PHC.

## Figures and Tables

**Table 1:** Sociodemographic and clinical characteristics of sample in 394 primary care patients with depression, Maule region, Chile, 2014.

**Sociodemographic Characteristics**		

	**Mean**	**Standard deviation**

**Age**	47,5 years	15,1

**Sex**	**N**	%

Women	344	86,8

**Scholarship**	**N**	%

Less than high school	156	39.6

High school/partial high school	164	41.6

College/partial college	74	18.8

**Other Features**	**N**	%

Lives with partner	179	45.4

Lives alone	48	12

Employed with incomes	52	13.2

**Clinical Characteristics Of Depression**		
	**Mean**	**Standard deviation**

Age at the first episode (years)	30.7	17.2

Number of depressive episodes	3.64	4.2

Duration of the longest episode (years)	3.58	7.2

Depressive symptoms at baseline HDRS (points)	20	4.6

	**N**	%

History of suicide attempt	138	35

History of previous depression treatment	177	44.9

**Comorbidities**		

**Biomedical**	**N**	%
0	177	45
1	84	21.4
3	74	18,9
More than 3	59	15

**Psychiatric**	**N**	%
0	43	10,9
1	81	20.6
2	80	20.3
3	77	19.5
More than 3	113	28.6

**Adverse Biographical Events**		

**Stressful life events during previous six months **	**N**	%
0	30	7.6
1	83	21.1
2	87	22.1
3	64	16.2
More than 3	136	34.6

**Intimate partner violence events (entire life)**	**N**	%
0	165	41.9
1	25	6.4
2	18	4.7
3 or more	186	47.2

**Childhood trauma events**	**N**	%
0	71	18
1	67	17
2	66	16.7
3	67	17
4 or more	123	31.2

**Table 2:** Frequency of psychiatric comorbidities according to mini in 394 primary care patients with depression, Maule region, Chile, 2014.

**Comorbid diagnosis**	**N**	%
Post traumatic stress disorder (PTSD)	58	14.8

Dysthymia	79	20.2

Panic disorder	115	29.3

Social anxiety disorder	66	16.8

Generalized anxiety disorder (GAD)	100	25.5

Agoraphobia	92	23.5

Obsessive-compulsive disorder	24	6.1

Alcohol abuse	24	6.1

Bulimia	29	7.4

Antisocial personality disorder	22	5.6

**Table 3:** Correlations between psychiatric and medical comorbidities with other variables of the study in 394 primary care patients with depression, Maule region, Chile, 2014.

	**Psychiatric comorbidities**		**Biomedical comorbidities**	
	R	p	R	p
Age at time of first consultation for depression	-0.17	0.01	0.59	0.01

Age at time of the first depressive episode	-0.32	0.01	0.36	0.01

Number of previous depressive episodes	0.34	0.01	0.12	0.01

Duration of the longest depressive episode	0.12	0.05	0.1	NS

Number of biomedical comorbidities	-0.14	0.05	1	-

Number of psychiatric comorbidities	1	-	-0.14	0.01

Number of childhood trauma events	0.23	0.01	-0.03	NS

Number of intimate partner violence events	0.22	0.01	0.07	NS

Number of stressful life events during the last 6 months	0.19	0.01	-0.12	0.05

Severity of depressive symptoms	0.36	0.01	-0.02	NS

NS: no significant value.

**Table 4:** Linear regression model of significant variables that best explain the psychiatric comorbidity in 394 primary care patients with depression, Maule region, Chile, 2014.

Model	R	R^2^	Corrected R^2^	Typical error of the estimation
1	-0.327_ _^a^	0.107	0.104	1.58

2	0.358_ _^b^	0.128	0.122	1.65

3	0.380_ _^c^	0.145	0.136	1.55

a. Predictor variables: (constant), younger age at the time of the first depressive episode.

b. Predictor variables: (constant), younger age at the time of the first depressive episode, number of childhood trauma events.

c. Predictor variables: (constant), younger age at the time of the first depressive episode, number of childhood trauma events, and number of recent stressful life events.

## Data Availability

(1) The database used to support the findings of this study is included in the final form of the project “Factors associated with the different evolutions by patients admitted to GES depression in Primary Care Region VII: following a cohort”, CONICYT FONIS Project SA13/20135. You can access to this form contacting the mail fonis@conicyt.cl. (2) Most of the data used in this article are included in tables in the main article. (3) Also the database used to support the findings of this study is included in PDF within the supplementary information file. (4) Finally the database used to support the findings of this study is available from the corresponding author upon request.

## Supplementary Materials

Supplementary Materials Regarding the supplementary materials submitted with our manuscript, this corresponded to the database of the CONICYT FONIS Project SA13/20135. The data that we used in this paper are part of this project. We send this database in PDF as part of the data availability but this material cannot be published with the manuscript.
