# Supplementary material for: Factors Associated with Psychiatric Comorbidity in Depression Patients in Primary Health Care in Chile
Source: Depress Res Treat. 2018 Oct 1;2018:1701978. doi: 10.1155/2018/1701978 (PMC6188730; doi:10.1155/2018/1701978)
Supplement: Supplementary Materials — Regarding the supplementary materials submitted with our manuscript, this corresponded to the database of the CONICYT FONIS Project SA13/20135. The data that we used in this paper are part of this project. We send this database in PDF as part of the data availability but this material cannot be published with the manuscript. [file 1701978.f1.pdf]

ficha	FACTORES_CLINICOS_PSICOSOCIALES_ASISTENCIALES	edad	edad_4_grupos	edad_2_grupos
1		67	4	2
2		62	3	2
3		72	4	2
4		49	2	1
5		57	3	2
6		52	3	2
7		64	3	2
8		37	2	1

9	53	3	2
10	36	2	1
11	68	4	2
12	50	2	1
13	58	3	2
14	45	2	1
15	62	3	2
16	58	3	2
17	65	3	2

18	24	1	1
19	51	3	2
20	66	4	2
21	60	3	2
22	50	2	1
23	61	3	2
24	45	2	1
25	36	2	1
26	85	4	2

27	30	1	1
28	41	2	1
29	63	3	2
30	50	2	1
31	68	4	2
32	34	2	1
33	66	4	2
34	52	3	2
35	35	2	1

36	25	1	1
37	63	3	2
38	46	2	1
39	45	2	1
40	48	2	1
41	49	2	1
42	65	3	2
43	17	1	1
44	46	2	2

45	34	2	1
46	16	1	1
47	48	2	1
48	48	2	1
49	17	1	1
50	38	2	1
51	35	2	1
52	31	2	1
53	66	4	2

54	43	2	1
55	61	3	2
56	64	3	2
57	32	2	1
58	33	2	1
59	68	4	2
60	41	2	1
61	74	4	2
62	30	1	1

63	74	4	2
64	71	4	2
65	72	4	2
66	64	3	2
67	71	4	2
68	31	2	1
69	69	4	2
70	52	3	2
71	71	4	2

72	64	3	2
73	54	3	2
74	22	1	1
75	92	4	2
76	60	3	2
77	48	2	1
78	41	2	1
79	72	4	2
80	55	3	2

81	48	2	1
82	54	3	2
83	26	1	1
84	58	3	2
85	41	2	1
86	33	2	1
87	63	3	2
88	44	2	1
89	61	3	2

90	34	2	1
91	51	3	2
92	40	2	1
93	23	1	1
94	26	1	1
95	54	3	2
96	32	2	1
97	68	4	2
98	68	4	2

99	24	1	1
100	55	3	2
101	33	2	1
102	52	3	2
103	53	3	2
104	50	2	1
105	44	2	1
106	44	2	1
107	42	2	1

108	37	2	1
109	44	2	1
110	35	2	1
111	48	2	1
112	54	3	2
113	57	3	2
114	40	2	1
115	50	2	1
116	46	2	1

117	49	2	1
118	64	3	2
119	60	3	2
120	39	2	1
121	67	4	2
122	67	4	2
123	42	2	1
124	71	4	2
125	56	3	2

126	34	2	1
127	45	2	1
128	46	2	1
129	25	1	1
130	45	2	1
131	34	2	1
132	64	3	2
133	26	1	1
134	47	2	1

135	66	4	2
136	25	1	1
137	31	2	1
138	51	3	2
139	46	2	1
140	46	2	1
141	32	2	1
142	52	3	2
143	40	2	1

144	41	2	1
145	47	2	1
146	36	2	1
147	41	2	1
148	16	1	1
149	52	3	2
150	71	4	2
151	25	1	1
152	54	3	2

153	53	3	2
154	33	2	1
155	58	3	2
156	25	2	1
157	66	4	2
158	60	3	2
159	54	3	2
160	76	4	2
161	23	1	1

162	39	2	1
163	16	1	1
164	37	2	1
165	24	1	1
166	44	2	1
167	41	2	1
168	58	3	2
169	55	3	2
170	60	3	2

171	49	2	1
172	17	1	1
173	55	3	2
174	62	3	2
175	67	4	2
176	28	1	1
177	58	3	2
178	32	2	1
179	55	3	2

181	52	3	2
183	21	1	1
184	66	4	2
185	27	1	1
186	0	0	0
187	56	3	2
188	55	3	2
189	16	1	1
190	26	1	1

191	30	1	1
192	52	3	2
193	43	2	1
194	47	2	1
195	48	2	1
196	33	2	1
197	52	3	2
198	54	3	2
199	23	1	1

200	56	3	2
201	51	3	2
202	37	2	1
203	26	1	1
204	64	3	2
205	30	1	1
206	41	2	1
207	39	2	1
208	47	2	1

209	28	1	1
210	37	2	1
211	25	1	1
212	64	3	2
213	23	1	1
214	52	3	2
215	52	3	2
216	32	2	1
217	51	3	2

218	18	1	1
219	69	4	2
220	48	3	2
221	41	2	1
222	0	0	0
223	69	4	2
224	79	4	2
225	36	2	1
226	40	2	1

227	66	4	2
228	53	3	2
229	43	2	1
230	26	1	1
231	30	1	1
232	51	3	2
233	66	4	2
234	41	2	1
235	55	3	2

236	25	1	1
237	39	2	1
238	24	1	1
239	44	2	1
240	23	1	1
241	51	3	2
242	48	2	1
243	29	1	1
244	54	3	2

245	49	2	1
246	30	1	1
247	60	3	2
248	55	3	2
249	21	1	1
250	25	1	1
251	50	2	1
252	24	1	1
253	43	2	1

254	49	2	1
255	47	2	1
256	43	2	1
257	60	3	2
258	53	3	2
259	39	2	1
260	42	2	1
261	55	3	2
262	40	2	1

263	33	2	1
264	35	2	1
265	40	2	1
266	48	2	1
267	68	4	2
268	43	2	1
269	67	4	2
270	52	3	2
271	65	3	2

272	62	3	2
273	22	1	1
274	19	1	1
275	73	4	2
276	47	2	1
277	52	3	2
278	50	2	1
279	48	2	1
280	52	3	2

281	68	4	2
282	16	1	1
283	64	3	2
284	67	4	2
285	39	2	1
286	71	4	2
287	64	3	2
288	64	3	2
289	54	3	2

290	37	2	1
291	66	4	2
292	33	2	1
293	32	2	1
294	44	2	1
295	50	2	1
296	32	2	1
297	39	2	1
298	58	3	2

299	22	1	1
300	41	2	1
301	35	2	1
302	55	3	2
303	32	2	1
304	58	3	2
305	59	3	2
306	46	2	1
307	31	2	1

308	32	2	1
309	21	1	1
310	62	3	2
311	71	4	2
312	57	3	2
313	48	2	1
314	75	4	2
315	69	4	2
316	47	2	1

317	46	2	1
318	61	3	2
319	53	3	2
320	46	2	1
321	65	3	2
322	27	1	1
323	44	2	1
324	39	2	1
325	52	3	2

326	32	2	1
331	58	3	2
332	64	3	2
333	49	2	1
334	31	2	1
335	56	3	2
336	52	3	2
337	23	1	1
338	32	2	1

339	81	4	2
340	62	3	2
341	45	2	1
342	55	0	0
343	54	3	2
344	55	3	2
345	33	2	1
346	58	3	2
347	18	1	1

348	22	1	1
349	35	2	1
350	69	4	2
351	63	3	2
352	55	3	2
353	77	4	2
354	68	4	2
355	71	4	2
356	54	3	2

357	72	4	2
358	60	3	2
359	58	3	2
360	15	1	1
361	57	3	2
371	66	4	2
372	62	3	2
373	20	1	1
374	34	2	1

375	30	1	1
376	80	4	2
377	44	2	1
378	61	3	2
379	61	3	2
380	23	1	1
381	59	3	2
382	42	2	1
383	23	1	1

384	57	3	2
385	34	2	1
386	32	2	1
387	48	2	1
391	53	3	2
392	29	1	1
393	76	4	2
394	37	2	1
395	67	4	2

396	52	3	2
397	23	1	1
398	25	1	1
399	59	3	2
400	60	3	2
401	47	2	1
402	73	4	2
403	51	3	2
404	62	3	2

410	46	2	1
411	60	3	2
412	48	2	1
413	58	3	2
414	58	3	2
415	65	3	2
416	30	1	1
422	34	2	1
423	31	2	1

424	60	3	2
425	69	4	2
426	53	3	2
427	33	2	1
428	34	2	1
429	21	1	1
430	60	3	2
431	52	3	2
432	51	3	2

433	60	3	2
434	36	2	1
435			
436	54	3	2
437	20	1	1
438	68	4	2
439	17	1	1
440	40	2	1
461	50	2	1

462	35	2	1
463	23	1	1
464	71	4	2
465	53	3	2
466	48	2	1
467	40	2	1
468	56	3	2
469	35	2	1
470	21	1	1

471	60	3	2
472	39	2	1
473	36	2	1
474	48	2	1
475	75	4	2
476	47	2	1
477	39	2	1
478	58	3	2
479	65	3	2

480	54	3	2
501	46	2	1
502	38	2	1
561	63	3	2
562	48	2	1
600	56	3	2
700	34	2	1
701	41	2	1
702			

704	32	2	1
705			

total_marshall_	marshall_2_grupos	negligencia_marshall1	testigo_alcoholismo_marshall2	castigo_fisico_marshall3
0	1	0	0	0
4	2	0	1	0
1	1	0	1	0
1	1	1	0	0
5	2	1	0	1
2	1	0	1	0
3	2	1	0	1
0	1	0	0	0

3	2	0	1	1
3	2	1	1	0
4	2	1	1	1
2	1	0	1	0
1	1	1	0	0
0	1	0	0	0
0	1	0	0	0
3	2	1	1	0
2	1	0	1	0

7	2	1	1	1
1	1	0	0	1
1	1	0	1	0
0	1	0	0	0
1	1	0	0	0
2	1	1	1	0
1	1	0	1	0
0	1	0	0	0
2	1	0	1	0

2	1	0	1	0
7	2	1	1	1
0	1	0	0	0
5	2	0	1	1
4	2	1	0	1
6	2	1	1	1
0	1	0	0	0
5	2	1	1	0
2	1	1	0	0

0	1	0	0	0
4	2	1	1	0
5	2	1	1	1
7	2	1	1	1
5	2	1	1	1
3	2	0	1	1
3	2	1	0	1
2	1	0	1	0
2	1	1	0	1

0	1	0	0	0
1	1	1	0	0
3	2	0	1	0
7	2	1	1	1
6	2	0	1	1
1	1	0	0	0
1	1	1	0	0
5	2	0	1	1
3	2	0	0	1

5	2	1	1	1
6	2	1	1	1
1	1	0	0	0
0	1	0	0	0
0	1	0	0	0
3	2	1	1	0
3	2	0	1	1
2	1	1	1	0
1	1	0	1	0

4	2	0	1	0
6	2	1	1	1
3	2	0	1	1
0	1	0	0	0
0	1	0	0	0
2	1	1	1	0
1	1	1	0	0
5	2	0	1	1
0	1	0	0	0

4	2	1	0	1
5	2	0	1	1
4	2	1	0	1
3	2	0	0	1
5	2	1	1	1
6	2	1	1	1
0	1	0	0	0
3	2	0	0	1
1	1	0	1	0

3	2	1	0	1
5	2	1	1	1
4	2	1	1	1
2	1	1	0	1
3	2	1	0	1
4	2	1	1	1
1	1	0	1	0
4	2	1	1	1
5	2	1	1	1

0	1	0	0	0
0	1	0	0	0
2	1	0	1	0
2	1	1	1	0
2	1	0	0	1
3	2	0	1	1
2	1	1	0	0
3	2	1	0	1
0	1	0	0	0

1	1	1	0	0
3	2	1	0	1
0	1	0	0	0
2	1	0	0	1
2	1	1	1	0
6	2	1	1	1
0	1	0	0	0
0	1	0	0	0
6	2	1	1	1

3	2	0	1	1
2	1	0	0	1
1	1	0	0	0
2	1	0	1	0
3	2	1	0	1
4	2	1	1	0
4	2	1	0	1
0	1	0	0	0
6	2	1	1	1

0	1	0	0	0
0	1	0	0	0
1	1	0	1	0
3	2	0	1	0
3	2	0	0	1
4	2	0	1	1
5	2	0	1	1
1	1	0	1	0
0	1	0	0	0

3	2	1	1	0
4	2	1	1	0
3	2	0	0	1
1	1	0	0	0
4	2	1	1	0
5	2	0	1	1
2	1	1	1	0
0	1	0	0	0
2	1	0	1	0

4	2	0	0	1
4	2	1	1	0
2	1	0	1	0
6	2	1	1	1
2	1	0	0	1
5	2	1	1	0
3	2	0	1	1
0	1	0	0	0
3	2	1	1	1

4	2	0	1	1
0	1	0	0	0
5	2	1	1	1
1	1	1	0	0
3	2	1	1	0
6	2	1	1	1
1	1	1	0	0
2	1	0	1	0
0	1	0	0	0

3	2	1	0	1
1	1	0	0	0
2	1	0	1	0
0	1	0	0	0
4	2	1	0	1
0	1	0	0	0
5	2	1	1	1
3	2	1	1	0
5	2	1	1	1

1	1	1	0	0
1	1	0	0	0
1	1	1	0	0
5	2	1	1	1
2	1	0	1	0
0	1	0	0	0
4	2	1	1	1
3	2	0	1	1
1	1	0	0	0

0	1	0	0	0
5	2	1	1	1
6	2	0	1	1
4	2	1	0	1
0	1	0	0	0
2	1	0	1	0
0	1	0	0	0
0	1	0	0	0
6	2	1	1	1

6	2	1	1	1
1	1	0	0	0
4	2	1	1	1
4	2	1	1	0
5	2	1	1	1
4	2	1	1	1
1	1	1	0	0
4	2	1	0	1
1	1	1	0	0

2	1	0	1	0
1	1	0	1	0
3	2	0	1	0
2	1	0	1	0
0	1	0	0	0
3	2	0	0	1
5	2	1	1	1
2	1	1	0	1
2	1	1	0	0

5	2	1	1	1
4	2	1	0	1
4	2	1	1	1
5	2	0	1	1
2	1	0	1	0
3	2	0	1	1
3	2	0	0	1
3	2	1	1	0
2	1	1	1	0

1	1	0	1	0
0	1	0	0	0
0	1	0	0	0
1	1	0	0	1
2	1	0	0	1
2	1	0	1	0
2	1	1	0	0
1	1	0	0	0
5	2	0	1	1

3	2	1	0	1
1	1	1	0	0
5	2	1	1	1
2	1	0	1	0
0	1	0	0	0
3	2	0	0	1
4	2	0	0	1
0	1	0	0	0
3	2	1	0	0

0	1	0	0	0
3	2	1	1	1
0	1	0	0	0
0	1	0	0	0
3	2	0	1	1
4	2	0	1	1
1	1	0	0	0
3	2	0	1	1
1	1	0	1	0

2	1	0	1	0
5	2	1	1	1
4	2	1	0	1
2	1	0	1	1
3	2	0	0	1
4	2	0	1	1
3	2	0	1	1
1	1	0	0	0
0	1	0	0	0

3	2	1	0	1
1	1	1	0	0
4	2	1	1	1
2	1	0	1	0
3	2	1	1	0
0	1	0	0	0
0	1	0	0	0
1	1	1	0	0
4	2	1	0	1

1	1	0	1	0
0	1	0	0	0
3	2	1	1	0
1	1	0	0	0
1	1	0	1	0
3	2	1	0	1
6	2	1	1	1
0	1	0	0	0
0	1	0	0	0

1	1	0	0	0
4	2	1	1	0
4	2	1	0	1
1	1	0	0	0
4	2	0	1	1
0	1	0	0	0
2	1	0	0	1
4	2	1	0	1
3	2	1	0	1

3	2	1	1	1
4	2	1	1	1
0	1	0	0	0
3	2	0	1	1
3	2	1	1	0
4	2	1	1	1
5	2	1	1	1
3	2	1	1	0
4	2	1	1	1

0	1	0	0	0
0	1	0	0	0
2	1	0	1	0
1	1	0	0	1
2	1	1	0	1
4	2	0	1	1
4	2	1	0	1
2	1	1	0	1
3	2	1	1	0

2	1	0	0	1
2	1	1	0	0
3	2	1	0	1
3	2	0	0	1
4	2	1	1	0
1	1	1	0	0
6	2	1	1	1
1	1	0	0	0
1	1	1	0	0

3	2	1	0	0
2	1	0	1	0
5	2	1	0	1
1	1	1	0	0
2	1	1	0	0
2	1	1	1	0
3	2	1	1	0
6	2	1	1	1
2	1	0	0	0

4	2	1	0	1
3	2	0	1	1
2	1	1	0	1
2	1	0	1	0
0	1	0	0	0
4	2	0	1	0
1	1	0	0	1
4	2	0	0	1
1	1	0	0	0

5	2	1	1	1
6	2	1	1	1
4	2	1	1	0
6	2	0	1	1
3	2	1	0	1
1	1	1	0	0
6	2	1	1	1
1	1	1	0	0
6	2	1	1	1

5	2	1	1	1
0	1	0	0	0
0	1	0	0	0
1	1	0	0	0
3	2	0	1	1
2	1	1	0	0
5	2	1	1	0
3	2	1	0	1
1	1	1	0	0

1	1	1	0	0
3	2	0	1	0
1	1	0	0	0
7	2	1	1	1
5	2	1	0	1
6	2	1	1	1
3	2	1	0	1
5	2	0	1	1
2	1	0	0	1

1	1	0	0	0
0	1	0	0	0
0	1	0	0	0
4	2	1	1	0
1	1	1	0	0
1	1	0	1	0
4	2	0	1	1
2	1	0	1	0
0	1	0	0	0

5	2	1	1	1
0	1	0	0	0
5	2	1	1	1
6	2	0	1	1
3	2	1	1	1
1	1	0	1	0
2	1	1	1	0
1	1	0	0	0
1	1	1	0	0

1	1	0	0	0
4	2	0	1	1
3	2	0	0	1
1	1	1	0	0
3	2	1	1	0
2	1	1	0	1
1	1	0	1	0
3	2	1	0	1
2	1	1	0	1

2	1	1	1	0
3	2	1	1	0
2	1	1	0	0
4	2	1	1	1
2	1	0	1	0
4	2	0	1	1
1	1	1	0	0
0	1	0	0	0
3	2	1	0	1

6	2	1	1	1
5	2	1	0	1
2	1	0	1	0
3	2	1	0	1
0	1	0	0	0
6	2	1	1	1
0	1	0	0	0
0	1	0	0	0
5	2	1	1	1

0	1	0	0	0
3	2	0	0	1
0	1	0	0	0
2	1	0	0	1
5	2	0	0	1
0	1	0	0	0
4	2	1	1	1
6	2	1	0	1
1	1	0	0	1

1	1	1	0	0
3	2	1	1	1
3	2	1	0	0
0	1	0	0	0
1	1	0	0	0
4	2	1	0	1
1	1	1	0	0
0	1	0	0	0
5	2	0	1	1

0	1	0	0	0
5	2	1	1	1
0	1	0	0	0
3	2	0	1	1
2	1	0	0	1
1	1	0	0	0
2	1	0	0	1
3	2	0	0	1
1	1	1	0	0

1	1	1	0	0
0	1	0	0	0
1	1	1	0	0
5	2	1	1	1
7	2	1	1	1
1	1	1	0	0
4	2	1	1	0
3	2	1	1	0
3	2	1	1	0

1	1	0	1	0
4	2	0	1	1
0	1	0	0	0
2	1	0	0	0
0	1	0	0	0
1	1	0	1	0
2	1	0	1	0
0	1	0	0	0
0	1	0	0	0

0	1	0	0	0
1	1	0	0	0
2	1	1	1	0
0	1	0	0	0
5	2	1	0	1
5	2	1	1	1
2	1	0	1	0
5	2	0	1	1
2	1	1	0	0

4	2	0	1	1

secuela_castigo_fisico_marshall4	testigo_vif_marshall5	abuso_sexual_fliar_marshall6	abuso_sexual_no_fliar_marshall7	mujeres
0	0	0	0	1
0	1	1	1	1
0	0	0	0	1
0	0	0	0	0
1	1	1	0	1
0	1	0	0	0
0	0	1	0	1
0	0	0	0	1

1	0	0	0	1
0	0	0	1	1
0	1	0	0	1
0	0	1	0	1
0	0	0	0	1
0	0	0	0	1
0	0	0	0	1
0	1	0	0	1
0	0	1	0	1

1	1	1	1	1
0	0	0	0	1
0	0	0	0	1
0	0	0	0	1
0	0	0	1	1
0	0	0	0	1
0	0	0	0	1
0	0	0	0	1
0	1	0	0	1

0	1	0	0	1
1	1	1	1	1
0	0	0	0	1
1	1	1	0	1
1	0	1	0	1
1	1	1	0	1
0	0	0	0	1
0	1	1	1	1
0	0	0	1	1

0	0	0	0	0
0	1	1	0	1
1	0	0	1	1
1	1	1	1	1
1	1	0	0	1
0	1	0	0	0
1	0	0	0	1
0	1	0	0	1
0	0	0	0	1

0	0	0	0	1
0	0	0	0	0
0	1	1	0	1
1	1	1	1	1
1	1	1	1	1
0	0	0	1	1
0	0	0	0	1
1	1	0	1	1
1	1	0	0	1

0	1	0	1	1
1	1	1	0	1
0	1	0	0	1
0	0	0	0	1
0	0	0	0	1
0	1	0	0	1
0	1	0	0	1
0	0	0	0	1
0	0	0	0	1

0	1	1	1	1
0	1	1	1	0
0	1	0	0	1
0	0	0	0	1
0	0	0	0	1
0	0	0	0	1
0	0	0	0	1
0	1	1	1	1
0	0	0	0	1

1	1	0	0	1
1	1	0	1	1
1	1	0	0	1
1	1	0	0	1
1	1	0	0	1
1	1	0	1	1
0	0	0	0	1
1	1	0	0	1
0	0	0	0	1

0	0	0	1	1
0	1	0	1	1
0	0	0	1	1
0	0	0	0	1
0	0	1	0	1
0	1	0	0	1
0	0	0	0	1
0	1	0	0	1
0	1	1	0	1

0	0	0	0	1
0	0	0	0	1
0	1	0	0	1
0	0	0	0	1
0	0	0	1	1
0	1	0	0	0
0	0	0	1	0
0	1	0	0	1
0	0	0	0	1

0	0	0	0	1
1	0	0	0	1
0	0	0	0	1
0	1	0	0	1
0	0	0	0	1
1	1	1	0	1
0	0	0	0	1
0	0	0	0	1
1	1	1	0	1

0	1	0	0	1
0	0	1	0	1
0	1	0	0	1
0	1	0	0	1
1	0	0	0	1
0	1	0	1	1
0	1	1	0	1
0	0	0	0	1
1	1	1	0	1

0	0	0	0	1
0	0	0	0	1
0	0	0	0	0
0	1	1	0	1
1	1	0	0	1
0	1	1	0	1
1	1	1	0	0
0	0	0	0	0
0	0	0	0	1

0	1	0	0	1
0	1	0	1	1
1	1	0	0	1
0	1	0	0	1
0	1	1	0	1
1	1	1	0	1
0	0	0	0	1
0	0	0	0	1
0	1	0	0	1

1	0	1	1	1
0	1	1	0	1
0	1	0	0	1
1	1	1	0	1
1	0	0	0	1
0	1	1	1	1
0	1	0	0	0
0	0	0	0	1
0	0	0	0	1

1	1	0	0	1
0	0	0	0	1
0	1	1	0	1
0	0	0	0	1
0	1	0	0	1
1	1	0	1	1
0	0	0	0	1
0	0	1	0	1
0	0	0	0	1

0	0	0	1	1
0	0	0	1	1
0	1	0	0	1
0	0	0	0	1
1	1	0	0	1
0	0	0	0	1
1	1	0	0	1
0	1	0	0	0
1	0	1	0	1

0	0	0	0	1
0	1	0	0	0
0	0	0	0	1
1	1	0	0	1
0	1	0	0	1
0	0	0	0	1
0	1	0	0	1
0	0	1	0	1
0	1	0	0	1

0	0	0	0	1
1	1	0	0	1
1	1	1	1	1
1	0	0	1	1
0	0	0	0	1
0	1	0	0	1
0	0	0	0	1
0	0	0	0	1
1	1	0	1	1

1	1	1	0	1
0	1	0	0	1
0	1	0	0	0
0	1	1	0	1
0	1	0	1	1
0	1	0	0	1
0	0	0	0	1
0	1	1	0	1
0	0	0	0	1

0	1	0	0	1
0	0	0	0	1
0	1	0	1	1
0	1	0	0	1
0	0	0	0	1
0	1	1	0	1
0	1	1	0	0
0	0	0	0	1
0	0	0	1	1

1	1	0	0	1
1	0	1	0	1
0	0	0	1	1
1	1	1	0	1
0	1	0	0	1
1	0	0	0	1
1	1	0	0	1
0	1	0	0	1
0	0	0	0	1

0	0	0	0	1
0	0	0	0	1
0	0	0	0	1
0	0	0	0	0
1	0	0	0	1
0	1	0	0	1
0	0	0	1	1
0	0	0	1	1
0	1	1	1	1

1	0	0	0	1
0	0	0	0	1
1	1	0	0	1
0	1	0	0	0
0	0	0	0	1
1	0	0	1	1
1	0	1	1	1
0	0	0	0	0
0	1	1	0	1

0	0	0	0	1
0	0	0	0	1
0	0	0	0	1
0	0	0	0	0
0	1	0	0	1
0	1	0	1	1
0	1	0	0	0
0	1	0	0	1
0	0	0	0	1

0	1	0	0	1
1	1	0	0	0
1	1	0	0	1
0	0	0	0	1
0	0	1	1	1
0	1	1	0	1
1	0	0	0	1
0	0	0	1	0
0	0	0	0	1

1	0	0	0	1
0	0	0	0	1
0	0	1	0	1
0	1	0	0	1
0	1	0	0	1
0	0	0	0	1
0	0	0	0	0
0	0	0	0	1
0	1	1	0	1

0	0	0	0	0
0	0	0	0	1
0	1	0	0	1
0	1	0	0	0
0	0	0	0	0
0	1	0	0	1
1	1	0	1	1
0	0	0	0	1
0	0	0	0	1

0	1	0	0	1
0	1	1	0	1
1	1	0	0	0
0	1	0	0	1
1	1	0	0	1
0	0	0	0	1
1	0	0	0	1
1	1	0	0	1
0	0	0	1	1

0	0	0	0	1
0	1	0	0	1
0	0	0	0	1
0	1	0	0	0
0	1	0	0	1
0	1	0	0	1
0	1	0	1	1
0	1	0	0	1
0	1	0	0	1

0	0	0	0	1
0	0	0	0	1
0	1	0	0	1
0	0	0	0	1
0	0	0	0	1
1	1	0	0	0
1	1	0	0	1
0	0	0	0	1
0	1	0	0	1

0	0	0	1	1
0	0	1	0	1
0	1	0	0	1
1	1	0	0	1
0	1	0	1	0
0	0	0	0	0
0	1	1	1	1
0	0	1	0	1
0	0	0	0	1

0	1	1	0	1
0	0	0	1	1
1	1	1	0	1
0	0	0	0	1
0	1	0	0	1
0	0	0	0	1
0	1	0	0	1
1	1	1	0	1
0	1	1	0	1

0	1	1	0	1
0	1	0	0	1
0	0	0	0	0
0	1	0	0	1
0	0	0	0	0
0	1	1	1	1
0	0	0	0	0
1	0	1	1	1
0	1	0	0	1

1	1	0	0	1
1	1	0	1	1
0	1	0	1	1
1	1	1	1	1
0	0	0	1	0
0	0	0	0	1
1	1	1	0	1
0	0	0	0	1
1	1	1	0	1

1	0	1	0	1
0	0	0	0	1
0	0	0	0	1
0	1	0	0	0
0	1	0	0	1
0	0	1	0	1
0	1	1	1	1
0	1	0	0	1
0	0	0	0	1

0	0	0	0	1
0	0	1	1	0
0	0	0	1	1
1	1	1	1	1
1	1	1	0	1
1	1	0	1	1
0	1	0	0	1
1	1	0	1	1
1	0	0	0	0

0	1	0	0	1
0	0	0	0	1
0	0	0	0	1
0	1	1	0	1
0	0	0	0	1
0	0	0	0	1
1	1	0	0	1
0	1	0	0	1
0	0	0	0	1

1	1	0	0	1
0	0	0	0	1
1	1	0	0	1
1	1	1	1	1
0	0	0	0	0
0	0	0	0	0
0	0	0	0	0
0	0	0	1	1
0	0	0	0	1

0	1	0	0	1
1	1	0	0	1
1	0	0	1	1
0	0	0	0	1
0	1	0	0	1
0	0	0	0	1
0	0	0	0	1
0	1	0	0	1
0	0	0	0	1

0	0	0	0	0
0	1	0	0	1
0	1	0	0	1
0	1	0	0	1
0	0	1	0	1
1	1	0	0	1
0	0	0	0	1
0	0	0	0	1
1	0	0	0	1

1	1	1	0	0
1	1	1	0	1
0	1	0	0	1
1	0	0	0	1
0	0	0	0	0
1	1	0	1	1
0	0	0	0	1
0	0	0	0	1
1	1	0	0	1

0	0	0	0	1
1	0	0	1	1
0	0	0	0	1
0	0	0	1	0
1	1	1	1	0
0	0	0	0	1
0	0	0	1	0
1	1	1	1	1
0	0	0	0	1

0	0	0	0	1
0	0	0	0	0
0	1	1	0	0
0	0	0	0	1
0	0	0	1	1
1	1	0	0	0
0	0	0	0	1
0	0	0	0	0
1	0	1	1	1

0	0	0	0	1
1	1	0	0	1
0	0	0	0	1
0	1	0	0	1
0	1	0	0	1
0	0	1	0	1
0	1	0	0	0
0	1	1	0	1
0	0	0	0	1

0	0	0	0	1
0	0	0	0	1
0	0	0	0	0
1	1	0	0	1
1	1	1	1	1
0	0	0	0	1
0	1	0	1	1
0	1	0	0	1
0	1	0	0	0

0	0	0	0	1
1	1	0	0	1
0	0	0	0	1
0	1	1	0	1
0	0	0	0	1
0	0	0	0	1
0	1	0	0	0
0	0	0	0	0
0	0	0	0	1

0	0	0	0	1
0	0	1	0	1
0	0	0	0	1
0	0	0	0	1
1	1	0	1	0
0	1	0	1	1
0	1	0	0	1
1	1	1	0	1
0	1	0	0	1

1	1	0	0	1

toma_medicam_segun_indicacion	tto_depresion_anterior	riesgo_suicidio	comorbilidad_psiquiatrica_sin_suicidio	comorbilidad_2_grupos
0	1	0	2	1
1	1	2	2	1
1	1	0	2	1
1	0	0	0	1
1	0	0	3	2
1	0	1	1	1
1	0	1	2	1
1	1	0	2	1

1	1	0	2	1
1	1	2	2	1
1	1	0	1	1
1	0	1	4	2
1	0	1	1	1
1	0	0	1	1
1	1	0	2	1
1	0	2	2	1
0	1	1	1	1

1	1	0	6	2
1	0	1	2	1
1	1	0	1	1
1	1	2	5	2
0	1	1	3	2
1	0	0	3	2
1	0	1	1	1
0	0	0	0	1
1	1	0	1	1

1	1	2	6	2
1	0	1	4	2
1	0	0	1	1
1	1	1	3	2
1	1	2	1	1
1	0	0	2	1
1	1	0	2	1
1	1	0	3	2
1	1	2	4	2

1	0	0	3	2
1	0	0	3	2
0	1	2	1	1
1	0	0	2	1
1	1	0	1	1
1	0	0	1	1
1	1	0	4	2
1	0	0	1	1
1	1	1	1	1

1	1	0	1	1
1	0	0	1	1
1	1	0	2	1
1	0	1	4	2
0	0	2	0	1
1	0	1	0	1
1	1	2	3	2
1	1	2	5	2
1	1	2	1	1

1	1	1	3	2
1	1	0	1	1
1	0	0	3	2
0	0	0	2	1
1	0	0	0	1
1	1	0	1	1
0	0	0	4	2
0	1	0	2	1
1	0	2	4	2

1	1	0	2	1
1	0	2	1	1
1	0	2	2	1
1	0	0	3	2
1	0	0	1	1
1	1	2	5	2
1	0	0	1	1
1	0	3	3	2
1	0	0	0	1

1	0	2	0	1
1	0	2	0	1
0	0	3	1	1
1	0	2	1	1
1	0	2	2	1
1	1	3	6	2
0	1	1	1	1
1	0	0	1	1
1	1	1	3	2

1	1	1	1	1
0	1	2	3	2
0	1	1	4	2
0	0	0	0	1
1	1	2	1	1
1	1	2	2	1
0	0	0	0	1
1	1	3	3	2
1	1	2	1	1

1	0	2	1	1
0	0	2	3	2
1	1	0	1	1
0	1	0	0	1
1	0	2	5	2
1	1	0	2	1
1	1	1	2	1
1	1	1	3	2
1	1	2	4	2

0	0	0	2	1
0	1	2	4	2
0	0	0	1	1
1	0	0	4	2
1	1	2	3	2
0	0	2	4	2
1	0	0	1	1
1	0	2	3	2
1	0	3	9	2

0	0	0	0	1
1	1	0	1	1
1	0	0	5	2
1	1	2	7	2
1	0	1	1	1
0	1	3	4	2
1	0	0	1	1
0	1	0	3	2
0	1	2	4	2

1	0	0	2	1
1	1	0	2	1
1	1	2	4	2
1	1	1	4	2
1	0	0	1	1
1	1	0	3	2
0	1	1	2	1
1	0	2	4	2
1	1	0	3	2

0	0	1	1	1
1	0	1	5	2
0	1	2	3	2
1	0	2	3	2
1	1	0	3	2
1	0	2	5	2
1	1	0	3	2
1	0	0	1	1
1	0	0	1	1

1	0	2	3	2
1	0	2	2	1
0	1	0	1	1
1	0	0	3	2
1	1	2	2	1
1	0	0	2	1
1	0	0	1	1
1	1	0	3	2
1	0	0	1	1

1	0	0	1	1
1	0	1	0	1
1	0	1	3	2
1	0	0	2	1
1	0	0	2	1
1	1	1	2	1
1	1	1	1	1
1	0	2	4	2
1	1	1	2	1

1	1	0	2	1
1	0	0	0	1
1	0	0	1	1
0	0	0	2	1
1	0	0	1	1
1	1	0	4	2
1	0	2	2	1
1	0	0	0	1
1	1	3	5	2

0	1	1	4	2
1	0	0	0	1
1	0	1	1	1
1	0	1	6	2
1	0	1	2	1
1	0	0	1	1
1	1	0	2	1
0	1	2	1	1
0	0	1	3	2

1	0	0	0	1
0	0	0	2	1
0	1	2	3	2
1	1	2	2	1
1	0	2	2	1
0	1	2	3	2
0	0	0	0	1
1	0	0	3	2
0	1	0	4	2

0	1	3	3	2
0	0	1	2	1
0	1	0	0	1
1	1	2	3	2
1	1	0	2	1
0	1	2	1	1
1	0	1	1	1
0	0	2	4	2
1	1	2	3	2

1	0	1	3	2
1	0	1	1	1
0	1	0	4	2
1	1	2	2	1
1	1	0	2	1
0	0	0	1	1
1	0	0	3	2
1	0	0	2	1
0	0	2	5	2

1	1	3	5	2
0	1	1	3	2
1	0	1	3	2
1	1	0	2	1
1	1	1	1	1
1	0	0	0	1
1	0	0	1	1
1	1	0	3	2
1	0	0	4	2

0	0	0	1	1
1	0	0	1	1
1	1	3	3	2
1	0	0	0	1
1	0	2	7	2
0	1	2	5	2
1	1	1	3	2
1	1	2	2	1
1	1	2	7	2

1	0	0	2	1
1	1	0	0	1
0	1	2	10	2
0	0	0	1	1
0	0	2	0	1
1	1	0	1	1
1	1	0	1	1
1	1	3	3	2
1	1	3	4	2

1	1	0	1	1
1	1	3	3	2
1	0	2	2	1
1	0	3	0	1
1	1	0	3	2
1	1	2	1	1
1	0	1	3	2
1	0	2	7	2
1	0	2	0	1

0	0	0	2	1
1	0	1	3	2
1	0	0	1	1
1	1	0	3	2
1	1	2	8	2
1	0	1	2	1
1	1	1	2	1
1	0	1	2	1
1	1	0	0	1

1	1	0	1	1
0	0	0	0	1
1	0	2	1	1
1	1	1	4	2
1	1	0	1	1
1	0	0	2	1
1	0	0	1	1
1	1	2	5	2
0	0	0	1	1

1	0	0	2	1
1	0	0	3	2
0	0	0	3	2
1	0	1	4	2
1	0	0	3	2
1	0	0	3	2
0	1	2	2	1
1	1	0	2	1
1	0	0	1	1

1	1	0	0	1
0	0	2	0	1
0	0	3	1	1
1	0	0	3	2
0	0	0	0	1
1	0	2	1	1
1	0	2	2	1
1	0	2	0	1
1	0	2	1	1

1	0	1	0	1
0	0	2	1	1
0	0	0	1	1
0	0	0	0	1
1	0	1	2	1
0	1	2	2	1
0	1	2	2	1
1	0	2	5	2
1	0	1	1	1

1	1	1	3	2
1	0	0	2	1
1	1	0	1	1
1	1	0	1	1
0	1	1	2	1
1	1	0	3	2
1	1	0	2	1
1	1	1	1	1
0	1	0	0	1

1	1	1	4	2
1	1	0	6	2
0	0	2	4	2
1	0	1	5	2
1	0	0	2	1
1	0	0	2	1
1	0	3	6	2
0	0	1	1	1
1	0	0	1	1

0	0	2	1	1
0	1	0	6	2
0	0	0	3	2
1	1	0	7	2
1	0	1	4	2
1	1	1	3	2
1	1	2	3	2
1	0	2	4	2
1	1	0	5	2

1	1	2	5	2
1	1	1	4	2
1	0	0	0	1
1	1	0	3	2
1	0	0	3	2
1	0	2	0	1
1	0	1	1	1
1	1	0	2	1
1	1	2	2	1

1	0	1	3	2
1	1	2	3	2
1	0	2	2	1
1	0	0	4	2
1	0	0	0	1
1	0	3	5	2
0	1	1	4	2
1	0	2	0	1
1	1	2	2	1

1	1	0	1	1
1	1	0	2	1
1	0	1	1	1
0	0	0	1	1
1	1	2	4	2
1	0	0	4	2
0	0	1	3	2
1	0	0	0	1
1	0	1	2	1

1	0	0	4	2
1	0	2	0	1
0	0	1	5	2
0	1	0	6	2
1	1	2	6	2
1	1	2	6	2
1	1	0	3	2
1	1	2	4	2
1	0	0	3	2

1	0	0	2	1
1	0	0	1	1
1	0	0	0	1
1	0	0	0	1
1	0	0	0	1
0	0	0	2	1
0	0	0	1	1
1	1	0	2	1
1	1	2	3	2

0	0	0	2	1
1	1	0	0	1
1	0	3	2	1
0	0	3	1	1
1	0	1	0	1
1	0	0	0	1
1	1	0	0	1
1	1	3	6	2
1	0	1	2	1

0	1	2	4	2
1	0	0	0	1
1	1	0	2	1
1	1	0	2	1
1	0	0	1	1
1	0	0	2	1
1	1	0	1	1
1	0	1	2	1
0	0	2	2	1

0	0	0	2	1
1	0	1	3	2
1	0	2	5	2
1	0	1	3	2
1	0	0	6	2
0	1	3	2	1
0	1	0	1	1
0	0	0	0	1
1	1	0	2	1

0	0	1	0	1
0	0	0	1	1
1	0	0	0	1
1	1	0	1	1
1	0	0	3	2
1	1	3	5	2
1	0	0	0	1
1	0	0	1	1
1	1	1	2	1

1	1	2	1	1
1	1	0	4	2
1	1	1	4	2
1	1	2	4	2
1	1	2	6	2
1	0	0	1	1
1	0	2	4	2
1	0	0	6	2
0	0	0	2	1

1	1	0	2	1
1	1	0	2	1
1	0	2	2	1
1	1	0	4	2
0	1	2	2	1
1	1	0	1	1
1	1	0	1	1
1	0	0	1	1
1	0	0	4	2

1	1	0	4	2
1	1	0	2	1
1	1	0	2	1
1	0	0	1	1
0	0	0	2	1
0	0	0	0	1
1	0	0	1	1
1	0	0	3	2
0	1	0	1	1

1	0	0	0	1
1	0	1	0	1
1	0	0	0	1
1	1	1	3	2
1	1	1	2	1
1	1	1	0	1
1	1	3	2	1
1	0	2	3	2
1	1	0	4	2

1	0	0	2	1
1	1	2	4	2
0	0	0	0	1
1	1	0	0	1
1	1	0	2	1
1	0	1	1	1
0	0	2	2	1
1	0	0	0	1
1	0	0	0	1

0	0	0	0	1
0	0	2	1	1
1	0	0	1	1
1	0	2	2	1
1	1	1	4	2
1	1	3	1	1
1	0	0	0	1
1	1	2	4	2
1	1	0	1	1

0	1	0	0	0

EPISODIO_DEPRESION	EPI_DEPRES_RECU	DISTIMIA	TRASTORNO_DISTIMICO	TRASTORNO_DISTIMICO_RECU
1	1	1	0	1
1	1	0	1	0
1	1	0	1	0
1	0	1	1	0
1	0	1	1	0
1	0	1	1	0
0	0	0	1	0
1	1	0	1	0

1	1	1	1	0
1	1	1	1	0
1	1	0	0	0
1	1	0	0	1
1	1	0	1	0
1	1	0	1	0
0	0	0	1	0
1	1	1	1	0
1	1	0	1	0

1	1	1	0	1
1	1	0	0	1
0	0	0	0	0
1	1	0	0	0
1	1	0	1	0
1	1	0	1	0
1	1	1	1	0
0	0	0	1	0
1	1	0	0	0

1	1	0	0	0
1	1	1	0	1
1	1	0	0	0
1	1	1	0	1
1	1	0	0	0
1	1	0	1	0
1	1	0	0	1
1	1	0	0	1
1	1	1	1	0

0	0	1	0	0
1	1	0	0	1
1	1	0	0	0
1	1	0	0	1
1	1	0	0	0
1	0	1	0	0
0	1	0	0	0
0	0	0	0	0
0	1	1	1	0

1	1	0	1	0
1	0	1	1	0
1	1	1	1	0
1	1	0	0	0
1	0	0	0	0
1	0	0	1	0
1	1	0	1	0
1	0	0	0	1
0	0	0	0	0

1	1	1	1	0
1	0	0	0	0
0	0	0	0	1
0	0	0	0	0
1	0	0	1	0
0	0	1	0	1
0	1	0	0	0
0	0	0	1	0
1	1	0	0	1

0	0	0	0	1
1	0	0	1	0
1	1	1	0	1
0	0	0	1	0
1	1	0	0	0
1	1	0	0	1
1	0	0	0	1
1	1	1	1	0
1	0	0	0	0

0	0	0	1	0
0	0	0	1	0
1	0	0	1	0
1	0	0	0	0
0	1	1	1	0
1	1	1	1	0
1	1	0	1	0
1	0	0	0	0
1	1	0	0	0

0	1	1	1	0
1	1	0	0	1
1	1	1	1	0
0	0	0	1	0
1	1	0	1	0
1	1	0	1	0
1	0	0	1	0
1	1	0	1	0
1	1	1	1	0

1	1	0	0	0
1	1	0	0	0
1	1	0	1	0
1	0	1	0	0
1	1	0	1	0
1	1	0	0	0
1	1	0	0	0
1	1	0	1	0
1	1	0	1	0

1	1	0	1	0
1	1	0	0	1
1	1	0	0	0
1	1	0	0	1
1	1	0	0	1
1	1	0	0	1
0	0	0	0	1
1	1	1	1	0
1	1	0	0	1

0	0	0	0	0
0	0	0	0	0
1	1	0	0	1
1	1	1	1	0
0	0	0	1	0
1	1	0	0	1
0	1	0	1	0
1	1	0	1	0
1	1	0	1	0

1	0	1	1	0
1	1	0	1	0
1	1	0	1	0
1	1	0	0	1
1	0	1	0	1
1	1	0	0	1
1	1	0	1	0
1	1	1	1	0
1	1	0	0	0

1	0	1	0	1
1	1	1	0	1
1	1	0	1	0
1	1	0	1	0
1	1	0	1	0
1	1	1	1	0
1	1	0	0	1
1	0	0	0	0
1	0	0	0	0

1	1	0	1	0
1	1	0	0	1
1	1	0	0	0
1	1	0	0	1
1	1	0	1	0
1	0	1	1	0
0	0	0	0	0
0	1	0	0	0
1	0	1	0	0

0	0	0	0	0
1	0	0	1	0
0	1	1	0	0
0	0	0	1	0
0	0	0	0	0
1	1	1	1	0
1	1	0	1	0
1	1	0	0	0
1	1	0	1	0

1	1	0	0	0
0	0	0	1	0
0	0	0	0	0
0	0	0	0	0
1	1	0	0	0
1	1	0	0	0
0	0	0	0	1
0	0	0	0	0
1	1	0	0	0

1	1	1	1	0
1	0	0	1	0
1	1	1	1	0
1	1	0	0	0
1	1	0	0	0
1	0	0	0	0
1	1	0	0	0
1	1	0	0	0
1	1	0	0	1

1	0	0	0	0
1	0	0	0	0
1	0	0	1	0
1	1	0	0	0
1	1	1	0	0
1	1	0	0	0
1	0	0	0	0
1	1	1	0	1
0	1	1	1	0

1	1	0	0	0
1	1	0	0	0
0	0	0	0	0
1	1	0	0	0
0	0	0	0	1
1	1	0	0	0
1	1	0	1	0
1	1	0	0	1
1	1	0	0	0

1	1	0	0	0
1	0	0	0	0
1	1	0	0	0
1	1	0	0	0
1	1	0	0	0
1	1	1	1	0
1	1	0	0	1
1	1	0	1	0
1	1	1	1	0

1	1	1	1	0
1	0	0	0	0
1	0	0	1	0
0	0	0	1	0
1	1	1	1	0
1	0	0	0	0
0	0	0	0	1
1	1	0	0	1
1	1	0	0	0

0	0	0	0	0
0	0	0	1	0
1	1	0	0	1
0	0	0	0	0
1	1	0	1	0
1	1	0	0	1
1	1	1	0	1
1	1	0	0	0
1	1	1	0	1

1	0	1	0	0
0	0	0	1	0
1	1	0	0	1
1	0	0	1	0
0	0	0	1	0
1	1	0	1	0
0	1	0	1	0
1	1	1	1	0
1	1	0	1	0

1	1	0	1	0
1	1	1	0	1
1	0	0	1	0
1	0	0	0	0
1	1	0	0	1
1	1	0	0	0
1	0	1	0	0
1	1	0	0	1
1	0	1	1	0

1	1	0	0	0
1	0	0	0	1
1	0	0	0	0
1	1	0	0	1
1	1	0	0	1
1	0	0	1	0
1	1	0	0	0
1	1	1	1	0
0	0	0	1	0

1	1	1	1	0
0	0	0	0	0
1	0	0	1	0
1	1	1	1	0
1	1	0	0	0
1	0	0	0	0
0	1	0	0	0
1	1	0	1	0
1	1	0	0	0

1	0	0	0	0
1	0	0	0	0
1	1	0	1	0
1	1	0	0	0
0	1	0	0	0
1	1	0	0	0
1	1	0	1	0
1	1	1	0	1
1	0	0	0	1

0	0	0	0	0
1	0	1	0	0
1	1	0	0	0
1	0	0	1	0
0	0	0	1	0
1	0	0	0	0
1	1	0	1	0
1	0	0	0	0
1	1	0	0	0

1	0	0	0	0
1	1	0	1	0
0	0	0	0	0
0	0	0	0	0
1	1	0	1	0
1	1	0	0	1
0	1	0	0	1
1	1	0	0	1
1	0	0	1	0

1	1	1	1	0
1	0	0	0	0
1	1	0	0	0
1	1	0	0	0
1	1	0	0	0
1	1	0	0	0
1	1	1	1	0
1	1	0	0	0
0	0	0	0	0

1	1	0	0	1
1	1	0	0	0
1	1	0	0	0
1	1	0	0	1
1	1	0	0	0
1	1	0	1	0
1	1	0	1	0
1	0	0	1	0
1	0	0	1	0

1	1	0	0	0
1	1	0	0	0
1	1	1	1	0
1	1	0	0	1
1	1	0	1	0
1	1	0	1	0
1	1	0	1	0
1	1	0	0	0
1	1	0	0	1

1	1	0	0	1
1	1	0	0	0
1	0	0	0	0
0	1	0	0	1
1	0	0	0	0
1	0	0	0	0
1	0	0	1	0
1	1	0	0	0
0	1	0	1	0

1	1	0	0	0
0	1	0	1	0
1	0	0	0	1
1	1	0	0	1
1	0	0	0	0
1	1	0	0	1
1	1	0	1	0
1	0	0	0	0
1	1	0	1	0

1	0	0	1	0
1	1	0	1	0
1	1	0	0	0
0	0	0	1	0
1	1	0	1	0
1	1	1	0	1
0	0	0	0	1
1	0	0	1	0
1	1	0	1	0

1	1	1	0	1
1	0	0	1	0
1	0	0	0	1
1	1	0	0	1
1	1	0	1	0
1	1	0	0	1
1	1	0	1	0
1	1	0	0	1
0	1	1	0	1

1	1	0	1	0
1	1	0	1	0
0	0	0	1	0
1	0	0	0	0
1	0	0	0	0
1	0	0	0	0
1	0	0	0	1
1	1	0	0	0
1	1	0	0	0

0	0	0	1	0
0	0	0	0	0
1	1	0	0	0
1	0	0	0	0
0	0	0	0	0
0	0	0	0	0
0	0	0	0	0
1	1	0	0	0
1	1	0	1	0

1	1	0	0	0
1	0	0	0	0
1	1	0	0	0
1	1	0	0	0
0	0	0	1	0
0	1	0	0	0
1	0	0	1	0
1	0	0	0	0
1	1	0	1	0

1	0	0	0	0
1	1	0	0	0
1	1	0	0	0
1	1	0	1	0
1	1	1	0	1
1	1	0	0	0
0	1	0	0	0
0	0	0	0	0
1	1	0	0	1

1	0	0	0	0
1	1	0	0	0
1	0	0	1	0
1	1	0	1	0
1	1	1	0	1
1	1	1	1	0
0	0	0	0	0
1	0	0	0	0
1	1	1	1	0

1	1	0	0	0
0	1	0	0	1
1	1	0	1	0
1	1	0	0	0
1	1	0	0	0
1	0	1	1	0
1	1	1	0	1
1	1	1	1	0
1	1	0	0	1

1	1	0	0	1
0	1	0	0	0
1	0	0	0	1
1	1	0	0	1
1	1	0	0	1
0	0	0	0	0
1	1	0	1	0
1	1	0	1	0
1	1	0	0	0

1	1	0	1	0
1	1	0	0	0
0	1	0	0	0
1	0	1	0	0
1	0	0	1	0
0	0	0	1	0
1	0	0	0	0
1	0	0	0	0
1	0	0	0	0

1	0	0	0	0
1	0	0	0	0
0	0	0	0	0
1	1	0	0	1
1	1	0	1	0
0	0	0	0	0
0	0	0	0	0
1	1	0	0	0
1	1	0	1	0

1	1	1	1	0
1	1	0	1	0
0	0	0	0	0
1	0	0	1	0
1	1	0	0	0
1	0	0	0	0
1	1	0	0	0
0	0	0	1	0
0	0	0	0	0

0	0	0	0	0
1	1	0	1	0
1	1	0	0	0
0	0	0	0	1
1	1	0	1	0
1	1	1	1	0
1	0	1	1	0
1	1	0	1	0
1	1	0	0	0

0	0	0	0	0

TRAST_PANICO	RIESGOSUICIDIO_AHORA	RIESGOSUICIDIO_ALGUNAVEZ	HIPOMANIACO	FOBIA_SOCIAL
0	0	0	0	0
1	0	1	0	0
0	0	0	1	0
0	0	0	0	0
1	0	0	0	0
0	0	0	1	0
1	0	0	0	0
1	0	0	0	0

0	0	0	0	1
0	0	1	0	0
0	0	0	0	0
0	0	0	0	0
0	0	0	0	0
0	0	0	0	0
0	0	0	0	1
0	0	1	0	0
0	0	0	0	0

1	0	0	1	0
0	0	0	0	0
0	0	0	0	0
1	0	1	1	0
1	0	0	0	0
0	0	0	0	0
0	0	0	0	0
0	0	0	0	0
0	0	0	0	0

1	0	1	0	1
1	0	1	0	0
0	0	0	0	0
0	0	0	0	0
0	0	1	0	0
0	0	0	0	0
0	0	0	0	0
0	0	0	0	0
1	0	1	0	0

1	0	0	0	0
0	0	0	0	0
0	0	1	0	0
0	0	0	0	0
0	0	0	0	0
0	0	0	0	0
1	0	0	0	0
0	0	0	0	1
0	0	0	0	0

0	0	0	0	0
0	0	0	0	0
0	0	0	0	0
1	0	1	0	0
0	0	1	0	0
0	0	0	0	0
1	0	1	0	0
1	0	1	0	0
1	0	1	0	0

1	0	1	0	0
1	0	0	0	0
0	0	0	0	0
1	0	0	0	0
0	0	0	0	0
0	0	0	0	0
0	0	0	1	1
0	0	0	0	1
1	0	1	0	0

0	0	0	0	0
0	0	1	0	0
0	0	1	0	0
0	0	0	0	1
0	0	0	0	0
1	0	1	0	1
0	0	0	0	0
1	1	1	0	0
0	0	0	0	0

0	0	1	0	0
0	0	1	0	0
0	1	1	0	0
0	0	1	0	0
0	0	1	0	0
1	1	1	0	1
0	0	0	0	0
0	0	0	0	0
0	0	0	0	0

0	0	0	0	0
0	0	1	0	0
0	0	0	0	0
0	0	0	0	0
0	0	1	0	0
1	0	1	0	0
0	0	0	0	0
0	1	1	1	0
0	0	1	0	0

0	0	1	0	0
1	0	1	0	0
0	0	0	0	0
0	0	0	0	0
1	0	1	0	0
0	0	0	0	0
0	0	0	0	0
0	0	0	0	1
1	0	1	0	0

1	0	0	0	0
0	0	1	1	0
0	0	0	0	0
1	0	0	0	1
0	0	1	1	0
1	0	1	0	1
0	0	0	0	0
0	0	1	0	0
1	1	1	1	1

0	0	0	0	0
1	0	0	0	0
1	0	0	0	0
1	0	1	0	1
0	0	0	0	0
0	1	1	0	0
0	0	0	0	0
0	0	0	0	0
1	0	1	0	0

0	0	0	0	0
0	0	0	0	0
0	0	1	0	0
1	0	0	0	1
0	0	0	0	0
1	0	0	0	0
0	0	0	0	0
0	0	1	0	0
1	0	0	0	0

0	0	0	0	0
0	0	0	0	1
1	0	1	0	0
0	0	1	0	0
1	0	0	0	0
1	0	1	0	1
0	0	0	0	0
1	0	0	0	0
0	0	0	0	0

0	0	1	0	0
0	0	1	0	0
0	0	0	0	0
0	0	0	1	0
0	0	1	0	0
0	0	0	0	1
0	0	0	0	0
1	0	0	0	0
0	0	0	0	0

0	0	0	0	1
0	0	0	0	0
1	0	0	0	0
1	0	0	0	1
0	0	0	0	1
0	0	0	0	1
0	0	0	0	0
1	0	1	0	1
0	0	0	0	0

0	0	0	0	0
0	0	0	0	0
0	0	0	0	0
1	0	0	0	0
0	0	0	0	0
1	0	0	0	0
0	0	1	1	0
0	0	0	0	0
0	1	1	0	1

1	0	0	0	1
0	0	0	0	0
0	0	0	0	0
1	0	0	0	0
0	0	0	0	0
0	0	0	0	0
1	0	0	0	0
0	0	1	0	0
1	0	0	0	0

0	0	0	0	0
0	0	0	0	1
1	0	1	0	1
0	0	1	0	0
0	0	1	0	0
1	0	1	0	0
0	0	0	0	0
1	0	0	0	0
1	0	0	0	0

0	1	1	0	0
1	0	0	0	0
0	0	0	0	0
1	0	1	0	0
1	0	0	0	0
0	0	1	0	0
0	0	0	0	0
0	0	1	0	1
0	0	1	0	0

1	0	0	0	0
0	0	0	0	0
1	0	0	0	1
0	0	1	0	0
0	0	0	0	0
0	0	0	0	0
0	0	0	0	0
0	0	0	0	0
1	0	1	1	0

1	1	1	0	0
0	0	1	1	0
1	0	0	0	0
1	0	0	0	0
0	0	0	0	0
0	0	0	0	0
0	0	0	0	0
1	0	0	0	0
0	0	0	1	0

0	0	0	0	0
0	0	0	0	1
0	1	1	0	1
0	0	0	0	0
1	0	1	0	1
1	0	1	1	0
0	0	0	0	1
0	0	1	0	0
0	0	1	0	1

0	0	0	1	1
0	0	0	0	0
1	0	1	0	1
0	0	0	0	0
0	0	1	0	0
0	0	0	0	0
0	0	0	0	0
1	1	1	0	0
0	1	1	1	0

0	0	0	0	0
0	1	1	0	0
0	0	1	0	0
0	1	1	0	0
0	0	0	0	0
0	0	1	0	0
0	0	0	1	0
0	0	1	0	1
0	0	1	0	0

0	0	0	0	0
0	0	0	0	1
0	0	0	0	0
0	0	0	0	0
1	0	1	1	1
0	0	0	0	1
0	0	0	0	0
0	0	0	0	1
0	0	0	0	0

0	0	0	0	0
0	0	0	0	0
0	0	1	0	0
1	0	0	0	0
0	0	0	0	0
0	0	0	0	0
0	0	0	0	0
1	0	1	0	1
0	0	0	0	0

0	0	0	0	0
1	0	0	0	0
1	0	0	0	0
1	0	0	0	0
0	0	0	0	0
0	0	0	0	0
0	0	1	0	1
0	0	0	0	0
0	0	0	0	0

0	0	0	0	0
0	0	1	0	0
0	1	1	0	0
1	0	0	0	0
0	0	0	0	0
0	0	1	0	0
1	0	1	0	0
0	0	1	0	0
0	0	1	0	0

0	0	0	0	0
0	0	1	0	0
0	0	0	0	0
0	0	0	0	0
1	0	0	0	0
0	0	1	0	0
0	0	1	0	0
1	0	1	0	0
0	0	0	0	0

0	0	0	0	0
1	0	0	0	1
0	0	0	0	0
0	0	0	0	0
1	0	0	0	0
1	0	0	0	0
0	0	0	0	0
0	0	0	0	0
0	0	0	0	0

1	0	0	0	0
1	0	0	0	0
1	0	1	0	0
0	0	0	0	1
0	0	0	0	0
0	0	0	0	0
0	1	1	0	1
0	0	0	0	1
1	0	0	0	0

0	0	1	0	0
0	0	0	1	1
0	0	0	1	0
0	0	0	1	1
1	0	0	0	1
0	0	0	1	0
1	0	1	0	0
1	0	1	0	0
1	0	0	1	0

1	0	1	0	1
1	0	0	0	1
0	0	0	0	0
0	0	0	0	0
1	0	0	1	0
0	1	0	0	0
0	0	0	0	0
0	0	0	0	0
1	0	1	0	0

0	0	0	0	1
0	0	1	0	1
0	0	1	0	0
1	0	0	0	0
0	0	0	0	0
1	1	1	0	0
1	0	0	0	1
0	0	1	0	0
1	0	1	0	0

0	0	0	0	0
0	0	0	0	0
0	0	0	0	0
0	0	0	0	0
0	0	1	1	0
1	0	0	0	0
0	0	0	0	0
0	0	0	0	0
0	0	1	1	0

1	0	0	0	0
0	0	1	0	0
1	0	0	0	0
1	0	0	0	1
1	0	1	1	0
0	0	1	0	1
0	0	0	0	1
0	0	1	0	0
0	0	0	0	1

0	0	0	0	0
0	0	0	0	0
0	0	0	0	0
0	0	0	0	0
0	0	0	0	0
1	0	0	0	0
0	0	0	0	0
0	0	0	0	0
0	0	1	0	1

1	0	0	0	0
0	0	0	0	0
0	1	1	0	0
0	1	1	0	0
0	0	0	0	0
0	0	0	0	0
0	0	0	0	0
1	1	1	0	1
0	0	0	0	0

1	0	1	0	0
0	0	0	0	0
0	0	0	0	0
0	0	0	0	0
0	0	0	0	0
1	0	0	0	0
0	0	0	0	0
0	0	0	0	1
0	0	1	0	0

0	0	0	0	0
0	0	0	0	0
1	0	1	0	0
1	0	0	0	0
1	0	0	0	1
0	1	1	0	1
0	0	0	0	0
0	0	0	0	0
0	0	0	0	0

0	0	0	0	0
0	0	0	0	0
0	0	0	0	0
0	0	0	0	0
0	0	0	0	0
0	1	1	1	1
0	0	0	0	0
0	0	0	0	0
0	0	0	1	0

0	0	1	0	0
1	0	0	0	0
1	0	0	0	1
1	1	0	0	1
1	0	1	1	1
1	0	0	0	0
0	0	1	0	1
1	0	0	0	1
0	0	0	0	0

0	0	0	0	0
0	0	0	0	0
0	0	1	0	0
1	0	0	0	0
0	1	0	0	0
0	0	0	0	1
0	0	0	0	0
0	0	0	0	0
1	0	0	0	0

1	0	0	0	0
0	0	0	1	0
0	0	0	0	0
0	0	0	0	1
0	0	0	0	1
0	0	0	0	0
0	0	0	0	0
1	0	0	0	0
0	0	0	0	0

0	0	0	0	0
0	0	0	0	0
0	0	0	0	0
0	0	0	0	0
0	0	0	0	0
0	0	0	0	0
0	1	1	0	0
0	0	1	0	0
1	0	0	0	1

0	0	0	0	0
0	0	1	0	1
0	0	0	0	0
0	0	0	0	0
1	0	0	0	0
1	0	0	0	0
0	0	1	0	0
0	0	0	0	0
0	0	0	0	0

0	0	0	0	0
0	0	1	0	0
0	0	0	0	0
1	0	1	0	0
1	0	0	0	0
0	1	1	0	0
0	0	0	0	0
1	0	1	0	0
0	0	0	0	0

0	0	0	0	0

ANSIEDAD	TRAS_ANSIEDAD	AGORAFOBIA	TOC	DEPENDENCIA_OH
0	0	0	0	0
1	0	0	0	0
0	0	0	0	0
0	0	0	0	0
0	0	0	1	0
0	0	0	0	0
0	1	0	0	0
0	0	0	0	0

1	0	0	0	0
0	1	0	0	0
0	0	0	0	0
0	1	1	0	0
0	0	0	0	0
0	0	0	0	0
0	0	0	0	0
0	1	0	0	0
0	0	0	0	0

1	0	1	0	0
1	0	0	0	0
1	0	1	0	0
0	0	1	0	1
0	1	0	0	0
0	1	0	0	0
0	0	0	0	0
0	0	0	0	0
0	0	0	0	0

0	0	1	1	0
1	0	0	0	0
0	0	0	0	0
1	0	0	0	0
0	0	0	0	0
0	1	0	0	0
0	0	0	0	0
0	0	0	0	0
0	1	0	0	0

0	1	0	1	0
0	1	0	0	0
0	0	0	0	0
0	0	0	0	0
0	0	0	0	0
0	0	0	0	1
0	1	1	0	0
0	0	0	0	0
0	0	0	0	0

0	0	0	0	0
0	0	0	1	0
1	0	0	0	0
0	1	1	0	0
0	0	0	0	0
1	0	0	0	0
0	0	0	0	0
0	1	1	0	0
1	0	0	0	0

1	0	1	0	0
0	0	0	0	0
0	1	0	0	0
0	1	0	0	0
0	0	0	0	0
1	0	0	0	0
0	0	1	0	0
0	0	0	0	0
0	0	1	0	0

0	1	0	0	0
0	0	0	0	0
0	0	0	0	0
0	1	0	1	0
0	0	0	0	0
0	1	0	0	0
0	0	0	0	0
1	0	0	0	0
0	0	0	0	0

0	0	0	0	0
0	0	0	0	0
0	0	0	0	0
0	1	0	0	0
0	1	0	0	0
0	1	1	0	0
1	0	0	0	0
0	1	0	0	0
0	1	1	0	0

0	0	0	0	0
0	1	0	0	0
0	0	0	0	1
0	0	0	0	0
1	0	0	0	0
1	0	0	0	0
0	0	0	0	0
0	0	1	0	0
1	0	0	0	0

0	0	0	0	0
0	0	1	0	0
0	0	0	0	0
0	0	0	0	0
0	0	0	0	0
0	0	1	0	0
0	0	0	0	0
0	1	0	0	0
0	0	1	0	0

0	0	0	0	0
0	1	0	0	0
0	0	0	0	0
0	0	0	0	0
0	0	0	0	0
1	0	0	0	0
0	0	0	0	0
0	0	1	1	0
1	0	1	0	1

0	0	0	0	0
0	0	0	0	0
0	1	1	0	0
0	1	1	1	1
0	0	0	0	1
0	0	1	0	0
0	0	0	0	0
0	1	0	0	0
1	0	0	0	1

1	0	0	1	0
0	0	0	0	0
0	1	1	0	0
0	0	0	0	0
0	0	0	0	0
0	0	0	0	0
1	0	0	1	0
0	1	1	0	0
1	0	1	0	0

0	0	0	0	0
1	0	1	1	0
0	0	1	0	0
1	0	0	0	0
0	0	0	0	0
1	0	1	0	0
0	1	0	0	0
0	0	0	0	0
0	0	1	0	0

0	1	1	0	0
0	0	0	0	0
0	0	0	0	0
0	0	0	0	0
0	0	0	0	0
0	1	0	0	0
0	0	0	0	0
0	1	0	0	0
1	0	1	0	0

1	0	0	0	0
0	0	0	0	0
0	0	0	0	0
0	0	0	0	0
0	0	0	0	0
0	0	0	0	0
0	0	0	0	0
0	0	0	0	0
1	0	0	0	0

0	1	0	0	0
1	0	0	0	0
0	1	0	0	0
0	1	0	0	0
0	0	0	0	0
0	1	1	0	0
0	0	0	0	0
0	0	0	0	0
0	0	1	0	0

0	0	1	0	0
0	0	0	0	0
0	0	0	0	0
0	0	1	1	1
0	0	1	0	0
0	0	0	0	0
0	0	0	0	0
0	0	0	0	0
0	0	0	0	0

1	0	0	0	0
0	0	0	0	1
0	1	0	0	0
0	1	0	0	0
0	1	0	0	0
0	0	0	0	1
0	0	0	0	0
1	0	0	0	0
0	1	1	0	0

0	0	0	0	1
1	0	0	0	0
0	0	0	0	0
1	0	1	0	0
0	0	0	0	0
0	0	0	0	0
0	0	0	0	0
1	0	0	0	0
0	0	1	0	0

0	1	0	0	0
0	1	0	0	0
0	1	0	0	0
1	0	0	0	0
0	1	0	0	0
1	0	0	0	0
1	0	0	0	0
1	0	0	0	0
1	0	0	1	0

0	1	1	0	0
0	0	1	1	0
0	0	1	0	0
0	0	0	0	0
0	0	0	0	0
0	0	0	0	0
0	0	0	0	0
0	0	0	0	0
0	0	0	0	1

0	1	0	0	0
0	0	0	0	0
1	0	0	0	0
0	0	0	0	0
1	0	1	0	1
0	0	0	1	0
0	0	0	0	0
0	1	0	0	0
0	0	1	0	0

0	0	0	0	0
0	0	0	0	0
1	0	1	1	1
1	0	0	0	1
0	0	0	0	0
0	0	0	0	0
1	0	0	0	0
0	1	0	0	0
0	0	1	1	0

0	0	0	0	0
0	1	0	0	0
0	1	1	0	0
1	0	0	0	0
0	0	0	0	0
0	0	0	0	0
0	1	1	0	0
0	1	1	0	1
0	0	0	0	0

0	1	0	0	0
0	0	0	0	1
0	1	0	0	0
0	1	0	0	0
0	1	1	1	0
0	1	0	0	0
0	1	0	0	0
0	0	0	0	0
0	0	0	0	0

0	0	0	0	0
0	0	0	0	0
0	1	0	0	0
0	1	1	0	0
0	0	0	0	0
0	1	1	0	0
0	0	0	0	0
0	1	0	0	0
0	0	0	0	0

0	0	0	0	1
0	1	1	0	0
0	0	1	0	0
0	0	1	0	0
0	1	1	0	0
0	1	0	1	0
0	0	0	0	0
0	0	0	0	0
0	0	0	0	0

0	0	0	0	0
0	0	0	0	0
0	0	0	0	0
0	0	1	0	0
0	0	0	0	0
0	1	0	0	0
0	0	0	0	0
0	0	0	0	0
1	0	0	0	0

0	0	0	0	0
0	0	0	0	0
0	1	0	0	0
0	0	0	0	0
1	0	0	0	0
1	0	0	0	0
0	0	0	0	0
1	0	1	0	0
0	0	0	0	0

0	1	0	0	0
0	0	0	0	0
1	0	0	0	0
0	0	0	0	0
1	0	0	0	0
0	0	0	0	0
1	0	0	0	0
0	0	0	0	0
0	0	0	0	0

1	0	0	0	0
0	0	1	0	1
1	0	1	0	0
1	0	1	0	0
0	0	0	0	1
0	0	0	0	1
0	1	1	0	0
0	0	0	0	0
0	0	0	0	0

0	0	0	0	0
0	0	1	1	1
1	0	0	0	0
0	1	1	1	0
1	0	1	0	0
0	0	0	0	0
0	0	1	0	0
1	0	0	0	0
1	0	1	0	0

0	0	0	0	0
0	0	1	0	0
0	0	0	0	0
0	1	0	0	0
0	0	1	0	0
0	0	0	0	0
0	1	0	0	0
0	1	0	0	0
0	0	0	0	0

0	1	0	0	0
0	1	0	0	0
0	0	1	0	0
0	1	0	0	0
1	0	0	0	0
1	0	1	0	0
0	1	0	0	0
0	0	0	0	0
0	0	0	0	0

0	0	0	0	0
0	1	0	0	0
1	0	0	0	0
0	0	0	0	1
0	1	0	0	0
0	0	0	1	0
0	1	0	0	0
0	0	0	0	0
0	0	0	0	0

0	1	0	0	0
1	0	0	0	0
0	1	1	0	0
1	0	1	0	0
0	1	1	0	0
0	1	1	0	0
0	0	1	0	0
1	0	0	0	0
1	0	0	0	0

0	1	0	0	0
1	0	0	0	0
0	0	0	0	0
0	0	0	0	0
0	0	0	0	0
0	0	1	0	0
0	0	0	0	0
0	1	0	0	0
0	0	1	0	0

0	0	1	0	0
0	0	0	0	0
0	1	0	0	0
0	0	0	0	0
0	0	0	0	0
0	0	0	0	0
0	0	0	0	0
0	0	1	0	0
0	0	0	0	0

0	1	0	0	1
0	0	0	0	0
0	1	0	0	0
0	1	0	0	0
0	1	0	0	0
0	0	0	0	0
0	0	1	0	0
0	0	1	0	0
0	0	0	0	0

0	0	0	0	1
0	0	1	0	0
0	0	1	1	0
0	0	1	0	0
0	1	0	0	0
0	0	0	0	0
0	0	0	0	0
0	0	0	0	0
0	0	0	0	0

0	0	0	0	0
0	0	0	0	0
0	0	0	0	0
0	0	0	0	0
0	1	0	0	0
0	0	1	0	0
0	0	0	0	0
0	0	1	0	0
0	0	0	0	0

0	0	0	0	0
0	1	0	0	0
0	0	1	0	0
0	0	0	0	1
0	1	1	0	0
0	0	0	0	0
1	0	0	0	0
1	0	1	1	0
0	0	0	0	0

0	0	0	0	0
0	0	0	0	0
0	1	0	0	0
0	0	0	0	0
1	0	0	0	0
0	0	0	0	0
1	0	0	0	0
1	0	0	0	0
0	0	1	0	0

0	1	1	0	0
0	0	0	0	0
0	0	1	0	0
0	0	0	0	0
0	1	0	0	0
0	0	0	0	0
0	1	0	0	0
0	0	1	1	0
0	1	0	0	0

0	0	0	0	0
0	0	0	0	0
0	0	0	0	0
1	0	0	0	0
0	1	0	0	0
0	0	0	0	0
0	1	0	0	0
0	1	0	0	0
1	0	1	0	0

0	1	0	0	0
0	1	0	1	0
0	0	0	0	0
0	0	0	0	0
0	0	0	0	0
0	0	0	0	0
0	1	0	0	0
0	0	0	0	0
0	0	0	0	0

0	0	0	0	0
1	0	0	0	0
0	0	0	0	0
1	0	0	0	0
0	0	1	0	1
0	0	0	0	0
0	0	0	0	0
1	0	1	0	0
1	0	0	0	0

0	0	0	0	0

ANOREXIA	BULIMIA	T_ANTISOCIAL	TEPT	actividad_remunerada
0	0	0	0	0
0	0	0	0	1
0	0	0	0	1
0	0	0	0	0
0	0	0	1	0
0	0	0	0	1
0	0	0	0	1
0	0	0	0	1

0	0	0	0	1
0	0	0	0	1
0	0	0	0	0
0	0	0	0	1
0	0	0	0	1
0	0	0	0	0
0	0	0	1	1
0	0	0	0	1
0	0	0	0	0

0	1	0	0	0
0	0	0	0	0
0	0	0	0	1
0	0	0	0	1
0	0	0	0	0
0	0	0	1	1
0	0	0	0	0
0	0	0	0	1
0	0	0	0	0

0	1	0	0	1
0	1	0	0	0
0	0	0	0	0
0	0	0	1	0
0	0	0	0	0
0	0	0	0	1
0	0	0	0	1
0	0	1	0	0
0	0	1	0	0

0	0	0	0	1
0	0	0	0	0
0	0	0	0	0
0	0	0	0	0
0	0	0	0	1
0	0	0	0	1
0	0	0	0	0
0	0	0	0	1
0	0	0	0	0

0	0	0	0	1
0	0	0	0	1
0	0	0	1	0
0	0	0	0	0
0	0	0	0	0
0	0	0	0	1
0	0	1	0	0
0	0	0	1	1
0	0	0	0	0

0	0	0	0	0
0	0	0	0	0
0	1	0	0	0
0	0	0	0	1
0	0	0	0	0
0	0	0	0	0
0	0	0	0	0
0	0	1	0	0
0	0	0	0	1

0	0	0	0	0
0	0	1	0	1
0	0	0	0	0
0	0	0	0	0
0	0	0	0	0
0	0	0	0	1
0	0	0	0	0
0	0	0	1	1
0	0	0	0	0

0	0	0	0	0
0	0	0	0	1
0	1	0	0	1
0	0	0	0	0
0	0	0	0	0
0	0	1	0	0
0	0	0	0	1
0	0	0	0	0
0	0	0	0	1

0	0	0	0	1
0	0	0	0	1
0	1	1	0	1
0	0	0	0	0
0	0	0	0	1
0	0	0	0	0
0	0	0	0	0
0	0	0	0	0
0	0	0	0	0

0	0	0	0	0
0	0	0	0	1
0	0	0	0	1
0	0	0	0	1
0	1	1	1	1
0	0	0	0	1
0	0	1	0	1
0	0	0	0	0
0	0	0	1	0

0	0	0	0	0
0	0	0	0	1
0	0	0	0	0
0	0	0	0	0
0	0	0	0	0
0	0	0	0	1
0	0	0	0	1
0	0	0	0	1
0	0	1	1	0

0	0	0	0	1
0	0	0	0	1
0	0	0	0	0
0	0	0	0	0
0	0	0	0	1
0	1	0	0	0
0	0	0	0	0
0	0	0	1	1
0	0	0	1	0

0	1	0	0	1
0	0	0	1	1
0	0	0	1	0
0	0	0	0	1
0	0	0	0	1
0	0	0	0	0
0	0	0	0	1
0	0	0	1	1
0	0	0	0	1

0	0	0	0	1
0	0	0	0	0
0	0	0	0	1
0	1	0	1	1
0	0	0	1	1
0	0	0	1	1
0	0	0	0	1
0	0	0	0	0
0	0	0	0	0

0	0	0	0	0
0	0	0	0	0
0	0	0	0	0
0	0	0	0	1
0	0	0	1	1
0	0	0	0	1
0	0	0	1	1
0	0	0	0	1
0	0	0	0	0

0	0	0	0	1
0	0	0	0	0
0	0	0	1	0
0	0	0	0	1
0	1	0	0	1
0	0	0	0	0
0	0	0	0	0
0	0	0	1	0
0	0	0	1	0

0	0	0	0	0
0	0	0	0	0
0	0	0	0	0
0	0	0	0	0
0	0	0	0	0
0	0	0	0	1
0	0	0	0	0
0	0	0	0	1
0	1	0	1	0

0	0	0	0	0
0	0	0	0	1
0	0	0	0	1
0	1	0	0	1
0	0	0	0	0
0	1	0	0	1
0	0	0	0	0
0	0	0	0	1
0	0	0	0	0

0	0	0	0	1
0	0	0	0	1
0	0	0	0	1
0	0	0	0	1
0	0	0	0	0
0	0	0	0	1
0	0	0	0	1
0	0	0	0	0
0	0	0	0	0

0	0	1	0	1
0	0	0	0	0
0	0	0	0	0
0	0	0	0	0
0	0	0	0	0
0	0	0	0	1
0	0	0	0	0
0	0	0	1	0
0	0	0	1	0

0	0	0	0	1
0	0	0	0	0
0	0	0	0	1
0	0	0	1	0
0	0	0	0	1
0	0	0	0	1
0	0	0	1	1
0	0	0	1	1
0	0	0	1	0

0	0	0	1	0
0	0	0	0	1
0	0	1	0	1
0	1	0	0	0
0	0	0	0	0
0	0	0	0	0
0	0	0	0	0
0	0	0	0	0
0	0	1	0	1

0	0	0	0	0
0	0	0	0	0
0	0	0	0	1
0	0	0	0	1
0	1	1	0	1
0	0	0	0	1
0	0	0	0	0
0	0	0	0	1
0	1	1	1	1

0	0	0	0	1
0	0	0	0	0
0	1	1	1	0
0	0	0	0	1
0	0	0	0	0
0	0	0	0	0
0	0	0	0	1
0	0	0	0	0
0	0	0	0	0

0	0	0	0	0
0	0	0	0	0
0	0	0	0	1
0	0	0	0	0
0	1	0	0	0
0	0	0	0	0
0	0	0	0	1
0	0	1	0	1
0	0	0	0	0

0	0	0	0	1
0	0	0	0	1
0	0	0	0	1
0	0	0	0	0
0	0	0	0	1
0	0	0	0	0
0	0	0	0	0
0	0	0	0	0
0	0	0	0	1

0	0	0	0	0
0	0	0	0	1
0	0	0	0	0
0	0	0	0	0
0	0	0	0	0
0	0	0	0	1
0	0	0	0	1
0	0	1	0	1
0	0	0	0	0

0	1	0	0	0
0	0	0	0	0
0	0	0	0	1
0	0	0	1	1
0	0	0	0	1
0	0	0	0	0
0	0	0	0	0
0	0	0	0	0
0	0	0	0	1

0	0	0	0	0
0	0	0	0	1
0	0	0	0	1
0	0	0	1	1
0	0	0	0	0
0	0	0	0	0
0	0	0	0	0
0	0	0	0	0
0	0	0	0	1

0	0	0	0	0
0	0	0	0	0
0	0	0	0	0
0	0	0	0	0
0	0	0	0	0
0	0	0	0	1
0	0	0	0	1
0	0	0	1	1
0	0	0	1	0

0	0	0	1	0
0	0	0	0	1
0	0	0	0	0
0	0	0	0	0
0	0	0	0	1
0	0	0	1	0
0	0	0	1	0
0	0	0	0	0
0	0	0	0	1

0	0	0	1	1
0	1	0	1	0
0	1	0	0	1
0	1	0	0	1
0	0	0	0	0
0	0	0	0	1
0	0	1	1	1
0	0	0	0	0
0	0	0	0	0

0	0	0	0	1
0	0	0	0	0
0	0	0	1	1
0	0	0	0	0
0	0	0	0	1
0	1	0	0	1
0	0	0	0	0
0	1	1	0	0
0	0	0	0	0

0	1	0	0	1
0	0	0	0	0
0	0	0	0	1
0	0	0	0	0
0	0	0	0	1
0	0	0	0	1
0	0	0	0	0
0	0	0	0	0
0	0	0	0	0

0	0	0	0	1
0	0	0	0	1
0	0	0	0	0
0	0	0	0	1
0	0	0	0	0
0	1	0	0	1
0	0	0	0	1
0	0	0	0	1
0	0	0	0	0

0	1	0	0	1
0	0	0	0	1
0	0	0	0	1
0	0	0	0	1
0	0	1	0	1
0	0	0	0	0
0	0	0	1	0
0	0	0	0	1
0	0	0	0	1

0	0	0	0	1
0	0	0	0	1
0	0	0	1	1
0	0	0	1	0
0	0	0	1	1
0	0	0	1	0
0	0	0	0	0
0	0	1	1	0
0	0	0	0	1

0	0	0	0	1
0	0	0	0	1
0	0	0	0	0
0	0	0	0	0
0	0	0	0	0
0	0	0	0	0
0	0	0	0	0
0	0	0	0	0
0	0	0	0	1

0	0	0	0	0
0	0	0	0	1
0	0	0	0	0
0	0	0	1	1
0	0	0	0	0
0	0	0	0	0
0	0	0	0	1
0	1	0	1	1
0	1	0	0	1

0	0	0	0	1
0	0	0	0	0
0	0	0	0	1
0	0	0	0	1
0	0	0	0	0
0	0	0	0	0
0	0	0	0	0
0	0	0	0	0
0	1	0	0	0

0	0	1	0	1
0	1	0	0	1
0	1	0	0	1
0	0	0	0	0
0	0	0	1	0
0	0	0	0	1
0	0	0	0	0
0	0	0	0	0
0	0	0	0	0

0	0	0	0	1
0	0	0	0	0
0	0	0	0	0
0	0	0	0	0
0	0	0	0	0
0	0	0	1	0
0	0	0	0	0
0	0	0	0	0
0	0	0	0	0

0	0	0	0	0
0	0	0	0	0
0	0	0	0	1
0	0	0	0	1
0	0	0	0	1
0	0	0	0	0
0	0	0	1	0
0	0	0	1	0
0	0	0	0	1

0	0	0	0	0
0	0	1	0	0
0	0	0	0	0
0	0	0	1	0
0	0	0	0	0
0	0	0	0	1
0	0	0	0	0
0	0	0	0	0
0	0	0	1	0

0	0	0	0	0
0	0	0	0	0
0	0	0	0	0
0	0	0	0	1
0	0	0	0	1
0	0	0	0	0
0	0	0	0	1
0	0	0	0	0
0	0	0	0	1

0	0	0	0	1
0	0	0	0	1
0	0	0	0	1
0	0	0	1	1
0	0	0	0	1
0	0	0	0	0
0	0	0	1	1
0	0	0	1	1
0	0	0	0	1

0	0	0	0	0
0	0	0	0	1
0	0	0	0	1
0	0	0	0	0
0	0	0	0	0
0	0	0	0	1
0	0	0	0	1
0	0	0	0	1
0	0	0	0	0

0	0	0	0	0
0	0	0	0	1
0	0	0	0	0
0	0	0	0	0
0	0	0	0	1
0	0	0	0	0
0	0	0	0	1
0	0	0	1	1
0	0	0	0	0

0	0	0	0	1

vivesolo	hombres	n_hijos	fecha_nacimiento	nivel_educacional
1	0	0	54007	1
0	0	2	55775	6
0	0	4	15550	0
1	1	0	24035	0
1	0	0	20867	1
1	1	0	22966	1
1	0	1	18432	4
1	0	1	28217	4

1	0	2	22634	1
1	0	2	28730	5
1	0	3	17011	1
0	0	0	23654	3
0	0	1	20600	1
1	0	1	25360	4
1	0	4	19181	1
1	0	4	20739	1
1	0	3	18146	5

1	0	2	33191	4
1	0	2	23354	1
1	0	2	17722	3
0	0	5	19857	3
1	0	2	23679	4
0	0	2	19626	1
1	0	3	25315	4
1	0	1	28550	4
1	0	1	47359	1

1	0	2	31047	6
1	0	4	26917	1
1	0	2	18930	2
1	0	3	23440	2
0	0	0	16987	2
1	0	4	29325	2
1	0	3	17815	1
1	0	0	22843	2
1	0	2	29189	4

1	1	0	41689	5
1	0	1	18864	2
1	0	3	25134	1
1	0	2	25279	2
0	0	4	24197	1
1	1	3	24057	1
1	0	2	18151	1
1	0	0	35457	3
1	0	11		1

1	0	1	29365	5
1	1	0	36104	3
1	0	2	24458	1
1	0	2	24439	4
1	0	2	35641	3
1	0	1	28067	5
1	0	5	29190	4
1	0	2	30419	4
1	0	0	17624	1

1	0	4	26181	1
0	0	3	19464	1
0	0	2	18593	4
1	0	1	30237	4
1	0	2	29884	5
1	0	1	17145	1
1	0	1	26780	4
0	0	1	14753	1
1	0	1	30937	5

1	0	3	14824	1
1	1	4	15734	1
1	0	7	15698	1
0	0	0	18594	4
0	0	0	15798	1
1	0	1	30452	5
1	0	3	16567	6
1	0	2	22693	2
1	0	2	15976	3

1	0	4	18548	1
1	0	5	22227	2
1	0	0	33872	6
1	0	3	8076	1
1	0	2	19985	2
0	0	3	24191	3
1	0	2	26950	4
0	0	14	15635	0
1	0	2	21711	4

1	0	1	24146	1
1	0	3	22063	1
1	0	1	32322	4
1	0	3	20589	2
1	0	3	26899	6
1	0	1	29761	4
0	0	3	18934	2
0	0	3	25948	4
0	0	3	19464	4

1	0	2	29458	4
1	0	4	23220	6
1	0	3	27370	4
1	0	0	33454	5
1	0	0	32467	5
1	1	2	22114	2
1	1	0	30330	4
1	0	1	17089	1
0	0	2	16863	2

1	0	2	33231	5
1	0	3	21728	3
1	0	0	29802	4
1	0	3	22907	4
1	0	3	22471	2
1	0	5	23629	3
1	0	2	25885	3
1	0	3	25762	1
0	0	4	26561	3

1	0	2	28280	3
1	0	2	25817	6
1	0	1	28983	3
1	0	2	24311	1
0	0	1	22292	4
1	0	3	20875	6
1	0	0	27259	4
1	0	2	23714	2
1	0	1	24954	3

1	0	3	23911	4
1	0	2	18621	3
1	1	1	19849	0
1	0	1	27566	2
1	0	4	17298	3
0	0	3	17467	1
1	1	3	26574	0
1	1	2	15912	1
0	0	1	21557	6

1	0	1	29584	4
1	0	1	25360	6
1	0	4	25215	4
1	0	2	32541	6
1	0	2	25243	1
1	0	2	29364	2
0	0	0	18482	4
1	0	1	32394	4
1	0	3	24606	1

0	0	4	17671	0
1	0	2	32814	2
1	0	1	30452	4
1	0	4	23032	1
1	0	3	25090	2
1	0	2	25177	4
1	1	2	30177	5
1	0	2	22892	4
1	0	2	27282	1

1	0	2	26857	4
1	0	3	24653	2
1	0	3	28546	1
1	0	1	26805	1
1	0	0	36100	1
1	0	4	22699	1
1	0	6	15987	1
1	0	1	32537	4
1	0	2	22040	6

1	0	4	22341	3
1	0	2	29685	4
1	0	2	20576	4
1	0	1	41741	6
1	0	3	17893	1
1	0	1	20099	4
1	0	3	22280	4
1	1	7	14123	1
1	0	0	33563	4

1	0	2	27576	3
1	1	0	35929	3
0	0	0	28383	6
1	0	1	33194	5
1	0	3	25871	6
1	0	1	26912	4
0	0	3	20498	2
1	0	5	21648	6
1	0	1	19919	5

1	0	5	23916	2
1	0	0	35785	3
1	0	2	21856	4
1	0	3	19287	1
1	0	2	17407	1
1	0	1	31776	4
1	0	2	20696	4
0	0	3	30004	4
1	0	4	21629	4

1	0	3	22974	1
1	0	0	34344	3
0	1	4	17591	4
1	0	1	31873	4
1	0	0 #NULL!		2
1	0	1	21442	4
0	0	0	21707	2
1	0	2	35933	2
1	0	2	32296	3

0	0	1	31037	4
1	0	2	23012	1
1	0	4	25972	3
1	0	4	24718	2
1	0	2	24283	4
1	0	2	29811	4
1	1	2	22801	4
1	0	3	22285	2
1	0	1	33337	2

1	0	2	21205	3
1	0	0	23335	2
1	0	3	28340	1
1	0	1	32390	1
1	0	5	18595	3
1	0	1	#NULL!	1
1	0	3	26706	3
1	0	3	27528	4
1	0	6	24735	1

1	0	3	31791	4
1	0	2	28381	1
1	0	1	32569	5
1	1	3	18624	3
1	0	0	33336	5
0	0	2	22757	3
1	0	2	23011	6
1	0	0	30268	5
1	0	2	23230	4

1	0	0	35377	3
0	0	2	16613	6
0	0	0 #NULL!		1
1	1	2	26759	6
1	0	6	41739	1
1	0	3	16788	3
1	0	5	12844	2
1	1	2	28810	6
1	0	3	27329	3

1	0	4	17891	1
1	0	4	22557	3
1	0	2	26287	2
1	1	0	32199	5
1	0	1	31032	5
1	0	4	23064	3
1	1	18	17574	6
1	0	2	27034	1
1	0	8	21760	2

1	0	1	32783	4
1	1	2	27607	3
1	0	2	33054	4
1	0	4	25671	4
1	0	0	33462	4
1	0	4	23117	2
1	0	3	24159	1
1	1	0	31405	6
1	0	2	22150	6

1	0	5	24025	1
1	0	1	30804	4
1	0	2	19843	4
1	0	3	21897	1
1	0	0	34328	4
1	0	2	32773	6
1	1	2	23446	6
1	0	0	33011	5
1	0	2	26154	6

1	1	1	23971	4
1	0	2	24828	4
1	0	1	26042	4
1	1	2	20007	4
1	1	3	22577	5
1	0	3	27628	4
1	0	5	26675	1
1	0	3	21728	2
1	0	1	27215	6

1	0	2	29702	2
1	0	1	29139	4
0	1	3	27182	4
1	0	2	24466	2
0	0	4	17010	2
1	0	1	26292	4
1	0	2	17188	3
1	0	1	22713	1
1	0	2	41782	6

1	0	3	19016	1
1	0	2	33916	1
1	0	1	34735	3
1	1	4	15339	1
1	0	3	24566	1
1	0	4	22994	2
1	0	3	23527	3
1	0	3	24374	1
1	0	3	22889	3

1	0	4	17054	1
1	0	0	36119	3
1	0	2	18446	4
0	0	0	17403	1
1	0	3	27506	4
1	1	4	15824	1
1	0	2	18607	4
1	0	5	18492	1
1	0	1	22298	3

1	0	1	28403	4
1	0	2	17844	3
1	0	1	29724	6
1	0	1	30333	6
1	1	2	25839	5
0	1	2	23553	4
1	0	2	30335	4
1	0	0	27630	5
1	0	2	20814	3

1	0	0	33630	5
1	0	3	26806	6
1	0	2	29080	4
1	0	4	21575	1
1	0	2	30075	4
1	0	1	20582	4
1	0	2	20309	3
1	0	4	25162	1
1	0	3	30650	2

1	0	1	30098	5
1	0	1	34289	4
1	1	3	19154	4
1	0	2	16028	3
1	1	3	20893	6
1	0	2	24230	1
1	1	3	14538	1
1	0	3	16548	2
1	0	2	24722	5

1	0	4	24964	2
0	0	3	19378	1
1	0	3	22396	1
1	0	3	24880	1
0	1	0	18267	4
0	0	0	31992	4
1	0	2	25656	1
0	0	0	27418	3
1	0	5	22843	5

1	0	2	30260	2
1	0	3	20762	6
1	0	2	18618	3
1	1	2	23986	6
1	0	2	30533	4
1	0	3	21259	1
1	0	2	22916	1
1	0	1	33517	4
1	0	2	30014	4

0	0	2	12436	6
1	1	3	19322	1
1	0	3	25468	6
0	0	4	18756	0
1	0	4	22064	2
1	0	4	21655	2
1	0	3	29873	4
1	0	5	20764	1
1	1	0	35096	3

1	0	1	33898	6
1	0	0	29215	6
1	0	5	16559	1
1	0	2	18744	1
1	0	6	21671	1
1	0	6	13856	0
1	0	4	17149	0
1	0	6	16038	1
1	0	3	22188	3

1	0	5	15506	1
1	0	2	19746	3
1	0	3	20772	1
1	0	0	36768	1
0	1	4	21013	6
1	1	1	17691	4
1	1	2	19264	3
1	0	0	34704	4
0	0	0	29439	6

1	0	1	30831	4
1	0	6	12554	1
1	0	2	25643	6
1	0	5	19482	6
1	0	5	19619	1
1	0	2	33347	4
1	0	0	20328	4
1	0	2	26585	2
1	0	1	33584	4

1	1	2	21031	1
1	0	2	29578	5
1	0	1	30066	6
1	0	5	24184	3
1	0	3	22456	3
1	0	4	31162	2
0	0	7	14211	1
1	0	1	28382	1
1	0	7	17521	3

0	1	3	22666	4
1	0	2	33613	2
1	0	2	32813	1
1	0	3	20162	4
0	1	2	19906	2
1	0	3	24770	1
1	0	6	15149	1
1	0	1	23259	1
1	0	2	19168	3

1	0	1	25013	4
1	0	3	20098	4
1	0	1	24339	6
0	1	0	20832	4
1	1	2	20692	1
1	0	2	17948	1
0	1	0	30983	4
1	0	2	29311	4
1	0	2	30589	5

0	0	2	#NULL!	6
1	1	3	16541	3
0	1	1	22523	4
1	0	2	29856	2
1	0	2	29446	4
1	1	0	34153	5
1	0	2	19938	6
0	1	4	22711	3
1	0	3	23179	6

1	0	2	19810	4
1	0	3	28639	2
1	0	1 #NULL!		4
1	0	4	22028	4
1	0	0	34717	3
1	0	2	17089	1
1	1	0	35541	3
1	0	3	27225	1
1	0	2	23473	4

1	0	3	29033	4
1	0	1	33312	4
1	1	3	15935	2
0	0	3	22448	3
1	0	2	24281	3
1	0	3	27284	4
1	0	2	21540	1
1	0	2	29239	6
1	1	0	34157	5

1	0	3	19951	1
1	0	3	27650	6
1	0	2	28700	4
1	0	4	24275	4
1	0	4	14510	1
1	0	2	24541	3
1	1	3	27430	4
0	1	7	20774	4
1	0	3	18172	1

0	0	2	21939	2
1	0	0	24977	4
1	0	1	27954	4
1	0	3	19003	6
0	1	3	24358	4
1	0	3	21465	0
1	0	1	29296	5
1	0	4	26715	4
1	0	4	41840	3

0	0	0	30066	0
1	0	4	41821	0

estado_civil	isdc30total0	maltratoinfantil0	violencia_pareja	Suma_Trastornos
3	42	0	0	2
5	45	4	0	3
5	25	1	0	2
3	39	1	0	0
4	36	5	0	3
4	36	2	0	1
1	29	3	0	2
5	37	0	0	2

0	32	3	0	2
4	31	3	0	3
4	24	4	0	1
1	30	2	0	4
5	21	1	0	1
2	15	0	6	1
5	31	0	0	2
4	26	3	0	3
2	43	2	0	1

1	41	7	0	6
3	47	1	0	2
1	11	1	0	1
4	53	0	0	6
5	32	1	0	3
4	4	2	11	3
3	33	1	4	1
3	15	0	0	0
5	19	2	0	1

1	47	2	3	7
2	36	7	12	5
3	22	0	6	1
4	46	5	12	3
5	39	4	0	2
2	30	6	10	2
3	32	0	0	2
4	28	5	8	3
1	35	2	0	5

1	15	0	0	3
5	26	4	1	3
3	24	5	6	2
2	36	7	1	2
4	20	5	4	1
3	17	3	0	1
5	26	3	9	4
1	14	2	0	1
3	32	2	0	1

1	29	0	8	1
1	25	1	2	1
4	41	3	7	2
4	29	7	12	5
2	24	6	7	1
1	38	1	4	0
1	53	1	0	4
3	34	5	0	6
3	37	3	0	2

4	37	5	0	4
5	38	6	0	1
1	14	1	0	3
2	12	0	0	2
3	42	0	0	0
3	28	3	0	1
1	14	3	1	4
5	15	2	9	2
1	46	1	1	5

3	28	4	10	2
3	14	6	3	2
5	26	3	10	3
1	21	0	0	3
5	25	0	3	1
1	35	2	10	6
3	18	1	2	1
4	30	5	10	5
3	30	0	0	0

3	25	4	11	1
4	23	5	12	1
1	51	4	0	3
3	33	3	8	2
3	35	5	4	3
4	64	6	11	8
3	56	0	0	1
5	35	3	0	1
3	39	1	6	3

2	35	3	0	1
3	38	5	10	4
1	42	4	2	4
3	16	2	0	0
3	33	3	2	2
1	37	4	5	3
5	17	1	5	0
3	41	4	12	5
5	42	5	8	2

3	30	0	3	2
4	49	0	4	4
4	41	2	5	1
1	41	2	2	0
1	46	2	3	6
1	35	3	4	2
2	50	2	4	2
3	41	3	2	3
4	52	0	8	5

1	41	1	7	2
3	46	3	9	5
3	39	0	0	1
3	18	2	0	4
5	52	2	11	4
4	47	6	9	5
4	16	0	7	1
3	21	0	0	4
1	68	6	12	11

1	4	3	10	0
4	12	2	1	1
2	42	1	1	5
2	38	2	11	8
5	22	3	10	1
3	48	4	7	6
3	18	4	5	1
2	41	0	4	3
2	35	6	7	5

3	37	0	0	2
3	27	0	0	2
3	43	1	3	5
1	33	3	0	4
4	30	3	3	1
4	23	4	9	3
4	41	5	3	2
5	21	1	0	5
5	28	0	0	3

2	37	3	8	1
1	48	4	6	5
3	27	3	6	4
1	54	1	7	4
2	26	4	0	3
4	38	5	2	6
1	29	2	0	3
3	47	0	0	1
4	21	2	0	1

5	26	4	10	4
2	40	4	5	3
2	24	2	3	1
2	35	6	11	3
3	38	2	0	3
3	34	5	0	2
2	11	3	4	1
3	13	0	0	3
3	18	3	0	1

3	29	4	4	1
3	31	0	9	0
5	26	5	0	3
3	18	1	6	2
1	15	3	0	2
3	21	6	9	2
3	27	1	3	1
2	30	2	10	5
3	35	0	6	2

3	46	3	9	2
3	34	1	3	0
3	23	2	2	1
3	20	0	4	2
1	40	4	4	1
4	33	0	0	4
3	46	5	0	3
3	26	3	0	0
1	41	5	0	7

4	51	1	8	4
1	35	1	0	0
1	36	1	3	1
1	40	5	10	6
3	40	2	8	2
4	34	0	5	1
5	35	4	7	2
3	34	3	1	2
1	32	1	1	3

3	29	0	0	0
1	28	5	0	2
3	48	6	4	4
3	39	4	0	3
3	37	0	0	3
1	25	2	1	4
3	28	0	1	0
1	45	0	2	3
3	33	6	0	4

4	54	6	10	5
1	30	1	6	2
4	24	4	5	0
3	40	4	4	4
3	20	5	6	2
2	30	4	1	2
5	23	1	0	1
1	60	4	0	5
2	31	1	11	4

1	34	2	4	3
3	35	1	0	1
2	22	3	12	4
4	34	2	12	3
3	34	0	0	2
1	31	3	8	1
3	28	5	0	3
4	39	2	12	2
4	37	2	7	6

3	47	5	5	7
5	26	4	0	4
4	34	4	10	3
2	21	5	3	2
4	29	2	8	1
2	23	3	0	0
3	21	3	0	1
3	43	3	0	3
1	12	2	3	4

3	7	1	0	1
2	30	0	0	1
1	37	0	6	5
5	3	1	0	0
1	45	2	9	8
4	33	2	11	6
4	56	2	0	3
1	33	1	0	3
3	33	5	3	8

1	19	3	8	2
4	6	1	5	0
2	57	0	5	11
4	43	2	4	1
2	33	0	11	1
3	26	3	0	1
5	20	4	11	1
4	43	0	5	5
3	47	3	8	6

3	24	0	0	1
2	33	3	11	5
2	27	0	10	3
1	28	0	0	2
1	32	3	2	3
4	27	4	9	2
2	37	1	0	3
3	50	3	5	8
3	37	1	6	1

4	31	2	6	2
1	27	5	7	3
3	31	4	3	1
3	26	2	1	3
3	44	3	7	9
2	36	4	10	2
3	25	3	4	2
1	30	1	9	2
4	22	0	8	0

2	29	3	9	1
2	19	1	0	0
4	29	4	3	2
3	32	2	8	4
1	21	3	5	1
3	27	0	10	2
3	23	0	0	1
1	25	1	0	6
3	39	4	7	1

3	28	1	1	2
3	48	0	6	3
4	42	3	10	3
3	26	1	1	4
4	18	1	3	3
3	40	3	2	3
4	35	6	9	3
5	32	0	8	2
1	33	0	0	1

2	17	1	7	0
1	58	4	5	1
4	50	4	0	3
3	52	1	1	3
1	28	4	12	0
3	44	0	1	2
1	42	2	3	3
1	53	4	0	1
4	41	3	10	2

3	46	3	1	0
1	46	4	9	2
2	15	0	0	1
5	23	3	0	0
4	35	3	4	2
4	46	4	11	3
1	25	5	10	3
1	44	3	3	6
3	34	4	9	1

3	37	0	6	3
1	26	0	0	2
3	22	2	0	1
4	24	1	7	1
4	26	2	9	2
3	30	4	1	3
4	37	4	8	2
5	33	2	6	1
1	0	3	0	0

1	45	2	5	4
5	44	2	6	6
1	45	3	5	5
3	48	3	1	5
3	33	4	2	2
4	29	1	3	2
3	45	6	4	8
1	28	1	0	1
5	38	1	7	1

1	27	3	3	2
3	51	2	11	6
4	30	5	6	3
2	44	1	12	7
2	25	2	5	4
4	28	2	1	3
3	37	3	2	4
4	48	6	10	5
2	35	2	6	5

1	38	4	7	6
2	22	3	0	4
2	30	2	0	0
3	19	2	3	3
2	27	0	0	3
2	40	4	8	1
3	27	1	0	1
3	23	4	0	2
4	22	1	6	3

2	33	5	1	3
4	35	6	6	4
3	31	4	9	3
3	29	6	4	4
1	24	3	0	0
1	46	1	7	7
4	51	6	10	4
1	17	1	0	1
1	38	6	10	3

1	26	5	3	1
4	23	0	8	2
3	25	0	6	1
4	7	1	0	1
2	31	3	5	5
3	41	2	3	4
3	24	5	12	3
1	48	3	0	0
3	28	1	0	3

5	29	1	3	4
3	40	3	3	1
4	28	1	6	5
0	45	7	1	6
4	45	5	12	7
3	49	6	3	7
3	39	3	0	3
4	48	5	12	5
1	22	2	0	3

2	34	1	0	2
3	32	0	0	1
5	10	0	6	0
2	45	4	0	0
5	48	1	3	0
5	55	1	0	2
3	49	4	9	1
5	41	2	0	2
4	49	0	0	4

3	28	5	0	2
3	15	0	0	0
3	45	5	11	4
1	59	6	0	3
1	13	3	4	0
1	22	1	4	0
3	20	2	0	0
1	48	1	2	8
4	37	1	7	2

1	38	1	0	5
5	16	4	5	0
3	30	3	0	2
3	37	1	3	2
4	29	3	5	1
1	29	2	6	2
1	34	1	0	1
1	36	3	4	2
1	34	2	6	3

4	19	2	3	2
2	40	3	2	3
1	35	2	0	6
4	41	4	5	3
5	47	2	12	6
5	52	4	4	4
5	15	1	0	1
1	4	0	0	0
3	38	3	2	2

4	36	6	4	0
2	35	5	2	1
2	34	2	0	0
3	38	3	0	1
4	45	0	2	3
3	46	6	12	7
4	10	0	3	0
1	31	0	3	1
3	32	5	10	2

4	26	0	9	2
5	13	3	6	4
1	46	0	1	4
1	28	2	0	5
3	48	5	5	7
3	23	0	0	1
1	34	4	11	5
2	54	6	3	6
1	31	1	7	2

5	44	1	0	2
3	21	3	0	2
4	20	3	6	3
3	39	0	3	4
3	23	1	4	3
1	11	4	0	1
4	38	1	0	1
4	39	0	8	1
3	30	5	1	4

3	41	0	0	4
3	22	5	0	2
1	13	0	0	2
5	39	3	7	1
1	35	2	0	2
3	6	1	0	0
1	19	2	0	1
2	15	3	3	3
1	31	1	3	1

3	36	1	2	0
1	35	0	2	0
5	8	1	0	0
4	40	5	10	3
4	24	7	5	2
3	22	1	0	0
3	24	4	9	4
3	27	3	0	4
1	31	3	0	4

5	49	1	12	2
4	41	4	10	5
2	16	0	4	0
4	27	2	7	0
3	50	0	4	2
3	42	1	0	1
3	36	2	0	3
4	13	0	3	0
2	29	0	0	0

1	15	0	7	0
3	37	1	4	2
3	18	2	3	1
3	44	0	7	3
4	41	5	1	4
4	45	5	10	3
2	25	2	0	0
2	41	5	12	5
4	0	2	10	1

0	0	0	6	0
3	0	4	12	0

total_eventos_vitales_ahora	suma_sucesos	total_enfermedades_no_mentales	al_menos_un_maltrato_inf	al_menos_un_vif
2	3	2	0	1
0	3	5	1	1
0	3	2	1	1
0	1	1	1	1
0	1	5	1	1
0	1	0	1	1
0	2	4	1	1
0	4	0	0	1

3	4	1	1	1
5	1	0	1	1
0	3	4	1	1
0	4	0	1	1
0	1	1	1	0
0	2	0	0	1
0	4	5	0	1
8	2	2	1	1
8	3	1	1	1

2	6	0	1	1
0	4	3	1	1
0	2	2	1	1
11	8	1	0	1
5	1	2	1	1
0	3	3	1	1
2	2	2	1	1
0	1	2	0	0
0	4	2	1	0

0	5	1	1	1
9	5	0	1	1
0	2	0	0	1
0	2	3	1	1
0	5	3	1	0
1	3	0	1	1
0	3	1	0	0
0	4	0	1	1
0	2	0	1	0

0	4	0	0	0
0	3	1	1	1
0	3	0	1	1
0	4	1	1	1
0	6	0	1	1
0	0	0	1	0
0	1	0	1	1
0	1	0	1	1
0	3	3	1	0

0	4	1	0	1
0	3	0	1	1
0	4	2	1	1
5	4	0	1	1
10	5	0	1	1
0	2	0	1	1
6	0	0	1	1
9	1	0	1	1
0	1	2	1	1

0	2	5	1	1
0	1	2	1	1
0	0	6	1	1
0	2	0	0	0
0	4	1	0	1
0	5	3	1	1
2	8	0	1	1
0	0	4	1	1
4	3	1	1	1

2	2	5	1	1
6	2	2	1	1
0	4	5	1	1
0	2	0	0	0
0	2	2	0	1
0	2	0	1	1
3	1	2	1	1
0	5	2	1	1
0	1	3	0	0

2	0	1	1	1
0	1	3	1	1
0	0	0	1	0
13	2	1	1	1
4	2	0	1	1
0	0	1	1	1
0	0	1	0	0
0	0	3	1	0
9	1	3	1	1

1	2	3	1	0
3	5	2	1	1
0	4	0	1	1
0	2	2	1	0
0	4	1	1	1
0	4	0	1	1
9	1	1	1	1
0	4	1	1	1
16	3	2	1	1

0	4	0	0	1
0	4	0	0	1
6	1	0	1	1
0	2	1	1	1
1	4	0	1	1
3	1	0	1	1
0	6	0	1	1
0	2	2	1	1
0	3	3	0	1

2	2	0	1	1
2	4	0	1	1
0	2	0	0	0
1	5	1	1	0
0	3	2	1	1
0	6	0	1	1
0	1	1	0	1
0	2	0	0	0
0	8	0	1	1

16	4	0	1	1
0	0	1	1	1
1	2	1	1	1
0	1	3	1	1
0	4	3	1	1
12	4	3	1	1
0	3	0	1	1
0	2	2	0	1
4	4	0	1	1

0	2	0	0	0
0	1	2	0	0
6	1	1	1	1
0	2	0	1	0
6	3	3	1	1
0	0	1	1	1
6	4	2	1	1
0	0	1	1	0
0	1	0	0	0

0	1	0	1	1
13	3	0	1	1
9	1	1	1	1
11	3	0	1	1
0	1	0	1	0
0	4	0	1	1
0	2	2	1	0
0	6	0	0	0
0	5	2	1	0

0	3	2	1	1
5	4	0	1	1
12	2	0	1	1
7	5	2	1	1
0	2	1	1	0
0	5	0	1	0
0	6	0	1	1
0	2	0	0	0
0	0	0	1	0

0	5	0	1	1
0	6	1	0	1
0	2	1	1	0
2	3	1	1	1
0	2	0	1	0
4	3	0	1	1
6	2	2	1	1
0	12	0	1	1
0	2	2	0	1

0	1	0	1	1
3	5	0	1	1
2	1	0	1	1
1	2	0	0	1
0	2	4	1	1
0	3	0	0	0
0	5	1	1	0
0	1	2	1	0
0	6	1	1	0

4	5	0	1	1
0	2	0	1	0
4	4	0	1	1
8	8	0	1	1
10	4	0	1	1
0	2	0	0	1
0	5	2	1	1
1	4	1	1	1
0	5	1	1	1

2	1	1	0	0
0	2	0	1	1
2	2	3	1	1
0	1	2	1	0
0	0	2	0	0
1	2	0	1	1
2	1	0	0	1
3	0	0	0	1
0	1	1	1	0

6	8	0	1	1
0	4	0	1	1
0	3	2	1	1
1	4	1	1	1
10	6	2	1	1
0	2	2	1	1
0	1	1	1	0
0	3	0	1	0
4	3	1	1	1

5	2	0	1	1
1	1	0	1	0
0	4	2	1	1
0	5	1	1	1
0	2	3	0	0
0	2	0	1	1
0	1	0	1	0
0	3	4	1	1
1	5	0	1	1

8	2	4	1	1
0	2	0	1	0
17	2	0	1	1
8	9	1	1	1
1	2	2	1	1
2	4	2	1	0
13	7	0	1	0
0	1	1	1	0
8	1	0	1	1

0	1	0	1	0
0	2	0	0	0
9	2	0	0	1
0	1	1	1	0
7	6	0	1	1
3	4	1	1	1
0	5	1	1	0
0	3	0	1	0
6	2	1	1	1

8	2	0	1	1
0	2	3	1	1
0	10	2	1	1
8	4	0	1	1
16	1	3	0	1
0	2	5	1	0
0	3	2	1	1
8	4	1	0	1
0	1	0	1	1

0	2	2	0	0
12	3	2	1	1
0	0	1	0	1
7	4	0	0	0
0	4	0	1	1
4	2	2	1	1
1	4	1	1	0
2	6	0	1	1
4	1	3	1	1

12	6	0	1	1
10	5	0	1	1
4	7	0	1	1
2	3	2	1	1
2	8	0	1	1
6	2	1	1	1
0	3	2	1	1
0	5	2	1	1
0	3	2	0	1

0	2	1	1	1
0	3	1	1	0
0	3	2	1	1
12	7	1	1	1
0	4	0	1	1
5	5	0	0	1
0	2	3	0	0
0	2	0	1	0
2	2	0	1	1

0	2	0	1	1
0	5	1	0	1
0	1	2	1	1
3	6	3	1	1
4	3	1	1	1
1	3	3	1	1
0	3	0	1	1
0	1	2	0	1
0	1	0	0	0

12	1	0	1	1
0	1	0	1	1
0	1	0	1	0
2	1	0	1	1
0	1	2	1	1
2	1	1	0	1
0	2	3	1	1
0	1	0	1	0
0	2	1	1	1

1	3	3	1	1
18	4	0	1	1
0	1	0	0	0
0	3	2	1	0
0	3	2	1	1
0	3	2	1	1
0	1	2	1	1
0	2	1	1	1
0	2	3	1	1

0	0	4	0	1
0	2	0	0	0
0	4	2	1	0
0	3	5	1	1
3	2	0	1	1
0	4	2	1	1
0	0	0	1	1
0	2	2	1	1
0	0	1	1	0

10	5	0	1	1
0	5	2	1	1
5	5	0	1	1
1	4	0	1	1
2	8	1	1	1
0	4	1	1	1
4	5	0	1	1
0	1	4	1	0
1	4	1	1	1

1	4	0	1	1
4	5	0	1	1
1	6	0	1	1
0	4	4	1	1
5	5	1	1	1
0	4	4	1	1
0	4	2	1	1
10	7	0	1	1
9	7	1	1	1

0	6	0	1	1
0	4	0	1	0
8	3	5	1	0
6	3	2	1	1
0	5	2	0	0
0	1	1	1	1
0	4	1	1	0
0	3	4	1	0
0	2	1	1	1

0	3	1	1	1
0	1	0	1	1
0	2	4	1	1
6	1	1	1	1
0	3	1	1	0
14	8	0	1	1
10	8	0	1	1
0	2	0	1	0
0	4	1	1	1

0	1	1	1	1
0	2	2	0	1
0	4	4	0	1
9	2	0	1	0
6	2	0	1	1
6	3	1	1	1
12	3	0	1	1
0	0	0	1	0
0	2	0	1	0

3	3	3	1	1
0	1	3	1	1
9	6	1	1	1
5	3	3	1	1
14	4	0	1	1
8	4	0	1	1
0	2	0	1	0
24	5	2	1	1
0	1	0	1	1

8	6	0	1	0
0	4	0	0	0
0	1	2	0	1
0	2	1	1	0
0	0	1	1	1
0	1	2	1	0
9	0	3	1	1
0	1	2	1	0
0	1	0	0	0

0	1	2	1	0
0	0	1	0	0
0	1	2	1	1
0	1	0	1	1
0	1	3	1	1
0	0	1	1	1
0	2	1	1	0
0	0	0	1	1
0	7	0	1	1

0	6	0	1	0
0	1	2	1	1
0	1	0	1	0
10	4	2	1	1
0	3	2	1	1
9	2	0	1	1
0	0	1	1	0
0	5	1	1	1
0	6	0	1	1

0	4	0	1	1
2	3	0	1	1
0	4	0	1	1
0	6	5	1	1
0	1	3	1	1
0	2	0	1	1
0	0	3	1	0
0	0	1	0	0
3	1	3	1	1

6	1	0	1	1
4	1	0	1	1
0	2	0	1	0
0	0	2	1	0
0	2	1	0	1
8	4	3	1	1
0	3	2	0	1
3	2	1	0	1
0	2	3	1	1

0	2	0	0	1
0	3	0	1	1
0	1	0	0	1
18	5	3	1	0
0	3	1	1	1
0	2	4	0	0
22	4	0	1	1
6	3	0	1	1
3	3	0	1	1

0	2	2	1	0
0	2	3	1	0
0	9	1	1	1
10	3	0	0	1
9	7	0	1	1
0	3	0	1	0
0	3	3	1	0
0	7	0	0	1
0	8	0	1	1

0	5	3	0	0
0	4	0	1	0
2	2	2	0	0
0	4	3	1	1
0	2	1	1	0
0	1	1	1	0
0	2	0	1	0
0	3	0	1	1
0	1	0	1	1

3	1	0	1	1
3	1	0	0	1
0	2	1	1	0
0	5	2	1	1
0	1	1	1	1
8	1	0	1	0
8	5	2	1	1
4	3	1	1	0
8	1	0	1	0

0	2	2	1	1
0	0	0	1	1
0	0	0	0	1
0	1	2	1	1
8	1	2	0	1
0	1	1	1	0
0	1	0	1	0
0	1	1	0	1
0	2	3	0	0

0	1	2	0	1
0	3	0	1	1
1	3	1	1	1
0	1	1	0	1
0	1	0	1	1
0	2	3	1	1
5	3	0	1	0
0	3	0	1	1
0	1	1	1	1

0	0	0	1	1
0	5	6	1	1

al_menos_una_comorb_psiq	hospitalizado_por_depresion	intento_suicidio	antecedentes_depresion_anteriores	VARIABLES_RESPUESTA_TIEMPO
1	0	0	1	0
1	0	1	0	0
1	0	0	1	0
0	0	0	0	0
1	0	0	0	0
1	0	0	1	0
1	0	0	1	0
1	0	0	1	0

1	0	0	1	0
1	1	1	1	0
1	0	0	1	0
1	0	0	1	0
1	0	0	1	0
1	0	1	1	0
1	0	0	1	0
1	0	1	1	0
1	0	0	1	0

1	0	0	1	0
1	0	0	1	0
1	0	0	1	0
1	1	1	1	0
1	0	0	1	0
1	0	1	1	0
1	0	0	0	0
0	0	0	0	0
1	0	0	1	0

1	0	1	1	0
1	0	1	1	0
1	0	0	1	0
1	0	0	1	0
1	0	1	1	0
1	0	0	0	0
1	0	0	1	0
1	0	0	1	0
1	0	1	1	0

1	0	0	0	0
1	0	0	0	0
1	0	1	1	0
1	0	0	0	0
1	0	0	1	0
1	0	0	0	0
1	0	0	1	0
1	0	0	0	0
1	0	0	1	0

1	0	0	1	0
1	0	0	0	0
1	0	1	1	0
1	0	1	1	0
0	0	1	0	0
0	0	0	0	0
1	0	1	1	0
1	0	1	1	0
1	0	0	1	0

1	0	1	1	0
1	0	1	1	0
1	0	0	0	0
1	0	0	0	0
0	0	0	0	0
1	0	0	1	0
1	0	0	1	0
1	0	0	1	0
1	0	1	1	0

1	0	0	1	0
1	0	1	0	0
1	0	0	0	0
1	0	0	0	0
1	0	0	1	0
1	0	1	1	0
1	1	1	1	0
1	0	1	1	0
0	0	0	0	0

0	0	1	0	0
0	1	1	1	0
1	0	1	0	0
1	0	1	0	0
1	0	0	0	0
1	1	1	1	0
1	0	0	1	0
1	0	0	0	0
1	0	0	1	0

1	0	0	1	0
1	0	1	1	0
1	0	0	1	0
0	0	0	0	0
1	1	1	0	0
1	0	0	1	0
0	0	0	0	0
1	1	1	1	0
1	0	1	1	0

1	0	1	1	0
1	0	1	1	0
1	0	0	1	0
0	0	0	1	0
1	1	1	1	0
1	0	0	1	0
1	0	0	1	0
1	0	0	1	0
1	0	0	1	0

1	0	0	1	0
1	1	1	1	0
1	0	0	0	0
1	0	0	1	0
1	0	1	1	0
1	0	1	1	0
1	0	0	1	0
1	0	1	1	0
1	0	1	0	0

0	0	0	1	0
1	0	0	1	0
1	0	0	1	0
1	0	1	1	0
1	0	1	0	0
1	0	1	1	0
1	0	0	1	0
1	0	0	1	0
1	0	1	1	0

1	0	0	0	0
1	0	0	1	0
1	0	1	0	0
1	0	0	1	0
1	0	0	0	0
1	0	0	1	0
1	0	0	1	0
1	0	0	0	0
1	0	0	1	0

1	0	0	0	0
1	1	0	1	0
1	0	1	1	0
1	0	1	1	0
1	0	0	1	0
1	0	1	1	0
1	0	0	1	0
1	1	0	0	0
1	0	0	0	0

1	0	1	1	0
1	0	1	1	0
1	0	0	1	0
1	0	0	1	0
1	0	1	1	0
1	0	0	0	0
1	0	0	0	0
1	0	0	1	0
1	0	0	0	0

1	0	0	0	0
0	0	0	0	0
1	0	0	1	0
1	0	0	0	0
1	0	0	0	0
1	0	0	1	0
1	0	0	1	0
1	0	1	1	0
1	0	0	1	0

1	0	0	1	0
0	0	0	1	0
1	0	0	0	0
1	0	1	1	0
1	0	0	0	0
1	0	0	1	0
1	0	1	1	0
0	0	0	0	0
1	0	1	1	0

1	0	0	1	0
0	0	0	0	0
1	0	0	1	0
1	0	0	1	0
1	0	0	1	0
1	0	0	0	0
1	0	0	1	0
1	0	1	1	0
1	0	0	1	0

0	0	0	0	0
1	0	0	0	0
1	0	1	1	0
1	0	0	1	0
1	0	0	1	0
1	0	1	1	0
0	0	0	0	0
1	0	0	1	0
1	0	0	1	0

1	1	1	1	0
1	0	1	1	0
0	0	0	1	0
1	0	1	1	0
1	0	0	1	0
1	0	0	1	0
1	0	0	1	0
1	0	1	1	0
1	0	1	1	0

1	0	0	0	0
1	0	0	0	0
1	0	0	1	0
1	0	0	1	0
1	0	0	1	0
1	0	1	0	0
1	0	0	0	0
1	0	0	1	0
1	0	1	1	0

1	1	1	1	0
1	0	0	1	0
1	0	0	0	0
1	0	0	1	0
1	0	1	1	0
0	0	0	0	0
1	0	1	1	0
1	0	0	1	0
1	0	1	0	0

1	0	0	0	0
1	0	0	0	0
1	0	1	1	0
0	0	0	0	0
1	0	1	1	0
1	0	1	1	0
1	0	0	1	0
1	0	1	0	0
1	1	1	1	0

1	0	0	0	0
0	0	0	1	0
1	0	1	1	0
1	0	0	0	0
0	0	1	1	0
1	0	0	1	0
1	0	0	1	0
1	0	1	1	0
1	0	1	1	0

1	0	0	1	0
1	0	1	1	0
1	0	1	0	0
0	0	1	0	0
1	0	0	1	0
1	0	1	1	0
1	0	0	0	0
1	0	1	1	0
0	0	1	0	0

1	0	0	0	0
1	0	0	0	0
1	0	0	0	0
1	0	0	1	0
1	1	1	1	0
1	0	0	0	0
1	0	0	1	0
1	0	0	1	0
0	0	0	1	0

1	0	0	1	0
0	0	0	0	0
1	0	0	0	0
1	0	0	0	0
1	0	0	1	0
1	0	0	0	0
1	0	0	1	0
1	0	1	1	0
1	0	0	1	0

1	0	0	0	0
1	0	0	0	0
1	0	0	1	0
1	0	0	1	0
1	0	0	1	0
1	0	0	1	0
1	0	1	1	0
1	0	0	1	0
1	0	1	0	0

0	0	0	1	0
0	0	0	0	0
1	0	1	1	0
1	0	0	0	0
0	0	1	0	0
1	0	0	0	0
1	0	0	1	0
0	0	0	0	0
1	0	0	1	0

0	0	0	0	0
1	0	1	0	0
1	0	0	0	0
0	0	0	1	0
1	0	0	1	0
1	0	1	1	0
1	0	1	1	0
1	0	1	1	0
1	0	0	0	0

1	0	0	0	0
1	0	0	0	0
1	0	0	1	0
1	1	0	1	0
1	0	0	1	0
1	0	1	1	0
1	0	0	1	0
1	0	0	1	0
0	0	0	1	0

1	0	0	1	0
1	0	0	1	0
1	0	1	1	0
1	0	0	1	0
1	0	0	1	0
1	0	0	1	0
1	0	1	1	0
1	0	0	0	0
1	0	0	0	0

1	0	1	1	0
1	1	0	1	0
1	0	0	1	0
1	0	0	1	0
1	0	0	1	0
1	0	0	1	0
1	1	1	1	0
1	0	1	1	0
1	0	0	1	0

1	0	1	1	0
1	0	0	1	0
0	0	0	0	0
1	0	0	1	0
1	0	0	0	0
0	0	1	0	0
1	0	0	0	0
1	0	0	1	0
1	0	1	1	0

1	0	0	0	0
1	0	1	1	0
1	0	1	0	0
1	0	0	0	0
0	0	0	0	0
1	0	1	1	0
1	0	0	1	0
0	0	1	0	0
1	1	1	1	0

1	0	0	1	0
1	0	0	1	0
1	0	0	1	0
1	0	0	1	0
1	0	1	1	0
1	0	0	0	0
1	0	0	1	0
0	0	0	0	0
1	0	0	1	0

1	0	0	0	0
0	0	1	0	0
1	0	0	0	0
1	0	0	1	0
1	0	1	1	0
1	0	1	1	0
1	0	0	1	0
1	1	1	1	0
1	0	0	1	0

1	0	0	1	0
1	0	0	0	0
0	0	0	0	0
0	0	0	0	0
0	0	0	0	0
1	0	0	0	0
1	0	0	0	0
1	0	0	1	0
1	0	1	1	0

1	0	0	0	0
0	0	1	1	0
1	0	1	1	0
1	0	1	0	0
0	0	0	0	0
0	0	0	1	0
0	0	0	1	0
1	0	1	1	0
1	0	0	1	0

1	0	1	1	0
0	0	0	0	0
1	0	0	1	0
1	0	0	1	0
1	0	1	0	0
1	0	0	1	0
1	0	0	0	0
1	0	0	0	0
1	0	1	1	0

1	0	0	0	0
1	0	0	1	0
1	0	1	1	0
1	1	0	1	0
1	0	0	0	0
1	0	1	1	0
1	0	0	1	0
0	0	0	0	0
1	0	1	1	0

0	0	0	0	0
1	0	0	1	0
0	0	0	0	0
1	0	0	1	0
1	0	0	0	0
1	0	1	1	0
0	0	0	0	0
1	0	0	0	0
1	0	1	1	0

1	0	1	1	0
1	0	0	1	0
1	0	0	1	0
1	0	1	1	0
1	0	1	1	0
1	0	0	0	0
1	0	1	1	0
1	0	0	1	0
1	0	1	1	0

1	0	0	1	0
1	0	1	1	0
1	0	1	1	0
1	0	0	1	0
1	0	1	1	0
1	0	0	1	0
1	0	1	1	0
1	0	0	1	0
1	0	1	1	0

1	0	1	1	0
1	0	1	1	0
1	0	0	1	0
1	0	0	0	0
1	0	0	0	0
0	0	0	0	0
1	0	0	0	0
1	0	1	0	0
1	0	0	1	0

0	0	1	0	0
0	0	0	0	0
0	0	0	0	0
1	0	0	1	0
1	0	0	0	0
0	0	0	1	0
1	0	1	1	0
1	0	1	1	0
1	0	0	1	0

1	0	1	1	0
1	0	1	1	0
0	0	0	0	0
0	0	0	1	0
1	0	0	1	0
1	0	0	0	0
1	0	1	1	0
0	0	0	0	0
0	0	0	0	0

0	0	0	1	0
1	0	1	1	0
1	0	0	1	0
1	1	1	0	0
1	0	0	1	0
1	0	1	1	0
0	0	0	0	0
1	1	1	1	0
1	0	0	1	0

1	0	0	0	0
1	0	0	1	0

hamilton_0	hamilton_3	hamilton_6	hamilton_9	hamilton_12
24	11	18	20	3
26	24	25	18	18
13	14	6	7	3
28	13	13	1	4
18	15	6	3	3
27	10	21	13	15
20	15	19	15	11
25	14	12	14	1

16	12	18	15	13
20	4			
20	4	19	3	2
18	14	6	0	2
16	7	5	1	4
14	4	2	1	3
25	11	18	14	13
16	17	3	8	16
17	0	15	9	14

21	6			
21	9	9	7	8
10	7	8	4	2
25	16	16	16	20
20	9	9	2	2
31	19	24	17	17
21	5			
11	2	2	0	0
11	9	22	6	9

34	8	3	3	4
27	19	9	14	14
15	4	2	7	3
31	19	22	9	4
19	13	12	15	24
14	9	6	0	1
17	8	6	5	5
14	10	6	6	7
20	15			

8	7	5	5	7
14	15	17	17	11
16	9	8	13	7
17	5	9	2	1
12	7	9	13	7
14	5	1	4	3
20	4	4	8	12
14	1	4	4	5
20	13	15	19	12

29	16	10	4	10
17	7	2	6	5
27				
23	9	5	25	9
17	12	18	14	22
23	7	7	0	1
20				
20	6	17	22	15
20	20	14	17	19

20	24	14	6	11
20	21	17	29	20
8	17	11	6	8
7	4	10	0	1
20				
14	6			
10			0	
8	6	10	4	4
28				

7	9			
7	6	9	7	5
19	8	15	14	19
8	13	22	11	14
18	10	8	3	7
17	12	12	14	15
11	10	10	3	3
14				
17	0			

12	18	16	15	16
10				
29	8			
19	19	18	15	16
17				20
26				
22	25			
21	2	0	16	5
20	12			11

20	8	9	7	7
25	11	16	3	5
23	16			
9	8	7	5	7
25	20	19	24	21
27	18			
15	10			
25				
28	22			

18	3	2	0	0
33				
29	19	19	12	12
23	4	11	0	0
29	17	19	25	13
26	8	13	10	9
35	13	15	22	11
20	9	12	12	12
33	18	18	18	18

23	5	2	0	0
30				
19				
12	16	19	16	14
25	18	28	18	8
23	17	14	17	20
12				
15	15	15	15	16
34	32	30	32	34

6		10		
7		2		
18	14	11	6	0
24	18	19	17	11
15	12	9	3	27
25	9	1	6	3
11	0	0	0	0
30	12	17	16	15
29	12	24	18	18

24	15	8	11	12
21	6	21	1	4
28	16	10	10	18
24	18	18	13	16
19	15	20	19	22
14	11	10	4	3
25	4	1	4	4
16	5	2	23	8
15	14	15	22	5

27	7	0	0	0
30	22	2	6	1
15	8	15	19	18
32				
22	3	7	20	0
23	14	15	12	9
19	10	15	11	11
22	6	8	3	2
12	7	7	3	0

19	6	7	6	5
22				
15	12			
25	8	8	2	4
24	21	16	18	14
17	10	17	6	9
9	5	2	2	3
10	3	2	1	1
15	6	8	8	4

23	8	2	4	5
21	5	5	0	2
25				
12	1	3	1	19
11	8	12	8	17
18	18	17	16	19
21	7	4	5	12
22	12			
19	8	0	0	0

15	8	11	2	0
24	1	1	1	1
13	10	7	3	5
13	8	6	10	16
22	12	5	8	8
20	2	1	1	0
25	3	2	4	0
18				
31				

27	13	24	20	25
22		4		
24	10	3	19	3
26				
26	7			
23	6	4	13	1
24	24	12	18	17
19	8	9	9	2
22	23	16	11	7

18	4	13	1	0
18	1	0	0	1
28	18	27	23	23
21	11	7	0	2
24	17	10	11	7
20	6	5	9	6
22	5	6	0	8
18	13	12	18	10
19	17	7	4	9

29				
24				
10	14	21	9	15
23		3		
10				
18		23		
16	10	7	11	9
29				
14	11	6	0	0

18				
22	12	2	0	0
14	7	0	3	7
16	15	4	8	8
18	5	13	2	1
14	4	4	5	6
16	20	25	3	2
23	11	11	10	8
23	5	18	13	12

33	23	8	14	15
21	11	9	11	14
16	1	1	9	18
10	1	4	11	16
12	10	18	10	3
15	4	6	4	2
7	0	1	0	2
27	0	6	12	19
12				

4	0	7	0	0
11	4	5	4	3
13			14	
22	17	2	22	26
26	12			
26	15	5	12	12
34	23	23	18	29
25	11	7	4	7
19	10	3	12	8

11	8	8	8	8
7	12	18	0	2
32	23	18	15	19
30	14	24	5	5
23	2	0	2	2
20	16	17	16	16
16	9	5	7	13
22	24	5	18	16
27	20	8	4	0

16	12	9	4	10
23	18	0	13	24
17	2	16	0	11
17	7	6	3	1
21	9	11	14	10
15	20	8	3	8
24				
27	10	13	10	8
20	10	9	2	7

15	0	17	0	6
22	14	4	10	12
16	14			
18	4	7	4	7
25	11	2	8	3
25	15	5	15	8
17	7	4	5	16
20	4	3	10	17
15	5	14	8	8

21	3	13	3	21
8	7	7	6	4
24	13	5	3	15
29	4	3	5	5
14	10	5	7	7
18	7	4	1	3
19	4	4	6	1
16	19	19	19	19
24	9	19	1	0

16	21	7	13	6
28	4	7	4	2
28	11	7	4	7
17	13			
11				
30	20	7	11	25
25	25			
20	5	18	20	22
16	1	0	0	0

7	2	0	0	0
34	20	11	24	23
32	14	2	3	0
26	3	12	0	0
17	9	8	13	0
23	16	7	6	5
26	6	25	2	6
31	6	4	2	1
24	12	10	2	0

30	29	27	28	21
27				
10	2	0	0	0
14	9	4	4	1
18	0	0	0	0
25	21	22	22	22
14	18	3	10	8
23	15	4	11	6
18				1

21	3	3	0	0
17	1	0	1	0
15	0	9	0	1
11	11	1	11	0
11	8	6	8	3
19	3	2	7	2
19	12	0	10	4
20	7	2	6	10
2	0	0	0	0

28				
24	22	5	4	1
28	5	2	3	3
26	5	15	11	0
24	1	0	0	0
19	11	8	4	7
28				
16	11			
20	17	6	16	12

20				
30				
16	8	15	9	4
28	10	13	8	0
18	13	11	11	12
15	15	15	2	6
20	15	10	7	9
25	23			
23	12	3	5	7

20	12	10	16	0
13	8	16	0	5
20				
14	5	13	0	6
16	21	10	13	9
25	14	8	15	11
19	13	16	6	7
17	11	11	7	9
15	5	10	6	13

21	19	12	14	10
19	15	4	7	2
19	15	3	7	1
20	12	19	4	0
11	4	3	0	0
25	5	3	5	7
29	14	7	11	13
12	6	8	0	4
22	22	2	12	12

16	13	7	5	5
17	3	10	17	6
5	7	12	1	0
21	10	8	6	6
20	0	5	5	5
32	2	0	4	9
20	18	10	2	2
29	10	10	3	0
21				

19	8	17	3	1
26	15			
19	13	15	14	14
29	32	10	14	1
29	6	21	20	0
31	16	6	10	15
22	21	0	23	18
28	13	5	20	5
15	10	11	5	12

22	3	3	0	0
20	16	0	0	9
4	0	10	1	3
24	3	2	0	0
30	0	2	4	2
32	2	6	2	3
32	9	12	6	6
25	7	0	4	1
35				

16	1	4	3	5
9	1	0	0	0
28	8	3	20	8
32	0	0	0	0
9	10	3	0	0
13	2	2	2	0
9	5	1	6	0
26	9			
18	6	3	5	7

19	8	12	10	10
9	2	13	3	6
15	10	6	12	10
22	27			
12				
17				
19	7	12	12	16
24	19	11	6	29
18	9	5	17	0

15	12	17	15	9
22	12	10	11	11
17	14	3	8	8
28	0	1	20	22
27	17	0	8	3
35	18	18	18	18
14	4	3	2	2
7	0	0	0	0
27	0	0	0	0

24	2	1	0	1
23	3	17	15	0
20	0	23	0	0
22	0	15	0	0
21	10	10	10	10
31	21	20	18	19
8	0	4	0	13
21	7	1	5	2
24	12	8	3	20

21	0	3	1	3
15	0	6	1	0
29	12	12	12	13
21	7	0	2	0
30	12			
16	3	0	0	5
21	7	3	3	3
26	26	21	22	24
15	1	1	1	1

30	15	15	15	13
13	3	6	3	3
15	0	0	0	0
24	18	18	20	22
18				
8	0	18	0	0
23	20	24	10	20
23				
22	18	18	23	28

24	22	17	22	27
20	14	14	13	13
9	0	0	0	6
24	6	1	6	12
14	4	6	4	1
9	0	0	0	0
15	4	6	4	1
10		6		
19				

19				
18			9	
1	1	3	0	0
21	14	9	6	8
16	6	17	17	11
18	17	6	0	0
12	8	1	4	2
18	7			
21				

23	18	12	7	10
25	22			
12				
19	4	2	2	1
27	2	2	2	2
25	3	5	1	6
17	0	0	17	5
9	3	0	0	0
16	2	3	8	2

7	2	0	0	1
20	1			
11				
28	2	3	5	3
28	1			
23				
13				
22				
20				

20

20

p_hamilton_3m	p_hamilton_6m	p_hamilton_9m	p_hamilton_12m	hamilton inicial
1	0	0	1	24
0	0	0	0	26
0	1	0	1	16
1	1	1	1	28
0	1	1	1	18
1	0	1	0	27
0	0	0	0	20
0	1	0	1	25

0	0	0	0	16
1	0	0	0	20
1	0	1	1	20
0	1	1	1	18
1	1	1	1	16
1	1	1	1	14
1	0	0	0	25
0	1	1	0	16
0	0	0	0	17

1	0	0	0	21
1	1	1	1	21
0	0	1	1	10
0	0	0	0	25
0	1	1	1	20
0	-4	-2	-2	31
1	0	0	0	21
1	1	1	1	11
0	-1	0	0	11

1	1	1	1	34
0	1	0	0	27
1	1	1	1	15
0	0	1	1	31
0	0	0	0	19
0	1	1	1	14
1	1	1	1	18
0	1	1	1	14
0	0	0	0	20

0	0	0	0	8
0	0	0	0	14
0	1	0	1	16
1	0	1	1	17
0	0	0	0	12
1	1	1	1	14
1	1	1	0	20
1	1	1	1	14
0	0	0	0	20

0	0	1	0	29
1	1	1	1	17
0	0	0	0	27
1	1	0	1	23
0	0	0	0	17
1	1	1	1	23
0	0	0	0	19
1	0	0	0	18
0	0	0	0	12

0	0	1	0	20
0	0	0	0	20
0	0	1	1	8
1	1	0	1	7
0	0	0	0	20
1	0	0	0	14
0	0	0	0	10
1	1	1	1	8
0	0	0	0	28

1	0	0	0	7
0	0	0	0	7
1	0	0	0	19
-1	-2	0	-1	8
0	1	1	1	18
0	0	0	0	17
0	0	1	0	11
0	0	0	0	14
0	0	0	0	17

-1	0	1	0	12
0	0	0	0	10
1	0	0	0	29
0	0	0	0	19
0	0	0	0	17
0	0	0	0	26
0	1	0	0	22
1	1	0	1	21
0	0	0	0	20

1	1	1	1	20
1	0	1	1	25
0	0	0	0	23
0	0	0	0	9
0	0	0	0	25
0	0	0	0	27
0	0	0	0	15
0	0	0	0	25
0	0	0	0	28

1	1	0	0	18
0	0	0	0	33
0	0	1	1	19
1	1	0	1	23
0	0	0	1	29
1	1	0	1	26
1	1	0	1	35
1	0	0	0	20
0	0	0	0	33

1	1	1	1	25
0	0	0	0	30
0	0	0	0	19
0	-1	0	0	12
0	0	0	1	25
0	0	0	0	23
0	0	0	0	12
0	0	0	0	15
0	0	0	0	34

0	-1	0	0	6
0	1	0	1	7
0	0	0	1	18
0	0	0	1	24
0	0	1	-1	15
1	1	1	1	25
0	1	0	1	11
1	0	0	0	30
1	0	0	0	29

0	1	1	1	24
1	0	1	1	21
0	1	1	0	28
0	0	0	0	24
0	0	0	0	19
0	0	1	1	14
1	1	1	1	25
1	1	0	1	16
0	0	0	1	15

1	1	1	1	27
0	1	1	1	30
0	0	0	0	15
0	0	0	0	32
1	1	0	1	22
0	0	0	1	23
0	0	0	0	19
1	1	1	1	22
0	0	0	1	12

1	1	1	1	19
0	0	0	0	22
0	0	0	0	15
1	1	1	1	25
0	0	0	0	24
0	0	1	0	17
0	1	1	1	9
1	1	1	1	10
1	0	0	1	15

1	1	1	1	23
0	1	1	1	21
0	0	0	0	25
1	1	1	-1	12
0	0	0	-1	11
0	0	0	0	18
1	1	1	0	21
0	0	0	0	22
1	1	1	1	19

0	0	1	1	15
1	1	0	0	24
0	0	1	1	13
0	1	0	0	13
0	1	0	0	22
1	0	1	1	20
0	1	1	1	25
0	0	0	0	18
0	0	0	0	31

1	0	0	0	27
0	1	0	0	22
1	1	0	1	24
0	0	0	0	26
1	0	0	0	26
1	1	0	1	23
0	1	0	0	24
1	1	1	1	19
0	0	1	1	23

1	0	1	1	18
1	1	0	1	18
0	0	0	0	28
0	1	1	1	21
0	1	0	1	24
1	1	1	1	20
1	1	1	1	22
0	0	0	0	18
0	1	1	1	19

0	0	0	0	29
0	0	0	0	24
0	-1	-1	0	10
0	1	0	0	23
0	1	0	0	10
0	0	0	0	18
0	1	0	0	18
0	0	0	0	29
0	1	1	1	14

0	0	0	0	18
0	1	1	1	22
1	0	1	1	14
0	1	0	0	18
1	0	1	1	18
0	0	1	1	14
0	-1	1	1	16
1	1	1	1	23
1	0	0	0	23

0	1	1	1	33
0	1	0	0	21
0	1	0	0	16
0	0	0	-1	10
0	-1	0	1	12
0	1	0	1	15
0	1	0	1	7
0	1	0	0	27
0	0	0	0	12

0	-1	0	1	4
0	1	0	1	11
0	0	0	0	13
-1	1	0	-3	22
1	0	0	0	26
0	1	1	1	26
0	0	0	0	34
1	0	1	1	25
0	1	0	1	19

0	0	1	1	11
-1	-2	1	1	7
0	0	1	0	32
0	0	1	1	24
1	0	1	0	23
0	0	0	0	20
0	1	1	0	16
0	1	0	0	22
0	1	1	1	23

0	0	1	0	16
0	0	0	0	23
1	0	1	0	17
1	1	1	1	17
1	0	0	1	21
0	0	1	0	15
0	0	0	0	24
0	1	0	1	27
1	1	1	1	20

1	0	1	1	15
0	1	0	0	22
0	0	0	0	16
1	1	1	1	18
1	1	0	1	25
0	1	0	1	25
1	1	1	0	17
1	1	1	0	20
1	0	0	0	15

1	0	1	0	21
0	0	0	1	8
0	1	1	0	24
1	1	1	1	29
0	1	1	1	14
1	1	1	1	18
1	1	1	1	19
0	0	0	0	16
1	0	1	1	24

0	1	0	1	16
1	1	1	1	28
1	0	1	1	28
0	0	0	0	17
0	0	0	0	11
0	1	1	0	30
0	1	0	0	25
1	0	0	0	20
1	1	1	1	16

1	0	0	0	7
0	1	0	0	34
1	1	1	1	26
1	1	1	1	32
0	1	0	0	17
0	1	1	1	23
1	0	1	1	26
1	1	1	1	31
1	1	1	0	24

0	1	0	0	30
0	0	0	0	27
1	0	0	0	10
0	1	1	1	14
0	0	0	0	18
0	0	0	0	25
0	1	0	0	14
0	1	1	1	23
0	0	0	1	18

1	1	0	1	21
1	0	1	0	17
1	0	1	1	15
0	1	0	1	11
0	0	0	1	11
0	1	1	1	19
0	0	0	1	19
1	1	1	1	20
0	0	0	0	2

0	0	0	0	28
0	1	1	0	24
1	1	0	0	28
1	0	1	1	26
1	0	0	0	24
0	1	1	1	19
0	0	0	0	28
0	0	0	0	16
0	1	0	0	20

0	0	0	0	20
0	0	0	0	30
1	0	0	1	16
1	1	0	1	28
0	0	0	0	18
0	0	1	1	15
0	1	1	1	20
0	0	0	0	25
0	1	1	1	23

0	1	0	0	20
0	0	0	1	13
0	0	0	0	20
1	0	1	1	14
0	0	0	0	16
0	1	0	1	25
0	0	1	1	19
0	0	1	0	17
1	0	1	0	15

0	0	0	1	21
0	1	1	1	19
0	1	1	1	19
0	0	1	1	20
1	1	1	1	11
0	0	0	1	25
1	1	0	1	29
1	0	1	1	12
0	1	0	0	25

0	1	1	1	16
1	0	0	1	18
1	0	1	1	5
-1	-1	0	0	21
0	0	0	1	20
1	0	1	1	32
0	0	1	1	20
1	1	1	1	29
0	0	0	0	21

1	0	1	1	19
0	0	0	0	30
0	0	0	0	19
0	0	0	1	29
1	0	0	0	29
0	1	1	1	31
0	1	0	0	22
1	1	0	1	28
0	0	1	0	15

1	0	0	1	22
0	0	0	1	22
0	-2	1	0	4
1	1	1	1	24
1	0	1	0	30
1	1	1	0	32
1	1	1	1	32
1	1	1	1	25
0	0	0	0	35

1	1	1	1	16
1	0	0	1	9
1	1	0	1	28
1	1	1	1	32
0	1	0	0	9
1	1	1	0	13
0	1	0	0	9
1	0	0	0	26
1	1	1	0	18

1	0	0	0	19
1	0	1	0	9
0	1	0	0	15
0	0	0	0	22
0	0	0	0	12
0	0	0	0	17
1	1	0	0	19
0	1	1	0	24
1	1	0	0	18

0	0	0	0	15
0	1	0	0	22
0	1	0	0	17
0	1	0	0	28
0	1	1	1	27
1	0	0	0	35
1	0	1	1	14
1	0	1	1	7
1	0	1	1	27

1	0	1	0	26
1	0	0	1	23
1	0	1	0	22
1	0	1	1	22
0	0	1	1	21
0	0	0	0	31
1	1	1	-1	8
1	1	1	1	21
1	1	1	0	24

1	1	1	1	21
1	1	1	1	15
1	0	0	1	29
1	0	1	1	29
1	0	0	0	30
1	1	1	1	16
1	0	0	0	21
0	1	0	0	26
0	1	0	1	15

0	1	0	1	30
0	1	0	0	15
0	0	0	1	15
0	0	0	0	24
0	0	0	0	18
0	-1	0	1	8
0	0	0	0	23
0	0	0	0	23
0	1	0	0	23

0	0	0	0	23
0	0	0	0	20
0	1	0	0	9
0	1	0	1	24
0	1	0	1	14
0	1	0	1	9
0	0	0	1	15
0	-1	0	0	10
0	0	0	0	19

0	0	0	0	19
0	0	1	0	18
0	-2	1	1	1
0	1	1	1	21
1	1	1	0	16
0	1	1	0	18
0	1	1	1	12
1	0	0	0	18
0	0	0	0	21

0	0	1	1	28
0	0	0	0	25
0	0	0	0	12
1	1	1	1	19
1	0	1	1	27
1	1	1	1	25
1	1	0	1	17
1	1	1	1	9
1	1	1	1	16

1	0	1	1	7
1	0	0	0	20
0	0	0	0	11
1	0	1	1	28
1	0	0	0	28
0	0	0	0	23
0	0	0	0	13
0	0	0	0	22
0	0	0	0	20

0	0	0	0	20
0	0	0	0	20

estado remision 3	estado remision 6	estado remision 9	estado remision 12	total_remisiones
2	3	3	1	1
3	3	3	3	0
3	1	1	1	3
2	2	1	1	2
2	1	1	1	3
2	3	2	2	0
3	3	3	2	0
2	2	2	1	1

3	4	3	3	0
1	0	0	0	1
1	3	1	1	3
3	1	1	1	3
1	1	1	1	4
1	1	1	1	4
2	3	2	2	0
3	1	1	3	2
3	2	3	3	0

0	0	0	0	1
2	2	1	1	2
1	1	1	1	4
2	2	2	3	0
2	2	1	1	2
3	3	3	3	0
1	0	0	0	1
1	1	1	1	4
3	4	1	3	1

1	1	1	1	4
3	2	2	2	0
1	1	1	1	4
3	3	2	1	1
3	3	3	4	0
3	1	1	1	3
1	1	1	1	4
3	1	1	1	3
3	0	0	0	0

1	1	1	1	4
3	4	4	3	0
2	1	3	1	2
1	1	1	1	3
1	3	3	1	2
1	1	1	1	4
1	1	1	2	3
1	1	1	1	4
3	3	3	3	0

2	2	1	2	1
1	1	1	1	4
0	0	0	0	0
2	1	3	1	1
3	3	3	4	0
1	1	1	1	4
0	0	0	0	0
1	3	4	3	1
4	3	4	4	0

4	3	1	2	1
3	3	4	3	0
4	4	1	1	2
1	4	1	1	2
0	0	0	0	0
1	0	0	0	1
0	0	0	0	0
1	4	1	1	3
0	0	0	0	0

4				0
1	4	1	1	3
1	2	2	2	1
4	4	4	4	0
3	1	1	1	3
3	3	3	3	0
3	3	1	1	1
0	0	0	0	0
0	0	0	0	0

4	4	4	4	1
0	0	0	0	0
1	0	0	0	1
3	3	3	3	0
0	0	0	4	0
0	0	0	0	0
3	1	0	0	1
1	1	3	1	3
2	0	0	2	0

1	1	1	1	3
2	3	1	1	2
3	0	0	0	0
1	1	1	1	4
3	3	3	3	0
3	0	0	0	0
3	0	0	0	0
0	0	0	0	0
3	0	0	0	0

1	1	0	0	2
0	0	0	0	0
3	3	2	2	0
1	2	1	1	2
3	3	3	2	0
1	2	2	2	1
2	2	3	2	0
2	3	2	2	0
2	2	2	2	0

1	1	1	1	4
0	0	0	0	0
0	0	0	0	0
4	4	4	4	0
3	4	3	1	1
3	3	3	3	0
0	0	0	0	0
3	3	3	3	0
3	3	3	3	0

	4			0
	1			2
3	3	1	1	1
3	3	3	2	0
3	2	1	4	1
2	1	1	1	3
1	1	1	1	2
2	2	2	2	0
2	3	3	3	0

3	1	2	2	1
1	3	1	1	3
3	2	2	3	0
3	3	2	3	0
3	3	3	4	0
3	3	1	1	2
1	1	1	1	4
1	1	4	1	3
3	3	4	1	1

1	1	1	1	4
3	1	1	1	3
1	3	3	3	1
0	0	0	0	0
1	1	3	1	3
3	3	2	2	0
2	3	2	2	0
1	1	2	1	4
1	1	1	1	3

1	1	1	1	4
0	0	0	0	0
3	0	0	0	0
1	1	1	1	4
3	3	3	2	0
3	3	1	2	1
1	1	1	1	4
1	1	1	1	4
1	1	1	1	4

1	1	1	1	4
1	1	1	1	3
0	0	0	0	0
1	1	1	4	3
2	2	1	4	2
3	3	3	3	0
1	1	1	2	3
2	0	0	0	0
1	1	1	1	4

1	3	1	1	3
1	1	1	1	2
3	1	1	1	2
2	1	3	4	2
2	1	1	1	1
1	1	1	1	3
1	1	1	1	3
0	0	0	0	0
0	0	0	0	0

2	3	3	3	0
0	1	0	0	1
2	1	3	1	2
0	0	0	0	0
1	0	0	0	1
1	1	2	1	3
3	2	3	3	0
1	1	1	1	2
3	3	2	1	1

1	3	1	1	3
1	1	1	1	3
3	3	3	3	0
2	1	1	1	3
3	2	2	1	1
1	1	1	1	3
1	1	1	1	4
3	3	3	3	0
1	1	1	1	2

0	0	0	0	0
1	0	0	0	0
4	4	3	3	0
0	1	0	0	1
0	1	0	0	1
0	4	0	0	0
3	1	3	2	1
0	0	0	0	0
3	1	1	1	3

0	0	0	0	0
2	1	1	1	3
1	1	1	1	3
3	1	1	1	1
1	3	1	1	3
1	1	1	1	2
4	4	1	1	2
2	2	2	1	1
1	3	2	2	1

3	1	2	2	1
2	2	2	3	0
1	1	3	4	1
1	1	3	4	0
3	4	3	1	1
1	1	1	1	2
1	1	1	1	2
1	1	2	3	1
0	0	0	0	0

1	4	1	1	2
1	1	1	1	2
2	0	0	0	0
3	1	3	4	1
2	0	0	0	0
3	1	2	2	1
3	3	3	3	0
2	1	1	1	2
2	1	3	1	2

1	1	1	1	4
4	4	1	1	2
3	3	2	3	0
2	3	1	1	2
1	1	1	1	2
3	3	3	3	0
3	1	1	3	2
3	1	3	3	1
3	1	1	1	3

3	1	1	3	1
3	3	1	3	0
3	1	1	3	2
1	1	1	1	4
2	2	3	2	0
4	1	1	1	3
0	0	0	0	0
2	2	2	1	1
2	2	1	1	2

1	3	1	1	3
3	1	2	2	1
3	0	0	0	0
1	1	1	1	4
2	1	1	1	2
3	1	3	1	2
1	1	1	3	3
1	1	2	3	2
1	3	1	1	1

1	1	1	3	2
1	1	1	1	4
2	2	1	3	2
1	2	1	1	4
3	1	1	1	3
1	1	1	1	4
4	4	6	1	4
4	4	4	4	0
2	3	1	1	2

4	1	3	1	2
1	1	1	1	4
2	1	1	1	2
3	0	0	0	0
0	0	0	0	0
3	3	2	3	1
3	3			1
1	3	3	3	1
1	1	1	1	4

1	1	1	1	1
3	3	3	3	0
2	1	1	1	3
1	2	1	1	3
2	1	3	0	1
3	1	1	1	3
2	2	1	1	3
1	1	1	1	4
2	2	1	1	1

3	3	3	3	0
0	0	0	0	0
1	1	1	1	1
2	1	1	1	3
0	0	0	0	0
3	3	3	3	0
4	1	2	1	2
3	2	2	3	2
0	0	0	1	1

1	1	1	1	3
1	1	1	1	2
1	1	1	1	3
2	1	2	1	2
1	1	1	1	3
1	1	1	1	3
3	0	2	1	1
1	1	1	2	3
1	1	1	1	0

0	0	0	0	0
3	1	1	1	2
1	1	1	1	1
1	1	2	1	2
1	0	0	0	1
2	1	1	1	3
0	0	0	0	0
3	0	0	0	0
3	3	3	3	1

0	0	0	0	0
0	0	0	0	0
2	3	2	1	2
2	2	1	1	1
3	3	3	3	0
3	3	1	1	2
3	2	1	1	1
3	0	0	0	0
2	1	1	1	3

3	2	3	0	0
1	3	3	1	2
0	0	0	0	0
1	3	1	1	3
4	3	3	3	0
3	3	2	2	1
3	3	1	1	2
3	3	1	1	1
1	3	1	3	2

3	3	3	2	0
3	1	1	1	3
3	1	1	1	3
3	3	1	1	2
1	1	1	1	4
1	1	1	1	1
2	1	2	2	1
1	1	1	1	4
3	1	2	2	1

1	1	1	1	3
1	2	3	1	2
4	4	1	1	3
2	1	1	1	3
1	1	1	1	1
1	1	1	1	2
3	2	1	1	2
2	2	1	1	2
0	0	0	0	0

1	1	1	1	3
2	0	0	0	0
3	3	3	3	0
3	2	2	1	1
1	3	3	3	1
2	2	2	2	1
3	3	3	3	1
2	1	2	1	2
3	3	1	3	1

1	1	1	1	2
3	1	1	1	0
1	4	1	1	2
1	1	1	1	4
1	1	1	1	2
1	1	1	1	3
1	2	1	1	2
1	1	1	1	4
0	0	0	0	0

1	1	1	1	4
1	1	1	1	2
1	2	3	1	3
1	1	1	1	4
4	1	1	1	1
1	1	1	1	3
1	1	1	1	3
2	0	0	0	0
1	1	1	1	3

1	2	2	2	1
1	1	1	1	3
2	1	2	2	1
4	0	0	0	0
0	0	0	0	0
0	0	0	0	0
1	3	3	3	2
3	2	1	3	1
1	4	4	0	1

3	3	3	2	0
2	2	2	2	0
3	1	1	1	1
3	3	3	3	1
1	1	1	1	3
2	2	2	2	0
1	1	1	1	3
1	1	1	1	3
1	1	1	1	3

1	1	1	0	2
1	3	3	1	2
1	3	1	1	2
1	3	1	1	3
2	2	2	2	2
3	3	3	3	0
1	1	1	4	3
1	3	1	1	4
3	3	3	3	2

1	1	1	1	4
1	1	1	1	4
2	2	2	2	0
1	1	1	1	3
2	0	0	0	0
1	1	1	1	4
1	1	1	1	1
3	3	3	3	1
1	1	1	1	2

3	3	3	3	0
1	1	1	1	1
1	1	1	1	1
3	3	3	3	0
0	0	0	0	0
1	4	1	3	1
3	3	3	3	0
0	0	0	0	0
3	3	4	4	0

3	3	3	4	0
3	3	3	3	0
1	1	1	1	2
1	1	1	2	1
1	1	1	1	2
0	0	0	0	2
1	1	1	1	1
0	1	0	0	0
0	0	0	0	0

0	0	0	0	0
0	0	2	0	0
1	1	1	1	4
3	2	1	1	2
1	3	3	3	3
3	1	1	1	2
1	1	1	1	4
1	0	0	0	1
0	0	0	0	0

3	2	1	2	1
3	0	0	0	0
0	0	0	0	0
1	1	1	1	4
1	0	1	1	3
1	1	1	1	4
1	1	3	1	3
1	1	1	1	4
1	1	1	1	4

1	1	1	1	3
1	0	0	0	1
0	0	0	0	0
1	1	1	1	3
1	0	0	0	1
0	0	0	0	0
0	0	0	0	0
0	0	0	0	0
0	0	0	0	0

0	0	0	0	0
0	0	0	0	0

respuesta_3m	respuesta_6m	respuesta_9m	respuesta_12m	ptgi_3
2	1	0	2	6
0	0	1	1	1
0	2	1	2	3
2	2	2	2	5
0	2	2	2	2
2	0	2	0	0
1	0	1	1	5
1	2	1	2	5

1	0	0	0	5
2	0	0	0	4
2	0	2	2	6
0	2	2	2	5
2	2	2	2	5
2	2	2	2	5
2	1	1	1	5
0	2	2	0	1
0	0	1	0	0

2	0	0	0	5
2	2	2	2	0
1	0	2	2	7
1	0	1	0	5
0	2	2	2	7
0	0	0	0	0
2	0	0	0	6
2	2	2	2	7
0	0	1	0	5

2	2	2	2	6
1	2	0	0	2
2	2	2	2	7
1	1	2	2	2
1	1	0	0	5
1	2	2	2	7
2	2	2	2	7
1	2	2	2	5
1	0	0	0	4

0	1	1	0	5
0	0	0	0	5
1	2	0	2	6
2	1	2	2	7
1	1	0	1	7
2	2	2	2	6
2	2	2	1	5
2	2	2	2	5
1	1	0	1	5

1	0	2	0	6
2	2	2	2	4
0	0	0	0	0
2	2	0	2	6
1	0	0	0	5
2	2	2	2	6
0	0	0	0	0
2	0	0	1	6
0	1	0	0	5

0	1	2	1	2
0	0	0	0	5
0	1	2	2	5
2	2	0	2	5
0	0	0	0	0
2	0	0	0	5
0	0	0	0	0
2	2	2	2	6
0	0	0	0	0

2	0	0	0	2
0	0	0	1	5
2	0	1	0	6
0	0	0	0	5
1	2	2	2	5
1	1	0	0	3
0	0	2	0	2
0	0	0	0	0
0	0	0	0	0

0	0	2	0	6
0	0	0	0	0
2	0	0	0	6
0	0	0	0	4
0	0	0	0	0
0	0	0	0	0
0	2	0	0	2
2	2	0	2	7
1	0	0	1	5

2	2	2	2	7
2	1	2	2	6
1	0	0	0	7
0	0	1	0	6
0	0	0	0	2
1	0	0	0	5
1	0	0	0	6
0	0	0	0	0
0	0	0	0	2

2	2	0	0	6
0	0	0	0	0
1	1	2	2	3
2	2	0	2	7
1	1	0	2	5
2	2	0	2	5
2	2	1	2	5
2	1	1	1	5
1	0	0	1	4

2	2	2	2	6
0	0	0	0	0
0	0	0	0	6
0	0	0	0	6
0	0	0	2	0
0	1	0	0	6
0	0	0	0	4
0	0	0	0	6
0	0	0	0	6

0	0	0	0	6
0	2	0	2	6
0	1	0	2	6
1	0	1	2	4
0	1	2	0	5
2	2	2	2	6
0	2	0	2	7
2	1	1	0	5
2	0	0	0	2

1	2	2	2	5
2	0	2	2	6
1	2	2	1	4
1	1	1	1	2
0	0	0	0	4
0	1	2	2	5
2	2	2	2	6
2	2	0	2	6
0	0	0	2	5

2	2	2	2	6
1	2	2	2	4
1	0	0	0	5
0	0	0	0	0
2	2	0	2	6
1	1	1	2	5
1	0	1	1	5
2	2	2	2	7
1	1	0	2	6

2	2	2	2	4
0	0	0	0	0
0	0	0	0	6
2	2	2	2	5
0	1	1	1	4
1	0	2	1	6
1	2	2	2	7
2	2	2	2	6
2	1	1	2	6

2	2	2	2	5
0	2	2	2	6
0	0	0	0	0
2	2	2	0	6
1	0	1	0	4
0	0	0	0	5
2	2	2	1	6
1	0	0	0	5
2	2	2	2	2

1	1	2	2	5
2	2	0	0	6
0	0	2	2	6
1	2	0	0	6
1	2	0	0	3
2	0	2	2	6
0	2	2	2	7
0	0	0	0	0
0	0	0	0	0

2	0	0	0	6
0	2	0	0	0
2	2	0	2	4
0	0	0	0	0
2	0	0	0	6
2	2	1	2	6
0	2	1	1	1
2	2	2	2	6
0	1	2	2	3

2	1	2	2	6
2	2	0	2	7
1	0	0	0	5
1	2	2	2	5
1	2	0	2	2
2	2	2	2	2
2	2	2	2	4
1	1	0	1	2
0	2	2	2	3

0	0	0	0	0
0	0	0	0	0
0	0	0	0	6
0	2	0	0	0
0	2	0	0	0
0	0	0	0	0
1	2	1	0	6
0	0	0	0	0
0	2	2	2	1

0	0	0	0	0
1	2	2	2	3
2	0	2	2	6
0	2	0	0	6
2	1	2	2	6
0	0	2	2	6
0	0	2	2	0
2	2	2	2	5
2	0	1	0	5

1	2	2	2	1
0	2	0	1	5
0	2	0	0	3
0	0	0	0	5
0	0	0	2	5
0	2	0	2	6
0	2	0	2	5
0	2	0	1	7
0	0	0	0	0

0	0	0	2	7
0	2	0	2	6
0	0	0	0	7
0	2	0	0	4
2	0	0	0	5
1	2	2	2	4
1	0	1	0	0
2	0	2	2	4
1	2	1	2	5

1	1	2	2	0
0	0	2	2	5
1	1	2	1	3
0	0	2	2	0
2	0	2	0	0
0	0	0	0	1
1	2	2	0	5
0	2	0	1	2
1	2	2	2	1

1	1	2	1	6
0	0	1	0	4
2	0	2	1	6
2	2	2	2	6
2	0	1	2	5
0	1	2	1	4
0	0	0	0	0
0	2	0	2	0
2	2	2	2	6

2	0	2	2	7
1	2	0	1	5
0	0	0	0	3
2	2	2	2	6
2	2	0	2	6
1	2	1	2	5
2	2	2	0	7
2	2	2	0	6
2	0	0	0	7

2	1	2	0	7
0	0	1	2	6
1	2	2	1	6
2	2	2	2	7
1	2	2	2	6
2	2	2	2	5
2	2	2	2	5
0	0	0	0	3
2	0	2	2	7

0	2	0	2	4
2	2	2	2	0
2	0	2	2	6
0	0	0	0	7
0	0	0	0	0
1	2	2	0	3
0	2	0	0	2
2	0	0	0	6
2	2	2	2	6

2	0	0	0	6
1	2	1	1	2
2	2	2	2	2
2	2	2	2	5
1	2	0	0	3
1	2	2	2	4
2	0	2	2	6
2	2	2	2	6
2	2	2	0	2

0	2	0	0	1
0	0	0	0	0
2	0	0	0	6
1	2	2	2	6
0	0	0	0	0
0	0	0	0	4
0	2	0	1	6
1	2	2	2	5
0	0	0	2	0

2	2	0	2	7
2	0	2	0	5
2	1	2	2	7
0	2	0	2	3
1	0	1	2	4
0	2	2	2	0
1	0	1	2	3
2	2	2	2	6
0	0	0	0	0

0	0	0	0	0
0	2	2	0	4
2	2	0	0	6
2	1	2	2	7
2	0	0	0	6
1	2	2	2	5
0	0	0	0	0
1	0	0	0	4
0	2	0	1	5

0	0	0	0	0
0	0	0	0	0
2	0	0	2	6
2	2	0	2	6
1	1	0	1	5
0	0	2	2	4
1	2	2	2	4
0	0	0	0	4
1	2	2	2	5

1	2	0	0	4
1	0	0	2	5
0	0	0	0	0
2	0	2	2	6
0	1	0	1	6
1	2	1	2	6
1	0	2	2	5
1	1	2	1	6
2	1	2	0	6

0	1	1	2	5
0	2	2	2	5
0	2	2	2	6
1	0	2	2	5
2	2	2	2	6
0	1	0	2	0
2	2	0	2	2
2	1	2	2	6
0	2	0	1	2

0	2	2	2	6
2	1	0	2	6
2	1	2	2	6
0	0	0	0	5
0	0	0	2	4
2	0	2	2	7
0	0	2	2	2
2	2	2	2	4
0	0	0	0	0

2	0	2	2	5
1	0	0	0	4
0	0	0	0	4
0	0	0	2	1
2	1	1	0	6
1	2	2	2	5
0	2	0	0	1
2	2	1	2	5
1	0	2	0	3

2	0	0	2	6
0	0	0	2	3
0	0	2	1	0
2	2	2	2	6
2	0	2	0	6
2	2	2	0	5
2	2	2	2	6
2	2	2	2	2
0	0	0	0	0

2	2	2	2	7
2	0	0	2	6
2	2	1	2	7
2	2	2	2	7
0	2	0	0	7
2	2	2	0	6
1	2	1	0	5
2	0	0	0	4
2	2	2	0	0

2	1	0	0	6
2	0	2	1	7
0	2	0	1	0
0	0	0	0	0
0	0	0	0	0
0	0	0	0	0
2	2	0	0	5
0	2	2	0	6
2	2	0	0	3

0	0	0	1	7
1	2	0	0	5
0	2	0	0	4
0	2	1	0	4
1	2	2	2	6
2	0	1	0	2
2	0	2	2	6
2	0	2	2	7
2	0	2	2	7

2	0	2	0	6
2	1	1	2	7
2	0	2	0	7
2	1	2	2	7
0	0	2	2	0
1	0	1	1	4
2	2	2	0	7
2	2	2	2	6
2	2	2	0	5

2	2	2	2	6
2	2	2	2	6
2	0	0	2	4
2	0	2	2	7
2	0	0	0	5
2	2	2	2	6
2	0	0	0	6
0	2	0	0	1
0	2	0	2	3

0	2	0	2	6
0	2	0	0	6
0	0	0	2	0
0	1	0	0	4
0	0	0	0	7
0	0	0	2	5
0	0	0	1	5
0	0	0	0	5
0	2	0	0	4

0	1	0	0	7
0	1	0	1	6
0	2	0	1	5
0	2	0	2	4
0	2	0	2	0
0	2	0	2	6
0	1	0	2	6
0	0	0	0	7
0	0	0	0	0

0	0	0	0	0
0	0	2	0	0
0	0	2	2	5
1	2	2	2	6
2	2	2	0	4
0	2	2	0	4
1	2	2	2	0
2	0	0	0	6
0	0	0	0	0

0	0	2	2	5
0	0	0	0	2
0	0	0	0	0
2	2	2	2	4
2	0	2	2	7
2	2	2	2	6
2	2	0	2	7
2	2	2	2	6
2	2	2	2	5

2	0	2	2	5
2	0	0	0	6
0	0	0	0	0
2	0	2	2	5
2	0	0	0	4
0	0	0	0	0
0	0	0	0	0
0	0	0	0	0
0	0	0	0	0

0	0	0	0	0
0	0	0	0	0

ptgi_6	ptgi_9	ptgi_12	oq45_sint1	oq45_ri1
5	3	4	53	21
2	2	2	61	17
0	6	6	35	15
6	7	3	64	11
7	6	6	54	18
1	4	0	47	28
3	4	1	0	20
4	5	3	62	20

4	4	3	58	21
0	0	0	55	15
1	6	6	55	26
5	7	6	0	17
6	7	6	44	0
7	7	3	0	10
5	5	4	53	17
7	6	2	57	22
5	5	2	55	23

0	0	0	57	23
5	6	3	78	28
6	7	3	37	7
4	0	5	73	31
7	6	6	47	14
0	6	1	59	26
0	0	0	63	20
7	7	6	15	4
4	7	3	40	0

7	7	6	63	22
6	6	0	71	15
6	6	6	46	13
3	6	6	67	22
5	5	5	55	23
7	6	6	44	10
7	6	3	42	9
5	6	4	70	30
0	0	0	52	19

6	6	4	46	7
4	5	4	39	16
6	5	3	61	19
6	7	6	44	13
7	4	3	35	9
7	7	6	35	10
6	6	3	45	0
6	6	0	0	0
5	4	3	64	0

0	7	0	69	0
6	6	3	58	0
0	0	0	74	30
7	2	4	0	21
1	2	7	63	22
6	7	6	71	29
0	0	0	59	31
3	2	2	54	18
3	2	2	61	12

5	6	3	63	25
4	1	2	71	21
6	7	6	35	10
5	0	3	0	5
0	0	0	71	18
0	0	0	54	13
0	0	0	37	18
5	6	7	37	0
0	0	0	83	28

0	0	0	0	20
7	5	3	30	14
6	5	1	49	18
2	3	7	35	17
6	7	6	40	14
4	3	4	79	19
0	6	0	48	10
0	0	0	76	26
0	0	0	36	11

0	7	0	55	25
0	0	0	52	0
0	0	0	37	0
4	2	5	32	16
0	0	0	52	30
0	0	0	92	30
6	0	0	80	22
7	1	6	35	11
0	0	3	66	22

7	6	6	66	22
6	7	3	57	17
0	0	0	0	27
6	6	3	35	11
2	1	5	73	9
0	0	0	0	13
0	0	0	36	0
0	0	0	75	27
0	0	0	83	22

7	0	0	48	0
0	0	0	70	20
5	5	4	61	21
3	0	6	59	26
5	4	1	75	22
5	0	7	43	16
5	0	1	73	11
5	3	4	47	9
0	0	4	78	18

6	7	3	53	13
0	0	0	88	24
0	0	0	0	17
3	5	4	46	14
1	4	6	69	18
5	4	1	89	23
0	0	0	0	0
5	5	3	45	0
1	3	5	90	40

3	4	0	29	5
6	7	3	21	7
2	6	6	50	12
3	4	3	76	7
3	4	5	49	0
6	5	3	61	25
7	0	6	40	10
5	4	4	74	21
1	0	0	58	15

0	5	4	61	20
5	6	3	38	7
6	5	2	0	25
2	4	4	75	15
1	2	5	45	18
5	6	3	47	20
6	6	6	66	22
6	0	3	48	13
3	3	3	34	7

6	7	6	68	15
6	6	6	0	24
5	1	1	65	30
2	0	0	74	32
7	4	6	46	3
2	4	4	74	28
4	5	4	47	15
7	6	6	63	5
7	0	6	28	2

6	5	4	40	9
0	0	0	50	16
0	0	0	55	15
6	6	6	0	21
5	5	4	72	16
5	5	3	61	15
7	7	6	26	5
7	6	6	48	10
5	6	6	0	0

6	6	3	64	14
7	6	6	0	14
0	0	0	60	22
6	7	4	41	12
5	5	1	38	0
5	5	4	57	20
6	6	3	0	25
0	6	0	67	17
7	7	6	86	24

6	6	3	58	21
5	0	0	46	15
0	7	3	44	16
3	5	7	38	10
5	0	0	60	14
0	6	6	48	7
7	6	6	55	10
0	0	0	31	2
0	0	0	87	33

3	0	5	81	24
0	0	0	45	24
6	3	3	50	20
0	0	0	49	27
0	0	0	46	15
7	5	6	30	19
4	4	2	55	16
5	5	3	68	27
5	0	6	52	22

1	6	3	39	11
7	0	6	26	8
2	0	0	57	17
6	7	6	69	21
0	0	1	34	10
3	4	4	50	11
7	6	4	53	9
3	2	4	60	18
6	4	4	51	26

0	0	0	75	27
0	0	0	50	18
4	2	4	0	21
0	0	0	0	25
0	0	0	0	14
0	0	0	41	13
7	7	6	38	8
0	0	0	84	23
7	7	6	41	9

0	7	0	59	21
1	0	6	72	13
0	6	1	49	10
6	0	0	62	23
6	6	3	36	17
0	6	4	59	10
6	6	3	54	12
0	4	1	84	12
3	0	0	72	18

2	1	2	94	26
6	6	3	51	19
5	5	2	0	20
5	5	4	0	17
6	1	3	0	18
6	5	3	34	16
2	5	6	24	11
5	5	4	57	12
0	0	0	36	0

7	7	6	29	5
6	6	4	30	16
0	0	0	0	22
7	0	5	6	10
0	0	0	71	28
5	4	3	57	16
3	4	1	74	28
0	7	3	73	20
6	5	4	58	16

6	7	6	34	17
6	6	3	16	14
5	5	1	0	25
7	7	6	75	25
0	7	0	47	18
2	5	4	0	0
6	7	1	44	0
3	0	4	72	28
4	0	6	79	26

6	6	1	53	10
5	5	4	48	24
6	7	4	0	18
7	6	6	0	20
6	1	4	59	15
6	6	3	62	19
0	0	0	66	17
1	0	6	88	29
7	6	6	55	10

6	7	3	64	16
5	0	1	43	13
0	0	0	47	31
6	6	3	54	24
6	0	4	68	18
6	5	3	0	23
6	6	4	49	15
6	6	4	57	21
5	0	0	64	0

6	6	4	51	22
7	7	6	42	8
1	6	3	0	24
1	6	6	76	26
7	4	3	50	27
6	7	6	51	13
7	6	3	30	7
3	0	7	57	26
7	7	6	44	9

6	6	3	42	13
7	6	6	38	8
0	6	4	23	4
0	0	0	54	14
0	0	0	44	17
1	5	2	47	13
0	0	0	68	20
6	2	4	72	0
7	7	3	46	8

0	0	0	42	15
1	1	2	58	30
3	6	6	64	26
7	7	6	80	17
2	2	0	37	25
6	5	4	41	17
5	7	3	55	9
6	6	3	80	8
2	6	0	70	0

1	0	0	0	12
0	0	0	0	28
0	0	0	38	0
7	7	6	29	14
0	0	0	0	12
0	0	0	0	26
2	0	3	36	21
5	5	1	0	25
0	0	0	49	11

6	6	6	69	17
0	7	6	58	22
7	6	6	46	8
3	3	6	41	16
0	4	4	36	12
6	6	6	64	4
0	3	1	68	16
3	3	1	78	22
0	0	0	1	2

0	0	0	77	38
7	7	0	73	15
7	0	0	59	21
6	6	6	66	21
0	0	0	45	20
6	6	3	39	8
0	0	0	87	26
5	0	0	53	15
5	3	1	50	18

0	0	0	38	19
0	0	0	57	17
5	0	3	69	14
7	0	6	74	18
5	0	7	47	20
5	6	3	57	11
6	6	3	44	18
0	0	0	64	17
5	6	3	59	17

4	5	0	58	31
0	0	4	49	14
0	0	0	50	11
6	7	6	45	7
0	0	3	41	7
5	5	3	73	14
5	5	3	51	18
6	6	3	36	7
7	5	4	34	11

6	5	4	70	4
5	6	3	60	25
5	7	6	59	10
6	6	3	36	14
7	6	6	28	18
6	0	3	87	15
0	0	7	87	34
7	7	3	32	8
0	0	1	74	26

6	6	6	60	18
5	1	4	37	15
6	7	3	43	23
6	6	3	0	21
5	0	4	47	22
7	0	3	54	15
7	7	3	52	27
4	6	6	69	30
0	0	0	57	16

6	7	6	63	25
0	0	0	47	9
4	0	0	65	18
6	0	6	0	26
1	0	0	74	28
3	5	3	83	27
1	2	7	57	21
6	4	3	87	25
5	6	4	61	20

7	7	6	0	22
4	5	1	42	9
7	7	6	25	11
2	6	3	59	15
5	6	0	69	14
2	7	6	81	0
6	5	6	48	26
7	7	6	73	12
0	0	0	75	14

5	5	3	0	14
0	0	6	40	11
1	2	3	64	19
7	7	6	80	0
0	0	0	0	10
6	4	0	16	16
6	4	0	11	2
0	0	0	80	33
6	5	0	53	24

6	0	0	76	22
7	6	4	45	8
6	4	3	60	11
0	0	0	65	23
0	0	0	29	12
0	0	0	36	18
4	0	5	38	17
6	7	2	74	21
1	5	0	53	36

4	2	6	62	18
6	0	0	68	25
0	0	0	63	19
5	1	1	76	25
6	6	3	80	23
4	2	0	71	0
6	6	3	43	0
0	7	6	10	0
0	6	6	87	19

0	7	0	65	28
7	1	6	62	32
0	7	0	74	28
7	7	6	43	20
0	6	1	63	26
0	1	3	72	29
5	7	4	37	18
1	6	6	62	16
5	7	1	55	31

7	7	6	0	0
7	7	6	24	15
0	0	4	79	29
7	7	6	42	27
0	0	0	0	14
6	6	3	41	14
7	0	0	65	22
1	1	4	93	25
2	4	3	0	19

5	6	1	22	12
6	2	3	32	4
7	7	6	48	20
5	3	7	78	26
0	0	0	55	19
6	7	6	30	9
5	5	1	64	27
0	0	0	65	26
5	6	2	71	8

6	7	7	57	19
7	7	2	79	19
0	5	4	0	1
0	4	4	70	26
0	7	6	57	20
0	5	6	37	10
0	7	6	47	14
0	3	0	56	0
0	0	0	56	20

0	0	0	64	18
0	6	3	69	29
6	7	6	7	5
5	4	1	81	20
3	3	0	53	19
6	6	0	65	17
5	6	3	41	18
0	0	0	63	23
0	0	0	60	20

0	6	3	77	28
0	0	0	75	22
0	0	0	19	4
7	6	3	76	20
0	7	6	59	25
1	6	7	66	7
7	5	6	77	3
7	7	6	42	26
6	6	3	38	8

7	7	6	30	20
0	0	0	63	24
0	0	0	28	4
7	4	3	57	19
7	0	0	82	13
0	0	0	73	23
0	0	0	36	17
0	0	0	66	19
0	0	0	0	0

0	0	0	0	0
0	0	0	82	0

oq45_sol1	oq45_tot1	oq45_sint2	oq45_ri2	oq45_sol2
21	0	52	19	14
12	0	73	22	16
8	0	0	0	0
16	0	54	9	11
9	0	17	12	0
7	0	55	29	8
7	0	58	19	9
7	0	58	15	10

19	0	56	20	13
5	0	0	0	0
15	0	69	23	17
17	0	49	21	10
10	0	21	6	4
14	0	5	5	4
7	0	48	18	3
2	0	19	13	3
14	0	43	16	11

19	0	0	0	0
20	0	46	17	9
3	0	39	11	10
7	0	62	24	8
12	0	21	9	7
19	0	0	0	0
8	0	0	0	0
0	0	11	7	4
7	0	48	23	10

9	0	23	13	4
12	0	46	21	7
12	0	18	9	13
19	0	59	24	13
14	0	43	15	5
5	0	20	8	4
11	0	27	8	5
6	0	27	18	5
14	0	0	0	0

11	0	21	9	8
14	0	59	29	17
9	0	17	12	15
15	0	32	11	7
3	0	31	15	8
9	0	7	4	7
11	0	24	6	5
12	0	14	8	5
15	0	42	7	8

20	0	0	0	0
16	0	13	7	3
23	0	0	0	0
10	0	23	9	7
19	0	63	27	18
17	0	20	7	7
21	0	0	0	0
19	0	68	26	14
16	0	52	13	16

19	0	48	10	9
18	0	74	18	12
7	0	43	8	6
8	0	26	6	8
22	0	0	0	0
19	0	0	0	0
9	0	0	0	0
1	0	35	16	9
15	0	0	0	0

18	0	0	0	0
6	0	41	17	6
0	0	48	15	17
6	0	71	24	12
6	0	27	16	2
23	0	74	19	22
10	0	0	0	0
19	0	0	0	0
9	0	0	0	0

15	0	0	0	0
10	0	0	0	0
6	0	0	0	0
15	0	55	19	21
26	0	0	0	0
26	0	0	0	0
22	0	31	5	6
10	0	26	12	14
0	0	0	0	0

13	0	16	8	5
17	0	64	16	14
14	0	0	0	0
7	0	24	4	5
14	0	68	15	12
13	0	0	0	0
2	0	0	0	0
18	0	0	0	0
16	0	0	0	0

23	0	16	7	4
6	0	0	0	0
16	0	48	17	22
4	0	34	21	11
18	0	63	16	13
6	0	28	20	9
21	0	60	7	12
9	0	45	13	10
22	0	0	0	0

11	0	15	9	7
16	0	0	0	0
10	0	0	0	0
0	0	42	7	11
9	0	74	31	24
22	0	47	21	13
4	0	0	0	0
10	0	42	15	7
20	0	70	33	25

2	0	32	14	8
9	0	13	9	5
19	0	41	14	6
16	0	58	15	12
12	0	32	13	5
20	0	22	26	9
10	0	8	10	6
9	0	58	18	8
14	0	79	21	13

26	0	0	0	0
13	0	50	15	17
16	0	50	23	11
18	0	73	19	14
2	0	62	24	20
8	0	54	22	11
10	0	20	15	5
2	0	52	11	3
6	0	42	17	10

17	0	9	5	3
20	0	12	13	11
19	0	61	25	21
20	0	65	30	26
5	0	28	7	9
10	0	59	23	12
2	0	34	18	4
18	0	35	16	6
4	0	21	6	6

7	0	30	17	4
8	0	0	0	0
12	0	0	0	0
6	0	34	13	8
25	0	55	22	22
19	0	60	13	14
6	0	9	7	5
16	0	10	5	4
9	0	19	7	4

20	0	14	5	2
16	0	12	4	2
17	0	0	0	0
6	0	7	7	5
4	0	47	27	19
21	0	68	24	20
14	0	14	8	4
23	0	0	0	0
23	0	9	19	7

19	0	56	21	11
10	0	12	10	6
23	0	0	0	0
20	0	47	22	21
9	0	47	10	11
3	0	0	0	0
9	0	19	8	5
6	0	0	0	0
19	0	0	0	0

25	0	61	24	14
17	0	0	0	0
17	0	32	7	10
15	0	0	0	0
17	0	0	0	0
11	0	17	15	11
5	0	44	15	6
16	0	26	17	18
21	0	49	22	5

17	0	60	18	6
2	0	8	5	7
19	0	51	19	20
4	0	48	17	15
13	0	0	0	0
6	0	48	12	12
17	0	25	14	4
21	0	52	24	22
24	0	28	16	15

12	0	0	0	0
15	0	0	0	0
15	0	63	27	4
18	0	0	0	0
9	0	0	0	0
14	0	0	0	0
9	0	24	7	4
20	0	0	0	0
24	0	7	12	14

13	0	0	0	0
6	0	51	12	21
7	0	0	0	0
12	0	54	20	13
10	0	31	15	6
5	0	0	0	0
7	0	16	4	7
23	0	0	0	0
23	0	63	23	16

25	0	75	20	15
12	0	29	9	9
16	0	64	18	16
19	0	42	17	7
7	0	27	12	9
16	0	14	7	11
1	0	57	23	13
22	0	58	14	18
8	0	0	0	0

0	0	6	8	5
14	0	15	8	11
23	0	0	0	0
1	0	10	9	4
10	0	0	0	0
10	0	34	16	11
18	0	73	33	18
23	0	0	0	0
3	0	28	17	6

7	0	11	11	4
3	0	20	16	6
1	0	71	25	19
0	0	8	3	5
2	0	0	0	0
3	0	52	15	13
11	0	29	14	8
16	0	62	23	14
12	0	72	20	16

11	0	35	12	7
12	0	47	27	11
12	0	17	7	3
13	0	33	12	4
19	0	52	10	14
21	0	33	17	12
11	0	0	0	0
10	0	64	30	17
10	0	19	7	8

5	0	49	13	7
4	0	39	14	9
14	0	0	0	0
23	0	39	20	14
21	0	51	15	8
19	0	14	7	3
14	0	13	7	11
0	0	3	13	9
15	0	9	13	10

15	0	8	4	9
15	0	6	5	11
19	0	40	24	12
20	0	47	19	13
23	0	20	6	6
22	0	12	7	4
9	0	10	5	4
16	0	70	19	17
9	0	18	16	6

19	0	15	7	19
14	0	23	9	13
11	0	0	0	0
14	0	0	0	0
12	0	0	0	0
18	0	51	14	16
11	0	0	0	0
7	0	58	16	14
16	0	15	10	10

9	0	0	0	0
21	0	71	29	23
23	0	51	16	16
31	0	9	5	4
9	0	52	23	19
7	0	29	11	7
13	0	38	17	9
4	0	49	13	3
27	0	28	8	5

16	0	68	18	19
16	0	0	0	0
12	0	0	0	0
10	0	22	11	3
0	0	0	0	0
15	0	0	0	0
5	0	48	11	8
10	0	55	12	18
7	0	0	0	0

14	0	44	12	11
15	0	0	0	0
6	0	22	13	4
4	0	37	9	8
5	0	0	0	0
13	0	41	11	9
19	0	0	0	0
16	0	47	18	9
2	0	0	0	0

24	0	0	0	0
17	0	23	13	4
17	0	8	5	4
22	0	26	8	8
14	0	0	0	0
8	0	39	10	9
22	0	0	0	0
17	0	36	19	9
13	0	45	17	19

6	0	0	0	0
20	0	0	0	0
16	0	57	14	12
21	0	34	13	3
2	0	17	11	14
10	0	48	18	13
11	0	36	10	7
20	0	0	0	0
20	0	48	23	14

19	0	61	30	9
9	0	0	0	0
5	0	0	0	0
7	0	24	9	6
6	0	0	0	0
10	0	60	16	8
0	0	44	11	4
7	0	37	9	7
7	0	20	12	10

6	0	40	14	11
3	0	45	8	6
15	0	51	13	12
19	0	11	6	6
9	0	3	6	4
22	0	37	10	10
18	0	0	0	0
10	0	18	9	11
19	0	0	0	0

5	0	18	10	9
8	0	31	17	9
8	0	20	17	8
6	0	21	18	9
6	0	20	16	3
10	0	25	9	6
8	0	26	25	4
12	0	51	28	18
17	0	0	0	0

17	0	34	14	10
14	0	0	0	0
0	0	59	24	10
8	0	55	26	8
12	0	66	25	14
12	0	74	28	16
19	0	57	22	18
13	0	45	19	5
13	0	28	25	14

13	0	1	8	12
12	0	58	20	20
3	0	26	14	8
4	0	49	26	16
14	0	29	17	10
12	0	39	11	5
14	0	40	17	15
24	0	22	6	7
22	0	0	0	0

6	0	48	16	6
9	0	0	0	0
8	0	50	14	7
16	0	12	8	7
8	0	0	0	0
4	0	28	10	4
9	0	17	7	14
14	0	0	0	0
30	0	39	25	21

23	0	18	8	10
2	0	23	6	7
17	0	38	11	10
18	0	0	0	0
4	0	0	0	0
11	0	0	0	0
14	0	38	19	11
17	0	36	9	11
20	0	56	25	21

9	0	54	20	9
18	0	25	12	6
8	0	0	0	0
19	0	71	28	22
29	0	40	14	8
23	0	58	27	22
9	0	20	12	4
0	0	0	0	0
18	0	0	0	0

6	0	0	0	0
29	0	18	7	6
29	0	0	0	0
16	0	12	8	10
14	0	0	0	0
21	0	0	0	0
9	0	49	16	3
14	0	76	22	18
20	0	51	18	13

0	0	12	8	3
6	0	7	13	9
28	0	0	0	0
16	0	18	12	5
11	0	0	0	0
10	0	20	13	5
17	0	11	4	10
20	0	68	30	18
12	0	47	19	12

1	0	37	11	7
15	0	29	5	14
16	0	6	5	7
26	0	33	12	12
11	0	0	0	0
9	0	9	8	8
19	0	51	8	11
19	0	0	0	0
12	0	57	17	9

16	0	38	10	6
19	0	39	12	12
6	0	0	0	0
24	0	0	0	0
14	0	0	0	0
6	0	0	0	0
10	0	0	0	0
12	0	0	0	0
12	0	0	0	0

21	0	0	0	0
18	0	0	0	0
4	0	10	11	5
9	0	74	21	14
11	0	52	24	23
10	0	26	12	9
2	0	45	22	12
19	0	0	0	0
13	0	0	0	0

18	0	0	0	0
14	0	0	0	0
4	0	0	0	0
18	0	7	11	10
22	0	0	0	0
28	0	44	9	10
27	0	18	6	18
14	0	13	13	7
9	0	14	5	10

4	0	12	7	4
13	0	0	0	0
2	0	0	0	0
11	0	8	7	4
15	0	28	16	11
20	0	0	0	0
22	0	0	0	0
15	0	0	0	0
13	0	0	0	0

0	0	0	0	0
15	0	0	0	0

oq45_total2	oq45_sin3	oq45_ri3	oq45_sol3	oq45_total3
85	42	6	0	48
111	56	23	7	86
0	60	17	9	86
74	15	10	0	25
29	68	15	16	99
92	0	0	0	0
86	63	26	14	103
83	65	16	4	85

89	20	14	8	42
0	18	8	6	32
109	58	12	18	88
80	38	14	10	62
31	64	25	16	105
14	75	31	18	124
69	18	23	4	45
35	36	16	6	58
70	75	7	11	93

0	0	0	0	0
72	19	11	7	37
60	16	19	2	37
94	16	12	5	33
37	87	26	21	134
0	33	14	6	53
0	0	0	0	0
22	8	2	2	12
81	35	13	3	51

40	43	14	1	58
74	0	0	0	0
40	30	14	0	44
96	47	21	8	76
63	52	17	6	75
32	25	14	9	48
40	21	10	3	34
50	14	5	7	26
0	0	0	0	0

38	10	3	4	17
105	42	6	6	54
44	42	9	10	61
50	24	7	8	39
54	17	6	4	27
18	21	6	0	27
35	51	10	5	66
27	22	11	4	37
57	5	3	3	11

0	69	27	25	121
23	64	19	14	97
0	0	0	0	0
39	31	4	11	46
108	30	8	0	38
34	50	17	16	83
0	0	0	0	0
108	42	13	11	66
81	12	4	0	16

67	61	21	8	90
104	6	0	1	7
57	88	29	21	138
40	59	14	15	88
0	0	0	0	0
0	0	0	0	0
0	0	0	0	0
60	22	10	2	34
0	0	0	0	0

0	0	0	0	0
64	42	13	11	66
80	43	14	13	70
107	24	6	0	30
45	17	9	0	26
115	9	19	3	31
0	0	0	0	0
0	0	0	0	0
0	0	0	0	0

0	0	0	0	0
0	0	0	0	0
0	0	0	0	0
95	64	25	21	110
0	0	0	0	0
0	0	0	0	0
42	0	0	0	0
52	73	31	17	121
0	68	22	7	97

29	41	17	10	68
94	0	0	0	0
0	0	0	0	0
33	20	16	7	43
95	34	4	0	38
0	0	0	0	0
0	0	0	0	0
0	0	0	0	0
0	0	0	0	0

27	0	0	0	0
0	0	0	0	0
87	20	13	8	41
66	0	0	0	0
92	0	0	0	0
57	0	0	0	0
79	0	0	0	0
68	6	0	0	6
0	39	9	7	55

31	54	21	14	89
0	0	0	0	0
0	0	0	0	0
60	29	11	5	45
129	43	14	11	68
81	5	9	6	20
0	0	0	0	0
64	7	4	10	21
128	77	32	20	129

54	0	0	0	0
27	15	9	5	29
61	29	8	8	45
85	31	12	0	43
50	6	14	3	23
57	20	10	3	33
24	30	8	8	46
84	52	15	14	81
113	0	0	0	0

0	7	4	2	13
82	7	4	5	16
84	73	37	22	132
106	75	25	14	114
106	30	10	5	45
87	10	1	8	19
40	33	22	9	64
66	76	37	17	130
69	58	22	7	87

17	5	10	7	22
36	37	12	9	58
107	44	22	6	72
121	0	0	0	0
44	2	0	3	5
94	14	8	8	30
56	52	20	19	91
57	59	24	20	103
33	3	8	7	18

51	26	15	2	43
0	0	0	0	0
0	0	0	0	0
55	36	10	4	50
99	47	14	13	74
87	87	18	21	126
21	25	12	8	45
19	22	9	7	38
30	70	26	12	108

21	9	4	5	18
18	10	2	3	15
0	16	8	7	31
19	26	2	5	33
93	54	26	13	93
112	46	8	10	64
26	6	9	9	24
0	0	0	0	0
35	37	13	5	55

88	2	5	5	12
28	0	0	0	0
0	15	1	7	23
90	49	11	3	63
68	0	0	0	0
0	43	13	19	75
32	61	18	14	93
0	0	0	0	0
0	0	0	0	0

99	6	4	0	10
0	0	0	0	0
49	35	18	13	66
0	0	0	0	0
0	0	0	0	0
43	65	16	14	95
65	21	0	6	27
61	37	16	8	61
76	37	11	1	49

84	2	8	4	14
20	11	8	2	21
90	0	0	0	0
80	9	8	4	21
0	59	24	20	103
72	4	2	0	6
43	29	24	7	60
98	15	2	5	22
59	26	15	6	47

0	0	0	0	0
0	0	0	0	0
94	11	0	1	12
0	0	0	0	0
0	0	0	0	0
0	0	0	0	0
35	37	13	10	60
0	0	0	0	0
33	57	26	16	99

0	0	0	0	0
84	14	4	2	20
0	43	11	9	63
87	0	0	0	0
52	51	11	4	66
0	32	23	9	64
27	46	20	9	75
0	19	8	7	34
102	0	0	0	0

110	45	14	10	69
47	18	10	6	34
98	16	6	3	25
66	46	8	4	58
48	7	0	6	13
32	19	9	4	32
93	43	18	4	65
90	26	7	2	35
0	0	0	0	0

19	4	8	4	16
34	18	10	8	36
0	0	0	0	0
23	68	14	14	96
0	0	0	0	0
61	0	4	7	11
124	36	13	4	53
0	68	17	11	96
51	58	12	8	78

26	23	4	0	27
42	41	16	3	60
115	23	22	10	55
16	22	14	0	36
0	0	0	0	0
80	54	22	14	90
51	27	5	7	39
99	37	17	11	65
108	10	18	1	29

54	33	22	7	62
85	39	13	6	58
27	10	2	0	12
49	71	24	20	115
76	6	12	0	18
62	29	22	8	59
0	0	0	0	0
111	52	20	9	81
34	52	20	9	81

69	19	16	4	39
62	14	0	2	16
0	0	0	0	0
73	61	18	5	84
74	50	12	12	74
24	18	0	2	20
31	8	0	8	16
25	16	7	0	23
32	0	0	0	0

21	8	16	0	24
22	20	6	2	28
76	37	22	8	67
79	22	10	10	42
32	38	10	8	56
23	17	22	4	43
19	22	10	3	35
106	29	5	10	44
40	60	11	18	89

41	10	2	3	15
45	47	7	16	70
0	52	16	10	78
0	0	0	0	0
0	0	0	0	0
81	16	8	4	28
0	0	0	0	0
88	48	11	10	69
35	27	4	12	43

0	0	0	0	0
123	37	10	8	55
83	24	12	8	44
18	8	12	4	24
94	0	0	0	0
47	0	12	4	16
64	80	27	27	134
65	10	5	3	18
41	0	0	0	0

105	0	0	0	0
0	0	0	0	0
0	0	0	0	0
36	26	14	5	45
0	0	0	0	0
0	0	0	0	0
67	53	22	14	89
85	4	4	4	12
0	0	0	0	0

67	10	11	0	21
0	0	0	0	0
39	8	0	4	12
54	7	3	7	17
0	17	16	6	39
61	4	4	4	12
0	3	4	0	7
74	11	13	7	31
0	0	0	0	0

0	0	0	0	0
40	0	0	0	0
17	58	8	9	75
42	61	18	16	95
0	0	0	0	0
58	46	0	8	54
0	0	0	0	0
64	0	0	0	0
81	59	26	16	101

0	0	0	0	0
0	0	0	0	0
83	29	10	5	44
50	23	6	0	29
42	18	2	2	22
79	24	10	3	37
53	26	19	7	52
0	0	0	0	0
85	10	10	3	23

100	0	0	0	0
0	57	22	13	92
0	0	0	0	0
39	13	12	4	29
0	55	17	15	87
84	28	7	11	46
59	7	11	12	30
53	3	8	8	19
42	20	9	8	37

65	14	17	9	40
59	42	12	9	63
76	1	0	0	1
23	38	15	10	63
13	85	8	12	105
57	32	18	8	58
0	22	12	3	37
38	2	2	6	10
0	8	8	8	24

37	45	17	6	68
57	5	9	7	21
45	0	0	0	0
48	18	6	4	28
39	40	18	10	68
40	23	11	5	39
55	19	7	4	30
97	14	6	0	20
0	0	0	0	0

58	8	6	0	14
0	0	0	0	0
93	0	0	0	0
89	33	16	15	64
105	0	0	0	0
118	8	0	0	8
97	0	0	4	4
69	21	0	5	26
67	29	8	6	43

21	37	8	10	55
98	31	6	7	44
48	24	4	4	32
91	62	20	16	98
56	0	0	0	0
55	21	7	6	34
72	6	4	4	14
35	20	12	6	38
0	0	0	0	0

70	45	7	12	64
0	11	9	4	24
71	32	10	5	47
27	56	16	4	76
0	0	0	0	0
42	0	0	0	0
38	0	0	0	0
0	0	0	0	0
85	0	0	0	0

36	0	0	0	0
36	21	4	5	30
59	13	2	3	18
0	0	0	0	0
0	0	0	0	0
0	0	0	0	0
68	23	6	8	37
56	19	5	4	28
102	0	0	0	0

83	59	26	13	98
43	0	0	0	0
0	0	0	0	0
121	20	5	0	25
62	19	6	2	27
107	0	0	0	0
36	29	8	2	39
0	76	23	22	121
0	64	26	18	108

0	0	0	0	0
31	54	11	23	88
0	0	0	0	0
30	16	7	2	25
0	1	0	0	1
0	12	3	6	21
68	20	2	0	22
116	36	15	6	57
82	21	9	7	37

23	63	26	18	107
29	36	23	9	68
0	11	14	10	35
35	37	18	18	73
0	0	0	0	0
38	45	25	9	79
25	0	0	0	0
116	43	20	12	75
78	53	16	15	84

55	8	4	6	18
48	25	5	4	34
18	1	0	0	1
57	1	0	0	1
0	0	0	0	0
25	27	5	6	38
70	30	12	7	49
0	0	0	0	0
83	10	7	3	20

54	64	19	20	103
63	79	38	28	145
0	65	14	11	90
0	3	3	2	8
0	4	0	4	8
0	74	19	10	103
0	18	15	0	33
0	0	0	0	0
0	0	0	0	0

0	0	0	0	0
0	81	27	24	132
26	0	0	0	0
109	36	16	15	67
99	0	0	0	0
47	0	0	0	0
79	22	5	6	33
0	0	0	0	0
0	0	0	0	0

0	10	14	3	27
0	0	0	0	0
0	0	0	0	0
28	30	2	7	39
0	70	17	16	103
63	83	25	30	138
42	64	28	20	112
33	4	0	4	8
29	26	9	11	46

23	2	8	0	10
0	0	0	0	0
0	0	0	0	0
19	16	6	3	25
55	0	0	0	0
0	0	0	0	0
0	0	0	0	0
0	0	0	0	0
0	0	0	0	0

0	0	0	0	0
0	0	0	0	0

cont_1_mes3	cont_2_mes9	cont_3_mes12	toma_med_mes0	toma_med_mes3
0	0	0	0	1
1	1	0	0	1
1	1	0	0	1
1	1	0	0	1
0	1	0	0	0
0	0	0	0	0
0	1	0	0	0
1	1	0	0	1

1	1	0	0	1
1	0	0	0	0
1	1	0	0	1
0	1	0	0	1
1	1	0	0	1
1	1	0	0	0
0	0	0	0	1
0	0	0	0	1
0	1	0	0	0

1	0	0	0	0
0	0	0	0	0
1	1	0	0	1
0	0	0	0	0
0	0	0	0	0
0	0	0	0	0
1	0	0	0	1
1	1	0	0	1
1	1	0	0	1

1	1	0	0	1
1	0	0	0	0
1	1	0	0	1
1	0	0	0	0
1	0	0	0	1
1	1	0	0	1
1	1	0	0	1
1	0	0	0	1
0	0	0	0	0

1	1	0	0	1
1	1	0	0	1
1	0	0	0	0
1	1	0	0	1
0	0	0	0	0
1	0	0	0	1
1	1	0	0	1
1	0	0	0	1
1	0	0	0	1

1	1	0	0	0
1	0	0	0	1
0	0	0	0	0
1	0	0	0	1
0	0	0	0	0
1	0	0	0	1
0	0	0	0	0
1	0	0	0	1
1	1	0	0	1

1	1	0	0	1
0	0	0	0	1
1	0	0	0	1
0	0	0	0	0
0	0	0	0	0
1	0	0	0	1
0	0	0	0	0
1	0	0	0	0
0	0	0	0	0

1	0	0	0	0
1	1	0	0	0
1	0	0	0	0
1	0	0	0	1
1	1	0	0	0
1	1	0	0	1
1	1	0	0	1
0	0	0	0	0
0	0	0	0	0

1	1	0	0	1
0	0	0	0	0
0	0	0	0	0
1	1	0	0	1
0	0	0	0	0
0	0	0	0	0
1	0	0	0	1
1	1	0	0	1
1	0	0	0	1

1	1	0	0	1
1	1	0	0	1
0	0	0	0	1
1	0	0	0	1
1	1	0	0	1
0	0	0	0	1
0	0	0	0	0
0	0	0	0	0
0	0	0	0	0

0	0	0	0	1
0	0	0	0	0
1	1	0	0	1
1	0	0	0	1
1	0	0	0	1
0	0	0	0	0
1	0	0	0	1
1	1	0	0	1
1	0	0	0	1

1	1	0	0	0
0	0	0	0	0
0	0	0	0	0
1	0	0	0	1
0	1	0	0	0
0	0	0	0	1
1	0	0	0	1
0	0	0	0	1
1	0	0	0	1

0	0	0	0	0
1	1	0	0	1
1	1	0	0	1
1	0	0	0	1
0	1	0	0	0
0	0	0	0	1
0	0	0	0	1
1	1	0	0	1
0	0	0	0	0

1	1	0	0	1
1	1	0	0	1
1	1	0	0	1
1	1	0	0	1
0	0	0	0	1
1	1	0	0	1
1	1	0	0	1
0	0	0	0	0
1	0	0	0	0

1	0	0	0	1
1	1	0	0	1
1	0	0	0	1
0	0	0	0	0
1	1	0	0	1
1	1	0	0	1
1	1	0	0	0
0	0	0	0	0
1	0	0	0	1

1	1	0	0	1
0	0	0	0	0
1	0	0	0	1
1	0	0	0	1
1	1	0	0	1
1	1	0	0	0
0	0	0	0	0
1	1	0	0	1
1	1	0	0	1

1	1	0	0	1
1	1	0	0	1
0	0	0	0	0
1	1	0	0	1
1	1	0	0	0
1	1	0	0	0
1	0	0	0	1
1	1	0	0	1
0	0	0	0	0

1	1	0	0	1
1	0	0	0	1
1	1	0	0	1
1	1	0	0	1
0	0	0	0	1
1	1	0	0	1
1	0	0	0	1
0	0	0	0	0
0	0	0	0	0

0	0	0	0	0
0	0	0	0	0
1	0	0	0	1
0	0	0	0	0
1	0	0	0	1
1	1	0	0	1
1	1	0	0	1
1	0	0	0	1
0	0	0	0	0

1	0	0	0	0
0	0	0	0	0
1	0	0	0	0
1	0	0	0	1
1	0	0	0	1
0	0	0	0	0
0	0	0	0	0
1	1	0	0	1
0	0	0	0	0

0	0	0	0	0
0	0	0	0	0
1	1	0	0	1
0	0	0	0	0
0	0	0	0	0
0	0	0	0	0
1	0	0	0	1
0	0	0	0	0
1	1	0	0	1

0	0	0	0	0
1	0	0	0	0
1	0	0	0	0
1	0	0	0	1
1	1	0	0	1
0	0	0	0	0
0	0	0	0	0
1	1	0	0	1
0	0	0	0	0

1	0	0	0	0
1	1	0	0	1
0	1	0	0	0
1	0	0	0	0
1	1	0	0	1
1	0	0	0	1
1	1	0	0	1
1	0	0	0	1
0	0	0	0	0

0	1	0	0	0
1	1	0	0	1
0	0	0	0	0
1	0	0	0	1
0	0	0	0	0
1	0	0	0	1
0	1	0	0	0
1	1	0	0	1
1	1	0	0	1

0	0	0	0	0
1	0	0	0	1
0	0	0	0	1
0	0	0	0	0
0	0	0	0	0
0	0	0	0	0
1	1	0	0	1
1	0	0	0	1
1	0	0	0	1

1	1	0	0	1
1	1	0	0	0
0	0	0	0	0
1	1	0	0	1
1	1	0	0	1
1	1	0	0	1
0	0	0	0	0
0	0	0	0	0
0	1	0	0	0

0	0	0	0	0
1	0	0	0	0
1	0	0	0	1
0	0	0	0	0
0	0	0	0	1
1	0	0	0	1
1	0	0	0	1
1	0	0	0	1
1	0	0	0	1

1	1	0	0	1
0	0	0	0	0
1	1	0	0	1
1	0	0	0	1
1	0	0	0	1
1	1	0	0	1
0	0	0	0	1
1	0	0	0	1
0	1	0	0	0

1	0	0	0	1
1	1	0	0	1
0	0	0	0	0
1	0	0	0	1
0	0	0	0	0
1	1	0	0	1
1	0	0	0	0
1	1	0	0	1
0	0	0	0	1

0	0	0	0	1
0	1	0	0	0
1	1	0	0	1
0	0	0	0	0
1	0	0	0	1
1	1	0	0	1
1	1	0	0	1
1	1	0	0	1
1	1	0	0	1

1	0	0	0	1
0	0	0	0	0
0	0	0	0	0
1	1	0	0	1
0	0	0	0	0
0	0	0	0	0
1	0	0	0	1
0	1	0	0	1
0	0	0	0	0

1	1	0	0	1
1	1	0	0	1
1	1	0	0	1
1	1	0	0	1
1	0	0	0	1
0	1	0	0	0
1	1	0	0	1
1	1	0	0	1
0	0	0	0	0

0	0	0	0	0
0	1	0	0	1
0	0	0	0	0
0	0	0	0	0
0	0	0	0	1
1	0	0	0	1
0	0	0	0	0
1	0	0	0	0
1	1	0	0	1

0	0	0	0	0
0	0	0	0	0
0	0	0	0	0
1	0	0	0	1
0	0	0	0	0
1	1	0	0	1
1	1	0	0	1
1	0	0	0	0
1	1	0	0	1

0	0	0	0	0
0	0	0	0	1
0	0	0	0	0
0	0	0	0	0
0	0	0	0	1
1	1	0	0	1
1	1	0	0	1
1	1	0	0	1
1	0	0	0	1

1	1	0	0	1
1	0	0	0	1
1	0	0	0	1
1	1	0	0	1
1	0	0	0	1
0	0	0	0	0
0	0	0	0	0
0	0	0	0	1
0	0	0	0	0

1	0	0	0	1
1	1	0	0	1
1	1	0	0	1
1	1	0	0	1
1	0	0	0	1
1	0	0	0	1
0	1	0	0	0
1	0	0	0	1
0	0	0	0	0

1	1	0	0	1
0	0	0	0	0
0	0	0	0	1
0	0	1	0	0
1	0	0	0	0
1	1	0	0	1
0	1	0	0	0
1	1	0	0	1
1	1	0	0	1

1	0	0	0	1
1	1	0	0	0
0	1	0	0	0
1	0	0	0	1
0	0	0	0	0
1	0	0	0	0
1	0	0	0	1
1	1	0	0	1
0	0	0	0	0

1	1	0	0	1
1	0	0	0	1
1	0	0	0	1
1	0	0	0	1
1	0	0	0	1
0	0	0	0	0
1	1	0	0	1
1	0	0	0	1
0	0	0	0	0

0	0	0	0	0
1	1	0	0	1
0	0	0	0	0
0	0	0	0	0
0	0	0	0	0
0	0	0	0	0
1	0	0	0	1
1	1	0	0	1
0	0	0	0	0

0	0	0	0	1
0	0	0	0	1
1	0	0	0	1
1	1	0	0	1
1	1	0	0	1
1	0	0	0	1
1	1	0	0	1
0	0	0	0	0
1	1	0	0	1

0	0	0	0	0
0	0	0	0	0
1	1	0	0	1
1	0	0	0	0
0	1	0	0	0
1	0	0	0	1
1	1	0	0	1
1	1	0	0	1
1	1	0	0	1

1	1	0	0	1
1	0	0	0	0
0	0	0	0	0
1	1	0	0	1
0	0	0	0	1
1	1	0	0	1
0	0	0	0	0
1	0	0	0	1
1	0	0	0	1

1	1	0	0	1
1	1	0	0	1
0	1	0	0	0
1	0	0	0	1
1	0	0	0	1
0	0	0	0	0
1	1	0	0	1
1	0	0	0	1
1	1	0	0	1

1	1	0	0	1
1	0	0	0	1
1	1	0	0	1
0	1	0	0	1
0	0	0	0	0
0	1	0	0	1
1	1	0	0	1
1	1	0	0	1
0	0	0	0	0

0	0	0	0	0
0	0	0	0	0
1	1	0	0	1
1	1	0	0	1
1	0	0	0	0
1	1	0	0	1
0	1	0	0	0
0	0	0	0	0
0	0	0	0	0

1	1	0	0	1
1	0	0	0	1
0	0	0	0	0
0	1	0	0	0
1	1	0	0	1
0	1	0	0	1
0	0	0	0	0
1	1	0	0	1
1	1	0	0	1

0	0	0	0	0
0	0	0	0	0
0	0	0	0	0
1	1	0	0	1
1	0	0	0	1
0	0	0	0	0
0	0	0	0	0
0	0	0	0	0
0	0	0	0	0

0	0	0	0	0
0	0	0	0	0

toma_med_mes9	adherencia_6_meses	adherencia_12_meses	tto_psicologico_12	taller_12
0	18	59	0	0
1	47	47	0	0
1	72	70	0	0
0	0	69	0	0
1	73	69	1	0
1	63	60	0	0
0	52	62	0	0
1	63	50	0	0

1	71	67	0	0
0	0	0	0	0
1	78	76	1	0
1	59	76	0	0
1	75	78	1	0
1	78	78	0	0
0	0	48	0	0
1	21	34	0	0
1	0	58	0	0

0	0	0	0	0
1	0	49	0	0
1	68	66	0	0
0	0	27	0	0
0	28	29	0	0
0	0	45	0	0
0	0	0	0	0
0	0	72	0	0
1	67	73	1	0

1	68	73	0	0
0	57	0	0	0
1	72	78	0	0
1	54	73	0	0
0	76	53	0	0
1	78	68	0	0
1	78	53	1	0
1	43	72	0	0
0	0	0	0	0

0	68	50	1	0
1	73	59	0	0
1	30	28	1	0
1	78	63	0	0
0	65	61	0	0
0	78	13	0	0
1	77	13	1	0
0	76	0	0	0
1	77	75	1	0

1	0	0	0	0
1	75	78	0	0
0	0	0	0	0
0	26	75	1	0
0	18	13	0	0
0	78	78	1	0
0	0	0	0	0
0	71	33	0	0
0	74	75	1	0

1	42	78	1	0
0	45	33	0	0
0	74	78	0	0
0	41	35	0	0
0	0	0	0	0
0	0	0	0	0
0	0	0	0	0
0	29	33	1	0
0	0	0	0	0

0	0	0	0	0
1	61	69	0	0
1	78	77	0	0
1	59	69	0	1
1	73	77	1	0
1	75	49	1	0
1	0	0	0	0
0	0	0	0	0
0	0	0	0	0

1	0	0	0	0
0	0	0	0	0
0	0	0	0	0
1	73	75	0	0
0	0	0	0	0
0	0	0	0	0
0	78	0	0	0
0	75	47	0	0
0	0	18	0	0

1	76	78	0	0
1	75	78	0	0
0	0	0	0	0
0	71	35	1	0
1	73	67	0	0
0	0	0	0	0
0	0	0	0	0
0	0	0	0	0
0	0	0	0	0

0	64	64	0	0
0	0	0	0	0
0	74	68	1	0
0	51	66	0	0
1	69	18	0	0
0	46	27	0	0
0	68	78	1	0
1	70	77	0	0
0	0	76	0	0

1	70	78	1	0
0	0	0	0	0
0	0	0	0	0
0	65	28	0	0
1	64	55	1	0
0	38	23	0	0
0	0	0	0	0
0	0	66	0	0
1	38	16	0	0

0	37	0	0	0
1	75	68	0	0
1	73	34	0	0
0	68	70	0	0
0	53	41	0	0
1	0	71	0	0
0	0	72	0	0
1	49	48	0	0
0	17	17	0	0

1	0	62	0	0
1	65	64	1	0
1	0	47	0	0
1	69	70	1	0
0	44	51	0	0
1	76	78	0	0
1	58	73	0	0
0	0	71	0	0
0	25	69	0	0

0	52	44	0	0
0	71	55	0	0
1	0	51	0	0
0	18	0	0	0
0	70	65	1	0
1	64	78	1	0
1	71	78	0	0
0	13	34	0	0
0	0	59	0	0

1	73	76	1	0
0	0	0	0	0
0	0	0	0	0
0	69	66	0	0
1	67	75	0	0
1	65	62	0	0
0	48	12	0	0
1	78	72	1	0
1	78	78	1	0

1	78	78	1	0
1	3	78	1	1
0	0	0	0	0
1	78	13	0	0
0	68	44	1	0
1	76	38	1	0
0	78	78	1	0
1	0	0	0	0
0	73	66	0	0

1	65	69	0	0
0	62	0	0	0
1	0	64	0	0
1	73	78	1	0
0	78	0	0	0
1	0	72	0	0
0	0	68	0	0
0	0	0	0	0
0	0	0	0	0

0	45	16	0	0
0	0	0	0	0
1	63	72	1	0
0	0	0	0	0
0	0	0	0	0
1	71	72	1	0
1	76	70	1	0
0	23	18	0	0
0	18	25	0	0

0	33	33	0	0
0	23	15	0	0
0	64	0	0	0
1	40	78	0	0
0	0	13	0	0
0	18	18	0	0
0	30	13	0	0
0	30	18	0	0
0	23	23	0	0

0	0	0	0	0
0	0	0	0	0
1	47	59	1	1
0	0	0	0	0
0	0	0	0	0
0	0	0	0	0
0	0	78	0	0
0	0	0	0	0
1	69	78	1	0

0	0	0	0	0
0	20	13	0	0
0	0	13	0	0
0	0	0	0	0
0	66	69	0	0
0	0	24	0	0
0	78	78	0	0
1	0	69	1	0
0	76	76	0	0

0	62	13	0	0
1	48	53	1	0
1	27	55	0	0
0	20	16	0	0
1	0	73	1	0
0	0	33	0	0
1	0	27	0	0
0	0	28	0	0
0	0	0	0	0

0	0	13	0	0
0	0	41	0	0
0	0	0	0	0
0	17	36	0	0
0	0	0	0	0
0	75	55	0	0
1	44	29	0	0
1	64	68	0	0
1	74	73	0	0

0	0	24	0	0
1	68	78	1	0
1	0	74	0	0
0	1	30	0	0
1	0	0	0	0
0	48	35	0	0
0	54	63	0	0
0	0	78	1	0
0	65	77	0	0

1	68	70	0	0
1	0	73	0	0
0	0	21	0	0
1	0	56	0	0
1	76	66	0	0
0	47	66	0	0
0	0	0	0	0
0	0	78	0	0
1	78	76	0	0

0	77	48	0	0
0	71	60	0	0
0	0	0	0	0
0	71	69	0	0
0	54	62	0	0
0	76	75	1	1
0	50	13	0	0
0	41	13	0	0
0	46	0	0	0

1	75	75	0	0
0	0	13	0	0
1	73	78	1	0
0	77	78	1	0
0	0	13	0	0
1	0	78	1	0
1	70	73	1	0
0	0	63	1	0
1	0	58	0	0

1	0	67	0	0
1	0	72	0	0
0	0	38	0	0
0	0	0	0	0
0	0	0	0	0
1	0	67	0	0
0	0	0	0	0
1	78	78	0	0
1	70	73	0	0

0	0	0	0	0
1	65	18	0	0
1	72	73	0	0
0	20	13	0	0
0	76	0	0	0
1	71	75	1	0
1	78	73	1	0
1	76	73	1	0
1	0	0	0	0

0	0	0	0	0
0	0	0	0	0
0	0	0	0	0
1	0	78	0	0
0	0	0	0	0
0	0	0	0	0
0	0	76	1	0
1	0	59	0	0
0	0	0	0	0

1	0	73	1	0
1	0	78	1	0
1	0	73	0	0
1	0	78	0	0
0	0	71	1	0
1	0	78	0	0
1	0	78	1	0
1	0	78	1	0
0	0	0	0	0

0	0	0	0	0
1	0	0	0	0
0	0	0	0	0
0	0	43	0	0
0	0	0	0	0
1	0	73	1	0
0	0	0	0	0
0	0	0	0	0
1	0	66	0	0

0	0	0	0	0
0	0	0	0	0
0	23	56	0	0
0	75	45	0	0
0	69	71	0	0
1	76	78	1	0
1	62	78	0	0
0	0	0	0	0
1	71	70	0	1

1	60	0	0	0
0	0	61	0	0
0	0	0	0	0
1	37	78	0	0
0	0	39	0	0
1	0	76	0	1
1	0	70	0	0
1	0	78	0	0
0	0	52	0	0

0	0	65	1	0
0	0	66	0	0
0	0	61	0	0
1	0	78	1	0
1	0	78	0	0
0	0	59	0	0
0	0	59	0	0
0	0	49	0	0
0	0	44	0	0

0	0	69	0	0
1	0	60	0	0
1	0	37	0	0
1	0	65	0	0
0	0	60	0	0
0	0	74	0	0
0	0	66	0	0
1	0	26	0	0
0	0	0	0	0

1	0	52	0	0
0	0	0	0	0
0	0	0	0	0
0	1	26	0	0
0	0	0	0	0
1	0	77	0	0
1	0	48	0	0
0	0	65	0	0
1	0	24	0	0

0	0	16	0	0
0	0	35	0	0
1	0	56	0	0
0	0	15	0	0
0	72	0	0	0
0	70	13	0	0
0	70	23	0	0
1	75	73	1	0
0	0	0	0	0

1	77	78	1	0
0	0	76	0	0
0	0	48	1	0
0	0	13	0	0
0	0	0	0	0
0	0	0	0	0
0	0	0	0	0
0	0	0	0	0
0	0	0	0	0

0	0	0	0	0
1	0	66	1	0
0	0	41	1	0
0	0	0	0	0
0	0	0	0	0
0	0	0	0	0
0	0	70	0	0
1	0	65	1	0
1	0	0	0	0

0	0	11	0	0
0	0	0	0	0
0	0	0	0	0
1	0	64	1	0
0	0	73	0	0
1	0	0	0	0
1	0	78	0	1
0	0	15	0	0
1	0	78	0	0

0	0	0	0	0
0	0	13	0	0
1	0	0	0	0
0	0	48	0	0
0	0	73	0	0
1	0	78	0	0
1	0	13	0	0
0	2	78	0	0
1	6	77	1	0

1	0	78	0	0
0	0	28	0	0
0	0	39	0	0
1	0	68	0	0
0	0	0	0	0
1	0	75	0	0
0	0	0	0	0
0	0	71	0	0
0	0	14	0	0

1	0	76	0	0
1	0	77	1	0
1	0	64	1	0
0	0	28	0	0
0	0	0	0	0
0	0	15	0	0
1	0	74	0	0
0	0	0	0	0
1	0	58	0	0

1	0	73	0	0
1	0	16	0	0
1	0	73	0	0
1	0	73	0	0
0	0	13	0	0
1	0	78	0	0
1	0	75	0	0
1	0	0	0	0
0	0	0	0	0

0	0	0	0	0
0	0	59	0	0
0	0	75	0	0
1	0	75	1	0
0	0	0	0	0
1	0	0	0	0
1	0	75	1	0
0	0	0	0	0
0	0	0	0	0

1	0	73	0	0
0	0	0	0	0
0	0	0	0	0
0	23	18	0	0
1	0	73	0	0
1	0	78	0	0
0	23	13	0	0
0	78	73	0	0
1	78	73	1	0

0	20	23	0	0
0	0	0	0	0
0	0	0	0	0
1	78	75	0	0
0	30	0	0	0
0	0	0	0	0
0	0	0	0	0
0	0	0	0	0
0	0	0	0	0

0	0	0	0	0
0	0	0	0	0

alta_12	abandono_12	otro_estado_12	BASE_OCTUBRE_2015	NºFicha
0	0	0		1
0	0	0		2
0	0	0		3
1	0	0		4
0	0	0		5
0	0	0		6
0	0	0		7
0	0	0		8

0	0	0	9
0	1	0	10
0	0	0	11
0	1	1	12
0	0	0	13
0	0	0	14
0	0	1	15
0	1	0	16
0	0	0	17

0	0	0	18
0	0	0	19
0	0	0	20
0	0	0	21
0	1	0	22
0	0	0	23
0	0	0	24
1	0	0	25
0	0	0	26

0	0	0	27
0	0	0	28
0	0	0	29
0	0	0	30
0	0	1	31
1	0	0	32
0	0	1	33
0	1	0	34
0	0	0	35

0	0	0	36
0	0	0	37
1	0	1	38
1	0	0	39
0	0	1	40
0	1	0	41
0	0	1	42
0	1	0	43
0	0	1	44

0	0	1	45
0	0	1	46
0	0	0	47
0	0	1	48
0	1	0	49
0	0	0	50
0	0	0	51
0	1	0	52
0	0	0	53

0	0	0	54
0	1	0	55
0	0	0	56
0	1	0	57
0	0	0	58
0	0	0	59
0	0	0	60
0	0	0	61
0	0	0	62

0	0	0	63
0	0	0	64
1	0	0	65
0	0	1	66
0	0	0	67
0	0	0	68
0	0	0	69
0	0	0	70
0	0	0	71

0	0	0	72
0	0	0	73
0	0	0	74
0	0	0	75
0	0	0	76
0	0	0	77
0	0	0	78
0	0	0	79
0	1	0	80

1	0	0	81
0	0	0	82
0	0	0	83
0	0	0	84
0	0	0	85
0	0	0	86
0	0	0	87
0	0	0	88
0	0	0	89

0	0	0	90
0	0	0	91
0	0	0	92
0	0	0	93
0	1	0	94
0	0	0	95
0	0	0	96
0	0	0	97
0	0	0	98

0	0	0	99
0	0	0	100
0	0	0	101
0	1	0	102
0	0	0	103
0	1	0	104
0	0	0	105
0	0	0	106
0	1	0	107

0	0	0	108
0	1	1	109
0	0	0	110
0	0	0	111
0	0	0	112
0	0	0	113
0	0	0	114
0	0	0	115
0	0	0	116

0	0	0	117
0	0	0	118
0	0	0	119
0	0	0	120
0	1	0	121
0	0	0	122
0	0	0	123
0	0	0	124
0	0	1	125

1	0	0	126
0	1	0	127
0	0	0	128
0	0	0	129
0	0	0	130
0	0	0	131
0	0	0	132
0	1	0	133
1	0	0	134

0	0	0	135
0	0	0	136
0	0	0	137
0	0	1	138
0	0	0	139
0	0	0	140
0	1	1	141
0	0	0	142
0	0	0	143

0	0	0	144
0	0	0	145
0	1	0	146
0	1	0	147
0	0	1	148
0	0	0	149
0	0	0	150
0	0	0	151
0	1	0	152

0	0	0	153
0	0	0	154
0	0	0	155
0	0	0	156
0	0	0	157
0	0	0	158
0	0	0	159
0	0	0	160
0	0	0	161

0	1	0	162
0	0	0	163
0	0	0	164
0	0	0	165
0	0	0	166
0	0	0	167
0	0	0	168
0	0	1	169
0	0	1	170

0	0	0	171
0	1	0	172
0	0	0	173
1	0	0	174
0	1	0	175
0	1	0	176
0	1	0	177
0	0	0	178
0	0	1	179

0	0	0	181
0	0	0	183
0	0	0	184
0	0	0	185
0	0	0	186
0	0	0	187
1	0	0	188
0	0	0	189
1	0	0	190

0	0	0	191
0	1	0	192
0	1	0	193
0	0	0	194
0	1	0	195
0	1	0	196
1	0	0	197
0	0	0	198
0	0	0	199

0	1	0	200
0	0	0	201
0	1	0	202
0	0	0	203
0	0	0	204
0	1	0	205
0	1	0	206
0	1	0	207
0	0	0	208

1	0	0	209
0	1	0	210
0	0	0	211
0	1	0	212
0	0	0	213
0	1	1	214
0	0	0	215
0	1	0	216
0	0	0	217

0	1	0	218
0	0	0	219
0	0	0	220
1	0	0	221
0	0	0	222
0	0	0	223
0	0	0	224
0	0	0	225
0	0	0	226

0	0	0	227
0	0	0	228
0	0	0	229
0	0	0	230
0	0	0	231
1	0	0	232
0	0	0	233
0	0	0	234
1	0	0	235

1	0	0	236
0	0	0	237
0	0	0	238
0	0	1	239
0	1	0	240
0	0	0	241
0	1	0	242
0	1	0	243
0	0	0	244

0	0	1	245
0	1	0	246
0	0	0	247
0	0	0	248
0	1	1	249
0	0	1	250
0	0	0	251
0	0	0	252
0	0	0	253

0	0	1	254
0	0	0	255
0	0	0	256
0	0	0	257
0	0	0	258
0	0	1	259
0	0	0	260
0	0	0	261
0	0	0	262

0	0	0	263
0	1	0	264
0	0	0	265
0	1	0	266
0	0	0	267
0	0	0	268
0	0	0	269
0	0	0	270
0	0	0	271

0	0	0	272
0	0	0	273
0	0	0	274
0	1	0	275
0	0	0	276
0	0	0	277
0	0	0	278
0	0	1	279
0	0	0	280

0	0	0	281
0	0	0	282
0	0	0	283
0	0	0	284
0	0	0	285
0	0	0	286
0	0	0	287
0	0	0	288
0	0	0	289

0	0	0	290
0	0	0	291
0	0	1	292
0	1	0	293
0	0	0	294
0	0	0	295
0	0	0	296
0	0	0	297
0	0	0	298

0	0	0	299
0	0	0	300
1	0	0	301
0	1	0	302
0	0	0	303
0	0	0	304
0	0	0	305
0	0	0	306
0	0	0	307

0	0	0	308
0	0	1	309
0	0	0	310
1	0	1	311
0	0	0	312
0	0	0	313
0	0	0	314
1	0	0	315
1	0	0	316

0	0	0	317
0	0	1	318
0	1	0	319
0	0	0	320
1	0	0	321
1	0	0	322
0	0	0	323
1	0	0	324
0	1	0	325

0	1	0	326
0	0	0	331
0	0	1	332
1	0	0	333
0	0	0	334
0	0	1	335
0	0	0	336
1	0	0	337
0	0	0	338

0	0	1	339
0	0	0	340
0	0	0	341
0	1	0	342
0	0	0	343
0	0	0	344
0	1	0	345
0	0	0	346
0	1	0	347

0	1	0	348
0	0	1	349
0	0	0	350
0	0	0	351
0	0	0	352
0	1	0	353
0	1	0	354
0	0	0	355
0	0	0	356

0	0	0	357
0	0	0	358
0	0	0	359
0	1	0	360
0	0	0	361
0	0	0	371
0	0	0	372
0	0	0	373
0	0	0	374

0	0	0	375
0	0	0	376
0	0	1	377
0	0	0	378
0	0	0	379
0	0	0	380
0	0	0	381
0	0	0	382
0	0	0	383

0	0	0	384
0	0	0	385
0	0	0	386
0	0	0	387
0	0	0	391
0	0	0	392
0	0	0	393
0	1	0	394
0	0	0	395

0	0	0	396
0	1	0	397
0	0	0	398
0	1	0	399
0	0	0	400
0	0	1	401
0	1	0	402
0	0	1	403
0	0	1	404

0	0	0	410
0	0	1	411
0	1	0	412
0	0	0	413
0	0	0	414
1	0	0	415
0	0	0	416
0	1	0	422
0	1	0	423

0	0	0	424
0	0	0	425
0	0	0	426
0	1	0	427
0	0	0	428
0	1	0	429
0	0	0	430
0	0	0	431
0	0	0	432

0	0	0	433
0	1	0	434
0	0	0	435
0	0	0	436
0	1	0	437
0	0	0	438
1	0	0	439
0	0	0	440
0	0	0	461

0	0	0	462
0	1	0	463
0	0	0	464
0	0	0	465
0	0	0	466
0	0	0	467
0	0	0	468
0	0	0	469
0	0	0	470

0	0	0	471
0	0	0	472
0	0	0	473
0	1	0	474
0	0	0	475
0	0	0	476
0	0	0	477
0	0	0	478
0	0	0	479

0	1	0	480
0	0	0	501
0	0	0	502
0	0	0	561
0	0	0	562
0	0	0	600
0	0	0	700
0	0	0	701
0	0	0	702

0	0	0	704
0	0	0	705

VIF_SEIS_MESES	DAÑO_A_USTED	AMENZ_ALGUIEN_ QUE_QUIER	ATEMORIZAA	SIN_APOY_ECONO MM
1	0	0	0	0
0	0	0	0	0
0	0	0	0	0
0	0	0	0	0
0	0	0	0	0
0	0	0	0	0
0	0	0	0	0
0	0	0	0	0

2	0	0	0	0
3	1	0	1	0
0	0	0	0	0
0	0	0	0	0
0	0	0	0	0
0	0	0	0	0
0	0	0	0	0
4	0	0	0	1
4	1	0	0	0

2	0	0	0	0
0	0	0	0	0
0	0	0	0	0
8	1	0	0	0
5	0	0	1	0
0	0	0	0	0
2	0	0	0	0
0	0	0	0	0
0	0	0	0	0

0	0	0	0	0
5	1	1	0	0
0	0	0	0	0
0	0	0	0	0
0	0	0	0	0
1	0	0	0	0
0	0	0	0	0
0	0	0	0	0
0	0	0	0	0

0	0	0	0	0
0	0	0	0	0
0	0	0	0	0
0	0	0	0	0
0	0	0	0	0
0	0	0	0	0
0	0	0	0	0
0	0	0	0	0
0	0	0	0	0

0	0	0	0	0
0	0	0	0	0
0	0	0	0	0
3	0	0	0	0
6	1	1	1	0
0	0	0	0	0
3	0	0	0	0
5	0	0	1	0
0	0	0	0	0

0	0	0	0	0
0	0	0	0	0
0	0	0	0	0
0	0	0	0	0
0	0	0	0	0
0	0	0	0	0
1	0	0	0	0
0	0	0	0	0
2	0	0	0	0

1	0	0	0	0
3	0	0	0	0
0	0	0	0	0
0	0	0	0	0
0	0	0	0	0
0	0	0	0	0
2	0	0	0	0
0	0	0	0	0
0	0	0	0	0

1	0	0	1	0
0	0	0	0	0
0	0	0	0	0
7	1	1	0	0
2	0	0	0	0
0	0	0	0	0
0	0	0	0	0
0	0	0	0	0
5	0	0	1	0

1	0	0	0	0
3	0	0	0	0
0	0	0	0	0
0	0	0	0	0
0	0	0	0	0
0	0	0	0	0
5	0	0	0	1
0	0	0	0	0
8	1	0	1	1

0	0	0	0	0
0	0	0	0	0
3	0	0	0	0
0	0	0	0	0
1	0	0	0	0
2	0	0	0	0
0	0	0	0	0
0	0	0	0	0
0	0	0	0	0

2	0	0	0	0
2	0	0	0	0
0	0	0	0	0
1	0	0	0	0
0	0	0	0	0
0	0	0	0	0
0	0	0	0	0
0	0	0	0	0
0	0	0	0	0

8	1	1	1	1
0	0	0	0	0
1	0	0	0	0
0	0	0	0	0
0	0	0	0	0
6	1	0	1	1
0	0	0	0	0
0	0	0	0	0
4	0	0	1	0

0	0	0	0	0
0	0	0	0	0
3	0	0	0	0
0	0	0	0	0
3	0	0	0	0
0	0	0	0	0
3	0	0	0	0
0	0	0	0	0
0	0	0	0	0

0	0	0	0	0
7	1	0	1	0
5	0	1	0	0
6	1	1	0	1
0	0	0	0	0
0	0	0	0	0
0	0	0	0	0
0	0	0	0	0
0	0	0	0	0

0	0	0	0	0
5	1	0	0	0
6	0	0	1	0
4	0	1	0	0
0	0	0	0	0
0	0	0	0	0
0	0	0	0	0
0	0	0	0	0
0	0	0	0	0

0	0	0	0	0
0	0	0	0	0
0	0	0	0	0
1	0	0	0	0
0	0	0	0	0
3	0	0	0	0
3	0	0	0	0
0	0	0	0	0
0	0	0	0	0

0	0	0	0	0
3	0	0	0	0
1	0	0	0	1
1	0	0	0	0
0	0	0	0	0
0	0	0	0	0
0	0	0	0	0
0	0	0	0	0
0	0	0	0	0

2	0	0	0	0
0	0	0	0	0
3	0	0	0	1
5	0	0	1	1
6	1	0	1	0
0	0	0	0	0
0	0	0	0	0
1	0	0	0	1
0	0	0	0	0

1	0	0	0	0
0	0	0	0	0
2	0	0	1	0
0	0	0	0	0
0	0	0	0	0
1	0	0	0	0
1	0	0	0	0
2	0	0	0	0
0	0	0	0	0

3	0	0	0	0
0	0	0	0	0
0	0	0	0	0
1	0	0	1	0
5	1	0	1	0
0	0	0	0	0
0	0	0	0	0
0	0	0	0	0
4	0	0	1	0

3	0	0	1	1
1	0	0	0	0
0	0	0	0	0
0	0	0	0	0
0	0	0	0	0
0	0	0	0	0
0	0	0	0	0
0	0	0	0	0
1	0	0	0	0

4	0	0	1	0
0	0	0	0	0
9	1	0	1	0
4	0	0	1	0
1	0	0	0	0
1	0	0	0	0
7	1	1	1	0
0	0	0	0	0
5	0	1	0	1

0	0	0	0	0
0	0	0	0	0
6	1	1	1	0
0	0	0	0	0
6	1	0	0	1
3	0	0	0	0
0	0	0	0	0
0	0	0	0	0
3	0	0	0	0

4	0	1	0	0
0	0	0	0	0
0	0	0	0	0
4	1	0	0	0
8	0	0	0	1
0	0	0	0	0
0	0	0	0	0
4	1	0	0	0
0	0	0	0	0

0	0	0	0	0
6	1	1	1	0
0	0	0	0	0
4	0	0	0	0
0	0	0	0	0
4	0	0	1	0
1	0	0	0	0
2	0	0	0	1
3	0	0	1	0

6	1	0	1	0
7	1	0	1	0
3	0	0	1	0
1	0	0	1	0
1	0	0	1	0
3	0	0	0	0
0	0	0	0	0
0	0	0	0	0
0	0	0	0	0

0	0	0	0	0
0	0	0	0	0
0	0	0	0	0
6	1	1	1	0
0	0	0	0	0
4	0	0	0	1
0	0	0	0	0
0	0	0	0	0
2	0	0	0	0

0	0	0	0	0
0	0	0	0	0
0	0	0	0	0
2	0	0	0	0
2	0	0	1	0
1	0	0	0	0
0	0	0	0	0
0	0	0	0	0
0	0	0	0	0

7	1	1	0	0
0	0	0	0	0
0	0	0	0	0
1	0	0	0	0
0	0	0	0	0
1	0	0	0	0
0	0	0	0	0
0	0	0	0	0
0	0	0	0	0

1	0	0	0	0
10	1	1	1	1
0	0	0	0	0
0	0	0	0	0
0	0	0	0	0
0	0	0	0	0
0	0	0	0	0
0	0	0	0	0
0	0	0	0	0

0	0	0	0	0
0	0	0	0	0
0	0	0	0	0
0	0	0	0	0
2	0	0	0	0
0	0	0	0	0
0	0	0	0	0
0	0	0	0	0
0	0	0	0	0

5	1	0	0	1
0	0	0	0	0
3	0	0	0	0
1	0	0	0	0
1	0	0	0	0
0	0	0	0	0
3	0	0	0	0
0	0	0	0	0
1	0	0	0	0

1	0	0	0	0
4	1	0	0	0
1	0	0	1	0
0	0	0	0	0
3	0	0	1	0
0	0	0	0	0
0	0	0	0	0
6	1	1	0	1
5	0	0	1	0

0	0	0	0	0
0	0	0	0	0
4	0	0	1	0
3	0	0	0	0
0	0	0	0	0
0	0	0	0	0
0	0	0	0	0
0	0	0	0	0
0	0	0	0	0

0	0	0	0	0
0	0	0	0	0
0	0	0	0	0
3	0	0	0	0
0	0	0	0	0
8	1	0	1	1
5	0	0	0	1
0	0	0	0	0
0	0	0	0	0

0	0	0	0	0
0	0	0	0	0
0	0	0	0	0
5	1	1	0	0
3	0	0	0	0
3	0	0	0	0
6	1	0	1	1
0	0	0	0	0
0	0	0	0	0

3	0	0	0	0
0	0	0	0	0
6	1	0	1	0
0	0	0	0	0
7	1	1	1	1
4	0	0	0	0
0	0	0	0	0
12	1	1	1	1
0	0	0	0	0

4	0	0	0	0
0	0	0	0	0
0	0	0	0	0
0	0	0	0	0
0	0	0	0	0
0	0	0	0	0
5	1	0	0	1
0	0	0	0	0
0	0	0	0	0

0	0	0	0	0
0	0	0	0	0
0	0	0	0	0
0	0	0	0	0
0	0	0	0	0
0	0	0	0	0
0	0	0	0	0
0	0	0	0	0
0	0	0	0	0

0	0	0	0	0
0	0	0	0	0
0	0	0	0	0
5	0	0	1	1
0	0	0	0	0
6	0	0	1	0
0	0	0	0	0
0	0	0	0	0
0	0	0	0	0

0	0	0	0	0
2	0	0	0	0
0	0	0	0	0
0	0	0	0	0
0	0	0	0	0
0	0	0	0	0
0	0	0	0	0
0	0	0	0	0
2	0	0	0	0

4	0	0	0	0
2	0	0	0	0
0	0	0	0	0
0	0	0	0	0
0	0	0	0	0
5	1	0	0	0
0	0	0	0	0
2	0	0	0	0
0	0	0	0	0

0	0	0	0	0
0	0	0	0	0
0	0	0	0	0
11	1	1	1	1
0	0	0	0	0
0	0	0	0	0
11	1	1	1	0
3	0	0	0	0
3	0	0	0	0

0	0	0	0	0
0	0	0	0	0
0	0	0	0	0
5	0	0	1	0
6	0	0	1	0
0	0	0	0	0
0	0	0	0	0
0	0	0	0	0
0	0	0	0	0

0	0	0	0	0
0	0	0	0	0
2	0	0	0	0
0	0	0	0	0
0	0	0	0	0
0	0	0	0	0
0	0	0	0	0
0	0	0	0	0
0	0	0	0	0

2	0	0	0	0
2	0	0	1	0
0	0	0	0	0
0	0	0	0	0
0	0	0	0	0
5	0	0	1	0
4	1	0	0	0
4	0	1	0	0
5	0	0	1	0

0	0	0	0	0
0	0	0	0	0
0	0	0	0	0
0	0	0	0	0
4	0	0	1	0
0	0	0	0	0
0	0	0	0	0
0	0	0	0	0
0	0	0	0	0

0	0	0	0	0
0	0	0	0	0
1	0	0	0	0
0	0	0	0	0
0	0	0	0	0
0	0	0	0	0
4	0	0	0	1
0	0	0	0	0
0	0	0	0	0

0	0	0	0	0
0	0	0	0	0

BOFETEADAA	PATEADOO	PUÑETAZOOS	PALIZAA	FORZ_REL_SEXUALESS
0	0	0	0	0
0	0	0	0	0
0	0	0	0	0
0	0	0	0	0
0	0	0	0	0
0	0	0	0	0
0	0	0	0	0
0	0	0	0	0

0	0	0	0	0
0	0	0	0	0
0	0	0	0	0
0	0	0	0	0
0	0	0	0	0
0	0	0	0	0
0	0	0	0	0
0	0	0	0	0
0	0	0	0	0

0	0	0	0	0
0	0	0	0	0
0	0	0	0	0
1	1	1	1	0
1	0	0	0	1
0	0	0	0	0
0	0	0	0	0
0	0	0	0	0
0	0	0	0	0

0	0	0	0	0
0	0	0	0	0
0	0	0	0	0
0	0	0	0	0
0	0	0	0	0
0	0	0	0	0
0	0	0	0	0
0	0	0	0	0
0	0	0	0	0

0	0	0	0	0
0	0	0	0	0
0	0	0	0	0
0	0	0	0	0
0	0	0	0	0
0	0	0	0	0
0	0	0	0	0
0	0	0	0	0
0	0	0	0	0

0	0	0	0	0
0	0	0	0	0
0	0	0	0	0
0	0	0	0	0
0	0	0	0	0
0	0	0	0	0
0	0	0	0	0
0	0	0	0	1
0	0	0	0	0

0	0	0	0	0
0	0	0	0	0
0	0	0	0	0
0	0	0	0	0
0	0	0	0	0
0	0	0	0	0
0	0	0	0	0
0	0	0	0	0
0	0	0	0	0

0	0	0	0	0
0	0	0	0	0
0	0	0	0	0
0	0	0	0	0
0	0	0	0	0
0	0	0	0	0
0	0	0	0	0
0	0	0	0	0
0	0	0	0	0

0	0	0	0	0
0	0	0	0	0
0	0	0	0	0
1	0	1	0	0
0	0	0	0	0
0	0	0	0	0
0	0	0	0	0
0	0	0	0	0
0	0	0	0	1

0	0	0	0	0
0	0	0	0	0
0	0	0	0	0
0	0	0	0	0
0	0	0	0	0
0	0	0	0	0
1	0	0	0	0
0	0	0	0	0
1	0	0	0	1

0	0	0	0	0
0	0	0	0	0
0	0	0	0	0
0	0	0	0	0
0	0	0	0	0
0	0	0	0	0
0	0	0	0	0
0	0	0	0	0
0	0	0	0	0

0	0	0	0	0
0	0	0	0	0
0	0	0	0	0
0	0	0	0	0
0	0	0	0	0
0	0	0	0	0
0	0	0	0	0
0	0	0	0	0
0	0	0	0	0

1	0	0	0	0
0	0	0	0	0
0	0	0	0	0
0	0	0	0	0
0	0	0	0	0
0	0	0	0	0
0	0	0	0	0
0	0	0	0	0
0	0	0	0	0

0	0	0	0	0
0	0	0	0	0
0	0	0	0	0
0	0	0	0	0
0	0	0	0	0
0	0	0	0	0
0	0	0	0	0
0	0	0	0	0
0	0	0	0	0

0	0	0	0	0
1	1	0	0	0
1	0	0	0	0
0	0	0	0	0
0	0	0	0	0
0	0	0	0	0
0	0	0	0	0
0	0	0	0	0
0	0	0	0	0

0	0	0	0	0
1	0	0	0	0
1	0	1	0	0
0	0	0	0	0
0	0	0	0	0
0	0	0	0	0
0	0	0	0	0
0	0	0	0	0
0	0	0	0	0

0	0	0	0	0
0	0	0	0	0
0	0	0	0	0
0	0	0	0	0
0	0	0	0	0
0	0	0	0	0
0	0	0	0	0
0	0	0	0	0
0	0	0	0	0

0	0	0	0	0
0	0	0	0	0
0	0	0	0	0
0	0	0	0	0
0	0	0	0	0
0	0	0	0	0
0	0	0	0	0
0	0	0	0	0
0	0	0	0	0

0	0	0	0	0
0	0	0	0	0
0	0	0	0	0
0	0	0	0	0
0	0	0	0	1
0	0	0	0	0
0	0	0	0	0
0	0	0	0	0
0	0	0	0	0

0	0	0	0	0
0	0	0	0	0
0	0	0	0	1
0	0	0	0	0
0	0	0	0	0
0	0	0	0	0
0	0	0	0	0
0	0	0	0	0
0	0	0	0	0

0	0	0	0	0
0	0	0	0	0
0	0	0	0	0
0	0	0	0	0
0	0	0	0	0
0	0	0	0	0
0	0	0	0	0
0	0	0	0	0
0	0	0	0	0

0	0	0	0	0
0	0	0	0	0
0	0	0	0	0
0	0	0	0	0
0	0	0	0	0
0	0	0	0	0
0	0	0	0	0
0	0	0	0	0
0	0	0	0	0

0	0	0	0	0
0	0	0	0	0
1	1	1	1	0
0	0	0	0	0
0	0	0	0	0
0	0	0	0	0
1	0	0	0	0
0	0	0	0	0
0	0	0	0	0

0	0	0	0	0
0	0	0	0	0
0	0	0	0	0
0	0	0	0	0
1	1	1	1	0
0	0	0	0	0
0	0	0	0	0
0	0	0	0	0
0	0	0	0	0

0	0	0	0	0
0	0	0	0	0
0	0	0	0	0
0	0	0	0	0
1	1	1	1	0
0	0	0	0	0
0	0	0	0	0
0	0	0	0	0
0	0	0	0	0

0	0	0	0	0
0	0	0	0	0
0	0	0	0	0
1	0	0	0	0
0	0	0	0	0
0	0	0	0	0
0	0	0	0	0
0	0	0	0	0
0	0	0	0	0

1	0	0	0	0
1	0	1	0	0
0	0	0	0	0
0	0	0	0	0
0	0	0	0	0
0	0	0	0	0
0	0	0	0	0
0	0	0	0	0
0	0	0	0	0

0	0	0	0	0
0	0	0	0	0
0	0	0	0	0
0	0	0	0	0
0	0	0	0	0
1	0	0	0	0
0	0	0	0	0
0	0	0	0	0
0	0	0	0	0

0	0	0	0	0
0	0	0	0	0
0	0	0	0	0
0	0	0	0	0
0	0	0	0	0
0	0	0	0	0
0	0	0	0	0
0	0	0	0	0
0	0	0	0	0

0	1	1	0	0
0	0	0	0	0
0	0	0	0	0
0	0	0	0	0
0	0	0	0	0
0	0	0	0	0
0	0	0	0	0
0	0	0	0	0
0	0	0	0	0

0	0	0	0	0
1	0	1	1	0
0	0	0	0	0
0	0	0	0	0
0	0	0	0	0
0	0	0	0	0
0	0	0	0	0
0	0	0	0	0
0	0	0	0	0

0	0	0	0	0
0	0	0	0	0
0	0	0	0	0
0	0	0	0	0
1	0	0	0	0
0	0	0	0	0
0	0	0	0	0
0	0	0	0	0
0	0	0	0	0

0	0	0	0	0
0	0	0	0	0
0	0	0	0	0
0	0	0	0	0
0	0	0	0	0
0	0	0	0	0
0	0	0	0	0
0	0	0	0	0
1	0	0	0	0

0	0	0	0	0
0	0	0	0	1
0	0	0	0	0
0	0	0	0	0
0	0	0	0	0
0	0	0	0	0
0	0	0	0	0
0	0	0	0	0
0	1	0	0	0

0	0	0	0	0
0	0	0	0	0
0	0	0	0	0
0	0	0	0	0
0	0	0	0	0
0	0	0	0	0
0	0	0	0	0
0	0	0	0	0
0	0	0	0	0

0	0	0	0	0
0	0	0	0	0
0	0	0	0	0
0	0	0	0	0
0	0	0	0	0
1	0	1	0	0
0	0	0	0	1
0	0	0	0	0
0	0	0	0	0

0	0	0	0	0
0	0	0	0	0
0	0	0	0	0
0	0	0	0	0
0	0	0	0	0
0	0	0	0	0
0	0	0	0	0
0	0	0	0	0
0	0	0	0	0

0	0	0	0	0
0	0	0	0	0
1	0	0	0	0
0	0	0	0	0
0	0	0	0	0
0	0	0	0	1
0	0	0	0	0
1	1	1	1	1
0	0	0	0	0

1	0	0	0	0
0	0	0	0	0
0	0	0	0	0
0	0	0	0	0
0	0	0	0	0
0	0	0	0	0
0	0	0	0	0
0	0	0	0	0
0	0	0	0	0

0	0	0	0	0
0	0	0	0	0
0	0	0	0	0
0	0	0	0	0
0	0	0	0	0
0	0	0	0	0
0	0	0	0	0
0	0	0	0	0
0	0	0	0	0

0	0	0	0	0
0	0	0	0	0
0	0	0	0	0
0	0	0	0	0
0	0	0	0	0
1	1	0	0	0
0	0	0	0	0
0	0	0	0	0
0	0	0	0	0

0	0	0	0	0
0	0	0	0	0
0	0	0	0	0
0	0	0	0	0
0	0	0	0	0
0	0	0	0	0
0	0	0	0	0
0	0	0	0	0
0	0	0	0	0

0	1	1	0	0
0	0	0	0	0
0	0	0	0	0
0	0	0	0	0
0	0	0	0	0
0	0	0	0	1
0	0	0	0	0
0	0	0	0	0
0	0	0	0	0

0	0	0	0	0
0	0	0	0	0
0	0	0	0	0
1	1	1	1	0
0	0	0	0	0
0	0	0	0	0
1	1	1	1	1
0	0	0	0	0
0	0	0	0	0

0	0	0	0	0
0	0	0	0	0
0	0	0	0	0
1	0	0	0	0
1	1	0	0	0
0	0	0	0	0
0	0	0	0	0
0	0	0	0	0
0	0	0	0	0

0	0	0	0	0
0	0	0	0	0
0	0	0	0	0
0	0	0	0	0
0	0	0	0	0
0	0	0	0	0
0	0	0	0	0
0	0	0	0	0
0	0	0	0	0

0	0	0	0	0
0	0	0	0	0
0	0	0	0	0
0	0	0	0	0
0	0	0	0	0
1	0	0	0	0
0	0	0	0	0
0	0	0	0	0
0	0	0	0	1

0	0	0	0	0
0	0	0	0	0
0	0	0	0	0
0	0	0	0	0
0	0	0	0	0
0	0	0	0	0
0	0	0	0	0
0	0	0	0	0
0	0	0	0	0

0	0	0	0	0
0	0	0	0	0
1	0	0	0	0
0	0	0	0	0
0	0	0	0	0
0	0	0	0	0
0	0	0	0	0
0	0	0	0	0
0	0	0	0	0

0	0	0	0	0
0	0	0	0	0

INSULTADOO	HUMILLADOO	DESCALIFICADOO	Enlosúltimosseismeses Insultado	Enlosúltimosseismeses Humillado
0	0	1	0	0
0	0	0	0	0
0	0	0	0	0
0	0	0	0	0
0	0	0	0	0
0	0	0	0	0
0	0	0	0	0
0	0	0	0	0

1	0	1	1	0
1	0	0	2	0
0	0	0	0	0
0	0	0	0	0
0	0	0	0	0
0	0	0	0	0
0	0	0	0	0
1	1	1	2	2
1	1	1	2	2

1	1	0	1	1
0	0	0	0	0
0	0	0	0	0
1	1	1	2	2
0	1	1	0	1
0	0	0	0	0
0	1	1	0	1
0	0	0	0	0
0	0	0	0	0

0	0	0	0	0
1	1	1	2	2
0	0	0	0	0
0	0	0	0	0
0	0	0	0	0
0	1	0	0	1
0	0	0	0	0
0	0	0	0	0
0	0	0	0	0

0	0	0	0	0
0	0	0	0	0
0	0	0	0	0
0	0	0	0	0
0	0	0	0	0
0	0	0	0	0
0	0	0	0	0
0	0	0	0	0
0	0	0	0	0

0	0	0	0	0
0	0	0	0	0
0	0	0	0	0
1	1	1	1	2
1	1	1	2	2
0	0	0	0	0
1	1	1	2	2
1	1	1	2	2
0	0	0	0	0

0	0	0	0	0
0	0	0	0	0
0	0	0	0	0
0	0	0	0	0
0	0	0	0	0
0	0	0	0	0
0	1	0	0	2
0	0	0	0	0
1	0	1	2	0

1	0	0	2	0
1	1	1	2	2
0	0	0	0	0
0	0	0	0	0
0	0	0	0	0
0	0	0	0	0
0	1	1	0	1
0	0	0	0	0
0	0	0	0	0

0	0	0	0	0
0	0	0	0	0
0	0	0	0	0
1	1	1	2	2
0	1	1	0	2
0	0	0	0	0
0	0	0	0	0
0	0	0	0	0
1	1	1	2	2

0	0	1	0	0
1	1	1	1	1
0	0	0	0	0
0	0	0	0	0
0	0	0	0	0
0	0	0	0	0
1	1	1	2	2
0	0	0	0	0
1	1	1	2	2

0	0	0	0	0
0	0	0	0	0
1	1	1	2	2
0	0	0	0	0
1	0	0	1	0
1	1	0	2	1
0	0	0	0	0
0	0	0	0	0
0	0	0	0	0

1	0	1	1	0
0	1	1	0	1
0	0	0	0	0
1	0	0	1	0
0	0	0	0	0
0	0	0	0	0
0	0	0	0	0
0	0	0	0	0
0	0	0	0	0

1	1	1	2	2
0	0	0	0	0
0	0	1	0	0
0	0	0	0	0
0	0	0	0	0
1	1	1	2	2
0	0	0	0	0
0	0	0	0	0
1	1	1	1	1

0	0	0	0	0
0	0	0	0	0
1	1	1	2	2
0	0	0	0	0
1	1	1	2	2
0	0	0	0	0
1	1	1	2	2
0	0	0	0	0
0	0	0	0	0

0	0	0	0	0
1	1	1	2	2
1	1	1	2	2
1	1	1	2	2
0	0	0	0	0
0	0	0	0	0
0	0	0	0	0
0	0	0	0	0
0	0	0	0	0

0	0	0	0	0
1	1	1	1	1
1	1	1	2	2
1	1	1	2	2
0	0	0	0	0
0	0	0	0	0
0	0	0	0	0
0	0	0	0	0
0	0	0	0	0

0	0	0	0	0
0	0	0	0	0
0	0	0	0	0
1	0	0	2	0
0	0	0	0	0
1	1	1	2	1
1	1	1	2	2
0	0	0	0	0
0	0	0	0	0

0	0	0	0	0
1	1	1	1	1
0	0	0	0	0
1	0	0	1	0
0	0	0	0	0
0	0	0	0	0
0	0	0	0	0
0	0	0	0	0
0	0	0	0	0

0	1	1	0	2
0	0	0	0	0
1	1	0	1	1
1	1	1	2	2
1	1	1	2	2
0	0	0	0	0
0	0	0	0	0
0	0	0	0	0
0	0	0	0	0

1	0	0	2	0
0	0	0	0	0
0	0	0	0	0
0	0	0	0	0
0	0	0	0	0
1	0	0	1	0
0	0	1	0	0
0	1	1	0	1
0	0	0	0	0

1	1	1	2	2
0	0	0	0	0
0	0	0	0	0
0	0	0	0	0
1	1	1	2	2
0	0	0	0	0
0	0	0	0	0
0	0	0	0	0
1	1	1	1	1

0	1	0	0	2
0	0	1	0	0
0	0	0	0	0
0	0	0	0	0
0	0	0	0	0
0	0	0	0	0
0	0	0	0	0
0	0	0	0	0
1	0	0	1	0

1	1	1	2	2
0	0	0	0	0
1	1	1	2	2
1	1	1	2	2
1	0	0	1	0
1	0	0	2	0
1	1	1	2	2
0	0	0	0	0
1	1	1	2	2

0	0	0	0	0
0	0	0	0	0
1	1	1	2	2
0	0	0	0	0
0	0	0	0	0
1	1	1	1	1
0	0	0	0	0
0	0	0	0	0
1	1	1	2	2

1	1	1	2	2
0	0	0	0	0
0	0	0	0	0
1	1	1	2	2
1	1	1	2	2
0	0	0	0	0
0	0	0	0	0
1	1	1	2	2
0	0	0	0	0

0	0	0	0	0
1	1	1	2	2
0	0	0	0	0
1	1	1	2	2
0	0	0	0	0
1	1	1	1	1
1	0	0	1	0
1	0	0	1	0
0	1	1	0	1

1	1	1	2	2
1	1	1	2	2
1	1	0	2	1
0	0	0	0	0
0	0	0	0	0
1	1	1	2	2
0	0	0	0	0
0	0	0	0	0
0	0	0	0	0

0	0	0	0	0
0	0	0	0	0
0	0	0	0	0
1	1	1	2	2
0	0	0	0	0
1	0	1	1	0
0	0	0	0	0
0	0	0	0	0
1	1	0	1	1

0	0	0	0	0
0	0	0	0	0
0	0	0	0	0
1	0	1	2	0
0	0	1	0	0
0	1	0	0	1
0	0	0	0	0
0	0	0	0	0
0	0	0	0	0

1	1	1	2	2
0	0	0	0	0
0	0	0	0	0
0	0	1	0	0
0	0	0	0	0
1	0	0	2	0
0	0	0	0	0
0	0	0	0	0
0	0	0	0	0

1	0	0	1	0
1	1	1	2	1
0	0	0	0	0
0	0	0	0	0
0	0	0	0	0
0	0	0	0	0
0	0	0	0	0
0	0	0	0	0
0	0	0	0	0

0	0	0	0	0
0	0	0	0	0
0	0	0	0	0
0	0	0	0	0
0	0	1	0	0
0	0	0	0	0
0	0	0	0	0
0	0	0	0	0
0	0	0	0	0

1	1	1	2	2
0	0	0	0	0
1	1	1	1	2
0	0	1	0	0
0	0	1	0	0
0	0	0	0	0
1	1	1	2	1
0	0	0	0	0
0	0	0	0	0

0	0	1	0	0
0	1	1	0	1
0	0	0	0	0
0	0	0	0	0
1	1	0	2	1
0	0	0	0	0
0	0	0	0	0
1	1	1	2	2
1	1	1	2	2

0	0	0	0	0
0	0	0	0	0
1	1	1	2	2
1	1	1	2	2
0	0	0	0	0
0	0	0	0	0
0	0	0	0	0
0	0	0	0	0
0	0	0	0	0

0	0	0	0	0
0	0	0	0	0
0	0	0	0	0
1	1	1	2	2
0	0	0	0	0
1	1	1	2	2
1	1	1	2	2
0	0	0	0	0
0	0	0	0	0

0	0	0	0	0
0	0	0	0	0
0	0	0	0	0
1	1	1	2	2
1	1	1	2	2
1	1	1	2	2
1	1	1	2	2
0	0	0	0	0
0	0	0	0	0

1	1	1	1	1
0	0	0	0	0
1	1	1	2	2
0	0	0	0	0
1	1	1	2	2
1	1	1	2	2
0	0	0	0	0
1	1	1	2	2
0	0	0	0	0

1	1	1	2	2
0	0	0	0	0
0	0	0	0	0
0	0	0	0	0
0	0	0	0	0
0	0	0	0	0
1	1	1	2	2
0	0	0	0	0
0	0	0	0	0

0	0	0	0	0
0	0	0	0	0
0	0	0	0	0
0	0	0	0	0
0	0	0	0	0
0	0	0	0	0
0	0	0	0	0
0	0	0	0	0
0	0	0	0	0

0	0	0	0	0
0	0	0	0	0
0	0	0	0	0
1	1	1	2	2
0	0	0	0	0
1	1	1	2	2
0	0	0	0	0
0	0	0	0	0
0	0	0	0	0

0	0	0	0	0
1	0	1	1	0
0	0	0	0	0
0	0	0	0	0
0	0	0	0	0
0	0	0	0	0
0	0	0	0	0
0	0	0	0	0
0	1	1	0	2

0	1	1	0	2
0	1	1	0	2
0	0	0	0	0
0	0	0	0	0
0	0	0	0	0
1	1	1	2	2
0	0	0	0	0
1	1	0	2	1
0	0	0	0	0

0	0	0	0	0
0	0	0	0	0
0	0	0	0	0
1	1	1	2	2
0	0	0	0	0
0	0	0	0	0
1	1	1	2	2
1	1	1	2	2
1	1	1	1	1

0	0	0	0	0
0	0	0	0	0
0	0	0	0	0
1	1	1	2	2
1	1	1	2	2
0	0	0	0	0
0	0	0	0	0
0	0	0	0	0
0	0	0	0	0

0	0	0	0	0
0	0	0	0	0
0	1	1	0	1
0	0	0	0	0
0	0	0	0	0
0	0	0	0	0
0	0	0	0	0
0	0	0	0	0
0	0	0	0	0

1	0	1	2	0
1	0	0	1	0
0	0	0	0	0
0	0	0	0	0
0	0	0	0	0
1	1	1	2	2
1	1	1	2	2
1	1	1	1	1
1	1	1	2	2

0	0	0	0	0
0	0	0	0	0
0	0	0	0	0
0	0	0	0	0
1	1	1	2	2
0	0	0	0	0
0	0	0	0	0
0	0	0	0	0
0	0	0	0	0

0	0	0	0	0
0	0	0	0	0
0	0	0	0	0
0	0	0	0	0
0	0	0	0	0
0	0	0	0	0
1	1	1	1	1
0	0	0	0	0
0	0	0	0	0

0	0	0	0	0
0	0	0	0	0

Enlosúltimosseismeses Descalificado	Enlosúltimosseismeses Amenazadoconhacerledañoaud	Enlosúltimosseismeses Haamenazadoaalguienqueudquier	Enlosúltimosseismeses Lehahechoalgoquelaatemorizos	Enlosúltimosseismeses Lahadejadoseismesessinapoyoe
2	0	0	0	0
0	0	0	0	0
0	0	0	0	0
0	0	0	0	0
0	0	0	0	0
0	0	0	0	0
0	0	0	0	0
0	0	0	0	0

2	0	0	0	0
0	1	0	2	0
0	0	0	0	0
0	0	0	0	0
0	0	0	0	0
0	0	0	0	0
0	0	0	0	0
2	0	0	0	2
2	2	0	0	0

0	0	0	0	0
0	0	0	0	0
0	0	0	0	0
2	1	0	0	0
1	0	0	1	0
0	0	0	0	0
1	0	0	0	0
0	0	0	0	0
0	0	0	0	0

0	0	0	0	0
2	2	1	0	0
0	0	0	0	0
0	0	0	0	0
0	0	0	0	0
0	0	0	0	0
0	0	0	0	0
0	0	0	0	0
0	0	0	0	0

0	0	0	0	0
0	0	0	0	0
0	0	0	0	0
0	0	0	0	0
0	0	0	0	0
0	0	0	0	0
0	0	0	0	0
0	0	0	0	0
0	0	0	0	0

0	0	0	0	0
0	0	0	0	0
0	0	0	0	0
2	0	0	0	0
1	2	2	1	0
0	0	0	0	0
2	0	0	0	0
2	0	0	2	0
0	0	0	0	0

0	0	0	0	0
0	0	0	0	0
0	0	0	0	0
0	0	0	0	0
0	0	0	0	0
0	0	0	0	0
0	0	0	0	0
0	0	0	0	0
2	0	0	0	0

0	0	0	0	0
2	0	0	0	0
0	0	0	0	0
0	0	0	0	0
0	0	0	0	0
0	0	0	0	0
2	0	0	0	0
0	0	0	0	0
0	0	0	0	0

0	0	0	2	0
0	0	0	0	0
0	0	0	0	0
2	2	1	0	0
2	0	0	0	0
0	0	0	0	0
0	0	0	0	0
0	0	0	0	0
2	0	0	2	0

1	0	0	0	0
1	0	0	0	0
0	0	0	0	0
0	0	0	0	0
0	0	0	0	0
0	0	0	0	0
2	0	0	0	2
0	0	0	0	0
2	2	0	2	2

0	0	0	0	0
0	0	0	0	0
2	0	0	0	0
0	0	0	0	0
0	0	0	0	0
0	0	0	0	0
0	0	0	0	0
0	0	0	0	0
0	0	0	0	0

1	0	0	0	0
1	0	0	0	0
0	0	0	0	0
0	0	0	0	0
0	0	0	0	0
0	0	0	0	0
0	0	0	0	0
0	0	0	0	0
0	0	0	0	0

2	2	2	2	2
0	0	0	0	0
1	0	0	0	0
0	0	0	0	0
0	0	0	0	0
2	2	0	2	2
0	0	0	0	0
0	0	0	0	0
1	0	0	1	0

0	0	0	0	0
0	0	0	0	0
2	0	0	0	0
0	0	0	0	0
2	0	0	0	0
0	0	0	0	0
2	0	0	0	0
0	0	0	0	0
0	0	0	0	0

0	0	0	0	0
2	2	0	2	0
2	0	1	0	0
1	2	2	0	2
0	0	0	0	0
0	0	0	0	0
0	0	0	0	0
0	0	0	0	0
0	0	0	0	0

0	0	0	0	0
1	1	0	0	0
2	0	0	2	0
2	0	1	0	0
0	0	0	0	0
0	0	0	0	0
0	0	0	0	0
0	0	0	0	0
0	0	0	0	0

0	0	0	0	0
0	0	0	0	0
0	0	0	0	0
0	0	0	0	0
0	0	0	0	0
1	0	0	0	0
2	0	0	0	0
0	0	0	0	0
0	0	0	0	0

0	0	0	0	0
1	0	0	0	0
0	0	0	0	2
0	0	0	0	0
0	0	0	0	0
0	0	0	0	0
0	0	0	0	0
0	0	0	0	0
0	0	0	0	0

2	0	0	0	0
0	0	0	0	0
0	0	0	0	2
2	0	0	1	1
2	1	0	2	0
0	0	0	0	0
0	0	0	0	0
0	0	0	0	1
0	0	0	0	0

0	0	0	0	0
0	0	0	0	0
0	0	0	1	0
0	0	0	0	0
0	0	0	0	0
0	0	0	0	0
2	0	0	0	0
2	0	0	0	0
0	0	0	0	0

2	0	0	0	0
0	0	0	0	0
0	0	0	0	0
0	0	0	1	0
2	2	0	2	0
0	0	0	0	0
0	0	0	0	0
0	0	0	0	0
1	0	0	1	0

0	0	0	1	2
1	0	0	0	0
0	0	0	0	0
0	0	0	0	0
0	0	0	0	0
0	0	0	0	0
0	0	0	0	0
0	0	0	0	0
0	0	0	0	0

2	0	0	2	0
0	0	0	0	0
2	2	0	2	0
2	0	0	2	0
0	0	0	0	0
0	0	0	0	0
2	2	2	2	0
0	0	0	0	0
2	0	1	0	1

0	0	0	0	0
0	0	0	0	0
2	1	1	1	0
0	0	0	0	0
0	1	0	0	2
1	0	0	0	0
0	0	0	0	0
0	0	0	0	0
2	0	0	0	0

2	0	2	0	0
0	0	0	0	0
0	0	0	0	0
2	2	0	0	0
2	0	0	0	2
0	0	0	0	0
0	0	0	0	0
2	2	0	0	0
0	0	0	0	0

0	0	0	0	0
2	2	2	2	0
0	0	0	0	0
2	0	0	0	0
0	0	0	0	0
1	0	0	1	0
0	0	0	0	0
0	0	0	0	1
1	0	0	2	0

2	2	0	2	0
2	1	0	1	0
0	0	0	1	0
0	0	0	2	0
0	0	0	2	0
2	0	0	0	0
0	0	0	0	0
0	0	0	0	0
0	0	0	0	0

0	0	0	0	0
0	0	0	0	0
0	0	0	0	0
2	2	2	2	0
0	0	0	0	0
1	0	0	0	2
0	0	0	0	0
0	0	0	0	0
0	0	0	0	0

0	0	0	0	0
0	0	0	0	0
0	0	0	0	0
1	0	0	0	0
2	0	0	2	0
0	0	0	0	0
0	0	0	0	0
0	0	0	0	0
0	0	0	0	0

2	2	2	0	0
0	0	0	0	0
0	0	0	0	0
2	0	0	0	0
0	0	0	0	0
0	0	0	0	0
0	0	0	0	0
0	0	0	0	0
0	0	0	0	0

0	0	0	0	0
2	2	1	2	2
0	0	0	0	0
0	0	0	0	0
0	0	0	0	0
0	0	0	0	0
0	0	0	0	0
0	0	0	0	0
0	0	0	0	0

0	0	0	0	0
0	0	0	0	0
0	0	0	0	0
0	0	0	0	0
2	0	0	0	0
0	0	0	0	0
0	0	0	0	0
0	0	0	0	0
0	0	0	0	0

2	2	0	0	2
0	0	0	0	0
2	0	0	0	0
1	0	0	0	0
2	0	0	0	0
0	0	0	0	0
1	0	0	0	0
0	0	0	0	0
0	0	0	0	0

1	0	0	0	0
1	1	0	0	0
0	0	0	1	0
0	0	0	0	0
0	0	0	2	0
0	0	0	0	0
0	0	0	0	0
2	2	1	0	1
2	0	0	2	0

0	0	0	0	0
0	0	0	0	0
2	0	0	2	0
2	0	0	0	0
0	0	0	0	0
0	0	0	0	0
0	0	0	0	0
0	0	0	0	0
0	0	0	0	0

0	0	0	0	0
0	0	0	0	0
0	0	0	0	0
2	0	0	0	0
0	0	0	0	0
2	2	0	1	1
2	0	0	0	2
0	0	0	0	0
0	0	0	0	0

0	0	0	0	0
0	0	0	0	0
0	0	0	0	0
2	1	2	0	0
2	0	0	0	0
2	0	0	0	0
2	2	0	2	2
0	0	0	0	0
0	0	0	0	0

1	0	0	0	0
0	0	0	0	0
2	1	0	1	0
0	0	0	0	0
2	2	2	2	2
2	0	0	0	0
0	0	0	0	0
2	2	2	2	2
0	0	0	0	0

2	0	0	0	0
0	0	0	0	0
0	0	0	0	0
0	0	0	0	0
0	0	0	0	0
0	0	0	0	0
2	2	0	0	1
0	0	0	0	0
0	0	0	0	0

0	0	0	0	0
0	0	0	0	0
0	0	0	0	0
0	0	0	0	0
0	0	0	0	0
0	0	0	0	0
0	0	0	0	0
0	0	0	0	0
0	0	0	0	0

0	0	0	0	0
0	0	0	0	0
0	0	0	0	0
2	0	0	2	2
0	0	0	0	0
2	0	0	1	0
0	0	0	0	0
0	0	0	0	0
0	0	0	0	0

0	0	0	0	0
1	0	0	0	0
0	0	0	0	0
0	0	0	0	0
0	0	0	0	0
0	0	0	0	0
0	0	0	0	0
0	0	0	0	0
1	0	0	0	0

2	0	0	0	0
2	0	0	0	0
0	0	0	0	0
0	0	0	0	0
0	0	0	0	0
2	1	0	0	0
0	0	0	0	0
0	0	0	0	0
0	0	0	0	0

0	0	0	0	0
0	0	0	0	0
0	0	0	0	0
2	2	2	2	2
0	0	0	0	0
0	0	0	0	0
2	2	2	2	0
2	0	0	0	0
1	0	0	0	0

0	0	0	0	0
0	0	0	0	0
0	0	0	0	0
2	0	0	2	0
2	0	0	1	0
0	0	0	0	0
0	0	0	0	0
0	0	0	0	0
0	0	0	0	0

0	0	0	0	0
0	0	0	0	0
1	0	0	0	0
0	0	0	0	0
0	0	0	0	0
0	0	0	0	0
0	0	0	0	0
0	0	0	0	0
0	0	0	0	0

1	0	0	0	0
0	0	0	2	0
0	0	0	0	0
0	0	0	0	0
0	0	0	0	0
2	0	0	1	0
2	2	0	0	0
1	0	1	0	0
2	0	0	1	0

0	0	0	0	0
0	0	0	0	0
0	0	0	0	0
0	0	0	0	0
2	0	0	2	0
0	0	0	0	0
0	0	0	0	0
0	0	0	0	0
0	0	0	0	0

0	0	0	0	0
0	0	0	0	0
0	0	0	0	0
0	0	0	0	0
0	0	0	0	0
0	0	0	0	0
1	0	0	0	2
0	0	0	0	0
0	0	0	0	0

0	0	0	0	0
0	0	0	0	0

EnlosúltimosseismesesLehadadobofetada	EnlosúltimosseismesesLahapateado	EnlosúltimosseismesesLehadadopuñetazos	EnlosúltimosseismesesLehadadounapaliza	EnlosúltimosseismesesLahaforzadoatenerrelacionesse
0	0	0	0	0
0	0	0	0	0
0	0	0	0	0
0	0	0	0	0
0	0	0	0	0
0	0	0	0	0
0	0	0	0	0
0	0	0	0	0

0	0	0	0	0
0	0	0	0	0
0	0	0	0	0
0	0	0	0	0
0	0	0	0	0
0	0	0	0	0
0	0	0	0	0
0	0	0	0	0
0	0	0	0	0

0	0	0	0	0
0	0	0	0	0
0	0	0	0	0
1	1	1	1	0
1	0	0	0	1
0	0	0	0	0
0	0	0	0	0
0	0	0	0	0
0	0	0	0	0

0	0	0	0	0
0	0	0	0	0
0	0	0	0	0
0	0	0	0	0
0	0	0	0	0
0	0	0	0	0
0	0	0	0	0
0	0	0	0	0
0	0	0	0	0

0	0	0	0	0
0	0	0	0	0
0	0	0	0	0
0	0	0	0	0
0	0	0	0	0
0	0	0	0	0
0	0	0	0	0
0	0	0	0	0
0	0	0	0	0

0	0	0	0	0
0	0	0	0	0
0	0	0	0	0
0	0	0	0	0
0	0	0	0	0
0	0	0	0	0
0	0	0	0	0
0	0	0	0	1
0	0	0	0	0

0	0	0	0	0
0	0	0	0	0
0	0	0	0	0
0	0	0	0	0
0	0	0	0	0
0	0	0	0	0
0	0	0	0	0
0	0	0	0	0
0	0	0	0	0

0	0	0	0	0
0	0	0	0	0
0	0	0	0	0
0	0	0	0	0
0	0	0	0	0
0	0	0	0	0
0	0	0	0	0
0	0	0	0	0
0	0	0	0	0

0	0	0	0	0
0	0	0	0	0
0	0	0	0	0
2	0	2	0	0
0	0	0	0	0
0	0	0	0	0
0	0	0	0	0
0	0	0	0	0
0	0	0	0	1

0	0	0	0	0
0	0	0	0	0
0	0	0	0	0
0	0	0	0	0
0	0	0	0	0
0	0	0	0	0
1	0	0	0	0
0	0	0	0	0
2	0	0	0	2

0	0	0	0	0
0	0	0	0	0
0	0	0	0	0
0	0	0	0	0
0	0	0	0	0
0	0	0	0	0
0	0	0	0	0
0	0	0	0	0
0	0	0	0	0

0	0	0	0	0
0	0	0	0	0
0	0	0	0	0
0	0	0	0	0
0	0	0	0	0
0	0	0	0	0
0	0	0	0	0
0	0	0	0	0
0	0	0	0	0

2	0	0	0	0
0	0	0	0	0
0	0	0	0	0
0	0	0	0	0
0	0	0	0	0
0	0	0	0	0
0	0	0	0	0
0	0	0	0	0
0	0	0	0	0

0	0	0	0	0
0	0	0	0	0
0	0	0	0	0
0	0	0	0	0
0	0	0	0	0
0	0	0	0	0
0	0	0	0	0
0	0	0	0	0
0	0	0	0	0

0	0	0	0	0
2	1	0	0	0
2	0	0	0	0
0	0	0	0	0
0	0	0	0	0
0	0	0	0	0
0	0	0	0	0
0	0	0	0	0
0	0	0	0	0

0	0	0	0	0
1	0	0	0	0
2	0	2	0	0
0	0	0	0	0
0	0	0	0	0
0	0	0	0	0
0	0	0	0	0
0	0	0	0	0
0	0	0	0	0

0	0	0	0	0
0	0	0	0	0
0	0	0	0	0
0	0	0	0	0
0	0	0	0	0
0	0	0	0	0
0	0	0	0	0
0	0	0	0	0
0	0	0	0	0

0	0	0	0	0
0	0	0	0	0
0	0	0	0	0
0	0	0	0	0
0	0	0	0	0
0	0	0	0	0
0	0	0	0	0
0	0	0	0	0
0	0	0	0	0

0	0	0	0	0
0	0	0	0	0
0	0	0	0	0
0	0	0	0	0
0	0	0	0	1
0	0	0	0	0
0	0	0	0	0
0	0	0	0	0
0	0	0	0	0

0	0	0	0	0
0	0	0	0	0
0	0	0	0	1
0	0	0	0	0
0	0	0	0	0
0	0	0	0	0
0	0	0	0	0
0	0	0	0	0
0	0	0	0	0

0	0	0	0	0
0	0	0	0	0
0	0	0	0	0
0	0	0	0	0
0	0	0	0	0
0	0	0	0	0
0	0	0	0	0
0	0	0	0	0
0	0	0	0	0

0	0	0	0	0
0	0	0	0	0
0	0	0	0	0
0	0	0	0	0
0	0	0	0	0
0	0	0	0	0
0	0	0	0	0
0	0	0	0	0
0	0	0	0	0

0	0	0	0	0
0	0	0	0	0
2	2	2	1	0
0	0	0	0	0
0	0	0	0	0
0	0	0	0	0
1	0	0	0	0
0	0	0	0	0
0	0	0	0	0

0	0	0	0	0
0	0	0	0	0
0	0	0	0	0
0	0	0	0	0
1	1	1	1	0
0	0	0	0	0
0	0	0	0	0
0	0	0	0	0
0	0	0	0	0

0	0	0	0	0
0	0	0	0	0
0	0	0	0	0
0	0	0	0	0
2	2	2	2	0
0	0	0	0	0
0	0	0	0	0
0	0	0	0	0
0	0	0	0	0

0	0	0	0	0
0	0	0	0	0
0	0	0	0	0
1	0	0	0	0
0	0	0	0	0
0	0	0	0	0
0	0	0	0	0
0	0	0	0	0
0	0	0	0	0

2	0	0	0	0
1	0	1	0	0
0	0	0	0	0
0	0	0	0	0
0	0	0	0	0
0	0	0	0	0
0	0	0	0	0
0	0	0	0	0
0	0	0	0	0

0	0	0	0	0
0	0	0	0	0
0	0	0	0	0
0	0	0	0	0
0	0	0	0	0
1	0	0	0	0
0	0	0	0	0
0	0	0	0	0
0	0	0	0	0

0	0	0	0	0
0	0	0	0	0
0	0	0	0	0
0	0	0	0	0
0	0	0	0	0
0	0	0	0	0
0	0	0	0	0
0	0	0	0	0
0	0	0	0	0

0	1	1	0	0
0	0	0	0	0
0	0	0	0	0
0	0	0	0	0
0	0	0	0	0
0	0	0	0	0
0	0	0	0	0
0	0	0	0	0
0	0	0	0	0

0	0	0	0	0
2	0	2	2	0
0	0	0	0	0
0	0	0	0	0
0	0	0	0	0
0	0	0	0	0
0	0	0	0	0
0	0	0	0	0
0	0	0	0	0

0	0	0	0	0
0	0	0	0	0
0	0	0	0	0
0	0	0	0	0
1	0	0	0	0
0	0	0	0	0
0	0	0	0	0
0	0	0	0	0
0	0	0	0	0

0	0	0	0	0
0	0	0	0	0
0	0	0	0	0
0	0	0	0	0
0	0	0	0	0
0	0	0	0	0
0	0	0	0	0
0	0	0	0	0
1	0	0	0	0

0	0	0	0	0
0	0	0	0	1
0	0	0	0	0
0	0	0	0	0
0	0	0	0	0
0	0	0	0	0
0	0	0	0	0
0	0	0	0	0
0	1	0	0	0

0	0	0	0	0
0	0	0	0	0
0	0	0	0	0
0	0	0	0	0
0	0	0	0	0
0	0	0	0	0
0	0	0	0	0
0	0	0	0	0
0	0	0	0	0

0	0	0	0	0
0	0	0	0	0
0	0	0	0	0
0	0	0	0	0
0	0	0	0	0
2	0	2	0	0
0	0	0	0	2
0	0	0	0	0
0	0	0	0	0

0	0	0	0	0
0	0	0	0	0
0	0	0	0	0
0	0	0	0	0
0	0	0	0	0
0	0	0	0	0
0	0	0	0	0
0	0	0	0	0
0	0	0	0	0

0	0	0	0	0
0	0	0	0	0
1	0	0	0	0
0	0	0	0	0
0	0	0	0	0
0	0	0	0	2
0	0	0	0	0
2	2	2	2	2
0	0	0	0	0

2	0	0	0	0
0	0	0	0	0
0	0	0	0	0
0	0	0	0	0
0	0	0	0	0
0	0	0	0	0
0	0	0	0	0
0	0	0	0	0
0	0	0	0	0

0	0	0	0	0
0	0	0	0	0
0	0	0	0	0
0	0	0	0	0
0	0	0	0	0
0	0	0	0	0
0	0	0	0	0
0	0	0	0	0
0	0	0	0	0

0	0	0	0	0
0	0	0	0	0
0	0	0	0	0
0	0	0	0	0
0	0	0	0	0
1	1	0	0	0
0	0	0	0	0
0	0	0	0	0
0	0	0	0	0

0	0	0	0	0
0	0	0	0	0
0	0	0	0	0
0	0	0	0	0
0	0	0	0	0
0	0	0	0	0
0	0	0	0	0
0	0	0	0	0
0	0	0	0	0

0	1	1	0	0
0	0	0	0	0
0	0	0	0	0
0	0	0	0	0
0	0	0	0	0
0	0	0	0	1
0	0	0	0	0
0	0	0	0	0
0	0	0	0	0

0	0	0	0	0
0	0	0	0	0
0	0	0	0	0
1	1	1	1	0
0	0	0	0	0
0	0	0	0	0
2	2	2	2	2
0	0	0	0	0
0	0	0	0	0

0	0	0	0	0
0	0	0	0	0
0	0	0	0	0
2	0	0	0	0
1	1	0	0	0
0	0	0	0	0
0	0	0	0	0
0	0	0	0	0
0	0	0	0	0

0	0	0	0	0
0	0	0	0	0
0	0	0	0	0
0	0	0	0	0
0	0	0	0	0
0	0	0	0	0
0	0	0	0	0
0	0	0	0	0
0	0	0	0	0

0	0	0	0	0
0	0	0	0	0
0	0	0	0	0
0	0	0	0	0
0	0	0	0	0
1	0	0	0	0
0	0	0	0	0
0	0	0	0	0
0	0	0	0	1

0	0	0	0	0
0	0	0	0	0
0	0	0	0	0
0	0	0	0	0
0	0	0	0	0
0	0	0	0	0
0	0	0	0	0
0	0	0	0	0
0	0	0	0	0

0	0	0	0	0
0	0	0	0	0
1	0	0	0	0
0	0	0	0	0
0	0	0	0	0
0	0	0	0	0
0	0	0	0	0
0	0	0	0	0
0	0	0	0	0

0	0	0	0	0
0	0	0	0	0

ESTÁENTRATAMIENTOPORDOLORCRÓNICOdebidoacualquieret	ESTÁENTRATAMIENTOPOROTRAPATALOGÍA	Tiempodeevoluciondelepisodiodepresivoactual	Factordesencadenantedelprimerepisodio	ADHERENCIAATRATAMIENTOSPREVIOS
0	0	3	0	0
0	1	1	6	0
0	1	1	12	1
0	1	1	12	0
0	1	0	2	0
0	0	1	0	0
0	0	2	11	0
0	1	1	4	0

0	0	38	3	0
0	0	0	0	0
1	0	0	5	0
0	0	0	2	0
1	1	1	2	0
0	0	1	1	0
1	1	3	1	0
0	1	9	11	0
0	0	3	1	0

0	0	1	1	0
0	1	1	0	0
0	1	1	0	1
0	0	0	1	0
0	0	1	0	0
1	1	1	3	0
0	0	3	6	0
0	0	1	9	0
0	0	0	0	1

1	0	3	2	0
0	0	2	3	0
1	1	1	11	0
1	0	2	5	0
0	0	1	2	0
0	0	1	3	0
0	0	0	8	1
0	0	1	0	0
0	0	1	4	0

0	0	1	1	0
0	1	1	11	0
0	0	1	1	0
0	0	1	7	0
0	0	1	1	0
1	0	1	8	0
0	0	2	0	1
0	0	2	0	0
1	0	0	1	1

0	0	10	1	0
0	0	1	7	0
1	0	25	8	0
0	0	1	9	0
0	1	1	11	0
0	1	1	0	0
0	0	0	8	0
0	0	0	4	0
0	1	0	4	0

0	0	0	2	0
1	0	0	1	0
1	1	0	1	0
0	1	1	11	0
0	1	0	8	0
0	0	0	0	0
0	1	2	6	0
0	1	1	2	0
0	0	2	1	0

0	1	1	11	1
1	0	6	10	0
1	0	1	5	0
0	0	1	3	0
0	1	2	0	0
0	0	1	1	0
1	0	2	7	1
0	1	1	2	0
1	1	1	2	0

1	1	45	0	0
0	0	12	1	0
0	1	15	3	0
1	1	1	0	0
0	0	22	9	0
0	0	14	11	0
1	1	5	3	0
1	0	1	4	0
0	1	1	0	1

0	0	2	0	1
0	0	1	2	0
0	1	1	1	0
0	0	1	10	0
0	1	2	10	1
0	0	2	13	0
1	0	1	1	0
1	0	7	0	1
1	0	1	12	0

1	0	1	9	0
0	0	1	6	0
0	0	4	0	0
0	0	1	0	0
0	0	3	10	0
0	0	1	7	0
1	1	1	12	1
0	0	2	6	0
0	0	2	7	1

0	0	2	4	0
0	0	1	4	0
0	0	1	1	0
0	0	1	0	0
0	0	1	1	1
0	0	1	8	0
0	0	1	3	0
0	0	3	8	0
0	1	1	11	1

0	0	1	10	0
0	1	1	4	0
1	0	1	10	0
1	0	1	1	1
1	0	5	0	0
1	0	2	0	0
0	1	1	7	0
0	0	2	0	0
0	0	2	8	0

0	0	1	6	0
1	0	2	10	0
1	1	4	1	0
0	0	1	11	0
0	0	1	7	0
0	1	1	0	0
0	0	1	2	0
0	0	3	6	0
0	0	0	10	1

0	0	6	6	0
0	0	10	0	0
0	0	1	0	0
0	0	4	8	0
0	0	1	2	1
0	0	4	8	0
1	1	7	14	0
0	1	1	3	0
0	1	1	10	0

0	1	2	2	0
0	0	7	2	0
1	0	1	0	1
0	0	3	6	0
0	1	1	1	0
0	0	1	10	0
0	0	1	7	0
1	0	1	5	1
0	0	1	11	0

0	1	1	6	0
1	0	1	0	0
0	1	1	6	0
0	0	1	5	0
0	0	1	11	0
1	0	1	9	0
1	0	3	4	1
0	0	1	6	0
1	0	4	1	1

0	0	1	3	1
0	1	1	1	0
1	1	1	9	0
0	0	1	9	0
1	0	1	0	0
0	0	1	1	1
0	0	1	11	0
0	0	1	0	0
0	0	1	10	0

0	1	1	3	0
0	0	1	0	0
0	1	1	6	0
0	0	3	1	0
0	1	1	2	0
0	0	1	7	0
1	0	1	6	0
0	0	1	3	1
0	0	2	2	0

1	1	1	10	0
0	0	1	3	0
1	1	1	2	1
0	1	1	10	1
1	0	1	11	0
0	0	1	3	0
0	0	6	3	0
0	0	4	11	0
0	1	1	1	0

1	0	1	1	0
0	0	1	2	0
0	1	1	5	0
0	0	1	11	0
1	1	1	3	1
1	1	3	14	1
1	0	1	8	0
0	0	1	14	1
0	0	1	0	1

0	0	1	1	0
1	0	1	7	0
1	0	1	3	0
1	0	1	2	0
0	0	1	4	0
0	1	1	3	0
1	0	1	1	0
1	0	1	1	0
0	0	1	7	0

1	0	1	10	1
1	0	1	1	0
1	0	1	6	0
0	0	1	10	0
0	0	1	11	1
0	0	1	10	0
0	0	1	1	0
0	0	1	10	1
0	0	1	1	0

0	0	1	3	0
0	0	1	5	0
0	0	1	11	1
0	0	1	12	0
0	0	1	9	0
1	0	4	3	1
0	1	1	11	0
0	0	1	11	0
0	0	4	0	1

0	0	1	0	0
0	1	2	8	1
0	0	0	6	0
0	0	1	0	0
0	0	5	0	0
1	0	1	3	0
0	0	1	1	0
1	0	1	10	0
0	0	1	7	0

1	0	1	11	1
1	0	1	1	0
0	0	1	7	0
0	0	1	6	0
0	0	1	1	0
0	0	2	9	0
0	0	1	8	0
0	0	1	3	0
0	0	1	0	0

0	0	1	11	0
0	0	1	1	0
0	0	1	2	0
0	0	1	7	0
0	1	3	7	0
0	0	2	3	0
0	0	1	0	1
0	0	0	10	0
1	0	0	2	0

0	0	1	3	1
0	0	1	10	0
1	0	1	9	0
0	0	26	0	1
0	0	1	1	1
0	0	1	7	0
0	1	2	1	0
0	0	4	10	0
0	0	1	7	1

0	1	1	13	0
0	0	1	11	0
1	0	2	1	0
0	0	1	0	0
0	0	1	1	0
0	1	1	0	0
0	0	2	9	1
1	1	1	8	1
0	0	3	11	0

0	0	1	1	1
0	0	2	1	0
0	0	1	10	0
1	1	2	1	0
0	1	0	5	0
1	1	1	14	0
0	1	2	5	0
0	0	1	6	0
1	0	1	2	1

1	0	1	0	0
0	0	1	1	0
0	0	1	4	0
1	1	1	3	1
1	1	6	1	0
0	1	1	2	0
1	1	0	2	1
1	0	1	0	0
1	1	3	0	0

1	0	1	9	1
0	0	1	11	0
1	0	1	2	1
1	0	1	0	1
0	0	1	10	0
0	0	1	3	0
0	0	1	8	0
0	0	1	3	1
0	0	1	6	1

0	1	1	8	0
0	0	1	6	1
0	0	1	4	1
0	0	1	10	0
0	1	1	7	0
0	0	3	13	0
0	0	2	2	0
1	1	3	1	0
0	1	1	1	0

0	1	1	1	0
0	1	1	7	0
0	0	13	1	0
0	0	1	4	0
0	0	1	0	0
0	0	4	1	1
1	0	2	3	1
0	0	1	0	0
1	0	1	2	1

0	0	1	0	0
0	0	1	1	0
0	0	1	8	0
0	0	2	10	1
0	0	1	0	0
0	0	1	6	0
0	1	3	5	0
0	0	1	0	1
1	0	1	11	1

0	0	1	6	0
0	0	7	1	0
0	0	1	2	0
0	0	5	0	0
0	0	1	11	0
0	0	1	0	0
0	1	1	0	0
0	0	1	0	0
0	0	1	0	0

0	0	10	11	0
1	0	2	1	1
0	0	1	6	0
0	0	1	12	0
0	0	1	1	0
0	0	20	0	0
1	0	7	1	0
0	0	1	0	0
0	1	1	0	0

1	0	1	0	0
0	1	1	0	0
0	0	3	0	0
0	0	0	0	0
0	1	1	14	0
0	0	1	0	1
0	0	1	0	0
0	0	1	0	1
0	0	1	2	1

0	0	1	1	0
0	0	1	8	0
0	0	1	0	0
1	0	1	9	0
1	1	1	0	0
1	1	1	7	0
0	0	5	0	0
1	1	1	4	1
1	0	1	6	0

1	1	65	2	0
1	0	1	1	1
0	1	1	9	0
0	0	1	9	0
0	1	1	0	1
0	0	1	11	0
0	0	1	13	1
0	0	1	0	1
0	0	1	9	0

0	0	1	1	1
0	0	1	10	0
0	0	1	0	0
0	0	1	8	0
1	0	1	2	0
0	0	1	4	0
0	1	4	6	0
0	0	1	0	0
0	0	3	8	0

0	0	1	2	0
0	1	1	2	1
0	0	1	10	0
1	0	2	2	0
1	1	46	0	0
0	0	1	3	1
0	1	1	1	1
0	0	1	4	0
0	1	3	2	1

0	0	1	0	0
0	0	1	0	0
1	0	4	2	0
0	0	1	2	1
1	1	39	6	0
1	0	0	0	0
1	0	1	13	1
1	0	1	6	0
1	0	0	6	1

0	1	1	11	1
0	0	11	12	1
0	0	1	4	0
0	0	1	10	1
0	0	1	4	0
0	0	1	0	0
0	0	8	4	0
0	0	1	8	0
0	0	1	6	0

0	0	1	6	1
0	0	1	0	0
0	0	3	3	0
0	0	1	2	0
0	0	1	5	0
0	0	1	0	0
1	1	8	0	0
1	1	1	8	0
1	0	1	0	0

1	0	4	10	0
1	0	1	2	0
1	0	1	3	0
1	0	1	13	0
0	0	1	11	0
1	0	1	7	0
0	0	1	2	0
0	0	1	3	0
0	0	1	6	1

0	0	1	6	0
0	0	1	4	0
0	0	1	0	0
0	1	1	1	1
0	0	1	6	0
0	0	1	11	1
0	0	0	1	0
0	0	1	4	0
0	0	1	2	0

1	1	4	0	0
0	0	2	1	0
0	0	1	10	0
0	1	1	0	1
1	1	1	8	0
1	1	1	3	0
0	0	1	1	0
0	1	4	11	0
0	1	1	6	0

0	0	1	0	0
0	0	1	3	0
1	0	1	6	0
0	1	45	3	0
0	0	1	3	1
0	1	1	1	1
0	0	1	2	0
0	0	1	14	0
1	1	1	3	1

0	0	0	1	0
1	1	2	1	1

AlgunavezenlavidaInsultado	AlgunavezenlavidaHumillado	AlgunavezenlavidaDescalificado	AlgunavezenlavidaAmenazadoconhacerledañoaud	AlgunavezenlavidaHaamenazadoaalguienqueudquiere
0	0	0	0	0
0	0	0	0	0
0	0	0	0	0
0	0	0	0	0
0	0	0	0	0
0	0	0	0	0
0	0	0	0	0
0	0	0	0	0

0	0	0	0	0
0	0	0	0	0
0	0	0	0	0
0	0	0	0	0
0	0	0	0	0
1	1	1	1	0
0	0	0	0	0
0	0	0	0	0
0	0	0	0	0

0	0	0	0	0
0	0	0	0	0
0	0	0	0	0
0	0	0	0	0
0	0	0	0	0
2	2	2	2	2
2	2	2	0	0
0	0	0	0	0
0	0	0	0	0

2	2	2	0	0
2	2	2	2	2
2	2	2	0	0
2	2	2	2	2
0	0	0	0	0
2	1	0	2	2
0	0	0	0	0
2	2	2	0	0
0	0	0	0	0

0	0	0	0	0
0	0	0	0	0
2	2	2	0	0
0	0	0	0	0
2	2	2	0	0
0	0	0	0	0
2	2	2	2	0
0	0	0	0	0
0	0	0	0	0

2	2	2	1	1
1	1	0	0	0
2	2	2	0	0
2	2	2	2	2
2	2	1	1	2
1	1	1	0	0
0	0	0	0	0
0	0	0	0	0
0	0	0	0	0

0	0	0	0	0
0	0	0	0	0
0	0	0	0	0
0	0	0	0	0
0	0	0	0	0
0	0	0	0	0
0	2	0	0	0
2	2	2	2	0
0	0	0	0	0

2	2	2	2	2
2	2	2	0	0
2	2	2	2	0
0	0	0	0	0
2	2	0	0	0
2	2	2	1	0
0	1	2	0	0
2	2	2	2	2
0	0	0	0	0

2	2	2	2	2
2	2	2	2	2
0	0	0	0	0
2	2	2	2	2
2	2	2	0	0
2	2	2	2	0
0	0	0	0	0
0	0	0	0	0
2	2	2	0	0

0	0	0	0	0
1	1	1	1	1
1	0	0	0	0
0	0	0	0	0
0	1	1	0	0
2	2	2	0	0
2	2	2	0	0
2	2	2	2	2
2	2	2	2	0

2	2	1	0	0
0	0	2	2	2
2	2	2	0	0
1	0	0	0	0
1	1	1	0	0
2	1	1	0	0
1	1	1	0	0
2	0	2	0	0
2	2	2	2	2

2	2	2	2	0
2	2	2	2	0
0	0	0	0	0
0	0	0	0	0
2	2	2	2	2
2	2	2	2	2
2	2	2	2	0
0	0	0	0	0
2	2	2	2	2

2	2	2	2	2
1	0	0	0	0
0	1	0	0	0
2	2	2	2	2
2	2	2	2	0
2	2	2	2	2
1	1	1	1	0
2	2	2	0	0
2	2	2	0	0

0	0	0	0	0
0	0	0	0	0
2	2	2	0	0
0	0	0	0	0
0	0	0	0	0
2	2	2	2	2
2	2	2	0	0
0	0	0	0	0
0	0	0	0	0

2	2	2	2	0
2	2	2	2	2
2	2	2	1	0
2	2	2	2	0
0	0	0	0	0
0	0	0	2	0
0	0	0	0	0
0	0	0	0	0
0	0	0	0	0

2	2	2	2	0
1	1	1	1	0
2	2	2	0	0
2	2	2	2	2
0	0	0	0	0
0	0	0	0	0
1	1	1	1	0
0	0	0	0	0
0	0	0	0	0

1	1	2	0	0
2	2	2	2	0
0	0	0	0	0
2	2	2	2	0
0	0	0	0	0
2	2	2	2	1
2	2	2	0	0
2	2	2	2	2
2	2	2	2	0

2	2	2	0	0
2	2	2	0	0
0	0	0	0	0
2	0	2	0	0
1	0	0	2	0
0	0	0	0	0
0	0	0	0	0
0	0	0	0	0
0	0	0	0	0

2	2	2	1	0
0	0	0	0	0
1	1	0	0	0
2	2	2	0	0
2	2	2	2	0
2	2	2	0	0
2	2	1	0	1
0	0	0	0	0
0	0	0	0	1

0	0	0	0	0
0	0	0	0	0
0	2	0	0	0
0	0	0	0	0
0	0	0	0	0
1	0	0	0	0
0	0	2	0	0
0	2	2	0	0
0	0	0	0	0

2	2	2	2	2
2	2	1	1	0
1	1	1	0	0
2	1	0	0	0
2	2	2	2	0
1	0	0	0	0
0	0	0	0	0
0	0	0	0	0
2	2	2	2	2

1	0	0	1	0
0	0	0	0	0
2	2	2	2	2
2	2	2	2	2
0	0	0	0	0
2	2	2	2	0
0	0	0	0	0
2	2	2	2	2
2	2	2	2	2

2	2	2	0	0
0	0	0	0	0
2	2	2	2	0
1	1	0	0	1
2	2	2	2	0
0	0	0	0	0
0	0	0	0	0
0	0	0	0	0
2	2	2	0	0

0	0	0	0	0
0	0	0	0	0
2	2	2	2	1
0	0	0	0	0
2	2	2	2	2
2	2	2	2	1
0	0	0	0	0
0	0	0	0	0
2	2	2	0	0

2	2	2	2	0
2	2	2	2	0
0	0	0	0	0
1	1	1	1	0
2	2	2	2	2
0	0	0	0	0
2	2	2	2	2
2	2	2	2	0
2	2	2	0	0

0	0	0	0	0
2	2	2	2	2
2	2	2	2	0
0	0	0	0	0
0	0	1	0	0
2	2	2	2	2
0	0	0	0	0
2	0	0	0	0
2	2	2	0	0

2	2	2	2	0
2	2	2	2	0
2	1	0	0	0
0	0	0	0	0
2	2	2	0	0
2	2	2	1	0
2	1	0	0	0
2	2	2	2	0
2	2	2	1	1

2	2	2	2	0
0	0	0	0	0
2	2	2	0	0
2	0	2	2	2
1	1	1	0	0
2	2	2	2	1
0	0	0	0	0
0	0	0	0	0
2	2	2	0	0

1	0	0	0	0
1	0	0	1	0
2	2	2	2	1
2	0	0	0	0
2	0	2	0	0
0	2	2	0	0
2	2	2	2	2
2	2	2	2	0
0	0	0	0	0

2	2	2	2	2
2	2	2	0	0
0	0	0	0	0
0	0	2	0	0
2	2	2	2	2
2	0	0	0	0
0	0	0	1	1
0	0	0	0	0
2	2	2	2	2

1	0	0	0	0
2	1	2	2	0
0	0	0	0	0
0	0	0	0	0
0	0	1	0	0
2	2	2	2	2
2	2	2	2	0
1	1	1	0	0
2	2	2	2	0

2	2	2	0	1
0	0	0	0	0
0	0	0	0	0
2	2	2	1	0
2	2	2	2	0
0	0	0	0	0
2	2	2	2	0
2	2	2	0	0
0	0	0	0	0

2	2	2	2	0
2	2	2	2	0
2	2	2	1	0
0	0	1	0	0
1	0	1	0	0
2	2	2	0	0
2	2	2	0	0
0	0	0	0	0
2	0	1	0	1

1	1	1	0	0
2	2	2	2	1
2	2	2	1	1
2	2	2	2	2
2	2	0	0	0
0	2	0	0	0
0	0	0	0	0
2	2	2	2	1
2	2	2	0	0

2	2	2	0	0
0	0	0	0	0
0	0	0	0	0
2	2	2	0	0
0	0	0	0	0
2	2	2	2	0
0	0	0	0	0
0	0	0	0	0
2	2	2	2	0

2	0	0	0	0
2	2	2	1	1
2	2	2	2	2
2	2	2	0	0
0	0	0	0	0
2	2	2	2	0
2	2	2	2	0
0	0	0	0	0
2	2	2	2	2

0	0	0	0	0
2	2	2	2	0
2	2	2	2	2
0	0	0	0	0
0	0	0	0	0
2	2	2	0	0
2	2	2	2	2
0	0	0	0	0
0	0	0	0	0

2	2	2	0	0
1	1	1	0	0
2	2	2	0	2
0	0	0	0	0
2	2	2	2	2
2	2	2	0	0
0	0	0	0	0
2	2	2	2	2
0	0	0	0	0

0	0	0	0	0
0	0	0	0	0
1	1	1	0	0
0	0	0	0	0
2	2	2	0	0
0	0	0	0	0
2	2	2	2	0
0	0	0	0	0
0	0	0	0	0

0	0	0	0	0
0	0	0	0	0
2	2	2	2	0
0	0	0	0	0
1	1	1	0	0
0	1	2	0	0
0	0	0	0	0
0	1	0	0	0
2	1	1	0	2

0	0	0	0	0
2	2	2	2	0
0	0	0	0	0
2	2	2	0	0
2	2	2	0	0
2	2	2	0	0
0	0	0	0	0
1	2	0	1	0
2	2	2	1	0

2	0	0	0	0
1	0	1	0	0
0	0	0	0	0
2	2	2	2	0
2	2	2	2	2
2	2	2	0	0
0	0	0	0	0
0	0	0	0	0
0	2	1	0	0

0	2	2	0	0
0	2	2	0	0
0	0	0	0	0
0	0	0	0	0
0	0	2	0	0
2	2	2	2	2
1	1	1	0	0
2	2	2	0	0
1	1	1	1	1

2	2	2	2	0
2	2	2	0	0
0	0	1	0	0
0	0	0	0	0
1	1	1	0	1
0	0	0	0	0
2	2	2	2	2
2	2	2	0	0
2	2	2	1	1

0	0	0	0	0
0	0	0	0	0
2	2	2	2	0
2	2	2	0	0
1	1	1	0	0
0	0	0	0	0
0	0	0	0	0
2	2	2	2	0
1	0	0	0	0

0	0	0	0	0
0	0	0	0	0
0	0	0	0	0
2	2	2	2	2
0	0	0	0	0
0	0	0	0	0
0	0	0	0	0
1	1	1	0	0
1	1	1	0	0

2	0	1	0	0
1	0	0	0	0
0	0	0	0	0
2	2	2	2	2
2	2	2	2	0
0	0	0	0	0
2	2	2	2	0
0	0	0	0	0
0	0	0	0	0

2	2	2	2	2
2	2	2	1	0
1	2	2	0	0
2	2	2	2	2
2	2	2	0	0
0	0	0	0	0
0	0	0	0	0
2	2	2	0	0
0	0	0	0	0

2	2	2	2	2
2	2	2	0	0
2	2	2	0	0
2	2	2	2	2
1	0	0	0	0
2	2	2	2	2
0	0	0	0	0
2	2	2	2	2
1	1	1	1	1

2	2	0	2	2
2	2	2	2	2

AlgunavezenlavidaLehahechoalgoquelaatemorizosinto	AlgunavezenlavidaLahadejadoseismesessinapoyoeconó	AlgunavezenlavidaLehadadobofetada	AlgunavezenlavidaLahapateado	AlgunavezenlavidaLehadadopuñetazos
0	0	0	0	0
0	0	0	0	0
0	0	0	0	0
0	0	0	0	0
0	0	0	0	0
0	0	0	0	0
0	0	0	0	0
0	0	0	0	0

0	0	0	0	0
0	0	0	0	0
0	0	0	0	0
0	0	0	0	0
0	0	0	0	0
1	0	1	0	0
0	0	0	0	0
0	0	0	0	0
0	0	0	0	0

0	0	0	0	0
0	0	0	0	0
0	0	0	0	0
0	0	0	0	0
0	0	0	0	0
2	2	2	2	2
0	0	1	0	0
0	0	0	0	0
0	0	0	0	0

0	0	0	0	0
2	2	2	2	2
0	0	1	0	1
2	2	2	2	2
0	0	0	0	0
0	2	2	2	2
0	0	0	0	0
2	2	1	0	2
0	0	0	0	0

0	0	0	0	0
0	2	0	0	0
2	2	2	0	0
0	2	0	0	0
0	0	1	0	0
0	0	0	0	0
2	0	2	1	2
0	0	0	0	0
0	0	0	0	0

1	2	1	0	0
0	0	0	0	0
0	2	1	0	1
2	2	2	2	2
2	0	1	0	0
0	2	0	0	0
0	0	0	0	0
0	0	0	0	0
0	0	0	0	0

0	0	0	0	0
0	0	0	0	0
0	0	0	0	0
0	0	0	0	0
0	0	0	0	0
0	0	0	0	0
0	0	0	0	0
2	2	2	0	2
0	2	0	0	0

2	0	2	2	2
0	0	0	0	0
2	2	2	2	2
0	0	0	0	0
2	0	0	0	0
1	2	1	1	0
0	0	0	0	0
1	2	2	0	2
0	0	0	0	0

2	0	2	2	2
2	2	2	2	2
0	0	0	0	0
2	0	2	0	2
0	0	0	0	0
2	2	2	2	2
0	0	0	0	0
0	0	0	0	0
1	0	1	0	0

0	0	0	0	0
1	0	1	1	1
0	0	0	0	0
0	0	0	0	0
0	0	0	0	0
2	1	0	0	0
0	2	1	0	0
2	2	2	2	2
2	2	2	0	0

0	0	0	0	0
0	0	0	0	0
2	0	1	0	0
1	0	0	0	0
0	0	0	0	0
0	0	2	0	0
0	0	1	0	0
0	0	0	0	0
0	0	2	2	2

2	0	0	0	0
0	0	2	2	2
0	0	0	0	0
0	0	0	0	0
2	1	2	2	2
2	2	2	0	0
2	2	2	0	0
0	0	0	0	0
2	2	2	2	2

2	2	2	2	1
0	0	0	0	0
0	0	0	0	0
1	2	2	2	2
1	1	2	2	2
2	2	0	0	0
0	0	0	0	0
2	0	0	0	0
0	0	1	1	1

0	0	0	0	0
0	0	0	0	0
0	0	0	0	0
0	0	0	0	0
0	2	1	0	0
2	2	2	0	0
0	0	0	0	0
0	0	0	0	0
0	0	0	0	0

2	2	2	0	0
1	0	0	0	0
0	0	2	2	0
2	2	2	0	0
0	0	0	0	0
1	0	0	0	0
0	0	0	0	0
0	0	0	0	0
0	0	0	0	0

0	1	2	2	2
0	0	1	0	0
0	0	0	0	0
2	2	2	2	2
0	0	0	0	0
0	0	0	0	0
0	0	0	0	0
0	0	0	0	0
0	0	0	0	0

1	0	0	0	0
2	0	2	2	2
0	0	0	0	0
2	0	2	0	0
0	0	0	0	0
2	0	2	0	2
0	0	0	0	0
2	0	2	1	1
1	2	0	0	0

1	0	2	2	2
0	0	0	0	0
0	2	1	0	0
2	0	1	0	0
1	2	0	0	0
0	0	0	0	0
0	0	0	0	0
0	0	0	0	0
0	0	0	0	0

2	1	2	0	1
0	0	0	0	0
0	2	0	0	0
1	1	2	2	2
2	0	1	1	0
2	0	1	0	0
1	0	1	1	0
0	2	0	0	0
0	0	0	0	0

0	0	0	0	0
0	0	0	0	0
1	2	0	0	0
0	0	0	0	0
0	0	0	0	0
0	0	0	0	0
0	0	0	0	0
0	0	0	0	0
0	0	0	0	0

2	0	2	2	2
2	0	0	0	1
0	0	1	0	1
1	0	1	0	0
2	0	1	0	0
0	0	0	0	0
0	0	0	0	0
0	0	0	0	0
2	0	2	2	2

1	0	1	0	0
0	0	0	0	0
2	2	2	2	2
2	2	2	2	2
0	0	0	0	0
1	2	2	0	0
0	0	0	0	0
2	2	2	2	2
2	0	0	0	0

2	1	0	0	0
0	0	0	0	0
2	2	2	2	2
0	0	0	0	0
0	0	2	1	2
0	0	0	0	0
0	0	0	0	0
0	0	0	0	0
0	0	0	0	0

0	0	0	0	0
0	0	0	0	0
1	0	0	0	0
0	0	0	0	0
0	0	1	2	2
0	2	2	2	2
0	0	0	0	0
0	0	0	0	0
0	0	0	0	0

2	0	0	2	2
0	0	1	0	0
0	0	0	0	0
0	0	0	0	0
2	2	2	2	2
0	0	0	0	0
0	2	2	2	2
0	0	0	0	0
2	0	2	0	1

0	0	0	0	0
2	0	2	2	2
0	2	2	2	2
0	0	0	0	0
1	0	0	0	0
2	1	1	1	0
0	0	0	0	0
0	0	1	1	1
2	0	2	2	0

2	0	2	0	0
2	0	2	0	2
1	0	0	0	0
2	0	0	0	0
2	0	2	2	2
2	2	2	2	2
1	0	1	0	0
2	0	2	2	2
1	2	2	0	0

2	0	2	2	2
0	0	0	0	0
0	0	0	0	0
2	0	2	0	2
1	0	1	0	0
1	2	1	1	1
0	0	0	0	0
0	0	0	0	0
0	0	2	1	0

0	0	0	0	0
0	0	1	1	1
2	2	2	0	1
0	0	0	0	0
1	0	0	0	0
0	0	0	0	0
2	0	2	0	2
2	2	1	0	1
0	0	0	0	0

0	0	0	1	1
1	2	0	0	0
0	0	0	0	0
0	0	0	0	0
2	2	2	1	2
0	0	0	0	0
1	0	0	0	0
0	0	0	0	0
2	2	2	0	0

0	0	0	0	0
2	2	2	0	2
0	0	0	0	0
0	0	0	0	0
0	0	2	2	2
2	1	2	2	2
2	0	2	2	2
0	0	0	0	0
2	0	2	2	2

2	0	0	0	0
0	0	0	0	0
0	0	0	0	0
2	2	0	0	1
2	2	2	2	2
0	0	2	0	0
2	0	0	1	1
0	2	2	0	1
0	0	0	0	0

0	2	0	0	0
2	0	2	0	0
1	0	0	0	0
0	0	0	0	0
0	0	0	0	0
0	0	0	0	0
0	0	0	0	1
0	0	0	0	0
1	0	1	1	0

0	0	0	0	0
2	2	1	0	1
1	0	0	0	0
2	2	2	2	2
2	0	1	0	0
0	0	0	0	0
0	0	2	0	2
0	2	2	1	1
2	2	0	0	2

0	2	0	0	2
0	0	0	0	0
0	0	0	0	0
0	0	0	0	0
0	0	0	0	0
0	0	2	1	2
0	0	0	0	0
0	0	0	0	0
2	0	2	0	0

0	0	0	0	0
1	0	0	0	0
2	0	2	0	2
0	0	1	0	0
0	0	0	0	0
0	1	2	0	2
0	2	2	2	2
0	0	0	0	0
2	0	2	2	2

2	0	1	0	0
2	0	2	0	2
0	2	0	0	0
0	0	0	0	0
0	2	1	1	1
0	0	0	0	0
2	2	1	1	1
0	0	0	0	0
0	0	0	0	0

0	0	0	0	0
0	0	0	0	0
2	2	0	0	0
0	0	0	0	0
2	2	2	2	2
0	0	0	0	0
0	0	0	0	0
2	2	2	2	2
0	0	0	0	0

0	0	0	0	0
0	0	0	0	0
1	0	1	0	0
0	0	0	0	0
0	0	0	0	0
0	0	0	0	0
0	1	2	2	2
0	0	0	0	0
0	0	0	0	0

0	0	0	0	0
0	0	0	0	0
2	2	2	2	2
0	0	0	0	0
0	0	0	0	0
1	2	0	0	0
0	0	0	0	0
0	0	1	0	0
2	2	1	0	0

0	0	0	0	0
0	0	2	0	0
0	0	0	0	0
0	0	0	0	0
1	0	1	0	0
0	0	2	2	2
0	0	0	0	0
0	2	0	0	0
0	2	0	1	0

2	0	1	0	0
0	0	0	0	0
0	0	0	0	0
1	0	0	0	0
2	2	2	2	2
0	0	1	0	0
0	0	0	0	0
0	0	0	0	0
0	0	0	0	0

0	0	0	1	1
0	0	0	0	0
0	0	0	0	0
0	0	0	0	0
0	0	1	0	0
2	2	2	2	2
0	0	0	0	0
0	0	0	0	0
1	2	1	0	1

2	2	2	2	2
0	0	2	2	2
0	0	0	0	0
0	0	0	0	0
1	0	0	0	0
0	0	0	0	0
2	0	2	2	2
0	0	0	0	0
2	0	1	0	0

0	0	0	0	0
0	0	0	0	0
2	0	2	0	0
0	0	0	0	0
0	0	1	0	0
0	0	0	0	0
0	0	0	0	0
2	0	2	2	1
0	0	0	0	0

0	0	0	0	0
0	0	0	0	0
0	0	0	0	0
2	0	2	0	0
0	0	0	0	0
0	0	0	0	0
0	0	0	0	0
0	0	0	0	0
0	0	0	0	0

0	0	0	0	0
2	0	0	0	0
0	0	0	0	0
2	2	2	0	2
0	0	1	0	0
0	0	0	0	0
0	0	2	2	2
0	0	0	0	0
0	0	0	0	0

2	2	2	1	2
1	2	1	1	1
0	0	1	0	0
0	2	0	0	0
2	0	0	0	0
0	0	0	0	0
0	0	0	0	0
0	0	0	0	0
0	0	0	0	0

2	0	2	0	0
0	0	0	2	0
0	0	0	0	0
2	0	2	0	0
0	0	0	0	0
2	0	2	2	2
0	0	0	0	0
2	2	2	2	2
1	1	1	1	0

1	2	0	0	0
2	2	2	2	2

AlgunavezenlavidaLehadadounapaliza	AlgunavezenlavidaLahaforzadoatenerrelacionessexuale	DEPRESION	filter_$	hamilton_total
0	0	1	1	24
0	0	1	1	26
0	0	1	1	13
0	0	1	1	28
0	0	1	1	18
0	0	1	1	27
0	0	0	0	20
0	0	1	1	25

0	0	1	1	16
0	0	1	1	20
0	0	1	1	20
0	0	1	1	18
0	0	1	1	16
0	0	1	1	14
0	0	0	0	25
0	0	1	1	16
0	0	1	1	17

0	0	1	1	21
0	0	1	1	21
0	0	0	0	10
0	0	1	1	25
0	0	1	1	20
2	0	1	1	5
0	0	1	1	21
0	0	0	0	11
0	0	1	1	11

0	0	1	1	33
2	2	1	1	27
1	0	1	1	15
2	2	1	1	31
0	0	1	1	19
2	2	1	1	14
0	0	1	1	17
2	0	1	1	14
0	0	1	1	20

0	0	1	1	8
0	0	1	1	14
0	0	1	1	16
0	0	1	1	17
0	0	1	1	12
0	0	1	1	14
1	0	1	1	20
0	0	0	0	14
0	0	1	1	20

0	0	1	1	29
0	0	1	1	17
0	1	1	1	27
2	2	1	1	23
0	0	1	1	17
0	0	1	1	23
0	0	1	1	20
0	0	1	1	20
0	0	1	1	20

0	0	1	1	20
0	0	1	1	20
0	0	0	0	20
0	0	0	0	20
0	0	1	1	20
0	0	1	1	20
0	0	1	1	20
2	0	0	0	20
0	0	1	1	20

0	2	0	0	20
0	0	1	1	7
0	2	1	1	19
0	0	1	1	8
0	0	1	1	18
1	1	1	1	17
0	0	1	1	11
0	2	1	1	14
0	0	1	1	17

2	2	0	0	12
2	2	1	1	10
0	0	1	1	29
0	0	1	1	19
0	2	1	1	17
2	2	1	1	26
0	0	1	1	22
0	0	1	1	21
0	2	1	1	20

0	0	0	0	20
0	1	1	1	25
0	2	1	1	23
0	0	1	1	9
0	0	1	1	25
0	0	1	1	27
0	0	1	1	15
2	2	1	1	25
0	2	1	1	28

0	0	1	1	18
0	2	1	1	33
0	0	1	1	29
0	0	1	1	23
0	0	1	1	29
0	0	1	1	26
0	0	1	1	35
0	0	1	1	20
0	0	1	1	33

1	1	1	1	23
2	2	1	1	30
0	0	1	1	19
0	0	1	1	12
0	2	1	1	25
0	2	1	1	23
0	0	0	0	12
0	0	1	1	15
2	2	1	1	34

0	0	1	1	6
0	0	0	0	7
0	0	1	1	18
1	0	1	1	24
2	0	0	0	15
0	0	1	1	25
1	0	1	1	11
0	0	1	1	30
1	0	1	1	29

0	0	1	1	24
0	0	1	1	21
0	0	1	1	28
0	0	1	1	24
0	1	1	1	19
0	2	1	1	14
0	0	1	1	25
0	0	1	1	16
0	0	1	1	15

0	2	1	1	27
0	0	1	1	30
0	0	1	1	15
0	0	1	1	32
0	0	1	1	22
0	0	1	1	23
0	0	1	1	19
0	0	1	1	22
0	0	1	1	12

2	2	1	1	19
0	0	1	1	22
0	0	1	1	15
0	2	1	1	25
0	0	1	1	24
0	0	1	1	17
0	0	0	0	9
0	0	1	1	10
0	0	1	1	15

0	0	1	1	23
1	0	1	1	21
0	0	1	1	25
0	0	0	0	12
0	0	0	0	11
0	2	1	1	18
0	0	1	1	21
0	1	1	1	22
0	0	1	1	19

2	2	1	1	15
0	0	0	0	24
0	0	1	1	13
0	0	0	0	13
0	0	1	1	22
0	0	1	1	20
0	0	0	0	25
0	0	1	1	18
0	0	1	1	31

0	0	1	1	27
0	0	1	1	22
0	0	1	1	24
2	2	1	1	26
0	1	1	1	26
0	0	1	1	23
0	0	1	1	24
0	0	1	1	19
0	0	1	1	22

0	0	1	1	18
0	0	1	1	17
0	1	1	1	28
0	0	1	1	21
0	0	1	1	24
0	0	1	1	20
0	0	1	1	22
0	0	1	1	18
0	0	1	1	19

2	0	1	1	29
0	0	1	1	18
0	0	0	0	10
0	0	1	1	23
0	0	0	0	10
0	0	1	1	18
0	0	1	1	16
0	0	1	1	29
2	2	1	1	14

0	0	1	1	18
0	0	1	1	22
2	2	1	1	14
2	2	1	1	16
0	0	1	1	18
0	2	1	1	14
0	0	1	1	16
2	2	1	1	23
0	1	1	1	23

0	0	1	1	33
0	0	1	1	21
2	0	1	1	16
0	0	0	0	10
2	0	1	1	12
0	0	1	1	15
0	0	0	0	7
0	0	1	1	27
0	0	1	1	12

0	0	0	0	4
0	0	0	0	11
0	0	1	1	17
0	0	0	0	7
2	0	1	1	26
2	2	1	1	26
0	0	1	1	34
0	0	1	1	25
0	0	1	1	19

2	0	1	1	11
0	0	0	0	7
0	0	1	1	32
0	0	1	1	30
2	0	1	1	23
0	0	1	1	20
2	2	1	1	16
1	0	1	1	22
1	2	1	1	27

0	0	1	1	16
2	2	1	1	23
2	2	1	1	17
0	0	1	1	17
0	0	1	1	21
0	0	1	1	15
0	0	1	1	24
1	0	1	1	27
0	0	1	1	20

0	0	1	1	15
0	0	1	1	22
0	0	1	1	16
0	0	1	1	18
0	0	1	1	25
1	0	1	1	25
0	0	1	1	17
0	1	1	1	20
0	0	1	1	15

0	2	1	1	21
0	0	1	1	8
0	0	1	1	24
0	2	1	1	29
0	0	1	1	14
0	0	1	1	18
0	0	1	1	19
0	0	1	1	16
1	1	1	1	24

0	0	1	1	16
1	0	1	1	28
0	1	1	1	28
0	0	1	1	17
0	0	1	1	11
0	0	1	1	30
0	2	1	1	25
0	0	1	1	20
0	0	1	1	16

0	0	1	1	7
0	0	1	1	34
0	0	1	1	32
0	0	1	1	26
2	2	1	1	17
0	0	1	1	23
0	0	1	1	26
0	0	1	1	31
2	2	1	1	24

0	0	1	1	30
2	0	1	1	27
0	0	0	0	10
0	0	1	1	14
0	0	1	1	18
2	0	1	1	25
2	2	1	1	14
0	0	1	1	23
2	0	1	1	18

0	2	1	1	21
0	0	1	1	17
0	0	0	0	15
0	0	1	1	11
0	0	1	1	11
0	0	1	1	19
1	0	1	1	19
0	0	1	1	20
0	0	0	0	2

0	0	1	1	28
0	0	1	1	24
0	0	1	1	28
0	0	1	1	26
0	0	1	1	24
0	0	1	1	19
0	0	1	1	28
0	0	1	1	16
0	1	1	1	20

0	0	1	1	20
1	2	1	1	30
0	0	1	1	16
2	2	1	1	28
1	0	1	1	18
0	0	1	1	15
0	0	1	1	20
0	1	1	1	25
0	0	1	1	23

2	2	1	1	20
0	0	1	1	13
0	0	1	1	20
0	0	1	1	14
0	0	1	1	16
1	0	1	1	25
0	0	1	1	19
0	0	1	1	17
0	0	0	0	15

0	0	1	1	21
0	0	1	1	19
2	0	1	1	19
0	0	1	1	20
0	0	1	1	11
0	0	1	1	25
2	2	1	1	29
0	0	1	1	12
0	2	1	1	22

0	2	1	1	16
0	1	1	1	17
0	0	1	1	21
0	0	0	0	5
1	0	1	1	20
0	0	1	1	34
1	1	1	1	20
0	0	1	1	29
0	0	1	1	21

0	0	1	1	19
0	0	1	1	26
0	0	1	1	19
0	0	1	1	29
2	2	1	1	29
0	0	1	1	31
0	0	1	1	22
2	2	1	1	28
0	0	1	1	14

0	0	1	1	22
0	0	1	1	20
0	1	1	1	4
0	0	1	1	24
0	0	1	1	30
0	0	1	1	32
2	0	1	1	32
0	0	1	1	25
0	0	0	0	35

0	0	0	0	16
0	0	0	0	9
2	2	1	1	28
0	0	1	1	32
1	0	0	0	9
0	0	0	0	13
0	0	0	0	9
0	0	1	1	26
0	0	1	1	18

0	0	1	1	19
0	0	1	1	9
0	0	1	1	15
0	0	1	1	22
0	0	0	0	12
0	0	1	1	17
0	0	1	1	19
0	0	1	1	24
0	0	1	1	18

0	0	1	1	15
0	0	1	1	22
0	0	1	1	17
0	0	1	1	28
2	2	1	1	27
0	0	1	1	35
0	0	0	0	12
0	0	1	1	7
0	0	1	1	27

0	0	1	1	24
0	0	1	1	23
0	0	1	1	20
0	0	1	1	22
0	0	1	1	21
1	2	1	1	31
0	0	0	0	8
0	0	1	1	21
0	1	1	1	24

0	0	1	1	21
0	0	0	0	15
0	0	1	1	29
0	0	1	1	21
0	0	1	1	30
0	0	1	1	16
2	2	1	1	21
0	0	1	1	26
0	0	1	1	15

0	0	1	1	30
0	0	1	1	13
0	0	1	1	15
0	0	1	1	24
0	0	1	1	16
0	0	1	1	8
0	0	1	1	23
0	0	1	1	23
0	0	1	1	22

0	0	1	1	24
0	0	1	1	20
0	0	1	1	9
0	0	1	1	24
0	0	1	1	14
0	0	1	1	9
0	0	1	1	15
0	0	1	1	10
0	0	1	1	19

0	0	1	1	19
0	0	1	1	18
0	0	0	0	1
1	0	1	1	21
0	0	1	1	16
0	0	0	0	18
2	2	1	1	12
0	0	1	1	18
0	0	1	1	21

2	2	1	1	23
1	0	1	1	25
0	0	1	1	12
0	2	1	1	19
0	0	1	1	27
0	0	1	1	25
0	0	1	1	17
0	0	1	1	9
0	0	1	1	16

0	0	1	1	7
0	0	1	1	20
0	0	1	1	11
0	0	0	0	28
0	0	1	1	28
0	2	1	1	23
0	0	1	1	13
2	2	1	1	22
0	1	1	1	20

0	0	0	0	20
2	2	0	0	20

isdc30_total	TOTAL_MARSHALL	maltrato_infantil	SumaOQ45	ASISTENCIAPRIMARIASALUDALQUEINGRESA
42	0	0	77	central
45	4	4	88	central
25	1	1	92	central
39	1	1	105	Colon
36	5	5	91	central
36	2	2	64	central
29	3	3	0	central
37	0	0	89	central

32	3	3	104	central
31	3	3	93	Curicó
24	4	4	94	Curicó
30	2	2	0	CESFAM Sol de Sept ; Curicó
21	1	1	0	Cesfam s Sept Curicó
15	0	0	0	Cesfam Colón Curicó
31	0	0	95	central
26	3	3	87	central
43	2	2	80	central

41	7	7	97 central
47	1	1	94 central
11	1	1	95 central
53	0	0	99 central
32	1	1	87 central
4	2	2	82 central
33	1	1	87 central
15	0	0	63 central
19	2	2	0 central

47	2	2	94	central
36	7	7	106	central
22	0	0	75	central
46	5	5	86	central
39	4	4	84	cesfam colon
30	6	6	89	cesfam miguel angel arenas lopez
32	0	0	94	cesfam miguel arenas lopez
28	5	5	98	cesfam miguel arenas lopez
35	2	2	89	cesfam sol de septiembre

15	0	0	98	cesfam miguel arenas lopez
26	4	4	89	cesfam central
24	5	5	117	cesfam muguel a arenas lopez
36	7	7	96	cesfam central
20	5	5	83	cesfam miguel arenas lopez
17	3	3	96	sol de sptiembre, Curicó
26	3	3	0	Cesfam Colón, Curicó
14	2	2	0	Cesfam Colón, Curicó
32	2	2	0	cesfam sol de spt, Curicó

29	0	0	0	cesfam sol de septiembre, Curicó
25	1	1	0	cesfam sol de sept, Curicó
41	3	3	87	cesfam Los Rromos Curico
29	7	7	0	cesfam sol de septiembre ; Curicó
24	6	6	100	cesfam sol de septiembre, Curicó
38	1	1	95	cesfam sol de septiembre, Curicó
53	1	1	83	CESFAM LAS AMÉRICAS
34	5	5	103	CESFAM LAS AMÉRICAS
37	3	3	101	CESFAM LAS AMÉRICAS

37	5	5	93	cesfam Las Américas
38	6	6	82	CESFAM LAS AMÉRICAS
14	1	1	88	CESFAM LAS AMÉRICAS
12	0	0	0	CESFAM LA FLORIDA
42	0	0	95	CESFAM LAS AMERICAS
28	3	3	88	CESFAM LAS AMERICAS
14	3	3	82	LA FLORIDA
15	2	2	0	CESFAM LA FLORIDA
46	1	1	104	CESFAM LA FLORIDA

28	4	4	0	CESFAM LA FLORIDA
14	6	6	74	LA FLORIDA
26	3	3	0	cesfam Julio Contardo
21	0	0	72	LA FLORIDA
25	0	0	86	La Florida
35	2	2	107	Norte
18	1	1	100	NORTE
30	5	5	123	norte
30	0	0	74	Faustino González

25	4	4	89	Faustino González
23	5	5	0	Faustino González
51	4	4	0	Faustino González
33	3	3	67	FAUSTINO GONZÁLEZ
35	5	5	74	FAUSTINO GONZÁLEZ
64	6	6	116	FAUSTINO GONZÁLEZ
56	0	0	116	Carlos Trupp
35	3	3	68	Carlos Trupp
39	1	1	0	Carlos Trupp

35	3	3	89	cesfam Julio Contardo
38	5	5	93	CESFAM JULIO CONTARDO
42	4	4	0	cesfam Julio Contardo
16	2	2	55	CESFAM JULIO CONTARDO
33	3	3	110	CESFAM JULIO CONTARDO
37	4	4	0	CESFAM JULIO CONTARDO
17	1	1	0	cesfam julio contardo
41	4	4	92	cesfam Las Américas
42	5	5	99	cesfam Las Américas

30	0	0	0	cesfam Las américas
49	0	0	94	La Florida
41	2	2	100	LA FLORIDA
41	2	2	93	LA FLORIDA
46	2	2	105	LA FLORIDA
35	3	3	89	La Florida
50	2	2	93	La Florida
41	3	3	93	La Florida
52	0	0	98	La Florida

41	1	1	95	La Florida
46	3	3	108	La Florida
39	0	0	0	sarmiento
18	2	2	0	sarmieto
52	2	2	120	sarmiento (doña carmen)
47	6	6	120	sarmiento (doña carmen)
16	0	0	0	sarmiento
21	0	0	0	sarmiento
68	6	6	98	sarmiento, dona carmen

4	3	3	76	sarmiento (doña carmen)
12	2	2	89	sarmiento (doña carmen)
42	1	1	97	sarmiento
38	2	2	125	central
22	3	3	0	central
48	4	4	84	central
18	4	4	66	central
41	0	0	106	central
35	6	6	89	central

37	0	0	95	central
27	0	0	90	central
43	1	1	0	central portador de polio
33	3	3	110	central
30	3	3	81	central
23	4	4	73	central
41	5	5	80	central
21	1	1	89	central
28	0	0	83	central

37	3	3	92	central
48	4	4	0	central
27	3	3	90	central
54	1	1	92	central vif `por parte del padre
26	4	4	100	central
38	5	5	120	CESFAM MIGUEL ARENAS LOPEZ
29	2	2	78	cesfam curico centro
47	0	0	118	cesfam miguel arenas lopez
21	2	2	86	cesfam miguel arenas lopez

26	4	4	88	cesfam miguel arenas lopez
40	4	4	96	cesfam curico centro
24	2	2	92	cesfam miguel arenas lopez
35	6	6	0	cesfam miguel arenas lopez
38	2	2	111	cesfam miguel arenas lopez
34	5	5	111	cesfam central
11	3	3	75	cesfam Colón , Curicó
13	0	0	84	cesfam sol de septiembre, Curicó
18	3	3	0	cesfam sol de septiembre, Curicó

29	4	4	100	cesfam sol de septiembre, Curicó
31	0	0	0	cesfam sol de septiembre, Curicó
26	5	5	77	cesfam sol de sept., Curicó
18	1	1	85	cesfam Colón, Curicó
15	3	3	0	cesfam sol de septiembre, Curicó
21	6	6	92	cesfam sol de septiermbre, Curicó
27	1	1	0	cesfam sol de septiembre; curicó
30	2	2	113	NORTE
35	0	0	115	norte

46	3	3	112	norte
34	1	1	87	Norte
23	2	2	89	norte
20	0	0	68	norte
40	4	4	109	norte
33	0	0	100	norte
46	5	5	100	norte
26	3	3	81	norte
41	5	5	115	La Florida

51	1	1	106	La Florida
35	1	1	82	La Florida
36	1	1	69	La Florida
40	5	5	79	La Florida
40	2	2	82	La Florida
34	0	0	90	La Florida
35	4	4	104	La Florida
34	3	3	93	La Florida
32	1	1	85	La Florida

29	0	0	99	Carlos Trupp
28	5	5	76	Carlos Trupp
48	6	6	87	Carlos Trupp
39	4	4	92	Carlos Trupp
37	0	0	83	Carlos Trupp
25	2	2	97	Carlos Trupp
28	0	0	107	Carlos Trupp
45	0	0	107	Carlos Trupp
33	6	6	83	Carlos Trupp

54	6	6	104	cesfam las Américas
30	1	1	83	Cesfam Las Américas
24	4	4	0	cesfam Las AMéricas
40	4	4	0	cesfam Las américas
20	5	5	0	CESFAM LAS AMÉRICAS
30	4	4	76	cesfam las américas
23	1	1	77	CESFAM LAS AMÉRICAS
60	4	4	97	CESFAM LAS AMÉRICAS
31	1	1	82	NORTE

34	2	2	95	norte
35	1	1	113	NORTE
22	3	3	108	NORTE
34	2	2	101	NORTE
34	0	0	83	NORTE
31	3	3	122	NORTE
28	5	5	111	NORTE
39	2	2	99	NORTE
37	2	2	111	Norte

47	5	5	121	NORTE
26	4	4	82	sarmiento
34	4	4	0	sarmineto
21	5	5	0	sarmineto (doña carmen)
29	2	2	0	DOÑA CARMEN, SARMIENTO
23	3	3	78	sarmiento
21	3	3	78	sarmiento
43	3	3	103	sarmiento cecof
12	2	2	0	sarmiento

7	1	1	82	SARMIENTO
30	0	0	80	SARMIENTO
37	0	0	0	consultorio central
3	1	1	51	c central
45	2	2	97	central
33	2	2	101	central
56	2	2	86	central
33	1	1	100	central
33	5	5	93	central

19	3	3	60	central
6	1	1	59	219
57	0	0	0	220
43	2	2	0	Central
33	0	0	69	222
26	3	3	0	Central
20	4	4	0	Central
43	0	0	80	Central
47	3	3	111	Central

24	0	0	86	Central
33	3	3	72	Central
27	0	0	0	Central
28	0	0	0	Central
32	3	3	99	cesfam central
27	4	4	96	cesfam central
37	1	1	110	cesfam central
50	3	3	121	cesfam miguel arenas lopez
37	1	1	119	cesfam central

31	2	2	105	cesfam central
27	5	5	86	cesfam central
31	4	4	92	CESFAM MIGUEL ARENAS LOPEZ
26	2	2	99	cesfam central
44	3	3	101	cesfam central
36	4	4	0	cesfam sol de septiembre, Curicó
25	3	3	94	CEsfam Sol de Septiembre, Curicó
30	1	1	0	Cesfam Colón, Curicó
22	0	0	0	Cesfam Colón, Curicó

29	3	3	76	cesfam Colón, Curicó
19	1	1	91	cesfam sol de septiembre, Curicó
29	4	4	0	cesfam Sol de Septiembre Curicó
32	2	2	106	CESFAM SOL DE SEPTIEMBRE, CURICÓ
21	3	3	68	Cesfam Sol de Sept, Curicó
27	0	0	94	cesfam sol de septiembre , Curico
23	0	0	84	La Florida
25	1	1	89	La Florida
39	4	4	88	La Florida

28	1	1	72	La Florida
48	0	0	92	La Florida
42	3	3	80	La Florida
26	1	1	122	La Florida
18	1	1	89	La Florida
40	3	3	88	La Florida
35	6	6	113	La Florida
32	0	0	0	Carlos Trupp
33	0	0	78	Carlos Trupp

17	1	1	100	Carlos Trupp
58	4	4	91	Carlos Trupp
50	4	4	75	Carlos Trupp
52	1	1	124	Carlos Trupp
28	4	4	87	Carlos Trupp
44	0	0	89	Carlos Trupp
42	2	2	85	Carlos Trupp
53	4	4	120	Carlos Trupp
41	3	3	0	cesfam Las Américas

46	3	3	0	CESFAM LAS AMERICAS
46	4	4	0	cesfam Las Américas
15	0	0	0	CESFAM LAS AMÉRICAS
23	3	3	69	cesfam Las Américas
35	3	3	0	CESFAM LAS AMÉRICAS
46	4	4	0	CESFAM LAS AMERICAS
25	5	5	64	cesfam Las Américas
44	3	3	0	CESFAM LAS AMÉRICAS
34	4	4	85	CESFAM LAS AMÉRICAS

37	0	0	104	NORTE
26	0	0	85	NORTE
22	2	2	96	NORTE
24	1	1	89	NORTE
26	2	2	77	NORTE
30	4	4	123	NORTE
37	4	4	95	NORTE
33	2	2	92	NORTE
0	3	3	53	NORTE

45	2	2	97	Norte
44	2	2	97	La Florida
45	3	3	85	La Florida
48	3	3	113	La Florida
33	4	4	91	La Florida
29	1	1	95	La Florida
45	6	6	107	La Florida
28	1	1	87	La Florida
38	1	1	73	La Florida

27	3	3	81	La Florida
51	2	2	104	La Florida
30	5	5	109	cesfam miguel arenas lopez
44	1	1	109	cesfam miguel arenas lopez
25	2	2	85	cesfam central
28	2	2	110	cesfam miguel arenas lopez
37	3	3	93	miguel arenas lopez
48	6	6	97	cesfam miguel arenas lopez
35	2	2	108	cesfam central

38	4	4	74	cesfam central
22	3	3	94	cesfam central
30	2	2	94	cesfam central
19	2	2	111	cesfam central
27	0	0	88	cesfam central
40	4	4	113	cesfam central
27	1	1	0	cesfam central
23	4	4	86	cesfam curico centro
22	1	1	82	cesfam curico centro

33	5	5	120	cesfam m arenas lopez
35	6	6	92	cesfam central
31	4	4	110	cesfam m arenas lopez
29	6	6	97	cesfam miguel arenas lopez
24	3	3	57	CESFAM CENTRAL
46	1	1	124	cesfam central
51	6	6	103	casfam miguel arenas lopez
17	1	1	78	cesfam central
38	6	6	89	cesfam miguel arenas lopez

26	5	5	87	cesfam miguel arenas lopez
23	0	0	78	CentralSom
25	0	0	78	central
7	1	1	0	central
31	3	3	77	central
41	2	2	93	centralk
24	5	5	75	central
48	3	3	79	central
28	1	1	104	Central

29	1	1	79	central
40	3	3	92	central
28	1	1	103	central
45	0	0	0	342
45	5	5	90	central
49	6	6	104	central
39	3	3	97	central
48	5	5	99	central
22	2	2	102	central

34	1	1	0	Central
32	0	0	77	Central
10	0	0	73	central
45	4	4	88	Carlos Trupp
48	1	1	117	Carlos Trupp
55	1	1	0	Carlos Trupp
49	4	4	74	Carlos Trupp
41	2	2	119	Carlos Trupp
49	0	0	101	Carlos Trupp

28	5	5	0	Carlos Trupp
15	0	0	96	Carlos Trupp
45	5	5	95	Carlos Trupp
59	6	6	0	Carlos Trupp
13	3	3	0	CESFAM LAS AMÉRICAS
22	1	1	64	NORTE
20	2	2	66	NORTE
48	1	1	101	NORTE
37	1	1	95	NORTE

38	1	1	93	NORTE
16	4	4	91	NORTE
30	3	3	108	NORTE
37	1	1	98	NORTE
29	3	3	77	NORTE
29	2	2	95	NORTE
34	1	1	73	La Florida
36	3	3	100	La Florida
34	2	2	81	La Florida

19	2	2	105	La Florida
40	3	3	91	La Florida
35	2	2	100	La Florida
41	4	4	96	La Florida
47	2	2	102	Carlos Trupp
52	4	4	0	Carlos Trupp
15	1	1	0	Carlos Trupp
4	0	0	62	Carlos Trupp
38	3	3	110	Carlos Trupp

36	6	6	89	Carlos Trupp
35	5	5	81	Carlos Trupp
34	2	2	105	Carlos Trupp
38	3	3	57	Carlos Trupp
45	0	0	77	Carlos Trupp
46	6	6	100	Cesfam Miguel Angel Arenas Lopez, Curicó
10	0	0	78	cesfam sol de septiembre, Curicó
31	0	0	102	Cesfam sol de septiembre, Curicó
32	5	5	88	cesfam Colón, Curicó

26	0	0	0	Cesfam Sol de septiembre, Curicó
13	3	3	69	central
46	0	0	114	central
28	2	2	67	central
48	5	5	0	central
23	0	0	85	central
34	4	4	70	vvg
54	6	6	112	c central
31	1	1	0	c central

44	1	1	73	central
21	3	3	77	central
20	3	3	72	consultorio central
39	0	0	102	consultorio central
23	1	1	97	consultorio central curico
11	4	4	72	cons central curico
38	1	1	82	consult central curico
39	0	0	76	consult central curico
30	5	5	113	consult central curico

41	0	0	100	cons central curico
22	5	5	115	cons central curico
13	0	0	0	consultorio central
39	3	3	88	cons central curico
35	2	2	93	consultorio central
6	1	1	97	consultorio central
19	2	2	65	cons central curico
15	3	3	0	cons central curico
31	1	1	84	NORTE

36	1	1	113	NORTE
35	0	0	108	NORTE
8	1	1	66	NORTE
40	5	5	110	NORTE
24	7	7	97	NORTE
22	1	1	118	NORTE
24	4	4	93	NORTE
27	3	3	107	NORTE
31	3	3	93	NORTE

49	1	1	79	Carlos Trupp
41	4	4	99	Carlos Trupp
16	0	0	79	Carlos Trupp
27	2	2	98	Carlos Trupp
50	0	0	100	Carlos Trupp
42	1	1	99	Carlos Trupp
36	2	2	111	Carlos Trupp
13	0	0	78	Carlos Trupp
29	0	0	79	Carlos Trupp

15	0	0	66	Carlos Trupp
37	1	1	112	NORTE
18	2	2	74	NORTE
44	0	0	89	Carlos Trupp
41	5	5	116	Carlos Trupp
45	5	5	92	Cesfam Las Américas
25	2	2	61	central
41	5	5	98	central
0	2	2	0	CESFAM LAS AMÉRICAS

0	0	0	0	La Florida
0	4	4	0	Central

Tiempomesesepisodioanterior	ANTECEDENTESGINECOLÓGICOSNºDEPARTOS	ANTECEDENTESGINECOLÓGICOSNºDEABORTOS	MESESEMBARAZO	MESESPOSTPARTO
4	1	1	0	0
2	1	1	0	0
9	2	2	0	0
0	0	0	0	0
0	2	2	0	0
2	0	0	0	0
24	1	1	0	0
2	1	0	0	0

0	2	0	0	0
84	1	0	0	0
6	2	0	0	0
4	0	0	0	0
18	1	1	0	0
24	1	0	0	0
14	2	0	0	0
240	2	1	0	0
36	2	0	0	0

12	2	0	0	0
12	1	0	0	0
12	2	0	0	0
18	2	1	0	0
6	2	0	0	0
3	1	1	0	0
36	2	0	0	0
0	1	0	0	0
12	2	1	0	0

12	1	0	0	0
60	2	0	0	0
12	1	0	0	0
24	2	0	0	0
4	0	0	0	0
0	2	0	0	0
24	2	2	0	0
6	2	1	0	0
3	1	0	0	0

0	0	0	0	0
0	1	1	0	0
4	2	1	0	0
2	1	0	0	0
1	2	1	0	0
0	0	0	0	0
12	1	0	0	0
0	0	0	0	0
0	2	0	0	0

12	1	1	0	0
0	0	0	0	0
24	1	1	0	0
18	1	0	0	0
0	1	0	0	2
0	1	0	0	0
6	2	1	0	0
7	1	0	0	0
3	0	0	0	0

18	2	0	0	0
0	2	0	0	0
0	1	0	0	0
0	1	0	0	0
0	1	0	0	0
0	1	0	0	0
0	1	0	0	0
24	1	0	0	0
3	1	1	0	0

7	2	1	0	0
0	0	0	0	0
0	2	0	0	0
0	0	0	0	0
3	0	1	0	0
7	1	0	0	0
24	2	1	0	0
0	1	0	0	0
0	1	0	0	0

0	2	2	0	0
60	2	1	0	0
0	0	0	0	0
0	2	0	0	0
0	1	0	0	0
24	2	0	0	0
2	1	1	0	0
0	2	1	0	0
60	1	1	0	0

3	1	1	0	0
0	2	0	0	0
36	1	0	0	0
0	2	0	0	0
24	2	1	0	0
0	1	1	0	0
0	2	1	0	0
24	2	0	0	0
84	2	0	0	0

5	1	0	0	0
24	2	0	0	0
24	2	0	0	0
3	0	0	0	0
12	0	1	0	0
4	0	0	0	0
3	0	0	0	0
24	1	1	0	0
120	1	0	0	0

48	1	1	0	2
36	2	1	0	0
0	1	0	5	0
12	2	0	0	0
12	2	0	0	0
2	2	0	0	0
3	1	1	0	0
0	1	0	0	0
0	2	1	0	0

24	1	2	0	0
36	1	0	0	0
24	1	0	0	0
36	1	0	0	0
60	1	0	0	0
36	2	0	0	0
0	1	0	0	0
60	1	0	0	0
1	1	1	0	0

0	2	1	0	0
12	2	0	0	0
48	1	0	0	0
12	1	0	0	0
0	2	1	0	0
48	2	0	0	0
2	0	0	0	0
12	0	0	0	0
12	1	0	0	0

84	0	0	2	0
120	1	2	0	0
12	2	0	0	0
48	1	0	0	4
48	2	0	0	0
4	1	0	0	0
588	0	0	0	0
0	1	0	0	0
0	2	0	0	0

0	2	1	0	0
84	1	1	0	0
12	1	0	0	7
24	2	1	0	0
24	2	0	0	0
0	1	0	0	0
0	0	0	0	0
3	1	0	0	0
0	1	1	0	0

0	1	0	0	0
0	2	1	0	0
6	2	0	0	0
0	1	0	0	0
0	0	0	0	0
6	2	1	0	0
24	2	0	0	0
3	1	0	0	0
1	1	0	0	0

36	2	0	0	0
4	1	1	0	0
0	1	0	0	0
24	1	1	0	0
0	2	0	0	0
12	1	0	0	0
24	2	0	0	0
0	0	0	0	0
24	0	0	0	0

12	1	1	0	0
0	0	0	0	0
24	0	1	0	0
12	1	1	0	0
12	2	0	0	0
0	1	0	0	7
12	2	1	0	0
12	2	0	0	0
24	1	0	0	0

0	2	0	0	0
0	0	0	0	0
2	1	1	0	0
7	2	1	0	0
36	1	0	0	0
12	0	0	0	0
0	1	0	0	0
0	2	0	0	0
12	2	1	0	0

72	2	1	0	0
3	0	0	0	0
48	0	0	0	0
0	1	1	0	0
24	0	0	0	0
24	1	1	0	0
5	0	0	0	0
9	1	0	0	1
6	1	0	0	0

36	0	0	7	0
0	1	1	0	0
12	2	1	0	0
5	2	0	0	0
6	1	1	0	0
2	1	0	0	0
1	0	0	0	0
48	2	2	0	0
12	1	0	0	0

168	1	0	0	0
12	2	1	0	0
0	2	0	0	0
24	1	0	0	0
36	2	0	0	0
0	1	0	0	0
5	2	1	0	0
3	2	1	0	0
0	2	0	0	0

0	1	0	0	8
0	1	0	0	0
2	1	0	0	0
0	0	0	0	0
36	2	0	0	0
48	2	1	0	0
4	2	0	0	0
5	0	0	0	0
60	2	2	0	0

0	0	0	0	0
1	2	1	0	0
0	0	0	0	0
0	0	0	0	0
36	2	1	0	0
6	2	1	0	0
12	2	1	0	0
24	0	0	0	0
3	2	0	0	0

6	2	0	0	0
36	2	1	0	0
0	1	0	0	0
0	0	0	0	0
6	1	0	0	0
12	2	0	0	0
0	0	0	0	0
0	1	0	0	0
0	2	1	0	0

0	1	0	0	0
0	0	0	0	0
0	1	0	0	3
12	2	1	0	0
12	0	0	3	0
24	2	1	0	0
12	2	0	0	0
0	0	0	0	0
12	1	1	0	0

12	2	0	0	0
0	1	0	0	7
0	2	1	0	0
0	2	0	0	0
24	0	1	6	0
0	1	0	7	0
1	0	0	0	0
24	0	0	0	0
12	1	1	0	0

0	0	0	0	0
0	1	0	0	0
24	1	0	0	0
6	0	0	0	0
1	0	0	0	0
12	2	1	0	0
12	2	1	0	0
6	2	0	0	0
0	1	0	0	0

8	2	0	0	0
0	1	0	0	0
3	0	0	0	0
0	1	0	0	0
636	2	1	0	0
0	1	0	0	0
18	1	0	0	0
0	1	0	0	0
24	2	0	0	0

0	2	0	0	0
5	1	1	0	0
0	1	0	7	0
36	0	0	0	0
60	2	0	0	0
6	2	0	0	0
12	2	0	0	0
24	2	1	0	0
0	2	0	0	0

18	2	0	0	0
0	0	0	0	0
24	1	1	0	0
36	2	1	0	0
6	2	0	0	0
36	0	0	0	0
36	1	0	0	0
12	2	0	0	0
6	1	0	0	0

48	1	0	0	0
24	2	1	0	0
2	1	0	0	6
4	1	0	0	0
2	0	0	0	0
36	0	0	0	0
12	1	0	0	0
0	0	0	0	0
0	1	1	0	0

6	0	1	0	0
4	2	1	0	0
3	1	0	0	0
12	2	1	0	0
5	1	0	0	0
60	1	1	0	0
12	1	1	0	0
1	2	0	0	0
12	2	0	0	0

6	1	0	0	0
6	1	0	0	0
0	0	0	0	0
36	1	1	0	0
0	0	0	0	0
0	1	1	0	0
0	0	0	0	0
12	2	1	0	0
6	1	1	0	0

0	2	1	0	0
1	2	0	0	0
0	2	0	0	0
0	2	0	0	0
0	0	0	0	0
12	0	0	0	0
6	1	0	0	0
0	0	0	0	0
3	2	0	0	0

0	1	0	0	0
96	2	1	0	0
12	2	0	0	0
3	0	0	0	0
12	1	1	0	0
240	2	0	0	0
84	1	1	0	0
12	1	0	0	2
12	2	0	0	3

6	0	0	0	0
0	0	0	0	0
36	2	0	0	0
0	0	0	0	0
6	2	0	0	0
24	2	0	0	0
12	2	0	0	9
360	2	1	0	0
120	0	0	0	0

2	1	0	0	0
0	0	0	7	0
0	2	1	0	0
0	1	0	0	0
0	2	1	0	0
0	2	0	0	0
0	2	0	0	0
2	2	0	0	0
6	2	0	0	0

0	2	1	0	0
2	2	1	0	0
120	2	1	0	0
0	0	0	0	0
10	0	0	0	0
12	1	0	0	0
2	0	0	0	0
24	0	0	0	0
12	0	0	0	0

4	1	0	0	0
0	2	1	0	0
18	1	1	0	0
240	2	0	0	0
0	2	1	0	0
18	1	1	0	0
0	0	0	0	0
0	1	0	0	0
12	1	0	0	0

0	0	0	0	0
6	1	0	0	0
7	1	0	0	7
12	2	0	0	0
0	2	1	0	0
6	2	0	0	0
48	2	0	0	0
0	1	1	0	0
8	2	1	0	0

0	0	0	0	0
3	2	1	0	0
0	1	0	0	0
24	2	0	0	0
0	0	0	0	0
1	2	1	0	0
0	2	0	0	0
0	1	0	0	0
12	1	1	0	0

12	1	1	0	0
144	2	0	0	0
6	1	0	0	0
12	0	0	0	0
12	0	0	0	0
0	2	1	0	0
96	0	0	0	0
24	1	1	0	0
2	1	1	0	4

12	1	1	0	0
0	1	1	0	0
18	0	0	0	0
12	1	0	0	3
24	1	1	0	0
0	0	0	0	0
18	1	0	0	0
12	0	0	0	0
12	2	1	0	0

3	1	1	0	0
2	2	1	0	8
12	1	0	0	0
0	2	1	0	0
0	0	0	0	0
24	1	1	0	0
0	0	0	0	0
0	2	0	0	0
0	1	0	0	0

0	2	1	0	0
3	1	0	0	0
0	0	0	0	0
24	2	0	0	0
2	1	0	0	0
12	2	0	0	0
12	1	0	0	0
6	1	0	0	0
24	0	0	0	0

20	2	0	0	0
3	1	0	0	0
0	2	1	0	0
60	2	1	0	0
1	2	0	0	0
0	2	0	0	0
0	0	0	0	0
0	0	0	0	0
4	2	0	0	0

45	1	1	0	0
240	0	1	0	0
3	1	0	0	0
0	2	0	0	0
2	0	0	0	0
6	2	1	0	0
0	1	0	0	0
12	2	1	0	0
12	2	0	0	0

0	0	0	0	0
12	2	1	0	0

VIHSIDA	CUANTOSEPISODIOSDEDEPRESIÓNHATENIDO	EDADDELPRIMEREPISODIODEPRESIVO	TIEMPODEDURACIÓNDELEPISODIODEPRESIVOANTERIOR	TIEMPODEDURACIÓNDELEPISODIOMÁSPROLONGADO
0	6	63	4 meses	1
0	5	17	2meses	1
0	1	51	9meses	1
0	0	0		0
0	0	0		0
0	1	12	2meses	0
0	5	53	2 años	2
0	1	26	2meses	1

0	3	40	1 a	1
0	1	28	7 años	7
0	3	60	6 meses	1
0	1	26	4 m	1
0	5	25	1, 5 año	1
0	3	13	2 años	2
0	3	46	14	14
0	1	20	20 años	20
0	4	28	3 años	3

0	2	19	1 año	1
0	1	31	1	1
0	3	30	1 año	1
0	25	18	1año 6 meses	3
0	2	42	meses	1
0	2	53	tres meses	1
0	1	41	3años	3
0	0	36	0	0
0	3	26	1 año	1

0	4	15	1 año	1
0	15	9	años	10
0	2	24	1 año	1
0	25	19	2 años	2
0	2	44	4 meses	1
0	0	33		1
0	2	30	2 años	7
0	3	37	6 meses	1
0	4	16	3 meses	3

0	0	0	0	0
0	0	0		0
0	3	23	4 meses	1
0	1	39	2 meses	1
0	1	35	1 mes	1
0	0	48	0	1
0	1	60	1 año	2
0	0	0		2
0	1	45	hasta ahora	24

0	4	23	1 año	1
0	0	0	-	1
0	3	15	2 años	7
0	5	7	1,5 años	3
0	0	0		0
0	0	0		1
0	2	25	6 meses	3
0	6	17	7 meses	1
0	4	26	3 meses	14

0	15	38	un año y medio	1
0	1	46	HASTA LA FECHA	14
0	0	63	NO APLICA	1
0	0	0		0
0	0	31	NO APLICA	1
0	1	63	NO RECUERDA	1
0	15	28	NO RECUERDA	3
0	2	58	2 AÑOS	0
0	1	19	3 MESES	1

0	2	52	7 MESES	1
0	1	66		5
0	0	70	no aplica	1
0	1	63	NO	1
0	2	68	3 meses	1
0	9	12	7 meses	1
0	10	28	2 AÑOS	5
0	4	32	no lo sabe	1
0	0	0	no aplica	1

0	0	15	No aplica	45
0	2	15	5 años	12
0	1	15	No aplica	6
0	0	91		1
0	1	37	NO APLICA	22
0	15	18	2 AÑOS	14
0	4	15	2 meses	5
0	0	72	no aplica	1
0	2	39	5 años	5

0	3	22	3 MESES	1
0	15	10	NO RECUERDA	1
0	10	6	3 AÑOS	0
0	0	57		1
0	15	5	2 AÑOS	3
0	10	26	NO RECUERDA	1
0	0	0		0
0	5	15	2 años	5
0	10	38	6 años	6

0	2	6	5 meses	1
0	3	30	2 AÑOS	2
0	3	16	2 años	2
0	2	20	3 meses	1
0	3	10	1 año	2
0	2	48	4 meses	1
0	3	24	3 meses	1
0	2	57	2 años	2
0	3	44	10 años	10

0	3	8	4 años	4
0	10	5	3 años	3
0	0	0		0
0	3	31	1 año	1
0	4	37	1 año	1
0	2	20	2 meses	1
0	2	36	3 meses	1
0	0	30	no sabe	13
0	1	40		1

0	4	14	2 años	2
0	4	22	3 años	3
0	1	32	2 años	2
0	15	15	3 años	59
0	1	4	5 años	45
0	15	20	3 años	3
0	2	30		0
0	5	42	5 años	5
0	10	15	1 mes	25

0	1	48	0	0
0	2	62	1 año	1
0	1	56	4 años	4
0	20	18	1 año	1
0	1	60	0	0
0	3	45	4 años	4
0	5	30	2meses	1
0	2	50	1 año	3
0	1	35	1 año	1

0	1	28	6 años	6
0	4	20	10 años	10
0	4	37	1 año	1
0	1	21	4 años	4
0	3	28	4 años	4
0	1	15	4 MESES	1
0	2	58	es el mismo que se ha mantenido	7
0	0	0		0
0	0	0		0

0	3	41	17 años	17
0	3	7	7 años	7
0	1	23	1 año	1
0	2	30	2 años	3
0	2	24	2 años	3
0	0	45		0
0	0	0		1
0	3	43	3 meses	1
0	0	0	nc	1

0	0	40	nc	1
0	0	0		1
0	1	16	6-8 meses	1
0	0	40	nc	1
0	0	0	nc	1
0	1	36	6 meses	1
0	2	61	2 años	3
0	2	21	3 MESES	1
0	3	32	1 mes	1

0	2	44	3 año	3
0	4	16	4 meses	1
0	1	57	no	1
0	2	20	2 años	3
0	0	65	no	0
0	6	23	1 año	5
0	2	19	2 años	2
0	1	75	NO	1
0	4	8	2 años	3

0	5	19	1 año	1
0	1	15	N/A	1
0	8	13	2 años	2
0	8	13	1 año	1
0	4	21	1 año	1
0	0	40	N/A	1
0	4	14	1 año	1
0	2	39	1 año	1
0	5	16	2 años	2

0	1	48	no aplica	1
0	0	15	NO aplica	1
0	15	50	2 meses	1
0	1	51	no recuerda pero fueron hartos meses	1
0	15	8	3 años	3
0	3	23	12 meses	1
0	0	56	no hubo	1
0	0	12	no sabe	0
0	7	12	12 meses	1

0	5	25	2009	3
0	3	17	3 meses	1
0	10	22	4 años aprox	9
0	5	14	asociado a problemas familiares, tíos. amlos tratos, VIF	4
0	5	10	2 AÑOS	10
0	3	30	2 AÑOS	3
0	5	10	5 MESES	1
0	10	7	9 MESES	1
0	2	16	6 MESES	1

0	2	17	3 AÑOS	3
0	1	50	NO	0
0	3	23	1 AÑO	3
0	2	20	5 MESES	1
0	2	43	6 MESES	1
0	3	16	2 MESES	1
0	1	51	ACTUAL	1
0	8	4	4 AÑOS	4
0	3	18	12 MESES	1

0	6	32	14 AÑOS	14
0	1	42	1 año	1
0	0	0		0
0	3	15	2 años	2
0	10	24	3 AÑOS	3
0	0	0		0
0	2	23	5 MESES	1
0	8	31	3 a 4 meses	2
0	0	0		0

0	0	0		0
0	0	0		0
0	5	12	2 meses	1
0	0	0		0
0	2	14	3 años	3
0	2	36	4 años	4
0	5	20	4 meses	1
0	3	15	5 meses	1
0	2	4	5 años	5

0	0	15		0
0	6	20	1 mes	1
0	0	0		0
0	0	41		0
0	2	43	3-años	3
0	2	60	6meses	1
0	5	25	1año	1
0	6	8	Dos años	3
0	3	13	3 meses	1

0	8	46	6meses	2
0	25	5	3años	3
0	1	42	0	1
0	1	26	0	1
0	3	18	6 meses	1
0	2	38	1 año	1
0	0	0		0
0	10	0		0
0	0	0		0

0	0	0		0
0	0	0		0
0	0	0		0
0	2	30	1 año	1
0	1	15	1 año	3
0	1	49	es el actual: 2 años	0
0	2	27	1 año	1
0	0	13	nc	0
0	2	41	1 año.	0

0	1	33	un año, Pero refiere que nunca quedó sin síntomas	1
0	0	0	nc	0
0	1	0	nc	0
0	1	28	el actual	1
0	1	13	2 años	2
0	0	0	nc	1
0	2	21	1 mes	2
0	2	9	2 años	4
0	4	19	1 año	1

0	0	49	N/A	1
0	1	46		1
0	2	16	2 años	2
0	10	25	6 meses	4
0	5	25	1 mes	1
0	2	25	1 año	1
0	5	8	1 año	10
0	25	27	6 meses	2
0	0	35		3

0	2	19	8 meses	1
0	0	32		2
0	2	26	3 meses	1
0	1	45	No tiene	2
0	1	8	siempre	59
0	1	41	No aplica	1
0	15	30	1,5 años	2
0	1	52	no aplica	1
0	2	10	2 años	2

0	1	61		1
0	1	21	5 meses	1
0	0	19		1
0	2	64	3 AÑOS APROX	3
0	2	26	5 AÑOS	6
0	10	45	NO RECUERDA, REFIERE MESES	2
0	2	36	1 año	2
0	2	34	2 AÑOS	3
0	1	49		3

0	13	19	18 MESES	2
0	1	15	NO	1
0	3	44	2 AÑOS	2
0	4	48	3 AÑOS	4
0	3	32	6 MESES	1
0	4	8	3 AÑOS	10
0	7	8	3 AÑOS	4
0	2	39	1 AÑO	1
0	5	14	6 MESES	1

0	5	5	4 AÑOS	4
0	8	22	2 años	3
0	5	12	2 meses	1
0	3	15	4 meses	1
0	5	18	2 meses	1
0	3	40	3 años	6
0	4	14	1 año	1
0	1	35	N/A	3
0	1	57	N/A	1

0	3	15 6 meses	1
0	5	22 4 meses	1
0	1	21 3 meses	1
0	2	40 1 año	2
0	2	19 5 meses	1
0	1	43 5 años	5
0	4	16 1 año	3
0	1	38 1 mes	1
0	1	22 1 año	1

0	3	17	6 meses	1
0	1	17	6 meses	1
0	0	0		1
0	1	46	3 años	3
0	0	0		0
0	0	0		1
0	0	0		1
0	1	55	1 año	1
0	1	43	6 meses	1

0	0	0		1
0	1	54	1 mes	60
0	0	0		0
0	0	0		0
0	0	64		0
0	1	24	1 año	1
0	1	34	6 meses	1
0	0	0		0
0	4	36	3 meses	4

0	1	27	no se mejoró solo dejo el tratamiento	10
0	2	51	8años	8
0	3	30	1año	1
0	10	42	3 meses	1
0	2	17	1 año	1
0	1	30	20 años	20
0	5	8	7 años	15
0	0	22	1 año	1
0	2	15	1 año	1

0	2	55	6 meses	1
0	1	60	0	1
0	1	42	3	3
0	4	30	12	1
0	14	39	5-6 meses	1
0	3	43	2 años	2
0	2	29	1 año	1
0	15	9	30 años	30
0	10	8	10 años	10

0	3	14	2 meses	2
0	2	27	No recuerda	0
0	1	69	0	0
0	1	62	no aplica	1
0	1	54	No aplica	1
0	1	77	No aplica	1
0	1	62	No aplixa	5
0	2	68	2 meses	1
0	3	35	6 meses	1

0	1	7	No aplica	65
0	3	43	2 meses	1
0	2	33	10 años	10
0	1	13	No aplica	1
0	1	56	10 MESES	1
0	7	20	1 AÑO	1
0	3	51	2 MESES	1
0	3	6	2 AÑOS	2
0	2	21	1 AÑO	1

0	3	18	4 MESES	1
0	0	80	0	0
0	3	30	1 1/2 AÑO	2
0	3	25	20 AÑOS	20
0	1	60	NO	0
0	2	15	18 MESES	1
0	1	52	N/A	4
0	1	40	N/A	1
0	6	9	1 año	3

0	1	57	N/A	1
0	3	26	6 meses	1
0	4	13	7 meses	3
0	3	43	1 año	2
0	1	7	no aplica	46
0	2	26	6 meses	1
0	3	37	4 años	7
0	0	0	no aplica	1
0	2	58	8 meses	3

0	1	51	no aplica	1
0	15	14	3 meses	1
0	1	20	No aplica	4
0	7	29	2 años	2
0	1	21	NO aplica	39
0	1	0	el actual	1
0	0	72	mismo actual	1
0	0	0	nc	1
0	4	20	1 año	1

0	2	25	1 año	1
0	3	39	12 años	12
0	2	47	meses	1
0	5	18	1 año	1
0	20	25	1 año	2
0	1	64		1
0	10	10	8 años	8
0	3	13	1 a 2 años	3
0	3	14	2 meses	1

0	3	53	1 ano	1
0	4	50	6 meses	1
0	3	35	1 ano y medio aprox	3
0	3	28	1 ano	1
0	7	11	2 años	2
0	1	20	dias (renuncio al trabajo y se sintio mejor)	1
0	5	20	1 ano zy un poco mas	8
0	10	35	1 ano	1
0	2	45	mas de un ano	1

0	3	60	3 meses	3
0	3	18	2 meses	1
0	1	40	1 año	1
0	0	0		0
0	0	0		0
0	2	62	2 anos	2
0	0	0		0
0	0	0		0
0	3	27	10 MESES	1

0	1	36	NO	0
0	1	23	3 MESES ACTUAL	1
0	1	70	NO	1
0	8	20	2 AÑOS	6
0	3	31	2 MESES	1
0	2	32	1 AÑO	1
0	12	28	1 AÑO	2
0	4	15	6 MESES	1
0	2	19	2 AÑOS	2

0	2	16	20	20
0	5	19	3 meses	2
0	1	34	No aplica	1
0	2	43	5 años	5
0	3	56	1 mes	1
0	1	46	No aplica	1
0	5	28	no recuerda	1
0	1	53	NO aplica	4
0	1	64	4 meses	1

0	4	40	45	1
0	5	13	20 AÑOS	20
0	6	7	3 MESES	15
0	1	17	no aplica	45
0	5	30	1 mes y medio	2
0	10	12	no recuerda, meses	1
0	1	32		1
0	5	17	1 año	10
0	1	31	1 AÑO	1

0	0	0		0
0	3	32 1año		2

ANTECEDENTESDEFAMILIARCONDEPRESION	TRATAMIENTOPSICOLOGICOPORDEPRESIÓNANTERIOR	INDIQUESIALGÚNFAMILIARSESUICIDÓPADREMADRETÍOSABU	ESTÁENTRATAMIENTOPORHIPERTENSIÓN	ESTÁENTRATAMIENTOPORDIABETES
0	0	0	0	0
0	0	0	1	1
1	0	0	1	0
0	0	0	0	0
1	0	0	1	0
0	1	0	0	0
0	0	0	1	1
1	0	0	0	0

1	1	0	0	0
0	1	1	0	0
1	1	0	1	0
0	1	0	0	0
1	0	0	0	0
1	0	1	0	0
1	1	0	0	0
0	0	1	1	0
0	0	0	0	0

0	1	1	0	0
0	0	0	1	0
1	0	0	0	0
1	0	0	0	0
1	1	0	0	0
1	1	0	1	0
1	0	0	0	0
0	0	0	0	1
1	1	0	1	0

1	0	0	0	0
1	0	1	0	0
0	0	0	0	0
1	1	1	1	0
0	0	1	1	0
0	0	0	0	0
0	0	0	1	0
1	0	0	0	0
1	1	0	0	0

1	0	0	0	0
0	0	0	1	0
0	1	0	0	0
1	0	0	1	0
1	0	0	0	0
0	0	0	0	0
0	0	0	0	0
0	0	0	0	0
1	1	0	1	0

0	1	0	1	0
0	0	0	0	0
1	1	1	1	1
1	1	1	0	0
1	0	0	0	0
1	0	0	0	0
1	1	1	0	0
0	1	1	0	0
0	0	0	0	0

0	1	0	0	0
0	1	0	0	0
1	0	0	1	0
1	0	0	0	0
0	0	0	0	0
1	1	1	0	1
1	0	0	0	0
1	0	0	1	1
1	0	0	0	0

1	0	0	1	0
0	0	1	1	0
1	0	0	1	1
0	0	0	0	0
1	0	0	1	1
0	1	0	0	0
1	1	0	1	0
1	0	1	1	1
0	0	0	1	0

0	0	0	1	0
1	0	0	1	1
1	0	0	0	0
0	0	1	1	0
0	0	0	0	0
1	0	0	1	0
0	1	0	0	0
0	0	0	1	0
0	1	0	1	0

1	0	0	1	1
0	1	1	1	0
1	1	1	0	0
1	0	0	1	1
1	1	0	0	0
0	1	0	0	0
0	0	0	1	0
1	0	1	0	0
0	1	0	1	0

0	1	0	0	0
1	0	0	0	0
1	0	0	0	0
1	0	0	0	1
1	1	0	0	0
0	0	0	0	0
1	1	1	0	0
0	1	0	1	1
1	1	1	1	1

1	0	0	0	0
1	1	0	0	0
1	0	0	0	0
1	0	0	0	0
1	1	1	1	0
1	0	1	0	0
0	0	0	0	1
0	0	0	0	0
0	0	1	0	0

1	1	0	0	0
1	1	0	1	0
1	1	0	1	0
1	1	0	1	0
1	0	1	1	0
1	0	0	1	0
0	1	0	0	0
1	0	1	1	1
1	0	0	0	0

0	0	0	0	0
1	0	0	1	1
1	0	1	0	0
1	1	0	0	0
1	0	0	0	1
0	0	0	0	0
1	0	0	1	0
0	0	0	1	0
0	0	1	0	0

1	0	1	0	0
1	0	0	0	0
0	1	0	1	0
1	0	0	0	0
1	1	1	0	0
0	0	0	0	0
1	0	0	1	0
1	0	1	0	0
0	0	0	0	0

0	0	0	1	0
1	0	1	0	0
1	1	1	0	0
1	0	0	0	0
1	1	0	0	0
1	0	0	0	0
1	0	0	0	0
1	0	0	0	0
1	0	1	0	0

0	0	0	0	0
1	0	0	0	0
1	0	0	1	0
1	0	0	0	0
1	0	0	0	0
0	1	0	0	0
0	1	0	1	0
1	0	0	0	0
0	1	0	0	0

0	1	0	0	0
0	0	0	0	0
1	0	0	0	0
0	0	0	0	0
1	0	1	1	0
0	0	0	0	0
0	0	0	0	0
1	0	0	1	0
0	0	0	0	0

0	1	0	0	0
0	0	0	0	0
1	0	0	0	0
1	0	0	0	0
0	0	0	0	0
1	0	0	0	0
0	0	0	0	1
0	0	0	0	0
0	0	0	0	0

0	0	0	0	0
1	0	0	0	0
1	1	0	1	1
1	1	0	0	1
1	0	0	1	0
0	1	0	0	0
0	0	0	0	0
1	0	0	0	0
0	1	0	0	0

0	1	0	0	0
1	1	0	0	0
0	0	0	1	0
1	1	1	0	1
1	1	0	1	1
1	0	1	1	1
0	0	1	0	0
0	1	0	0	0
1	1	0	0	0

1	0	0	0	0
0	0	0	0	0
0	1	0	0	0
0	1	0	0	0
0	1	0	1	0
0	0	0	0	0
0	0	0	0	0
0	0	0	1	0
1	0	0	0	0

0	1	0	1	0
0	0	1	0	0
0	0	0	0	0
1	1	1	0	0
1	1	1	1	0
0	0	0	1	0
1	0	0	0	0
0	1	0	1	0
0	0	0	0	0

0	0	0	0	0
0	0	1	0	0
0	1	0	0	0
0	0	0	0	1
0	0	0	0	0
0	1	0	0	1
1	1	0	0	0
1	1	0	0	0
1	0	0	0	0

1	0	0	0	0
0	0	1	0	1
0	0	0	0	0
1	0	0	0	0
1	0	1	1	1
1	0	0	1	1
1	0	0	1	0
0	1	0	0	0
0	0	0	0	0

1	0	0	0	0
0	0	0	0	1
0	0	0	1	0
1	0	1	0	0
1	1	0	0	0
1	1	0	1	0
0	0	0	0	0
0	0	0	0	0
0	0	0	1	1

0	0	0	0	0
0	0	0	0	0
0	0	0	0	0
1	1	0	1	0
1	1	0	0	0
0	0	0	1	0
0	0	0	1	1
0	1	0	0	0
1	0	0	0	0

0	0	0	1	0
1	0	0	1	0
0	0	0	1	0
1	1	0	0	0
1	1	0	0	0
1	0	0	0	0
0	0	0	1	1
1	0	0	0	0
0	0	0	0	0

0	0	0	0	0
0	0	0	0	0
0	0	1	0	0
0	0	0	1	1
1	0	1	1	0
1	0	1	0	1
0	0	0	0	0
1	1	0	0	0
1	0	0	0	0

1	1	0	0	0
0	0	0	0	0
1	0	0	0	0
1	0	0	0	0
1	0	0	1	0
1	0	0	0	0
1	0	0	1	0
1	0	0	0	0
0	1	0	1	0

0	0	0	1	0
0	0	0	0	0
0	0	0	0	0
1	1	0	1	0
0	1	0	1	0
0	1	1	0	0
1	0	1	0	0
1	0	0	0	0
1	0	1	1	1

1	1	0	1	0
1	0	0	0	0
0	0	1	0	0
0	1	0	1	1
0	1	0	0	0
0	1	1	1	0
1	1	1	0	0
1	0	1	1	1
1	1	0	1	0

1	0	1	0	0
0	1	1	1	1
0	0	0	0	0
0	0	0	0	0
1	0	0	1	0
1	0	1	1	0
0	0	0	0	0
1	0	0	1	1
0	0	1	0	0

1	0	0	0	0
1	1	0	0	0
0	1	0	0	0
1	1	1	1	0
1	0	0	0	0
1	1	0	1	0
0	1	1	1	0
0	1	0	0	0
0	1	0	0	0

1	1	0	0	0
1	1	0	0	0
0	0	1	1	1
1	0	0	1	0
0	0	1	1	1
0	0	0	0	0
1	0	0	1	0
1	1	0	1	1
1	0	0	0	0

1	0	0	1	0
0	0	1	0	0
0	0	1	1	1
1	0	0	0	0
0	0	0	1	0
0	0	0	0	0
0	1	0	0	0
1	0	0	0	0
0	1	0	1	0

0	1	1	0	0
1	1	1	1	1
0	0	0	1	1
0	0	0	0	0
1	1	1	0	0
0	0	0	1	0
1	0	0	0	0
1	0	1	0	0
1	0	0	0	0

0	0	0	1	0
0	0	0	1	1
1	0	0	0	1
1	1	1	1	1
1	0	1	0	0
0	0	0	0	0
1	0	0	0	0
1	1	0	0	0
0	1	0	0	0

0	1	0	0	0
0	0	1	0	0
0	0	0	1	0
0	0	0	0	1
0	0	0	0	0
0	0	0	0	0
0	0	0	1	1
0	0	0	1	0
0	1	0	0	0

1	0	0	1	0
1	1	0	0	0
0	0	0	0	1
1	0	1	0	0
1	0	0	1	0
1	0	0	0	0
0	0	0	0	0
0	1	0	0	0
1	0	0	0	0

0	1	0	0	0
1	0	0	0	1
1	1	0	0	0
0	1	0	0	0
1	0	0	1	1
1	0	0	0	0
0	0	1	0	1
0	1	0	0	0
1	0	0	0	0

1	0	0	0	0
0	1	0	0	0
1	0	0	0	0
0	0	0	1	1
0	0	1	1	1
1	1	0	0	0
1	1	0	1	0
0	0	0	0	0
1	1	0	1	0

0	0	0	0	0
1	1	0	0	0
1	0	0	0	0
0	0	0	1	0
0	0	0	0	0
0	1	0	0	1
1	0	0	1	0
0	0	0	0	0
0	0	0	1	0

1	1	0	0	0
1	0	0	0	0
1	0	0	0	0
1	1	0	1	0
1	1	1	1	0
0	0	0	1	1
1	0	0	0	0
1	0	1	0	0
1	0	0	0	0

0	0	0	1	0
0	0	1	1	1
0	0	1	1	0
0	1	1	0	0
1	1	1	0	0
1	0	0	0	0
1	0	0	0	1
0	0	0	0	0
1	1	1	0	0

1	1	0	1	0
1	1	1	0	0
1	0	0	1	0
1	0	0	1	1
1	0	0	1	0
0	0	0	0	0
0	0	0	0	0
0	0	1	0	0
0	0	0	0	0

0	0	0	0	0
0	0	0	0	0
0	0	0	1	0
1	1	1	0	1
1	0	0	0	0
0	1	0	0	0
1	0	0	1	0
0	0	0	0	0
1	0	0	0	0

1	0	0	1	0
0	1	0	0	0
1	0	0	0	0
0	1	0	0	0
0	0	0	0	1
0	0	1	0	0
1	0	1	0	0
0	0	0	1	0
1	0	0	1	1

0	0	0	0	1
1	0	0	0	0
1	0	0	0	0
1	0	0	0	0
1	1	0	0	0
0	1	0	1	0
1	0	0	0	0
1	1	1	0	0
1	0	1	1	0

0	0	0	0	0
1	0	1	1	1

ESTÁENTRATAMIENTOPORCÁNCER	ESTÁENTRATAMIENTOPORPATOLOGÍACARDIOVASCULAR	ESTÁENTRATAMIENTOPORHIPOTIROISDISMO	ESTÁENTRATAMIENTOPORARTRITISOARTROSIS	ESTÁENTRATAMIENTOPORASMA
0	0	0	1	0
0	1	0	0	0
0	0	0	0	0
0	0	0	0	0
0	0	0	1	0
0	0	0	0	0
0	0	0	0	0
0	0	0	0	0

0	0	0	0	0
0	0	0	0	0
0	0	0	1	0
0	0	0	0	0
0	0	0	1	0
0	0	0	0	0
1	1	1	1	0
0	0	0	1	0
0	0	0	0	0

0	0	0	0	0
0	0	0	0	0
0	0	0	0	1
0	0	0	0	0
1	0	0	0	0
0	0	0	1	0
0	0	1	0	0
0	0	1	0	0
0	0	0	1	0

0	0	1	0	0
0	0	0	0	0
0	0	0	0	0
1	0	0	1	0
0	0	0	1	1
0	0	0	0	0
0	0	0	0	0
0	0	0	0	0
0	0	0	0	0

0	0	0	0	0
0	0	0	0	0
0	0	0	0	0
0	0	0	0	0
0	0	0	0	0
0	0	0	0	0
0	0	0	0	0
0	0	0	0	0
0	0	1	1	0

0	0	0	0	0
0	0	0	0	0
0	0	0	0	0
0	0	0	0	0
0	0	0	0	0
0	0	0	0	0
0	0	0	0	0
0	0	0	0	0
0	0	0	1	0

0	1	0	1	0
0	0	0	1	0
0	0	1	1	0
0	0	0	0	0
0	0	0	0	0
0	1	0	1	0
0	0	0	0	0
0	1	0	0	0
0	0	0	0	0

0	0	0	0	1
0	1	0	0	0
0	1	1	1	0
0	0	0	0	0
0	0	0	0	0
0	0	0	0	0
0	0	0	1	0
0	0	0	0	0
0	0	0	1	1

0	0	0	0	0
1	0	0	0	0
0	0	0	0	0
0	0	0	0	0
0	0	0	0	0
0	0	0	0	0
0	0	1	0	0
0	0	0	1	1
0	0	1	1	0

0	0	0	1	0
0	0	1	0	0
0	0	0	0	0
0	0	0	0	0
0	0	0	1	0
0	0	0	0	0
0	0	0	0	0
0	0	0	0	1
0	0	0	1	0

0	0	0	0	0
0	0	0	0	0
0	0	0	0	0
0	0	0	0	0
0	0	0	0	0
0	0	0	0	0
0	0	0	0	0
0	0	0	0	0
0	1	0	0	0

0	0	0	0	0
0	0	0	0	0
0	0	0	0	0
0	1	0	0	0
0	1	0	0	0
0	0	0	0	0
0	0	0	0	0
0	0	0	0	0
0	0	0	0	0

0	0	0	0	0
0	0	0	0	0
0	0	0	0	0
0	0	0	1	1
0	0	0	1	1
0	0	0	1	1
0	0	0	0	0
0	0	0	0	0
0	0	0	0	0

0	0	0	0	0
0	0	0	0	0
0	0	0	1	0
0	0	0	0	0
0	0	1	1	0
0	0	0	1	0
0	1	0	0	0
0	0	0	0	0
0	0	0	0	0

0	0	0	0	0
0	0	0	0	0
0	0	0	0	0
0	0	0	0	0
0	0	0	0	0
0	0	0	0	0
0	0	1	0	0
0	0	0	0	0
0	0	1	1	0

0	0	0	1	0
0	0	0	0	0
0	0	0	0	0
0	0	0	1	1
0	0	1	0	0
0	0	0	0	0
0	0	0	0	0
0	0	0	0	0
0	0	0	0	0

0	0	0	0	0
0	0	0	1	0
0	0	0	0	0
1	0	0	0	0
0	0	0	0	0
0	0	0	0	0
0	0	0	1	0
0	0	0	0	0
0	0	1	1	0

0	0	0	0	0
0	0	0	0	0
0	0	0	0	0
0	0	0	0	0
0	1	0	1	1
0	0	0	0	0
0	0	0	1	0
0	0	0	1	0
0	0	1	0	0

0	0	0	0	0
0	0	0	0	0
0	0	0	0	0
0	0	0	0	0
0	0	0	0	0
0	0	0	0	0
0	0	0	1	0
1	0	0	0	0
0	1	0	0	0

0	0	1	0	0
0	0	0	0	0
0	0	0	1	0
0	0	0	1	0
0	0	0	1	0
0	0	0	0	0
0	0	0	0	0
0	0	0	0	0
0	0	1	0	0

0	0	0	0	0
0	0	0	0	0
0	1	0	0	0
0	0	0	0	0
0	0	0	0	0
0	0	0	0	0
0	0	0	1	0
0	0	0	0	0
0	0	0	0	1

0	0	0	0	0
0	0	0	0	0
1	0	1	0	0
0	1	0	0	0
1	1	0	0	0
0	0	0	0	0
0	0	0	0	0
0	0	1	1	1
0	0	0	0	0

0	1	1	1	0
0	0	0	0	0
0	0	0	0	0
0	0	1	0	0
0	1	0	0	0
0	0	1	0	0
0	0	0	0	0
0	0	0	0	0
0	0	0	0	0

0	0	0	0	0
0	0	0	0	0
0	0	0	0	0
0	0	0	0	0
0	0	0	0	0
0	0	0	0	0
0	0	1	0	0
0	0	0	0	0
0	0	1	0	0

0	0	0	0	0
0	0	1	1	0
0	0	0	0	0
0	0	0	0	0
0	0	0	1	0
0	1	0	1	1
0	0	0	1	0
0	1	0	0	0
0	0	0	0	0

0	1	0	1	0
0	0	0	1	0
0	0	0	0	0
0	0	0	0	0
0	0	0	0	0
0	0	1	0	0
1	0	0	0	0
0	0	0	0	0
0	0	0	0	1

0	0	0	0	0
0	0	0	0	0
0	0	0	0	0
0	0	0	0	1
0	0	0	0	0
0	0	0	0	0
0	0	0	0	0
0	1	1	0	0
0	0	1	0	1

0	0	0	0	0
0	0	0	0	0
0	0	0	1	0
0	0	0	1	0
0	0	0	0	0
0	0	0	0	0
0	0	1	0	0
0	0	0	0	0
0	0	0	0	0

0	0	0	0	0
0	0	0	0	1
0	0	1	1	0
0	1	0	0	0
0	0	0	0	0
0	0	1	0	1
0	0	0	0	0
0	0	1	1	0
0	0	0	0	0

0	0	0	0	0
0	0	0	0	0
0	0	0	0	0
0	0	0	0	0
0	0	0	1	0
0	0	1	0	0
0	1	1	0	0
0	0	0	0	0
0	0	0	0	0

0	0	0	1	1
0	0	0	0	0
0	0	0	0	0
0	0	0	1	0
0	0	0	1	0
0	1	0	1	0
0	0	0	1	1
0	0	1	0	0
0	1	0	0	0

0	1	0	1	1
0	0	0	0	0
0	0	1	1	0
0	1	0	1	1
0	0	0	0	0
0	1	0	0	0
0	0	0	0	0
0	0	0	0	0
0	0	0	0	0

0	0	0	0	0
0	0	0	0	0
0	0	0	0	0
0	0	0	0	0
0	0	0	0	0
0	0	0	0	0
0	0	0	0	0
0	1	1	0	0
0	0	0	1	0

0	0	0	0	0
0	0	0	0	0
0	0	0	0	0
1	1	0	1	0
0	0	1	0	0
0	1	1	0	1
0	1	0	0	0
0	0	0	0	0
0	0	0	0	1

0	0	0	0	0
0	0	0	0	0
1	1	0	1	0
0	0	0	1	0
0	0	0	0	0
0	0	1	0	0
0	0	0	0	0
0	0	1	1	0
0	0	0	1	0

0	0	0	0	0
0	0	0	0	0
0	0	1	0	1
0	0	1	0	0
0	0	0	0	0
0	0	0	0	0
0	0	0	0	0
0	0	0	0	0
0	0	0	0	0

0	1	0	0	0
0	0	0	0	0
1	1	0	0	0
0	0	0	0	0
0	0	0	0	0
0	0	0	0	0
0	0	0	0	0
0	0	0	0	0
0	0	0	0	0

0	0	1	1	0
0	1	0	0	0
0	0	0	0	0
1	0	0	0	0
0	0	0	0	0
0	0	0	0	0
0	0	0	0	0
1	0	1	0	0
0	0	0	0	0

0	0	0	0	0
0	0	0	0	0
1	0	0	0	0
0	0	0	0	0
0	0	0	1	0
0	0	1	1	0
0	0	1	0	0
0	1	0	0	0
0	0	0	0	0

0	0	0	1	0
0	0	0	1	0
0	0	0	1	0
0	0	0	0	0
0	1	0	0	1
0	0	0	1	0
0	0	1	0	0
0	0	0	0	0
0	0	0	0	0

0	0	0	0	0
0	0	0	1	0
0	0	0	0	0
0	0	1	1	0
0	0	0	0	0
0	0	0	0	0
0	0	0	0	0
0	0	0	1	0
0	0	0	0	0

0	0	0	0	0
0	0	0	0	0
0	0	0	0	0
0	1	1	1	0
0	0	0	0	1
0	0	0	0	0
0	1	0	1	0
0	0	1	0	0
0	0	1	1	0

0	0	0	0	0
0	0	0	0	0
0	0	0	0	0
0	0	1	0	0
0	0	0	1	0
1	0	0	0	1
0	0	0	1	0
1	0	0	0	0
0	0	0	1	1

0	0	0	0	0
0	0	0	0	0
0	0	0	0	0
0	1	0	0	1
0	0	0	0	0
0	0	1	1	0
0	0	0	0	0
0	0	0	0	0
0	0	0	0	0

0	0	0	1	0
0	1	0	0	0
0	0	0	0	0
0	0	0	0	0
0	0	0	0	0
0	0	0	0	0
0	0	1	1	0
0	0	0	0	0
0	0	0	0	0

1	0	0	1	0
0	0	0	0	0
0	0	0	1	0
0	1	0	0	0
0	0	0	0	0
0	0	0	1	0
0	0	0	0	0
0	0	0	0	0
0	0	0	0	0

0	0	0	0	0
0	0	0	0	0
0	0	0	0	0
0	0	0	1	0
0	0	0	0	1
0	0	0	0	0
0	0	0	1	0
0	1	0	0	0
0	0	0	0	0

0	0	0	1	0
0	0	0	0	0
0	0	0	0	0
1	0	0	1	0
0	0	0	1	0
0	0	0	1	0
0	0	0	0	0
0	0	0	0	0
0	0	0	1	0

0	0	1	0	0
0	0	0	0	0
0	0	1	0	0
0	0	0	1	0
0	0	0	0	0
0	0	1	1	0
0	0	0	0	0
0	0	0	0	0
0	0	0	0	0

0	0	0	0	0
0	1	1	1	1

SUM_ENFERMEDADES_MEDICAS_ACTUAL	UNA_ENFERM_MED_ACT	HAESTADOENTRATAMIENTOPORHIPERTENSIÓN	HAESTADOENTRATAMIENTOPORDIABETES	HAESTADOENTRATAMIENTOPORCÁNCER
1	1	0	0	0
3	1	1	1	0
1	1	1	0	0
0	1	0	0	0
2	1	1	0	0
0	0	0	0	0
2	1	1	1	0
0	0	0	0	0

0	0	0	0	0
0	0	0	0	0
2	1	1	0	0
0	0	0	0	0
1	1	0	0	0
0	0	0	0	0
4	1	0	0	0
2	1	0	0	0
0	0	0	0	0

0	0	0	0	0
1	1	1	0	0
1	1	0	0	0
0	0	0	0	0
1	1	0	0	1
2	1	0	0	0
1	1	0	0	0
2	1	0	0	0
2	1	0	0	0

1	1	0	0	0
0	0	0	0	0
0	0	0	0	0
3	1	0	0	0
3	1	0	0	0
0	0	0	0	0
1	1	0	0	0
0	0	0	0	0
0	0	0	0	0

0	0	0	0	0
1	1	0	0	0
0	0	0	0	0
1	1	0	0	0
0	0	0	0	0
0	0	0	0	0
0	0	0	0	0
0	0	0	0	0
3	1	0	0	0

1	1	0	0	0
0	0	0	0	0
2	1	0	0	0
0	0	0	0	0
0	0	0	0	0
0	0	0	0	0
0	0	0	0	0
0	0	0	0	0
1	1	0	0	0

2	1	0	0	0
1	1	0	0	0
3	1	1	0	0
0	0	0	0	0
0	0	0	0	0
3	1	0	0	0
0	0	0	0	0
3	1	0	0	0
0	0	0	0	0

2	1	1	0	1
2	1	0	0	0
5	1	0	0	0
0	0	0	0	0
2	1	0	0	0
0	0	0	0	0
2	1	0	0	0
2	1	0	0	0
3	1	0	0	0

1	1	0	0	0
3	1	0	0	0
0	0	0	0	0
1	1	0	0	0
0	0	0	0	0
1	1	0	0	0
1	1	0	0	0
3	1	0	0	0
3	1	0	0	0

3	1	0	0	0
2	1	0	0	0
0	0	0	0	0
2	1	0	0	0
1	1	0	0	0
0	0	0	0	0
1	1	0	0	0
1	1	0	0	0
2	1	0	0	0

0	0	0	0	0
0	0	0	0	0
0	0	0	0	0
1	1	0	0	0
0	0	0	0	0
0	0	0	0	0
0	0	0	0	0
2	1	0	0	0
3	1	0	0	0

0	0	0	0	0
0	0	0	0	0
0	0	0	0	0
1	1	0	0	0
2	1	0	0	0
0	0	0	0	0
1	1	0	0	0
0	0	0	0	0
0	0	0	0	0

0	0	0	0	0
1	1	0	0	0
1	1	0	0	0
3	1	0	0	0
3	1	0	0	0
3	1	0	0	0
0	0	0	0	0
2	1	0	0	0
0	0	0	0	0

0	0	0	0	0
2	1	0	0	0
1	1	0	0	0
0	0	0	0	0
3	1	0	0	0
1	1	0	0	0
2	1	0	0	0
1	1	0	0	0
0	0	0	0	0

0	0	0	0	0
0	0	0	0	0
1	1	0	0	0
0	0	0	0	0
0	0	0	0	0
0	0	0	0	0
2	1	0	0	0
0	0	0	0	0
2	1	0	0	0

2	1	0	0	0
0	0	0	0	0
0	0	0	0	0
2	1	0	0	0
1	1	0	0	0
0	0	0	0	0
0	0	0	0	0
0	0	0	0	0
0	0	0	0	0

0	0	0	0	0
1	1	0	0	0
1	1	0	0	0
1	1	0	0	0
0	0	0	0	0
0	0	0	0	0
2	1	0	0	0
0	0	0	0	0
2	1	0	0	0

0	0	0	0	0
0	0	0	0	0
0	0	0	0	0
0	0	0	0	0
4	1	0	0	0
0	0	0	0	0
1	1	0	0	0
2	1	0	0	0
1	1	0	0	0

0	0	0	0	0
0	0	0	0	0
0	0	0	0	0
0	0	0	0	0
0	0	0	0	0
0	0	0	0	0
2	1	0	0	0
1	1	0	0	0
1	1	0	0	0

1	1	0	0	0
0	0	0	0	0
3	1	0	0	0
2	1	0	0	0
2	1	0	0	0
0	0	0	0	0
0	0	0	0	0
0	0	0	0	0
1	1	0	0	0

0	0	0	0	0
0	0	0	0	0
2	1	0	0	0
1	1	0	0	0
2	1	0	0	0
2	1	0	0	0
1	1	0	0	0
0	0	0	0	0
1	1	0	0	0

0	0	0	0	0
0	0	0	0	0
2	1	0	0	0
1	1	0	0	0
3	1	0	0	0
0	0	0	0	0
0	0	0	0	0
4	1	0	0	0
0	0	0	0	0

4	1	0	0	0
0	0	0	0	0
0	0	0	0	0
1	1	0	0	0
2	1	0	0	0
2	1	0	0	0
0	0	0	0	0
1	1	0	0	0
0	0	0	0	0

0	0	0	0	0
0	0	0	0	0
0	0	0	0	0
1	1	0	0	0
0	0	0	0	0
1	1	0	0	0
1	1	0	0	0
0	0	0	0	0
1	1	0	0	0

0	0	0	0	0
3	1	0	0	0
0	1	0	0	0
0	0	0	0	0
3	1	0	0	0
5	1	0	0	0
2	1	0	0	0
1	1	0	0	0
0	0	0	0	0

2	1	0	0	0
2	1	0	0	0
1	1	0	0	0
0	0	0	0	0
0	0	0	0	0
2	1	0	0	0
1	1	0	0	0
0	0	0	0	0
3	1	0	0	0

0	0	0	0	0
0	0	0	0	0
0	0	0	0	0
2	1	0	0	0
0	0	0	0	0
1	1	0	0	0
2	1	0	0	0
2	1	0	0	0
2	1	0	0	0

1	1	0	0	0
1	1	0	0	0
2	1	0	0	0
1	1	0	0	0
0	0	0	0	0
0	0	0	0	0
3	1	0	0	0
0	0	0	0	0
0	0	0	0	0

0	0	0	0	0
1	1	0	0	0
2	1	0	0	0
3	1	0	0	0
1	1	0	0	0
3	1	0	0	0
0	0	0	0	0
2	1	0	0	0
0	0	0	0	0

0	0	0	0	0
0	0	0	0	0
0	0	0	0	0
0	0	0	0	0
2	1	0	0	0
1	1	0	0	0
3	1	0	0	0
0	0	0	0	0
1	1	0	0	0

3	1	0	0	0
0	0	0	0	0
0	0	0	0	0
2	1	0	0	0
2	1	0	0	0
2	1	0	0	0
2	1	0	0	0
1	1	0	0	0
3	1	0	0	0

4	1	0	0	0
0	0	0	0	0
2	1	0	0	0
5	1	0	0	0
0	0	0	0	0
2	1	0	0	0
0	0	0	0	0
2	1	0	0	0
1	1	0	0	0

0	0	0	0	0
2	1	0	0	0
0	0	0	0	0
0	0	0	0	0
1	1	0	0	0
1	1	0	0	0
0	0	0	0	0
4	1	0	0	0
1	1	0	0	0

0	0	0	0	0
0	0	0	0	0
0	0	0	0	0
4	1	0	0	0
1	1	0	0	0
4	1	0	0	0
2	1	0	0	0
0	0	0	0	0
1	1	0	0	0

0	0	0	0	0
0	0	0	0	0
5	1	0	0	0
2	1	0	0	0
2	1	0	0	0
1	1	0	0	0
1	1	0	0	0
4	1	0	0	0
1	1	0	0	0

1	1	0	0	0
0	0	0	0	0
4	1	0	0	0
1	1	0	0	0
1	1	0	0	0
0	0	0	0	0
0	0	0	0	0
0	0	0	0	0
1	1	0	0	0

1	1	0	0	0
2	1	0	0	0
4	1	0	0	0
0	0	0	0	0
0	0	0	0	0
1	1	0	0	0
0	0	0	0	0
0	0	0	0	0
0	0	0	0	0

3	1	0	0	0
3	1	0	0	0
1	1	0	0	0
4	1	0	0	0
0	0	0	0	0
0	0	0	0	0
0	0	0	0	0
2	1	0	0	0
0	0	0	0	0

0	0	0	0	0
0	0	0	0	0
2	1	0	0	0
1	1	0	0	0
1	1	0	0	0
2	1	0	0	0
3	1	0	0	0
2	1	0	0	0
0	0	0	0	0

2	1	0	0	0
1	1	0	0	0
2	1	0	0	0
0	0	0	0	0
3	1	0	0	0
1	1	0	0	0
1	1	0	0	0
0	0	0	0	0
0	0	0	0	0

0	0	0	0	0
2	1	0	0	0
0	0	0	0	0
2	1	0	0	0
2	1	0	0	0
0	0	0	0	0
1	1	0	0	0
1	1	0	0	0
0	0	0	0	0

0	0	0	0	0
0	0	0	0	0
0	0	0	0	0
5	1	0	0	0
3	1	0	0	0
0	0	0	0	0
3	1	0	0	0
1	1	0	0	0
3	1	0	0	0

0	0	0	0	0
0	0	0	0	0
0	0	0	0	0
2	1	0	0	0
1	1	0	0	0
3	1	0	0	0
2	1	0	0	0
1	1	0	0	0
3	1	0	0	0

0	0	0	0	0
0	0	0	0	0
0	0	0	0	0
3	1	0	0	0
1	1	0	0	0
4	1	0	0	0
0	0	0	0	0
0	0	0	0	0
0	0	0	0	0

2	1	0	0	0
3	1	0	0	0
1	1	0	0	0
0	0	0	0	0
0	0	0	0	0
0	0	0	0	0
3	1	0	0	0
0	0	0	0	0
0	0	0	0	0

3	1	0	0	0
0	0	0	0	0
2	1	0	0	0
3	1	0	0	0
1	1	0	0	0
1	1	0	0	0
0	0	0	0	0
0	0	0	0	0
0	0	0	0	0

0	0	0	0	0
0	0	0	0	0
1	1	0	0	0
2	1	0	0	0
1	1	0	0	0
0	0	0	0	0
2	1	0	0	0
1	1	0	0	0
0	0	0	0	0

2	1	0	0	0
0	0	0	0	0
0	0	0	0	0
2	1	0	0	0
2	1	0	0	0
1	1	0	0	0
0	0	0	0	0
1	1	0	0	0
3	1	0	0	0

2	1	0	0	0
0	0	0	0	0
1	1	0	0	0
1	1	0	0	0
0	0	0	0	0
3	1	0	0	0
0	0	0	0	0
0	0	0	0	0
1	1	0	0	0

0	1	0	0	0
6	1	0	0	0

HAESTADOENTRATAMIENTOPORPATOLOGÍACARDIOVASCULAR	HAESTADOENTRATAMIENTOPORHIPOTIROISDISMO	HAESTADOENTRATAMIENTOPORARTRITISOARTROSIS	HAESTADOENTRATAMIENTOPOROTRAPATOLOGÍA	ALGUIENDESUFAMILIAHATENIDOANTECEDENTESDEDEPRESIÓN
0	0	1	0	0
0	0	0	0	0
0	0	0	0	0
0	0	0	1	0
0	0	1	1	1
0	0	0	0	0
0	0	0	0	0
0	0	0	0	1

0	0	0	1	1
0	0	0	0	0
0	0	1	0	1
0	0	0	0	0
0	0	0	0	1
0	0	0	0	1
0	0	0	1	0
0	0	0	0	1
0	0	0	1	1

0	0	0	0	1
0	0	0	1	0
0	0	0	1	1
0	0	0	1	1
0	0	0	0	0
0	1	0	0	1
0	1	0	0	1
0	0	0	0	0
0	0	0	0	1

0	0	0	0	1
0	0	0	0	1
0	0	0	0	0
0	0	0	0	1
0	0	0	0	1
0	0	0	0	0
0	0	0	0	0
0	0	0	0	1
0	0	0	0	1

0	0	0	0	1
0	0	0	0	0
0	0	0	0	0
0	0	0	0	1
0	0	0	0	1
0	0	0	0	0
0	0	0	0	0
0	0	0	0	0
0	0	0	0	1

0	0	0	0	0
0	0	0	0	0
0	0	0	0	1
0	0	0	0	1
0	0	0	0	1
0	0	0	0	1
0	0	0	0	1
0	0	0	0	0
0	0	0	1	0

1	1	1	0	0
0	0	1	0	0
0	1	0	1	1
0	0	0	0	1
0	0	0	1	0
0	0	0	0	0
0	0	0	0	1
0	0	0	1	1
0	0	0	1	1

0	0	0	1	1
0	0	0	0	1
0	0	0	0	1
0	0	0	0	0
0	0	0	0	1
0	0	0	0	0
0	0	0	0	1
0	0	0	0	1
0	0	0	0	0

0	0	0	0	0
0	0	0	0	1
0	0	0	0	1
0	0	0	0	0
0	0	0	0	0
0	0	0	0	1
0	0	0	0	0
0	0	0	0	0
0	0	0	0	0

0	0	0	0	1
0	0	0	0	1
0	0	0	0	1
0	0	0	0	0
0	0	0	0	1
0	0	0	0	0
0	0	0	0	1
0	0	0	0	1
0	0	0	0	0

0	0	0	0	0
0	0	0	0	1
0	0	0	0	1
0	0	0	0	1
0	0	0	0	1
0	0	0	0	0
0	0	0	0	1
0	0	0	0	0
0	0	0	0	1

0	0	0	0	1
0	0	0	0	1
0	0	0	0	1
0	0	0	0	1
0	0	0	0	1
0	0	0	0	1
0	0	0	0	0
0	0	0	0	0
0	0	0	0	0

0	0	0	0	1
0	0	0	0	1
0	0	0	0	1
0	0	0	0	1
0	0	0	0	1
0	0	0	0	1
0	0	0	0	0
0	0	0	0	1
0	0	0	0	1

0	0	0	0	0
0	0	0	0	1
0	0	0	0	1
0	0	0	0	1
0	0	0	0	1
0	0	0	0	0
0	0	0	0	1
0	0	0	0	0
0	0	0	0	0

0	0	0	0	1
0	0	0	0	1
0	0	0	0	0
0	0	0	0	1
0	0	0	0	1
0	0	0	0	0
0	0	0	0	1
0	0	0	0	1
0	0	0	0	0

0	0	0	0	0
0	0	0	0	1
0	0	0	0	1
0	0	0	0	1
0	0	0	0	1
0	0	0	0	1
0	0	0	0	1
0	0	0	0	1
0	0	0	0	1

0	0	0	0	0
0	0	0	0	1
0	0	0	0	1
0	0	0	0	1
0	0	0	0	1
0	0	0	0	0
0	0	0	0	0
0	0	0	0	1
0	0	0	0	0

0	0	0	0	0
0	0	0	0	0
0	0	0	0	1
0	0	0	0	0
0	0	0	0	1
0	0	0	0	0
0	0	0	0	0
0	0	0	0	1
0	0	0	0	1

0	0	0	0	1
0	0	0	0	0
0	0	0	0	1
0	0	0	0	1
0	0	0	0	0
0	0	0	0	1
0	0	0	0	0
0	0	0	0	0
0	0	0	0	0

0	0	0	0	0
0	0	0	0	1
0	0	0	0	1
0	0	0	0	1
0	0	0	0	1
0	0	0	0	1
0	0	0	0	0
0	0	0	0	1
0	0	0	0	0

0	0	0	0	0
0	0	0	0	1
0	0	0	0	1
0	0	0	0	1
0	0	0	0	1
0	0	0	0	1
0	0	0	0	0
0	0	0	0	0
0	0	0	0	1

0	0	0	0	1
0	0	0	0	0
0	0	0	0	0
0	0	0	0	0
0	0	0	0	0
0	0	0	0	0
0	0	0	0	0
0	0	0	0	0
0	0	0	0	1

0	0	0	0	0
0	0	0	0	0
0	0	0	0	0
0	0	0	0	1
0	0	0	0	1
0	0	0	0	0
0	0	0	0	1
0	0	0	0	0
0	0	0	0	0

0	0	0	0	1
0	0	0	0	0
0	0	0	0	0
0	0	0	0	0
0	0	0	0	0
0	0	0	0	0
0	0	0	0	1
0	0	0	0	1
0	0	0	0	1

0	0	0	0	1
0	0	0	0	0
0	0	0	0	0
0	0	0	0	1
0	0	0	0	1
0	0	0	0	1
0	0	0	0	1
0	0	0	0	0
0	0	0	0	0

0	0	0	0	1
0	0	0	0	0
0	0	0	0	0
0	0	0	0	1
0	0	0	0	1
0	0	0	0	1
0	0	0	0	0
0	0	0	0	0
0	0	0	0	0

0	0	0	0	0
0	0	0	0	0
0	0	0	0	0
0	0	0	0	1
0	0	0	0	1
0	0	0	0	0
0	0	0	0	0
0	0	0	0	0
0	0	0	0	1

0	0	0	0	0
0	0	0	0	1
0	0	0	0	0
0	0	0	0	1
0	0	0	0	1
0	0	0	0	1
0	0	0	0	0
0	0	0	0	1
0	0	0	0	0

0	0	0	0	0
0	0	0	0	0
0	0	0	0	0
0	0	0	0	0
0	0	0	0	1
0	0	0	0	1
0	0	0	0	0
0	0	0	0	1
0	0	0	0	1

0	0	0	0	1
0	0	0	0	1
0	0	0	0	1
0	0	0	0	1
0	0	0	0	1
0	0	0	0	1
0	0	0	0	1
0	0	0	0	1
0	0	0	0	0

0	0	0	0	0
0	0	0	0	0
0	0	0	0	1
0	0	0	0	1
0	0	0	0	0
0	0	0	0	0
0	0	0	0	1
0	0	0	0	1
0	0	0	0	1

0	0	0	0	1
0	0	0	0	1
0	0	0	0	1
0	0	0	0	0
0	0	0	0	1
0	0	0	0	1
0	0	0	0	1
0	0	0	0	1
0	0	0	0	1

0	0	0	0	1
0	0	0	0	0
0	0	0	0	0
0	0	0	0	0
0	0	0	0	1
0	0	0	0	1
0	0	0	0	0
0	0	0	0	1
0	0	0	0	0

0	0	0	0	1
0	0	0	0	1
0	0	0	0	0
0	0	0	0	1
0	0	0	0	1
0	0	0	0	1
0	0	0	0	0
0	0	0	0	0
0	0	0	0	0

0	0	0	0	1
0	0	0	0	1
0	0	0	0	0
0	0	0	0	1
0	0	0	0	0
0	0	0	0	0
0	0	0	0	1
0	0	0	0	1
0	0	0	0	1

0	0	0	0	1
0	0	0	0	0
0	0	0	0	1
0	0	0	0	1
0	0	0	0	0
0	0	0	0	0
0	0	0	0	0
0	0	0	0	1
0	0	0	0	0

0	0	0	0	0
0	0	0	0	1
0	0	0	0	0
0	0	0	0	1
0	0	0	0	1
0	0	0	0	0
0	0	0	0	1
0	0	0	0	1
0	0	0	0	1

0	0	0	0	0
0	0	0	0	0
0	0	0	0	1
0	0	0	0	0
0	0	0	0	1
0	0	0	0	0
0	0	0	0	1
0	0	0	0	1
0	0	0	0	0

0	0	0	0	0
0	0	0	0	0
0	0	0	0	0
0	0	0	0	0
0	0	0	0	0
0	0	0	0	0
0	0	0	0	0
0	0	0	0	1
0	0	0	0	0

0	0	0	0	1
0	0	0	0	1
0	0	0	0	0
0	0	0	0	0
0	0	0	0	1
0	0	0	0	1
0	0	0	0	0
0	0	0	0	0
0	0	0	0	1

0	0	0	0	1
0	0	0	0	1
0	0	0	0	1
0	0	0	0	0
0	0	0	0	1
0	0	0	0	1
0	0	0	0	1
0	0	0	0	0
0	0	0	0	1

0	0	0	0	1
0	0	0	0	0
0	0	0	0	1
0	0	0	0	0
0	0	0	0	0
0	0	0	0	1
0	0	0	0	1
0	0	0	0	0
0	0	0	0	1

0	0	0	0	0
0	0	0	0	1
0	0	0	0	1
0	0	0	0	0
0	0	0	0	0
0	0	0	0	0
0	0	0	0	1
0	0	0	0	0
0	0	0	0	1

0	0	0	0	1
0	0	0	0	1
0	0	0	0	1
0	0	0	0	1
0	0	0	0	1
0	0	0	0	0
0	0	0	0	1
0	0	0	0	1
0	0	0	0	1

0	0	0	0	0
0	0	0	0	0
0	0	0	0	0
0	0	0	0	1
0	0	0	0	1
0	0	0	0	1
0	0	0	0	1
0	0	0	0	0
0	0	0	0	1

0	0	0	0	1
0	0	0	0	1
0	0	0	0	1
0	0	0	0	1
0	0	0	0	1
0	0	0	0	0
0	0	0	0	1
0	0	0	0	1
0	0	0	0	0

0	0	0	0	0
0	0	0	0	0
0	0	0	0	0
0	0	0	0	1
0	0	0	0	0
0	0	0	0	0
0	0	0	0	1
0	0	0	0	1
0	0	0	0	1

0	0	0	0	1
0	0	0	0	0
0	0	0	0	1
0	0	0	0	1
0	0	0	0	0
0	0	0	0	1
0	0	0	0	1
0	0	0	0	0
0	0	0	0	1

0	0	0	0	0
0	0	0	0	1
0	0	0	0	1
0	0	0	0	1
0	0	0	0	1
0	0	0	0	0
0	0	0	0	1
0	0	0	0	1
0	0	0	0	1

0	0	0	0	0
0	0	0	0	1

ALGUIENDESUFAMILIAHATENIDOANTECEDENTESDEALCOHOLISMO	ALGUIENDESUFAMILIAHATENIDOANTECEDENTESDEPSICOSIS	ALGUIENDESUFAMILIAHATENIDOANTECEDENTESDEDEMENCIA	ALGUIENDESUFAMILIAHATENIDOANTECEDENTESDEDEPENDENCIAA	ALGUIENDESUFAMILIAHATENIDOANTECEDENTESDEBIPOLARIDAD
1	0	0	0	0
1	0	0	0	0
0	0	0	0	1
0	0	0	0	0
0	0	0	0	0
1	0	0	0	0
0	0	1	0	0
0	0	0	0	0

1	0	0	0	0
0	0	0	0	0
1	0	0	0	0
0	0	0	0	0
1	0	0	0	0
0	0	0	0	0
0	0	0	0	0
1	0	0	1	0
1	0	0	0	0

1	0	0	0	0
0	0	0	0	0
1	0	0	0	0
1	1	1	1	1
0	0	0	0	1
1	0	1	0	0
0	0	0	0	0
0	0	0	0	0
0	0	0	0	0

1	0	0	1	0
0	0	0	0	1
0	0	0	0	0
1	0	0	0	0
0	0	0	0	0
1	0	0	0	0
0	0	0	0	0
1	0	0	1	0
0	0	0	0	0

0	1	0	0	0
1	1	0	0	0
0	0	0	0	0
0	0	0	0	0
0	1	0	0	0
1	0	0	0	0
0	0	0	0	0
1	0	0	0	0
0	0	0	0	1

0	0	0	0	0
0	0	0	0	0
1	0	0	0	0
0	0	0	1	0
0	0	0	0	0
0	0	1	0	0
1	0	1	0	1
0	0	0	1	0
0	0	0	0	0

1	0	0	1	0
0	0	0	0	0
0	1	0	0	1
0	0	0	0	0
0	0	0	0	1
0	0	0	0	0
0	0	0	0	0
1	1	0	0	0
1	0	0	1	0

1	0	0	0	0
1	0	0	1	0
1	0	0	0	0
0	0	0	0	0
0	0	1	0	0
0	0	0	0	0
0	0	1	0	0
1	0	0	0	0
0	0	0	0	0

1	0	0	1	0
1	1	0	0	1
0	0	0	0	1
1	0	0	0	0
1	0	0	0	0
0	1	1	0	0
0	0	0	0	0
0	0	0	0	0
1	0	0	1	0

0	0	0	0	0
1	1	0	0	0
0	0	0	0	0
0	0	0	0	0
1	0	0	0	0
1	0	1	1	0
0	0	0	1	0
1	1	1	1	0
1	0	0	0	0

0	0	0	0	0
1	0	1	0	0
0	0	0	1	0
0	0	0	0	0
0	0	0	0	0
1	0	0	0	0
1	1	0	0	0
0	0	0	0	0
1	0	0	0	1

0	0	1	0	1
1	1	0	1	0
0	0	0	0	0
0	0	0	0	0
1	0	0	0	0
1	1	1	1	0
1	0	0	0	0
0	0	0	0	0
1	0	1	1	1

1	0	0	1	0
0	0	0	0	0
0	0	1	0	0
1	0	0	1	1
1	1	0	1	0
1	0	0	0	1
1	0	0	0	0
0	0	0	0	0
1	1	0	1	1

0	0	0	0	0
0	0	0	0	0
0	0	0	0	0
0	0	0	0	0
1	0	0	1	1
0	0	0	0	0
1	0	0	1	0
1	0	0	0	0
0	0	0	0	0

1	1	0	0	1
1	0	0	0	0
1	0	0	0	0
0	0	0	0	1
1	1	0	1	1
1	0	1	1	0
1	0	1	0	1
0	0	0	1	0
0	0	0	0	0

0	0	0	1	0
1	1	0	1	0
1	0	0	0	0
1	1	1	1	0
1	0	0	0	0
1	0	0	0	0
0	0	0	0	0
0	0	0	0	0
1	0	0	0	0

0	0	0	0	0
0	0	0	0	0
1	0	0	1	0
0	0	1	0	0
1	0	0	1	0
1	0	0	1	0
0	0	0	0	0
1	0	1	0	0
0	0	1	0	0

0	0	0	0	0
0	0	0	0	0
1	0	0	1	0
0	0	0	0	0
0	0	1	0	0
0	1	1	0	0
0	0	1	0	0
1	0	1	0	0
1	0	0	1	1

1	0	0	0	1
1	0	0	0	0
0	0	0	0	0
0	0	0	1	0
1	0	0	0	0
0	0	0	0	0
1	0	1	0	0
1	0	0	0	1
1	1	0	0	0

0	0	0	0	0
1	0	0	0	1
0	0	0	0	0
1	0	1	0	0
0	0	0	0	1
1	0	0	0	0
1	0	0	0	0
0	0	0	0	0
0	0	1	0	0

1	0	1	0	1
0	0	0	0	0
1	0	0	0	0
1	0	0	0	0
1	0	1	0	0
1	0	0	0	0
0	0	1	0	0
0	0	0	0	0
0	0	0	0	1

1	0	1	0	1
1	0	0	0	0
0	0	0	0	0
1	0	0	0	0
0	0	0	0	0
1	0	0	0	0
0	0	1	0	0
0	0	0	0	0
0	0	0	0	0

1	1	0	0	0
0	0	0	0	0
0	0	0	1	0
1	0	0	0	0
1	1	0	1	0
0	0	0	0	0
1	1	0	1	1
0	1	0	0	0
1	0	0	1	0

0	0	0	0	0
0	0	0	0	0
0	0	0	0	0
0	0	0	0	0
1	0	0	0	0
1	0	0	0	0
1	0	1	1	0
0	0	0	0	0
1	0	0	0	0

0	0	0	0	0
0	0	0	0	0
0	0	0	0	0
1	0	1	1	0
1	1	0	0	0
0	0	0	1	0
0	1	0	0	0
0	0	0	1	1
0	0	0	0	0

1	0	0	0	0
0	0	0	0	0
0	0	1	0	0
0	1	1	0	0
1	0	0	0	0
1	0	0	0	0
0	0	1	0	0
1	0	1	1	0
1	0	0	0	0

0	0	0	0	0
1	0	1	0	0
0	0	0	0	0
1	1	0	0	0
1	0	0	0	0
0	0	0	0	0
0	0	0	0	0
0	0	0	0	0
0	0	0	0	0

0	0	0	0	0
0	0	0	0	0
0	0	0	0	0
1	0	0	0	0
1	0	0	1	1
0	0	0	0	0
0	0	0	0	0
1	0	0	0	0
1	0	1	0	0

1	0	0	0	0
1	0	0	1	0
1	0	1	1	0
0	0	0	0	0
1	0	0	1	0
1	1	1	0	0
1	0	0	1	0
1	1	0	0	1
0	0	0	0	0

0	0	0	0	0
1	0	1	0	0
0	0	0	0	0
1	0	0	0	0
1	0	0	0	1
0	0	1	0	1
0	0	0	0	0
0	0	0	0	0
0	0	1	0	0

1	0	0	0	0
1	0	0	0	0
0	0	0	0	0
1	0	0	1	0
1	0	0	0	0
1	0	0	0	0
1	0	0	0	0
1	0	0	0	0
1	0	0	0	0

1	0	0	0	0
1	0	0	0	0
1	0	0	0	0
0	0	0	0	0
0	0	0	0	0
1	0	0	0	0
0	0	0	0	0
0	0	0	0	0
1	0	0	0	0

1	0	1	0	0
1	0	0	1	0
1	0	0	1	1
0	0	1	0	0
1	1	0	0	0
0	0	0	0	0
1	0	0	0	1
0	0	0	0	0
1	0	0	1	0

0	0	0	0	0
1	1	0	1	1
0	0	0	1	0
1	0	0	1	0
0	0	0	1	0
0	0	0	0	0
1	0	0	0	0
1	0	0	0	0
0	0	0	0	0

0	0	0	0	0
0	0	0	0	0
0	0	0	0	0
1	0	0	0	0
0	0	0	0	0
0	0	0	0	0
0	0	0	0	0
0	0	0	0	0
0	0	1	0	0

1	0	0	1	0
1	0	0	0	0
1	0	0	1	0
1	0	0	0	0
1	0	1	0	0
1	0	0	0	0
1	0	0	1	0
0	0	1	0	0
0	0	0	0	0

1	0	0	0	0
0	0	0	0	1
0	0	0	0	0
0	0	0	0	0
1	0	0	0	0
0	0	0	0	0
1	0	0	0	0
0	0	0	0	0
1	1	0	0	0

0	0	0	0	0
0	0	0	0	0
0	0	0	0	0
0	0	0	0	0
0	0	0	1	0
1	1	0	0	0
0	0	0	0	0
1	0	0	0	0
0	0	0	0	0

0	0	0	1	0
0	0	0	0	0
0	0	0	0	0
1	0	0	0	0
0	0	0	0	0
1	0	0	0	0
1	0	0	0	0
1	0	0	0	0
0	1	0	0	0

1	0	0	0	0
0	0	0	0	0
1	0	0	0	0
1	1	0	1	0
1	0	0	0	0
1	0	0	0	0
0	0	0	0	0
0	0	1	0	0
1	0	0	1	0

0	0	0	0	0
0	0	0	0	0
0	0	1	0	0
0	0	0	0	0
1	0	0	0	0
1	0	1	0	0
1	0	0	0	0
1	0	1	0	0
0	0	0	0	1

1	0	0	0	0
1	0	0	1	0
0	0	0	1	1
1	0	0	0	0
1	0	0	0	0
1	0	1	0	0
0	0	0	0	0
0	0	0	0	0
0	0	0	0	0

1	0	0	0	0
1	0	1	1	1
1	0	0	0	0
0	0	0	0	0
1	0	1	0	0
0	0	0	0	0
0	0	0	0	0
0	0	0	0	0
1	0	1	0	0

0	0	1	0	0
0	0	0	1	0
0	0	0	0	0
0	1	0	0	0
1	0	0	0	0
0	0	0	0	0
1	0	0	0	0
0	0	0	0	0
0	0	0	0	0

0	0	0	0	0
1	0	0	0	0
0	0	0	0	0
1	0	0	0	0
0	0	1	0	0
0	0	0	0	0
0	0	0	0	0
1	1	0	0	0
1	0	0	1	0

0	0	0	0	0
1	0	0	1	1
0	0	0	0	0
1	0	0	0	0
0	0	0	0	0
0	0	1	0	0
1	0	0	0	0
0	0	0	0	0
0	0	0	0	0

0	1	0	1	0
0	0	0	0	0
1	0	0	0	0
1	0	1	0	0
1	1	0	0	0
0	0	0	0	0
1	1	1	1	1
0	0	0	0	0
1	0	0	0	0

0	0	0	0	0
1	0	0	0	0
0	0	0	0	0
1	0	0	0	0
0	0	1	1	0
1	1	0	0	0
1	0	0	0	0
0	0	0	0	0
0	0	0	0	0

1	0	0	0	0
0	0	0	0	0
1	0	0	0	0
0	1	0	0	0
1	0	0	0	0
0	0	0	0	0
1	0	0	0	0
1	0	0	0	1
0	1	0	0	0

0	0	0	0	0
1	0	0	1	0

ALGUIENDESUFAMILIAHATENIDOANTECEDENTESDEPÁNICO	ALGUIENDESUFAMILIAHATENIDOANTECEDENTESDEOTRO	ALGUIENDESUFAMILIAHATENIDOANTECEDENTESDEHIPERTENSIÓN	ALGUIENDESUFAMILIAHATENIDOANTECEDENTESDEDIABETES	ALGUIENDESUFAMILIAHATENIDOANTECEDENTESDECÁNCER
0	0	0	1	0
0	0	1	0	0
0	0	0	0	0
0	0	0	0	0
0	0	1	1	0
0	0	0	0	1
0	0	1	1	0
0	0	1	0	0

0	0	0	1	0
0	0	1	1	0
0	0	1	1	1
0	0	0	0	1
0	0	1	1	1
1	0	1	0	1
0	0	1	1	1
0	0	0	0	0
0	0	1	1	0

0	0	1	0	0
0	0	1	1	1
0	0	0	0	0
1	0	1	1	0
0	0	1	1	1
0	0	1	1	0
0	0	1	1	0
0	0	1	1	0
0	0	0	1	0

0	0	1	1	1
0	0	1	1	0
0	0	0	0	1
0	0	0	1	0
0	0	1	1	1
0	0	0	0	0
0	0	1	1	0
0	0	1	0	1
1	0	0	1	1

0	0	1	1	0
0	0	0	0	0
0	0	0	1	1
0	0	1	1	1
0	0	1	0	1
0	0	1	0	0
0	0	1	1	0
0	0	0	0	0
0	0	1	0	1

0	0	1	0	1
0	0	1	1	0
0	0	1	1	1
1	0	1	1	0
1	0	0	1	0
0	0	1	0	1
0	0	0	0	1
0	0	0	1	0
0	0	0	1	1

0	1	0	0	0
0	0	1	0	0
0	0	1	1	0
1	0	1	1	0
0	1	0	0	0
0	0	0	1	0
0	0	1	0	1
0	0	0	0	0
1	0	1	0	1

1	0	1	0	1
0	0	0	0	1
0	0	1	1	1
0	0	1	0	0
0	0	1	1	0
1	0	1	0	0
0	0	1	0	0
0	0	0	1	1
0	0	1	1	0

0	0	1	1	1
0	0	1	1	1
0	0	1	1	1
0	0	0	0	0
0	0	1	1	0
1	0	1	1	0
0	0	1	0	1
0	0	0	0	0
1	0	1	0	1

0	0	1	1	0
1	0	1	0	0
0	1	1	1	0
0	0	1	0	0
1	0	1	1	1
0	0	1	1	1
0	0	0	0	0
1	0	1	1	0
0	0	1	0	0

0	0	1	1	1
1	0	0	0	1
1	1	0	1	0
0	0	1	1	0
0	0	1	1	1
0	0	1	1	1
0	0	1	1	1
0	0	1	0	0
0	0	1	0	0

1	0	0	1	1
1	0	1	1	0
0	0	1	1	0
0	0	1	1	0
0	0	1	0	0
1	0	1	1	0
0	0	1	1	1
0	0	1	1	0
1	0	1	1	1

0	0	1	1	1
1	0	1	1	1
1	0	1	1	0
0	0	1	0	0
0	0	1	1	0
0	0	1	0	1
0	0	1	1	0
0	0	1	0	0
1	0	1	0	1

0	0	1	1	0
0	0	1	1	1
1	0	0	0	0
0	0	0	0	0
0	0	0	1	0
0	0	0	0	1
0	0	1	0	0
0	0	0	0	0
0	0	0	0	1

0	0	1	1	1
0	0	1	1	1
0	0	1	1	0
0	0	1	1	1
0	0	1	0	0
0	0	1	1	0
1	0	1	0	0
0	0	1	1	1
0	0	1	1	1

0	0	1	1	1
0	0	0	1	1
0	0	0	0	1
0	0	1	1	1
0	0	1	1	1
0	0	1	1	0
1	0	1	1	0
1	1	1	1	1
1	0	1	0	0

0	0	1	1	0
0	1	1	1	0
0	0	1	1	1
1	1	0	0	1
0	1	1	0	0
0	0	1	0	0
0	0	0	0	1
0	0	1	1	1
0	0	1	1	0

0	0	1	0	1
0	0	1	1	1
0	0	1	0	0
0	0	1	1	1
0	0	1	0	1
1	0	1	0	0
1	0	1	0	1
0	0	1	0	0
1	0	0	1	1

1	1	1	1	1
0	1	1	1	0
0	0	0	0	1
0	0	0	0	0
0	1	1	1	0
0	0	0	1	0
0	0	1	1	0
0	0	0	1	0
0	1	0	1	0

0	0	1	1	0
0	0	0	0	0
0	0	1	1	1
1	0	1	1	1
0	0	1	1	1
0	1	0	1	0
0	0	0	1	1
0	0	1	1	1
0	0	1	1	0

0	0	1	1	1
0	0	0	1	0
0	0	1	1	0
0	0	1	1	0
0	0	1	1	0
0	1	1	1	1
0	0	1	1	1
0	0	0	1	0
1	0	1	1	1

0	0	1	1	1
1	0	1	0	0
0	0	0	1	0
0	0	0	0	0
0	0	1	0	0
0	0	1	1	0
0	0	1	1	0
0	0	1	0	1
0	0	0	1	1

0	0	0	1	1
0	0	0	0	0
0	0	0	0	0
1	0	1	0	0
0	0	1	0	0
0	0	1	0	1
0	0	1	1	1
0	0	1	1	1
0	0	0	0	1

0	0	1	0	0
0	0	1	0	1
0	0	0	1	0
0	0	0	1	1
0	0	1	1	0
0	0	0	1	0
0	0	1	0	1
0	0	1	1	1
0	0	0	0	0

0	0	0	0	0
0	0	0	1	1
0	0	0	0	0
0	0	0	0	0
0	0	0	0	0
1	0	1	1	1
0	0	1	0	0
0	0	1	1	1
0	0	0	1	0

0	0	1	0	1
0	0	0	0	0
0	0	1	1	0
0	0	1	0	0
0	0	1	0	0
0	0	1	0	0
0	0	0	0	0
0	0	0	0	0
0	0	1	0	1

0	0	0	1	0
0	0	1	1	0
0	0	0	0	0
0	0	1	1	0
0	0	0	0	0
0	0	1	1	0
0	0	1	1	0
1	0	1	1	1
1	0	1	0	1

0	0	1	1	0
0	0	1	1	1
0	0	1	0	0
1	0	1	1	1
0	0	0	0	0
0	0	0	1	0
0	0	1	0	0
1	0	1	1	1
0	0	1	1	1

0	0	0	1	1
0	0	1	1	0
0	0	1	1	0
1	0	1	1	0
0	0	1	1	0
1	1	1	1	1
0	0	0	0	0
0	1	1	1	1
0	0	1	1	1

0	0	0	0	1
0	0	1	1	0
1	0	0	0	1
0	0	1	1	0
0	0	0	1	1
0	0	1	1	0
1	0	1	1	1
0	0	0	0	0
0	0	1	1	1

0	0	0	0	0
0	0	0	0	0
0	0	0	1	0
0	1	1	0	1
0	0	1	0	1
0	0	1	1	0
0	1	0	0	1
0	0	1	1	1
0	0	1	1	0

0	0	1	0	1
1	0	1	1	1
0	0	1	1	1
0	0	0	1	1
0	0	1	0	1
0	0	1	1	0
0	0	1	0	1
0	0	0	0	0
0	0	1	1	1

1	0	1	1	1
0	0	1	0	0
0	0	1	1	1
0	0	1	0	0
0	0	1	0	1
0	0	1	1	1
1	1	1	1	1
0	0	1	1	0
0	0	1	1	0

0	0	1	1	0
1	1	0	1	1
0	0	1	0	0
0	0	1	0	1
1	0	0	0	0
0	0	1	0	1
0	0	1	1	0
0	0	1	1	0
0	0	1	0	0

0	0	1	1	1
0	0	0	0	1
0	0	0	0	0
0	0	1	1	1
0	0	1	1	0
0	0	1	0	1
0	0	0	1	1
0	0	0	0	0
0	0	1	0	1

0	0	1	0	1
0	0	1	1	1
0	0	1	1	1
0	0	1	0	1
0	0	1	1	0
0	0	1	1	1
0	0	1	1	1
0	0	1	1	1
0	0	1	0	1

0	0	1	1	0
0	0	0	1	1
0	0	1	1	1
0	0	1	0	0
0	0	1	1	1
0	0	0	0	0
0	0	1	0	1
0	0	1	1	0
0	0	1	1	1

0	0	1	0	0
0	0	0	1	1
0	0	0	0	1
0	0	0	0	0
1	0	1	1	0
0	0	0	0	0
0	0	0	0	1
0	0	1	1	1
0	0	0	0	0

0	0	1	1	1
0	0	1	1	1
0	0	1	1	1
0	0	0	1	1
0	0	1	0	1
0	0	0	0	1
0	0	0	0	0
0	0	0	0	1
0	0	1	0	0

0	0	1	1	0
1	0	1	1	1
0	0	0	0	1
0	1	1	0	0
0	0	1	1	0
0	0	1	0	1
0	0	1	0	0
1	0	0	0	1
0	0	1	1	1

0	0	0	0	0
0	0	0	1	0
0	0	1	0	0
0	0	0	1	0
1	0	1	1	1
0	0	0	0	0
0	0	1	1	0
0	0	1	1	1
0	0	0	1	1

0	1	1	0	0
0	0	1	1	0
0	0	0	0	1
0	0	1	1	1
0	0	1	1	1
0	0	1	1	1
0	0	0	0	0
0	0	1	0	0
0	0	1	0	0

0	0	0	0	1
1	0	1	1	1
1	0	1	1	0
0	0	0	0	1
0	0	1	0	0
0	0	0	0	0
0	0	1	1	0
0	0	0	0	1
0	0	0	0	0

0	0	1	1	0
0	0	1	1	0
0	0	1	1	1
0	0	1	1	1
0	0	1	1	1
0	0	1	0	1
0	0	1	0	1
0	0	1	1	1
0	0	1	1	0

0	0	1	0	1
0	0	0	0	0
1	0	0	0	1
0	0	0	1	0
0	0	1	1	1
0	0	0	1	0
0	0	1	0	0
0	0	0	0	1
1	0	1	1	1

0	0	1	1	0
1	0	1	1	1
0	0	0	0	1
0	0	1	1	1
0	0	1	1	0
0	1	0	0	0
0	0	1	0	1
1	0	0	0	0
0	0	1	1	0

0	0	1	1	1
0	0	0	0	0
0	0	0	0	0
0	0	0	1	0
0	0	1	1	0
0	0	1	1	1
1	0	1	1	0
0	0	0	0	1
0	0	1	1	1

0	0	1	0	0
0	0	1	1	0
0	0	1	1	0
1	0	1	1	1
1	0	0	1	1
0	0	1	1	0
0	0	0	0	1
0	0	1	0	1
0	0	1	1	1

0	0	1	0	0
0	0	1	0	0
0	0	0	0	0
1	0	1	1	1
0	0	1	0	1
0	0	0	0	0
0	0	1	1	0
0	0	1	1	0
0	0	1	1	1

0	0	0	0	0
1	0	1	1	0

ALGUIENDESUFAMILIAHATENIDOANTECEDENTESDEPATOLOGÍACAR	ALGUIENDESUFAMILIAHATENIDOANTECEDENTESDEHIPOTIROISDISM	ALGUIENDESUFAMILIAHATENIDOANTECEDENTESDEARTRITISOART	ALGUIENDESUFAMILIAHATENIDOANTECEDENTESDEOTRAPATOLOGÍ	FÁRMACOSTRATAMIENTOMÉDICOQUEESTÁCONSUMIENDOALINGRESO
0	0	0	0	
1	0	0	1	10 med
0	0	0	0	arorvastatina enalapril hidroclo
0	0	0	0	tensodox
1	0	1	1	inhaladores salbutamol clorfenanmina calcio info paracetamol
0	0	0	0	sertralina
0	0	0	0	losartan metformina
0	0	0	1	

0	0	0	0	antidepresivo
0	0	0	1	fluoxetina, clorpromazina 25 mg
1	0	1	1	fluoxetina, amitriptilina
0	0	1	0	no
1	0	1	0	Fluoxetina y clonazepam 2 mg
0	1	1	1	Anticonceptivo
1	1	1	1	enalpril 5 digoxina aspirina eutirox 100
0	0	0	0	
0	0	0	0	

1	1	0	0	no
0	0	0	0	hidroclorotiazida
0	0	0	0	fluoxetina antialergicos
0	0	1	0	fluoxetina clorpromazina
1	1	1	0	amitriptilina
1	0	1	1	hidrocllorotizazida
0	0	0	0	eutirox
1	0	0	0	metformina glibenclamida para el colesterol
0	0	0	0	enlapril y colesterol y calciio

1	1	0	0	sertralina50 eutirox
0	0	0	1	fluoxetina 20 amitrip25
0	0	0	0	fluox y amitrip
0	0	0	0	venlafaxina 75 mg clona 1 seoquel xr50 antihi
1	0	1	0	
0	0	0	0	no
1	1	1	1	losartan 50 mg al dia
1	0	0	0	np
1	1	0	0	aco.

1	1	0	0	crema para los hongos al 1% dermoxil
0	0	0	0	losartan 100 mg por dia
0	0	0	0	aco
0	1	0	0	enalapril 10 mg 1 cp mañana y otra en la noche
0	0	0	0	carbamazepina 100 mg por noche indicado por maxilofacial
1	1	1	0	no
0	0	0	0	Gemfibrozilo y atorvastatina
0	0	0	0	0
0	0	0	0	metotrexato, prednisona, celecoxbib, paracetamol

0	1	0	0	enalapril, hidroclorotiazida
0	0	0	0	-
1	1	1	0	venlafaxina 75 mg
0	0	0	1	no
0	0	0	1	no
0	0	0	1	tensoliv, omeprazol, alprazolam
0	0	0	0	Diazepam 10 mg/noche
0	0	0	1	quetiapina 100 mg/noche, Clonazepam 2 mg/noche
1	0	1	0	Clonazepam 2 mg/noche

0	0	0	1	Sertralina 100 mg 1, clonazapam 2 mg 1 en la noche
0	0	1	0	1000 MG/D, TRAMADOL 1/D, OMEPRAZOL 40 MG/D, PROPANOLOL 20
1	0	1	0	MG/D, HIDROCLOROTIAZIDA 1/D, ENALAPRIL 20' MG/D, EUTIROX 50 MG/D, ASPIRINA
1	1	0	0	CLONAZEPAM 2 MG/N
0	1	0	1	SERTRALINA 100 MG 1 AL DÍA, AMIPTRIPTILINA 25 MG 1 EN LA NOCHE
1	0	1	0	FLUOXETINA, 1 AL DIA, CLORDIAZEPOXIDO 1 NOCHE
1	0	0	0	CITALOPRAM 20 MG, ALPRAZOLAM 0,5
0	0	0	1	SERTRALINA 100 MG
1	0	0	0	SERTRALINA 100 MGRS, CLONAZEPAM 2 MGRS

1	0	0	0	
0	0	0	0	FLUOXETINA, CLONAZEPAM
1	1	0	0	clonazepam 2 mg. 0,5
0	0	0	0	PREGABALINA, CITALOPRAN
0	0	0	0	flunarizina 10 mgrs c/12 hrs, metformina 850mgrs, enalapril 20 mgrs, nifedipino
0	1	0	0	clonazepam, fluoxetina
0	0	1	0	VENLAFAXINA, ALPRAZOLAN,
0	0	0	0	enalapril, omeprazol
0	1	1	0	Atorvastatina, trimebutino, omeprazol, paracetamol (SOS)

0	0	0	0	enalapril, atenolol, hidroclorotiazida, aspirina atorvastatina
0	0	1	0	metformina, losartan, tramadol, aspirina, insulina
0	0	1	0	ninguno
0	0	0	0	HIDROCLOROTIAZIDA, GEMFIBROZILO
1	0	1	0	
0	0	1	0	LOSARTAN, ASPIRINA
1	0	0	0	eutirox 200 mg/d, atorvastatina 20 mg/noche
0	0	0	0	enalapril 20 mg/d, paracetamol 500 mg/d
0	1	1	0	enalapril 20 mg/d, levotiroxina 50 mg/d y paracetamol 500 mg/d

1	0	1	0	GLUCOFAGE, LOZARTAN,SERTRALINA 100, 1 AL DÍA, CLONAZEPAM 0,5
1	0	1	0	NO LOS RECUERDA
0	1	1	0	CLONAZEPAM 0,5 MG
0	0	0	1	ENALAPRIL, INSULINA, ATORVASTATINA, DIAZEPAM
1	1	1	1	PARACETAMOL, DICLOFENACO, METATREXATO,ACIDO FOLICO, CLONAZEPAM
0	0	1	1	CLONAZEPAM 2MG, RIZE, RISPERIDONA
1	0	1	0	lozartan
1	0	1	1	paracetamol, propanolol
0	0	0	0	clonazepam, amiptriptilina, omeprazol, celebra, paracetamol

0	0	0	0	Alprazolam
1	0	0	0	SI
0	1	0	1	sertralina, amitriptilina
0	0	0	0	alparzolam 1/2 por noche, insulina NPH 20 unidades, glucopaghe 1 por dìa, atorvastatina
1	1	0	1	sertralina 25 mgrs, clonazepam 0,5 mgrs, ACO
1	0	1	0	alprazolam 1/2 por noche y Fluoxetina 20 mgrs/dìa, ranitidina 300 mgrs/dìa
1	1	1	1	prednisona, colchicina, plaquinol,tramadol
1	0	1	0	glucopage, enalapril, paracetamol, hidroclorotiazida, omeprazol,
1	0	0	0	insulina, procralan, amplex, escitalopran, clonazepam

0	0	1	0	fluoxetina 20 mgrs/dia
1	0	0	0	mgrs/noche, ac. valproico 200mgrs/dìa, quetiapina 50 mgrs/dìa
0	0	1	0	nada por embarazo
1	1	0	0	no
1	0	0	0	enalapril, atenolol, aspirina, atorvastatina
0	0	1	0	sertralina,ranitidina , carbamazepina
1	0	0	0	si
0	0	0	0	ibuprofeno
0	0	0	0	sertralina y clonazepan

1	1	0	0	alprazolam,fluoxetina
0	1	1	0	serttralina, clormezanona (SOS)
0	1	0	0	flouxetina
0	0	0	0	losartam sertralina 50 clona
1	0	0	0	sertralina amitriptilina calcio celebra
0	1	1	0	venlafaxina 75 enalapril hidrorronol t
0	0	0	0	fluoxetina 20
0	0	0	0	neuryl 2 mg
1	1	1	0	venlafaxina75

1	0	0	0	fluoxetina
1	1	0	0	glibenclamida me losartan hidroclorotiazida atenolol
0	0	0	0	clonazepam
1	0	0	0	sertralina 50 alprazolam 0,5 risp
0	0	0	0	sertralina
1	1	1	0	fluoxetina amitriptilina
1	0	0	0	enalpril carbidol aspirina espirolnolactona
0	0	0	0	antihipertensivo
0	0	0	1	sertralina y alparzolanm

1	0	0	0	vitaminas
1	0	0	0	fluoxetina 40 clona 2
0	0	0	0	sertralina
1	0	1	0	sertrtalina
0	0	0	0	imipramina clonazepam
0	0	0	0	NO
1	0	1	1	noche por insomnio hace años, carbamazepina por neuralgia al trigemino 500 mg
1	0	1	0	
1	0	1	0	hace 1 mes le suspendieron eutirox-

1	0	1	0	
1	1	1	0	
0	0	0	0	aco inyectable
1	0	1	0	paracetamol, prednisona, celebra, calcio, vit b12, inhalador ocasional
1	1	1	1	zopiclona compra ilegal, levotiroxina 100 dia, migranol 4 - 5 diarios
0	1	1	1	no
0	0	1	0	no
0	0	1	0	ciclobenzaprina, por su cuenta, para dormir mejor
0	0	0	0	ninguno

0	0	0	0	alprazolam 0,50, 1/2 noche
0	0	1	0	paracetamol
1	0	1	0	ninguno
0	0	1	0	no
1	0	0	0	no
0	0	0	1	no
1	0	1	0	solo medicamentos para Hipertensión: furosemida, nifedipino, losartan
0	1	1	0	SERTRALINA Y CLONAZEPAM, TENSOVIL
1	1	0	0	eutirox

1	0	1	0	antidepresivo
0	0	0	0	
0	0	1	0	natidepresivo suspendido por intolerancia
0	0	0	0	anticonceptivos
1	1	1	0	paarcetamol, tramadol. omeprazol, cerebra, hipertensión
1	1	0	0	angelic, ciclobenzapina
1	0	1	0	hormonas
1	0	0	0	ENLAPRIL, HIDROCLOROTIAXIDA, ASPIRINA
0	0	0	1	fluoxetina 20 mgrs/dia, eutirox 50 ugrs/dìa, clonazepam 1 mgrs/dìa

1	1	1	1	alprazolam 0,5 mgrs, fluoxetina 20 mgrs/dìa
1	0	0	0	Fluoxetina 20 mgrs 1/2 por dìa, Amitriptilina 25 mgrs 1/2 por dìa
0	0	0	0	Fluoxetina 20 mgrs/ dìa, Clonazepam 2mgrs 1/4 noche
0	0	0	0	sertralina 50 mgrs/dia
1	1	1	0	sertralina 50 mgrs/dia, clonazepam 2 mgrs 1/2 por noche
0	0	0	0	sertralina 100 mgrs/dìa, anulet
0	1	1	0	sertralina 150 mgrs/dìa, alprazolam 0,5 mgrs 1/2 por noche
0	0	0	0	sertralina 50 mgrs/dia, alprazolam 0,5 mgrs
0	0	1	0	fluoxetina 20 mgrs/dìa, alprazolam 0,5 mgrs/dìa, propanolol 5 mgrs

0	0	1	1	levotiroxina 25 mc
0	0	0	0	no toma
1	1	1	1	enalapril 20 mg/d, atorvastatina 20 mg/n
1	1	1	1	Metoclorpropamida , Omeprazol, Paracetamol
0	0	0	0	Losartán 2/d, paraceramol 500 mg, celebra
0	0	0	1	no toma medicamentos
0	0	0	0	ninguno
1	1	0	0	ninguno
1	1	1	0	eutirox 100 mcgr/d

1	0	1	0	risperidona, clonazepam, diclofenaco
0	0	0	0	no recuerda
1	1	0	1	losartan, aspirina
0	0	1	0	metformina,
0	0	1	1	OMEPRAZOL, RANITIDINA
0	0	0	1	METFORMINA,LOZARTAN
0	0	0	0	CLONAZEPAM
0	0	0	0	
0	1	1	0	ANTIDEPRESIVOS

0	1	1	0	SULFATO FERROSO
1	0	0	0	
0	1	1	0	EUTIROX
1	0	0	0	ANTIDEPRESIVOS
0	0	1	0	LOZARTAN, HIDROCLORDIAT, SERTRALINA
0	0	0	0	
0	0	0	0	ATROVASTATINA
0	1	1	0	PROPANOLOL
0	0	1	0	ANTIDEPRESIVOS

1	0	1	0	ENALAPRIL, EUTIROX,
0	0	0	0	no
1	0	1	1	si
0	0	1	0	si
1	0	0	0	aspirina,
0	0	0	0	eutirox, alprazolam, sertralina
1	0	0	0	FLUOXETINA, ALPRAZOLAM
1	0	1	0	LOSATARTAN? (PARA LA PRESION)ASPIRINA
0	0	0	0	antidepresivos no recuerda el nombre (no esta la ficha)

1	0	0	0	ALPRAZOLAM++++++++++++++
0	0	0	0	FLUOXETINA, ALPRAZOLAM
0	0	0	0	si
0	0	0	0	metformina
1	0	0	0	venlafaxina
1	0	1	0	alprazolam amitriptilina glibenclamida
1	1	1	0	eutirox 75
1	0	0	0	carabamacepina sertralina
0	0	0	0	fluoteina 20

0	0	0	0	
0	0	0	0	Sertralina50 alprazolam glucosamina metformina
0	0	0	0	
0	0	0	0	Anota us sertralina clona
0	0	0	0	No conoce
1	0	0	0	Fluoxetina20amitriptilona
0	0	0	0	Sertralina
1	0	1	0	Metoprolo
0	0	0	0	Clonazepam sertralina

1	0	1	0	C
1	0	0	0	Giben lamida y metro riman aspirina
1	0	0	0	No sabe
0	0	0	0	
0	0	1	1	no
1	1	0	0	
0	0	0	0	no
0	0	0	0	dusulfiram y alprazolam
1	0	1	0	losartan, hidroclorotiazida y omeprazol

0	0	1	0	aco inyectable
0	1	1	0	
0	0	0	0	aco
0	1	1	0	hodroclorotiazida, aspirina, aco
1	0	0	0	acido valproico por neurologo epilepsia
1	0	0	0	no
0	0	0	0	metformina, insulina, enalapril
0	0	1	0	melatonina para insomnio automedicado
1	0	1	0	levotiroxina

0	0	0	0	migranol
1	1	1	0	no
1	0	0	0	atorvastatina
1	1	1	0	no
0	0	0	0	no
0	1	0	0	no
1	0	0	1	propanolol, metformina, clonazepam, sertralina, omeprazol
1	0	0	0	sertralina, clonazepam y propanolol
1	0	0	0	fluoxetina 20 mgrs1/2 por dìa, alprazolam 0,5 mgrs

0	0	0	0	gemfibrozilo 300 mgrs, Fluoxetinas 20 mgrs/dìa
0	0	0	0	paroxetina40 mgrs, topiramato 50 mgrs, clonazepam 2 mgrs
0	1	1	0	paracetamol 6 comprimidos/dìa, clonazepam 0,5 mgrs, sertralina 50 mgrs
0	0	1	0	crestor 20 mgrs, amaryl 2/dia, aprovasc 1/dia, januvia 1/dia
0	1	0	1	quetiapina 25 mgrs, modafinilo 100 mgrs,vartalan 320, disgrin, venlafaxina 75 mgrs
1	1	1	0	1/4, sertralina 50 mgrs/dìa, levetiracetam 1 grs 1/2 cada 12 hrs, valcote er 500 mgrs
0	0	0	0	fluoxetina 2/dia, alprazolam 0,5 mgrs/noche
1	0	1	1	Eutirox 50 mcgr/día
1	1	0	0	Nada

0	0	0	0	Nada
1	1	0	0	Nada
1	0	1	1	Nada
1	0	1	0	Celebra
0	0	1	0	enalapril, atorvastatina
1	0	1	1	Eutirox 75 mg/d, atorvastatina 5 mg/noche
0	0	0	0	Neosintron, atorvastatina, losartan,
1	0	0	0	calcio
1	1	0	0	alprazolam, enalapril, paracetamol, no recuerda

0	0	0	0	NO RECUERDA
0	0	0	0	
0	0	0	0	NINGUNO
0	0	1	0	ENALAPRIL, ALPRAZOLAM
1	0	1	0	OMEPRAZOL, ENALAPRIL, PARECETAMOL, CACIO
0	0	1	0	CLONAZEPAM,
0	0	0	0	no consume
1	0	0	0	CLONAZEPAM
1	0	1	0	ASPIRINZA, ATORVASTATINA, ENALAPRIL

1	0	1	1	ATENELOL. LOZARTAN, ASPIRINA, FURASEMIDA, OMEPRAZOL
1	0	1	0	CICLO
1	1	1	0	EUTIROX, OMEPRAZOL, PARACETAMOL, DICLOFENACO
1	0	0	0	OMEPRAZOL, TRAMADOL, CELEBRA, FINEDIPINO, ASPIRINA,
0	1	1	0	KETOPROFENO, PARACETAMOL
1	0	0	0	ASTORBASTATINA, OMEPRAZOL, ENALAPRIL
1	1	1	0	ANTIDEPRESIVO
0	0	0	0	DEPRESION, DIABETES, HIPERTENSION
0	0	1	0	ASORBATATINA, PROPANOLOL, LOZARTAN

1	0	1	1	OMEPRAZOL, AEROGASTROL
1	0	1	0	mgrs/ dia, Clonazepam 2 mgrs 1/2 cada 12 hrs,gemfibrozilo, atenolol,hidroclorot
1	1	1	0	escitalopram 10 mgrs 1/2 por día, clonazepam 1mgrs/noche
1	0	1	0	venlafaxina 75 mgrs/dia, Clonazepam 1 mgrs/noche
0	0	0	0	fluoxetina 20 mgrs/dia, acido valproico 200 mgrs/dia, cardioplus
1	0	1	1	mgrs/dia, Clonazepam 1 mgrs/SOS, atorvastatina, allopurinol
0	0	1	1	sertralina 200 mgrs/dia, clonazepam 2 mgrs/noche
1	1	1	0	mgrs/día, eutirox 50 ugrs/día, prednisona 10 mgrs/día, omeprazol 20
1	0	0	1	clonazepam 1 mgrs/noche, fluoxetina 20 mgrs/dia

0	0	0	1	sin fármacos
0	0	1	0	nalerona, paroxetina, lamotrigina, quetiapina, lorazepam
1	0	1	1	acido folico por sospecha de embarazo
1	1	1	0	clonazepam,
0	1	0	0	eutirox 100 mg dia
1	0	0	0	furosemida, losartan, atorvastatina, levotiroxina
0	0	0	0	enalapril, aspirina, paracetamol
0	0	0	0	
0	0	0	0	antidepresivo indicado particular y clonazepam

1	1	1	1	no
0	0	0	0	amitriptilina y clonazepam
0	0	0	0	hta, artrosis, diabetes
0	0	0	0	tto hongos pies
0	1	0	0	metformina, losartan. nifedipino , aspirina, atorvastatina
1	0	0	0	levotiroxina 50 la mitad en ayunas
0	0	0	0	
1	0	1	0	nifedipino, omeprazol, glibenclamida, metformina,y otros del hospital
1	1	1	0	tramadol ocasional, ketoprofeno

0	1	0	0	enalapril
1	0	1	0	sertralina y amitriptilina indicadas hace un mes
0	1	0	0	metformina, levotiroxina, hidroclorotiazida, salbutamol y otro puff
1	1	0	0	eutirox 75 dia
0	0	0	0	valapres, cardioaspirina
0	0	1	1	vitaminas
1	1	1	1	no
1	1	1	0	no
1	0	0	0	enalapril, atorvastatina, hidroclorotiazida

0	0	0	0	no
0	1	0	0	Sertralina 50 amitrip. 12,5losartan o,5 hidroclorotiazida metformina
1	0	0	1	glnenclamida losratan
0	0	0	0	no sabe
1	0	0	0	0
1	0	0	0	enalapril lovastatin aspirina
0	0	1	0	metoclopropamida
0	0	0	0	
0	0	1	0	no

1	0	1	1	muchos ver en la ficha metrotexato ac folico tramadol atenolol alprazolam
0	0	0	0	atorvastaina nifedipino
0	1	1	0	metformina
0	0	0	0	
1	0	1	0	
1	0	0	0	
0	0	0	0	
1	0	0	0	
0	0	0	0	

0	0	1	0	
0	0	1	0	
1	0	0	0	no recuerda
0	0	0	0	Eutirox 50 mc
0	1	0	0	Nada
0	0	0	0	Eutirox
0	0	0	0	Metformina, Eutirox, enlapril
0	0	1	1	enalapril
0	0	0	0	ibuproeno por dolor en zona lumbar

0	0	0	1	atenolol, losartán, cardicon, tramadol
1	0	0	1	Nada
1	0	0	0	celebra, metrotrexato, acido fólico, calcio, paracetamol
0	0	0	1	Nada
1	0	1	0	ANTICOAGULANTE, ENALAPRIL,
0	0	0	0	PARACETAMOL, OMEPRAZOL, TRAMADOL
0	0	0	0	EUTIROX
0	0	0	0	ANTIDEPRESIVOS Y BDZ
1	1	1	0	GEMFIBROZILO

0	0	1	0	ARILEX FT
0	0	0	0	NIFEDIPINO, LOSARTAN, HIDROCLORDITIZIDA, INSULINA
0	1	0	0	ANTIDEPRESIVOS, DIPIRONA
0	0	0	0	AMITRIPTILINA, LEVO, PARACETAMOL Y TRAMADOL
1	0	1	0	LOSARTAN,HIDROCLORITIAXIDA, METFORMINA, ANLODOPINO, ASPIRINA
0	0	0	0	ANTIDEPRESIVOS
1	0	1	0	citalopram 10 mgrs/día
1	0	1	0	prednisona, celebra, metrotexate inyectable, paracetamol
1	0	0	1	fluoxetina 20 mgrs/día, Clonazepam 2 mgrs/noche

1	0	0	0	clonazepam, sertralina
1	0	0	0	sertralina 100 mgrs/día, clonazepam 2 mgrs/noche
0	1	0	1	sertralina 150 mgrs
1	0	1	0	eutirox, aspirina, furosemida, metformina, losartan, angiodisten
1	0	0	0	metformina, glibenclamida, atorvastatina, hisdroclorotiazida, omeprazol
1	0	0	0	Nada
1	0	0	0	enalapril, hidroclotiazida, nitrofurantoina, atorvastatina
0	0	1	0	Eutirox 75 microg
1	0	1	0	Eutirox, Enalapril, Losartán, paracetamol, ácido fólico

1	1	1	0	Nada
1	0	0	1	Nada
1	0	1	0	Nada
0	0	1	0	Eutirox 100 mc/d, enalapril, aspirina, hidroclorotiazida
1	0	0	1	Prednisona, y otros que no recuerda
0	0	0	0	ninguno
0	1	1	0	nifedipino, losartan y aspirina
1	1	1	0	alprazolam noche, desde último intento suicidio, desde febrero,
0	0	0	1	paracetamol,

1	1	0	0	clonazepam 0,5 mg noche SOS por su cuenta
0	0	0	0	
1	1	0	0	sertralina
1	0	0	0	paracetamos maganesio aspirina ebalapril
1	1	0	0	atenolol
1	1	1	1	regimen hisposodico
0	0	0	0	
0	0	1	0	alprazolam, propanolol
0	0	0	0	sertralina

0	0	1	1	acidoalendronico (fijafor de calcio) , enalaprim, elcal d,piascledine
0	0	0	0	enalapril, para la diabetes, atenolol
1	0	1	0	enalaprin, aspirina, clonazepan
0	1	1	1	
0	0	0	1	acido folico.....
0	0	0	0	propanolol
0	0	1	1	eutirox, metformina
0	0	0	0	
1	1	1	0	

1	0	0	0	enalapril, atorvastatina
0	1	0	0	
0	0	0	0	doloneurobionta, paracetamol, para la presion
0	0	1	0	insulina, atorvastatina, paracetamol, enalapril
0	0	0	0	levotiroxina, omeprazol
0	0	1	0	eutirox
1	0	1	0	
0	0	0	0	anticonceptivo
0	0	1	0	ANTIDEPRESIVOS

1	1	1	0	CLONAZEPAN
0	0	0	0	CLONAZEPAN, SERTRALINA
0	0	0	0	LOSARTAN
0	0	0	0	METFORMINA, ASTORBASTATINA, PARACETAMOL
0	0	0	0	ANTIDEPRESIVO,SALBUTAMOL
1	1	1	0	OMEPRAZOL Y ANTIDEPRESIVOS
1	1	1	0	LOSARTAN
0	0	0	0	ANTIDEPRESVOS, GRIFODROXILO
0	1	1	0	ANTIDEPREIVOS, PROPANOLOL

1	0	1	0	Losartan, enalapril, tramadol,
0	0	0	0	Nada
0	0	1	0	Nada
1	0	1	0	Nada
0	0	0	0	Omeprazol
0	0	1	1	Araba, metrotexato, celebra, calcio, omeprazol
1	0	0	0	Nada
1	0	0	0	losartán 2/día Atorvastatina
0	0	1	1	Methformina, losartán, paracetamol, tramadol, celebra

1	0	1	0	Calcio
0	0	0	0	DOMPERIDONA, FAMOTIDINA
0	0	0	0	ANTIDEPRESIVOS
1	0	1	0	Paracetamol 500 mg/d
0	0	0	0	Nada
0	0	0	0	no sabe
1	1	1	1	sertralina
1	1	0	1	sertralina 100
1	1	0	0	ENALAPRIL, HIDROCLOROTIAZIDA, SULFATO FERROSO, ÁCIDO FÓICO

0	0	0	0
1	0	1	0 Muchos

ALERGIAAFÁRMACOS	ANTIDEPRESIVOSINDICADOS	SETOMALOSMEDICAMENTOSINDICADOSSEGÚNLOQUEELMÉDICOLEI	TIPODETERAPIASUGERIDA	@1.¿Conquéfrecuenciaconsumesbebidasalcohólicas
0	Sertralina	0	0	1
0	Sertralina	1	0	0
0	Sertralina, clonazepam	1	2	2
0	Sertralina, amitriptilina 25	1	0	0
0	Fluoxetina, clonazeapm 1 noche	1	2	0
0	Sertralina	1	0	1
0	Fluoxetina, clorfenamiona	1	0	1
0	Fluoxetina, amitriptilina 25	1	2	1

0	tratamiento ad	1	2	0
0	Fluoxetina	1	2	2
0	Fluoxetina, amitriptilina	1	2	1
0	Fluoxetina, alprazolam, propranolol	1	2	0
0	Fluoxetina	1	2	0
0	Fluoxetina	1	2	0
0	lorazepam	1	2	0
1	alprazolam 0,5	1	2	1
0	Sertralina, propanolol clona	0	2	1

0	Fluoxetina, clona zepam	1	2	1
0	Sertralina	1	2	0
0	Fluoxetina	1	0	0
0	Fluoxetina, clona y clopromazina	1	0	2
0	amitriptilina ver ficha	0	2	1
0	Sertralina, alprazolam	1	2	1
0	Fluoxetina	1	2	1
0	Fluoxetina, alprazolam	0	0	1
0	Fluoxetina, alprazolam 0,25	1	0	1

0	Sertralina, clonazepam	1	6	0
0	Fluoxetina	1	2	0
0	Fluoxetina	1	0	0
0	Venlafaxina, Benzodiazepina, clonazepam 2 venlafaxina75	1	2	0
0	Sertralina, Benzodiazepina, sertralina 50 mg dia, diazepam 5 mg noche	1	2	0
0	Sertralina, 50 mg por dia	1	2	1
0	Sertralina, Benzodiazepina, sertralina 50 mg por dia clonazepan 2mg por dia	1	2	1
0	Sertralina, Benzodiazepina, sertr 50 mg dia clnz 1mg noche	1	2	0
1	Fluoxetina, fluoxetina 30 mg mañana haloperidol 1 mg noche	1	2	1

1	Fluoxetina, 20 mg mañana	1	2	0
0	Benzodiazepina, sertralina 50 mg mañana clonazepam 2 mg noche	1	2	0
0	Fluoxetina, amitriptilina 25 mg noche	0	2	1
0	Sertralina, sertralina 50 mg am , amitriptilina 25mg, media por noche	1	2	1
0	Benzodiazepina, alprazolam 0.5 noche y sertralina 50 mg mañana	1	2	1
0	Fluoxetina, clordiazepóxido	1	2	2
0	Benzodiazepina, fluoxetina 2o mg 1 en la mañana /// clonazepam 2 mg 1/2 dos veces al dia	1	2	0
0	Fluoxetina, fluoxetina 20 mg, 1/2 am	1	2	1
0	Benzodiazepina, alprazolam (0,50) 1/2 noche y Sertralina (50 mg) 1 AM	1	2	0

1	Fluoxetina, Benzodiazepina, Fluoxet 1 1/2 al dia, y Alpraz 1 noche	1	2	0
0	Sertralina, sertralina 50 mg 1 dia, agregó Stressam 1 c/12,	1	2	1
0	Venlafaxina	1	2	1
0	Fluoxetina, Benzodiazepina, fluoxetina 1 1/2 al dia y alprazolam 0.50 1 en la noche	1	2	0
0	ninguno	0	2	0
0	Fluoxetina, fluoxetina: 1 1/2 al dia; clorpromazina 25: 1 cp noche	1	2	0
0	Amitriptilina 25 mg/día	1	4	0
0	Venlafaxina	1	2	0
0	Fluoxetina, no toleeró la fluoxetina	1	4	0

0	Sertralina	1	4	0
0	Sertralina, 100 MG/D	1	1	0
0	Sertralina, 50 MG/D	1	2	0
0	Sertralina, 100 MG/D	0	2	0
0	Sertralina, AMIPTRIPTILINA 25 MG 1 AL DÍA	1	2	0
0	Fluoxetina	1	2	0
0	Citalopram	0	2	1
1	Sertralina	0	2	0
0	Sertralina	1	2	2

0	Citalopram, 20 MG.	1	2	0
0	Fluoxetina, Benzodiazepina	1	0	1
0		1	2	0
0	Citalopram, PREGABALINA	1	2	1
0	Sertralina, Benzodiazepina	1	2	0
0	Fluoxetina, Benzodiazepina, alprazolam,	1	4	0
0	Venlafaxina	1	2	0
0		1	2	0
0	Sertralina, Benzodiazepina, 100 mg de Sertralina y =,5 mg Alprazolam	1	0	1

0	Benzodiazepina, Alprazolam 0,25 mg/noche, Clonazepam 2 mg/noche	1	4	0
0	Sertralina, 50 mg de sertralina y 1 mg/noche de Clonazepam	1	4	0
0	Sertralina, 50 mg y eszopiclona 3 mg	0	7	0
0	Sertralina, 50 MG DE sERTRALINA Y 0,5 MG/ CLONAZEPAM	1	6	0
1	Citalopram, Benzodiazepina, 20 MG/D	1	4	0
0	Benzodiazepina, 100 SERTRALINA, 1 MG CLONAZEPAM, ACIDO VALPROICO 400	1	0	0
0	Fluoxetina, Benzodiazepina, 20 mg y alprazolam 0,5 mg/noche	0	2	1
0	Fluoxetina, Benzodiazepina, 20 mg de Fluoxetina y 2 mg/noche de Clonazepam	1	2	0
0	Fluoxetina, Benzodiazepina, 20 fluoxetina y 2 mg Clonazepam	1	2	0

0	Sertralina	1	6	0
0	NO RECUERDA	0	6	0
0	Fluoxetina, 1 AL DÍA	0	2	1
0		0	2	0
0	Sertralina, 50 MG	1	4	0
0	Sertralina, 150 MG	1	2	0
0		0	6	2
1	Sertralina	1	7	0
0	Sertralina	1	4	0

0	Sertralina	1	4	1
0	Sertralina, Benzodiazepina	0	0	0
0	Sertralina, SRT 100 mgr, AMT 25 mgrs	1	2	1
0	Fluoxetina, Benzodiazepina	0	1	1
0	Fluoxetina, Sertralina, Benzodiazepina, SRT 100 mgrs	1	2	1
0	Benzodiazepina, Fluoxetina 20 mgrs/dìa, Alprazolam 1/2 por noche	1	2	1
1	Sertralina, Benzodiazepina	1	2	1
0	Fluoxetina, Benzodiazepina, Fluoxetina 20 mgrs, Alprazolam 0,5 mgrs	1	2	1
1	escitalopram 10 mgrs	1	2	0

0	Fluoxetina, Benzodiazepina	0	2	0
0	Benzodiazepina, acido valproico, quetiapina	0	2	0
0		0	0	0
0	Fluoxetina	1	2	0
0	Fluoxetina	1	2	0
0	Sertralina, carbamazepina	0	2	0
0	Sertralina	1	2	0
0	fluoxetina y alprazolam	1	2	0
0	Sertralina, clonazepan	1	4	2

0	Fluoxetina, alpz	0	2	0
0	Sertralina	1	2	2
0	Fluoxetina	1	6	0
0	Sertralina, Benzodiazepina	1	6	2
0	Sertralina	1	6	1
0	Venlafaxina	0	2	0
0	Fluoxetina	1	3	0
0	Sertralina, clona	0	2	0
0	Venlafaxina, Benzodiazepina	0	2	4

0	Fluoxetina	1	2	0
0	Sertralina, Benzodiazepina	1	0	0
0	Fluoxetina	1	0	0
0	Sertralina	1	2	1
0	Sertralina	1	2	1
0	Fluoxetina	1	6	1
0	Sertralina, Benzodiazepina	0	2	0
0		1	2	1
0	Sertralina, Benzodiazepina	1	6	0

0		0	6	1
0	Fluoxetina, Benzodiazepina, clona 2	1	2	1
0	Sertralina, Benzodiazepina	0	6	0
0	Sertralina	1	6	1
1		1	0	1
0	CLONAZEPAM 2MG NOCHE FLUOXETINA 40 MG MAÑANA Y 40 MG A LAS 16 HRS	1	2	0
0	Sertralina, sertralina 50 mg noche amitriptilina 25 mg noche	1	2	0
0	Benzodiazepina, clonazepam en gotas y ectiban 10mg mañana	1	2	0
0	Benzodiazepina, sertralina 50 mg mañana clonazepam 1mg noche	1	2	1

0	Sertralina, Benzodiazepina, sertralina 50 mg dia clonazepam 1mg noche	1	2	1
0	Sertralina, Benzodiazepina, sertralina 50 mg dia y clnz 1 mg noche	1	2	2
0		0	2	0
0	Fluoxetina, Benzodiazepina, fluoxetina 20 mg dia, clonazepan 2 mg noche	1	2	0
0	Benzodiazepina, clonazepam 4 mg noche, sertralina 100mg noche	1	2	0
0	Benzodiazepina, imipramina 1 cp mañana y media en la once, alprazolam 0,5 mg noche	1	0	1
0	Sertralina, sertralina 50 mg: 1 am	1	0	2
0	Benzodiazepina, sertralina 50mg: 2 al dia y alprazolam 0,50: 1/2 por noche	1	2	0
0	Sertralina, Benzodiazepina, sertralina 50 mg: 2 al dia; alprazolam 0,50: 2 al dia	1	4	0

0	Benzodiazepina, fluoxetina 20 mg: 1 al dia; alprazolam 0,50: 1/2 por noche y reducir	1	2	0
0	Fluoxetina, fluoxetina de 20 mg: 1 AM/ clorpromazina de 25 mg: 1 noche	1	2	0
0	Fluoxetina, Benzodiazepina	1	2	0
0	Sertralina, Benzodiazepina, sertralina 50 mg: 1 am/ clonazepam 2 mg: 1/4 solo SOS	1	2	0
0	Fluoxetina, fluoxetina 20 mg: 1 am/ clorpromazina 25 mg: 1 noche	1	2	0
0	Fluoxetina, fluoxetina 20 mg: 2 al dia	1	2	0
0	Fluoxetina, fluoxetina 20 mg: 1 al dia	1	2	0
1	Sertralina, CLONAZEPAM	1	2	1
1	Citalopram, reljante muscular	1	2	0

0	Fluoxetina, Benzodiazepina	1	2	0
0		1	2	0
0	Sertralina, Benzodiazepina	1	2	0
0	Fluoxetina	0	2	2
0	Sertralina, Benzodiazepina	1	2	0
0	Sertralina, Benzodiazepina	1	2	0
1	Sertralina, Benzodiazepina	1	2	0
0	Benzodiazepina	1	2	0
0	Fluoxetina, Benzodiazepina, FXT 20 mgrs, CNZ 1 mgrs	1	2	0

1	Fluoxetina, Benzodiazepina	0	2	0
0	Fluoxetina, amitriptilina	1	2	0
0	Fluoxetina, Benzodiazepina	1	2	1
0	Sertralina	1	2	3
0	Sertralina, Benzodiazepina	1	2	1
0	Sertralina	1	2	1
0	Sertralina, Benzodiazepina	1	2	0
1	Sertralina, Benzodiazepina	0	2	0
0	Fluoxetina, Benzodiazepina	0	0	1

0	Fluoxetina, Benzodiazepina, 20 mg/d y Alprazolam 0,5 mg/noche	1	2	0
0	Fluoxetina, 20 mg/d	0	2	1
0	Sertralina, Benzodiazepina, 75 mg/d y Clonazepam 2 mg/n	0	2	0
0	Fluoxetina, Benzodiazepina, Fluoxetina 20 mg/d y Alprazolam 0,5 ng/noche	1	2	0
0	Fluoxetina, Benzodiazepina, 40 mg/d, Clonazepam 2 mg/d	1	0	0
0	no toma	0	2	2
0	Sertralina, Benzodiazepina, 100 mg/d y 2 mg/clonazepam	0	2	0
0	Sertralina, 100 mg/d	1	2	0
0	escitalopram 10 mg/d, Clonacepam 1 mg/noche	0	2	0

0	Sertralina	0	4	2
0	Sertralina	0	6	1
0	Sertralina	0	2	0
0	amiptriptilina, sertralina	1	2	1
0	Fluoxetina, Sertralina	1	6	0
0	Fluoxetina	0	6	0
0	Sertralina	1	6	0
0	Sertralina	0	2	0
0		1	2	0

0		1	2	0
0	Fluoxetina, Benzodiazepina	1	2	0
0	Fluoxetina	0	2	1
0	Sertralina, Benzodiazepina	1	2	1
0	Sertralina	1	2	1
0	Fluoxetina	0	2	0
0	Sertralina	1	2	0
0	Fluoxetina, Benzodiazepina	1	2	0
0	Fluoxetina, Benzodiazepina	0	2	1

0	Benzodiazepina	1	2	0
0	ninguno	0	2	0
0	Sertralina	1	2	2
1	Fluoxetina	1	2	0
0	Fluoxetina, ALPRAZOLAM	1	2	0
0	Sertralina, alprazolam	1	2	0
0	Fluoxetina	1	2	0
0	Sertralina, CLONAZEPAN	1	2	0
0	Citalopram, , clonazepan	1	2	0

0	Fluoxetina	0	2	0
0	Fluoxetina	1	2	0
1	Sertralina	1	2	0
0	Fluoxetina, para dormir, no recuerda el nombre	1	0	2
0	Venlafaxina, Benzodiazepina	1	2	2
0	Benzodiazepina	0	0	1
0	Sertralina, Benzodiazepina, amitriptilina 12,5	1	6	0
0	Sertralina	1	2	1
0	Fluoxetina, amitriptilina	1	6	1

0	Sertralina, carbamacepina	1	6	1
0	Sertralina	1	3	0
0		0	0	0
0	Sertralina, Ría perdona clona	0	2	4
0	Sertralina, Risperidona carbamacepina	0	2	1
0	Fluoxetina, Amitriptilina	1	2	0
0	Sertralina, Benzodiazepina	1	6	1
0	Sertralina	1	2	2
1	Sertralina, Benzodiazepina	1	0	1

1	Sertralina, Benzodiazepina	1	2	0
0	Sertralina, Benzodiazepina	1	3	1
0		1	2	0
0	Sertralina, Benzodiazepina	1	2	1
0	Benzodiazepina, sertralina 50 mg am y 25 mg tarde, alprazolam 0,5 mg noche	1	6	1
0	imipramina 25 mg 1 cp mañana y media en la tarde, clorfenamina 4mg noche	1	6	0
0	Sertralina, Benzodiazepina, clonazepam 2 mg 1 noche sertralina 1 am y 1 once	1	2	2
0	Benzodiazepina, sertralina 50 mg media en la mañana y media en la tarde, alprazolam	1	2	1
0	Benzodiazepina, diazepam 10 mg noche, fluoxetina 1 cp mañana y media almuerzo	1	0	0

0	Sertralina, 1 cp mañana y media tarde	0	6	2
0	Sertralina, Benzodiazepina, sertralina 100mg dia alprazolam 1 noche	1	0	3
0	Sertralina, 50 mg dia	1	2	0
0	Sertralina, Benzodiazepina, sertralina 50 mg dia, alprazolam 0,5 medio noche	1	6	1
0	Sertralina, 50 mg dia	1	2	0
0	Benzodiazepina, fluoxetina 20 mg: 2 al dia/ clorpromazina 25 mg: 1 noche/	1	2	0
0	Fluoxetina, Benzodiazepina, fluoxetina 20 mg: 2 dia y alprazolam 0,50: 1 noche	1	2	0
0	Fluoxetina, fluoxetina 20 mg 1 am/ amitriptilina 25 mg 1 noche	1	2	2
0	Fluoxetina, fluoxetina 1 /dia y amitriptilina 1/2 noche	1	2	0

0	Fluoxetina, fluoxetina 1 al dia y amitriptilina 1 en la noche	1	2	0
0	ninguno, considerando cuadro no tan severo y lactancia	0	2	0
1	Benzodiazepina, sertralina 50 mg 1 am y clonazepam 2mg 1/4 por la noche	1	2	4
0	Benzodiazepina, fluoxetina:2 al dia/ alprazolam 0,5: 1/2 por noche/ clorpromazina 25	1	2	0
1	Sertralina, sertralina 50 mg : 1/dia	1	4	0
0	Sertralina, sertralina 50 mg 1 al día	1	2	0
0	Sertralina, Benzodiazepina, sertralina 75 mgrs, clonazepam 1 mgrs	1	2	0
0	Benzodiazepina, sertralina 100 mgrs, clonazepam 1,5 mgrs, propanolol 30 mgrs	1	2	1
0	Sertralina, Benzodiazepina	0	2	0

0	Fluoxetina	1	2	2
0	Paroxetina, Benzodiazepina, topiramato	1	4	0
0	Sertralina, Benzodiazepina	0	3	1
0	los desconoce	1	0	1
0	Venlafaxina, quetiapina, modafinilo,	1	2	0
1	Sertralina, Benzodiazepina, srt 50 mgrs y CNZ 0,5 mgrs	1	4	0
0	Fluoxetina, Benzodiazepina	0	4	0
1	Sertralina, 100 mg/d, Alprazolam 0,25 mg/noche	1	2	0
0	Fluoxetina, 20 mg/día	1	2	0

0	Sertralina, tomó un mes	1	2	0
0	Sertralina, tomó un mes y lo dejó	0	2	0
0	Sertralina, no recuerda que le prescribieron, pero en ficha sale sertralina 100 mg	0	2	0
0	Fluoxetina, 20 mg/día	1	2	0
1	Sertralina, Benzodiazepina, 100 mg/d y 1/3 de 10 mg de Diazepam	0	2	0
1	Fluoxetina, Benzodiazepina, 40 mg fluoxetina y 1 mg de clonazepam/noche	1	2	0
0	Fluoxetina, 40 mg de fluoxetina, alprazolam 0,25 mg/noche	1	2	0
0	Fluoxetina, Benzodiazepina, 20 fm fluoxetina alprazolam, no recuerda bien	1	2	0
0	Sertralina	1	4	1

0 Sertralina	1	7	0
0 Sertralina	0	4	0
0	0	7	0
0 Sertralina	0	2	0
0	1	4	0
0 Fluoxetina	0	2	0
0	0	2	0
0 Sertralina	1	4	1
0	1	2	0

0	Citalopram, Benzodiazepina	1	2	0
0	Fluoxetina	1	2	0
1	Fluoxetina, Benzodiazepina, ALPRAZOLAM	1	2	0
0	Sertralina, Benzodiazepina	1	2	0
0	Venlafaxina, Benzodiazepina	0	2	0
0	Fluoxetina, Benzodiazepina	1	2	0
0	Sertralina, Benzodiazepina	1	2	0
0		1	2	0
0	Fluoxetina, Benzodiazepina	0	2	0

0	Fluoxetina, Benzodiazepina	1	2	0
0	Sertralina, Benzodiazepina	1	2	4
0	Benzodiazepina, escitalopram	0	0	1
0	Venlafaxina, Benzodiazepina	1	4	0
0	Fluoxetina, acido valproico	1	2	3
0	Sertralina, Benzodiazepina	1	2	2
1	Sertralina, Benzodiazepina	1	2	1
0	Sertralina, Benzodiazepina, amitriptilina	0	4	0
0	Fluoxetina, Benzodiazepina	1	2	1

0	ninguno	0	2	2
0	Paroxetina, Benzodiazepina, lamotrigina, quetiapina, nalerona	0	2	4
0	ninguno	0	2	1
0	Fluoxetina, Benzodiazepina, flx 2 al dia, alp 1 noche	1	2	0
0	Benzodiazepina, clnz 1 mg noche, sertralina 1 desayuno media once	1	2	1
0	Fluoxetina, Benzodiazepina, flx 40 mg dia clnz 1 mg noche	1	2	1
0	Fluoxetina, Benzodiazepina, flx 40 mg dia, dzp 10 mg noche	1	0	1
0	Fluoxetina, Benzodiazepina, flx 40 mg dia y bdz no recuerda	1	2	1
0	Benzodiazepina, sertralina 100 mg dia, clonazepam 1 mg noche y carbamazepina 200	1	6	0

1	Sertralina	1	6	1
0	Benzodiazepina	1	2	0
0	Fluoxetina, Benzodiazepina, fluoxetina 30 mg dia alprazolam 0,5 noche	1	0	1
1	Sertralina, Benzodiazepina, sertralina 50 mg dia, alprazolam 0.5 medio noche	1	0	0
0	Benzodiazepina, alp 1 po noche y otro	1	2	1
0		1	3	1
0	Sertralina, Benzodiazepina, sertralina 50mg dia, alp 0.5 noche	1	0	1
1	Sertralina, Benzodiazepina, alprazolam y sertralina	1	0	1
1	amitriptilina	1	0	1

0		1	2	1
0	Sertralina, sertralina 50 mg día y amitriptilina 25 mg media en la noche	1	2	1
0	Sertralina, Benzodiazepina, sertralina 50 mg dia, alprazolam 0.5 medio noche	1	2	0
0	Fluoxetina, 20mg dia	1	2	1
0	Sertralina, Benzodiazepina, clonazepam 1 mg noche, sertralina 50mg dia	1	0	1
0	Paroxetina, Benzodiazepina, alprazolam 1 al dia y paroxetina 20 my 1 al dia	1	2	0
0	no recuerda inicio hoy	0	2	1
1	Paroxetina	1	2	1
0	Sertralina, Benzodiazepina, sertralina 50 mg dia, alp o,5 noche	1	2	0

0	Sertralina, Benzodiazepina, sertralina 25 mg am, clonazepam 1 mg noche	1	2	1
0	Sertralina	1	2	0
0	Fluoxetina	1	6	0
0	no sabe	0	0	1
0	Sertralina, carbamacepina	1	6	1
0	Benzodiazepina	1	0	0
1	Fluoxetina	0	3	0
0	Sertralina	1	2	1
0	Sertralina	1	2	1

1	Sertralina	1	0	1
0	Fluoxetina, amitriptina	1	0	1
0	Benzodiazepina	0	2	1
0		0	0	0
0	Fluoxetina, Benzodiazepina	1	2	1
0	Sertralina	1	2	3
1	Sertralina	1	2	0
1	Sertralina, Benzodiazepina	1	6	0
0		1	2	0

1	Sertralina	1	2	1
1	Fluoxetina	1	0	0
0		1	3	0
0	Fluoxetina, Benzodiazepina, 40 mg fluoxetina y 0,5 mg/noche clonazepam	1	2	0
0	Escitalopram 5 mg/d/día	1	2	0
0	Sertralina, Benzodiazepina, Sertralina 50 mg/día y 0,5 mg/noche	0	2	0
0	Fluoxetina, 20 mg/fluoxetina	0	2	0
0	Sertralina, Benzodiazepina, 100 mg/d y 10 mg Diazepam	1	2	0
0	Benzodiazepina, 50 mg/d, Risperidona 1 mg/noche, Clonazepam 2 mg/noche	1	2	0

0	Fluoxetina, Benzodiazepina, Fluoxetina 40 mg/d y Clonazepam 0,5 mg/noche	0	2	0
0	Sertralina, Benzodiazepina, Alprazolam 0,25 mg y Sertralina 25 mg	1	2	0
0	Fluoxetina, quetiapina 25 mg/noche y 20 mg fluoxetina	1	2	0
0	Sertralina, Benzodiazepina, Clonazepam 1 mg/noche y 25 mg/sertralina	0	2	0
0		1	4	2
0	Fluoxetina	1	2	1
0	Sertralina	1	2	0
1	Venlafaxina, Benzodiazepina	1	2	2
0	Sertralina, Benzodiazepina	1	2	0

0	Sertralina, Benzodiazepina	0	2	4
0	Sertralina, Benzodiazepina	1	2	0
1	Fluoxetina, Benzodiazepina	1	2	0
0	Citalopram, Benzodiazepina	1	2	0
1	Bupropión, Citalopram, Benzodiazepina	1	2	0
0	Fluoxetina, Benzodiazepina	1	2	0
1	Citalopram	1	2	0
0	Benzodiazepina, Sertralina 200 mgrs/día, Clonazepam 1 mgrs/noche	1	2	0
0	Benzodiazepina, fluoxetina 20 mgrs/dia, Clonazepam 2 mgrs/noche	0	1	0

0	Sertralina, Benzodiazepina	0	2	4
0	Sertralina, Benzodiazepina	1	2	0
0	Sertralina, sertralina 150 mgrs/dia	1	2	0
0	Paroxetina, Benzodiazepina, quetiapina	1	2	0
1	Sertralina, Benzodiazepina, 100 mg/ d sertralina y 1 mg noche Clonazepam	1	0	0
0	Citalopram, Benzodiazepina, 10 mg de citalopram y 2 mg de clonazepam	0	2	0
0	Citalopram, Benzodiazepina, 20 mg de citalopram y clorodiozepóxido 5 mg/noche	0	2	0
0	Fluoxetina, Benzodiazepina, 20 mg de Fluoxetina y 2 mg Clonazepam	0	2	0
0	Sertralina, 100 mg/sertralina, 1 mg de Clonazepam	1	2	0

0	Sertralina, No recuerda	0	2	0
0	Sertralina, 100 mg Sertralina	0	2	0
0	Sertralina, Sertralina 100 mg/d	1	2	0
0	Fluoxetina, 20 mg/d	1	2	0
0	Fluoxetina, Benzodiazepina, 60 mg de fluoxetina, 4 mg/n clonazepam,	1	2	0
0	no los recuerda, solo sabe que son dos tipos y los está tmando	1	2	0
0	Sertralina, Sertralina 50 mg: 1 1/2 al dia	1	2	0
0	Sertralina, Benzodiazepina, sertralina 50 mg= 1 am / alprazolam 0,50 mg= 1 noche	1	2	0
0	Benzodiazepina, quetiapina 50 mg por la noche. Clonazepam 2 mg:	1	2	3

1	Fluoxetina, Benzodiazepina, fluoxetina: 2 al dia; clonazepam 2mg: 1/2 en la noche	1	2	1
0	Sertralina, Benzodiazepina	1	0	0
0	Sertralina, amitriptilina	1	2	1
0	Sertralina	1	0	4
0		1	0	0
0	Sertralina	1	6	1
0	Sertralina, alprazolam	1	2	0
1	Sertralina	1	2	0
0	Sertralina	0	2	0

0	Sertralina, alprazolam	1	0	0
0	Sertralina, amitriptilina, alprazolam	1	0	1
0	Venlafaxina, , alprazolam	1	2	0
0	Sertralina	1	0	0
0	Sertralina, alprazolam	0	0	0
0	Sertralina	1	2	1
0	Venlafaxina, clonazepam	1	0	0
0	Venlafaxina, clonazepam, risperidona	1	2	1
0	Sertralina, clonazepam	1	0	0

1	Sertralina	1	2	0
0	Fluoxetina	1	2	0
0	Sertralina, amitriptilina	1	2	0
0	Sertralina, amitriptilina	1	0	0
0	Fluoxetina, Sertralina, clorfenamina	0	2	0
0	Sertralina	0	2	0
0	Paroxetina, amitriptilina	1	2	0
0	Sertralina, alprazolam	1	0	2
0	Sertralina, Benzodiazepina	0	2	0

0	Sertralina, Benzodiazepina	1	2	0
0	Sertralina, Benzodiazepina	1	2	0
0	Benzodiazepina	1	0	1
0	Fluoxetina, Benzodiazepina	1	2	1
0	Fluoxetina, Benzodiazepina	1	2	1
0	Fluoxetina, Benzodiazepina	1	2	0
0	Benzodiazepina, AMITRIPTILINA	1	2	0
0	Fluoxetina, Benzodiazepina	1	2	0
0	Paroxetina, Benzodiazepina	1	2	1

0	Sertralina, Benzodiazepina, 100 mg/día Sertralina, 2 mg/n de clonazepam	1	2	0
0	Sertralina, Sertralina 50 mg/d	1	2	0
0	Sertralina, 100 mg/d y 2 mg/n clonazepam	0	0	0
1	Sertralina, Benzodiazepina, 200 mg/sertralina, 1 mg/noche clonazepam	1	2	0
0	nada	1	0	0
0	Sertralina, 100 mg/noche	1	2	0
0	Sertralina, Benzodiazepina, 100 mg/día y 0,5 mg/noche de alprazolam	0	2	0
0	Fluoxetina, Benzodiazepina, no recuerda el nombre del antidepresivo	1	2	4
0	Sertralina, Benzodiazepina, 200 mg/d, 5 mg/noche	1	2	0

0	Fluoxetina, 20 mg/noche	0	2	0
1	Sertralina, Benzodiazepina	0	2	0
0	Venlafaxina, Benzodiazepina	1	2	2
0	Sertralina, Benzodiazepina, Sertralina 50 mg/d y clonazepam 2 mg/noche	1	2	0
0	Benzodiazepina, fluoxetina 20 mg/d, Clonazepam 1 mg/noche, Ácido Valproico 400 mg/d	1	2	2
0	Sertralina	1	0	0
0	Sertralina	1	2	1
0	Sertralina, Benzodiazepina	1	6	1
0	Sertralina, AMIPTRIPTILINA	1	4	0

0	0	0	0
0 Sertralina	0	0	0

@2.¿Cuántasbebidasalcohólicasconsumesnormalmentecuandob	@3.¿Conquéfrecuenciatetomas6omásbebidasalcohólicas	@4.¿Conquéfrecuenciaenelcursodelúltimoañohassido	@5.¿Conquéfrecuenciaenelcursodelúltimoañonopudis	@6.¿Conquéfrecuenciaenelcursodelúltimoañohasnece
1	0	0	0	0
1	0	0	0	0
1	0	0	0	0
1	0	0	0	0
1	0	0	0	0
1	1	1	0	0
1	0	0	0	0
1	0	0	0	0

1	0	0	0	0
2	0	0	0	0
1	0	0	0	0
0	0	0	0	0
0	0	0	0	0
0	0	0	0	0
1	0	0	0	0
1	0	0	0	0
2	0	0	0	0

1	0	0	0	0
0	0	0	0	0
1	0	0	0	0
5	3	0	0	3
1	0	0	0	0
1	0	0	0	0
1	0	0	0	0
1	0	0	0	0
1	0	0	0	0

1	0	0	0	0
1	0	0	0	0
1	0	0	0	0
1	0	0	0	0
0	0	0	0	0
1	0	0	0	0
1	0	0	0	0
0	0	0	0	0
1	0	0	0	0

0	0	0	0	0
0	0	0	0	0
1	0	0	0	0
1	0	0	0	0
1	0	0	0	0
2	1	0	0	0
0	0	0	0	0
1	0	0	0	0
1	0	0	0	0

0	0	0	0	0
1	0	0	0	0
1	0	0	0	0
0	0	0	0	0
0	0	0	0	0
0	0	0	0	0
1	0	0	0	0
1	0	0	0	0
1	0	0	0	0

1	0	0	0	0
1	0	0	0	0
1	0	0	0	0
1	0	0	0	0
0	0	0	0	0
0	0	0	0	0
1	0	0	0	0
0	0	0	0	0
2	0	0	0	0

1	0	0	0	0
1	0	0	0	0
0	0	0	0	0
2	0	0	0	0
1	0	0	0	0
0	0	0	0	0
0	0	0	0	0
0	0	0	0	0
1	0	0	0	0

1	0	0	0	0
1	0	0	0	0
1	0	0	0	0
1	0	0	0	0
1	0	0	0	0
1	0	0	0	0
1	0	0	0	0
1	0	0	0	0
1	0	0	0	0

0	0	0	0	0
0	0	0	0	0
1	0	0	0	0
0	0	0	0	0
0	0	0	0	0
0	0	0	0	0
1	0	0	0	0
0	0	0	0	0
0	0	0	0	0

1	0	0	0	0
0	0	0	0	0
1	0	0	0	0
1	0	0	0	0
3	1	0	1	0
1	0	0	0	0
1	0	0	0	0
1	0	0	0	0
0	0	0	0	0

0	0	0	0	0
0	0	0	0	0
0	0	0	0	0
0	0	0	0	0
0	0	0	0	0
0	0	0	0	0
0	0	0	0	0
0	0	0	0	0
3	1	0	0	0

0	0	0	0	0
1	0	0	0	0
0	0	0	0	0
3	2	0	2	0
5	2	1	2	0
1	0	0	0	0
1	0	0	0	0
1	0	0	0	0
5	3	2	2	3

1	0	0	0	0
1	0	0	0	0
1	0	0	0	0
1	0	0	0	0
1	0	0	0	0
1	0	0	0	0
1	0	0	0	0
1	1	0	0	0
1	0	0	0	0

1	0	0	0	0
1	0	0	0	0
1	0	0	0	0
1	0	0	0	0
1	0	0	0	0
0	0	0	0	0
0	0	0	0	0
0	0	0	0	0
1	0	0	0	0

1	0	0	0	0
1	0	0	0	0
1	0	0	0	0
1	0	0	0	0
1	0	0	0	0
1	0	0	0	0
2	1	0	0	0
0	0	0	0	0
0	0	0	0	0

0	0	0	0	0
0	0	0	0	0
0	0	0	0	0
0	0	0	0	0
0	0	0	0	0
0	0	0	0	0
0	0	0	0	0
1	0	0	0	0
0	0	0	0	0

0	0	0	0	0
0	0	0	0	0
0	0	0	0	0
1	0	0	0	0
0	0	0	0	0
0	0	0	0	0
0	0	0	0	0
0	0	0	0	0
0	0	0	0	0

0	0	0	0	0
0	0	0	0	0
1	0	0	0	0
1	1	2	2	0
1	0	0	0	0
1	0	0	0	0
0	0	0	0	0
0	0	0	0	0
1	0	0	0	0

1	0	0	0	0
1	1	1	0	0
1	0	0	0	0
1	0	0	0	0
1	0	0	0	0
1	2	0	1	0
1	0	0	0	0
1	0	0	0	0
1	0	0	0	0

5	2	2	2	0
2	0	0	0	0
0	0	0	0	0
1	0	0	0	0
0	0	0	0	0
0	0	0	0	0
0	0	0	0	0
0	0	0	0	0
0	0	0	0	0

0	0	0	0	0
0	0	0	0	0
1	0	0	0	0
1	0	0	0	0
1	0	0	0	0
0	0	0	0	0
0	0	0	0	0
0	0	0	0	0
3	0	0	0	0

0	0	0	0	0
0	0	0	0	0
2	0	0	2	0
0	0	0	0	0
0	0	0	0	0
0	0	0	0	0
0	0	0	0	0
0	0	0	0	0
0	0	0	0	0

0	0	0	0	0
0	0	0	0	0
0	0	0	0	0
1	0	0	0	0
2	3	0	1	0
1	0	0	0	0
1	0	0	0	0
2	0	0	0	0
1	0	0	0	0

1	0	0	0	0
1	0	0	0	0
0	0	0	0	0
3	3	3	3	1
1	0	0	0	1
1	0	0	0	0
1	0	0	0	0
2	0	0	0	0
1	1	0	0	0

1	0	0	0	0
1	0	0	0	0
1	0	0	0	0
1	1	0	0	0
1	0	0	0	0
0	0	0	0	0
2	0	0	0	0
3	1	1	1	1
1	0	0	0	0

2	0	0	0	0
1	1	2	0	1
1	0	0	0	0
1	0	0	0	0
0	0	0	0	0
0	0	0	0	0
0	0	0	0	0
1	0	0	0	0
0	0	0	0	0

0	0	0	0	0
0	0	0	0	0
1	1	1	0	0
0	0	0	0	0
0	0	0	0	0
0	0	0	0	0
0	0	0	0	0
1	0	0	0	0
1	0	0	0	0

3	2	2	0	0
0	0	0	0	0
1	0	0	0	0
1	0	0	0	0
0	0	0	0	0
0	0	0	0	0
0	0	0	0	0
1	0	0	0	0
1	0	0	0	0

1	0	0	0	0
1	0	0	0	0
1	0	0	0	0
1	0	0	0	0
1	0	0	0	0
1	0	0	0	0
1	0	0	0	0
1	0	0	0	0
1	0	0	0	0

0	0	0	0	0
0	0	0	0	0
0	0	0	0	0
0	0	0	0	0
0	0	0	0	0
0	0	0	0	0
0	0	0	0	0
0	0	0	0	0
0	0	0	0	0

0	0	0	0	0
0	0	0	0	0
0	0	0	0	0
0	0	0	0	0
0	0	0	0	0
0	0	0	0	0
0	0	0	0	0
0	0	0	0	0
0	0	0	0	0

0	0	0	0	0
2	0	0	0	0
2	0	0	0	0
0	0	0	0	0
1	0	0	0	0
3	1	1	1	0
1	0	0	0	0
0	0	0	0	0
1	0	0	0	0

2	0	0	0	0
4	4	3	3	1
2	0	0	0	0
0	0	0	0	0
1	0	0	0	0
1	0	0	0	0
1	0	0	0	0
1	0	0	0	0
0	0	0	0	0

3	0	0	0	0
0	0	0	0	0
1	1	0	0	0
0	0	0	0	0
1	0	0	0	0
1	0	0	0	0
1	0	0	0	0
1	0	0	0	0
1	0	0	0	0

1	0	0	0	0
1	0	0	0	0
0	0	0	0	0
1	0	0	0	0
1	0	0	0	0
0	0	0	0	0
1	0	0	0	0
1	0	0	0	0
0	0	0	0	0

1	0	0	0	0
1	1	0	0	0
1	0	0	0	0
1	0	0	0	0
2	1	0	0	0
1	0	0	0	0
1	0	0	0	0
1	0	0	0	0
1	1	0	0	0

1	1	0	0	0
1	0	0	0	0
2	0	0	0	0
0	0	0	0	0
1	0	0	0	0
1	0	0	0	0
1	0	0	0	0
0	0	0	0	0
1	0	0	0	0

2	0	0	0	0
1	0	0	0	0
1	0	0	0	0
1	0	0	0	0
1	0	0	0	0
1	0	0	0	0
1	0	0	0	0
1	0	0	0	0
1	0	0	0	0

1	0	0	0	0
1	0	0	0	0
1	0	0	0	0
1	0	0	0	0
1	0	0	0	0
1	0	0	0	0
0	0	0	0	0
3	2	0	0	0
0	0	0	0	0

2	3	1	0	0
0	0	0	0	0
0	0	0	0	0
0	0	0	0	0
0	0	0	0	0
0	0	0	0	0
0	0	0	0	0
0	0	0	0	0
0	0	0	0	0

5	2	2	2	2
0	0	0	0	0
0	0	0	0	0
0	0	0	0	0
1	0	0	0	0
1	0	0	0	0
1	0	0	0	0
1	0	0	0	0
1	0	0	0	0

1	0	0	0	0
1	0	0	0	0
1	0	0	0	0
1	0	0	0	0
1	0	0	0	0
0	0	0	0	0
0	0	0	0	0
0	0	0	0	0
1	0	0	0	0

1	0	0	0	0
1	0	0	0	0
1	0	0	0	0
3	3	3	3	4
1	0	0	0	0
1	0	0	0	0
0	0	0	0	0
0	0	0	0	0
0	0	0	0	0

0	0	0	0	0
2	1	0	0	0
0	0	0	0	0
0	0	0	0	0
0	0	0	0	0
0	0	0	0	0
0	0	0	0	0
0	0	0	0	0
0	0	0	0	0

0	0	0	0	0
0	0	0	0	0
0	0	0	0	0
0	0	0	0	0
0	0	0	0	0
0	0	0	0	0
0	0	0	0	0
1	0	0	0	0
0	0	0	0	0

0	0	0	0	0
0	0	0	0	0
1	0	0	0	0
1	0	0	0	0
1	0	0	0	0
0	0	0	0	0
0	0	0	0	0
0	0	0	0	0
1	1	0	1	0

1	0	0	0	0
1	0	0	0	0
1	0	0	0	0
1	0	0	0	0
1	0	0	0	0
0	0	0	0	0
1	0	0	0	0
1	0	0	0	0
1	0	0	0	0

1	0	0	0	0
0	0	0	0	0
3	1	0	0	0
1	0	0	0	0
2	2	2	0	0
0	0	0	0	0
1	1	0	0	0
1	0	0	0	0
0	0	0	0	0

0	0	0	0	0
1	0	0	0	0

@7.¿Conquéfrecuenciaenelcursodelúltimoañohasteni	@8.¿Conquéfrecuenciaenelcursodelúltimoañonohasp	@9.Túoalgunaotrapersona¿Habéisresultadoheridosporque	@10.¿Algúnfamiliaramigomédicooprofesionalsanitarioha	Indiquesilehaocurridoalgunodelossiguientessucesoslos
0	0	0	0	0
0	0	0	0	0
0	0	0	0	0
0	0	0	0	1
0	0	0	0	1
0	0	0	0	0
0	0	0	0	0
0	0	0	0	0

0	0	0	0	0
0	0	0	0	0
0	0	0	0	1
0	0	0	0	0
0	0	0	0	0
0	0	0	0	0
0	0	0	0	1
0	0	0	0	0
0	0	0	0	0

0	0	0	0	0
0	0	0	0	1
0	0	0	0	0
3	3	0	0	0
0	0	0	0	0
0	0	0	0	1
0	0	0	0	0
0	0	0	0	0
0	0	0	0	1

0	0	0	0	0
0	0	0	0	0
0	0	0	0	1
0	0	0	0	1
0	0	0	0	0
0	0	0	0	0
0	0	0	0	0
0	0	0	0	0
0	0	0	0	0

0	0	0	0	1
0	0	0	0	0
0	0	0	0	0
0	0	0	0	0
0	0	0	0	1
0	0	0	2	0
0	0	0	0	0
0	0	0	0	0
0	0	0	0	1

0	0	0	0	0
0	0	0	0	0
0	0	0	0	1
0	0	0	0	0
0	0	0	0	1
0	0	0	0	1
0	0	0	0	0
0	0	0	1	0
0	0	0	0	1

0	0	0	0	0
0	0	0	0	0
0	0	0	0	0
0	0	0	0	0
0	0	0	0	1
0	0	0	0	1
0	0	0	0	1
0	0	0	0	0
1	0	0	1	0

0	0	0	0	0
0	0	0	0	0
0	0	0	0	1
0	0	0	0	0
0	0	0	0	0
0	0	0	0	0
0	0	0	0	0
0	0	0	0	0
0	0	0	0	1

0	0	0	0	0
0	0	0	0	1
0	0	0	0	0
0	0	0	0	1
0	0	0	0	1
0	0	0	0	0
0	0	0	0	0
0	0	0	0	0
0	0	0	0	0

0	0	0	0	0
0	0	0	0	1
0	0	0	0	0
0	0	0	0	1
0	0	0	0	1
0	0	0	0	1
0	0	0	0	0
0	0	0	0	0
0	0	0	0	0

0	0	0	0	0
0	0	0	0	0
0	0	0	0	0
0	0	0	0	0
1	0	0	2	0
0	0	0	0	0
0	0	0	0	1
0	0	0	0	0
0	0	0	0	0

0	0	0	0	0
0	0	0	0	0
0	0	0	0	0
0	0	0	0	0
0	0	0	0	0
0	0	0	0	0
0	0	0	0	0
0	0	0	0	0
2	0	0	0	1

0	0	0	0	0
0	0	0	0	0
0	0	0	0	0
2	2	0	2	0
2	0	0	2	1
0	0	0	0	1
0	0	0	0	0
0	0	0	0	0
3	2	0	0	0

0	0	0	0	0
0	0	0	0	0
0	0	0	0	0
0	0	0	0	0
0	0	0	0	0
0	0	0	0	0
0	0	0	0	1
0	0	0	0	0
0	0	0	0	0

0	0	0	0	0
0	0	0	0	0
0	0	0	0	0
0	0	0	0	0
0	0	0	0	0
0	0	0	0	0
0	0	0	0	0
0	0	0	0	0
0	0	0	0	0

0	0	0	0	0
0	0	0	0	0
0	0	0	0	0
0	0	0	0	1
0	0	0	0	0
0	0	0	0	0
0	0	0	1	0
0	0	0	0	1
0	0	0	0	0

0	0	0	0	0
0	0	0	0	1
0	0	0	0	1
0	0	0	0	0
0	0	0	0	0
0	0	0	0	1
0	0	0	0	0
0	0	0	0	0
0	0	0	0	0

0	0	0	0	0
0	0	0	0	0
0	0	0	0	0
0	0	0	0	0
0	0	0	0	1
0	0	0	0	0
0	0	0	0	1
0	0	0	0	0
0	0	0	0	0

0	0	0	0	1
0	0	0	0	0
0	0	0	0	0
2	2	0	2	0
0	0	0	0	0
0	0	0	0	0
0	0	0	0	0
0	0	0	0	0
0	0	0	0	0

0	0	0	0	0
0	0	0	2	0
0	0	0	0	0
0	0	0	0	0
0	0	0	0	0
0	1	0	2	1
0	0	0	0	0
0	0	0	0	0
0	0	0	0	0

2	2	2	2	1
0	0	0	0	0
0	0	0	0	0
0	0	0	0	0
0	0	0	0	1
0	0	0	0	0
0	0	0	0	0
0	0	0	0	0
0	0	0	0	0

0	0	0	0	0
0	0	0	0	0
0	0	0	0	0
0	0	0	0	1
0	0	0	0	0
0	0	0	0	0
0	0	0	0	0
0	0	0	0	0
0	1	0	0	0

0	0	0	0	0
0	0	0	0	0
3	0	0	0	0
0	0	0	0	0
0	0	0	0	0
0	0	0	0	0
0	0	0	0	0
0	0	0	0	0
0	0	0	0	0

0	0	0	0	0
0	0	0	0	0
0	0	0	0	0
0	0	0	0	0
0	0	0	0	0
0	0	0	0	1
0	0	0	0	0
0	0	0	0	0
0	0	0	0	0

0	0	0	0	0
0	0	0	0	0
0	0	0	0	0
3	0	0	2	1
0	0	0	0	0
0	0	0	0	0
0	0	0	0	0
0	0	0	0	0
0	0	0	0	0

0	0	0	0	1
0	0	0	0	0
0	0	0	0	0
0	0	0	0	0
0	0	0	0	0
0	0	0	0	1
0	1	2	2	0
1	1	2	2	0
0	0	0	0	0

1	0	0	0	0
1	0	2	2	0
0	0	0	0	0
0	0	0	0	0
0	0	0	0	0
0	0	0	0	0
0	0	0	0	1
0	0	0	1	0
0	0	0	0	0

0	0	0	0	0
0	0	0	0	0
1	0	0	0	0
0	0	0	0	0
0	0	0	0	0
0	0	0	0	0
0	0	0	0	0
0	0	0	0	0
0	0	0	0	0

2	2	0	2	1
0	0	0	0	0
0	0	0	0	0
0	0	0	0	1
0	0	0	0	0
0	0	0	0	0
0	0	0	0	0
0	0	0	0	0
0	0	0	0	0

0	0	0	0	0
0	0	0	0	0
0	0	0	0	0
0	0	0	0	0
0	0	0	0	0
0	0	0	0	0
0	0	0	0	0
0	0	0	0	0
0	0	0	0	0

0	0	0	0	1
0	0	0	0	0
0	0	0	0	0
0	0	0	0	0
0	0	0	0	0
0	0	0	0	0
0	0	0	0	0
0	0	0	0	0
0	0	0	0	0

0	0	0	0	0
0	0	0	0	0
0	0	0	0	1
0	0	0	0	1
0	0	0	0	0
0	0	0	0	0
0	0	0	0	0
0	0	0	0	0
0	0	0	0	0

0	0	0	0	0
4	0	1	1	1
0	0	0	0	1
0	0	0	0	0
0	0	0	0	0
4	1	2	2	0
0	0	0	0	0
0	0	0	0	1
0	0	0	0	0

0	0	0	0	0
3	1	2	2	1
1	0	0	2	0
0	0	0	0	1
0	0	0	0	0
0	0	0	0	1
0	0	0	0	0
0	0	0	0	0
0	0	0	0	1

0	0	0	0	0
0	0	0	0	0
0	0	0	0	0
0	0	0	0	0
0	0	0	0	1
0	0	0	0	0
0	0	0	0	0
0	0	0	0	1
0	0	0	0	1

0	0	0	0	0
0	0	0	0	0
0	0	0	0	0
0	0	0	0	0
0	0	0	0	0
0	0	0	0	1
0	0	0	0	1
0	0	0	0	0
0	0	0	0	0

0	0	0	0	0
0	0	0	0	1
0	0	0	0	1
0	0	0	0	0
0	0	0	0	0
0	0	0	0	0
0	0	0	0	0
0	0	0	0	0
0	0	0	0	0

0	0	0	2	1
0	0	0	1	0
0	0	0	2	1
0	0	0	0	0
0	0	0	0	0
0	0	0	0	0
0	0	0	0	0
0	0	0	1	0
0	0	0	0	0

0	0	0	0	1
0	0	0	0	0
0	0	0	0	0
0	0	0	0	0
0	0	0	0	0
0	0	0	0	0
0	0	0	0	0
0	0	0	0	0
0	0	0	0	0

0	0	0	0	1
0	0	0	0	0
0	0	0	0	0
0	0	0	0	0
0	0	0	0	0
0	0	0	0	0
0	0	0	0	1
2	0	1	0	0
0	0	0	0	0

0	0	0	0	0
0	0	0	0	0
0	0	0	0	0
0	0	0	0	0
0	0	0	0	0
0	0	0	0	0
0	0	0	0	0
0	0	0	0	0
0	0	0	0	0

2	2	2	2	0
0	0	0	0	0
0	0	0	0	0
0	0	0	0	0
0	0	0	0	0
0	0	0	0	0
0	0	0	0	0
0	0	0	0	0
0	0	0	0	0

0	0	0	0	0
0	0	0	0	0
0	0	0	0	1
0	0	0	0	0
0	0	0	0	1
0	0	0	0	0
0	0	0	0	0
0	0	0	0	0
0	0	0	0	0

0	0	0	0	0
0	0	0	0	0
0	0	0	0	0
3	3	0	2	0
0	0	0	0	0
0	0	0	0	0
0	0	0	0	0
0	0	0	0	0
0	0	0	0	0

0	0	0	0	1
0	0	0	0	1
0	0	0	0	0
0	0	0	0	0
0	0	0	0	0
0	0	0	0	0
0	0	0	0	0
0	0	0	0	1
0	0	0	0	1

0	0	0	0	1
0	0	0	0	0
0	0	0	0	1
0	0	0	0	1
0	0	0	0	0
0	0	0	0	0
0	0	0	0	0
0	0	0	0	0
0	0	0	0	0

0	0	0	0	0
0	0	0	0	0
0	0	0	0	0
0	0	0	0	1
0	0	0	0	0
0	0	0	0	0
0	0	0	0	0
0	0	0	0	0
1	1	1	1	0

0	0	0	0	0
0	0	0	0	0
0	0	0	0	0
0	0	0	0	0
0	0	0	0	0
0	0	0	0	0
0	0	0	0	0
0	0	0	0	0
0	0	0	0	1

0	0	0	0	1
0	0	0	0	0
0	0	0	0	0
0	0	0	0	0
2	2	0	0	0
0	0	0	0	1
0	0	0	0	0
0	0	0	0	1
0	0	0	0	0

0	0	0	0	0
0	0	0	0	1

Indiquesilehaocurridoalgunodelossiguientessucesoslos_A	Indiquesilehaocurridoalgunodelossiguientessucesoslos_B	Indiquesilehaocurridoalgunodelossiguientessucesoslos_C	Indiquesilehaocurridoalgunodelossiguientessucesoslos_D	Indiquesilehaocurridoalgunodelossiguientessucesoslos_E
0	0	0	0	0
0	0	1	0	0
0	0	1	0	0
0	0	0	0	0
0	0	0	0	0
0	0	0	0	0
0	0	1	0	0
0	0	1	0	0

0	0	0	0	1
0	0	0	0	1
0	0	0	0	1
0	0	1	0	0
0	0	0	0	1
0	0	1	0	1
0	0	1	0	1
0	0	0	0	0
0	0	1	0	0

0	0	0	1	0
0	0	1	1	1
0	0	1	0	0
1	0	1	0	1
0	0	0	0	0
0	0	0	0	1
0	0	0	0	0
0	0	1	0	0
0	0	1	0	0

0	0	0	1	1
1	1	1	0	0
0	0	1	0	0
0	0	1	0	0
0	0	1	0	1
0	0	1	0	1
0	0	0	1	1
0	0	0	0	1
0	0	1	0	0

0	0	1	0	1
0	0	0	0	1
1	0	0	0	1
0	0	0	0	1
0	0	1	0	0
0	0	0	0	0
0	0	0	0	1
0	0	0	0	1
0	0	1	0	1

0	0	1	0	1
0	0	0	0	1
0	0	0	0	1
0	0	0	0	1
0	0	0	0	1
0	0	0	0	0
0	0	0	0	0
0	0	0	0	0
0	0	0	0	0

0	0	0	0	1
0	0	0	0	0
0	0	0	0	0
0	0	1	0	0
0	0	0	0	1
0	0	0	1	1
0	0	0	0	1
0	0	0	0	0
0	0	0	0	0

0	0	0	0	1
0	0	0	0	0
0	0	1	0	1
0	0	1	0	0
0	0	0	0	0
0	0	0	1	1
0	0	0	0	0
0	0	1	1	0
0	0	0	0	0

0	0	0	0	0
0	0	0	0	0
0	0	0	0	0
0	0	0	0	0
0	0	0	0	0
0	0	0	0	0
0	0	0	0	0
0	0	0	0	0
0	0	0	0	0

0	0	0	0	1
0	0	0	0	1
1	0	0	0	1
0	0	1	0	0
0	0	0	0	1
0	0	0	0	1
0	0	0	0	1
0	0	1	1	1
0	0	1	0	1

0	0	0	0	0
0	0	1	0	0
0	0	0	0	0
0	0	0	0	1
0	0	1	0	1
0	0	0	0	0
0	0	0	0	1
0	0	1	0	0
0	0	1	0	1

0	0	0	0	0
0	0	1	0	0
0	0	1	0	1
0	0	0	1	1
0	0	1	0	0
0	0	0	1	1
0	0	0	0	0
0	0	1	0	1
1	0	1	1	0

0	0	0	0	1
0	0	0	0	0
0	0	0	0	0
1	0	0	0	0
1	0	1	0	0
0	0	0	0	0
0	0	1	0	0
0	0	0	0	0
1	0	1	0	0

0	0	1	0	0
0	0	1	0	0
0	0	1	0	0
0	0	0	0	0
0	0	0	0	1
0	0	0	0	0
0	0	0	0	0
0	0	0	0	0
0	0	0	0	0

0	0	1	0	0
0	0	0	0	0
0	0	0	0	0
0	0	0	0	0
0	0	0	1	0
0	0	0	1	1
0	0	1	0	1
0	1	0	0	0
0	0	0	0	1

0	0	0	0	1
0	0	0	1	0
0	0	0	0	0
0	0	0	0	1
0	0	0	0	0
0	0	0	0	1
0	0	1	0	1
0	0	0	0	1
0	0	0	0	0

0	0	0	0	1
0	0	1	0	1
0	0	0	0	1
0	0	0	0	1
0	0	0	0	1
0	0	0	1	1
0	0	0	0	0
0	0	1	0	1
0	0	0	0	0

0	0	0	0	0
0	0	0	0	1
0	0	0	0	0
0	0	1	0	0
0	0	0	0	0
0	0	1	0	1
0	0	1	0	1
0	0	1	0	0
0	0	1	1	0

0	0	1	0	1
0	0	0	1	0
0	0	0	0	1
1	0	0	1	0
0	0	0	0	1
0	0	0	0	0
0	0	0	1	1
0	1	0	0	0
0	0	1	0	0

0	0	0	0	0
0	0	0	0	0
0	0	0	0	0
0	0	1	0	0
0	0	0	0	0
0	0	1	0	0
0	0	0	0	0
0	0	0	0	0
0	0	0	0	0

1	0	0	1	1
0	0	1	1	1
0	0	1	0	1
0	0	1	0	1
0	0	1	1	1
0	0	0	0	0
0	0	0	0	0
0	0	1	0	1
0	0	0	0	0

0	0	0	0	0
0	0	0	0	0
0	0	0	1	0
0	0	1	0	0
0	0	0	0	1
0	0	0	0	1
0	0	0	0	0
0	0	1	0	1
0	0	0	1	0

0	0	0	0	1
0	0	0	0	1
0	0	0	0	0
0	0	1	1	1
0	0	0	0	0
0	0	0	0	1
0	0	1	0	0
0	0	1	0	0
0	0	0	0	0

0	0	1	0	0
0	0	0	0	1
0	0	0	0	1
0	0	0	0	1
1	0	0	1	0
0	0	1	0	0
0	0	0	1	1
0	0	0	0	0
0	0	0	0	0

0	0	0	0	0
0	0	1	0	1
0	0	0	0	0
1	0	0	0	1
0	0	0	0	0
0	0	0	0	1
0	0	0	0	0
0	0	0	0	0
0	0	0	0	0

0	0	1	0	0
0	0	1	0	0
0	0	0	0	0
0	0	0	0	0
0	0	0	0	1
0	0	0	0	1
0	0	1	1	0
1	0	1	0	1
0	0	0	0	1

1	0	0	0	1
1	0	0	0	1
0	0	1	1	0
0	0	0	0	1
0	0	0	1	1
0	0	0	0	1
0	0	0	0	1
1	0	0	1	1
0	0	1	0	1

0	0	0	0	1
0	0	0	0	1
0	0	0	0	1
0	0	1	0	1
0	0	0	0	1
0	0	0	0	1
0	0	0	0	0
0	0	1	0	0
0	0	1	0	0

1	0	0	0	0
0	0	1	0	1
0	0	0	0	1
0	0	1	0	1
0	0	1	0	1
0	0	0	0	1
0	0	0	1	0
0	0	0	0	1
0	0	0	0	0

0	0	0	0	0
0	0	1	0	0
0	0	0	0	0
0	0	0	0	0
0	0	0	0	0
0	0	0	0	0
0	0	1	0	0
0	0	0	0	0
0	0	0	0	0

0	0	0	0	1
0	0	0	0	0
0	0	0	0	0
0	0	1	0	0
0	0	0	0	1
0	0	0	1	1
0	0	0	0	0
0	0	0	0	1
0	0	0	0	1

0	0	0	0	0
0	0	1	1	0
0	0	1	0	1
0	0	1	0	1
0	0	0	0	0
0	0	1	1	1
0	0	0	0	0
0	0	1	0	0
0	0	0	0	0

0	0	1	0	0
1	0	1	0	1
0	0	1	0	1
0	0	0	0	1
0	0	1	1	1
1	0	0	0	1
0	0	1	0	0
0	0	0	0	0
0	0	1	0	1

0	0	0	1	1
1	0	0	0	0
0	0	1	0	1
0	0	0	0	1
1	0	0	1	1
0	0	0	1	1
0	0	0	0	1
0	0	0	0	1
0	0	1	0	1

0	0	0	1	1
0	0	1	0	1
0	0	1	0	1
0	0	1	0	1
0	0	0	0	1
0	0	0	0	1
0	0	1	0	1
0	0	0	0	1
0	0	0	0	1

0	0	1	0	1
0	0	0	0	1
0	0	0	0	1
0	0	0	0	0
0	0	0	0	1
0	0	0	1	1
0	0	0	0	1
0	0	0	0	1
0	0	1	0	1

0	0	0	0	0
0	0	1	0	0
0	0	1	0	1
0	0	0	0	0
0	0	0	0	0
0	0	1	0	0
0	0	0	0	1
0	0	0	0	0
0	0	1	0	0

0	0	0	0	1
0	0	0	0	0
0	0	1	1	1
0	0	0	0	0
0	0	1	0	0
0	0	1	0	0
0	0	0	0	0
0	0	0	0	0
0	0	0	0	0

0	0	1	0	1
0	0	0	0	1
0	0	0	0	0
0	1	0	0	0
0	0	0	0	0
0	0	0	0	0
0	0	0	0	0
0	0	0	0	0
0	0	0	0	0

0	0	0	0	0
0	0	0	0	0
0	0	0	0	0
0	0	0	0	0
0	0	0	0	1
0	0	0	0	0
0	0	0	0	0
0	0	0	0	0
0	0	1	1	1

1	0	0	0	1
0	0	1	0	0
0	0	1	0	0
0	0	0	0	0
0	0	0	1	0
0	0	0	0	0
0	0	0	0	0
0	0	0	1	1
0	0	1	0	0

1	0	0	0	0
0	0	1	0	0
0	0	0	0	1
0	0	1	0	1
0	0	1	0	0
0	0	1	0	0
0	0	0	0	0
0	0	0	0	0
0	0	1	0	0

0	0	0	0	0
0	0	0	0	0
0	0	0	0	0
0	0	0	0	0
0	0	0	0	0
0	0	0	1	1
0	0	1	0	1
0	0	1	0	1
0	0	0	0	1

0	0	0	0	1
0	0	1	0	0
0	0	1	0	0
1	0	1	0	0
0	0	1	0	0
0	0	1	0	0
0	0	0	1	0
0	0	0	0	1
0	0	0	1	1

0	0	0	0	1
0	0	0	0	1
0	0	1	1	1
0	0	0	0	1
0	0	0	0	0
0	0	0	0	1
0	0	0	0	1
0	0	1	0	1
0	0	1	0	1

0	0	1	0	1
0	0	1	1	0
0	0	0	0	1
0	0	0	0	1
0	0	0	0	1
0	0	0	0	0
0	0	0	0	1
0	0	1	0	0
0	0	1	0	0

0	0	1	0	0
0	0	0	0	0
0	0	1	0	0
0	0	1	0	0
0	0	0	0	0
0	0	0	0	0
0	0	1	1	0
0	0	1	0	0
0	0	0	0	0

0	0	0	0	0
0	0	0	0	0
0	0	0	0	0
0	0	0	0	0
0	0	0	0	0
0	0	0	0	0
0	0	1	0	0
0	0	0	0	0
0	0	0	0	0

0	0	0	0	0
0	0	1	1	0
0	0	0	0	0
0	0	0	0	0
0	0	0	0	0
0	0	0	0	1
0	0	0	1	0
0	0	0	0	0
0	0	0	0	1

0	0	0	0	0
0	1	1	0	1

Indiquesilehaocurridoalgunodelossiguientessucesoslos_F	Indiquesilehaocurridoalgunodelossiguientessucesoslos_G	Indiquesilehaocurridoalgunodelossiguientessucesoslos_H	Indiquesilehaocurridoalgunodelossiguientessucesoslos_I	Indiquesilehaocurridoalgunodelossiguientessucesoslos_J
0	0	1	0	1
0	0	0	0	0
0	0	0	0	0
0	0	0	0	0
0	0	0	0	0
0	0	0	0	0
0	0	0	0	0
0	0	0	0	0

0	1	0	0	1
0	0	0	0	0
0	0	0	0	0
0	0	0	0	0
0	0	0	0	0
0	0	0	0	0
0	0	0	0	0
0	1	0	0	0
0	0	0	1	0

0	0	0	0	1
0	0	0	0	0
0	0	0	0	0
0	1	1	1	1
0	0	0	0	0
0	0	0	0	0
0	1	0	0	0
0	0	0	0	0
0	0	0	0	1

0	0	0	1	0
0	0	0	0	0
0	0	0	0	0
0	0	0	0	0
0	0	0	0	0
0	0	0	1	0
0	0	0	0	0
0	1	0	0	0
0	0	0	0	0

0	0	0	0	0
0	0	0	0	0
0	1	0	0	0
0	1	0	0	0
0	0	1	1	1
0	0	0	0	0
0	0	0	0	0
0	0	0	0	0
0	0	0	0	0

1	0	0	0	0
0	0	0	0	0
0	0	0	0	0
0	0	0	0	0
0	1	0	0	0
0	0	0	0	0
0	0	0	0	0
0	0	0	0	0
0	0	0	0	0

0	1	0	0	0
0	0	0	0	0
0	0	0	0	0
0	0	0	0	0
0	1	0	0	0
0	1	0	0	0
0	0	1	1	1
0	0	0	0	0
0	0	0	1	0

0	1	0	0	0
0	1	0	0	0
0	0	0	0	0
0	0	0	0	0
0	0	0	1	0
0	0	0	0	0
0	0	0	0	0
0	0	0	1	0
0	0	0	0	0

0	0	0	0	0
0	0	0	0	0
0	0	0	0	0
0	0	0	0	0
0	0	0	0	0
0	0	0	0	0
0	0	0	0	0
0	0	0	0	0
0	0	0	0	0

0	1	0	0	0
0	1	0	0	1
0	0	0	0	0
0	0	0	0	0
0	0	0	0	0
0	1	0	0	0
0	0	0	0	0
0	0	0	0	0
0	0	0	0	0

0	0	0	1	0
0	1	0	0	1
0	0	0	0	0
0	0	0	0	0
0	0	1	0	0
0	0	0	0	0
0	1	0	0	0
0	1	0	0	0
0	0	0	0	0

0	0	0	0	0
0	1	0	0	0
0	0	0	0	0
0	0	0	0	0
0	0	0	0	0
0	0	0	0	1
0	0	0	0	0
0	0	0	0	0
0	0	1	1	1

0	0	0	0	0
0	0	0	0	0
0	0	0	0	0
0	0	0	0	0
0	0	0	0	0
0	0	0	0	1
0	0	0	1	0
0	0	0	1	0
0	0	0	0	0

0	0	0	0	0
0	0	0	0	0
0	0	0	0	0
0	0	0	0	0
0	0	0	0	1
0	0	0	0	0
0	0	0	1	0
0	0	0	0	0
0	0	0	0	0

0	0	0	0	0
0	1	0	1	0
0	0	0	0	0
0	0	0	0	1
0	0	0	0	0
0	0	0	1	0
0	0	0	0	0
0	1	0	1	1
0	0	0	1	0

0	0	0	1	0
0	0	1	0	1
0	0	1	0	0
0	1	0	1	0
0	0	0	0	0
0	1	0	0	0
0	0	0	0	0
0	0	0	0	0
0	0	0	0	0

0	1	0	0	0
0	0	1	0	0
0	0	0	0	0
0	0	0	0	0
0	0	0	0	0
0	0	0	0	0
0	1	0	0	0
1	0	1	0	1
0	1	0	0	0

0	1	0	0	0
0	1	0	0	0
0	1	0	0	0
0	0	0	0	0
0	0	0	0	0
0	0	0	0	0
0	1	0	0	0
0	0	0	0	0
0	0	0	0	1

0	0	0	0	0
0	0	1	0	0
1	1	0	0	0
0	1	0	0	0
0	1	0	1	0
0	0	0	0	0
0	0	0	1	0
0	1	0	0	0
0	0	0	1	0

0	0	0	0	0
0	0	0	0	0
0	0	0	0	0
0	0	0	0	0
0	0	0	0	0
0	0	0	0	0
0	0	0	0	1
0	0	0	0	0
0	0	0	0	0

0	1	1	0	1
0	0	0	0	0
0	0	0	0	0
0	1	0	0	0
0	1	1	0	0
0	1	0	0	0
0	0	0	0	0
0	0	0	0	0
0	0	0	0	1

0	0	0	0	0
0	1	0	0	0
0	0	0	1	0
0	1	0	0	0
0	1	0	0	0
0	0	0	0	0
0	1	0	0	0
0	0	0	0	0
0	0	0	0	1

0	1	0	0	0
0	0	0	0	0
0	0	1	1	0
0	0	0	0	1
0	0	0	0	0
0	1	0	0	0
0	1	1	1	1
0	0	0	0	0
0	0	0	0	0

0	0	0	0	0
0	0	0	0	0
0	1	0	0	0
0	0	0	0	0
0	0	1	0	1
0	0	0	1	0
0	0	0	0	1
0	0	0	0	0
0	1	0	0	0

0	0	0	0	0
0	0	0	0	0
0	0	0	0	0
0	0	0	0	0
0	0	0	0	0
0	0	0	0	0
0	0	1	1	0
0	0	0	0	0
0	0	0	0	0

0	0	0	0	0
0	0	0	0	0
0	0	0	0	0
0	0	0	0	1
0	0	0	1	0
0	0	0	0	0
0	1	0	0	0
0	0	0	1	0
0	0	0	0	0

0	0	1	0	1
0	0	1	0	0
0	1	0	0	1
0	1	0	0	0
0	1	1	0	1
0	0	0	0	0
0	0	0	0	0
0	0	0	0	0
0	0	0	1	0

0	1	0	0	0
0	0	0	0	0
0	1	0	0	0
0	1	0	1	1
0	0	0	0	0
0	0	0	0	0
0	1	0	0	0
0	0	0	0	0
0	0	0	0	1

0	0	0	0	0
0	1	0	0	0
0	0	0	0	0
0	1	0	0	0
0	0	0	0	0
0	1	0	0	0
0	0	0	1	0
0	0	0	0	0
0	0	0	0	0

0	0	1	0	0
0	0	0	0	0
0	0	0	0	0
0	0	0	0	0
0	0	0	0	0
0	0	0	0	0
0	0	0	0	0
0	0	0	0	0
0	0	0	0	0

0	1	0	0	0
0	1	0	0	0
0	0	0	0	0
0	1	0	0	0
0	1	0	0	0
0	1	0	0	0
0	1	0	0	0
0	0	0	0	0
0	1	0	0	0

0	0	0	0	0
0	0	0	0	0
0	0	0	0	0
0	0	0	0	0
0	0	0	0	0
0	0	0	0	0
0	0	0	0	0
0	0	0	0	0
0	0	0	0	0

0	0	0	0	0
0	0	0	0	0
0	1	0	0	0
0	1	0	0	0
0	0	0	1	1
0	0	0	0	0
0	1	0	0	0
0	0	0	0	0
0	0	0	1	0

0	1	0	0	0
0	1	0	0	0
0	0	0	0	0
0	0	0	0	0
0	1	0	0	0
0	0	0	0	0
0	1	0	0	0
0	0	1	1	1
0	1	1	0	1

0	0	1	0	0
0	0	0	0	0
0	0	0	0	0
0	0	0	0	0
0	1	0	0	0
0	0	0	0	0
0	1	0	0	0
0	0	0	0	0
0	0	0	0	0

0	1	0	0	0
0	0	0	0	0
0	0	0	0	0
0	0	0	0	0
0	1	0	0	0
0	1	1	0	1
0	1	0	1	0
0	0	0	0	0
0	0	0	0	0

0	0	0	0	0
0	0	0	0	0
0	0	0	0	0
0	0	0	0	0
0	0	0	0	0
0	0	0	1	1
0	1	0	0	0
0	0	0	0	0
0	0	0	0	0

0	0	0	0	0
0	0	0	0	0
0	0	0	0	0
0	0	0	0	0
0	1	0	0	1
0	1	0	0	0
0	0	0	0	0
0	1	1	1	0
0	0	0	0	0

0	1	0	0	1
0	0	0	0	0
0	0	0	0	0
0	0	0	0	0
0	0	0	0	0
0	0	0	0	0
0	0	0	0	0
0	0	0	0	0
0	0	0	0	0

0	0	0	0	0
0	0	0	0	0
0	0	0	0	0
0	1	0	0	0
0	0	0	0	0
0	0	0	0	0
0	0	0	1	0
0	0	0	0	0
0	1	0	0	1

0	1	0	1	0
0	0	0	0	0
0	0	0	0	0
0	0	0	1	0
0	0	0	0	0
0	0	1	0	0
0	0	0	0	0
0	0	0	0	0
0	0	0	0	1

0	0	0	1	0
0	0	0	0	0
0	0	0	0	0
0	0	1	0	0
0	0	0	0	0
0	0	0	0	0
0	0	0	0	0
0	0	0	0	0
0	0	0	0	0

0	0	0	0	0
0	0	0	0	0
0	0	0	0	0
0	0	0	0	0
0	0	0	0	0
0	1	0	0	0
0	0	0	0	1
0	0	0	0	0
0	0	0	0	0

0	0	0	0	0
1	1	0	0	0
0	0	0	0	0
0	0	0	0	0
0	0	0	0	0
0	0	0	0	0
0	0	1	0	0
1	1	0	0	0
0	0	0	0	0

0	0	0	0	0
0	0	0	0	0
0	1	1	1	1
0	0	1	0	0
0	1	1	1	1
0	0	0	0	0
0	0	0	0	0
0	0	0	1	0
0	1	1	1	1

0	1	0	0	0
0	0	0	0	0
0	0	0	0	0
0	0	0	0	0
0	0	0	0	0
0	0	0	0	0
0	0	0	0	0
0	0	0	0	1
0	0	0	0	0

0	0	0	0	0
0	0	0	0	0
0	0	0	0	0
0	1	1	0	1
0	0	0	0	0
0	0	0	0	0
0	1	1	0	1
0	0	0	0	0
0	0	0	0	0

0	0	0	1	0
0	0	0	0	0
0	0	0	0	0
0	0	0	0	0
0	0	0	0	0
0	0	0	0	0
0	0	0	0	0
0	0	1	0	0
0	0	0	0	0

0	0	0	0	0
0	1	0	0	0
0	0	1	0	1
0	0	0	0	0
0	0	0	0	0
0	0	0	0	0
0	0	0	0	0
0	0	0	0	0
0	0	0	0	0

0	0	0	0	0
0	0	0	0	0

Indiquesilehaocurridoalgunodelossiguientessucesoslos_K	Indiquesilehaocurridoalgunodelossiguientessucesoslos_L	Indiquesilehaocurridoalgunodelossiguientessucesoslos_M	Indiquesilehaocurridoalgunodelossiguientessucesoslos_N	Indiquesilehaocurridoalgunodelossiguientessucesoslos_O
0	0	0	0	0
0	0	0	0	0
0	0	0	0	0
0	0	0	0	0
0	0	0	0	0
0	0	0	0	0
0	0	0	0	0
0	0	1	0	0

0	0	0	0	0
0	0	0	0	0
0	0	0	0	0
0	1	0	1	0
0	0	0	0	0
0	0	0	0	0
0	1	0	0	0
0	0	0	0	0
0	0	0	0	0

1	0	0	0	1
0	0	0	0	0
0	0	0	0	0
0	0	0	0	0
0	0	0	0	0
1	0	0	0	0
0	0	0	0	0
0	0	0	0	0
0	0	0	0	0

0	0	1	0	0
1	0	0	0	0
0	0	0	0	0
0	0	0	0	0
0	1	0	0	0
0	0	0	0	0
0	0	0	0	0
0	0	0	0	0
1	0	0	0	0

0	0	1	0	0
0	0	0	0	0
0	0	0	0	0
1	0	0	0	0
0	0	0	0	0
0	0	0	0	0
0	0	0	0	0
0	0	0	0	0
0	0	0	0	0

0	0	0	0	1
0	0	0	1	0
0	0	0	0	0
1	0	0	0	0
0	0	0	0	0
0	0	0	0	0
0	0	0	0	0
0	0	0	0	0
0	0	0	0	0

0	0	0	0	0
0	0	0	0	0
0	0	0	0	0
1	0	0	0	0
0	0	0	1	0
1	0	0	0	0
1	0	0	0	0
0	0	0	0	0
0	0	0	0	0

0	0	0	0	0
0	0	0	0	0
0	0	0	0	0
0	0	1	0	0
0	0	0	0	0
0	0	0	0	0
0	0	0	0	0
1	0	0	0	0
0	0	0	0	0

0	0	0	0	0
0	0	0	0	0
0	0	0	0	0
0	0	0	0	0
0	0	0	0	0
0	0	0	0	0
0	0	0	0	0
0	0	0	0	0
0	0	0	0	0

0	0	0	0	0
0	0	0	0	0
0	0	0	0	1
0	0	0	0	0
1	1	0	0	0
1	0	0	0	0
0	0	0	0	0
0	0	0	0	0
0	0	0	0	0

1	0	0	1	1
0	0	0	0	0
0	0	0	1	0
0	0	0	0	0
0	0	0	0	0
0	0	0	0	0
0	0	1	0	1
0	0	0	0	0
0	0	0	0	0

0	0	0	0	0
1	0	0	0	1
0	0	0	0	0
1	1	0	0	0
0	0	0	0	0
0	0	0	1	1
0	0	0	0	0
0	0	0	0	0
0	0	0	0	0

1	0	0	0	0
0	0	0	0	0
0	0	0	0	0
0	0	0	0	0
0	0	0	0	1
1	0	0	0	0
0	0	0	0	0
0	0	0	0	0
1	0	0	0	0

1	0	0	0	0
0	0	0	0	0
0	0	0	0	0
0	0	0	1	1
0	0	0	0	0
0	0	0	0	0
0	0	0	0	0
0	0	0	0	0
0	0	0	0	0

0	0	0	0	0
0	0	0	0	0
0	0	0	0	0
0	0	0	0	0
0	0	0	0	0
0	0	0	0	0
0	0	0	0	0
0	1	0	0	1
1	0	0	1	1

0	0	0	0	0
0	0	0	0	0
0	0	0	0	0
0	0	0	0	0
0	0	0	1	1
0	0	0	1	1
0	1	1	0	1
0	0	0	0	0
0	0	0	0	0

0	0	1	1	1
1	0	0	0	0
0	0	0	0	0
0	0	0	1	1
0	0	0	0	0
0	0	0	0	0
0	0	0	0	0
0	1	1	1	1
1	0	0	0	0

0	0	0	0	0
1	1	0	0	1
0	0	0	0	0
1	0	0	0	0
0	0	0	0	0
0	0	0	0	0
0	0	0	0	0
0	0	0	0	0
1	0	0	1	1

0	0	0	0	0
0	0	0	0	0
0	0	0	0	0
1	0	1	0	1
0	0	0	0	0
0	0	0	1	1
0	0	0	0	0
0	0	1	0	1
1	0	0	1	1

0	0	0	0	0
0	0	0	1	0
0	0	0	1	1
0	0	0	0	0
0	0	0	0	0
0	0	0	0	0
0	0	0	0	0
0	0	0	0	0
0	0	0	0	0

0	0	0	0	0
0	0	0	0	0
0	0	0	0	0
0	0	0	0	0
0	0	0	0	0
0	0	0	0	0
0	0	0	0	0
0	0	0	0	0
1	0	0	0	0

0	0	0	0	1
0	0	0	0	0
1	0	0	0	1
0	0	0	0	0
0	0	0	0	0
0	0	0	0	0
0	0	0	0	0
1	0	0	0	0
1	0	0	1	0

0	0	0	0	0
0	0	0	0	0
0	0	0	0	0
1	0	1	1	1
0	1	0	0	0
1	0	0	0	0
1	0	0	0	0
0	0	0	0	0
0	0	1	0	0

0	0	0	0	0
0	0	0	0	0
0	0	0	0	0
0	0	0	0	0
0	0	0	0	0
0	0	0	0	0
0	0	0	0	0
0	0	0	1	0
0	0	0	0	0

0	0	0	0	0
0	0	0	0	0
0	0	0	0	0
0	0	0	0	0
0	0	0	0	0
0	0	0	0	0
0	0	0	0	0
1	0	0	0	0
0	0	0	0	0

0	0	0	0	0
0	0	0	0	0
0	0	0	0	0
1	0	0	0	0
1	0	0	0	0
0	0	0	0	0
0	0	0	0	0
0	1	0	0	0
0	0	0	0	0

0	0	0	0	0
0	0	0	0	0
0	0	0	1	1
0	0	0	0	0
0	0	0	1	1
1	0	0	0	0
1	0	0	0	0
0	0	0	0	0
0	0	0	0	0

0	0	0	0	0
0	0	0	0	1
0	0	0	0	0
0	0	0	0	0
1	0	0	0	1
0	1	0	1	1
0	0	1	0	0
0	0	0	0	0
0	0	0	0	0

0	0	0	0	0
1	0	0	0	0
0	0	0	0	0
1	0	0	1	0
0	0	0	0	1
0	0	0	0	0
0	0	0	0	0
0	0	0	0	0
0	0	0	0	1

0	0	0	0	0
0	0	0	0	0
0	0	0	0	0
0	0	0	0	0
0	0	0	0	0
0	0	0	0	0
0	0	0	0	0
0	0	0	0	0
0	0	0	1	1

0	0	0	0	0
1	0	0	0	0
0	0	0	0	0
0	0	0	0	0
0	0	0	0	0
0	0	0	0	0
0	0	0	0	0
0	0	0	0	0
0	0	0	0	0

0	0	0	0	0
0	0	0	0	0
0	0	0	0	0
0	0	0	0	0
0	0	0	0	0
0	1	0	0	0
0	0	0	0	0
1	0	0	0	0
0	0	0	0	0

0	0	0	1	1
0	0	0	0	0
0	0	0	0	0
0	0	0	1	1
1	0	0	0	0
0	1	1	0	0
0	0	0	1	1
0	0	0	0	0
0	0	0	0	0

0	0	0	0	0
1	0	0	0	0
0	0	0	1	1
1	0	0	0	0
0	0	0	0	0
0	0	0	0	0
1	0	0	0	0
1	0	0	0	0
0	0	0	0	0

1	0	0	0	1
1	0	0	0	0
0	0	0	0	0
0	0	0	0	0
1	0	0	0	0
0	0	0	0	0
0	0	0	0	0
0	0	0	0	0
0	0	0	0	0

0	0	0	0	0
0	0	0	0	0
0	0	0	0	0
0	0	0	0	0
0	0	0	0	0
0	0	0	1	0
0	0	0	1	1
0	0	0	0	0
1	0	0	0	0

1	0	0	0	0
0	0	0	0	0
1	0	0	0	0
0	0	1	0	0
0	0	0	0	1
0	0	0	0	0
0	0	0	0	0
0	0	0	0	0
0	0	0	0	1

0	0	0	0	0
0	0	0	0	0
1	0	0	0	0
0	0	0	0	0
0	0	0	0	0
0	1	0	0	0
0	1	0	0	0
0	0	0	0	0
0	0	0	0	0

0	0	0	0	0
0	0	0	0	1
0	0	0	0	0
0	0	0	0	0
0	0	0	0	0
0	1	0	0	0
0	0	0	0	0
0	0	0	0	0
0	0	0	1	0

0	0	0	0	0
0	0	0	0	0
0	0	0	0	0
0	0	0	0	0
0	0	0	0	0
0	0	0	0	0
0	0	0	0	0
0	0	0	0	0
0	0	0	1	1

0	0	0	0	1
0	0	0	0	0
0	0	0	0	0
0	1	0	0	1
0	0	0	0	0
0	0	0	0	0
0	0	0	0	0
1	0	0	1	1
1	0	0	0	1

1	0	0	0	0
1	0	0	0	1
0	1	0	0	0
0	1	0	0	0
0	0	0	0	0
0	0	0	0	0
0	0	0	0	0
0	0	0	0	0
0	0	0	0	0

0	0	0	0	0
0	0	0	0	0
0	0	0	0	0
0	0	0	0	0
0	0	0	0	0
0	0	0	0	0
0	0	0	0	0
0	0	0	0	0
0	0	0	0	0

0	1	0	0	0
0	0	0	0	0
0	0	0	0	0
1	0	0	0	0
1	0	0	0	1
0	0	0	0	0
1	0	0	1	0
0	0	0	0	0
0	0	0	0	1

0	0	0	0	0
0	0	0	0	0
1	0	0	0	0
0	0	0	0	0
1	0	0	0	0
0	0	1	0	0
1	0	0	0	0
1	0	0	0	0
0	0	0	0	0

0	0	0	0	0
0	1	0	0	0
0	0	0	0	0
1	0	0	0	0
0	0	0	1	0
0	1	0	0	0
1	0	0	0	0
0	0	0	0	0
0	0	0	0	0

0	0	0	0	0
0	0	0	0	0
0	0	0	0	0
0	0	0	0	0
0	0	0	0	1
0	0	0	0	0
0	0	0	0	0
0	0	0	1	0
0	0	0	0	0

0	0	0	0	0
0	0	0	0	0
0	0	0	0	0
0	0	0	0	0
0	0	0	0	0
0	0	0	0	1
0	0	0	0	0
0	0	0	0	0
0	0	0	0	0

0	0	0	0	0
0	0	0	0	0
0	0	0	0	0
0	0	0	0	0
0	0	0	0	0
0	0	0	0	0
0	0	0	0	1
0	0	0	0	0
0	0	0	0	0

0	0	0	0	0
1	0	0	0	0

Indiquesilehaocurridoalgunodelossiguientessucesoslos_P	Indiquesilehaocurridoalgunodelossiguientessucesoslos_Q	Indiquesilehaocurridoalgunodelossiguientessucesoslos_R	Indiquesilehaocurridoalgunodelossiguientessucesoslos_S	@1.Mellevobienconotros
0	0	0	1	0
0	0	1	1	0
1	0	0	1	0
0	0	0	0	0
0	0	0	0	0
0	0	1	0	0
0	0	0	1	0
1	0	0	1	1

0	0	0	1	0
0	0	0	0	0
0	0	0	1	0
0	0	0	1	0
0	0	0	0	0
0	0	0	0	1
0	0	0	0	0
0	0	0	1	0
0	0	0	1	0

0	0	1	1	2
0	0	0	0	0
0	0	1	0	0
0	0	1	0	0
1	0	0	0	0
0	0	0	0	0
0	0	0	1	0
0	0	0	0	0
0	0	0	1	0

0	0	1	0	2
0	0	0	1	0
0	0	0	0	0
0	0	0	0	0
1	0	0	1	1
0	0	0	0	1
0	0	0	1	0
0	0	1	1	0
0	0	0	0	0

0	0	0	0	0
1	0	0	1	0
0	0	0	0	0
0	0	0	1	0
0	0	0	1	0
0	0	0	0	0
0	0	0	0	0
0	0	0	0	0
0	0	0	0	0

0	0	0	0	3
0	0	0	1	1
0	1	0	1	2
0	0	1	1	1
0	0	1	1	2
0	0	0	1	1
0	0	0	0	2
0	0	1	0	2
0	0	0	0	0

0	0	0	0	2
1	0	0	0	0
0	0	0	0	0
0	0	0	0	0
0	0	0	0	0
0	0	0	0	0
0	0	1	1	0
0	0	0	0	0
0	0	1	1	0

0	0	0	0	0
1	0	0	0	0
1	0	0	0	0
0	0	0	0	0
1	0	0	0	0
0	0	0	0	0
0	0	0	1	2
0	0	0	1	2
0	0	0	0	0

0	0	0	0	0
0	0	0	0	2
0	0	0	0	0
0	0	0	1	2
0	0	0	1	2
0	0	0	0	0
0	0	0	0	0
0	0	0	0	0
0	0	0	1	0

0	0	0	0	0
0	0	0	1	0
0	0	0	1	0
0	0	0	0	0
0	0	0	0	0
0	0	0	0	0
0	0	0	0	0
0	0	0	1	0
1	0	0	0	2

0	0	0	0	2
0	0	1	0	0
0	0	0	0	0
0	0	0	1	2
0	0	0	1	0
0	0	1	0	0
0	0	1	0	0
0	0	0	0	0
0	0	1	0	0

0	0	1	1	2
0	0	0	0	0
0	0	0	0	0
0	0	0	1	0
1	0	1	0	0
0	0	0	1	0
0	0	1	0	0
0	0	0	0	0
0	0	0	1	4

0	0	1	1	0
0	0	0	0	0
0	0	1	1	0
0	0	0	0	0
0	0	0	0	2
0	0	0	1	0
0	0	0	1	0
0	0	0	1	0
0	0	0	1	0

0	0	0	0	0
0	0	0	0	0
0	0	0	0	0
0	0	0	0	0
0	0	0	1	0
0	0	0	0	0
0	0	1	1	1
0	0	0	0	0
1	0	0	0	0

0	0	0	0	0
0	1	0	0	0
0	0	0	1	2
0	0	1	1	2
0	0	0	0	0
0	0	0	1	2
0	0	0	0	0
0	0	0	0	0
0	0	0	0	0

0	0	0	1	0
0	0	0	1	0
0	0	0	1	1
0	0	0	1	1
0	0	0	0	2
0	0	0	1	0
0	0	0	1	0
0	0	0	0	0
0	0	0	0	1

0	0	0	0	0
0	0	0	1	2
0	0	0	0	1
0	0	0	0	0
0	0	1	0	2
0	0	0	0	1
0	0	0	1	2
1	1	1	0	0
0	0	0	0	0

0	0	0	0	0
0	0	0	0	0
0	0	0	0	0
0	0	0	0	1
0	0	0	1	0
0	0	0	1	0
0	0	0	1	0
0	0	0	0	0
0	0	0	0	2

0	1	0	1	0
0	0	0	0	1
0	0	1	0	0
0	0	1	1	1
0	0	1	0	0
0	0	0	0	0
1	0	0	1	0
0	0	0	0	0
0	0	0	0	0

0	0	0	1	0
0	0	0	1	0
0	0	0	0	2
0	0	0	0	0
0	0	0	0	0
0	0	0	0	0
0	0	0	0	0
0	0	0	0	0
0	0	0	1	0

0	0	0	1	0
0	0	1	0	0
0	0	0	1	0
0	0	1	0	2
0	0	0	0	0
0	0	0	1	0
0	0	0	1	0
0	0	0	1	2
0	0	1	0	0

0	0	1	0	0
0	0	0	0	0
0	0	0	0	0
0	0	1	1	0
0	0	0	0	0
0	0	0	1	1
0	0	0	0	0
0	0	0	0	0
0	0	1	0	0

0	0	0	0	0
0	0	1	0	0
0	0	0	0	0
0	0	0	1	0
0	0	0	1	0
0	0	1	0	0
0	0	1	0	0
0	0	0	0	0
0	0	0	0	2

0	0	0	0	0
0	0	0	1	0
0	0	0	0	0
0	0	0	0	0
0	0	1	1	2
0	1	0	0	2
0	1	1	0	2
0	0	1	1	1
0	0	1	0	0

0	0	1	1	2
0	0	0	0	0
0	0	0	0	0
0	0	1	0	2
0	0	0	1	0
0	0	1	0	0
0	0	0	1	0
0	1	1	1	2
0	0	0	1	0

0	0	0	0	0
0	0	1	1	0
0	0	0	0	0
0	1	0	1	0
0	0	0	1	0
0	0	0	0	0
0	1	0	0	0
0	0	0	1	2
0	0	0	0	0

0	0	1	1	0
0	0	1	1	0
0	0	0	1	2
0	0	0	1	2
0	0	0	1	2
0	0	0	0	2
0	0	0	0	0
0	0	1	1	0
0	0	0	0	0

0	0	0	0	0
0	0	0	1	1
0	0	0	1	1
0	0	1	1	2
0	0	1	0	2
0	0	0	1	1
0	0	0	0	0
0	0	1	0	2
0	0	0	0	0

0	0	0	0	0
0	0	0	1	2
0	0	0	0	0
0	0	0	0	0
0	0	0	0	0
0	0	0	1	0
0	0	0	1	0
0	0	0	0	0
0	0	0	0	0

0	0	0	0	3
0	0	0	0	3
0	0	1	0	0
0	0	0	1	0
0	0	0	1	0
0	0	0	1	0
0	0	0	1	2
0	0	0	1	0
0	0	0	0	3

0	0	0	0	0
0	0	1	1	2
0	0	0	1	0
0	0	0	1	0
0	0	0	1	0
0	0	0	0	2
0	0	0	0	0
0	0	1	0	1
0	0	0	0	0

0	0	0	0	0
0	0	0	0	0
0	0	0	1	0
0	0	0	0	0
0	0	1	1	0
0	0	0	0	0
0	0	0	0	0
0	0	0	0	0
0	0	0	0	0

0	0	1	1	4
1	0	0	0	0
0	0	1	0	0
0	0	0	0	0
0	0	1	1	1
0	0	0	0	0
0	0	0	1	1
0	0	0	0	0
1	0	0	0	0

0	0	1	0	0
0	0	0	1	1
0	0	1	1	1
0	0	0	1	2
0	0	1	0	0
0	0	0	1	0
0	0	0	1	0
0	1	1	0	0
0	0	0	1	0

0	0	0	1	1
0	0	0	1	0
0	0	0	1	0
0	0	0	1	0
0	0	0	1	0
0	0	0	0	1
0	0	0	1	2
0	0	0	1	0
0	0	0	0	0

0	0	0	0	0
0	0	0	0	0
0	0	0	1	0
0	0	0	1	0
0	0	0	1	1
0	0	1	0	0
0	0	1	1	3
0	0	1	0	0
0	0	0	1	1

0	0	0	0	0
0	0	0	0	0
0	0	0	0	0
0	0	1	0	1
0	0	0	1	0
0	0	0	0	0
0	0	0	1	0
0	0	0	0	0
0	0	0	0	1

1	0	0	0	2
0	0	0	1	0
0	0	0	1	2
0	0	0	0	0
0	0	0	1	0
0	0	0	1	0
0	0	1	0	0
0	1	1	0	0
0	0	1	0	1

0	0	0	1	0
1	0	1	0	0
1	0	0	0	0
0	0	0	1	0
0	0	0	0	0
0	0	0	0	0
0	0	0	0	0
0	0	0	1	0
0	0	0	0	0

0	0	0	0	0
0	0	0	0	0
0	0	0	1	0
0	0	0	0	0
0	0	0	0	0
0	0	0	0	0
0	0	0	0	0
0	0	0	0	4
0	0	0	0	2

0	0	1	0	0
0	0	0	0	0
0	0	0	0	0
0	0	0	1	0
0	0	1	1	0
0	0	1	0	2
0	0	0	0	0
0	0	0	0	0
0	0	1	1	3

0	1	0	0	0
0	0	0	0	2
0	0	1	1	1
0	0	1	1	0
0	0	0	0	0
1	0	0	0	2
0	0	0	0	0
0	0	0	0	0
0	0	0	0	0

0	0	1	0	2
0	0	0	1	0
0	0	1	0	0
0	0	0	0	2
0	0	0	1	0
0	0	0	1	2
0	0	0	0	0
0	0	0	0	0
0	0	0	1	2

0	0	0	0	2
0	0	0	0	0
0	0	0	0	2
0	1	1	0	1
0	0	0	0	0
0	0	0	1	2
0	0	0	0	2
0	0	0	0	1
0	0	0	0	0

0	0	0	0	0
0	0	0	0	0
0	0	1	0	0
0	0	0	1	0
0	0	1	1	2
0	0	0	1	0
0	0	0	1	2
0	0	1	1	0
0	0	0	1	0

0	0	0	1	1
0	0	0	1	0
0	0	0	0	0
0	0	0	1	2
0	0	0	0	2
0	0	0	0	0
0	0	0	0	2
0	0	0	1	0
0	0	0	0	0

0	0	0	0	0
0	0	1	0	2
1	0	0	0	0
0	0	0	0	0
0	0	0	0	0
0	0	0	1	0
0	0	0	0	0
0	0	1	0	2
0	0	1	0	0

0	0	0	1	0
0	0	0	0	3
0	0	0	0	0
0	0	1	0	0
0	0	0	1	0
0	0	0	0	0
0	0	0	0	0
0	0	0	0	0
0	0	1	0	0

0	0	0	0	0
0	0	0	0	2
0	0	1	0	0
0	0	0	1	2
0	0	0	1	0
0	0	0	0	0
0	0	1	0	0
0	0	1	1	0
0	0	0	0	0

0	0	0	0	0
0	0	0	0	0

@2.Mecansorápidamente	@3.Nadameinteresa	@4.Mesientopresionadoestresadoeneltrabajoescuela	@5.Mesientoculpable	@6.Mesientoirritadomolesto
4	4	4	2	0
4	4	0	2	1
2	1	2	2	1
3	3	2	3	3
4	2	0	1	1
2	4	0	2	0
2	2	1	3	4
4	2	0	2	2

4	2	3	4	4
1	3	0	1	2
2	3	4	1	3
2	2	4	2	2
3	3	1	1	2
4	2	2	2	4
2	2	0	4	2
3	2	0	4	4
4	2	4	0	4

2	2	4	4	3
4	4	4	3	4
2	3	0	2	2
4	4	0	2	0
4	2	4	2	2
4	3	4	1	2
4	3	0	3	4
1	0	0	0	0
2	3	2	2	2

2	1	2	3	3
4	2	2	3	3
3	3	2	0	2
3	3	4	4	1
4	4	0	0	2
0	1	0	3	3
4	4	1	1	4
1	4	1	4	4
2	2	3	3	2

2	1	2	1	2
2	0	2	0	2
4	4	4	4	4
2	2	2	2	3
4	2	0	4	2
2	0	2	2	2
3	2	2	0	2
2	1	3	2	1
4	2	2	2	2

3	4	4	4	4
2	2	3	3	2
3	4	4	4	3
4	4	0	4	4
3	2	4	3	4
3	4	3	4	4
4	4	3	0	4
3	1	4	1	3
3	2	1	0	4

4	4	3	2	4
4	4	3	0	2
2	0	2	0	2
3	0	4	0	0
4	4	3	4	4
4	2	0	4	4
3	0	4	0	0
4	0	0	3	0
4	4	4	4	4

4	1	4	0	4
4	1	0	0	0
4	1	0	2	0
2	1	0	0	1
2	4	3	2	0
4	2	4	4	4
3	4	4	4	4
4	4	4	4	4
2	3	0	0	2

3	3	4	0	0
3	1	0	0	4
0	0	2	0	2
4	1	2	0	0
4	2	4	0	4
4	4	4	4	4
2	4	4	4	2
4	0	0	0	0
2	4	4	3	2

4	4	4	4	4
4	3	3	4	2
4	4	4	4	4
4	4	0	0	0
3	2	4	4	4
3	3	3	3	3
1	3	0	2	2
3	3	4	4	2
4	4	3	4	4

3	3	4	0	3
2	3	0	4	0
3	2	4	4	3
4	2	0	1	4
4	4	3	4	4
2	0	0	4	2
4	2	4	4	0
4	0	4	0	2
4	4	4	0	4

0	0	3	3	2
2	4	4	4	4
2	1	2	2	2
0	4	4	4	4
0	2	0	0	4
3	4	4	4	4
2	2	2	0	0
2	4	4	2	3
4	4	4	4	4

2	2	0	1	0
1	0	2	0	2
0	3	3	4	2
4	4	3	4	4
3	2	4	2	2
4	3	4	2	2
2	2	0	2	2
2	2	3	3	1
1	1	0	4	2

4	3	3	2	4
2	3	3	0	1
2	0	0	0	3
4	4	4	1	2
3	4	0	0	2
1	3	0	2	2
2	1	0	4	3
3	2	0	2	2
4	2	0	0	2

4	2	0	4	3
4	4	4	4	4
0	2	4	3	3
3	3	4	4	4
3	2	2	0	2
4	0	4	4	4
2	2	0	2	2
3	2	4	1	3
3	2	2	2	2

1	2	0	1	1
1	2	1	2	2
2	2	1	2	4
4	1	0	4	3
4	4	4	1	2
3	3	4	2	3
2	0	2	2	1
3	4	4	4	2
3	1	2	3	3

4	2	4	2	4
3	3	4	2	4
4	4	2	1	1
2	0	2	0	2
2	2	0	0	3
4	2	4	2	4
3	0	0	0	3
1	3	4	4	4
4	4	4	4	4

4	2	4	3	4
4	1	4	0	0
0	4	4	4	4
2	2	4	0	3
4	2	0	1	1
2	1	1	0	0
4	2	2	4	4
1	0	0	0	3
4	4	2	4	2

4	4	4	4	4
3	3	2	0	3
3	3	4	2	2
4	4	3	2	0
2	1	2	2	2
2	1	4	3	3
4	4	3	0	2
3	4	4	2	4
2	2	4	2	2

2	0	4	0	4
2	2	0	2	2
0	2	2	2	4
4	2	1	2	1
3	2	2	0	2
4	2	2	0	3
2	4	4	4	4
4	2	4	2	4
2	0	4	2	3

4	4	1	0	4
2	2	3	3	2
4	2	2	0	0
4	4	4	4	4
2	1	0	1	1
4	3	4	4	0
3	1	1	2	2
4	4	4	4	4
0	2	4	0	2

3	3	4	0	2
4	4	0	4	0
4	0	4	0	3
2	3	2	2	2
4	2	2	2	2
3	2	4	2	4
4	4	4	0	4
4	4	4	4	4
4	2	2	2	4

4	4	4	4	4
2	2	2	2	2
4	2	4	4	2
0	4	3	3	3
4	2	3	0	0
2	2	2	0	2
2	2	0	0	2
4	4	4	4	3
3	0	2	4	0

2	2	0	0	2
2	2	2	0	4
4	3	3	3	3
0	0	0	0	0
4	2	2	4	3
2	2	0	2	2
4	2	4	4	4
3	3	4	4	2
2	0	1	4	4

0	2	0	0	0
0	0	0	2	2
0	0	0	0	0
3	2	4	4	2
3	4	0	2	2
3	1	0	0	2
3	1	4	4	0
4	3	0	4	3
4	4	4	2	4

3	3	0	0	2
2	2	2	4	1
4	2	2	4	4
0	2	0	3	0
3	2	2	3	3
2	2	3	2	3
4	4	4	4	4
4	4	0	4	4
3	2	3	1	3

4	4	1	2	2
1	1	0	4	2
3	4	1	0	4
2	3	4	2	3
4	4	4	2	4
3	3	3	4	2
3	4	4	0	2
4	3	4	4	1
3	3	3	2	3

3	2	1	0	3
4	2	3	2	2
4	4	4	4	3
4	4	4	4	4
4	0	4	3	1
4	2	4	1	2
2	1	0	0	2
4	2	3	3	3
2	0	2	3	1

4	2	3	3	4
2	2	2	0	2
3	0	4	0	0
4	3	2	0	3
0	2	4	1	4
3	2	3	3	4
4	4	2	4	4
4	4	0	2	4
2	4	0	4	0

4	2	0	0	4
4	2	4	4	4
0	3	4	2	3
4	4	4	3	4
1	0	0	4	2
2	2	3	2	2
4	3	0	0	2
3	3	0	2	4
4	4	4	3	3

3	4	0	2	4
3	3	0	3	3
3	2	0	0	3
4	0	0	0	3
4	2	0	0	3
1	3	3	0	4
4	4	1	0	4
2	2	0	2	4
3	2	0	3	3

4	1	4	0	0
4	2	1	0	2
0	4	4	4	0
4	0	0	0	0
3	0	0	3	2
4	0	4	4	4
4	0	4	0	4
4	4	4	0	3
0	0	0	0	0

4	4	0	4	4
3	3	3	4	0
4	2	2	2	3
3	2	4	4	4
2	0	4	4	3
0	2	0	2	2
3	4	4	4	3
3	2	4	0	2
4	4	0	0	2

3	2	2	2	3
2	2	4	2	3
4	4	3	4	2
2	3	4	0	3
2	2	0	4	2
3	2	4	3	4
1	2	2	0	2
3	2	2	3	3
4	2	4	4	2

3	2	2	3	3
2	2	1	0	2
3	2	2	2	3
1	3	4	0	2
3	1	2	1	1
3	3	0	2	4
1	3	2	2	3
4	0	2	2	1
2	2	0	1	0

4	3	4	4	3
3	3	0	1	2
4	2	4	2	4
3	2	4	2	3
0	2	2	1	1
4	4	4	3	4
4	4	2	4	4
2	2	2	0	1
3	3	3	4	1

1	3	0	4	3
4	0	2	2	3
2	2	3	0	2
1	1	0	1	4
2	3	0	0	2
1	4	3	1	3
0	0	0	4	4
4	3	0	2	3
4	2	3	4	4

3	3	2	0	4
4	3	2	0	4
0	4	0	4	4
0	0	0	0	0
2	3	0	4	3
4	4	4	4	3
3	4	4	0	2
4	4	0	3	4
3	4	4	2	2

2	1	4	3	4
2	2	3	0	3
3	3	0	0	2
0	4	0	0	2
3	4	3	0	4
4	4	4	3	4
3	2	2	0	0
4	4	4	0	4
4	4	4	4	4

3	1	0	0	2
2	0	3	0	2
3	4	0	2	4
4	4	4	2	4
4	1	0	0	0
2	0	0	0	0
3	2	4	0	2
4	4	2	4	4
3	2	4	2	4

4	1	4	4	4
4	0	0	1	0
4	2	4	4	2
4	4	4	4	4
2	0	2	0	2
1	2	3	2	2
3	3	4	0	2
4	4	3	4	2
1	2	2	4	4

1	2	2	2	2
4	4	4	4	4
4	0	2	4	4
4	4	4	0	0
4	4	4	4	4
3	4	1	2	0
2	2	0	2	4
2	0	0	0	0
2	4	4	3	4

4	4	0	0	4
4	4	4	4	4
4	4	4	4	4
4	4	4	0	0
4	4	2	2	2
3	4	4	3	3
2	0	0	1	2
3	3	3	0	0
3	0	3	0	3

3	3	3	3	3
0	0	2	2	2
4	3	4	0	4
3	1	0	0	3
4	4	4	4	3
2	2	2	1	2
2	3	3	4	4
4	3	4	4	4
2	2	2	4	4

3	3	0	3	0
2	2	4	0	2
0	4	4	3	0
4	4	4	4	4
4	3	4	4	4
0	2	1	2	2
4	4	4	4	2
2	4	3	2	2
4	4	4	4	2

0	2	2	2	2
4	4	4	4	4
0	3	0	0	0
4	4	4	4	4
4	4	4	2	4
0	3	2	2	0
4	4	0	2	0
4	4	4	2	4
0	0	4	4	0

4	0	4	4	4
4	4	4	4	4
1	3	0	0	0
4	4	4	2	3
0	4	4	0	4
0	0	4	4	0
0	0	2	2	4
3	2	2	3	2
4	3	2	1	3

4	4	0	4	4
4	0	4	4	2
2	2	4	0	2
4	4	4	2	0
4	4	2	2	4
4	4	2	0	4
4	4	4	0	4
4	0	2	0	3
2	2	1	0	4

4	4	0	4	0
3	3	3	3	4
3	0	0	2	0
2	3	2	2	3
3	3	0	4	4
3	4	4	4	4
4	3	4	2	1
4	4	2	3	3
3	0	4	2	2

0	0	0	0	0
4	4	2	4	4

@7.Mesientocontentoconmimatrimoniopareja	@8.Piensoenquitarmelavida	@9.Mesientodébil	@10.Mesientoatemorizado	@11.Necesitotomarbebidasalcohólicasenlamañanadespués
4	2	2	2	0
4	2	3	3	0
1	0	2	2	0
0	2	3	3	0
4	0	3	2	0
4	3	3	0	0
4	2	2	2	0
4	0	3	3	0

1	0	3	4	0
2	2	1	2	0
4	0	3	3	0
4	1	3	4	0
0	0	2	2	0
0	0	3	2	0
4	0	2	2	0
4	2	4	0	0
4	0	2	4	0

2	0	2	3	0
4	2	4	4	0
0	0	1	0	0
4	2	3	0	4
3	0	1	2	0
4	0	3	1	0
2	2	3	2	0
0	0	0	0	0
1	0	2	2	0

3	2	3	2	0
0	2	4	3	0
2	0	2	0	0
3	0	4	4	0
4	3	2	3	0
0	0	3	0	0
0	0	2	2	0
4	0	4	4	0
4	0	3	4	0

4	0	3	4	0
0	0	2	2	0
2	0	4	4	0
0	0	4	2	0
2	0	2	2	0
0	0	2	0	0
1	0	2	3	0
1	0	3	3	0
1	0	4	3	0

0	0	2	4	0
0	1	4	2	0
4	0	3	3	0
4	0	3	2	0
2	2	3	2	0
4	1	4	4	0
3	2	2	0	0
1	3	1	3	0
0	0	0	3	0

2	0	4	0	0
4	0	4	3	0
0	0	2	3	0
0	0	0	2	0
2	0	3	2	0
2	0	4	4	0
3	0	2	3	0
4	0	3	0	0
3	1	4	4	0

4	4	2	0	0
4	0	4	0	0
2	1	2	1	0
2	0	4	4	0
4	0	1	0	0
4	1	4	4	0
0	0	2	1	0
0	2	4	4	0
1	0	0	0	0

4	0	4	4	0
0	0	0	0	0
0	2	2	2	0
2	2	4	0	0
4	0	2	2	0
4	4	0	4	0
4	2	4	2	0
2	0	3	0	0
4	2	2	2	0

4	3	4	4	0
2	0	3	3	0
4	4	4	0	0
4	0	0	0	0
1	3	4	2	0
4	2	4	4	0
0	0	3	1	0
4	3	4	4	0
4	4	4	3	0

0	0	3	2	0
4	3	4	4	0
4	0	4	4	0
4	0	4	4	0
0	1	3	3	0
0	0	2	0	0
1	2	4	4	0
2	0	2	4	0
4	0	4	4	0

0	0	4	3	0
2	4	4	4	0
4	0	2	2	0
3	0	2	1	0
0	1	4	4	0
4	0	4	4	0
0	0	2	2	0
4	2	0	0	0
4	4	4	4	0

4	0	4	2	0
0	0	0	0	0
2	0	4	4	0
0	2	4	3	0
0	0	4	2	2
4	2	3	4	0
3	0	2	2	0
2	2	4	4	0
2	2	2	4	0

3	0	4	1	0
0	0	3	3	0
3	3	4	4	0
2	0	4	4	0
4	0	2	2	0
4	0	2	3	0
2	2	2	2	0
4	0	0	4	0
0	0	2	2	0

0	2	4	4	0
4	2	0	4	0
3	0	4	4	0
3	2	4	4	0
0	0	0	2	0
4	2	3	4	0
4	0	4	0	0
0	2	3	4	0
0	0	0	2	0

0	0	2	2	0
1	2	3	1	0
0	0	4	2	0
3	0	2	0	0
0	0	4	2	0
2	1	3	3	0
0	0	2	1	0
0	0	1	2	0
0	0	2	2	0

2	0	4	2	0
0	0	2	3	0
4	0	4	4	0
2	0	2	3	0
4	0	2	0	0
3	1	2	2	0
3	0	3	0	0
0	2	4	3	0
4	2	4	0	0

2	0	4	1	0
2	0	2	2	0
4	0	4	0	0
1	0	2	2	0
4	0	2	1	0
0	0	2	2	0
0	0	4	0	0
0	0	3	0	0
4	4	4	4	0

4	0	4	4	0
4	0	3	0	0
4	0	2	2	0
2	1	2	2	0
2	0	3	3	0
4	0	2	2	0
4	0	4	3	0
4	4	4	0	0
4	2	2	2	0

2	0	0	2	0
2	0	2	2	0
0	2	4	2	0
4	0	0	4	0
2	0	0	0	0
2	0	3	0	0
1	0	0	0	0
3	0	1	3	0
3	0	4	2	0

1	4	3	1	2
4	2	3	3	0
4	0	0	1	0
4	2	4	4	0
2	0	0	1	0
2	3	3	0	0
0	0	2	3	0
2	4	4	4	0
2	2	1	0	0

4	3	3	3	0
0	1	4	4	0
0	0	2	3	0
4	2	2	3	0
2	0	0	0	0
0	0	2	4	0
0	0	0	2	0
4	0	4	2	0
0	2	4	4	0

2	4	4	4	0
4	1	3	2	0
4	2	4	4	0
2	0	2	3	0
4	0	2	2	0
2	0	3	2	0
0	0	0	0	0
0	0	4	0	0
4	0	2	0	0

0	0	2	2	0
0	0	0	0	0
1	3	3	3	0
4	0	0	0	0
4	3	3	0	0
2	0	4	2	0
4	0	4	4	0
3	4	3	4	0
4	0	4	4	0

4	0	2	0	0
4	0	0	0	0
0	0	0	0	0
2	2	4	4	2
4	1	2	0	0
0	0	4	2	0
4	0	2	2	0
4	3	4	0	0
4	2	4	2	0

1	0	0	2	0
2	2	2	0	0
0	0	0	4	0
4	3	3	0	0
0	0	3	3	0
4	1	2	3	0
2	2	4	4	0
0	2	4	4	2
0	1	3	3	0

3	0	4	4	0
1	1	3	4	1
3	0	4	4	0
2	0	3	3	0
2	0	3	4	0
2	2	2	4	0
0	0	4	0	0
4	0	2	2	0
0	0	3	3	0

2	0	3	0	0
0	0	2	0	0
2	0	3	4	0
4	3	3	4	0
4	0	4	4	0
1	0	2	2	0
0	0	2	0	0
1	0	3	3	0
1	0	2	2	0

2	0	0	2	0
0	0	1	4	0
0	0	4	0	0
0	2	4	4	0
0	0	2	4	0
2	0	2	2	0
4	0	4	2	0
0	0	4	4	0
0	0	0	0	0

0	0	2	0	0
3	0	0	2	0
4	2	2	0	0
0	0	4	4	0
4	0	0	2	0
2	2	2	2	0
0	0	4	0	0
0	4	4	4	0
3	0	4	1	0

0	4	4	4	0
3	4	4	4	0
0	0	2	4	0
4	0	0	3	0
4	0	2	2	0
4	0	3	3	0
4	0	0	0	0
4	2	3	0	0
1	0	2	0	0

4	1	4	2	0
4	0	2	3	0
0	0	4	0	0
4	0	3	0	0
2	0	4	0	0
0	0	4	4	0
4	0	4	0	0
1	0	4	4	0
0	0	0	0	0

4	3	4	4	0
0	0	3	4	4
2	2	4	2	0
2	0	2	2	0
1	0	2	1	0
0	0	2	2	0
2	4	4	4	0
4	0	2	4	0
4	0	2	2	0

4	1	0	1	0
2	0	2	4	0
0	1	4	4	0
0	0	3	4	0
2	1	4	1	0
2	2	3	4	0
2	0	2	1	0
2	2	2	4	0
2	0	2	2	0

4	2	2	2	0
1	2	2	2	0
0	0	3	3	0
0	0	4	4	0
0	0	2	3	0
2	3	4	2	0
0	2	2	2	0
0	0	2	2	0
1	0	2	2	0

0	2	2	4	0
4	1	2	0	0
2	0	2	4	0
2	0	3	0	0
4	0	2	0	0
4	4	4	3	0
3	3	4	2	0
0	1	2	2	0
4	2	3	2	0

4	0	3	0	0
4	0	2	2	0
4	1	2	2	0
4	0	0	0	0
4	0	2	2	0
2	2	3	0	0
4	0	2	0	0
4	0	4	2	0
1	2	2	2	0

4	0	3	3	0
1	0	2	2	0
4	4	4	4	0
0	0	0	0	0
4	4	2	3	0
4	2	4	3	0
2	0	4	2	0
4	3	4	4	0
4	0	2	2	0

3	2	3	4	0
2	0	3	3	0
0	0	2	0	0
0	0	2	3	0
0	0	4	4	0
0	0	4	4	0
4	0	0	0	0
0	0	4	4	0
2	0	4	4	0

0	0	0	2	0
1	0	0	2	0
0	2	4	4	0
0	2	4	4	0
4	0	0	2	0
4	0	2	2	0
0	0	0	2	0
4	4	4	4	0
2	2	2	2	0

4	0	4	4	0
0	0	4	4	0
0	0	4	2	0
4	0	4	4	0
4	0	0	0	0
2	0	0	2	0
4	0	2	0	0
4	1	4	3	0
4	0	4	2	0

4	0	3	2	4
2	3	4	1	0
4	0	2	4	0
4	4	4	4	0
4	0	4	4	0
3	2	4	4	0
0	0	2	0	0
0	0	0	0	0
0	0	4	4	0

4	3	4	0	0
4	0	0	0	0
2	0	2	2	0
2	0	0	0	0
4	0	4	2	0
3	4	4	4	0
4	0	0	0	0
1	0	3	3	0
3	3	3	0	0

4	2	3	1	0
4	0	2	2	0
2	2	4	3	0
4	2	2	0	3
0	3	0	4	0
4	0	3	0	0
4	2	4	0	0
0	2	4	4	0
2	0	0	0	0

4	0	0	0	0
0	0	0	0	0
4	3	0	0	0
2	2	4	4	0
2	2	2	3	0
2	0	0	0	0
4	2	4	1	0
4	3	4	1	0
0	0	4	4	0

2	2	2	2	0
2	0	4	4	0
0	0	0	2	0
2	2	4	0	0
2	0	4	2	0
0	0	0	2	0
0	0	4	0	0
0	0	2	4	0
4	0	0	4	0

4	0	4	4	0
4	2	4	4	0
0	0	0	0	0
4	2	4	4	0
2	0	3	4	0
3	1	4	4	0
4	3	0	0	0
2	1	3	3	0
3	0	2	2	0

4	0	0	4	0
4	0	2	4	0
0	0	1	0	0
4	0	4	4	0
4	0	2	2	0
0	2	4	4	0
0	3	0	4	0
4	0	0	2	0
0	0	2	0	0

4	0	0	0	0
0	4	4	4	0
0	0	2	0	0
2	0	0	2	0
1	2	3	4	0
4	4	4	3	0
4	0	2	3	0
0	0	3	4	0
0	1	3	3	0

0	0	0	0	0
0	0	3	4	0

@12.Encuentrosatisfacciónenmitrabajoescuela	@13.Soyunapersonafeliz	@14.Trabajoestudioexcesivamentemásdelacuenta	@15.Mesientoinútil	@16.Meabrumananangustianlosproblemasdemifamilia
3	3	2	0	4
4	3	1	3	4
1	0	1	0	4
3	2	0	3	3
2	2	0	2	1
1	4	0	2	4
0	3	2	2	2
0	4	0	0	3

2	3	3	4	4
1	2	2	2	2
2	2	1	2	4
4	2	1	2	4
1	2	1	0	0
2	1	1	1	2
0	0	0	4	4
0	2	0	0	1
4	2	0	0	2

2	2	1	3	4
3	3	0	4	0
0	0	2	0	4
0	3	0	0	4
2	1	0	0	2
3	4	4	2	2
1	4	2	2	4
0	0	0	0	4
0	0	0	1	4

2	2	1	1	2
1	2	1	2	4
1	2	2	3	2
4	4	1	4	4
4	2	0	0	2
0	2	4	0	3
0	1	4	0	4
0	3	1	0	4
0	2	2	2	3

1	0	4	1	0
2	0	2	0	2
0	0	4	1	4
2	0	1	2	2
0	2	0	0	4
0	1	3	0	1
2	0	1	0	0
1	1	2	1	3
2	3	1	4	3

3	4	0	2	4
3	3	0	4	1
3	3	0	4	2
1	3	1	4	4
2	2	1	3	3
3	4	3	1	4
4	3	0	4	4
2	2	2	2	4
0	2	0	4	4

2	4	4	2	4
4	4	0	4	4
3	0	0	2	4
2	0	0	0	4
2	3	4	4	2
4	2	4	2	4
1	1	0	0	4
0	3	0	0	3
4	4	0	2	4

4	3	3	0	4
0	0	0	0	1
2	2	1	0	2
2	3	1	0	4
2	2	0	0	4
3	4	4	0	4
0	0	0	0	4
4	0	4	4	4
2	2	1	0	4

3	2	2	2	3
2	2	0	2	2
1	2	0	0	4
2	1	0	0	0
4	4	2	0	4
4	4	2	4	4
4	4	4	2	4
2	0	1	4	0
0	2	4	0	4

4	4	0	0	2
2	2	3	2	3
3	4	4	0	2
2	2	0	0	0
2	2	0	4	4
4	2	0	0	0
0	4	0	2	2
4	4	0	4	4
3	3	0	4	3

4	3	4	0	0
0	3	0	0	4
2	3	3	2	3
0	2	0	2	4
3	3	3	3	4
0	3	1	0	4
4	4	3	4	4
0	2	0	2	4
4	4	0	4	4

4	2	0	0	4
4	4	4	4	4
0	0	0	0	4
0	3	2	0	4
0	2	3	4	4
2	4	4	2	4
0	1	0	0	2
0	0	0	0	0
4	4	0	4	4

2	0	0	1	0
0	0	3	0	4
2	2	4	4	4
1	0	4	3	4
3	3	1	2	1
3	4	3	0	4
4	4	1	0	2
0	4	0	2	4
1	2	2	1	4

4	2	3	2	4
1	0	3	0	3
4	4	0	4	3
3	3	3	1	4
0	2	0	0	4
1	4	2	0	1
1	4	0	3	4
0	2	0	0	0
0	0	1	0	2

3	3	3	0	4
4	2	0	4	4
1	4	3	0	4
3	3	0	2	4
0	1	2	0	2
0	2	2	2	4
0	2	0	0	0
0	2	4	2	1
0	0	2	0	0

0	2	2	0	4
0	3	2	2	2
2	2	4	2	3
0	4	4	2	4
3	3	4	2	2
4	2	4	0	4
0	2	1	0	2
3	1	0	3	3
0	0	2	0	2

2	4	4	3	4
3	3	1	4	4
3	3	1	2	4
1	3	0	0	4
1	0	0	2	2
2	4	3	3	4
1	4	2	4	3
0	3	3	2	4
4	4	4	4	4

1	2	2	2	4
1	2	1	2	4
4	4	0	0	0
4	2	0	2	3
0	0	1	4	4
0	0	0	2	4
0	2	2	0	4
0	1	1	2	1
4	2	2	4	4

4	4	4	4	4
2	1	2	2	3
3	4	4	1	3
1	3	3	2	3
2	2	1	2	4
0	0	4	0	2
0	2	0	2	4
2	4	4	2	4
2	2	3	2	3

2	1	4	0	4
0	2	0	0	2
2	3	4	2	2
0	3	1	1	2
4	2	4	0	4
2	1	0	0	4
4	2	0	0	4
3	2	4	4	4
4	2	4	0	4

0	2	2	1	3
4	2	0	2	0
4	2	0	0	4
4	3	1	4	4
4	1	0	1	2
2	2	3	0	1
4	2	0	2	3
4	4	0	4	4
2	0	2	4	2

2	2	2	3	3
4	3	0	4	4
1	2	2	0	4
0	4	2	2	4
0	1	2	0	4
0	0	0	2	4
0	0	0	0	4
4	4	1	4	0
4	2	0	2	4

0	4	3	4	4
0	4	2	0	3
2	4	4	2	2
2	0	2	2	3
0	3	0	2	4
2	0	0	2	4
0	0	0	0	3
4	2	4	4	4
0	4	2	0	4

0	1	0	0	4
4	1	4	0	2
1	2	2	2	3
0	0	0	0	0
0	4	0	2	0
2	1	4	2	4
4	4	0	3	4
4	2	4	4	3
0	2	0	2	3

4	4	0	2	4
0	0	0	0	2
0	0	0	0	0
3	4	2	4	4
0	4	0	1	0
0	2	0	0	4
1	1	3	0	4
4	4	0	4	4
0	4	4	4	4

2	1	1	2	4
2	4	1	2	4
0	0	0	2	4
4	3	0	3	2
4	2	0	3	3
2	3	4	2	3
0	1	0	0	4
0	4	0	2	4
1	0	1	0	4

1	2	1	0	4
0	2	1	0	3
0	2	4	1	4
3	2	3	1	4
4	2	2	4	4
2	3	2	2	4
2	3	0	2	4
4	2	0	2	4
0	2	3	0	4

3	3	0	0	4
1	1	4	0	1
1	4	3	4	4
2	3	1	2	4
2	2	0	4	4
3	2	3	0	2
0	0	3	0	0
2	3	2	2	4
1	2	0	2	2

2	2	2	2	4
0	0	4	3	4
0	2	4	0	4
0	0	4	0	4
0	2	4	0	4
2	1	4	2	4
0	2	4	2	4
1	2	2	4	4
4	4	4	0	4

0	2	0	2	4
2	2	0	0	4
3	4	4	0	4
4	2	4	0	2
0	0	1	0	4
0	2	0	0	4
4	4	0	0	4
0	4	0	4	4
4	4	3	3	1

4	2	0	4	1
4	4	0	2	3
4	0	0	0	2
4	0	0	2	2
0	2	0	3	2
2	0	1	3	3
0	2	0	2	2
1	3	0	2	0
4	2	0	2	3

0	4	4	2	4
3	2	0	1	3
0	2	0	0	4
0	0	0	4	4
0	2	3	0	2
0	0	2	0	4
1	3	1	4	4
4	4	0	0	4
0	0	2	0	0

4	4	4	0	4
2	3	0	4	4
3	2	0	2	2
2	2	4	2	4
1	2	1	1	4
2	1	2	0	2
4	4	0	4	4
3	3	4	2	3
2	4	0	2	3

1	1	2	1	2
2	2	4	1	4
3	2	1	4	4
4	3	4	2	4
0	2	0	0	4
0	0	4	0	4
0	3	4	1	3
3	3	2	2	3
3	2	3	0	4

3	4	1	3	4
2	2	0	2	4
1	2	0	2	1
0	0	3	0	4
1	0	0	2	3
0	2	1	2	4
0	1	1	0	4
0	1	3	2	3
1	1	3	3	4

0	2	2	0	4
0	2	0	2	4
2	2	4	0	4
0	1	4	0	4
2	2	1	1	1
3	4	4	2	4
4	4	0	2	4
0	2	4	0	2
3	3	4	3	4

2	3	1	2	3
1	0	0	0	4
1	4	2	0	4
0	4	0	0	4
0	3	3	0	4
1	2	1	3	4
0	4	1	0	4
2	4	0	3	4
2	1	4	2	4

4	3	1	2	4
1	3	4	1	2
0	2	0	0	4
0	0	0	0	0
0	4	2	0	4
1	4	0	4	4
1	2	4	0	4
4	4	0	4	3
2	2	1	4	3

1	2	0	2	4
3	2	0	0	2
0	0	0	0	2
4	4	0	0	4
0	0	2	4	4
0	0	0	4	4
2	4	0	0	4
4	0	4	4	4
4	2	4	4	0

0	2	0	0	4
0	3	4	0	4
2	4	0	0	4
0	4	0	2	4
1	2	0	0	3
0	0	2	3	4
0	0	1	0	2
2	3	0	2	4
4	2	4	0	4

4	4	1	1	4
0	2	0	3	4
0	2	4	1	4
2	2	4	4	4
0	0	0	0	4
2	1	2	1	4
3	2	1	0	2
4	2	0	2	3
3	3	1	4	4

0	2	0	2	2
4	2	0	3	4
0	2	2	2	4
4	4	1	4	4
4	4	4	4	4
2	4	0	4	4
1	2	0	2	0
0	0	0	0	0
3	4	4	4	4

0	4	1	0	4
4	4	2	0	4
4	4	4	2	4
4	4	0	0	2
2	3	0	3	4
4	3	0	1	4
1	0	3	2	3
1	3	2	3	3
2	3	3	0	4

2	3	0	3	3
0	2	0	0	4
3	3	4	3	4
4	2	0	0	1
0	0	1	2	4
1	2	2	0	4
2	3	0	3	0
2	4	2	4	4
0	2	0	2	4

0	0	0	2	4
0	0	0	4	4
4	4	0	3	4
4	4	0	4	4
0	2	0	0	3
0	0	2	2	3
2	2	0	3	3
4	4	0	2	4
2	3	0	4	0

2	0	4	4	4
0	3	1	4	4
0	2	0	2	0
4	4	0	2	2
2	1	0	2	4
0	0	0	0	4
0	3	0	2	4
0	0	4	0	4
0	4	4	0	4

0	2	4	0	4
2	4	0	2	4
0	1	0	0	3
0	4	0	2	4
0	4	4	0	4
0	2	4	2	4
0	1	0	0	2
1	2	4	2	4
2	2	0	1	4

4	4	2	3	4
2	4	4	2	4
0	0	0	0	4
4	4	4	4	4
2	2	4	2	4
4	2	4	0	0
4	4	4	0	0
2	3	4	0	4
0	2	4	0	4

2	2	0	0	4
2	2	0	0	4
0	2	0	0	0
2	3	0	2	4
0	2	4	0	4
4	3	0	3	3
4	2	0	2	2
3	2	3	2	4
2	2	4	2	2

0	0	0	0	0
2	4	2	4	3

@17.Mividasexual mellena	@18.Mesientosolo	@19.Discutofrecue ntemente	@20.Mesientoqueri doyquemenecesita n	@21.Disfrutomitie mpolibre
4	3	0	1	4
1	1	3	0	3
0	4	2	0	2
2	2	1	0	1
4	4	0	0	0
4	4	0	3	4
4	4	0	0	2
4	4	1	1	4

0	4	4	0	4
0	3	1	1	0
4	3	2	2	1
4	2	1	0	2
4	3	2	1	3
1	2	3	0	0
4	4	0	1	2
4	3	3	3	2
4	4	3	3	2

2	4	3	2	2
4	4	4	4	4
1	2	0	0	1
4	4	4	4	4
3	2	2	0	0
4	4	0	4	2
4	2	2	2	3
0	0	0	0	0
0	4	0	0	1

3	3	2	2	2
2	2	3	1	3
4	0	0	1	4
4	4	0	0	4
4	4	1	1	2
0	2	1	0	1
0	1	1	0	3
4	4	4	3	3
2	3	2	2	2

1	2	0	0	2
4	4	2	2	2
4	4	2	0	0
2	2	4	0	2
0	2	1	0	0
0	0	3	0	1
0	1	0	1	1
0	1	1	1	0
2	2	0	1	1

0	4	3	0	3
3	1	0	0	2
4	3	2	4	4
4	4	0	1	2
2	3	3	2	3
4	4	3	2	4
3	4	2	4	4
0	3	3	2	1
4	0	4	0	2

3	4	3	2	4
4	4	0	1	3
2	2	0	2	0
0	0	1	0	0
4	1	1	2	4
4	2	1	0	3
2	2	4	1	2
4	4	0	0	1
3	4	4	4	4

4	4	1	2	0
4	4	0	0	2
4	4	2	1	3
2	4	2	3	0
4	2	0	0	0
4	4	0	1	4
0	0	4	0	0
0	4	4	4	3
4	0	2	0	1

4	4	0	2	2
4	2	0	0	2
0	4	4	2	2
4	0	2	0	2
4	4	3	2	4
4	4	4	4	4
4	4	4	0	2
4	4	0	0	2
4	4	3	2	0

4	3	3	3	2
4	2	2	2	4
4	4	0	4	3
4	0	0	0	0
0	4	0	0	0
3	4	1	0	2
4	4	2	1	1
2	4	2	4	3
4	4	0	2	3

0	0	3	2	1
4	4	0	3	4
4	4	2	1	1
4	2	2	0	2
1	3	4	3	4
3	4	1	0	0
4	0	0	2	4
3	0	0	0	2
4	4	2	0	4

0	4	1	0	0
4	4	0	2	0
1	2	2	0	2
0	2	1	3	1
0	4	4	2	2
1	4	4	4	4
2	4	2	0	0
0	2	0	2	4
4	4	4	4	4

0	1	0	0	0
0	3	0	0	0
0	4	0	0	4
0	0	0	0	0
0	3	1	0	2
4	3	3	3	3
3	2	0	0	4
3	3	2	2	3
2	4	0	3	3

4	2	3	0	4
2	1	0	0	3
4	4	1	4	4
3	3	3	0	4
4	2	0	1	0
4	4	2	3	1
3	4	1	4	4
4	4	0	1	2
3	2	0	0	3

3	4	2	0	4
4	4	0	1	4
4	4	4	4	4
3	4	4	4	4
1	0	0	0	0
4	4	2	2	0
4	4	0	0	2
2	1	1	0	0
0	0	2	0	0

0	2	1	0	2
3	4	4	1	0
2	2	2	2	0
4	2	4	0	0
2	2	1	0	2
3	3	2	0	0
0	0	1	0	0
0	4	0	2	1
4	0	2	0	3

4	3	0	0	3
1	4	2	0	1
4	3	0	2	4
0	3	0	1	1
0	0	0	0	0
3	3	1	1	3
4	4	2	1	3
1	4	4	2	2
4	4	4	4	4

4	4	4	2	2
3	4	0	2	2
4	4	4	0	4
0	2	2	1	4
4	2	0	0	0
0	1	2	0	2
2	4	0	0	0
0	0	1	0	2
4	4	4	2	3

4	4	2	2	2
4	3	2	2	0
4	2	0	3	2
4	3	3	3	3
2	2	2	0	4
4	3	2	1	0
4	4	0	0	0
4	4	4	2	4
4	3	2	2	3

2	0	3	0	0
0	2	2	0	2
2	2	2	0	4
4	4	1	2	2
4	0	0	0	1
0	0	3	0	0
0	2	1	1	0
0	4	3	2	2
4	4	3	4	4

4	4	2	4	4
4	3	4	1	0
4	4	0	3	1
3	4	2	2	2
3	3	1	1	1
1	0	3	2	1
0	4	0	0	0
2	4	0	4	4
0	2	1	0	4

4	4	0	2	2
4	4	0	0	0
2	2	2	0	0
4	4	3	4	4
3	4	1	3	3
1	2	2	0	0
2	4	0	2	0
4	4	0	0	4
0	2	4	2	2

4	4	4	3	4
4	4	2	2	4
0	4	4	4	4
4	2	2	0	2
2	4	0	0	1
2	4	2	2	4
3	4	1	0	1
0	4	0	0	4
4	2	0	0	4

0	0	0	0	0
2	4	4	0	2
4	3	3	1	4
4	2	0	0	1
4	4	4	2	1
4	4	0	0	1
4	4	0	3	3
1	4	2	3	3
4	3	0	2	2

4	3	0	0	0
4	2	0	1	2
0	0	0	0	0
4	3	3	0	0
2	4	2	4	1
4	2	0	0	0
4	0	1	0	0
4	4	4	3	4
4	4	0	1	4

1	2	0	1	3
3	4	3	3	2
2	2	4	0	2
0	4	3	4	1
0	3	3	1	3
4	2	2	3	1
1	4	1	3	3
0	4	4	2	3
0	3	3	0	0

1	3	2	1	2
0	2	2	2	1
3	4	4	3	3
2	4	3	2	3
2	0	3	0	3
1	3	2	3	3
0	4	4	0	2
4	4	2	0	2
0	3	0	2	4

3	4	2	3	4
1	0	2	0	3
4	4	0	3	1
4	4	3	1	3
4	2	1	4	4
0	2	2	1	4
0	0	4	0	4
1	3	3	2	3
1	2	2	0	1

0	0	1	0	4
0	1	1	0	2
0	0	0	0	1
0	4	4	2	0
0	4	2	1	4
2	2	1	2	4
4	2	4	0	2
0	0	0	0	0
0	4	0	0	0

0	4	4	0	0
4	4	4	4	4
4	4	0	4	4
2	2	3	2	4
4	0	4	4	1
1	4	3	2	0
0	0	1	0	1
0	4	0	0	0
0	3	2	3	3

4	4	0	1	2
3	4	2	3	4
0	2	0	0	0
4	2	2	0	0
4	2	0	0	0
4	4	1	3	4
4	1	1	3	0
4	3	3	4	4
3	1	2	0	1

4	4	0	0	1
4	2	4	2	0
4	0	0	0	0
0	4	0	2	0
0	0	4	2	0
0	0	0	0	0
4	4	0	0	4
4	4	0	4	4
0	0	0	2	0

4	4	2	4	4
4	4	0	0	4
4	4	2	3	4
2	3	2	2	2
1	3	2	1	1
1	2	3	0	0
2	4	4	3	4
4	4	0	0	2
4	4	2	0	4

2	3	1	2	1
0	2	2	0	2
0	2	2	0	1
0	4	1	2	2
4	2	4	2	2
0	2	1	2	0
3	4	2	0	2
2	3	2	2	3
2	2	3	0	2

4	4	3	4	4
0	2	2	2	0
1	4	2	2	2
0	3	0	0	0
2	2	0	0	0
0	2	3	0	3
3	2	2	2	1
4	0	0	0	0
0	3	2	0	1

0	0	0	0	0
4	4	0	2	3
0	4	0	0	3
3	2	3	0	3
4	4	0	3	2
0	4	1	1	0
4	4	4	4	2
0	2	0	2	2
4	4	0	3	3

4	4	1	0	0
4	0	2	0	3
4	4	0	0	0
1	4	3	0	2
3	2	3	4	2
3	2	2	2	1
4	4	4	0	2
4	4	2	4	3
2	4	2	2	2

4	4	1	2	4
2	2	1	0	2
4	4	0	0	0
0	0	0	0	0
4	4	4	4	4
4	4	2	3	2
3	4	4	2	4
4	4	2	1	1
4	2	1	1	0

3	4	4	0	3
2	3	0	0	0
4	2	3	0	3
0	4	0	2	0
2	4	4	0	0
4	4	0	0	0
4	0	4	4	4
0	0	2	2	0
4	0	0	0	2

4	2	0	0	0
4	2	0	0	0
4	4	1	2	2
4	0	0	0	4
2	0	0	1	1
4	3	0	1	1
0	0	0	0	0
4	4	1	4	0
2	4	2	2	2

4	4	0	2	2
4	0	0	0	0
3	2	2	0	2
4	4	0	4	0
4	0	0	0	0
2	2	4	2	0
4	2	0	0	2
4	4	2	4	2
4	4	4	3	3

4	3	0	1	1
2	4	4	3	2
4	2	2	0	2
4	4	0	4	4
4	2	3	4	3
0	4	4	4	4
0	4	4	2	2
0	0	0	0	0
4	4	0	4	4

4	4	0	2	0
4	4	4	4	4
2	4	4	4	1
2	4	0	4	2
4	4	0	2	4
3	3	3	2	3
2	3	1	1	1
0	3	2	2	2
4	4	4	3	4

2	3	0	2	0
4	2	1	0	4
1	4	3	4	4
4	4	2	2	2
3	4	0	0	3
0	2	0	1	1
3	3	0	4	4
4	4	0	3	2
1	2	0	4	4

0	4	0	0	1
0	0	0	0	3
4	4	0	4	4
4	4	0	3	3
2	2	2	0	1
0	2	0	0	0
4	4	2	2	4
4	4	0	3	4
4	4	0	0	2

4	4	0	0	0
3	4	1	0	4
0	0	1	0	0
2	4	4	4	4
0	2	4	2	2
0	2	0	0	0
0	2	0	2	2
0	4	0	0	0
0	4	0	4	4

0	4	2	4	2
4	4	4	0	0
0	2	0	0	0
4	4	3	1	4
4	4	1	0	0
2	4	2	2	0
4	4	4	0	0
2	4	3	2	4
2	2	3	2	1

4	4	0	4	4
4	4	0	1	4
0	0	0	0	0
4	4	0	2	4
4	2	4	4	2
0	2	0	1	4
0	0	3	0	2
4	4	4	2	4
0	0	0	0	1

4	4	0	4	2
4	4	4	0	0
0	4	0	0	0
0	0	2	2	1
0	3	4	1	4
4	4	1	2	4
4	2	2	2	4
3	4	2	2	4
1	2	0	1	1

0	0	0	0	0
4	4	3	3	3

@22.Tengodificultadesparaconcentrarme	@23.Mesientosinesperanzaenelfuturo	@24.Estoycontentoconmigomismo	@25.Meperturbanomolestanpensamientosdelosquenomepuedo	@26.Memolestaquemecritiquenporquetomoomedrogo.Nose
1	1	3	2	0
3	3	3	2	1
2	1	1	1	2
3	3	3	1	3
2	2	2	3	4
2	2	4	3	0
4	0	2	4	0
2	3	2	1	0

0	0	4	4	2
4	1	3	4	0
3	2	2	3	0
4	1	3	3	0
2	2	2	2	0
2	0	3	1	0
1	1	0	2	0
1	1	2	2	0
2	4	4	2	0

1	2	4	2	0
4	4	3	4	0
4	0	0	2	0
3	3	3	4	4
2	4	4	2	0
4	0	2	2	0
4	1	3	2	0
0	4	0	0	0
4	3	2	0	0

3	2	3	3	0
4	2	2	4	0
3	2	2	4	0
4	0	4	4	0
3	1	1	3	0
4	1	2	4	0
0	1	0	4	0
4	4	4	4	0
2	2	2	2	0

0	2	0	3	0
2	4	0	2	0
4	1	2	4	0
1	1	2	2	0
0	2	0	0	0
1	4	1	3	3
0	2	1	3	0
4	1	2	2	0
1	2	2	3	0

3	2	3	4	0
2	3	2	4	0
3	3	4	3	0
3	3	2	4	0
2	2	3	3	0
3	3	3	4	0
4	4	0	4	0
3	0	1	3	0
0	2	1	4	0

2	3	3	3	0
4	4	4	2	0
0	4	0	1	0
2	0	3	3	0
4	3	3	2	0
4	2	2	2	0
2	0	1	3	0
3	3	0	2	0
2	4	4	4	0

4	2	0	0	0
2	3	0	2	0
3	1	2	2	0
3	0	2	2	0
4	4	0	3	0
4	4	3	3	0
2	4	4	3	0
4	0	4	4	0
4	0	2	0	0

2	2	2	2	0
3	2	2	4	0
4	0	2	2	0
0	0	0	0	0
2	0	4	4	0
4	4	4	4	0
4	4	4	4	0
4	0	2	0	0
1	2	2	4	0

3	3	1	4	0
3	3	2	2	0
3	2	3	4	3
3	3	3	0	0
3	2	4	4	0
4	2	2	3	0
1	2	1	0	0
4	0	3	4	0
4	0	3	4	0

3	1	2	1	0
4	3	2	4	0
2	2	3	3	0
1	1	2	2	0
4	3	4	4	0
4	2	0	0	0
2	4	3	4	0
2	2	0	4	0
2	4	4	4	0

4	4	2	2	0
4	4	4	4	0
2	0	2	2	0
3	2	2	2	0
4	4	2	0	0
4	4	4	4	0
0	2	0	0	0
0	2	4	2	0
4	4	4	4	0

4	0	0	4	0
3	0	0	1	0
4	3	0	0	0
4	3	3	4	2
0	3	3	2	2
1	4	3	4	0
0	4	0	3	0
4	3	3	3	0
4	3	2	4	0

3	1	3	1	0
2	2	1	2	0
3	1	4	4	0
4	4	3	4	0
2	0	1	2	0
4	2	2	0	0
4	1	4	4	0
0	3	0	4	0
3	0	0	0	0

4	4	4	4	0
4	2	2	3	0
2	4	4	4	0
4	2	4	4	0
3	0	0	4	0
4	2	2	4	0
2	1	2	3	0
3	2	2	3	0
2	0	0	0	0

0	4	0	2	0
1	4	2	3	0
2	1	3	1	0
2	2	4	4	0
4	3	2	3	0
4	2	2	4	0
2	0	0	1	0
0	1	3	1	0
2	0	1	4	0

0	3	2	4	0
3	1	1	4	0
4	1	3	4	0
1	3	1	4	0
4	3	0	2	0
2	1	3	2	0
1	3	4	4	0
1	1	3	4	0
2	4	4	4	0

4	0	0	3	0
4	2	2	2	0
0	0	0	4	0
3	2	3	0	0
4	2	0	4	0
4	1	2	0	0
2	0	2	4	0
2	4	0	0	0
4	2	4	4	0

4	4	4	4	0
4	3	2	3	0
3	3	3	4	0
3	3	3	3	2
4	3	2	3	0
2	0	0	2	0
4	0	0	4	0
2	2	4	2	0
2	2	3	2	0

2	4	0	0	0
0	4	0	2	0
2	0	3	4	0
4	4	4	4	0
4	0	0	0	0
4	4	2	0	0
4	4	0	4	0
4	4	2	2	0
2	2	2	3	0

3	4	4	3	2
4	2	2	4	0
3	3	1	2	0
3	2	2	2	0
1	3	1	1	0
2	2	2	3	0
0	3	2	3	0
4	1	3	4	0
0	0	4	2	0

0	4	2	3	0
3	4	1	4	0
4	0	0	0	0
4	0	0	4	0
2	0	2	2	0
2	2	0	4	0
4	0	0	4	0
4	2	4	4	0
4	2	3	2	0

4	4	4	2	0
0	1	2	4	0
4	2	4	2	0
3	0	2	4	0
2	2	3	4	0
2	0	2	0	0
2	0	1	0	0
0	0	4	4	0
0	4	0	0	4

2	0	0	2	0
3	4	0	0	0
4	2	2	0	0
0	2	0	0	0
4	2	2	4	0
4	1	2	4	0
4	4	4	2	0
3	4	2	4	0
4	2	2	4	0

4	0	2	0	0
0	0	0	2	0
0	0	0	0	0
2	4	1	4	3
0	4	3	3	0
0	0	0	3	0
4	0	0	4	0
3	4	4	4	0
3	3	4	4	0

4	2	4	4	0
0	1	3	4	0
4	3	2	4	0
3	4	0	3	0
2	3	2	3	0
4	1	3	2	0
0	3	3	0	2
4	4	4	4	4
3	3	0	3	0

4	2	3	3	0
1	0	3	4	1
0	2	2	0	0
3	2	3	1	0
3	2	2	2	0
3	1	2	4	0
0	2	3	4	0
2	4	4	4	0
2	1	2	3	0

3	3	3	3	0
4	0	1	2	0
4	4	4	4	0
1	1	3	3	0
3	0	3	4	0
0	2	2	1	0
4	0	0	1	0
4	2	3	4	0
2	2	0	3	0

4	2	3	2	2
2	0	2	4	0
3	0	0	0	0
2	2	0	2	0
0	2	2	4	0
2	1	2	4	0
4	4	3	3	0
4	2	2	2	0
4	0	2	0	0

0	4	3	4	0
4	2	4	4	0
4	4	4	4	0
4	4	4	4	0
0	4	0	4	0
0	0	2	2	0
0	2	2	2	0
4	4	4	4	0
3	3	3	1	0

4	4	2	3	0
4	1	3	3	0
0	4	0	0	0
2	2	0	3	0
4	0	3	4	0
3	1	3	3	0
2	3	1	0	0
4	4	3	2	0
2	2	2	3	0

4	4	3	4	0
4	1	4	2	0
0	2	0	0	0
4	4	0	0	0
4	0	2	0	0
2	0	0	4	0
4	4	4	0	0
4	4	4	0	0
0	0	0	0	0

4	4	1	4	0
3	4	4	3	2
4	2	2	2	0
4	1	2	3	0
1	2	2	3	2
3	0	2	3	0
4	4	4	4	0
4	4	2	2	0
2	2	3	0	0

2	0	1	2	0
3	4	2	1	0
2	1	3	4	0
4	4	3	4	0
0	1	2	2	0
2	2	2	0	0
2	1	0	3	0
3	1	2	3	0
2	0	2	4	0

2	2	4	2	0
2	2	2	2	0
2	3	1	2	0
1	2	0	3	0
2	1	2	0	0
3	2	3	4	0
2	4	0	2	0
2	0	1	2	0
0	0	2	1	0

3	2	2	3	0
4	2	2	4	0
3	0	0	2	0
2	0	0	4	0
2	2	2	2	0
2	4	4	4	0
4	4	4	4	0
2	0	0	2	0
4	4	4	4	0

4	2	4	4	0
3	0	2	2	0
2	0	2	2	0
2	0	2	2	0
2	2	4	2	0
4	3	2	1	0
0	0	4	4	0
3	3	4	2	0
2	1	2	3	0

3	2	3	4	0
2	1	0	3	0
4	2	2	4	0
0	0	0	0	0
2	4	4	2	0
2	4	3	2	0
2	4	4	2	0
3	4	4	4	0
4	4	2	2	0

4	0	2	4	0
3	1	2	3	0
1	0	2	2	0
4	2	4	4	0
0	4	4	4	0
4	4	4	4	0
4	0	2	4	0
0	4	2	4	0
0	4	2	4	0

2	0	0	2	0
3	4	0	0	0
4	1	2	4	0
4	4	4	4	0
1	3	1	0	0
0	1	0	0	0
0	0	0	0	0
4	4	2	4	0
2	2	2	2	0

4	2	4	4	0
3	0	0	4	0
2	1	0	4	0
4	0	2	0	0
0	0	0	4	0
1	2	0	4	0
3	2	2	0	0
2	2	2	4	0
4	2	3	2	1

3	3	2	3	2
2	2	4	2	0
4	1	2	4	0
0	4	3	4	0
4	2	4	4	0
4	0	4	4	0
0	0	2	2	0
0	4	0	0	0
4	4	4	4	0

4	0	4	4	0
4	0	4	0	0
4	4	4	4	0
4	2	4	4	0
1	2	4	4	0
3	3	3	3	0
1	3	1	3	0
2	3	3	3	0
2	3	3	3	0

1	3	2	3	0
0	0	0	0	0
3	4	4	4	0
3	1	1	0	2
2	2	2	4	0
3	1	1	4	0
4	3	4	3	0
4	4	4	4	0
4	2	2	4	0

0	2	2	4	0
2	4	0	2	0
0	4	4	3	0
4	1	4	3	0
2	4	3	3	0
2	2	2	2	0
4	4	4	2	0
2	4	3	3	0
4	0	0	4	0

4	4	3	2	0
4	4	4	4	0
0	4	4	2	0
2	4	4	0	0
2	0	2	0	0
0	4	0	4	0
0	4	4	2	0
0	4	0	2	0
0	0	4	4	0

2	0	2	4	0
4	2	3	4	0
0	0	0	0	0
4	4	4	0	0
4	4	2	0	0
4	0	0	4	0
4	2	1	0	0
2	2	2	4	0
2	2	3	3	0

4	4	4	0	0
4	2	4	4	0
0	4	0	0	0
4	0	4	4	0
4	0	2	2	0
4	2	2	4	0
4	4	4	4	0
0	2	1	4	0
0	2	2	2	0

0	2	0	0	0
0	4	2	4	0
0	0	0	4	0
0	0	4	4	0
4	4	4	4	0
4	1	2	4	0
2	1	4	2	0
4	2	4	4	0
3	3	1	1	0

0	0	0	0	0
3	4	3	3	0

@27.Tengomalestaresestomacales	@28.Trabajoestudiotanbiencomolohacíaantes	@29.Micorazónpalpitademasiado	@30.Tengodificultadesparallevarmebienconmisamigosycono	@31.Estoysatisfechoconmivida
2	2	2	0	3
4	3	0	0	2
2	1	4	0	0
0	3	2	0	2
2	3	3	0	2
0	1	0	3	3
0	1	2	0	2
4	3	3	1	2

0	1	2	0	1
0	1	4	2	3
0	1	2	3	2
2	2	1	1	4
2	2	1	1	2
2	4	2	1	1
2	3	2	0	1
0	0	4	0	2
2	2	2	0	2

2	4	3	2	2
4	3	2	1	3
2	0	4	0	0
1	2	2	0	4
0	2	1	2	2
1	3	3	4	4
1	2	1	0	3
0	0	0	0	4
0	3	2	0	1

4	1	2	2	2
3	3	3	0	3
2	2	2	0	1
3	3	2	1	2
3	3	3	2	0
3	0	3	1	3
2	3	3	0	0
1	0	2	1	3
2	3	2	1	3

3	1	4	0	0
0	2	2	0	2
0	0	0	1	2
2	2	1	1	0
2	2	0	0	2
2	0	2	2	1
3	3	3	0	1
0	3	2	1	1
3	4	3	1	3

3	4	0	1	4
3	3	1	3	3
2	4	3	3	4
4	3	0	0	2
3	2	1	1	2
3	1	3	3	4
0	3	3	2	4
2	3	2	0	2
2	4	3	0	4

2	2	1	1	3
4	4	4	0	4
2	1	2	0	0
4	0	0	0	0
4	3	2	1	3
1	4	0	0	0
0	1	0	1	3
3	0	2	0	0
4	0	4	1	4

0	2	2	0	0
0	3	0	0	0
0	3	2	0	2
0	0	0	0	2
2	1	0	0	0
4	4	4	0	4
1	0	0	0	4
4	0	4	4	4
4	2	0	0	2

3	2	2	4	2
4	3	2	4	0
0	0	2	4	2
2	3	2	2	2
2	3	2	2	4
4	0	4	0	4
2	0	4	0	4
0	4	2	0	2
4	0	4	0	2

0	3	4	0	4
3	1	0	0	2
4	0	3	0	4
1	1	3	0	2
0	4	2	0	2
4	3	3	0	2
0	0	3	0	1
4	3	2	1	3
3	4	3	0	3

0	3	3	0	2
2	2	3	0	4
3	2	0	0	0
2	0	4	4	2
2	2	3	2	4
2	3	1	1	2
4	4	4	0	4
2	3	2	0	0
2	4	3	0	4

2	0	1	2	4
4	0	4	0	4
3	2	2	0	1
0	1	2	1	0
4	0	4	0	2
4	4	4	2	4
2	0	2	0	0
0	2	4	0	2
0	4	2	4	4

0	0	2	0	0
0	0	2	0	0
2	4	0	0	2
3	4	2	0	0
0	1	2	2	3
0	4	2	0	2
0	0	2	0	4
4	1	2	3	3
0	4	2	0	2

4	3	3	0	2
0	2	2	1	0
2	4	2	0	4
4	1	4	0	4
4	1	2	0	1
0	2	2	0	2
2	3	3	0	4
0	0	2	0	0
2	2	2	0	0

0	4	0	0	4
4	4	4	0	0
3	4	0	4	4
0	3	3	2	4
2	0	4	0	0
2	0	3	2	2
2	0	0	0	2
3	0	2	0	0
1	0	2	0	0

1	1	0	0	2
0	1	0	0	2
0	3	2	1	2
4	2	2	0	4
4	3	4	2	2
3	2	2	1	2
2	2	0	0	0
0	4	0	0	2
0	0	0	0	1

3	2	1	0	3
0	1	0	0	2
3	3	2	0	3
0	1	0	0	1
2	0	0	0	0
3	2	3	1	2
0	2	3	2	3
4	3	2	0	4
4	1	4	0	4

4	1	1	0	2
0	2	2	0	2
4	3	2	0	0
4	4	0	0	2
4	4	4	0	1
3	0	1	0	1
1	1	2	0	2
0	2	2	0	0
4	2	2	4	3

4	2	4	0	4
3	4	0	3	2
2	2	1	0	4
0	3	0	1	2
2	3	2	0	2
2	0	2	1	0
4	2	3	0	0
2	2	0	0	4
0	2	2	0	3

2	2	4	0	0
2	0	0	0	0
2	2	4	2	3
4	0	3	0	4
0	0	4	0	0
4	0	4	0	2
2	0	3	0	0
2	2	2	0	2
2	2	2	0	3

3	1	4	1	4
0	4	0	2	1
0	4	0	0	2
3	1	4	0	2
3	4	3	0	2
0	2	3	0	2
0	0	2	0	2
3	4	3	1	4
0	2	2	0	2

3	2	2	2	2
4	1	3	0	0
4	0	4	0	0
4	4	4	0	0
2	2	2	0	0
4	0	0	0	0
4	3	4	0	0
4	4	4	0	4
3	3	2	2	3

4	4	4	0	4
0	4	3	0	2
0	2	0	0	4
4	2	2	0	0
0	0	2	4	3
2	4	2	0	0
2	0	0	0	0
0	0	0	0	0
0	0	0	0	0

2	0	2	0	0
2	2	0	0	1
2	4	0	3	2
0	0	0	0	0
4	3	3	2	4
4	0	3	0	0
4	3	2	3	4
2	4	2	0	4
2	0	0	0	2

0	3	0	0	2
1	0	2	0	0
0	0	0	0	0
4	3	3	4	0
0	0	2	0	4
2	3	0	0	0
2	1	3	0	1
3	4	4	0	4
4	0	0	3	2

4	3	0	0	1
0	3	0	0	3
4	3	3	3	2
2	4	2	0	3
3	2	2	0	2
4	3	3	0	2
4	0	2	0	3
2	3	4	3	3
3	3	3	0	0

2	0	4	1	2
1	1	1	0	1
2	3	0	4	1
0	3	2	2	2
4	3	2	1	2
3	2	3	2	3
0	2	2	0	2
0	0	4	0	2
3	3	4	0	2

3	3	0	0	2
2	1	2	1	1
3	3	3	1	3
3	3	2	0	2
0	4	0	0	3
4	2	2	2	2
4	0	2	0	0
2	3	2	4	3
2	2	1	0	3

4	4	0	0	2
4	4	3	0	0
0	2	0	0	0
4	2	2	0	0
0	0	4	0	3
0	2	1	0	2
0	0	4	2	2
4	3	2	0	0
4	4	4	0	4

0	1	3	0	0
0	2	2	1	2
4	4	4	0	4
4	4	4	4	1
0	0	2	0	0
2	0	2	0	2
4	4	4	0	2
0	4	4	0	0
3	3	3	1	3

3	4	3	0	2
0	4	3	2	3
2	4	2	0	4
2	4	0	0	0
3	4	2	0	0
0	2	3	1	3
0	0	0	0	1
0	1	4	0	3
0	1	3	0	1

2	4	4	0	2
4	4	4	0	2
4	2	4	0	0
4	4	4	0	0
0	2	0	0	2
4	3	4	0	2
3	2	3	0	4
4	4	4	0	4
0	0	0	0	0

4	4	4	4	2
4	4	2	0	4
2	4	2	2	2
3	4	3	3	1
2	2	3	2	3
0	0	4	0	0
4	4	2	2	4
4	3	0	0	3
2	4	2	0	2

3	0	2	1	1
2	0	2	2	3
0	3	2	1	2
2	3	4	0	2
0	0	4	0	2
4	1	2	0	3
1	0	2	0	2
2	3	1	0	2
4	2	2	0	1

3	2	2	0	4
0	2	2	0	1
0	0	2	0	2
2	0	3	0	0
3	3	2	0	1
3	3	2	0	2
2	3	3	1	1
2	2	2	0	0
3	2	0	0	2

3	0	3	0	2
0	0	4	2	2
2	2	2	0	2
2	3	2	0	0
0	1	0	0	2
4	3	4	0	3
0	4	4	2	4
2	2	2	0	2
0	3	3	0	4

0	0	2	0	3
3	2	0	0	0
0	1	3	2	2
0	0	0	0	4
2	0	2	0	2
0	2	2	0	2
0	2	3	0	4
3	3	2	2	4
3	2	0	0	2

2	4	3	0	3
3	3	2	0	0
2	0	1	0	2
0	0	0	0	0
3	4	4	0	4
4	3	4	1	4
0	2	2	0	2
4	4	2	2	4
2	1	1	2	1

4	4	2	0	2
0	3	0	0	2
0	0	2	0	0
4	0	2	0	2
4	4	4	0	4
4	4	4	0	4
4	0	2	0	3
4	2	0	0	3
2	3	4	0	0

0	2	4	4	0
2	0	2	0	0
2	0	2	0	0
4	4	2	0	2
2	2	3	0	1
0	1	0	0	1
0	4	0	0	0
2	4	2	2	4
4	4	0	2	2

4	4	2	0	4
0	2	2	0	0
4	4	2	0	2
4	4	0	0	1
0	2	2	0	0
0	0	0	0	0
2	0	0	4	2
4	4	2	0	2
0	3	0	4	3

2	2	3	2	2
4	4	1	0	3
4	0	2	0	2
4	0	3	0	4
4	4	4	0	4
4	4	3	4	4
0	0	4	0	1
2	0	0	0	0
2	0	4	0	4

2	1	0	2	4
4	3	2	0	4
4	4	4	2	4
2	2	0	0	3
2	4	0	0	2
2	3	2	3	1
3	1	3	0	2
1	1	2	1	2
4	2	2	3	3

3	1	0	3	3
0	0	0	0	0
3	3	3	3	2
0	4	2	2	2
3	3	4	0	2
1	3	2	1	0
3	4	2	0	3
4	4	4	2	4
0	0	0	2	2

0	0	0	0	0
0	4	2	0	2
0	0	0	0	4
1	4	4	4	3
2	2	2	2	2
2	4	2	0	2
0	4	1	1	4
1	4	2	2	4
0	4	4	0	0

2	2	2	0	2
2	3	4	1	2
0	4	3	0	2
4	4	0	2	4
4	4	2	0	2
4	4	2	4	0
0	4	0	0	4
0	0	2	4	0
4	0	4	0	4

4	3	4	0	2
0	4	0	4	0
0	0	0	0	0
4	1	4	0	4
0	3	0	0	0
4	0	4	0	4
4	0	2	0	0
2	2	4	2	2
3	4	3	1	2

2	4	4	0	4
4	0	2	0	4
0	0	2	0	0
4	0	4	0	4
4	3	3	0	2
4	4	4	0	2
4	2	4	0	4
4	2	2	0	2
4	3	0	0	2

0	0	0	0	2
4	4	4	4	2
0	2	2	0	2
2	2	1	3	3
4	3	4	0	4
1	4	2	1	3
0	3	0	0	2
2	3	3	0	2
1	1	0	0	1

0	0	0	0	0
4	2	3	3	2

@32.Tengoproblemaseneltrabajoescueladebidoalasdrogaso	@33.Sientoquealgomalovaaocurrir	@34.Tengolosmúsculosadoloridos	@35.Meatemorizanlosespaciosabiertoselmanejarelestard	@36.Mesientonervioso
3	3	3	0	2
0	2	3	0	2
0	2	2	0	2
2	2	3	4	3
0	2	4	0	2
0	2	0	0	1
0	2	2	0	4
0	2	3	2	4

0	4	4	0	4
0	3	1	0	2
0	3	3	0	3
0	2	3	4	4
0	2	2	0	2
0	1	1	0	3
0	2	4	4	4
0	1	3	4	4
0	0	3	0	4

0	0	2	0	4
0	2	3	1	4
0	0	2	4	3
0	4	4	4	4
0	2	4	2	2
0	4	4	0	2
0	2	2	0	3
0	0	0	0	2
0	0	4	0	0

0	3	3	2	2
0	2	2	2	3
0	0	4	0	4
0	2	4	0	4
1	2	2	0	3
0	0	0	0	3
0	1	4	0	2
0	2	2	2	3
0	0	0	0	4

0	1	4	0	4
0	2	3	0	4
0	0	4	0	4
0	1	3	2	4
0	2	1	0	2
0	0	3	0	3
0	4	0	0	3
0	1	2	3	4
0	3	4	0	3

0	0	2	2	4
0	4	1	0	3
0	3	4	0	3
0	3	0	2	4
0	3	2	0	3
0	0	3	0	3
0	0	2	0	2
0	2	2	2	3
0	2	4	3	3

0	3	2	2	2
0	2	2	2	2
0	4	2	0	2
0	2	0	4	2
0	3	1	2	1
0	2	2	1	2
0	4	2	4	3
0	0	3	0	2
0	4	0	4	4

0	4	0	0	4
0	0	2	4	2
0	3	2	3	3
0	4	2	0	3
0	0	3	0	3
0	2	4	0	4
0	0	1	0	2
0	3	3	0	4
0	0	2	1	2

0	1	4	0	2
0	2	3	2	4
0	4	0	0	2
0	0	4	0	2
0	0	4	0	4
0	4	4	4	4
0	4	4	0	4
0	0	2	0	0
0	2	4	4	4

0	0	0	0	2
0	2	3	0	3
0	2	3	0	3
0	0	0	0	3
0	3	3	3	3
0	3	3	3	3
0	0	0	0	3
0	3	3	0	3
0	4	4	3	3

0	1	3	0	3
0	3	0	4	4
0	2	3	0	4
0	2	4	0	4
0	3	1	0	3
0	0	0	4	3
0	4	4	0	2
0	2	2	0	4
0	4	2	4	4

0	4	0	0	4
0	4	2	0	4
0	0	2	4	2
0	2	3	2	2
0	0	4	4	4
0	4	4	4	4
0	2	4	4	4
0	2	2	2	4
0	4	4	4	4

0	0	1	2	0
0	0	4	0	0
0	0	4	0	0
0	4	3	3	4
1	2	2	1	2
0	3	3	3	2
0	2	0	1	2
0	4	2	4	4
0	2	3	2	2

0	3	3	0	4
0	1	2	3	3
0	4	2	4	0
0	0	4	2	4
0	0	4	3	2
0	4	0	0	2
0	3	1	0	3
0	4	3	3	4
0	2	2	1	2

0	4	3	0	4
0	0	4	4	4
0	4	0	4	4
0	4	4	0	3
0	3	3	3	2
0	4	2	4	4
0	0	4	2	4
0	3	3	0	4
0	0	2	2	1

0	3	3	3	3
0	1	3	3	1
0	2	4	0	2
0	2	3	0	4
0	3	3	2	4
0	2	3	1	3
0	2	2	2	1
0	0	3	2	3
0	3	3	1	2

0	2	3	0	4
0	0	3	0	4
0	2	0	0	3
0	0	3	0	3
0	3	0	0	2
0	2	2	0	2
0	0	3	0	3
0	3	2	1	4
0	2	4	4	4

0	0	4	0	4
0	4	4	0	3
0	0	2	0	0
0	0	4	0	0
0	4	4	0	2
0	4	4	4	4
0	4	4	0	2
0	2	2	0	3
0	4	4	4	4

0	4	2	2	4
0	1	0	0	3
0	0	3	0	2
0	3	2	2	2
0	2	1	0	1
0	0	0	0	2
0	2	3	0	2
0	2	3	2	4
0	2	2	2	2

0	2	0	4	4
0	0	0	0	2
0	4	0	0	4
0	4	2	2	2
0	0	0	0	4
0	0	4	2	3
0	4	0	0	2
0	0	4	0	4
0	2	2	4	2

3	3	3	0	4
0	4	2	0	2
0	0	3	0	0
0	4	4	2	2
0	1	2	0	2
0	0	2	0	0
0	2	0	0	2
0	3	3	2	3
0	0	0	4	2

0	3	0	0	3
0	4	3	4	3
0	1	4	1	4
0	0	4	0	4
0	2	3	0	2
0	4	4	0	4
0	4	4	0	4
0	4	4	0	4
0	4	4	1	2

0	4	4	4	4
0	2	3	0	4
0	2	2	2	4
0	4	0	0	2
0	4	2	0	4
0	0	0	0	2
0	0	3	0	0
0	4	4	4	2
0	0	4	4	3

0	2	0	0	2
0	0	0	3	3
0	0	2	0	3
0	0	0	0	2
0	3	3	3	4
0	4	4	0	4
0	3	4	0	4
0	4	0	0	4
0	3	2	2	4

0	0	0	4	2
0	0	2	0	1
0	0	0	0	0
1	4	4	0	4
0	0	2	0	0
0	2	4	0	3
0	0	3	0	3
0	4	2	0	0
0	2	4	4	4

0	2	4	2	4
0	0	2	0	2
0	4	4	3	4
0	0	1	0	3
0	3	2	0	3
0	2	4	2	4
0	3	3	4	4
2	4	2	3	4
0	4	3	0	3

0	3	4	1	4
0	3	0	0	3
0	0	2	2	1
0	3	3	1	4
0	0	3	4	4
0	0	3	1	4
0	3	3	0	2
0	2	2	0	2
0	3	3	4	4

0	2	3	0	2
0	0	4	0	2
0	0	4	0	4
0	4	3	4	4
0	0	0	0	3
0	2	4	2	3
0	0	2	0	3
0	2	0	0	3
0	1	3	2	2

0	0	0	0	2
0	1	1	0	2
0	0	4	4	0
0	2	4	2	4
0	3	0	4	3
0	2	2	0	2
0	2	0	0	4
0	4	4	2	4
0	0	4	0	0

0	0	0	0	2
0	2	4	0	4
0	2	4	0	0
0	4	4	4	4
0	4	0	0	4
0	2	2	0	3
0	2	4	0	4
0	4	4	0	4
0	2	3	3	3

2	0	0	3	4
0	3	2	1	0
0	3	0	0	3
0	2	3	0	0
0	3	3	1	3
0	3	3	0	3
0	2	3	0	3
0	2	3	4	4
0	0	3	0	4

0	4	4	0	4
0	3	1	0	4
0	0	4	0	4
0	0	4	0	0
0	0	4	0	2
0	4	4	4	4
0	3	4	0	4
0	3	4	4	4
0	0	0	0	0

0	4	0	0	3
0	2	2	4	4
0	2	0	2	2
0	4	4	3	4
0	0	1	0	3
0	0	4	0	4
0	4	2	3	4
0	3	2	0	3
0	2	2	0	2

0	2	1	1	1
0	3	2	2	4
0	4	4	2	3
0	4	4	4	4
0	4	0	2	2
0	4	0	0	4
0	1	4	3	1
0	4	3	2	3
0	2	4	2	4

0	2	0	0	3
0	4	2	2	3
0	3	3	0	2
0	3	3	1	3
0	2	3	2	2
0	4	4	2	4
0	4	2	0	4
0	0	4	0	2
0	1	2	2	2

0	3	4	2	4
0	3	3	0	4
0	4	4	2	4
0	0	2	0	3
0	2	0	0	0
0	3	4	3	4
0	4	4	4	4
0	0	2	0	2
0	3	4	0	4

0	0	3	0	3
0	2	2	0	4
0	0	4	0	3
0	1	0	0	2
0	2	2	0	2
0	4	0	0	3
0	4	2	0	4
0	2	4	0	3
0	2	3	1	3

0	2	4	0	4
0	0	2	0	2
0	2	0	0	4
0	0	0	0	0
0	4	2	2	4
0	4	2	4	4
0	2	4	2	2
0	2	4	2	4
0	4	2	2	4

0	4	4	3	4
0	2	0	0	2
0	2	2	0	0
0	0	4	4	4
3	2	4	0	4
0	4	0	2	4
0	4	4	0	0
0	4	4	4	4
0	3	4	2	4

0	2	4	0	4
0	2	4	0	4
0	2	2	0	4
0	4	4	0	2
0	0	0	0	0
0	0	0	0	0
0	0	0	0	0
0	2	2	4	1
0	0	4	0	4

0	2	0	4	4
0	4	4	0	0
0	2	4	0	2
0	0	4	0	4
0	2	4	0	4
0	4	4	0	0
0	0	3	2	2
0	4	4	4	4
0	1	0	0	2

4	3	3	4	3
0	0	4	2	2
0	2	4	2	4
0	2	2	4	2
0	0	4	0	4
0	4	4	0	4
0	0	2	0	4
0	0	0	0	0
0	4	4	4	4

0	4	0	0	4
0	4	4	0	0
0	4	0	0	2
0	0	0	0	0
0	2	4	0	4
0	4	3	2	3
0	0	4	0	3
0	2	4	4	4
0	0	3	0	4

0	3	3	2	3
0	4	2	0	0
0	4	4	3	4
2	1	4	0	2
0	4	2	2	4
0	0	2	0	4
0	1	0	3	3
0	4	4	4	4
0	2	2	4	0

0	0	0	0	0
0	0	4	0	0
0	3	0	0	0
0	4	3	0	2
0	2	2	0	2
0	0	0	0	4
0	0	4	0	3
0	4	2	0	4
0	4	2	4	4

0	2	0	2	4
0	4	0	4	4
0	3	4	4	4
0	0	4	0	4
0	2	2	4	4
0	2	2	0	2
0	0	2	2	0
0	4	2	4	4
0	4	4	0	4

0	4	4	0	4
0	4	4	0	4
0	0	0	0	0
0	4	4	0	4
0	4	4	0	4
0	4	4	0	4
0	3	0	0	2
0	4	2	4	2
0	2	3	4	4

0	4	4	0	4
0	2	4	4	4
0	0	0	0	1
0	2	4	2	4
0	0	2	4	4
0	0	0	0	4
0	4	0	2	4
0	0	3	0	0
0	0	4	0	2

0	0	2	0	3
0	0	0	0	4
0	0	0	0	0
0	2	4	3	3
0	4	4	4	4
0	3	4	0	3
0	0	0	0	0
0	4	0	4	4
0	4	1	0	2

0	0	0	0	0
0	3	4	4	4

@37.Mesatisfacenmisrelacionesconmisseresqueridos	@38.Sientoquemevabieneneltrabajoescuela	@39.Tengomuchasdiscusioneseneltrabajoescuela	@40.Sientoquealgoandamalconmimente	@41.Tengodificultadesparadormironomepuedoquedardormid
3	3	0	3	4
1	1	0	2	4
1	1	0	0	2
0	3	0	1	4
0	1	0	3	4
3	1	0	2	4
4	1	0	2	3
1	0	0	2	4

3	1	3	0	0
2	0	1	4	4
2	3	2	2	4
0	2	1	0	2
1	1	1	1	3
0	2	3	1	1
0	2	0	2	4
2	0	0	3	1
2	2	0	2	2

0	1	1	2	4
4	4	2	4	0
0	0	0	2	0
1	1	0	3	4
0	2	0	0	4
2	3	0	3	4
2	0	0	3	3
0	0	0	0	2
1	1	0	0	4

1	1	0	3	4
1	2	0	4	4
1	1	0	1	2
3	3	0	0	4
2	4	0	2	4
0	0	0	3	0
3	0	0	0	3
3	0	0	4	4
0	2	2	2	4

0	0	1	3	1
0	0	2	0	4
0	0	1	3	4
0	2	2	1	2
0	0	1	0	2
0	0	1	1	2
1	2	0	4	4
1	1	1	2	1
0	3	1	3	4

3	4	2	4	4
3	2	2	4	2
3	4	2	4	4
1	3	0	3	4
2	2	3	4	4
2	3	0	3	4
4	3	0	4	4
2	1	3	3	4
0	3	3	3	4

2	2	2	3	4
2	4	0	0	4
0	0	0	2	0
0	2	0	0	0
3	3	3	3	4
0	4	0	4	0
0	1	0	0	4
0	0	0	0	0
2	2	0	4	4

1	1	4	2	0
1	0	1	1	1
1	0	2	3	4
0	3	0	0	0
0	0	0	4	0
1	0	0	4	4
0	0	2	2	4
0	0	0	4	0
0	1	2	3	4

2	2	0	3	4
0	0	0	2	4
3	1	0	2	2
2	2	2	0	4
3	3	3	0	4
2	4	4	4	4
0	2	2	4	4
1	1	0	4	2
1	0	0	3	4

1	0	0	4	3
1	2	0	3	4
3	0	0	4	0
3	4	0	0	3
0	4	0	4	4
0	1	0	3	3
1	1	0	0	4
3	4	0	4	4
1	3	0	3	4

0	3	2	1	3
0	0	0	4	4
1	2	2	3	4
2	2	0	4	4
2	2	1	4	3
0	0	0	4	2
0	2	0	4	0
0	0	0	2	4
0	4	0	3	4

0	3	1	4	4
4	4	0	4	4
0	2	2	2	4
0	0	0	2	2
4	0	2	4	4
0	2	2	4	4
0	0	0	0	0
0	0	0	2	0
4	4	0	4	4

0	0	0	2	0
0	0	2	2	2
0	0	0	4	0
0	4	0	3	4
3	0	0	2	3
2	3	0	2	4
0	1	0	0	3
0	2	0	4	4
0	4	0	3	4

1	3	3	3	1
0	1	0	0	4
3	4	0	4	4
0	2	1	4	4
2	1	0	2	4
2	2	0	2	4
0	2	0	4	4
0	0	0	2	2
0	0	0	1	3

0	3	0	4	1
4	4	0	4	4
0	3	0	4	2
4	4	2	2	4
0	1	0	2	4
2	0	2	4	4
1	0	0	2	3
0	4	4	4	4
0	0	0	0	2

1	1	1	0	3
0	1	3	3	2
2	2	0	4	4
1	0	0	4	4
2	3	3	4	4
0	1	2	2	3
0	1	0	2	0
1	4	0	3	4
0	1	1	3	3

0	2	1	3	4
0	2	2	3	4
2	3	1	4	4
1	0	1	3	4
0	0	0	2	3
2	2	2	3	1
2	2	3	4	0
0	3	4	2	4
0	2	0	4	4

0	2	3	4	4
0	0	0	3	0
0	0	4	0	4
0	4	0	2	0
0	0	2	4	4
0	0	0	2	4
0	4	0	2	4
0	0	0	1	3
3	3	1	4	4

2	4	1	4	1
1	4	0	2	1
2	2	0	3	0
3	2	0	3	0
2	2	0	2	1
0	0	3	2	0
0	0	0	2	2
2	0	0	2	4
2	2	3	2	3

0	1	2	0	4
0	0	0	2	0
2	3	0	1	4
2	0	0	3	4
0	0	0	4	3
2	0	0	2	2
0	4	2	4	4
2	2	2	0	4
2	3	3	1	4

3	1	0	3	4
0	4	0	4	2
2	4	0	0	0
2	2	0	2	2
1	0	0	1	2
2	2	0	2	0
0	4	0	2	0
2	4	0	4	4
0	4	4	4	2

1	1	0	3	4
0	1	0	2	2
0	0	0	1	4
0	0	0	4	4
0	0	0	2	2
0	0	1	4	4
0	0	0	4	0
0	4	0	4	4
2	4	4	4	4

4	4	2	4	4
0	0	0	2	4
2	0	0	2	4
2	2	2	3	3
0	1	2	4	4
0	2	2	0	3
0	0	0	2	2
2	2	0	2	4
0	0	0	0	0

0	0	0	2	0
0	0	0	0	0
2	4	3	3	0
0	0	0	0	2
4	0	0	3	3
0	3	0	2	4
2	4	0	2	4
1	4	0	4	3
0	0	0	2	2

0	0	0	0	4
1	1	0	0	2
0	0	0	0	0
0	1	0	3	4
1	1	0	2	4
0	0	0	0	4
0	2	0	4	1
3	4	0	4	2
2	0	0	3	4

0	2	0	1	4
3	2	0	2	4
1	2	3	4	4
1	4	0	3	3
3	2	3	3	3
0	3	3	2	2
0	2	0	2	4
3	2	0	4	4
0	0	2	3	4

1	0	0	4	1
1	0	1	2	2
2	0	1	2	4
1	0	3	2	3
2	3	1	4	4
2	2	2	3	3
1	1	2	3	4
2	0	0	4	4
1	1	0	3	4

2	2	2	4	4
1	1	2	2	4
2	4	0	3	4
2	3	1	3	4
3	4	4	4	4
0	2	3	2	3
0	0	1	2	2
3	2	1	3	3
0	1	2	3	2

2	4	0	2	0
0	0	0	2	0
0	0	0	0	3
0	2	2	2	2
3	0	0	0	0
0	2	1	2	2
0	2	1	4	4
0	0	0	4	4
0	4	0	0	4

0	0	4	4	0
0	2	4	2	4
4	4	0	2	4
0	3	4	4	2
3	0	4	2	4
0	2	2	0	4
1	1	2	0	4
0	0	0	4	4
3	4	4	2	4

1	4	0	4	3
3	4	0	3	3
0	4	0	2	3
0	2	0	0	1
0	1	0	2	3
2	3	0	3	3
3	4	0	0	2
3	2	0	3	3
0	1	0	2	4

1	1	0	4	4
0	3	1	1	4
0	0	0	2	4
2	0	0	2	4
2	0	0	0	2
0	0	0	2	2
0	4	3	4	4
2	0	0	4	4
0	0	0	0	0

4	4	4	4	4
1	4	0	2	0
0	4	0	4	4
1	2	2	3	3
1	2	1	3	1
0	0	2	2	4
2	4	0	4	3
0	1	0	2	3
1	2	1	4	3

2	0	0	2	3
2	3	2	4	2
2	2	3	3	3
2	2	0	3	4
0	0	0	2	4
0	0	1	4	4
2	0	2	0	4
1	3	2	2	4
2	0	4	4	4

3	2	3	2	2
0	0	4	2	2
1	0	0	0	3
0	0	0	3	2
0	0	0	2	1
0	0	0	3	4
1	1	0	2	4
0	0	0	0	3
0	0	0	2	1

0	0	0	3	4
3	0	0	4	4
0	0	0	2	4
0	0	3	0	2
0	1	0	2	3
0	3	1	4	4
4	2	2	4	4
2	0	0	0	2
3	3	0	4	4

0	2	0	4	4
0	0	0	0	2
1	1	0	2	2
2	2	2	2	2
2	1	0	2	2
0	2	0	3	4
3	1	2	3	4
4	4	0	2	4
0	2	0	2	4

2	2	0	2	4
0	2	0	3	4
0	0	0	4	4
0	0	0	0	0
2	2	0	2	4
2	2	0	2	4
2	2	2	3	4
3	4	0	4	4
0	2	2	3	3

2	1	0	4	4
0	3	0	2	3
0	0	0	2	0
2	0	0	0	4
0	2	0	4	4
0	4	0	4	4
2	0	4	4	4
2	4	2	4	4
4	4	1	4	4

0	0	4	4	4
0	0	0	2	4
2	4	0	4	4
2	4	0	4	4
0	4	0	0	2
0	0	0	0	2
0	0	0	0	0
2	2	0	4	4
2	4	4	4	4

0	4	0	4	4
0	0	0	2	4
0	3	0	4	4
1	4	0	4	4
0	0	0	0	4
0	0	0	0	4
0	4	0	0	4
0	4	0	4	4
2	3	4	4	4

0	0	0	4	3
2	4	0	3	3
0	2	0	2	4
2	2	0	4	4
2	4	3	2	4
2	4	4	4	1
1	2	4	2	4
0	0	0	0	0
1	3	0	4	4

4	0	4	4	4
4	4	4	4	4
2	4	4	4	2
2	4	0	4	4
4	2	0	4	4
3	4	0	3	4
2	2	1	2	0
2	2	2	4	3
3	2	1	3	4

2	3	0	4	4
0	0	0	0	4
3	3	3	3	4
3	4	0	2	4
0	0	0	4	4
0	1	0	2	4
4	4	0	3	4
3	4	0	4	4
2	2	0	4	4

0	0	0	0	3
0	4	0	2	3
0	0	0	4	4
2	4	3	4	3
2	0	2	3	2
2	2	0	2	0
2	3	2	1	4
2	4	0	2	4
0	0	0	4	4

2	2	2	2	4
2	2	2	2	4
0	2	0	0	2
4	4	2	4	4
2	2	0	2	2
0	0	0	0	4
2	4	0	4	3
0	0	0	4	4
4	0	0	4	4

0	2	4	0	4
0	4	0	4	4
0	0	4	0	0
0	0	0	4	4
4	0	0	4	4
0	0	0	4	4
0	0	0	4	4
0	2	2	2	4
1	2	1	3	3

4	4	0	4	4
0	0	0	4	4
0	0	0	0	2
0	2	0	4	4
1	1	4	2	4
0	4	4	4	4
0	4	3	4	4
2	0	0	0	4
3	0	0	0	4

0	0	0	3	0
0	0	0	4	0
0	0	0	3	4
0	0	0	2	4
0	4	0	3	4
2	4	0	3	4
0	3	2	2	0
1	0	0	2	2
0	1	0	3	3

0	0	0	0	0
1	2	1	3	4

@42.Mesientotriste	@43.Misrelacionesconotrosmesatisfacen	@44.Meenojotantoeneltrabajoescuelaquepuedohaceralgod	@45.Medandoloresdecabeza	FACTORDESENCADENANTEDELEPISODIOACTUAL
3	2	0	2	12
4	2	0	2	10
4	1	0	2	1
4	0	2	4	3
3	1	3	3	2
4	3	0	0	3
4	2	0	2	11
4	0	0	4	4

4	3	2	3	10
3	2	0	3	6
4	2	1	3	14
2	1	1	2	11
3	2	0	3	10
3	0	0	0	6
4	0	0	2	0
4	2	0	4	11
4	1	0	4	11

4	2	4	4	3
4	3	0	4	1
2	0	0	2	8
4	2	0	4	1
2	0	2	2	11
4	2	0	2	5
4	2	0	4	8
2	0	0	0	11
4	0	0	2	13

4	2	0	4	12
4	2	0	4	14
2	3	0	2	11
4	3	0	3	0
4	2	0	4	2
4	2	0	2	3
2	0	0	2	1
4	3	1	3	0
3	2	0	4	8

3	0	0	4	11
2	2	2	4	1
4	2	0	4	2
2	2	2	3	10
2	0	0	2	11
2	1	2	1	8
2	0	0	3	6
2	1	1	1	2
3	2	1	3	1

4	3	0	3	2
3	1	1	0	4
4	3	2	3	13
4	2	0	1	12
4	2	2	3	13
3	2	0	3	2
2	3	4	3	8
3	1	3	3	2
4	0	3	4	10

4	2	0	2	1
4	2	0	4	10
1	0	1	4	10
0	0	0	0	2
4	2	0	4	11
4	0	0	2	0
2	1	0	0	6
4	0	0	2	10
4	3	1	2	10

3	0	0	0	11
1	0	0	3	8
4	2	0	2	5
2	0	0	0	2
3	0	0	3	6
4	1	4	4	1
2	0	4	1	10
4	4	4	4	13
0	0	0	3	10

4	2	0	4	11
2	0	3	4	1
2	0	0	3	8
4	2	2	0	3
4	2	3	0	10
4	4	4	4	11
4	2	4	4	14
4	0	0	2	2
4	0	0	3	11

4	2	0	3	0
4	1	2	0	2
4	3	0	3	1
2	0	0	2	1
4	0	0	4	10
2	1	0	4	13
3	1	0	0	8
4	3	0	3	10
4	2	0	4	12

4	0	2	4	12
4	1	0	2	5
4	2	0	3	10
4	2	0	0	10
4	3	0	4	8
4	3	2	2	11
4	0	0	2	11
2	0	0	3	2
4	0	2	2	12

4	0	0	0	10
4	4	0	4	7
2	4	0	4	1
2	0	2	2	6
4	0	2	4	8
4	0	0	4	8
0	0	2	3	8
4	4	0	2	8
4	4	0	4	10

2	0	0	0	8
2	0	2	2	2
4	2	2	4	11
4	1	0	4	2
2	3	0	0	1
4	2	0	0	2
3	0	0	0	2
4	2	0	2	1
4	0	0	2	8

4	3	3	4	0
2	0	0	2	10
4	3	0	0	1
4	0	0	3	1
2	1	0	1	2
4	0	0	2	2
4	3	0	2	4
4	0	0	2	12
4	0	0	0	10

4	2	0	2	2
4	3	0	4	1
4	1	0	2	10
4	2	0	3	10
3	0	0	3	3
4	2	2	4	2
3	2	0	3	14
4	0	2	4	8
2	0	0	3	12

3	1	0	2	8
4	1	0	3	8
4	0	0	4	3
4	2	0	4	11
4	3	3	4	1
4	0	2	4	2
0	2	0	2	2
4	0	0	2	11
3	2	0	2	8

4	1	2	3	2
4	1	2	3	8
4	2	0	0	1
3	1	0	3	8
2	1	3	4	6
3	1	3	4	9
4	2	1	4	3
4	2	4	2	11
4	0	4	4	10

2	1	4	4	2
2	0	0	3	11
4	0	4	4	1
3	0	0	0	5
4	0	2	4	0
4	0	0	1	8
4	0	0	2	4
2	0	1	0	4
4	2	2	4	14

4	2	4	0	2
3	1	3	3	11
3	2	0	0	8
3	2	0	0	6
2	1	3	2	5
2	2	0	3	4
4	0	0	4	8
4	3	0	4	3
4	2	2	3	8

0	0	2	4	3
0	0	0	0	11
3	3	2	2	14
4	2	0	4	1
2	0	2	4	11
3	0	2	1	1
4	0	3	2	8
4	0	2	4	11
4	2	0	1	8

4	3	0	4	12
2	0	0	0	10
4	0	0	0	5
4	2	4	0	6
1	1	0	2	2
3	2	0	1	12
3	1	0	0	14
4	2	0	3	1
4	2	2	4	1

4	1	0	4	1
4	1	0	3	7
4	0	0	4	10
4	0	0	4	2
2	0	1	2	8
4	0	0	4	2
4	0	0	4	1
4	0	2	4	1
4	2	4	4	10

4	1	4	4	1
4	0	0	2	1
4	0	0	4	6
4	2	4	3	11
3	0	0	4	1
4	0	0	4	2
2	0	0	4	1
2	2	4	2	8
4	0	0	4	10

2	1	0	2	2
3	4	0	2	5
3	2	2	1	11
0	0	0	0	9
4	2	4	0	7
3	0	0	1	12
4	2	0	0	1
4	2	0	4	11
3	0	0	2	3

2	0	0	4	10
2	0	0	0	5
0	0	0	0	3
4	0	0	3	1
4	1	0	0	5
4	0	0	3	3
2	0	0	4	1
4	0	0	1	2
4	4	0	4	10

4	1	0	0	2
4	2	0	4	1
3	2	0	4	2
3	2	0	0	11
3	2	3	3	1
3	1	2	4	9
4	0	2	0	8
4	3	0	4	8
3	0	0	4	0

3	0	0	2	1
3	1	0	0	10
4	2	2	3	2
4	2	4	2	10
2	2	1	3	11
4	2	3	4	11
3	2	1	0	11
3	1	0	0	12
3	2	1	4	11

2	2	0	3	1
2	1	0	3	11
4	3	3	4	3
4	2	3	4	0
4	3	1	0	1
3	2	1	4	1
1	3	1	2	1
3	3	0	0	10
2	1	0	2	6

2	2	0	0	8
1	0	2	2	2
0	0	0	0	1
4	0	2	2	0
4	3	0	0	2
2	0	0	4	12
4	0	0	4	5
2	0	1	4	1
4	0	0	2	2

4	0	4	2	5
4	3	3	2	2
4	2	0	4	9
4	2	4	4	8
2	2	3	2	1
2	1	0	2	3
4	1	1	4	0
4	0	0	4	12
4	3	2	4	4

4	1	0	3	0
4	3	0	4	12
1	0	0	0	7
2	0	0	0	8
3	0	0	3	1
4	2	0	3	4
2	3	0	1	14
4	3	2	1	8
3	1	0	3	0

4	0	0	4	2
4	3	3	4	2
4	0	0	4	9
0	0	0	4	0
2	0	0	4	10
4	0	4	4	5
4	0	0	4	12
4	3	0	4	6
0	0	0	1	8

4	4	0	4	1
3	0	0	4	11
4	2	0	2	3
3	2	2	4	12
3	2	2	3	4
2	0	2	0	14
4	2	2	3	4
3	0	0	0	1
3	0	0	1	11

1	2	0	2	10
2	2	3	3	1
4	2	0	3	5
4	3	2	4	0
4	2	0	0	0
4	0	0	1	8
4	2	1	4	8
4	2	2	4	5
2	2	2	4	2

3	4	2	3	5
3	3	0	4	2
4	0	0	3	1
3	0	0	2	9
3	0	0	2	1
4	2	3	4	8
4	1	2	1	10
2	0	0	2	0
2	1	0	1	11

4	0	0	4	1
4	2	0	4	8
4	0	0	4	1
4	0	2	1	9
2	1	0	0	4
4	1	4	4	7
4	2	2	4	5
2	0	0	2	2
4	3	0	4	8

4	2	0	4	1
2	1	0	2	11
4	4	0	2	12
3	2	0	0	12
3	0	0	4	10
4	0	0	3	3
4	4	0	2	10
4	2	0	4	5
4	0	2	2	2

4	2	0	2	0
4	1	0	2	0
4	0	0	4	2
0	0	0	0	0
4	2	0	4	5
4	3	0	4	2
4	0	0	3	11
4	2	0	4	8
4	2	1	2	11

4	2	0	2	12
3	0	0	4	5
2	0	0	0	8
4	3	0	2	12
4	0	0	0	2
4	4	0	4	8
4	4	2	0	11
4	2	4	4	1
4	4	0	4	10

2	0	0	4	1
4	0	2	0	6
4	2	0	2	10
4	2	0	4	8
1	0	0	0	11
3	0	0	0	4
0	0	0	2	11
4	4	4	4	14
2	2	4	2	1

4	4	4	4	1
4	0	0	0	2
4	0	0	4	3
4	2	0	4	2
2	0	0	3	5
2	0	4	4	10
2	1	0	2	6
4	0	0	2	11
4	3	1	0	0

4	0	0	0	4
4	2	0	3	0
2	2	0	2	11
4	3	4	4	2
4	0	3	4	0
0	4	4	4	10
4	2	0	2	8
0	0	0	2	1
4	2	0	4	11

4	2	0	4	10
4	4	4	4	10
4	4	4	4	2
2	2	0	2	8
4	4	0	2	4
4	3	3	3	0
2	2	0	3	5
4	2	1	3	4
3	1	3	3	5

3	2	2	3	11
4	0	0	0	6
4	3	4	4	8
2	2	0	2	10
4	3	0	2	11
2	0	0	3	0
2	2	0	2	1
4	4	2	4	2
4	2	4	0	6

0	0	0	0	11
0	0	0	1	5
4	0	4	1	3
4	3	4	4	2
2	2	2	0	13
0	0	0	2	5
4	3	0	3	1
4	3	0	2	8
4	0	0	4	8

4	2	2	4	2
2	2	3	4	12
2	0	0	0	2
4	0	2	4	14
3	2	0	3	10
2	0	0	4	3
3	2	0	0	7
4	0	4	2	2
4	0	0	0	10

4	0	2	4	11
4	3	4	0	10
2	0	0	0	10
4	0	0	4	10
4	0	0	0	1
4	0	2	4	12
4	0	0	1	1
4	2	2	2	11
3	2	1	2	1

4	4	0	4	10
4	2	0	3	2
0	0	0	3	10
4	2	0	2	10
3	2	4	1	8
4	4	2	4	6
4	0	4	4	1
4	2	0	4	10
2	1	0	2	8

4	0	0	0	12
4	2	4	4	2
4	0	0	0	0
4	4	4	4	9
2	0	0	4	7
4	2	0	3	8
2	1	2	2	2
4	3	0	0	6
3	0	0	0	10

0	0	0	0	5
4	0	1	3	9

FUMA	CUANTOFUMADIARIO	NúmerodepacienteonúmerodeFicha	@1.Insomnioalacostarse	@2.Insomniodemadrugadaentrelas1200ylas400a.m
0		1	2	2
0		2	2	2
0		3	0	1
0		4	2	2
0		5	2	2
0		6	2	2
0		7	2	1
0		8	2	2

0	9	0	0
0	10	2	1
0	11	2	1
0	12	1	0
1 10 cigarrillos al dia	13	2	1
0 0	14	0	0
0	15	2	2
0	16	0	0
0	17	2	1

0		18	2	1
0		19	0	0
0		20	0	0
0		21	2	2
0		22	2	2
0		23	0	0
0		24	0	1
0		25	1	1
0	nunca ha fumado	26	2	1

1 1 cigarao diario	27	2	2
1 nofuma	28	2	2
0	29	2	1
0	30	2	2
1 30 cigarros	31	2	0
0	32	0	0
0	33	2	2
1 3	34	0	1
1 4 diarios	35	2	1

1 5 cigarros	36	0	0
0	37	0	2
1 6 cigarros dia	38	2	0
0	39	2	1
1 1 semana	40	2	1
1 6	41	1	0
0	42	2	2
0	43	1	0
0 0	44	1	1

1	2-3 cigarillos diarios, variable, desde hace 1 mes	45	2	0
0	-	46	1	1
1	1 dia (ha bajado su consumo)	47	2	1
1	mm 5 cigarillos / dia	48	2	0
0	0	49	1	2
0	mediados de febrero de este año (llegó a fumar 1 cajetilla diaria, por años)	50	2	2
0		51	0	0
0		52	0	0
0		53	0	0

0	54	0	0
0	55	0	0
0	56	0	0
0	57	0	0
0	58	0	0
0	59	0	0
0	60	0	0
0	61	0	0
0	62	0	0

0		63	0	0
0		64	0	1
0	no aplica	65	2	2
0		66	0	0
0		67	0	0
0		68	1	2
1	20 CIGARRILLOS	69	0	0
0		70	0	1
0	No aplica	71	2	1

0	No aplica	72	2	2
0	No aplica	73	2	1
1	fumadora social	74	2	2
0	NO APLICA	75	2	2
1	7 AL DÍA, HACE UNOS MESES, ANTES UNA CAJETILLA DIARIA	76	2	2
0	NO APLICA	77	2	2
1	10 a 12 al día	78	2	2
0		79	0	0
0		80	2	2

0		81	2	0
0		82	2	0
1	3 CIGARRILLOS	83	0	0
0		84	0	1
0		85	2	0
0		86	2	2
1	1 cigarro promedio	87	2	0
1	promedio de 10 cigarros diarios	88	2	2
0		89	2	1

1 7 cigarrillos	90	2	2
0	91	2	2
1 1-6 cigarrillos	92	2	2
1 1 cigarrillo	93	2	2
1 6 cigarrillos	94	2	2
0	95	2	2
0	96	2	2
0	97	2	2
0	98	2	2

0	99	2	0
1 5 cigarrillos/dìa	100	2	2
0	101	2	1
0	102	1	1
0	103	2	2
1 10 cigarros	104	2	0
0	105	0	1
0	106	0	0
1 1 cajetilla	107	2	2

1 2 a la semana	108	0	0
1 5 o 6	109	0	1
0	110	0	0
0	111	2	2
1	112	0	1
0	113	2	2
0	114	2	1
0	115	2	2
1 5-6 diarios	116	2	2

0	117	1	0
1 ocasional	118	2	2
1 fumaba hasta antes del terrremoto ahora uno o dos	119	2	2
0	120	2	2
0 no	121	2	2
0	122	0	1
0	123	2	2
0 no fuma	124	1	1
1 ocasional	125	1	2

1 1 cajetila diaria	126	0	0
1 1 cajetilla sdiaria	127	2	2
0	128	0	0
1 tres a cuatro diarios	129	2	2
0	130	2	1
1 6 COGARROS DIA	131	2	1
1 4 por dia	132	2	0
1 20 dia	133	2	1
0	134	2	0

1	10 cigarros diarios	135	2	2
1	20 dia	136	2	2
0		137	2	0
1	10 dia	138	2	2
1	10 dia	139	2	2
1	2 dia	140	2	1
1	4 cigarillos en promedio	141	1	0
1	7 cigarrillos promedio	142	2	1
0	NC	143	2	1

0	nc	144	2	1
0		145	2	2
1	3-5 al dia	146	2	2
1	promedio 5 cigarrillos al dia	147	0	1
0	0	148	1	0
0		149	1	1
0		150	0	0
0		151	1	0
1	1 cajetilla	152	2	0

0	153	2	1
0	154	2	0
1 15	155	2	0
1 3	156	0	0
0	157	2	0
0	158	2	1
0	159	2	0
0	160	2	0
0	161	2	2

0		162	2	2
0		163	2	0
0		164	0	2
1	15-20 cigarrillos diarios	165	2	2
1	3-4 cigarrillos/ dìa	166	2	2
1	3-4 cigarrillos/dìa	167	2	2
0		168	2	2
1	5 cigarrillos/dìa	169	2	2
1	4 cigarrillos/dìa	170	2	2

0	171	2	2
0	172	0	0
0	173	2	2
0	174	2	2
0	175	0	0
0	176	1	1
0	177	2	2
1 fumadora social	178	2	2
0	179	2	2

1	10 cigarrillos promedio	181	2	2
0		183	2	1
0		184	0	1
1	20 cigarrillos	185	2	0
0		186	0	1
1	CIGARRO DE SEMANA	187	1	1
1	1 CAJETILLA	188	0	0
0		189	2	2
1	2 CIGARRILLOS	190	0	2

1	5 CIGARRILLOS DIARIOS	191	2	0
0		192	2	0
1	10	193	2	0
1	DE VEZ EN CUANDO 3 VECES AL MES	194	2	2
0		195	0	2
0		196	2	0
0		197	1	0
1	5	198	2	0
1	2	199	2	1

0	200	2	0
0	201	2	2
1 1 cajetilla diaria	202	2	0
0	203	2	1
0	204	2	1
0	205	2	0
1 2 CIGARROS AL DIA	206	0	0
0	207	2	2
0 1 cajetilla	208	0	0

0		209	0	0
0		210	0	1
0		211	0	0
0		212	0	0
1	2 cigrrsodiarion fin de semnas 20	213	0	2
0		214	2	2
0		215	2	2
0		216	2	2
0	1 cigarro	217	0	1

1 ocasional	218	2	0
1	219	0	1
0	220	2	2
1 1cajetilla	221	2	2
0	222	2	2
0	223	2	2
0	224	1	1
1 1cajdiaria	225	2	2
0	226	2	2

0	Fumaba 15cigarros antes del infarto	227	2	2
1	Ocasional	228	2	2
0		229	2	0
1	Ocasional	230	1	2
1	1 al mes	231	2	1
0		232	1	1
1	30 dia	233	2	1
1	20 dia	234	2	2
0		235	2	1

1	10 dia	236	0	1
1	1 dia	237	1	1
0		238	2	1
1	2 dia	239	1	1
0		240	1	0
1	3 cigarrillos al dia, ocasionalmente	241	2	2
0	nc	242	2	1
0		243	2	0
0		244	2	0

0		245	2	2
0		246	1	1
1	10 cigarrillos	247	2	2
0		248	2	1
0	no fuma desde que quedó embarazada	249	2	0
0		250	1	2
0		251	2	2
1	1 cigarrillo cada 6 meses	252	2	2
1	1-3 cigarrillos/ dìa	253	2	2

1 7 cigarrillos/día	254	2	0
0	255	2	2
1 6-8 cigarrillos/dìa	256	2	2
0	257	2	2
1 1 a 2 cigarrillos/dìa	258	0	0
0	259	2	2
1 2-3 cigarrillos/ dìa	260	2	2
0	261	2	2
1 ocasionalmente	262	2	2

0		263	0	0
1	2 cajetillas al día	264	2	2
1	Es relativo, 3 a 4 a 0 en el d{ia	265	2	2
0		266	1	1
1		267	2	2
1	2 cigarros al día	268	2	2
0		269	2	2
0		270	2	2
0		271	2	2

0	272	2	2
0	273	2	1
0	274	0	1
0	275	0	0
0	276	2	1
1 1 CIGARRO	277	2	2
0 2 diarios	278	0	1
1 1 CAJETILLA	279	0	0
1 2 CIGARROS	280	2	2

0	281	1	2
0	282	2	1
0	283	2	0
0	284	2	2
0	285	0	1
0	286	2	2
1 5	287	2	2
0	288	2	2
1 8	289	0	0

0	290	2	0
1 25 cigarrillos/dia	291	2	2
0	292	1	2
0	293	2	2
0	294	2	2
0	295	2	0
0	296	1	1
0	297	0	0
0	298	2	2

1	2 cigarrillos en fiestas	299	2	2
1	20 cigarrillos/día	300	2	2
0		301	2	1
0		302	0	2
1	2 dia	303	2	0
0		304	2	0
1	1 dia	305	2	2
1	20	306	0	0
0		307	2	0

1 8	308	2	2
0	309	2	0
1 fin de semana 5 por dia	310	2	1
0	311	0	2
0	312	0	1
0	313	2	2
0	314	1	2
0	315	2	2
1 6	316	0	1

1	1 al dia ocasionalmente	317	1	2
1	10 dia	318	2	2
0		319	2	1
0		320	0	2
0		321	1	0
0		322	0	1
1	10 dia	323	1	2
0		324	0	2
0		325	1	2

1 4	326	0	2
1 3cigarros	331	2	0
0	332	2	1
1 ocasional	333	1	0
1 2 cigarros diarios	334	1	2
0	335	2	2
1 seis	336	2	2
0	337	2	2
1 2 diarios	338	1	2

0	339	1	2
1 dos cajetillas diarias	340	2	2
1 ocasional	341	2	0
0	342	2	2
1 cutrao a cinco	343	2	2
0	344	2	2
0	345	2	2
1 2 diarios	346	2	2
0	347	2	1

1	2diarios	348	0	2
1	3a1caj ahora no el embarazo	349	1	2
1	4 diarios	350	1	0
1		351	2	2
0		352	2	2
0		353	2	2
0		354	2	2
0		355	2	2
1	10 cigarrillos diarios	356	2	2

0		357	2	2
1		358	2	2
1	20/día	359	2	2
1	4/día	360	2	2
0		361	0	2
0		371	2	1
1	20 CIGARRILLOS DIARIO	372	2	0
0		373	2	1
0		374	2	0

1	20	375	2	0
0		376	2	1
0		377	2	1
0		378	2	2
1	4	379	2	1
1	2	380	2	1
0		381	2	2
1	5 cigarrillos al día hasta hace 5 meses atrás.	382	2	2
0		383	2	2

1	2 cigarrillos/día	384	2	2
0		385	2	2
0		386	2	0
0		387	2	2
0		391	2	2
0	No aplica	392	2	2
0	no aplica	393	2	2
1	3 cigarros diarios	394	1	0
0		395	2	2

1	4/ día	396	2	2
1	6/día	397	2	2
0		398	2	2
0		399	0	0
0		400	2	2
1	ocasional<br>+ o - 1 vez al mes 1<br>cigarrillo y menos	401	2	2
1	'+ o - 4 cigarrillos al<br>dia	402	0	0
0	nc	403	2	2
0	actualmente NO<br>fuma<br>Dejó cigarrillo hace<br>13 años	404	2	2

0	410	2	1
1 1 diario	411	2	2
0	412	2	2
0	413	2	2
0	414	2	2
0	415	2	1
1 ocasional	416	2	2
0	422	2	2
1 1	423	2	1

1 5 cigarros,,	424	2	2
1 5 cigarros	425	2	2
0	426	2	1
1 2 cigarros	427	2	1
0	428	0	1
0	429	0	0
0	430	2	2
1 10	431	2	2
1 1 cajetilla	432	2	2

0	433	2	2
1 3 cajetillas diarias	434	2	2
0	435	0	0
1 3 cigarros	436	2	2
0	437	1	1
1 3 cigarris	438	0	2
0	439	2	1
1 15 cigarros	440	2	0
1 10	461	2	2

1 15	462	2	2
1 3	463	2	2
0	464	0	0
0	465	2	2
0	466	2	2
1 4	467	2	0
1 1	468	2	0
1 12	469	2	2
0	470	2	1

0		471	2	2
1	es poco; hay días que no fuma	472	2	2
0		473	0	1
0		474	2	2
0		475	2	2
0		476	2	2
0		477	0	0
0		478	0	2
0		479	2	2

0	480	0	0
1 8	501	0	0
1 10	502	2	2
0	561	2	2
1 un cigarro	562	2	2
0	600	2	2
0	700	0	0
1 10 cigarros diarios	701	1	0
1 3 CIGARRILOS	0	0	0

0	0	0	0
0	0	0	0

@3.Insomniotardío despuésdelas400a.m	@4.Estadodeánimo tristeza	@5.Ansiedadpsíquicasesientetensoirritableaprensivot	@6.Apetito	@7.Pérdidadepeso durantelaúltimasemana
2	3	0	0	0
2	3	0	2	2
1	1	1	1	1
2	3	2	0	0
2	2	3	0	0
2	3	2	2	2
0	2	3	0	0
1	3	3	2	2

0	3	2	0	0
1	3	3	1	1
1	3	3	0	0
1	2	2	1	1
1	2	1	1	0
0	2	1	0	0
1	3	3	0	1
0	2	1	1	2
0	3	1	0	0

0	3	2	0	0
0	3	3	0	0
0	1	1	0	0
2	4	1	0	0
2	1	2	2	2
0	0	2	0	0
1	0	3	0	0
0	1	0	0	0
1	1	0	0	0

2	3	4	1	2
2	3	3	1	0
1	1	0	0	0
2	3	3	2	2
2	3	2	2	1
0	2	2	0	0
0	3	2	0	0
2	2	1	1	1
0	2	3	0	0

0	1	2	0	1
0	2	1	0	0
2	1	1	0	0
2	1	2	0	0
2	1	1	0	0
0	2	1	1	0
1	2	4	0	0
0	2	3	0	0
0	2	2	0	0

1	4	4	1	1
0	2	2	1	0
1	4	4	0	0
2	3	1	2	1
1	3	1	1	0
1	2	2	0	0
0	0	0	0	0
0	0	0	0	0
0	0	0	0	0

0	0	0	0	0
0	0	0	0	0
0	0	0	0	0
0	0	0	0	0
0	0	0	0	0
0	0	0	0	0
0	0	0	0	0
0	0	0	0	0
0	0	0	0	0

0	0	0	0	0
0	1	1	0	0
0	2	0	0	2
0	1	1	0	0
2	2	0	2	1
0	3	2	1	1
1	1	1	0	0
2	2	1	0	0
0	3	2	0	0

2	2	0	0	0
1	1	1	0	0
2	4	3	2	2
2	2	1	1	0
2	2	2	0	0
2	4	4	1	0
0	4	3	0	0
0	4	4	2	0
2	0	3	0	0

0	3	3	2	2
0	3	2	1	1
0	3	3	2	2
1	2	1	0	0
2	3	3	2	2
2	2	4	1	1
0	2	0	0	1
0	3	2	2	1
2	3	3	0	0

0	3	3	0	0
2	4	1	2	2
2	2	2	1	2
0	2	3	2	2
2	4	4	1	0
2	2	3	2	2
2	4	3	2	2
2	2	3	0	0
2	4	4	2	2

2	2	3	2	2
2	4	3	0	1
0	2	2	0	0
0	1	2	0	0
1	4	2	0	0
1	1	3	0	0
0	1	2	0	0
0	2	1	1	0
2	3	4	2	2

0	2	0	1	0
0	0	1	0	0
0	3	2	0	0
2	3	3	0	0
2	1	0	1	0
2	4	2	0	0
1	2	1	0	0
2	2	4	1	2
2	2	3	1	2

0	3	3	0	0
2	2	3	0	0
0	3	4	1	1
2	3	3	1	1
1	2	2	1	1
1	2	3	1	1
2	3	3	2	1
1	2	3	1	1
0	2	1	2	1

0	3	3	2	2
2	3	2	2	2
0	3	3	0	0
2	3	3	2	2
0	2	4	1	1
2	3	3	1	1
0	2	2	1	1
1	2	3	2	2
0	1	2	0	0

2	2	2	1	1
1	2	1	1	1
1	2	2	1	0
0	3	3	1	1
0	3	3	1	1
0	2	2	0	0
0	0	2	0	0
2	2	0	0	0
1	2	2	0	0

1	3	2	1	0
2	2	2	0	0
1	3	3	1	1
0	2	0	1	1
1	1	1	2	1
1	2	2	0	0
2	3	1	0	0
2	4	2	1	0
2	3	2	1	1

0	2	2	0	0
2	4	2	1	2
2	2	2	1	0
0	4	2	0	0
2	4	2	0	0
2	2	2	1	0
2	4	2	2	1
2	3	3	1	1
2	4	3	1	2

2	3	3	0	0
2	2	2	2	2
2	3	3	1	1
2	3	3	0	0
2	2	3	1	1
2	3	3	1	2
1	2	2	0	2
0	3	1	2	2
1	2	1	2	2

2	2	3	2	2
0	2	3	2	2
2	3	2	2	2
2	4	1	2	2
0	4	4	2	2
1	4	3	0	0
0	3	3	1	0
2	3	2	0	0
2	3	2	0	0

1	4	3	0	2
0	3	2	0	0
0	2	2	0	0
0	4	3	0	0
0	1	1	0	0
0	3	2	0	0
0	3	3	1	0
2	4	3	0	0
2	1	2	0	0

0	3	2	1	1
1	2	2	0	1
2	2	2	1	1
0	2	1	1	1
2	3	2	1	1
1	2	2	0	0
2	2	2	0	0
2	3	2	1	1
2	3	2	1	0

2	4	3	1	1
2	3	2	1	1
0	2	2	0	0
0	2	0	1	0
1	1	1	0	0
0	1	0	2	1
0	1	0	0	0
0	3	3	2	0
0	2	3	0	0

0	0	1	0	0
0	2	1	0	0
0	2	2	0	0
0	2	1	0	0
2	2	2	0	0
2	2	2	0	0
2	4	3	2	2
1	3	4	0	0
2	2	3	0	0

0	1	0	0	0
1	1	0	0	0
2	4	3	2	2
2	3	3	2	2
2	4	2	1	2
0	1	3	0	0
1	2	2	0	0
0	4	3	0	0
2	3	3	1	0

1	2	1	0	0
2	3	2	0	0
0	1	3	0	0
1	0	2	2	2
1	3	2	0	0
2	1	2	1	1
2	3	2	2	2
2	3	3	0	0
2	2	2	0	0

0	1	2	1	2
1	2	2	2	2
0	2	2	1	1
1	2	2	0	1
2	2	2	1	1
2	3	2	0	0
0	3	0	2	2
2	2	1	0	0
0	3	3	1	1

0	2	2	1	0
0	2	1	0	0
0	3	2	2	2
2	4	3	1	0
0	3	1	1	1
1	3	3	0	0
1	3	2	0	0
2	2	1	1	0
1	3	3	2	2

0	2	2	0	0
1	3	2	0	0
0	3	3	2	2
0	0	3	0	0
0	0	4	1	1
2	3	3	2	2
2	2	2	2	2
2	1	0	0	0
2	3	1	1	0

0	1	2	0	0
2	4	4	0	2
2	4	3	2	2
1	3	4	1	1
2	1	1	2	2
2	3	1	0	0
2	4	4	0	0
2	4	4	2	2
2	3	3	0	0

2	3	3	1	1
1	4	3	0	0
0	0	3	0	0
0	2	3	0	0
0	3	2	0	0
2	3	2	1	1
0	2	2	0	0
0	3	3	0	1
2	2	2	0	0

2	4	2	1	1
2	2	2	0	0
1	2	1	1	1
2	1	0	1	1
0	2	1	0	0
2	3	3	2	1
2	0	2	1	1
0	3	0	1	1
0	0	0	0	0

0	4	3	2	2
2	3	2	0	0
2	2	3	2	2
2	3	2	0	0
2	3	3	0	0
2	3	2	0	0
0	3	3	1	2
0	2	2	1	2
2	3	1	1	0

1	1	2	2	2
0	3	3	2	2
0	2	2	0	0
1	3	3	1	1
0	2	2	1	0
1	2	2	0	0
0	2	1	2	2
2	3	3	2	2
0	2	2	2	2

0	3	3	0	0
0	2	2	0	0
0	3	2	1	1
0	1	2	2	2
0	2	2	2	1
0	2	3	2	1
1	1	2	1	1
0	1	1	1	0
0	1	2	1	1

1	3	3	0	0
2	2	2	0	0
1	2	2	1	1
2	2	2	0	0
2	2	1	0	0
0	3	2	2	1
2	3	3	1	0
0	1	1	1	1
0	3	2	1	1

0	3	2	0	0
0	2	2	0	0
2	3	3	1	1
2	1	1	0	0
1	2	2	2	2
2	4	3	2	2
2	3	1	0	0
2	2	3	2	2
1	3	3	0	0

2	4	2	1	1
2	2	3	2	2
0	2	3	0	0
2	4	3	1	0
1	4	3	2	2
2	3	3	2	2
2	2	3	0	0
2	4	3	2	2
0	2	2	0	0

0	3	3	0	0
1	3	2	1	1
0	1	0	0	0
2	4	2	0	2
2	4	4	2	2
2	4	4	2	2
2	4	4	2	2
2	4	4	1	1
2	4	4	2	2

2	2	2	0	0
2	0	2	0	0
2	4	4	0	0
2	4	4	2	2
0	0	1	0	0
1	2	2	0	0
0	1	1	2	1
2	3	3	2	2
1	4	3	0	0

0	3	2	1	1
0	0	2	1	1
0	2	1	1	1
2	4	2	1	0
0	3	0	0	0
1	2	2	1	0
2	1	1	0	0
2	3	1	2	1
0	2	2	2	2

0	2	2	0	0
0	3	3	0	0
0	3	3	0	0
2	2	3	1	0
2	4	4	0	0
2	4	4	2	1
2	2	0	0	0
1	0	0	0	0
2	4	2	0	0

2	4	4	2	2
2	3	3	0	0
2	4	4	0	0
2	4	4	0	0
2	4	4	0	0
1	4	3	0	0
0	2	3	0	0
2	4	3	0	0
2	3	2	1	1

1	4	3	0	0
2	2	2	0	0
2	4	4	0	0
2	3	2	1	1
2	4	4	2	2
0	2	2	0	0
1	3	3	0	0
2	3	4	0	0
1	2	2	0	0

2	4	3	1	2
2	0	1	0	0
0	3	1	0	0
1	2	2	1	1
0	2	2	0	0
0	3	3	0	0
2	3	2	0	0
2	3	3	2	2
2	3	1	2	2

2	3	2	2	1
0	1	1	2	2
0	1	2	0	0
0	3	3	1	1
1	1	1	0	0
0	0	1	0	2
0	1	1	2	2
0	2	1	0	0
2	4	1	1	1

2	3	2	0	0
2	3	0	0	0
0	1	0	0	0
2	2	2	0	1
2	3	1	1	1
0	3	2	1	1
0	3	1	0	0
0	2	1	1	1
0	2	3	0	0

2	4	0	2	0
2	4	4	0	0
2	0	2	2	2
2	4	4	0	0
2	4	4	0	0
2	2	2	2	2
0	3	4	0	0
2	3	0	0	0
2	3	2	1	0

0	2	0	0	0
0	3	3	1	1
0	3	0	0	0
1	3	4	2	1
2	2	4	2	2
1	3	2	0	0
0	2	1	0	0
0	3	3	0	0
0	0	0	0	0

0	0	0	0	0
0	0	0	0	0

@8.Sentimientosde culpabilidadalucinaciones	@9.Suicidio	@10.TrabajoeinteresesApatíapérdidadeinterés.enel	@11.Energíasomática	@12.Libido
2	2	3	2	0
2	3	2	2	0
2	1	0	1	0
1	2	3	2	1
0	0	1	1	2
0	2	3	2	2
2	2	2	1	2
1	1	1	1	2

2	0	2	2	1
1	2	1	0	0
1	0	2	1	0
1	1	1	1	0
1	1	2	1	0
3	0	3	1	1
1	0	1	2	2
2	2	0	1	2
0	1	2	2	2

1	0	2	2	1
0	3	3	2	2
1	0	2	1	0
2	3	2	1	2
2	0	0	1	1
0	0	0	0	0
2	2	3	2	1
1	0	2	1	0
1	0	1	1	0

3	3	3	2	0
3	2	2	1	1
0	0	2	2	0
3	0	3	2	1
0	2	1	1	0
3	0	1	1	0
2	0	0	1	0
2	0	0	0	0
3	0	2	2	0

0	0	0	0	0
2	0	0	2	0
1	0	0	1	2
1	0	2	1	1
2	0	0	1	0
2	0	1	0	0
1	0	1	2	0
1	0	2	1	0
1	1	2	2	1

2	0	4	2	0
1	1	2	1	1
2	1	3	2	0
2	1	3	2	1
2	1	1	1	2
2	2	2	2	2
0	0	0	0	0
0	0	0	0	0
0	0	0	0	0

0	0	0	0	0
0	0	0	0	0
0	0	0	0	0
0	0	0	0	0
0	0	0	0	0
0	0	0	0	0
0	0	0	0	0
0	0	0	0	0
0	0	0	0	0

0	0	0	0	0
0	0	0	1	2
0	1	3	2	2
0	0	1	1	0
1	0	1	1	0
1	1	1	2	0
1	0	2	1	2
2	1	1	1	0
0	0	3	1	2

0	0	0	0	2
0	0	0	2	2
0	4	3	2	0
0	2	1	2	2
0	0	3	2	2
0	3	3	2	2
2	0	0	2	2
0	0	4	2	2
4	0	0	1	2

0	2	2	2	2
3	2	2	2	2
2	1	2	2	2
0	0	0	1	0
3	2	3	2	0
2	2	2	1	2
1	0	1	1	2
2	3	3	2	0
2	2	3	2	2

0	0	2	2	0
1	4	4	2	1
3	2	2	2	2
1	0	2	2	0
3	1	2	1	1
2	0	3	1	0
3	1	4	2	1
1	1	1	1	0
0	1	3	2	0

2	0	1	1	1
3	2	2	1	2
1	0	2	2	1
1	0	0	0	0
0	1	2	2	0
2	2	3	2	0
0	0	1	2	0
2	3	1	0	2
3	2	3	2	2

1	0	0	1	0
1	0	1	1	0
0	1	2	1	1
1	2	2	2	0
2	1	1	1	0
1	2	3	2	2
0	1	0	1	0
2	2	3	2	1
2	2	3	1	1

1	1	3	2	2
0	0	3	2	1
1	2	3	2	0
0	1	1	1	1
0	2	1	1	0
0	1	0	1	0
2	2	2	2	0
0	0	1	1	0
0	0	3	1	1

3	2	3	2	2
2	2	2	2	2
0	0	2	2	2
3	2	3	2	2
0	0	2	1	2
2	2	0	1	0
2	0	1	1	0
0	0	2	1	2
1	0	1	2	0

0	0	1	2	0
1	2	2	2	1
1	0	1	1	0
3	0	0	2	2
0	0	2	1	0
1	0	1	2	1
1	0	1	1	0
1	0	1	1	0
2	0	1	2	1

2	1	2	2	2
1	1	2	2	0
2	1	2	1	0
2	0	0	1	0
2	0	0	1	0
2	1	2	1	1
0	2	2	2	2
2	4	1	2	1
0	1	1	1	2

0	0	2	2	2
2	0	2	2	2
0	0	0	1	2
0	0	0	2	1
1	0	4	2	0
0	0	3	2	2
0	0	3	2	2
0	0	1	1	0
2	3	3	2	0

3	1	3	1	0
2	0	1	1	0
2	1	2	2	1
2	1	2	2	2
1	0	2	1	1
2	0	0	1	0
0	1	2	2	0
0	2	0	1	0
1	1	1	1	0

0	0	0	1	0
0	0	4	2	0
3	0	3	2	2
1	0	3	2	0
0	0	4	2	2
0	0	4	2	0
2	0	4	1	2
2	0	0	2	0
2	0	0	1	2

0	4	2	2	2
2	2	3	1	0
0	0	1	1	2
2	2	3	2	1
1	0	0	1	2
2	2	1	1	2
2	1	0	2	2
3	2	3	2	1
0	2	1	2	0

1	2	2	0	1
4	1	2	1	2
0	0	0	1	0
1	2	0	1	0
0	0	1	1	2
0	0	2	1	1
3	0	1	1	1
2	0	2	2	2
3	2	2	2	0

3	3	2	2	2
1	1	0	1	1
3	0	1	2	2
2	0	0	0	1
0	1	0	1	1
0	0	2	1	1
0	0	0	1	1
3	0	2	2	1
2	1	0	0	0

0	0	0	0	0
0	0	2	2	1
2	3	2	2	2
1	0	0	0	1
3	2	2	2	2
3	1	2	2	2
2	1	4	2	2
3	2	3	1	0
3	0	1	1	2

0	0	1	0	0
2	0	0	0	0
1	2	4	2	0
3	2	3	1	2
2	1	0	0	2
0	0	2	2	0
2	0	2	1	0
2	3	1	2	0
2	3	2	2	2

1	0	3	2	0
2	2	1	1	0
3	0	1	2	2
2	3	0	0	1
2	0	2	2	0
2	0	1	0	0
2	0	0	1	1
3	2	2	1	0
0	0	2	2	0

2	0	1	1	0
3	1	0	1	0
0	0	3	1	1
2	0	1	1	1
2	0	3	2	1
3	1	3	1	1
0	1	3	2	0
2	2	2	1	1
0	0	0	0	0

0	0	2	2	2
0	0	1	1	0
3	0	2	2	2
2	2	2	2	2
2	0	1	2	0
1	0	3	2	0
0	0	0	1	2
2	0	1	1	0
2	0	2	1	1

2	0	2	0	0
1	0	3	2	2
1	1	2	2	1
1	1	1	1	2
0	0	3	0	2
2	0	3	2	2
1	2	2	1	0
1	0	4	2	2
1	0	1	2	0

1	0	0	0	0
4	2	4	2	2
2	3	3	2	2
2	0	4	2	2
2	0	0	0	2
1	1	4	2	2
0	2	4	2	2
0	2	3	2	2
2	1	2	2	1

2	2	3	2	2
2	2	3	2	2
0	0	1	1	1
0	0	2	2	2
1	0	2	2	2
2	2	2	2	2
0	2	2	2	2
2	2	3	2	2
1	1	1	2	2

0	1	1	2	2
0	0	3	1	0
4	0	0	1	0
0	0	1	1	0
0	0	2	1	1
0	0	0	0	2
0	0	2	2	2
0	0	3	2	2
0	0	0	0	0

2	0	3	2	2
1	0	2	2	2
2	2	3	2	1
1	1	3	2	2
2	1	1	1	2
2	2	2	0	0
2	2	3	2	2
1	1	1	1	0
2	1	2	1	0

1	1	2	1	0
2	1	4	2	2
3	0	1	1	0
2	0	3	2	0
3	0	0	1	2
2	0	1	0	0
0	0	1	1	1
2	2	3	1	0
1	0	2	2	1

2	1	1	1	0
0	1	1	1	0
2	0	1	0	0
0	0	0	1	0
0	0	0	1	1
2	2	1	2	0
2	1	1	1	1
2	0	0	2	2
1	0	1	1	0

2	1	0	2	0
2	0	0	1	0
2	0	1	1	0
2	0	0	1	2
0	0	2	0	0
2	3	3	2	0
2	2	2	2	2
0	0	1	1	0
3	1	1	1	0

2	0	0	1	0
1	0	2	2	0
0	2	1	1	0
0	0	0	0	0
1	1	0	1	2
0	2	3	2	0
3	1	1	0	2
1	1	3	2	2
2	1	1	2	1

0	0	1	0	1
0	1	3	2	1
2	2	0	1	0
3	1	3	2	0
3	2	2	1	0
2	1	2	2	2
0	0	2	2	2
2	1	2	2	0
1	0	2	0	0

3	0	1	2	2
0	0	3	2	0
0	0	1	1	0
0	0	4	2	2
0	0	4	2	2
2	0	4	2	2
0	0	4	2	2
0	0	4	2	0
3	0	4	2	2

0	0	0	0	2
0	0	0	0	1
2	1	4	2	2
2	2	4	2	2
0	1	1	2	2
0	0	1	1	2
0	0	0	0	1
3	3	2	1	1
0	2	2	2	1

2	2	1	1	2
0	0	0	1	0
0	0	1	2	1
1	0	2	2	2
2	1	0	1	2
2	0	0	1	2
0	0	3	1	0
2	1	1	2	0
2	0	2	1	0

2	0	0	0	1
2	2	2	1	2
2	0	1	1	0
2	2	2	2	0
1	0	4	2	2
4	2	4	2	2
1	0	0	0	2
0	0	1	1	0
0	0	4	2	2

0	2	0	0	2
2	0	3	2	2
1	0	0	1	0
0	0	4	2	2
1	0	0	2	2
3	3	2	1	2
0	0	0	0	0
0	0	2	2	2
0	2	2	1	2

2	2	2	2	0
2	0	0	0	0
1	2	2	2	1
1	1	1	1	1
2	2	1	2	0
1	0	1	2	0
3	2	1	1	1
3	1	2	1	2
2	1	1	1	1

0	0	3	2	2
0	0	1	1	1
2	2	0	0	1
3	1	2	2	2
3	3	1	2	1
1	0	0	0	0
3	2	2	2	0
0	2	1	1	1
2	0	1	1	2

2	0	2	1	2
1	0	2	2	1
0	0	1	0	1
2	1	0	1	0
1	0	1	2	0
1	0	0	0	2
2	0	1	1	0
2	1	0	0	2
1	0	1	1	2

1	0	2	2	1
2	0	2	2	2
0	0	0	0	0
1	2	2	1	2
2	0	1	1	0
2	2	1	1	0
1	1	1	1	2
2	0	2	1	1
2	0	2	2	0

0	0	3	2	2
2	0	2	2	2
0	0	0	0	0
1	0	0	0	2
3	0	4	0	2
0	1	4	2	2
0	2	4	2	0
0	0	0	0	0
0	0	2	1	0

0	0	2	1	2
1	2	1	2	2
2	0	0	1	0
4	2	3	0	1
3	0	2	2	0
2	2	2	2	2
1	0	3	1	2
3	1	2	1	2
0	0	0	0	0

0	0	0	0	0
0	0	0	0	0

@13.RetrasoLentitudpsicomotoradelpensamientodelhablay	@14.AgitaciónPuedecoexistirconretraso	@15.AnsiedadsomáticaAcompañantesfisiológicosdeansieda	@16.HipocondríaPreocupaciónporlasaludfísicaNocalif	@17.Pérdidadepercepción
2	0	2	1	1
2	0	2	0	0
1	0	1	0	1
0	3	3	2	0
1	1	1	0	0
1	1	0	1	0
0	1	2	0	0
1	1	1	1	0

0	1	2	1	0
0	1	1	2	0
1	2	2	1	0
0	2	3	1	0
0	0	2	1	0
0	0	2	1	0
2	1	2	2	0
0	0	1	2	0
1	0	2	0	0

1	0	4	2	0
1	0	3	0	1
0	1	3	0	0
1	0	2	1	0
0	0	3	0	0
0	0	2	1	0
1	0	3	2	0
1	1	1	1	0
0	0	1	1	1

0	1	4	1	0
0	2	2	1	0
1	0	2	2	1
0	1	3	2	0
1	0	1	1	0
1	0	2	2	0
1	0	2	2	0
1	1	1	1	0
1	1	2	1	0

0	0	2	2	0
0	0	2	2	1
1	0	2	2	1
1	0	2	1	0
1	0	1	0	0
1	1	2	2	0
0	0	3	2	0
0	0	4	0	0
2	0	2	3	0

1	2	2	3	0
0	1	1	2	0
0	2	3	2	0
1	1	1	0	0
0	0	1	0	0
1	0	3	0	0
0	0	0	0	0
0	0	0	0	0
0	0	0	0	0

0	0	0	0	0
0	0	0	0	0
0	0	0	0	0
0	0	0	0	0
0	0	0	0	0
0	0	0	0	0
0	0	0	0	0
0	0	0	0	0
0	0	0	0	0

0	0	0	0	0
0	1	0	0	0
1	0	2	0	0
1	1	0	1	1
2	0	2	3	1
1	0	1	0	0
1	0	0	1	0
1	0	2	0	0
0	0	3	0	0

0	0	2	0	0
0	0	0	0	0
0	0	3	0	0
0	2	0	0	0
0	0	0	0	0
0	0	1	0	0
1	0	4	0	0
0	0	3	0	0
0	0	4	0	0

0	0	0	0	0
2	0	3	0	0
1	0	3	0	0
0	0	1	0	2
0	0	1	0	0
0	1	2	0	1
0	1	2	0	2
1	0	2	0	0
3	0	3	0	0

2	0	2	0	0
2	0	4	0	0
1	1	2	0	1
2	0	3	0	0
2	1	2	0	1
2	1	1	0	1
2	0	2	3	0
1	1	2	0	1
3	1	2	3	0

1	1	2	0	1
1	2	2	0	1
1	0	3	2	0
2	0	1	3	0
2	0	4	3	0
3	0	3	1	0
1	1	2	1	0
0	1	2	0	0
0	2	3	0	0

0	0	0	1	0
0	0	2	0	0
1	0	4	3	0
1	1	2	1	0
0	1	3	1	0
2	0	0	1	0
0	0	1	1	0
0	2	2	1	0
0	2	2	2	0

2	1	3	2	0
0	1	1	2	0
0	3	2	2	0
0	1	3	2	0
0	1	2	0	1
1	0	1	1	0
0	1	1	0	0
1	1	1	1	0
1	0	0	0	0

1	0	2	1	1
1	0	3	1	0
0	1	2	0	0
0	1	3	0	0
0	1	3	2	0
0	2	3	0	0
2	0	2	3	0
0	1	2	1	0
0	0	2	1	0

1	1	1	1	0
2	1	1	0	0
0	2	2	0	0
1	0	3	2	0
1	2	3	3	0
0	2	2	1	0
0	0	2	0	1
0	0	0	0	0
0	0	1	0	0

0	0	2	2	0
0	0	2	3	0
1	0	2	3	0
0	0	3	1	0
0	0	1	0	0
0	0	2	2	0
0	0	4	3	0
1	0	1	0	0
0	0	3	0	0

0	0	2	0	0
1	0	1	1	0
0	1	0	0	0
1	0	3	0	0
2	0	3	0	0
1	0	2	0	0
1	1	3	0	0
1	0	2	1	0
1	0	1	2	1

1	1	2	3	0
2	1	1	1	1
1	1	1	1	0
1	1	1	1	1
1	1	2	3	1
1	1	2	1	0
1	1	3	3	0
2	0	1	0	1
1	0	2	3	0

0	0	2	0	0
0	0	2	0	0
1	0	2	0	0
0	0	0	0	0
0	0	4	0	0
0	0	4	0	0
0	0	2	0	0
0	0	3	0	0
0	0	3	0	0

1	1	2	0	1
1	0	1	0	0
1	0	0	0	0
1	0	3	0	0
0	1	2	0	0
0	1	2	0	0
1	0	1	0	0
2	0	3	0	0
1	0	1	0	0

0	1	2	0	0
2	1	1	0	0
0	1	2	0	0
1	1	1	0	0
0	1	2	0	0
0	1	2	0	0
1	0	2	0	0
2	0	2	0	0
0	1	2	0	0

2	1	3	2	0
1	0	2	1	0
0	0	2	0	0
0	0	1	0	0
0	0	3	0	0
1	1	2	0	1
0	2	2	0	0
2	0	2	2	1
1	0	3	0	0

0	0	1	0	2
1	0	1	0	0
2	0	0	0	0
0	1	0	0	1
0	1	3	3	0
1	1	1	3	0
3	0	1	2	0
0	2	2	0	0
1	1	1	1	0

2	0	2	2	1
0	0	1	0	1
1	1	2	2	0
0	1	1	1	0
0	1	2	0	0
1	1	3	2	1
0	0	3	1	0
2	0	1	0	0
1	0	1	1	0

0	0	0	2	0
0	1	3	2	0
0	0	2	1	0
0	0	1	0	0
1	2	2	1	0
0	0	2	1	0
0	2	2	2	0
2	1	3	1	0
2	1	2	2	0

0	2	1	1	0
0	2	2	2	0
0	0	2	0	0
2	1	2	0	0
2	1	3	2	0
2	0	3	0	0
0	0	1	0	0
0	0	2	3	0
0	0	3	2	0

0	0	3	3	0
0	0	1	0	0
0	0	2	0	0
0	0	3	3	0
1	0	0	0	0
0	0	2	0	0
0	0	2	3	1
0	1	1	0	0
0	0	2	1	0

2	0	2	1	1
2	1	3	3	1
2	1	1	2	1
0	1	1	2	0
0	0	0	0	0
2	1	1	0	1
0	1	1	2	1
0	0	3	1	0
0	0	1	0	0

0	0	3	0	0
0	0	4	0	0
0	0	3	0	0
0	0	4	0	0
0	0	1	0	0
0	0	3	0	0
0	0	2	0	0
0	0	4	0	0
2	0	2	0	0

2	0	3	0	0
2	0	3	0	0
0	0	3	0	0
1	0	2	0	0
0	0	3	0	0
0	0	2	0	0
0	0	0	0	1
1	0	3	0	1
0	0	1	0	0

0	0	2	0	0
2	0	2	0	0
1	0	1	0	0
0	0	0	0	0
1	0	2	0	0
0	0	2	0	0
1	0	1	1	0
1	0	3	0	0
0	0	0	0	2

1	0	3	2	0
2	0	2	1	1
1	1	2	0	0
1	0	2	3	0
1	1	2	0	1
0	1	1	1	1
1	0	3	1	1
1	0	1	2	1
1	0	1	1	0

1	0	1	0	1
1	0	3	1	0
0	1	2	1	0
3	1	3	3	0
1	1	1	2	0
1	1	2	1	0
0	2	2	2	0
2	0	2	1	0
1	1	3	2	0

1	1	2	1	0
1	0	2	1	0
2	0	2	3	0
1	0	2	1	0
0	2	2	2	0
1	0	3	2	0
2	0	2	0	0
0	1	2	1	0
0	1	2	2	1

1	1	2	2	0
1	1	2	2	0
1	0	2	2	0
1	1	2	2	1
2	0	1	0	0
2	0	2	2	0
2	0	3	2	0
0	0	2	2	0
2	0	2	2	0

2	0	2	2	0
1	1	2	2	0
1	0	1	2	0
0	0	0	0	0
1	0	2	0	0
3	0	3	3	1
0	0	1	1	1
1	0	3	0	1
1	0	2	1	0

0	0	2	2	0
1	0	2	1	0
2	0	3	2	0
0	1	2	3	0
0	1	2	2	0
1	0	3	2	0
1	0	2	2	0
2	0	0	2	0
1	0	1	2	0

0	1	2	3	0
0	0	3	1	0
0	0	0	0	0
0	0	2	0	0
2	0	2	0	0
2	0	2	0	0
2	0	4	0	0
0	0	3	0	0
2	0	4	0	0

0	0	4	0	0
0	0	0	0	0
0	0	3	0	0
2	0	0	0	0
0	0	0	0	0
1	0	0	0	0
0	0	1	0	0
1	0	0	0	0
1	0	0	0	0

0	1	1	0	0
1	0	0	0	0
1	1	1	0	0
0	0	2	0	0
0	0	0	0	0
0	1	2	0	0
1	1	2	2	1
2	0	1	1	1
1	0	0	0	0

0	1	1	2	0
1	0	1	1	0
1	0	2	2	0
0	1	3	3	1
0	0	4	0	0
2	0	2	0	0
0	0	1	0	0
0	0	1	0	2
4	0	3	0	0

1	0	1	0	0
0	1	1	0	0
1	0	3	0	0
1	0	3	0	0
0	0	2	0	0
1	1	3	3	0
0	0	1	2	0
0	0	2	0	0
0	1	3	0	0

0	0	2	0	0
0	1	0	1	1
0	1	4	2	0
0	1	2	0	0
0	1	2	2	0
1	0	2	1	1
0	1	0	1	0
1	1	2	0	0
0	0	1	0	0

2	0	2	3	0
0	0	1	2	0
0	0	3	0	0
1	0	3	0	0
0	0	0	1	0
0	0	0	0	1
1	0	2	0	0
0	1	1	0	0
0	1	1	0	0

1	0	2	0	0
0	0	4	0	0
1	0	1	2	0
1	1	4	2	0
1	0	3	1	0
0	0	0	0	1
0	0	0	2	0
0	0	0	0	0
0	0	1	0	0

0	0	2	0	0
1	0	0	0	0
0	0	0	0	0
1	0	1	0	0
0	0	0	0	0
0	0	2	1	0
0	0	0	0	0
1	1	1	0	0
2	1	3	1	0

0	0	4	0	0
0	0	3	0	0
0	0	3	0	0
0	0	2	0	0
0	0	4	0	0
0	0	2	0	0
0	0	2	0	0
0	0	2	0	0
0	0	1	0	0

0	0	0	0	0
1	1	2	0	0
1	0	0	0	0
0	0	3	0	0
2	0	3	0	0
1	0	2	0	0
1	1	0	1	0
0	1	3	2	0
0	0	0	0	0

0	0	0	0	0
0	0	0	0	0

@1.Insomnioalquererdormir	@2.Insomniodemedianoche	@3.Insomniodurantelamadrugada	@4.SueñoExcesivo	@5.Estadodeánimotristeza
3	3	3	0	2
3	3	0	0	3
1	1	0	0	1
3	3	3	0	0
3	3	3	0	2
3	3	3	0	3
2	2	0	0	1
2	2	1	0	2

0	0	0	0	2
3	2	1	0	2
3	1	1	1	2
1	0	1	0	2
2	1	1	0	2
0	0	0	0	2
3	3	1	0	2
0	0	0	0	2
3	2	0	0	2

3	1	0	0	3
0	0	0	3	3
0	0	0	0	1
3	3	3	0	3
3	3	3	0	1
0	0	0	0	0
1	1	0	0	3
1	0	0	0	1
3	3	1	0	1

3	3	2	0	3
2	2	2	0	2
2	2	1	0	1
3	3	3	0	2
3	0	3	0	3
0	0	0	0	2
3	3	0	0	2
0	2	2	0	2
3	3	0	0	2

0	0	0	0	1
0	3	0	0	2
3	0	2	0	1
3	3	2	0	1
2	2	2	0	1
0	0	0	0	1
2	2	2	0	2
1	0	0	0	1
2	2	0	0	2

3	0	1	0	2
1	1	0	0	2
2	3	1	1	3
2	0	2	0	3
2	3	0	0	2
2	3	1	0	2
3	3	3	0	1
3	3	1	0	2
0	3	3	0	3

3	3	2	0	1
3	3	3	0	3
0	0	0	0	1
0	0	0	0	0
3	0	0	0	3
0	0	0	2	2
1	3	1	0	1
0	0	0	0	1
3	3	0	0	3

0	0	0	0	3
0	2	0	0	1
3	3	0	0	2
0	0	0	0	1
0	0	2	1	2
1	3	0	1	2
0	1	0	1	1
0	1	2	0	2
3	2	0	0	2

3	3	2	0	2
3	2	0	0	0
3	3	3	0	3
3	3	2	0	2
3	3	3	0	2
3	3	3	0	3
3	3	0	0	3
0	0	0	0	3
3	3	3	0	3

3	0	0	0	3
3	0	0	0	3
0	0	0	1	3
0	2	1	0	2
3	0	3	0	2
3	3	2	0	1
3	0	0	0	2
3	3	0	0	3
3	1	2	0	3

3	3	0	0	2
3	3	3	0	3
2	3	2	0	2
2	3	0	0	2
2	3	1	0	3
3	3	2	0	2
3	3	3	0	3
2	3	3	0	2
3	3	2	0	3

2	0	2	0	2
3	3	2	0	3
3	3	0	3	2
2	2	0	0	0
3	3	3	0	3
3	2	0	0	3
0	2	0	0	1
0	0	0	0	3
3	3	3	0	3

0	0	0	0	1
0	1	0	0	0
0	0	0	0	3
3	3	2	0	2
0	1	3	0	1
3	3	3	0	3
2	2	1	0	1
3	3	3	0	2
2	3	2	0	2

1	0	0	1	3
3	3	3	0	1
3	3	0	0	2
3	3	1	0	0
3	2	1	0	2
0	1	1	0	2
3	3	2	0	3
1	1	1	0	2
1	3	1	0	2

0	0	0	1	2
3	3	3	0	2
0	0	0	1	2
3	3	3	0	2
3	1	0	0	1
3	3	2	0	2
2	0	0	1	2
3	3	1	0	2
2	0	0	0	1

3	3	3	0	1
3	3	2	0	2
3	0	2	0	2
3	3	0	0	2
3	2	0	0	2
3	2	0	0	2
1	0	0	0	0
2	2	1	0	1
2	2	0	0	2

2	1	0	0	2
2	3	3	0	2
3	3	1	0	2
1	0	0	0	2
1	0	0	1	1
1	1	0	0	2
0	0	2	0	2
0	0	3	0	2
3	3	3	0	2

2	3	0	0	2
3	0	1	0	2
3	0	3	0	2
0	0	0	0	3
3	0	3	3	3
3	1	3	0	2
3	0	3	0	3
2	0	2	0	2
3	3	2	0	2

3	3	3	0	3
2	0	3	0	2
0	3	2	0	2
3	3	3	0	2
3	3	3	0	2
2	3	2	0	2
3	3	0	0	2
3	3	0	0	2
2	3	0	0	2

3	3	3	0	1
0	0	0	3	2
3	3	3	0	3
3	3	3	0	3
0	3	3	0	2
1	2	0	0	3
3	2	0	0	3
3	3	3	0	1
3	3	3	0	1

3	3	0	0	3
3	2	0	0	3
0	2	0	0	2
3	0	0	1	3
0	2	0	0	1
0	1	0	0	3
0	0	0	0	3
3	3	3	0	3
0	3	3	0	1

2	0	0	0	2
3	0	2	0	3
3	0	2	0	2
3	3	0	0	3
0	3	3	0	2
3	0	0	0	2
1	0	3	0	2
3	0	2	0	3
3	1	3	0	2

3	0	2	0	3
2	3	3	0	2
3	0	0	0	2
3	0	0	0	0
3	1	2	0	3
2	0	0	0	1
0	0	0	1	2
3	2	0	0	2
0	0	0	1	1

0	0	0	0	0
0	2	0	0	2
0	0	0	0	2
0	0	0	0	1
0	3	2	0	2
3	3	3	0	2
3	3	3	0	3
1	2	2	0	2
0	2	1	0	1

1	0	0	1	1
0	1	0	0	1
3	3	3	0	3
3	3	3	0	3
3	3	3	0	3
3	3	0	0	1
1	1	0	0	2
3	3	0	1	3
3	3	2	0	3

3	2	0	0	1
3	3	3	0	2
3	0	0	0	1
2	2	3	0	0
3	2	1	0	2
1	1	2	0	1
3	1	3	0	2
3	2	2	0	3
2	2	2	0	2

0	2	0	0	1
1	2	1	0	2
3	2	0	0	2
1	2	0	0	2
1	0	3	0	2
2	2	2	0	2
2	1	0	0	2
3	0	2	0	2
2	0	0	0	2

3	3	0	0	2
2	1	0	0	1
2	3	0	0	3
2	1	2	0	3
2	0	0	1	2
1	2	1	1	2
2	3	1	0	2
2	3	3	0	2
3	3	1	0	2

3	0	0	0	2
3	3	1	0	2
2	3	0	0	2
2	3	0	0	0
0	0	0	0	0
3	3	2	0	3
3	3	2	0	2
3	3	3	0	2
3	3	3	0	2

0	0	0	0	1
3	3	3	0	3
3	3	3	0	3
3	1	3	0	3
3	3	2	0	1
3	3	3	0	3
3	3	3	0	3
3	3	3	0	3
3	3	1	0	2

3	3	3	0	3
3	2	0	0	3
0	2	0	0	0
0	0	0	0	2
3	2	0	0	3
3	3	3	0	3
0	1	0	0	2
0	0	0	0	3
3	3	2	0	2

1	1	3	0	3
1	1	2	0	2
3	0	2	0	1
3	3	3	0	1
0	1	0	0	1
3	3	3	0	2
3	3	3	0	0
3	3	0	0	3
0	0	0	0	0

3	0	0	0	3
3	3	2	0	2
2	3	3	0	2
3	3	3	0	3
2	3	3	0	2
3	0	3	0	2
3	2	0	0	3
0	0	0	0	2
3	2	3	0	2

3	3	1	0	1
3	3	0	0	3
3	2	0	0	2
0	3	1	0	3
3	0	0	3	2
3	0	0	0	2
3	3	0	0	2
0	0	3	0	3
3	0	0	0	0

2	3	0	0	3
3	0	0	2	1
3	1	0	0	2
0	3	0	0	1
0	1	0	1	2
3	2	0	0	2
1	3	1	0	1
2	3	0	0	1
0	2	0	0	1

1	3	1	0	2
3	3	3	0	2
3	2	2	0	2
0	3	2	0	1
2	0	3	0	2
0	3	0	0	2
1	3	3	0	3
0	2	0	0	1
2	3	1	0	2

0	3	1	0	2
2	0	0	0	1
3	2	2	0	3
1	0	1	0	1
1	3	1	0	2
3	3	3	0	0
3	3	3	0	2
3	3	2	0	2
2	2	2	0	2

2	3	2	0	2
3	3	3	0	2
3	0	0	0	1
3	3	2	0	3
3	3	2	0	3
3	3	3	0	2
3	3	3	0	2
3	3	3	0	3
1	1	0	0	2

0	2	0	0	2
3	3	2	0	2
1	0	0	0	0
3	3	3	0	3
3	3	3	0	3
3	3	3	0	3
3	3	3	0	3
3	3	2	0	3
3	3	3	0	3

3	3	3	0	1
3	3	3	0	0
3	3	3	0	3
3	3	3	0	3
0	3	0	0	0
3	1	0	0	2
3	0	0	0	2
3	2	3	0	2
3	0	1	0	3

3	0	0	0	2
3	1	0	0	2
3	3	0	0	2
3	3	0	0	2
3	3	0	0	2
3	3	1	0	2
3	3	3	0	1
3	3	2	0	2
3	3	0	0	2

3	3	0	0	1
3	3	0	0	3
3	0	0	0	2
3	3	2	0	2
3	3	3	0	2
3	3	2	0	3
3	3	3	0	0
1	0	1	0	0
3	3	3	0	3

3	3	3	0	3
3	3	3	0	2
3	3	3	0	3
3	3	3	0	3
3	3	3	0	3
3	3	0	0	3
0	0	0	0	1
3	3	3	0	3
3	2	2	0	2

2	1	0	0	3
3	3	2	0	1
3	3	3	0	3
3	3	2	0	1
3	3	3	0	3
2	2	0	0	1
3	3	1	0	2
3	3	3	0	3
2	1	1	0	2

3	3	3	0	3
3	3	2	0	0
2	2	0	0	2
2	2	2	3	2
0	2	0	0	2
0	0	0	0	2
2	3	2	0	3
3	3	3	0	3
3	3	2	0	2

3	3	2	0	1
3	3	0	0	1
0	0	0	0	1
3	3	0	0	3
2	3	1	0	2
0	3	0	0	0
2	2	0	0	1
3	0	0	0	1
3	3	1	0	3

3	3	3	0	2
3	3	3	0	3
0	0	0	0	2
2	3	2	0	3
3	3	3	0	3
3	0	0	0	2
3	0	0	0	2
3	3	0	0	2
3	1	0	0	2

3	3	3	0	3
3	3	3	0	3
0	2	3	0	0
3	3	3	0	3
3	3	3	0	3
3	3	2	0	3
3	3	3	0	3
0	3	3	0	2
3	3	3	0	3

0	0	0	0	2
0	0	0	2	3
3	3	0	0	2
3	3	2	0	3
3	3	3	0	1
3	3	1	0	3
0	0	0	2	1
1	0	0	2	2
0	0	0	0	0

0	0	0	0	0
0	0	0	0	0

@6.Estadodeánimo ansiedad	@7.Síntomasdepánicofobia	@8.Estadodeánimoirritabilidad	@9.Mejoramientodelestadodeánimo	@10.Variacióndelestadodeánimo
1	0	3	0	0
2	2	2	2	0
1	2	1	0	1
3	2	2	0	1
3	2	3	0	0
2	0	0	1	1
2	1	1	1	0
1	1	1	0	2

2	1	1	2	0
2	2	1	1	0
2	0	2	0	1
1	1	1	1	0
1	1	2	0	1
1	0	1	0	0
2	1	0	1	1
1	1	2	0	1
1	1	2	1	1

2	2	3	0	0
0	3	2	3	3
1	2	0	0	0
1	2	0	3	2
2	2	0	0	1
1	0	1	0	0
0	0	2	1	0
1	0	0	0	0
0	0	1	0	2

3	2	3	1	1
3	1	2	2	1
2	0	0	0	1
3	1	0	2	0
2	1	2	1	2
2	1	2	1	1
2	1	1	1	0
1	1	2	0	1
3	1	1	0	3

2	1	1	0	1
1	1	2	0	1
2	0	2	0	0
2	1	2	0	1
1	0	1	0	1
2	0	2	0	0
2	3	1	0	0
1	2	1	0	0
2	0	1	1	0

2	0	2	1	0
2	1	1	1	1
2	2	2	2	0
1	1	2	0	0
1	0	1	1	0
2	0	2	1	0
2	2	3	3	3
3	2	3	0	0
3	2	3	0	0

2	2	3	1	0
3	3	1	1	0
2	0	1	0	0
1	0	0	0	0
0	0	3	3	0
2	1	0	1	0
2	0	0	0	2
0	0	0	0	2
2	1	2	1	1

3	0	2	3	0
0	0	0	0	2
0	0	0	1	0
1	0	0	0	0
0	0	0	1	2
1	1	1	1	0
1	0	2	0	1
2	0	2	1	1
2	0	1	0	0

0	0	0	0	0
1	0	2	0	0
3	0	3	1	0
1	0	1	1	0
3	0	2	1	0
3	3	3	3	0
3	0	3	1	0
3	0	3	0	0
2	0	2	0	0

2	0	2	2	0
2	0	0	2	0
3	0	2	1	0
1	0	0	0	3
2	0	2	1	0
2	2	2	0	0
0	0	0	1	0
2	0	1	1	1
2	0	2	3	0

1	0	2	1	0
1	0	0	2	0
3	0	2	1	0
2	0	2	3	2
2	2	2	1	3
2	1	2	1	2
0	1	0	2	2
2	2	1	1	0
3	3	1	3	1

2	1	2	2	3
2	1	3	2	0
1	0	1	0	0
0	1	1	0	0
2	0	3	1	0
3	0	2	2	0
1	0	1	0	0
1	0	1	0	0
3	2	3	3	3

1	0	0	0	0
1	1	1	0	0
3	1	3	0	3
2	2	1	0	0
1	1	2	0	0
2	1	1	1	1
1	0	0	1	1
3	2	1	1	0
2	2	0	0	2

3	0	2	1	0
2	2	0	0	0
3	3	1	2	0
2	1	2	1	1
2	0	2	2	1
2	1	1	1	0
2	0	1	2	0
3	1	0	1	0
1	0	1	2	1

0	0	2	2	0
2	2	1	2	0
1	1	1	1	0
3	2	2	2	1
2	2	1	0	2
2	2	2	1	2
2	0	1	0	2
3	3	1	2	3
2	0	0	0	1

2	1	1	1	1
1	0	3	1	2
2	1	2	0	1
2	0	3	0	2
3	1	2	1	2
2	1	2	0	1
1	0	1	0	1
0	1	1	0	0
1	0	1	0	0

2	0	2	1	0
1	0	2	1	0
2	1	1	1	0
1	2	1	0	0
1	0	1	0	0
2	0	2	0	0
1	0	2	0	0
2	1	2	0	1
2	0	2	2	0

2	0	2	0	3
2	0	0	0	1
0	0	2	0	1
0	0	2	0	2
2	0	2	1	3
2	2	1	0	1
3	0	2	1	1
2	0	2	0	1
2	1	2	1	0

2	3	1	1	1
0	0	2	1	2
2	0	1	3	0
2	3	2	3	1
2	0	2	2	1
1	1	2	1	0
1	1	1	2	0
1	0	1	2	0
1	0	2	0	0

1	3	1	0	0
2	0	3	0	0
1	0	2	1	0
2	0	0	0	0
2	0	0	0	0
2	1	0	0	0
3	0	1	0	0
3	3	2	0	0
2	0	1	0	0

3	0	2	3	0
2	0	2	1	0
2	0	2	1	0
2	2	3	2	0
1	0	1	0	0
2	0	2	1	0
3	0	3	0	1
3	0	3	2	0
2	0	2	0	2

2	0	1	1	1
2	0	0	0	1
2	1	0	0	1
2	0	0	0	1
2	0	2	1	0
2	0	2	0	0
1	0	1	0	1
2	0	0	0	3
2	1	2	1	1

3	2	1	0	0
2	1	0	0	0
2	2	2	1	0
1	0	3	0	0
3	1	0	0	0
1	0	1	0	1
1	0	1	1	0
2	3	0	2	3
1	0	0	0	0

1	0	1	0	0
2	0	1	0	1
2	0	2	1	1
1	0	0	0	0
2	0	3	1	1
0	0	1	0	1
2	1	1	2	2
3	0	0	3	1
2	0	2	1	1

1	0	1	0	0
0	0	0	0	0
2	3	3	2	0
2	1	1	2	0
1	0	2	0	1
2	0	2	0	0
2	0	0	0	0
3	2	1	2	1
1	0	3	2	1

0	0	1	1	0
2	0	2	0	0
2	0	3	2	1
2	0	1	1	2
1	1	3	0	0
2	1	1	0	0
2	2	2	2	0
2	2	3	2	0
2	1	2	1	2

1	2	1	1	1
2	2	1	1	1
1	1	2	2	2
2	1	2	1	2
2	2	2	2	2
1	0	2	2	0
1	0	2	0	0
1	0	2	0	0
2	2	1	0	0

2	0	2	1	0
2	0	2	0	0
3	0	1	1	0
2	1	2	0	1
1	0	2	0	0
2	0	2	1	0
2	0	2	2	0
1	0	1	0	0
2	3	0	1	0

2	0	1	1	0
2	2	2	3	0
2	1	2	1	2
2	3	2	0	0
3	3	0	0	3
2	0	2	0	1
2	0	2	0	0
2	0	0	0	0
2	1	0	0	0

2	0	3	0	0
3	0	3	3	0
2	0	0	2	0
3	3	2	0	0
1	0	0	0	0
2	0	2	0	3
2	1	3	0	0
3	0	2	1	0
2	0	2	1	0

3	0	2	1	0
3	0	2	2	0
3	0	2	0	0
2	0	2	0	0
2	0	2	1	0
2	0	2	2	0
1	0	2	0	0
3	2	3	2	0
2	0	2	1	0

2	0	0	0	3
2	1	1	0	0
1	0	0	0	1
0	0	0	0	0
1	1	1	0	1
2	1	2	0	1
2	0	1	1	1
2	0	1	1	1
0	0	0	0	0

2	2	1	1	0
2	2	2	0	0
2	2	2	1	3
2	1	2	3	1
2	0	2	1	0
2	0	2	0	0
3	3	2	1	0
2	0	0	1	3
2	1	0	3	1

1	0	1	0	0
2	3	2	3	0
1	2	1	1	0
2	2	2	2	2
1	1	2	1	0
2	2	0	1	2
1	2	0	0	2
2	1	3	3	1
2	1	3	0	1

1	0	3	1	0
2	1	1	0	0
1	0	2	1	1
2	1	0	0	1
1	2	0	0	2
0	1	3	2	0
2	1	1	0	0
1	0	0	0	2
1	2	1	2	1

2	1	2	0	1
2	0	1	1	2
2	1	2	0	2
1	0	2	2	0
1	0	0	0	2
2	2	2	2	2
2	2	3	2	0
2	0	0	0	2
2	1	0	2	2

1	0	2	0	0
2	0	2	0	0
1	0	1	0	0
1	0	2	0	0
2	0	2	0	0
2	1	1	3	0
1	0	1	0	0
2	0	2	3	0
2	1	2	0	1

1	0	1	1	1
2	0	2	2	1
2	2	2	0	1
2	2	2	0	0
2	1	2	1	1
2	2	2	1	1
2	0	2	0	0
2	2	1	1	2
2	0	0	1	1

3	0	3	2	2
2	0	1	0	2
0	0	1	0	0
3	0	2	0	0
3	0	3	1	0
3	3	2	2	0
3	0	3	1	0
3	0	3	0	0
3	0	3	2	0

2	3	0	0	0
1	0	0	0	0
3	0	2	0	3
3	2	3	3	0
1	0	0	0	0
1	0	0	0	3
2	0	0	0	1
2	2	1	1	2
3	0	2	0	1

2	2	1	1	1
0	0	0	0	1
2	0	1	0	3
2	0	2	0	3
1	0	0	1	1
2	1	2	0	1
1	0	0	1	0
2	1	0	1	2
2	0	2	1	0

2	0	0	1	0
2	1	2	1	1
2	2	1	1	3
2	2	1	3	1
3	1	2	0	0
3	0	3	0	0
1	0	0	0	0
0	0	0	0	0
2	0	0	1	0

3	0	1	1	0
0	0	3	0	0
3	0	0	0	0
0	0	0	1	0
3	0	0	0	0
2	1	2	2	0
2	0	1	0	0
2	0	2	1	0
2	0	2	0	0

2	0	2	0	0
1	0	1	0	0
3	1	3	2	0
1	0	1	0	0
2	2	1	2	1
1	0	1	0	0
2	0	2	0	0
3	1	1	1	1
1	0	2	1	2

3	0	0	1	0
0	0	1	0	0
1	0	0	0	0
2	1	2	3	0
2	0	1	0	0
2	0	1	0	0
3	0	0	0	0
2	0	0	1	0
1	1	1	0	0

1	2	3	0	1
3	0	0	0	0
1	0	0	0	0
3	0	1	2	2
3	0	1	0	1
1	0	0	0	0
0	0	0	0	0
1	1	3	0	0
2	0	1	0	1

2	0	1	2	0
1	0	0	1	0
0	0	0	0	0
2	0	1	0	0
1	0	1	0	0
2	0	1	0	1
1	1	1	0	0
2	0	1	0	0
2	2	1	1	0

3	0	3	1	0
3	3	1	0	0
2	0	1	0	0
3	0	0	1	0
3	3	3	0	0
3	1	0	1	0
3	1	3	0	0
0	0	0	0	0
2	0	2	1	0

0	0	0	0	0
2	2	2	0	0
2	0	0	0	0
3	2	2	0	0
3	1	1	0	0
2	0	1	2	0
1	0	1	0	1
2	2	2	1	1
0	0	0	0	0

0	0	0	0	0
0	0	0	0	0

@10A¿Eselestadodeánimotípicamentepeoralamarqueun	@10B¿Eslavariacióndelestadodeánimoatribuidaporelpac	@11.Calidaddeestadodeánimo	@12.Apetitodisminuido	@13.Apetitoaumentado
Noche?	Sí	2	0	3
		2	2	0
Tarde?	Sí	2	1	0
Tarde?	NO	3	0	2
		2	0	2
Tarde?	Sí	2	2	0
		1	1	0
Tarde?	Sí	3	3	0

		2	0	2
		2	1	0
Tarde?	Sí	1	0	1
		1	1	0
Tarde?	Sí	0	1	0
		0	0	1
Tarde?	Sí	1	0	2
Tarde?		1	1	0
Tarde?	Sí	3	0	3

Mañana?	NO	1	2	0
		3	0	1
Mañana?	Sí	0	0	0
Tarde?	Sí	3	3	0
Tarde?	Sí	1	2	0
		0	0	0
		2	0	0
		1	0	3
Mañana?	NO	0	0	0

Noche?	Sí	2	0	2
	NO	2	0	1
Tarde?		0	0	1
	NO	2	2	0
Tarde?	NO	3	2	0
Tarde?		2	0	2
Mañana?	Sí	3	0	1
Tarde?	Sí	2	1	0
Mañana?	NO	2	0	2

Tarde?	Sí	1	1	0
Tarde?	Sí	2	0	1
		2	0	0
Mañana?	Sí	2	0	1
Mañana?	Sí	1	0	0
		0	1	0
		1	0	1
		2	0	0
		1	0	2

		1	1	0
Noche?	Sí	0	1	0
		1	0	2
		0	1	0
		0	1	0
	Sí	1	0	0
Mañana?		0	1	0
		3	0	0
		0	0	1

		1	0	1
		0	0	0
		3	0	1
		0	0	0
		1	2	0
		1	1	0
Tarde?	NO	2	0	0
Tarde?	NO	3	0	0
Mañana?	NO	3	2	0

		3	0	0
Mañana?	NO	2	0	0
		0	0	0
		3	0	0
Mañana?	NO	2	2	0
Noche?	Sí	3	1	0
Mañana?	Sí	2	0	0
Mañana?	Sí	3	0	0
		3	0	0

1	0	0
1	0	0
1	3	3
3	0	0
3	0	0
1	1	0
3	0	3
1	3	0
1	0	2

		3	2	0
		1	1	0
		3	2	0
Noche?	Sí	0	0	0
		1	1	0
		0	1	1
		0	1	0
		1	0	0
		0	0	1

		2	0	1
Noche?	NO	3	3	0
		3	1	0
Mañana?	NO	1	2	0
Noche?	NO	2	1	0
Mañana?	NO	3	2	0
Mañana?	NO	0	3	0
	NO	3	0	3
Tarde?	Sí	3	2	0

Mañana?	NO	3	2	0
		3	0	1
Mañana?	NO	0	0	3
Mañana?	Sí	2	0	1
		3	0	3
	NO	2	0	3
Tarde?	NO	3	0	0
Mañana?	NO	0	1	0
Mañana?, Noche?	NO	2	3	0

	NO	0	1	0
		0	0	3
Tarde?	NO	3	0	3
	NO	2	1	0
	NO	1	1	0
Tarde?	NO	3	0	2
Tarde?	Sí	1	0	0
		1	1	0
Mañana?	Sí	1	2	0

		2	0	2
		1	0	0
		2	1	0
Tarde?	Sí	2	1	0
Tarde?	Sí	2	1	0
		1	1	0
		1	2	0
		1	1	0
Tarde?	Sí	0	1	0

		3	2	0
		2	2	0
		1	1	0
Noche?	Sí	3	2	0
Mañana?	Sí	1	1	0
Tarde?	Sí	2	1	0
Tarde?	Sí	1	1	0
Mañana?	NO	2	2	0
Mañana?	Sí	1	0	2

Noche?	Sí	1	1	0
Tarde?	Sí	2	2	0
Mañana?	Sí	2	1	0
Mañana?	Sí	1	1	0
Tarde?	Sí	1	0	1
Mañana?	Sí	1	0	1
Tarde?	Sí	0	0	0
		0	0	1
		0	0	1

	Sí	0	1	0
	NO	1	0	1
		0	1	0
		0	1	0
		0	1	0
		0	0	1
		0	0	1
Noche?	Sí	2	1	0
Tarde?	NO	3	1	0

Mañana?	Sí	3	0	3
Tarde?	Sí	3	1	0
Tarde?	Sí	2	0	0
Tarde?	Sí	3	0	0
Tarde?		3	0	2
Tarde?	Sí	3	1	0
Mañana?	Sí	1	2	0
Tarde?	Sí	3	1	0
		0	1	0

Mañana?	Sí	3	0	2
Noche?	NO	3	2	0
		3	1	0
Mañana?	Sí	0	0	0
		3	1	0
		3	1	0
		0	0	2
		0	2	0
		1	2	0

0	3	1
0	2	0
0	1	0
0	2	0
3	2	0
0	0	0
1	1	0
1	0	2
3	0	3

		3	2	0
		0	0	0
		1	0	1
		0	0	1
		3	0	1
		3	0	2
Noche?	Sí	3	2	0
		2	0	2
Mañana?	NO	2	0	0

Noche?	Sí	2	0	0
Mañana?	Sí	3	1	0
Mañana?	Sí	2	1	0
Tarde?	Sí	2	1	0
Mañana?	NO	3	1	0
Tarde?	Sí	3	0	3
Mañana?	Sí	3	0	1
Tarde?	Sí	3	1	0
Tarde?	Sí	3	1	1

Noche?	NO	3	1	0
	NO	0	1	0
Tarde?	NO	3	0	2
	Sí	3	0	0
		3	0	0
Tarde?, Noche?	NO	3	1	0
	NO	3	0	1
Noche?	NO	3	2	0
		0	0	0

		0	0	1
Mañana?	Sí	2	0	1
Tarde?	Sí	3	0	0
		0	0	0
	Sí	1	0	3
Mañana?	Sí	1	0	3
Mañana?	NO	3	2	0
Mañana?	Sí	2	0	0
Tarde?	Sí	2	0	2

		0	0	2
		0	0	0
		3	3	0
		2	3	0
Tarde?	Sí	1	2	0
		1	0	2
Mañana?	Sí	1	0	2
Noche?	Sí	2	0	2
Tarde?	Sí	2	1	0

		2	0	0
Mañana?	Sí	2	0	1
Tarde?	NO	1	0	0
Mañana?	Sí	1	2	0
		1	0	2
		2	1	0
		3	2	0
		3	0	2
Mañana?	Sí	1	0	2

Mañana?	Sí	1	2	0
Tarde?	NO	1	2	0
Tarde?	NO	2	1	0
Mañana?	Sí	1	0	0
Tarde?	Sí	2	1	0
		1	0	1
		0	2	0
		1	0	1
		0	1	0

		0	1	0
		0	0	2
		0	2	0
Noche?	Sí	0	1	0
		0	1	0
		0	0	2
		0	0	0
		3	1	0
		3	2	0

		3	0	2
		3	0	2
Noche?	NO	3	1	0
		3	0	0
Mañana?	NO	0	0	0
		0	2	0
		3	2	0
		0	0	0
		2	1	0

		3	0	3
		0	3	0
		0	3	0
		3	1	0
		3	3	0
Mañana?	Sí, NO	0	0	3
		1	0	0
		0	3	0
		3	0	1

2	1	0
1	1	0
0	0	0
1	0	0
1	0	0
1	1	0
0	0	0
1	0	0
1	0	1

Noche?	Sí	3	1	0
Tarde?	NO	1	0	1
Tarde?	Sí	3	1	0
Noche?	Sí	3	1	0
Noche?	Sí	3	0	2
Noche?	Sí	3	1	0
Mañana?	Sí	3	1	0
Tarde?	Sí	0	1	0
	Sí	0	0	0

Noche?	NO	3	3	0
		1	0	3
Mañana?	NO	1	2	0
	Sí	3	0	2
		3	0	0
		3	0	0
		2	2	0
Mañana?	NO	3	1	0
Tarde?	Sí	3	1	0

		3	2	0
		3	2	0
		1	0	2
Tarde?	Sí	3	1	0
		1	1	0
Tarde?	NO	2	0	1
Tarde?	Sí	1	2	0
Tarde?	Sí	3	3	0
Mañana?	Sí	3	2	0

		2	0	3
		2	0	1
Mañana?	Sí	2	0	0
Noche?	Sí	0	2	0
Tarde?	Sí	2	2	0
		2	2	0
		1	1	0
Tarde?	NO	1	1	0
Tarde?	Sí	1	1	0

Tarde?	Sí	2	0	2
Noche?	Sí	1	0	1
Tarde?	Sí	2	1	0
		1	0	1
Noche?	NO	2	0	0
Mañana?	NO	1	3	0
		3	1	0
Noche?	NO	1	1	0
Noche?	NO	2	1	0

		2	0	2
		1	0	2
		1	1	0
		0	0	0
		1	2	0
		2	2	0
		0	0	0
		2	2	0
Tarde?	Sí	1	0	0

Mañana?	Sí	2	2	0
Tarde?	Sí	2	2	0
Tarde?	Sí	1	0	2
Mañana?	Sí	2	1	0
Tarde?	Sí	2	2	0
Tarde?	NO	2	2	0
		1	0	3
Tarde?	NO	1	3	0
Tarde?	Sí	1	0	0

Mañana?	NO	1	0	3
Tarde?	NO	2	2	0
Mañana?		0	0	0
		0	3	0
		0	3	0
		0	3	0
		0	3	0
		0	1	0
		0	3	0

		0	0	1
		0	0	0
Tarde?		0	0	1
		1	3	0
		1	0	0
Noche?	Sí	2	0	2
Tarde?	Sí	3	3	0
Mañana?		3	1	0
Mañana?	Sí	3	0	3

Noche?	Sí	3	1	1
Mañana?	Sí	3	1	0
Noche?	NO	3	1	0
Mañana?	Sí	3	1	0
Noche?	Sí	3	0	1
Noche?	Sí	3	1	1
		3	0	2
Mañana?	NO	0	2	0
		3	2	0

		0	0	0
Noche?	Sí	3	0	2
Noche?	NO	3	0	2
Mañana?	Sí	3	1	0
		2	0	3
		2	3	0
		0	0	0
		0	0	0
		0	0	0

0	3	0
0	0	3
1	0	3
0	0	0
0	0	2
1	0	2
0	0	1
0	0	1
0	1	0

		0	0	2
Mañana?	Sí	1	0	0
Mañana?	Sí	2	0	2
Mañana?	Sí	1	0	1
Tarde?	Sí	1	2	0
Mañana?	Sí	1	0	2
Mañana?	Sí	1	0	0
Mañana?	NO	0	0	3
Noche?	NO	1	0	1

	3	1	0
	3	0	0
	3	0	2
	3	1	0
Sí	3	0	0
	3	0	0
	0	0	0
	3	2	0
	3	2	0

Mañana?, Noche?	Sí	0	2	0
		0	2	0
		1	0	0
Mañana?, Tarde?	NO	0	2	0
Mañana?	Sí	2	0	2
		0	0	0
		0	3	0
Mañana?, Noche?		0	0	0
Tarde?	Sí	3	1	0

Noche?	NO	1	0	3
Noche?	NO	3	0	3
		0	1	0
		2	0	0
		3	1	0
Noche?	Sí	1	1	0
		3	0	0
		1	1	0
		3	0	2

0	3	0
0	0	2
0	2	0
0	0	0
0	0	0
0	2	0
0	0	0
0	0	0
0	1	0

		0	0	0
		1	1	0
		1	0	1
		1	2	0
		2	3	0
		2	0	0
Tarde?	Sí	1	0	2
Tarde?	Sí	3	0	3
		0	0	0

0	0	0
0	0	0

@14.Pesodisminuciónduranteelasúltimasdossemanas	@15.Pesoaumentodurantelasúltimasdossemanas	@16.Concentración tomadedecisiones	@17.Perspectivapropiaanálisis	@18.Perspectivafutura
0	3	1	1	2
3	0	2	1	3
1	0	1	1	1
0	1	1	0	2
0	3	1	0	1
2	0	1	0	1
0	0	2	2	0
3	0	1	0	1

0	3	0	2	1
1	0	3	0	1
0	0	1	0	1
2	0	1	1	2
0	0	1	0	1
0	2	0	1	0
0	3	0	1	0
2	0	0	1	2
0	3	2	0	3

0	3	1	1	1
0	0	3	0	3
0	0	0	1	1
0	3	2	1	3
3	0	0	1	1
0	0	0	0	0
1	1	2	2	2
0	3	1	1	0
0	0	0	1	1

0	3	1	1	2
0	0	2	3	1
0	0	1	0	1
3	0	3	2	2
3	0	1	0	1
0	2	2	2	1
0	2	0	1	1
2	0	2	1	2
0	2	1	2	1

2	0	0	0	1
0	2	0	1	1
1	0	0	1	0
0	2	0	1	1
0	1	0	2	0
1	0	1	0	1
0	2	0	0	1
0	0	1	0	0
0	3	1	1	2

1	0	1	1	1
0	0	2	1	1
0	0	1	2	2
1	0	1	0	1
0	0	1	1	1
0	0	1	2	2
2	0	3	3	3
0	0	1	0	0
0	0	0	3	1

0	2	1	0	1
0	0	0	0	3
0	3	0	0	0
3	0	0	0	0
3	0	2	2	2
1	0	2	0	2
0	0	1	0	0
1	0	1	1	1
1	0	1	2	2

0	0	1	0	0
0	0	1	0	1
3	0	0	0	2
0	0	3	3	3
1	0	1	1	1
1	0	2	3	1
0	0	0	0	2
0	0	1	3	1
0	0	2	0	2

0	0	2	0	0
0	0	2	0	1
3	0	2	1	3
0	0	0	3	1
0	0	1	0	3
0	0	3	3	3
0	3	3	3	3
1	0	1	0	0
0	3	1	3	1

3	0	1	2	0
1	0	2	2	1
2	0	2	1	1
0	0	0	0	0
2	0	1	1	1
1	1	1	1	1
0	0	1	0	1
2	0	2	2	3
0	0	3	1	3

0	2	1	0	2
3	0	2	1	2
3	0	1	2	1
2	0	0	1	0
0	0	2	2	1
3	0	2	2	0
3	0	2	2	3
0	2	2	1	2
3	0	2	0	3

3	0	2	2	2
2	0	1	2	3
0	3	2	0	1
0	1	1	1	0
3	3	3	0	0
0	3	2	0	0
0	0	1	0	1
0	0	0	1	3
2	0	3	2	3

0	0	0	0	0
0	2	0	0	0
0	3	3	3	1
1	0	2	1	1
1	0	1	1	1
0	3	1	1	3
0	0	0	1	1
2	0	2	2	1
2	0	1	2	2

0	2	1	0	1
0	0	1	0	1
1	0	2	1	2
1	0	2	0	2
1	0	1	0	0
1	0	1	0	2
3	0	1	2	1
1	0	0	0	0
1	0	2	0	1

2	0	2	2	2
3	0	2	2	1
0	0	0	1	2
3	0	2	2	1
1	0	1	0	0
1	0	2	2	1
1	0	1	1	1
3	0	1	1	2
0	2	2	1	0

1	0	0	0	1
1	0	1	1	1
0	0	1	1	0
1	0	0	3	1
1	0	2	0	2
0	1	2	1	2
0	0	0	0	0
0	1	0	1	1
0	0	1	0	1

0	0	1	1	1
0	0	1	0	1
1	0	1	0	1
1	0	1	0	0
1	0	1	0	1
0	1	1	1	1
0	2	0	0	1
0	0	0	3	1
1	0	1	1	1

0	3	2	3	3
2	0	1	3	2
0	0	0	0	1
0	0	2	0	1
0	3	2	0	0
0	0	2	0	0
3	0	3	1	1
1	0	1	0	1
2	0	2	2	2

0	3	2	2	3
3	0	2	1	2
1	0	1	1	2
0	0	2	1	1
1	0	2	1	3
3	0	1	1	1
3	0	1	0	1
3	0	1	0	2
3	0	1	1	1

3	0	0	0	0
3	0	0	0	0
3	0	2	1	2
3	0	0	0	3
3	0	3	0	0
0	0	2	0	0
0	0	1	0	0
0	3	3	0	0
0	0	0	0	0

2	0	2	1	3
0	0	2	1	1
0	1	2	0	0
0	0	2	3	1
0	2	1	0	0
0	3	0	1	1
0	0	1	1	0
0	3	3	3	2
0	0	2	3	1

0	0	2	1	1
1	0	2	1	1
1	0	2	0	0
1	0	1	2	1
1	0	1	3	0
0	3	1	0	1
0	3	1	0	0
0	0	2	3	1
0	0	1	2	2

1	0	2	2	3
2	0	0	0	1
0	3	1	1	1
0	0	2	3	2
0	0	1	0	1
1	0	0	0	0
0	1	2	1	1
0	0	0	3	0
0	0	1	0	0

0	2	0	0	0
0	2	1	0	1
2	0	3	3	2
0	0	0	0	0
0	3	2	3	1
0	1	1	1	0
2	0	3	1	2
0	0	2	2	2
0	3	2	2	1

0	2	2	0	0
0	0	0	1	0
3	0	2	1	2
2	0	1	1	1
2	0	0	1	1
0	2	0	0	2
0	1	2	1	0
0	3	1	2	2
1	0	2	1	2

0	0	2	1	1
0	0	0	1	2
0	0	2	1	1
3	0	1	2	1
0	2	1	1	1
1	0	2	1	1
2	0	0	1	0
0	2	2	2	2
0	2	2	0	1

3	0	1	2	2
3	0	0	2	0
1	0	1	0	1
1	0	0	1	0
1	0	1	2	1
0	1	2	1	1
3	0	2	1	2
0	2	2	1	1
1	0	1	0	0

0	0	1	1	1
0	2	0	0	0
2	0	0	0	2
0	0	1	1	1
1	0	2	2	1
0	2	0	1	1
0	0	0	0	0
0	0	2	1	1
3	0	1	1	1

0	0	2	2	1
0	3	0	1	2
3	0	1	1	1
0	0	1	0	1
0	0	0	0	1
3	0	2	1	2
3	0	1	1	1
0	0	3	0	1
0	0	3	0	0

0	3	0	0	1
3	0	3	3	3
3	0	3	0	3
2	0	1	0	3
3	0	0	0	0
0	3	0	1	1
0	0	0	0	2
3	0	0	3	3
0	2	2	1	2

1	0	3	1	2
1	0	2	3	2
0	0	0	0	0
0	0	0	0	1
0	0	2	1	2
1	0	2	2	2
0	0	1	1	2
2	0	1	3	2
0	0	1	1	1

1	0	1	0	3
0	1	2	0	1
1	0	1	2	0
1	0	1	2	0
0	3	1	1	1
2	0	1	0	0
1	0	1	3	3
2	0	1	3	1
0	0	0	0	0

3	0	2	1	2
0	3	1	1	2
3	0	1	2	2
0	3	2	1	1
0	0	1	2	1
0	2	0	1	1
3	0	1	2	2
3	0	1	1	2
0	1	1	1	2

2	0	1	1	0
3	0	2	2	2
0	3	1	1	1
1	0	2	0	2
0	0	0	3	1
0	3	0	2	0
3	0	2	0	1
3	0	2	2	1
2	0	1	1	0

0	3	1	2	2
0	0	1	0	1
2	0	2	1	0
3	0	0	0	0
1	0	1	0	1
2	0	2	2	1
1	0	1	1	2
0	0	0	2	0
1	0	0	1	0

0	3	1	2	1
0	1	2	2	1
1	0	1	2	0
0	2	1	2	0
0	0	2	0	2
2	0	1	2	2
0	0	2	2	2
2	0	1	0	0
1	0	1	3	2

0	2	1	2	1
0	0	2	1	1
1	0	1	0	1
0	0	0	0	0
2	0	1	1	1
3	0	2	0	2
0	0	1	2	1
3	0	2	1	1
0	0	1	1	0

2	0	2	0	1
2	0	1	0	2
0	3	2	1	0
0	2	2	2	3
2	0	1	3	3
3	0	1	2	2
0	3	1	0	0
3	0	1	1	3
0	0	2	1	3

0	3	2	1	1
1	0	1	0	0
0	0	1	0	0
3	0	0	0	0
3	0	0	0	3
3	0	3	3	3
3	0	3	0	1
2	0	3	0	2
3	0	0	0	0

0	2	2	0	0
0	0	1	0	0
0	2	2	0	1
3	0	3	2	3
0	0	0	0	0
0	2	0	0	0
1	0	0	0	0
3	0	2	3	1
0	2	1	0	1

1	0	1	1	2
1	0	1	0	0
1	0	1	0	1
1	0	2	2	0
0	1	1	1	1
0	0	1	1	0
0	2	1	0	1
2	0	1	1	1
3	0	1	1	1

0	0	1	2	1
0	3	0	1	1
0	3	0	1	1
0	0	0	2	2
0	3	1	0	1
1	0	3	2	2
0	0	0	0	1
0	0	0	0	0
0	0	2	0	3

3	0	2	0	1
0	3	0	1	2
0	3	2	1	3
0	0	1	1	2
0	3	3	0	3
0	2	2	2	2
0	1	1	0	0
0	1	1	1	1
1	0	1	1	2

0	2	0	1	1
0	0	0	1	0
0	0	2	1	2
1	0	2	1	1
3	0	1	2	1
0	2	1	1	0
0	0	1	1	3
0	3	3	2	1
0	0	2	0	2

2	0	1	3	3
0	0	1	0	1
0	2	0	0	0
2	0	1	1	0
0	1	1	2	1
0	0	0	0	2
0	0	2	1	2
3	0	1	0	2
3	0	2	0	0

2	0	3	1	2
3	0	1	2	0
0	0	1	0	1
2	0	2	1	0
0	3	2	0	0
0	0	0	0	0
3	0	0	0	1
0	0	2	0	0
1	0	1	1	2

0	1	1	1	2
0	0	1	1	0
1	0	0	0	0
1	0	2	1	1
1	0	1	0	0
1	0	1	0	1
0	0	1	1	1
1	0	1	1	1
0	1	1	1	0

0	0	3	1	3
0	0	2	2	1
2	0	0	0	0
0	0	1	1	1
0	0	2	1	2
3	0	1	1	1
0	0	1	0	2
0	0	0	0	1
0	0	0	0	2

0	0	0	0	0
1	0	0	3	2
0	0	0	0	0
0	0	0	2	3
3	0	3	0	1
0	0	3	2	2
0	3	1	1	0
0	3	1	2	2
0	0	0	0	0

0	0	0	0	0
0	0	0	0	0

@19.Intencióndesuicidarse	@20.Compromiso	@21.Placergozoexcluyendoactividadessexuales	@22.Energíafatiga	@23.Interéssexual
1	2	2	2	0
2	2	2	2	0
1	0	2	1	0
1	1	1	2	2
0	0	1	0	2
1	1	2	2	2
1	1	3	1	2
1	1	2	2	2

0	1	2	2	2
1	1	1	0	1
0	1	0	1	0
1	0	1	2	0
1	1	1	1	0
0	1	1	1	1
0	0	1	1	3
2	0	0	1	3
1	1	1	2	3

0	1	2	2	1
2	2	3	2	3
0	1	0	1	0
2	1	3	2	3
0	0	1	1	1
0	0	0	0	0
1	2	1	2	2
0	1	0	1	0
0	1	0	2	0

2	1	0	2	2
1	1	1	1	2
0	2	2	2	0
0	2	2	2	0
1	1	1	1	0
0	1	1	1	0
0	0	2	1	0
0	0	0	0	0
0	1	0	2	0

0	0	0	0	0
0	0	1	2	0
0	0	0	1	3
0	1	0	1	2
0	0	0	1	0
0	0	0	1	1
0	1	0	1	0
0	1	0	1	0
1	1	0	1	1

0	2	2	2	0
0	2	1	1	2
1	1	1	2	0
1	1	0	2	3
1	1	0	1	2
1	1	2	2	3
1	3	3	2	3
1	1	0	1	0
0	0	1	2	3

1	0	2	2	3
0	1	0	2	3
0	0	0	0	2
0	0	0	2	2
0	3	2	2	3
0	2	1	2	3
0	0	0	0	1
0	0	0	0	2
2	2	1	2	1

0	0	3	1	3
0	0	0	1	2
1	2	0	1	3
0	0	2	1	0
0	1	1	1	0
0	1	2	2	0
0	1	0	2	3
1	1	0	1	0
0	2	2	2	3

0	0	1	2	3
0	0	0	1	3
3	3	3	0	3
2	1	3	1	3
0	2	0	1	3
3	3	3	3	3
2	0	3	2	3
0	3	3	2	3
1	0	0	1	3

1	2	2	2	3
1	2	1	2	3
1	2	2	2	3
0	1	1	1	0
2	2	1	2	0
1	1	0	1	2
0	1	0	1	3
2	2	2	2	0
1	2	2	2	3

0	2	1	1	0
2	3	3	2	3
1	2	2	2	1
0	1	1	2	0
1	1	1	2	1
0	1	1	1	0
1	3	2	2	1
1	1	1	1	0
1	3	3	2	0

0	1	1	1	1
2	2	2	0	2
0	2	3	2	2
0	0	1	2	1
1	3	3	2	0
1	3	2	2	0
0	0	0	0	0
2	0	2	1	3
2	3	3	2	3

0	0	0	1	0
0	0	0	0	0
1	2	0	2	1
1	2	0	2	1
1	1	0	1	0
1	3	1	2	3
1	0	1	1	2
1	1	1	2	2
1	1	0	1	2

0	2	1	2	3
0	2	1	1	1
1	2	2	2	0
1	0	1	1	0
1	1	1	1	0
1	0	1	1	0
1	2	2	2	0
0	1	1	1	0
0	2	2	2	0

1	2	3	2	2
1	2	1	2	3
1	1	3	2	2
1	2	2	1	2
0	1	1	0	2
1	0	0	1	0
0	1	0	1	0
1	2	1	1	2
0	1	0	2	0

0	1	1	1	0
2	2	1	2	1
0	1	0	1	0
0	0	0	2	3
0	2	1	2	0
0	1	0	2	1
0	0	0	1	0
0	0	0	1	0
0	1	0	1	2

1	1	0	2	2
1	1	1	2	1
1	1	1	1	0
0	0	1	1	1
0	1	0	1	0
1	0	1	1	2
2	1	1	2	3
2	1	2	1	1
1	1	1	1	3

0	2	2	2	0
0	1	0	2	3
0	1	2	0	3
0	0	0	0	0
0	1	2	2	0
0	3	0	2	2
0	2	2	3	3
0	1	1	1	0
2	3	2	1	0

1	2	2	2	0
0	2	2	1	0
1	2	1	2	1
1	1	1	2	2
0	1	1	1	1
0	1	1	1	0
1	1	1	1	0
2	2	1	1	0
1	1	1	1	0

0	0	0	1	0
0	3	1	3	0
2	2	1	2	3
1	3	0	3	3
0	2	3	3	3
0	3	1	3	0
0	3	1	3	0
0	2	1	3	0
0	0	0	0	3

3	2	2	2	3
1	2	1	2	2
0	0	2	1	3
1	2	1	2	1
0	0	0	1	3
1	1	0	1	2
1	0	0	1	0
3	2	3	3	1
2	1	0	1	0

1	1	3	2	3
1	2	1	1	3
0	0	0	1	0
2	3	2	1	0
0	1	2	1	3
0	1	2	1	2
0	1	2	1	2
0	1	2	2	3
1	1	2	2	0

2	2	2	2	0
0	0	3	2	0
0	0	0	1	2
0	0	0	0	3
0	2	1	1	2
0	0	1	1	2
0	0	0	1	2
0	3	3	3	2
1	0	0	1	3

0	0	0	1	0
0	3	2	1	2
3	1	3	1	2
0	0	0	0	0
2	0	1	2	3
0	1	1	2	2
1	2	3	3	3
0	2	2	2	0
0	1	0	1	2

0	1	1	1	0
0	0	0	0	0
2	3	3	2	0
1	2	2	1	2
1	0	1	0	2
0	0	0	2	0
0	1	0	1	0
2	1	2	1	0
2	2	2	1	3

0	1	1	2	0
1	1	0	1	2
0	0	1	1	2
1	0	0	1	0
0	2	0	2	0
0	1	0	1	0
0	0	1	1	2
2	2	2	1	0
0	2	1	2	0

0	1	0	1	0
1	0	0	1	0
0	2	2	1	2
0	1	1	1	1
0	3	2	2	2
1	1	1	2	2
1	1	0	2	0
2	1	2	1	2
0	1	0	1	0

0	1	1	2	2
0	0	0	1	2
0	2	1	2	0
2	1	2	2	3
0	1	1	1	0
0	1	1	1	0
0	0	1	1	2
0	1	1	1	0
0	1	1	1	2

0	2	2	0	0
0	2	2	2	2
1	2	1	2	1
1	1	1	1	2
0	1	1	0	2
0	2	1	2	2
1	1	1	1	0
0	2	0	3	3
0	2	3	2	0

0	0	0	0	0
1	3	3	3	3
3	3	3	3	3
0	3	3	2	3
0	0	0	0	3
1	1	0	3	3
1	3	3	3	3
2	3	3	3	3
0	2	1	2	1

1	2	1	2	3
2	2	2	2	2
0	1	0	1	2
0	2	1	2	3
0	2	1	2	3
2	2	1	2	3
1	2	1	2	3
2	2	2	2	3
1	1	1	2	3

1	1	2	1	3
0	1	1	1	0
0	0	0	1	3
0	0	0	1	3
0	1	2	1	1
0	0	0	1	2
0	2	1	1	3
0	1	2	1	3
0	0	0	0	0

0	2	2	2	3
0	2	2	2	2
2	1	1	2	1
1	1	1	2	2
1	1	1	1	2
1	1	1	0	2
2	2	1	2	2
1	1	1	1	0
1	2	2	2	0

1	1	1	1	0
1	2	2	2	2
0	1	1	1	0
0	3	1	2	0
0	0	0	1	2
0	1	1	0	0
0	1	1	1	2
2	2	1	2	0
0	2	0	2	1

1	1	1	1	0
1	1	0	1	0
2	1	0	0	0
0	0	0	1	0
0	0	0	1	1
2	2	2	2	0
1	1	0	1	1
0	0	0	2	3
0	1	2	1	0

1	0	0	2	0
0	0	1	1	0
0	1	0	1	0
0	0	0	1	3
0	2	1	0	0
3	2	2	2	0
2	2	2	2	2
0	1	0	1	0
1	1	2	2	0

0	0	0	1	0
0	1	0	2	0
1	1	1	1	0
0	0	0	0	0
1	1	1	1	3
1	2	2	2	2
1	0	1	0	3
1	2	2	2	3
0	1	2	1	1

0	2	2	1	0
0	2	2	2	2
1	0	0	0	0
1	2	2	2	0
2	1	2	2	0
1	2	2	2	3
0	2	2	2	3
1	3	3	2	0
0	1	0	1	0

0	1	1	1	2
0	2	1	2	0
0	1	1	1	2
0	3	3	3	3
0	3	3	2	3
0	3	3	3	3
0	3	3	3	3
0	3	2	3	1
0	3	3	3	3

0	0	0	0	3
0	0	0	0	2
1	3	3	2	3
2	3	3	3	3
1	1	0	2	3
0	0	0	1	3
0	0	0	1	2
2	3	2	1	2
2	1	1	1	3

2	2	2	1	3
0	0	0	1	0
0	0	1	1	2
0	1	0	2	3
1	0	1	1	3
0	1	0	1	2
0	2	2	1	0
1	1	1	2	0
0	2	1	2	0

0	0	0	0	2
1	1	1	2	2
0	1	1	1	0
2	2	1	2	0
0	3	1	2	3
2	3	3	3	3
0	0	0	0	3
0	0	0	0	0
0	3	2	3	3

2	0	1	0	3
0	3	1	2	3
0	0	0	1	0
0	3	3	3	3
0	3	3	3	3
2	2	1	2	2
0	0	0	0	0
0	0	0	2	2
1	1	1	1	3

1	2	1	0	1
0	0	0	0	0
1	2	2	2	1
1	1	1	1	0
2	2	2	1	3
0	1	1	1	0
1	1	3	1	1
1	1	3	2	3
0	3	0	2	2

0	0	3	3	3
0	0	0	2	1
2	0	0	0	1
0	0	0	2	2
3	0	0	1	1
0	0	0	0	0
1	2	2	2	3
1	3	2	2	3
0	0	0	1	3

0	2	2	1	3
0	1	0	0	0
0	1	1	0	1
1	1	1	1	3
0	1	2	1	0
0	0	0	0	2
0	1	2	2	0
0	0	0	0	1
0	1	0	1	3

0	2	1	2	1
0	1	2	2	3
0	1	0	0	3
1	1	0	2	3
0	1	0	1	0
1	1	0	1	0
1	1	0	1	3
0	1	1	2	2
0	3	1	1	0

0	3	3	3	3
0	1	0	2	3
0	0	0	1	0
0	0	1	0	3
0	3	3	3	3
1	3	1	3	3
2	3	2	3	0
0	0	0	0	3
0	2	1	2	0

0	2	2	2	3
1	1	2	1	3
0	0	0	1	0
2	3	2	0	3
1	1	1	2	0
2	2	2	2	3
0	2	1	2	2
1	2	1	1	2
0	0	0	0	0

0	0	0	0	0
0	0	0	0	0

@24.Disminucióndelavelocidadpsicomotora	@25.Agitaciónpsicomotora	@26.Quejassomáticas	@27.Estimulodelsistemanerviososimpático	@28.Funcióngastrointestinal
2	0	1	1	2
1	0	1	1	1
1	0	2	1	0
0	0	2	1	0
1	0	1	1	1
1	0	0	0	0
0	0	1	1	1
1	0	1	2	0

0	0	2	2	0
0	0	0	2	0
1	0	1	1	0
0	2	2	2	0
0	0	1	1	0
0	0	1	0	1
1	0	1	1	0
1	0	0	2	1
1	0	2	1	0

1	0	3	2	2
1	0	3	1	0
0	0	1	2	0
1	0	2	1	2
0	0	2	2	0
0	0	1	1	0
1	0	2	0	1
1	0	0	0	0
0	0	1	0	0

0	0	2	1	2
0	0	0	1	1
1	0	1	1	0
0	0	2	2	0
1	0	2	1	1
1	0	1	1	2
1	0	2	2	1
1	0	2	0	1
0	0	2	0	1

0	0	2	1	0
0	0	1	1	1
1	0	2	1	0
1	0	2	2	2
1	0	1	0	2
1	0	2	1	1
0	0	1	2	0
0	0	0	2	0
1	0	2	2	1

0	0	1	2	1
0	0	0	1	1
0	0	2	2	1
0	0	1	2	2
0	0	1	1	2
1	0	2	2	3
0	0	0	2	0
0	0	3	3	0
0	0	3	1	1

0	0	1	1	0
0	0	3	0	3
0	0	1	0	0
0	0	0	0	2
2	0	2	1	0
2	0	1	0	0
0	0	0	0	0
0	0	1	1	0
2	0	2	2	1

0	0	0	0	3
0	0	1	1	0
1	0	1	2	0
1	0	1	0	0
0	0	1	2	2
1	0	1	1	0
0	0	0	0	0
0	0	1	1	2
0	0	3	0	0

0	0	1	2	2
0	0	1	1	2
0	0	1	0	2
1	0	1	0	0
0	0	0	0	2
0	0	3	2	3
1	0	3	1	3
0	0	1	2	0
0	0	2	3	0

0	0	0	0	0
2	0	2	2	2
0	0	2	2	2
0	0	1	1	1
0	0	1	1	0
0	0	2	2	2
0	0	1	1	0
1	0	1	2	1
1	0	2	2	1

1	0	2	1	0
1	0	1	2	0
1	0	1	1	1
1	0	2	3	3
2	0	1	2	3
1	0	1	0	0
2	0	1	2	1
1	0	1	2	1
2	0	1	2	0

1	0	1	1	0
1	0	1	2	1
0	0	2	2	2
0	0	1	1	0
0	0	2	2	0
1	0	2	3	3
1	0	1	2	0
0	0	1	1	0
1	2	2	2	1

0	0	0	0	0
0	0	0	1	0
1	0	2	1	0
1	0	2	2	2
0	0	2	1	0
1	0	1	1	0
0	0	0	0	0
1	0	1	2	1
0	0	1	1	1

1	0	2	2	3
0	0	1	1	2
0	2	1	0	3
0	0	2	2	2
0	0	3	1	0
0	0	1	2	0
0	0	1	2	1
2	0	1	1	0
1	0	0	1	2

1	0	1	1	0
1	0	2	1	2
0	0	0	2	0
1	0	2	2	3
0	0	2	2	1
0	0	2	2	2
2	0	2	2	2
0	0	2	2	2
0	0	1	1	0

1	0	1	0	0
2	0	1	1	0
0	0	2	1	0
1	0	2	2	2
1	2	2	2	0
0	0	3	2	1
0	0	1	1	2
0	0	0	0	0
0	0	1	1	0

0	0	1	2	3
0	0	2	2	0
1	0	1	1	0
0	0	1	1	0
0	0	1	1	0
0	0	1	1	0
0	0	2	1	0
0	0	1	1	0
0	0	1	0	1

0	0	1	2	1
1	0	1	2	1
0	0	1	0	1
1	0	2	0	0
0	0	1	1	0
0	0	2	0	1
1	0	2	1	1
0	0	1	2	1
1	0	1	1	1

1	0	2	2	2
1	0	1	0	1
1	0	1	0	1
1	0	1	1	0
1	0	1	1	1
1	0	1	1	1
1	0	2	2	2
2	0	1	1	0
1	0	2	2	1

0	0	3	2	0
0	0	2	0	0
1	0	3	2	1
0	0	2	1	1
0	0	1	1	0
0	0	2	2	0
0	0	1	1	0
0	0	1	3	2
0	0	2	2	3

1	0	2	2	2
1	0	1	1	0
1	0	1	0	0
1	0	2	2	2
0	0	2	1	0
0	0	1	2	0
1	0	1	1	0
1	0	2	2	2
1	0	1	1	0

0	0	0	2	2
1	0	1	1	2
0	0	1	1	1
0	0	1	1	1
0	0	1	1	2
0	0	1	1	0
1	0	1	1	0
1	0	1	1	2
0	0	1	1	0

1	0	2	3	2
1	0	1	1	0
0	0	1	2	2
0	0	0	0	0
0	0	1	2	0
1	0	2	2	0
0	2	1	0	0
0	0	2	1	1
1	0	1	0	0

0	0	1	0	0
0	0	1	1	0
0	0	0	0	1
0	0	1	0	0
1	0	2	2	2
1	0	2	1	1
2	0	2	1	2
1	0	1	1	0
1	0	1	1	1

1	0	2	1	0
0	0	1	1	0
1	0	2	2	2
0	0	1	1	1
1	0	1	1	2
1	0	3	1	0
0	0	2	2	0
1	0	2	1	0
1	0	2	1	2

1	0	3	0	0
0	0	3	1	0
1	0	2	1	0
0	0	1	0	0
1	0	2	2	1
0	0	2	2	2
0	2	2	1	1
2	0	2	2	2
2	0	2	2	1

0	2	1	2	2
0	2	1	1	0
0	0	1	0	1
2	0	1	1	0
2	0	2	1	2
1	0	1	1	1
0	0	1	0	0
0	0	1	1	0
0	0	2	2	2

0	0	2	2	1
0	0	2	1	1
0	0	2	2	0
0	0	1	2	0
1	0	0	0	0
0	0	1	1	2
0	0	2	1	1
0	0	0	1	0
0	0	2	2	1

2	0	1	1	1
2	0	2	2	2
1	0	2	2	2
0	0	1	2	0
0	0	0	0	0
2	0	1	1	0
0	0	1	1	1
0	0	2	2	1
0	0	2	1	1

0	0	1	0	0
0	0	3	3	3
0	0	2	2	0
0	0	3	3	3
0	0	0	2	1
0	0	2	2	2
0	0	3	1	1
1	0	3	2	0
1	0	2	1	2

1	0	2	2	0
2	0	2	2	0
0	0	1	2	0
1	0	2	2	0
0	0	2	2	2
0	0	2	2	0
0	0	2	0	0
1	0	2	3	0
0	0	2	2	0

0	0	2	1	0
1	0	1	1	1
0	0	1	0	0
0	0	1	0	0
0	0	1	1	0
0	0	1	1	0
1	0	1	1	0
1	0	1	1	0
0	0	0	0	0

0	0	1	2	2
1	0	2	2	1
1	0	1	1	1
1	0	1	1	1
1	0	1	0	1
0	0	1	1	0
1	0	1	1	1
1	0	1	1	0
1	0	1	1	1

1	0	1	0	1
1	0	2	2	0
0	0	2	1	0
2	0	2	2	2
1	0	0	1	0
1	0	0	2	2
0	2	2	2	1
2	0	2	2	1
1	0	2	2	3

1	0	1	2	2
1	0	1	1	0
2	0	2	1	0
1	0	1	2	1
0	2	1	2	2
1	0	2	1	3
2	0	1	1	1
0	0	2	1	2
0	0	2	0	1

1	0	2	0	1
1	0	2	1	0
1	0	2	1	1
1	0	1	2	2
2	0	1	0	0
2	0	2	1	2
2	0	2	2	2
0	0	1	1	0
2	0	1	2	0

2	0	2	1	0
1	0	2	1	0
1	0	1	1	0
0	0	0	0	0
1	0	2	1	0
2	0	1	0	0
0	0	1	1	0
1	0	2	1	2
1	0	2	1	0

0	0	0	1	0
1	0	1	1	0
2	0	2	1	0
1	0	2	2	0
1	0	2	1	0
1	0	2	2	0
1	0	2	2	0
1	0	2	0	0
1	0	1	0	0

0	0	1	1	0
0	0	2	1	0
0	0	0	1	0
0	0	2	2	3
2	0	2	2	0
1	0	0	2	0
1	0	2	2	0
0	0	2	2	0
1	0	3	1	1

0	0	3	2	0
0	0	1	0	0
0	0	2	1	2
2	0	2	0	0
0	0	0	0	0
0	0	0	1	0
0	0	1	0	0
1	0	1	0	0
1	0	1	1	0

0	0	1	1	2
0	0	1	0	0
0	0	1	1	1
0	0	1	1	1
0	0	1	0	0
0	0	1	0	0
1	0	2	2	2
2	0	1	0	1
1	0	0	0	0

0	0	1	1	0
1	0	2	1	0
1	0	1	1	1
0	0	2	2	0
0	0	3	3	1
1	0	1	1	0
0	0	1	0	0
0	0	0	0	2
2	0	0	2	0

1	0	2	0	0
0	0	1	0	0
1	0	2	2	0
2	0	2	0	2
0	0	2	1	1
1	0	1	2	1
0	0	1	1	1
0	0	1	1	2
0	0	2	2	1

0	0	1	2	1
0	0	0	0	0
0	0	2	2	1
0	0	1	1	1
1	0	2	2	0
1	0	1	1	0
0	0	1	1	2
1	0	3	2	2
1	0	1	1	0

1	0	0	1	3
1	0	1	1	0
0	0	0	2	0
0	0	1	2	1
0	0	1	0	0
0	0	0	0	0
1	0	2	2	0
0	0	0	0	0
0	0	0	1	1

0	0	2	1	2
0	0	2	0	0
0	0	3	2	0
1	0	2	2	0
1	0	2	2	0
0	0	0	0	0
0	0	1	0	0
0	2	0	0	0
0	0	1	1	0

0	0	1	1	1
1	0	1	0	1
0	0	0	0	0
1	0	2	2	2
0	0	0	0	0
0	0	1	1	1
0	0	1	1	0
0	0	1	1	0
1	0	1	1	0

0	0	3	2	2
0	0	2	2	2
0	0	2	0	1
0	0	1	1	2
0	0	2	3	3
0	0	1	2	2
0	0	2	1	1
0	0	1	0	0
0	0	1	1	0

0	0	0	1	0
1	0	1	2	1
1	0	1	0	0
0	0	2	2	3
2	0	1	1	2
1	0	2	2	0
1	0	0	0	0
0	0	1	2	1
0	0	0	0	0

0	0	0	0	0
0	0	0	0	0

@29.Sensibilidadinterpersonal	@30.Desánimoparalizantedeenergíafísica	Númeroficha	TOTAL_A1A3SI	total_patol_medicas
0	2	1	8	1
1	2	2	8	3
1	1	3	6	1
1	2	4	9	0
0	1	5	9	2
2	1	6	8	0
1	1	7	0	2
0	2	8	8	0

1	2	9	6	0
2	1	10	7	0
0	2	11	6	2
2	2	12	8	0
0	1	13	5	1
0	1	14	5	0
1	1	15	0	4
1	1	16	6	2
2	2	17	7	0

2	2	18	8	0
0	3	19	9	1
0	0	20	0	1
0	1	21	8	0
1	1	22	8	1
0	0	23	8	2
1	2	24	8	1
0	0	25	0	2
1	1	26	7	2

1	2	27	8	1
1	1	28	8	0
0	1	29	5	0
2	3	30	8	3
2	1	31	7	3
0	2	32	5	0
0	2	33	5	1
2	1	34	8	0
1	2	35	7	0

0	1	36	4	0
2	1	37	5	1
1	1	38	5	0
2	1	39	5	1
0	1	40	5	0
0	1	41	6	0
1	1	42	4	0
1	0	43	0	0
0	2	44	4	3

0	2	45	6	1
1	1	46	8	0
3	2	47	6	2
1	1	48	6	0
1	0	49	8	0
1	1	50	6	0
2	2	51	5	0
2	2	52	6	0
2	2	53	4	1

2	2	54	6	2
0	3	55	5	1
0	0	56	0	3
0	2	57	0	0
1	2	58	8	0
0	2	59	4	3
0	0	60	4	0
0	1	61	0	3
2	2	62	9	0

0	3	63	0	2
0	0	64	5	2
0	1	65	7	5
1	1	66	4	0
0	1	67	8	2
3	2	68	8	0
0	1	69	5	2
3	1	70	8	2
0	1	71	5	3

0	1	72	0	1
2	1	73	1	3
1	0	74	8	0
0	1	75	6	1
1	2	76	2	0
3	3	77	8	1
1	3	78	9	1
3	3	79	5	3
1	1	80	6	3

0	2	81	3	3
1	2	82	9	2
2	3	83	6	0
0	1	84	4	2
2	2	85	8	1
2	2	86	8	0
0	1	87	6	1
2	2	88	9	1
0	2	89	9	2

0	2	90	7	0
0	3	91	9	0
1	2	92	8	0
3	3	93	6	1
2	3	94	9	0
0	0	95	8	0
2	3	96	9	0
1	2	97	8	2
1	2	98	7	3

2	2	99	8	0
2	0	100	9	0
0	2	101	7	0
0	0	102	6	1
3	3	103	6	2
2	3	104	9	0
1	1	105	0	1
0	1	106	5	0
3	3	107	9	0

0	0	108	1	0
1	1	109	0	1
1	2	110	6	1
0	2	111	7	3
0	1	112	0	3
2	2	113	8	3
0	1	114	3	0
0	2	115	8	2
0	2	116	9	0

0	2	117	7	0
0	1	118	5	2
2	2	119	9	1
1	1	120	6	0
1	1	121	5	3
1	1	122	5	1
1	3	123	8	2
0	1	124	7	1
0	1	125	7	0

1	3	126	6	0
1	2	127	8	0
2	2	128	7	1
2	2	129	8	0
0	1	130	6	0
1	1	131	8	0
1	2	132	5	2
1	1	133	8	0
1	1	134	6	2

0	1	135	6	2
2	1	136	8	0
0	1	137	8	0
0	1	138	6	2
1	2	139	7	1
2	1	140	8	0
1	1	141	0	0
0	0	142	3	0
0	1	143	5	0

2	1	144	4	0
1	2	145	5	1
0	1	146	4	1
2	1	147	0	1
2	0	148	0	0
0	1	149	6	0
2	2	150	5	2
2	1	151	7	0
0	1	152	9	2

2	3	153	8	0
1	1	154	2	0
0	1	155	4	0
2	2	156	3	0
1	2	157	7	4
0	2	158	8	0
1	3	159	0	1
0	1	160	4	2
2	2	161	9	1

0	2	162	8	0
1	1	163	8	0
2	2	164	9	0
2	2	165	8	0
1	2	166	8	0
1	2	167	8	0
2	2	168	7	2
2	2	169	8	1
1	2	170	8	1

0	1	171	5	1
1	3	172	5	0
3	3	173	9	3
0	3	174	5	2
0	3	175	6	2
0	3	176	5	0
1	3	177	7	0
3	3	178	7	0
3	1	179	4	1

2	3	181	8	0
0	2	183	6	0
1	1	184	0	2
1	2	185	7	1
0	1	186	0	2
1	2	187	6	2
0	1	188	7	1
3	3	189	8	0
1	2	190	7	1

2	3	191	9	0
0	2	192	9	0
0	1	193	6	2
2	1	194	7	1
0	1	195	6	3
1	2	196	6	0
1	1	197	7	0
1	2	198	8	4
2	1	199	8	0

2	3	200	9	4
0	1	201	9	0
1	2	202	7	0
1	0	203	0	1
0	2	204	6	2
1	2	205	7	2
0	0	206	0	0
0	3	207	9	1
1	0	208	5	0

0	0	209	0	0
3	2	210	0	0
3	2	211	8	0
0	0	212	0	1
1	2	213	8	0
1	1	214	7	1
2	2	215	8	1
1	1	216	8	0
2	1	217	6	1

0	1	218	6	0
0	1	219	0	3
2	2	220	9	0
3	1	221	8	0
1	0	222	4	3
0	1	223	6	5
0	1	224	4	2
1	1	225	9	1
2	2	226	8	0

0	2	227	5	2
2	1	228	7	2
1	1	229	5	1
1	1	230	8	0
0	1	231	8	0
1	1	232	6	2
0	2	233	7	1
2	1	234	9	0
0	1	235	8	3

1	1	236	7	0
0	0	237	8	0
0	1	238	5	0
1	1	239	6	2
2	2	240	8	0
3	2	241	7	1
1	1	242	6	2
2	0	243	5	2
1	1	244	4	2

0	1	245	6	1
0	0	246	4	1
0	1	247	5	2
0	1	248	6	1
2	0	249	6	0
1	1	250	5	0
0	1	251	3	3
0	1	252	7	0
2	1	253	8	0

0	0	254	8	0
1	2	255	7	1
1	2	256	8	2
0	0	257	5	3
2	2	258	3	1
1	2	259	8	3
1	2	260	8	0
0	2	261	5	2
0	2	262	6	0

0	0	263	2	0
0	0	264	7	0
0	3	265	8	0
1	3	266	6	0
3	0	267	4	2
0	3	268	5	1
0	3	269	5	3
0	3	270	8	0
2	2	271	8	1

1	3	272	8	3
2	3	273	9	0
0	1	274	0	0
0	2	275	3	2
0	2	276	5	2
2	3	277	7	2
1	3	278	4	2
2	3	279	7	1
0	2	280	7	3

1	3	281	8	4
2	1	282	8	0
0	1	283	6	2
0	1	284	6	5
1	1	285	8	0
0	1	286	7	2
0	1	287	8	0
0	1	288	9	2
0	0	289	0	1

3	2	290	9	0
1	2	291	7	2
1	2	292	9	0
2	2	293	8	0
2	0	294	7	1
1	2	295	5	1
1	2	296	8	0
0	2	297	7	4
0	1	298	8	1

1	0	299	8	0
2	2	300	8	0
2	1	301	6	0
2	2	302	8	4
0	1	303	7	1
0	1	304	7	4
2	1	305	6	2
2	2	306	9	0
2	1	307	5	1

1	1	308	7	0
0	1	309	5	0
2	2	310	5	5
0	0	311	4	2
1	1	312	5	2
0	1	313	7	1
0	1	314	6	1
0	0	315	5	4
0	1	316	1	1

0	2	317	7	1
2	2	318	4	0
0	1	319	6	4
0	1	320	6	1
1	1	321	5	1
2	2	322	9	0
2	2	323	9	0
0	1	324	5	0
0	2	325	9	1

0	1	326	7	1
0	2	331	5	2
0	1	332	8	4
1	0	333	0	0
0	1	334	7	0
0	2	335	8	1
0	0	336	4	0
2	2	337	8	0
1	1	338	6	0

0	1	339	7	3
0	2	340	6	3
2	0	341	7	1
2	2	342	8	0
1	2	343	9	0
2	1	344	8	0
0	2	345	7	0
2	2	346	8	2
2	1	347	4	0

1	1	348	7	0
1	2	349	6	0
0	1	350	1	2
0	3	351	5	1
0	3	352	5	1
0	3	353	7	2
0	3	354	7	3
0	3	355	6	2
2	3	356	5	0

0	0	357	0	2
1	0	358	0	1
0	2	359	8	2
0	3	360	9	0
0	1	361	0	3
0	1	371	0	1
1	0	372	0	1
2	3	373	9	0
2	1	374	9	0

1	1	375	9	0
0	1	376	8	2
0	2	377	8	0
2	2	378	8	2
2	1	379	0	2
1	1	380	4	0
1	0	381	7	1
2	2	382	9	1
2	2	383	8	0

1	0	384	7	0
0	3	385	7	0
2	2	386	7	0
1	2	387	8	5
2	2	391	7	3
2	3	392	9	0
0	0	393	3	3
0	0	394	2	1
0	3	395	7	3

1	0	396	6	0
0	2	397	6	0
0	0	398	6	0
0	3	399	6	2
0	3	400	6	1
3	2	401	9	3
0	0	402	0	2
0	1	403	5	1
0	1	404	5	3

0	1	410	7	0
0	0	411	0	0
1	2	412	9	0
2	1	413	5	3
2	1	414	9	1
2	1	415	5	4
3	1	416	7	0
2	3	422	9	0
2	1	423	8	0

0	1	424	6	2
0	1	425	4	3
1	0	426	6	1
1	3	427	9	0
1	1	428	7	0
1	0	429	3	0
2	3	430	8	3
0	2	431	9	0
0	1	432	8	0

1	1	433	8	3
0	1	434	9	0
0	0	435	3	2
3	0	436	9	3
2	2	437	7	1
0	0	438	3	1
0	1	439	7	0
0	1	440	6	0
0	1	461	7	0

1	1	462	7	0
1	1	463	8	0
0	0	464	0	1
3	3	465	9	2
1	1	466	7	1
1	1	467	0	0
1	1	468	1	2
1	1	469	7	1
1	2	470	5	0

0	1	471	6	2
2	1	472	6	0
0	0	473	4	0
0	0	474	5	2
1	3	475	5	2
0	2	476	7	1
0	0	477	6	0
0	0	478	3	1
0	2	479	4	3

0	3	480	3	2
2	3	501	8	0
2	1	502	5	1
0	1	561	1	1
1	2	562	9	0
2	3	600	8	3
1	2	700	6	0
2	1	701	7	0
0	0	702	7	1

0	0	0	0	0
0	0	0	0	6

total_comorbilidad	TOTAL_FOBIA_SOCIAL	FOBIA_SOCIAL_total	RIESGOSUICIO_1DELASRESPUESTAS	PANICO_INESP
4	0	0	0	0
8	0	0	1	1
4	0	0	0	0
3	0	0	0	0
7	0	0	0	1
6	0	0	1	0
5	0	0	1	1
4	0	0	0	0

6	0	3	0	0
7	0	0	1	0
2	0	0	0	0
6	0	0	1	0
4	0	0	1	0
3	0	0	0	0
3	0	4	0	0
7	0	0	1	0
4	0	0	1	0

10	0	0	0	1
5	0	0	1	0
2	0	0	0	0
8	0	0	1	0
6	0	0	1	0
5	0	1	0	0
5	0	0	1	0
1	0	0	0	0
2	0	0	0	0

10	0	3	1	1
9	0	0	0	1
2	0	0	0	0
7	0	0	1	0
4	0	0	1	0
4	0	0	0	0
3	0	0	0	0
4	0	0	0	0
9	0	0	1	0

4	0	0	0	0
4	0	0	0	0
4	0	0	1	0
3	0	0	0	0
2	0	0	0	0
3	0	0	0	0
5	0	0	0	1
1	0	4	0	0
4	0	0	1	0

3	0	0	0	0
4	0	0	0	0
6	0	0	0	0
6	0	0	0	0
3	0	1	1	0
4	0	0	1	0
8	0	0	1	1
9	0	0	1	1
5	0	0	1	1

9	0	1	0	1
3	0	0	0	1
3	0	0	0	0
2	0	0	0	0
2	0	0	0	0
3	0	1	0	0
4	0	4	0	0
3	0	4	0	0
8	0	0	1	1

2	0	0	0	0
5	0	0	1	0
6	0	0	1	0
4	0	4	0	0
2	0	1	0	0
8	0	2	1	0
2	0	1	0	0
10	0	0	1	0
1	0	0	0	0

3	0	0	1	0
3	0	0	1	0
6	0	0	1	0
4	0	0	1	0
6	0	0	1	0
13	0	4	1	1
5	0	0	1	0
2	0	0	0	0
5	0	0	1	0

4	0	0	1	0
6	0	1	1	0
9	0	0	1	0
1	0	0	0	0
6	0	0	1	0
8	0	0	1	1
2	0	0	0	0
8	0	0	1	0
7	0	0	1	0

4	0	0	1	0
7	0	0	1	1
3	0	0	0	0
2	0	0	0	0
9	0	0	1	0
3	0	0	0	0
4	0	0	1	0
6	0	3	1	0
9	0	0	1	1

6	0	1	0	1
7	0	0	1	0
2	0	0	0	0
6	0	4	0	1
6	0	0	1	0
9	0	4	1	1
1	0	0	0	0
8	0	0	1	0
15	0	3	1	1

0	0	1	0	0
2	0	0	0	1
6	0	0	0	0
13	0	3	1	1
3	0	0	1	0
8	0	0	1	0
2	0	0	0	0
5	0	0	0	0
9	0	0	1	0

6	0	0	0	0
4	0	0	0	0
8	0	0	1	0
7	0	3	1	1
3	0	0	0	0
4	0	0	0	0
6	0	0	1	0
9	0	0	1	0
5	0	0	0	0

4	0	0	1	0
9	0	4	1	0
7	0	0	1	0
8	0	0	1	0
6	0	0	0	1
11	0	4	1	0
4	0	0	0	0
2	0	0	0	0
2	0	0	0	0

7	0	0	1	0
5	0	0	1	0
2	0	0	0	0
4	0	0	0	0
6	0	0	1	0
5	0	4	0	0
1	0	0	0	0
4	0	0	0	1
4	0	0	0	0

2	0	4	0	0
3	0	1	1	0
6	0	0	1	1
4	0	4	0	1
2	0	4	0	0
6	0	4	1	0
4	0	0	1	0
8	0	4	1	1
6	0	0	1	0

3	0	0	0	0
2	0	0	0	0
1	0	0	0	0
2	0	0	0	0
2	0	0	0	0
6	0	0	0	1
4	0	0	1	0
0	0	0	0	0
9	0	4	1	0

9	0	4	1	1
3	0	1	0	0
5	0	0	1	0
9	0	0	1	1
4	0	0	1	0
2	0	0	0	0
3	0	0	0	0
5	0	0	1	0
5	0	0	1	0

2	0	0	0	0
3	0	2	0	0
8	0	4	1	1
5	0	0	1	0
6	0	0	1	0
7	0	0	1	1
1	0	0	0	0
7	0	0	0	1
7	0	0	0	1

7	0	0	1	0
5	0	0	1	0
0	0	0	0	0
8	0	0	1	1
3	0	0	0	1
4	0	0	1	0
4	0	0	1	0
8	0	4	1	0
6	0	0	1	0

6	0	0	1	1
3	0	0	1	0
6	0	4	0	1
6	0	0	1	0
3	0	0	0	0
5	0	0	0	0
5	0	0	0	0
5	0	0	0	0
11	0	0	1	0

11	0	0	1	0
5	0	0	0	0
7	0	0	1	1
3	0	0	0	0
5	0	0	1	0
1	0	0	0	0
1	0	0	0	0
5	0	0	0	1
5	0	1	0	0

1	0	0	0	0
2	0	4	0	0
8	0	4	1	0
0	0	0	0	0
13	0	2	1	1
9	0	0	1	1
7	0	4	1	0
6	0	0	1	0
11	0	4	1	0

4	0	4	0	0
1	0	0	0	0
15	0	4	1	1
4	0	0	0	0
3	0	0	1	0
3	0	0	0	0
3	0	0	0	0
10	0	0	1	1
9	0	0	1	0

3	0	0	0	0
8	0	0	1	0
6	0	0	1	0
5	0	0	1	0
4	0	0	0	0
4	0	0	1	0
6	0	0	1	0
10	0	4	1	0
5	0	0	1	0

3	0	0	0	0
5	0	2	1	0
2	0	0	0	0
4	0	0	0	0
11	0	4	1	0
5	0	4	1	0
4	0	0	1	0
6	0	4	1	0
1	0	0	0	0

4	0	0	0	0
0	0	0	0	0
5	0	0	1	0
9	0	0	1	1
2	0	0	0	0
3	0	0	0	0
1	0	0	0	0
10	0	4	1	0
2	0	0	0	0

3	0	0	0	0
5	0	1	0	1
5	0	0	0	0
7	0	0	1	1
3	0	0	0	0
4	0	0	0	0
6	0	4	1	0
4	0	1	0	0
2	0	0	0	0

0	0	0	0	0
4	0	0	1	0
5	0	0	1	0
6	0	0	0	1
1	0	0	0	0
4	0	0	1	0
7	0	0	1	1
3	0	0	1	0
5	0	0	1	0

2	0	0	1	0
5	0	0	1	0
1	0	0	0	0
0	0	0	0	0
6	0	0	1	0
6	0	0	1	0
4	0	0	1	0
10	0	0	1	1
4	0	0	1	0

7	0	0	1	0
3	0	4	0	0
3	0	0	0	0
2	0	0	0	0
5	0	0	1	0
5	0	0	0	1
6	0	0	0	0
3	0	0	1	0
0	0	0	0	0

8	0	0	1	1
8	0	0	0	1
9	0	0	1	1
8	0	4	1	0
3	0	0	0	0
4	0	0	0	0
11	0	4	1	0
4	0	4	1	0
3	0	0	0	0

4	0	0	1	0
8	0	4	0	0
7	0	0	0	0
8	0	4	0	0
8	0	2	1	0
6	0	0	1	0
7	0	0	1	0
8	0	0	1	0
7	0	0	0	0

8	0	2	1	0
6	0	3	1	0
1	0	1	0	0
3	0	0	0	0
5	0	0	0	1
3	0	0	1	0
4	0	0	1	0
3	0	0	0	0
5	0	0	1	0

5	0	2	1	0
6	0	3	1	0
5	0	0	1	0
5	0	0	0	0
2	0	0	0	0
11	0	0	1	1
8	0	3	1	1
3	0	0	1	0
7	0	0	1	1

3	0	0	0	0
4	0	0	0	0
4	0	0	1	0
2	0	0	0	0
8	0	0	1	0
7	0	0	0	1
4	0	0	1	0
2	0	0	0	0
6	0	1	0	0

6	0	0	0	0
5	0	0	1	0
8	0	1	1	1
9	0	4	0	1
10	0	0	1	0
9	0	4	1	0
5	0	4	0	0
8	0	1	1	0
5	0	3	0	0

4	0	0	0	0
4	0	0	0	0
1	0	0	0	0
1	0	0	0	0
1	0	0	0	0
4	0	0	0	1
2	0	0	0	0
3	0	0	0	0
6	0	3	1	0

4	0	0	0	1
0	0	0	0	0
6	0	0	1	0
5	0	0	1	0
1	0	0	1	0
0	0	0	0	0
0	0	0	0	0
10	0	4	1	0
5	0	0	1	0

7	0	0	1	0
1	0	0	0	0
3	0	0	0	0
3	0	0	0	0
2	0	0	0	0
2	0	0	0	0
3	0	0	0	0
4	0	4	1	0
6	0	1	1	0

3	0	0	0	0
5	0	0	1	0
9	0	0	1	1
7	0	0	1	1
8	0	4	0	0
6	0	4	1	0
1	0	0	0	0
0	0	0	0	0
3	0	0	0	0

2	0	0	1	0
2	0	0	0	0
2	0	0	0	0
3	0	0	0	0
5	0	0	0	0
11	0	4	1	0
0	0	0	0	0
2	0	0	0	0
6	0	0	1	0

4	0	0	1	0
5	0	0	0	1
8	0	4	1	1
8	0	4	1	1
11	0	4	1	1
4	0	0	0	0
9	0	3	1	0
11	0	4	0	1
3	0	1	0	0

3	0	0	0	0
3	0	1	0	0
5	0	0	1	0
6	0	1	0	1
6	0	0	1	0
1	0	3	0	0
4	0	0	0	0
5	0	1	0	0
6	0	0	0	0

6	0	0	0	0
3	0	0	0	0
2	0	0	0	0
3	0	4	0	0
4	0	3	0	0
1	0	0	0	0
3	0	0	0	0
5	0	0	0	1
2	0	0	0	0

1	0	0	0	0
2	0	1	1	0
0	0	0	0	0
6	0	0	1	0
5	0	0	1	0
1	0	0	1	0
5	0	0	1	0
6	0	0	1	0
8	0	4	0	1

5	0	0	0	0
8	0	4	1	0
0	0	0	0	0
2	0	0	0	0
4	0	0	0	1
4	0	0	1	1
5	0	0	1	0
1	0	0	0	0
0	0	0	0	0

0	0	0	0	0
7	0	0	1	0
2	0	0	0	0
6	0	0	1	1
8	0	0	1	1
7	0	0	1	0
3	0	0	0	0
10	0	0	1	1
3	0	0	0	0

0	0	0	0	0
0	0	0	0	0

TOTAL_COMORBILIDADES	v_insultado	v_humillado	v_descalificar	v_amenza
4	0	0	0	0
6	0	0	0	0
4	0	0	0	0
3	0	0	0	0
6	0	0	0	0
5	0	0	0	0
3	0	0	0	0
4	0	0	0	0

6	0	0	0	0
6	0	0	0	0
2	0	0	0	0
5	0	0	0	0
3	0	0	0	0
3	1	1	1	1
3	0	0	0	0
6	0	0	0	0
3	0	0	0	0

9	0	0	0	0
4	0	0	0	0
2	0	0	0	0
7	0	0	0	0
5	0	0	0	0
5	1	1	1	1
4	1	1	1	0
1	0	0	0	0
2	0	0	0	0

8	1	1	1	0
8	1	1	1	1
2	1	1	1	0
6	1	1	1	1
3	0	0	0	0
4	1	1	0	1
3	0	0	0	0
4	1	1	1	0
8	0	0	0	0

4	0	0	0	0
4	0	0	0	0
3	1	1	1	0
3	0	0	0	0
2	1	1	1	0
3	0	0	0	0
4	1	1	1	1
1	0	0	0	0
3	0	0	0	0

3	1	1	1	1
4	1	1	0	0
6	1	1	1	0
6	1	1	1	1
2	1	1	1	1
3	1	1	1	0
6	0	0	0	0
7	0	0	0	0
3	0	0	0	0

8	0	0	0	0
2	0	0	0	0
3	0	0	0	0
2	0	0	0	0
2	0	0	0	0
3	0	0	0	0
4	0	1	0	0
3	1	1	1	1
6	0	0	0	0

2	1	1	1	1
4	1	1	1	0
5	1	1	1	1
4	0	0	0	0
2	1	1	0	0
7	1	1	1	1
2	0	1	1	0
9	1	1	1	1
1	0	0	0	0

2	1	1	1	1
2	1	1	1	1
5	0	0	0	0
3	1	1	1	1
5	1	1	1	0
11	1	1	1	1
4	0	0	0	0
2	0	0	0	0
4	1	1	1	0

3	0	0	0	0
5	1	1	1	1
8	1	0	0	0
1	0	0	0	0
5	0	1	1	0
6	1	1	1	0
2	1	1	1	0
7	1	1	1	1
6	1	1	1	1

3	1	1	1	0
5	0	0	1	1
3	1	1	1	0
2	1	0	0	0
8	1	1	1	0
3	1	1	1	0
3	1	1	1	0
5	1	0	1	0
7	1	1	1	1

5	1	1	1	1
6	1	1	1	1
2	0	0	0	0
5	0	0	0	0
5	1	1	1	1
7	1	1	1	1
1	1	1	1	1
7	0	0	0	0
13	1	1	1	1

0	1	1	1	1
1	1	0	0	0
6	0	1	0	0
11	1	1	1	1
2	1	1	1	1
7	1	1	1	1
2	1	1	1	1
5	1	1	1	0
8	1	1	1	0

6	0	0	0	0
4	0	0	0	0
7	1	1	1	0
5	0	0	0	0
3	0	0	0	0
4	1	1	1	1
5	1	1	1	0
8	0	0	0	0
5	0	0	0	0

3	1	1	1	1
8	1	1	1	1
6	1	1	1	1
7	1	1	1	1
5	0	0	0	0
10	0	0	0	1
4	0	0	0	0
2	0	0	0	0
2	0	0	0	0

6	1	1	1	1
4	1	1	1	1
2	1	1	1	0
4	1	1	1	1
5	0	0	0	0
5	0	0	0	0
1	1	1	1	1
3	0	0	0	0
4	0	0	0	0

2	1	1	1	0
2	1	1	1	1
4	0	0	0	0
3	1	1	1	1
2	0	0	0	0
5	1	1	1	1
3	1	1	1	0
6	1	1	1	1
5	1	1	1	1

3	1	1	1	0
2	1	1	1	0
1	0	0	0	0
2	1	0	1	0
2	1	0	0	1
5	0	0	0	0
3	0	0	0	0
0	0	0	0	0
8	0	0	0	0

7	1	1	1	1
3	0	0	0	0
4	1	1	0	0
7	1	1	1	0
3	1	1	1	1
2	1	1	1	0
3	1	1	1	0
4	0	0	0	0
4	0	0	0	0

2	0	0	0	0
3	0	0	0	0
6	0	1	0	0
4	0	0	0	0
5	0	0	0	0
5	1	0	0	0
1	0	0	1	0
6	0	1	1	0
6	0	0	0	0

6	1	1	1	1
4	1	1	1	1
0	1	1	1	0
6	1	1	0	0
2	1	1	1	1
3	1	0	0	0
3	0	0	0	0
7	0	0	0	0
5	1	1	1	1

4	1	0	0	1
2	0	0	0	0
5	1	1	1	1
5	1	1	1	1
3	0	0	0	0
5	1	1	1	1
5	0	0	0	0
5	1	1	1	1
10	1	1	1	1

10	1	1	1	0
5	0	0	0	0
5	1	1	1	1
3	1	1	0	0
4	1	1	1	1
1	0	0	0	0
1	0	0	0	0
4	0	0	0	0
5	1	1	1	0

1	0	0	0	0
2	0	0	0	0
7	1	1	1	1
0	0	0	0	0
11	1	1	1	1
7	1	1	1	1
6	0	0	0	0
5	0	0	0	0
10	1	1	1	0

4	1	1	1	1
1	1	1	1	1
13	0	0	0	0
4	1	1	1	1
2	1	1	1	1
3	0	0	0	0
3	1	1	1	1
8	1	1	1	1
8	1	1	1	0

3	0	0	0	0
7	1	1	1	1
5	1	1	1	1
4	0	0	0	0
4	0	0	1	0
3	1	1	1	1
5	0	0	0	0
9	1	0	0	0
4	1	1	1	0

3	1	1	1	1
4	1	1	1	1
2	1	1	0	0
4	0	0	0	0
10	1	1	1	0
4	1	1	1	1
3	1	1	0	0
5	1	1	1	1
1	1	1	1	1

4	1	1	1	1
0	0	0	0	0
4	1	1	1	0
7	1	0	1	1
2	1	1	1	0
3	1	1	1	1
1	0	0	0	0
9	0	0	0	0
2	1	1	1	0

3	1	0	0	0
4	1	0	0	1
5	1	1	1	1
5	1	0	0	0
3	1	0	1	0
4	0	1	1	0
5	1	1	1	1
4	1	1	1	1
2	0	0	0	0

0	1	1	1	1
3	1	1	1	0
4	0	0	0	0
5	0	0	1	0
1	1	1	1	1
3	1	0	0	0
5	0	0	0	1
2	0	0	0	0
4	1	1	1	1

1	1	0	0	0
4	1	1	1	1
1	0	0	0	0
0	0	0	0	0
5	0	0	1	0
5	1	1	1	1
3	1	1	1	1
8	1	1	1	0
3	1	1	1	1

6	1	1	1	0
3	0	0	0	0
3	0	0	0	0
2	1	1	1	1
4	1	1	1	1
4	0	0	0	0
6	1	1	1	1
2	1	1	1	0
0	0	0	0	0

6	1	1	1	1
7	1	1	1	1
7	1	1	1	1
7	0	0	1	0
3	1	0	1	0
4	1	1	1	0
10	1	1	1	0
3	0	0	0	0
3	1	0	1	0

3	1	1	1	0
8	1	1	1	1
7	1	1	1	1
8	1	1	1	1
7	1	1	0	0
5	0	1	0	0
6	0	0	0	0
7	1	1	1	1
7	1	1	1	0

7	1	1	1	0
5	0	0	0	0
1	0	0	0	0
3	1	1	1	0
4	0	0	0	0
2	1	1	1	1
3	0	0	0	0
3	0	0	0	0
4	1	1	1	1

4	1	0	0	0
5	1	1	1	1
4	1	1	1	1
5	1	1	1	0
2	0	0	0	0
9	1	1	1	1
6	1	1	1	1
2	0	0	0	0
5	1	1	1	1

3	0	0	0	0
4	1	1	1	1
3	1	1	1	1
2	0	0	0	0
7	0	0	0	0
6	1	1	1	0
3	1	1	1	1
2	0	0	0	0
6	0	0	0	0

6	1	1	1	0
4	1	1	1	0
6	1	1	1	0
8	0	0	0	0
9	1	1	1	1
8	1	1	1	0
5	0	0	0	0
7	1	1	1	1
5	0	0	0	0

4	0	0	0	0
4	0	0	0	0
1	1	1	1	0
1	0	0	0	0
1	1	1	1	0
3	0	0	0	0
2	1	1	1	1
3	0	0	0	0
5	0	0	0	0

3	0	0	0	0
0	0	0	0	0
5	1	1	1	1
4	0	0	0	0
0	1	1	1	0
0	0	1	1	0
0	0	0	0	0
9	0	1	0	0
4	1	1	1	0

6	0	0	0	0
1	1	1	1	1
3	0	0	0	0
3	1	1	1	0
2	1	1	1	0
2	1	1	1	0
3	0	0	0	0
3	1	1	0	1
5	1	1	1	1

3	1	0	0	0
4	1	0	1	0
7	0	0	0	0
5	1	1	1	1
8	1	1	1	1
5	1	1	1	0
1	0	0	0	0
0	0	0	0	0
3	0	1	1	0

1	0	1	1	0
2	0	1	1	0
2	0	0	0	0
3	0	0	0	0
5	0	0	1	0
10	1	1	1	1
0	1	1	1	0
2	1	1	1	0
5	1	1	1	1

3	1	1	1	1
4	1	1	1	0
6	0	0	1	0
6	0	0	0	0
9	1	1	1	0
4	0	0	0	0
8	1	1	1	1
10	1	1	1	0
3	1	1	1	1

3	0	0	0	0
3	0	0	0	0
4	1	1	1	1
5	1	1	1	0
5	1	1	1	0
1	0	0	0	0
4	0	0	0	0
5	1	1	1	1
6	1	0	0	0

6	0	0	0	0
3	0	0	0	0
2	0	0	0	0
3	1	1	1	1
4	0	0	0	0
1	0	0	0	0
3	0	0	0	0
4	1	1	1	0
2	1	1	1	0

1	1	0	1	0
1	1	0	0	0
0	0	0	0	0
5	1	1	1	1
4	1	1	1	1
0	0	0	0	0
4	1	1	1	1
5	0	0	0	0
7	0	0	0	0

5	1	1	1	1
7	1	1	1	1
0	1	1	1	0
2	1	1	1	1
3	1	1	1	0
2	0	0	0	0
4	0	0	0	0
1	1	1	1	0
0	0	0	0	0

0	1	1	1	1
6	1	1	1	0
2	1	1	1	0
4	1	1	1	1
6	1	0	0	0
6	1	1	1	1
3	0	0	0	0
8	1	1	1	1
3	1	1	1	1

0	1	1	0	1
0	1	1	1	1

v_amenzaaotro	v_atemoriza	v_econ	v_bofetada	v_pateado
0	0	0	0	0
0	0	0	0	0
0	0	0	0	0
0	0	0	0	0
0	0	0	0	0
0	0	0	0	0
0	0	0	0	0
0	0	0	0	0

0	0	0	0	0
0	0	0	0	0
0	0	0	0	0
0	0	0	0	0
0	0	0	0	0
0	1	0	1	0
0	0	0	0	0
0	0	0	0	0
0	0	0	0	0

0	0	0	0	0
0	0	0	0	0
0	0	0	0	0
0	0	0	0	0
0	0	0	0	0
1	1	1	1	1
0	0	0	1	0
0	0	0	0	0
0	0	0	0	0

0	0	0	0	0
1	1	1	1	1
0	0	0	1	0
1	1	1	1	1
0	0	0	0	0
1	0	1	1	1
0	0	0	0	0
0	1	1	1	0
0	0	0	0	0

0	0	0	0	0
0	0	1	0	0
0	1	1	1	0
0	0	1	0	0
0	0	0	1	0
0	0	0	0	0
0	1	0	1	1
0	0	0	0	0
0	0	0	0	0

1	1	1	1	0
0	0	0	0	0
0	0	1	1	0
1	1	1	1	1
1	1	0	1	0
0	0	1	0	0
0	0	0	0	0
0	0	0	0	0
0	0	0	0	0

0	0	0	0	0
0	0	0	0	0
0	0	0	0	0
0	0	0	0	0
0	0	0	0	0
0	0	0	0	0
0	0	0	0	0
0	1	1	1	0
0	0	1	0	0

1	1	0	1	1
0	0	0	0	0
0	1	1	1	1
0	0	0	0	0
0	1	0	0	0
0	1	1	1	1
0	0	0	0	0
1	1	1	1	0
0	0	0	0	0

1	1	0	1	1
1	1	1	1	1
0	0	0	0	0
1	1	0	1	0
0	0	0	0	0
0	1	1	1	1
0	0	0	0	0
0	0	0	0	0
0	1	0	1	0

0	0	0	0	0
1	1	0	1	1
0	0	0	0	0
0	0	0	0	0
0	0	0	0	0
0	1	1	0	0
0	0	1	1	0
1	1	1	1	1
0	1	1	1	0

0	0	0	0	0
1	0	0	0	0
0	1	0	1	0
0	1	0	0	0
0	0	0	0	0
0	0	0	1	0
0	0	0	1	0
0	0	0	0	0
1	0	0	1	1

0	1	0	0	0
0	0	0	1	1
0	0	0	0	0
0	0	0	0	0
1	1	1	1	1
1	1	1	1	0
0	1	1	1	0
0	0	0	0	0
1	1	1	1	1

1	1	1	1	1
0	0	0	0	0
0	0	0	0	0
1	1	1	1	1
0	1	1	1	1
1	1	1	0	0
0	0	0	0	0
0	1	0	0	0
0	0	0	1	1

0	0	0	0	0
0	0	0	0	0
0	0	0	0	0
0	0	0	0	0
0	0	1	1	0
1	1	1	1	0
0	0	0	0	0
0	0	0	0	0
0	0	0	0	0

0	1	1	1	0
1	1	0	0	0
0	0	0	1	1
0	1	1	1	0
0	0	0	0	0
0	1	0	0	0
0	0	0	0	0
0	0	0	0	0
0	0	0	0	0

0	0	1	1	1
0	0	0	1	0
0	0	0	0	0
1	1	1	1	1
0	0	0	0	0
0	0	0	0	0
0	0	0	0	0
0	0	0	0	0
0	0	0	0	0

0	1	0	0	0
0	1	0	1	1
0	0	0	0	0
0	1	0	1	0
0	0	0	0	0
1	1	0	1	0
0	0	0	0	0
1	1	0	1	1
0	1	1	0	0

0	1	0	1	1
0	0	0	0	0
0	0	1	1	0
0	1	0	1	0
0	1	1	0	0
0	0	0	0	0
0	0	0	0	0
0	0	0	0	0
0	0	0	0	0

0	1	1	1	0
0	0	0	0	0
0	0	1	0	0
0	1	1	1	1
0	1	0	1	1
0	1	0	1	0
1	1	0	1	1
0	0	1	0	0
1	0	0	0	0

0	0	0	0	0
0	0	0	0	0
0	1	1	0	0
0	0	0	0	0
0	0	0	0	0
0	0	0	0	0
0	0	0	0	0
0	0	0	0	0
0	0	0	0	0

1	1	0	1	1
0	1	0	0	0
0	0	0	1	0
0	1	0	1	0
0	1	0	1	0
0	0	0	0	0
0	0	0	0	0
0	0	0	0	0
1	1	0	1	1

0	1	0	1	0
0	0	0	0	0
1	1	1	1	1
1	1	1	1	1
0	0	0	0	0
0	1	1	1	0
0	0	0	0	0
1	1	1	1	1
1	1	0	0	0

0	1	1	0	0
0	0	0	0	0
0	1	1	1	1
1	0	0	0	0
0	0	0	1	1
0	0	0	0	0
0	0	0	0	0
0	0	0	0	0
0	0	0	0	0

0	0	0	0	0
0	0	0	0	0
1	1	0	0	0
0	0	0	0	0
1	0	0	1	1
1	0	1	1	1
0	0	0	0	0
0	0	0	0	0
0	0	0	0	0

0	1	0	0	1
0	0	0	1	0
0	0	0	0	0
0	0	0	0	0
1	1	1	1	1
0	0	0	0	0
1	0	1	1	1
0	0	0	0	0
0	1	0	1	0

0	0	0	0	0
1	1	0	1	1
0	0	1	1	1
0	0	0	0	0
0	1	0	0	0
1	1	1	1	1
0	0	0	0	0
0	0	0	1	1
0	1	0	1	1

0	1	0	1	0
0	1	0	1	0
0	1	0	0	0
0	1	0	0	0
0	1	0	1	1
0	1	1	1	1
0	1	0	1	0
0	1	0	1	1
1	1	1	1	0

0	1	0	1	1
0	0	0	0	0
0	0	0	0	0
1	1	0	1	0
0	1	0	1	0
1	1	1	1	1
0	0	0	0	0
0	0	0	0	0
0	0	0	1	1

0	0	0	0	0
0	0	0	1	1
1	1	1	1	0
0	0	0	0	0
0	1	0	0	0
0	0	0	0	0
1	1	0	1	0
0	1	1	1	0
0	0	0	0	0

1	0	0	0	1
0	1	1	0	0
0	0	0	0	0
0	0	0	0	0
1	1	1	1	1
0	0	0	0	0
1	1	0	0	0
0	0	0	0	0
1	1	1	1	0

0	0	0	0	0
0	1	1	1	0
0	0	0	0	0
0	0	0	0	0
0	0	0	1	1
1	1	1	1	1
0	1	0	1	1
0	0	0	0	0
0	1	0	1	1

1	1	0	0	0
0	0	0	0	0
0	0	0	0	0
0	1	1	0	0
0	1	1	1	1
0	0	0	1	0
0	1	0	0	1
0	0	1	1	0
0	0	0	0	0

0	0	1	0	0
0	1	0	1	0
0	1	0	0	0
0	0	0	0	0
0	0	0	0	0
0	0	0	0	0
0	0	0	0	0
0	0	0	0	0
1	1	0	1	1

0	0	0	0	0
1	1	1	1	0
1	1	0	0	0
1	1	1	1	1
0	1	0	1	0
0	0	0	0	0
0	0	0	1	0
1	0	1	1	1
0	1	1	0	0

0	0	1	0	0
0	0	0	0	0
0	0	0	0	0
0	0	0	0	0
0	0	0	0	0
0	0	0	1	1
0	0	0	0	0
0	0	0	0	0
0	1	0	1	0

0	0	0	0	0
1	1	0	0	0
1	1	0	1	0
0	0	0	1	0
0	0	0	0	0
0	0	1	1	0
0	0	1	1	1
0	0	0	0	0
1	1	0	1	1

0	1	0	1	0
0	1	0	1	0
1	0	1	0	0
0	0	0	0	0
0	0	1	1	1
0	0	0	0	0
1	1	1	1	1
0	0	0	0	0
0	0	0	0	0

0	0	0	0	0
0	0	0	0	0
1	1	1	0	0
0	0	0	0	0
1	1	1	1	1
0	0	0	0	0
0	0	0	0	0
1	1	1	1	1
0	0	0	0	0

0	0	0	0	0
0	0	0	0	0
0	1	0	1	0
0	0	0	0	0
0	0	0	0	0
0	0	0	0	0
0	0	1	1	1
0	0	0	0	0
0	0	0	0	0

0	0	0	0	0
0	0	0	0	0
0	1	1	1	1
0	0	0	0	0
0	0	0	0	0
0	1	1	0	0
0	0	0	0	0
0	0	0	1	0
1	1	1	1	0

0	0	0	0	0
0	0	0	1	0
0	0	0	0	0
0	0	0	0	0
0	1	0	1	0
0	0	0	1	1
0	0	0	0	0
0	0	1	0	0
0	0	1	0	1

0	1	0	1	0
0	0	0	0	0
0	0	0	0	0
0	1	0	0	0
1	1	1	1	1
0	0	0	1	0
0	0	0	0	0
0	0	0	0	0
0	0	0	0	0

0	0	0	0	1
0	0	0	0	0
0	0	0	0	0
0	0	0	0	0
0	0	0	1	0
1	1	1	1	1
0	0	0	0	0
0	0	0	0	0
1	1	1	1	0

0	1	1	1	1
0	0	0	1	1
0	0	0	0	0
0	0	0	0	0
1	1	0	0	0
0	0	0	0	0
1	1	0	1	1
0	0	0	0	0
1	1	0	1	0

0	0	0	0	0
0	0	0	0	0
0	1	0	1	0
0	0	0	0	0
0	0	0	1	0
0	0	0	0	0
0	0	0	0	0
0	1	0	1	1
0	0	0	0	0

0	0	0	0	0
0	0	0	0	0
0	0	0	0	0
1	1	0	1	0
0	0	0	0	0
0	0	0	0	0
0	0	0	0	0
0	0	0	0	0
0	0	0	0	0

0	0	0	0	0
0	1	0	0	0
0	0	0	0	0
1	1	1	1	0
0	0	0	1	0
0	0	0	0	0
0	0	0	1	1
0	0	0	0	0
0	0	0	0	0

1	1	1	1	1
0	1	1	1	1
0	0	0	1	0
1	0	1	0	0
0	1	0	0	0
0	0	0	0	0
0	0	0	0	0
0	0	0	0	0
0	0	0	0	0

1	1	0	1	0
0	0	0	0	1
0	0	0	0	0
1	1	0	1	0
0	0	0	0	0
1	1	0	1	1
0	0	0	0	0
1	1	1	1	1
1	1	1	1	1

1	1	1	0	0
1	1	1	1	1

v_punetazos	v_paliza	v_sex	SUM_ENF_MED_TOD_UNA	hamilton_total_CUATRO
0	0	0	1	3
0	0	0	1	4
0	0	0	1	1
0	0	0	1	4
0	0	0	1	2
0	0	0	0	4
0	0	0	1	2
0	0	0	0	4

0	0	0	1	1
0	0	0	0	2
0	0	0	1	2
0	0	0	0	2
0	0	0	1	1
0	0	0	0	1
0	0	0	1	4
0	0	0	1	1
0	0	0	1	2

0	0	0	0	3
0	0	0	1	3
0	0	0	1	1
0	0	0	1	4
0	0	0	1	2
1	1	0	1	1
0	0	0	1	3
0	0	0	1	1
0	0	0	1	1

0	0	0	1	4
1	1	1	0	4
1	1	0	0	1
1	1	1	1	4
0	0	0	1	2
1	1	1	0	1
0	0	0	1	2
1	1	0	0	1
0	0	0	0	2

0	0	0	0	1
0	0	0	1	1
0	0	0	0	1
0	0	0	1	2
0	0	0	0	1
0	0	0	0	1
1	1	0	0	2
0	0	0	0	1
0	0	0	1	2

0	0	0	1	4
0	0	0	0	2
1	0	1	1	4
1	1	1	0	3
0	0	0	0	2
0	0	0	0	3
0	0	0	0	2
0	0	0	0	2
0	0	0	1	2

0	0	0	1	2
0	0	0	1	2
0	0	0	1	2
0	0	0	0	2
0	0	0	1	2
0	0	0	1	2
0	0	0	0	2
1	1	0	1	2
0	0	0	1	2

1	0	1	1	2
0	0	0	1	1
1	0	1	1	2
0	0	0	0	1
0	0	0	1	2
0	1	1	0	2
0	0	0	1	1
1	0	1	1	1
0	0	0	1	2

1	1	1	1	1
1	1	1	1	1
0	0	0	0	4
1	0	0	1	2
0	0	1	0	2
1	1	1	1	4
0	0	0	1	3
0	0	0	1	3
0	0	1	1	2

0	0	0	1	2
1	0	1	1	4
0	0	1	0	3
0	0	0	1	1
0	0	0	1	4
0	0	0	0	4
0	0	0	1	1
1	1	1	1	4
0	0	1	1	4

0	0	0	0	2
0	0	1	0	4
0	0	0	0	4
0	0	0	1	3
0	0	0	0	4
0	0	0	0	4
0	0	0	0	4
0	0	0	1	2
1	0	0	1	4

0	1	1	0	3
1	1	1	0	4
0	0	0	0	2
0	0	0	1	1
1	0	1	1	4
0	0	1	0	3
0	0	0	1	1
0	0	0	0	1
1	1	1	0	4

1	0	0	0	1
0	0	0	1	1
0	0	0	1	2
1	1	0	1	3
1	1	0	1	1
0	0	0	1	4
0	1	0	0	1
0	0	0	1	4
1	1	0	0	4

0	0	0	0	3
0	0	0	1	3
0	0	0	1	4
0	0	0	0	3
0	0	1	1	2
0	0	1	1	1
0	0	0	1	4
0	0	0	1	1
0	0	0	0	1

0	0	1	0	4
0	0	0	0	4
0	0	0	1	1
0	0	0	0	4
0	0	0	0	3
0	0	0	0	3
0	0	0	1	2
0	0	0	0	3
0	0	0	1	1

1	1	1	1	2
0	0	0	0	3
0	0	0	0	1
1	0	1	1	4
0	0	0	1	3
0	0	0	0	2
0	0	0	0	1
0	0	0	0	1
0	0	0	0	1

0	0	0	0	3
1	1	0	1	3
0	0	0	1	4
0	0	0	1	1
0	0	0	0	1
1	0	1	0	2
0	0	0	1	3
1	0	1	0	3
0	0	0	1	2

1	1	1	0	1
0	0	0	0	3
0	0	0	0	1
0	0	0	0	1
0	0	0	1	3
0	0	0	0	2
0	0	0	1	4
0	0	0	1	2
0	0	0	1	4

1	0	0	0	4
0	0	0	0	3
0	0	0	0	3
1	1	1	0	4
0	0	1	0	4
0	0	0	0	3
0	0	0	1	3
0	0	0	1	2
0	0	0	1	3

0	0	0	1	2
0	0	0	0	2
0	0	1	1	4
0	0	0	1	3
0	0	0	1	3
0	0	0	0	2
0	0	0	0	3
0	0	0	0	2
0	0	0	1	2

1	1	0	0	4
1	0	0	0	2
1	0	0	1	1
0	0	0	1	3
0	0	0	1	1
0	0	0	1	2
0	0	0	1	1
0	0	0	0	4
1	1	1	1	1

0	0	0	0	2
0	0	0	0	3
1	1	1	1	1
1	1	1	1	1
0	0	0	1	2
0	0	1	0	1
0	0	0	0	1
1	1	1	1	3
0	0	1	0	3

0	0	0	1	4
0	0	0	0	3
1	1	0	0	1
0	0	0	1	1
1	1	0	1	1
0	0	0	1	1
0	0	0	0	1
0	0	0	1	4
0	0	0	0	1

0	0	0	0	1
0	0	0	0	1
0	0	0	0	2
0	0	0	1	1
1	1	0	0	4
1	1	1	1	4
0	0	0	1	4
0	0	0	0	4
0	0	0	1	2

1	1	0	0	1
0	0	0	1	1
0	0	0	0	4
0	0	0	0	4
1	1	0	1	3
0	0	0	1	2
1	1	1	1	1
0	1	0	1	3
1	1	1	0	4

0	0	0	1	1
1	1	1	1	3
1	1	1	1	2
0	0	0	0	2
0	0	0	0	3
0	0	0	1	1
0	0	0	1	3
1	1	0	0	4
0	0	0	1	2

0	0	0	0	1
1	0	0	0	3
0	0	0	0	1
0	0	0	1	2
1	0	0	0	4
1	1	0	1	4
0	0	0	1	2
1	0	1	1	2
0	0	0	1	1

1	0	1	1	3
0	0	0	1	1
0	0	0	1	3
1	0	1	1	4
0	0	0	0	1
1	0	0	0	2
0	0	0	1	2
0	0	0	0	1
0	1	1	0	3

0	0	0	0	1
1	1	0	1	4
1	0	1	1	4
0	0	0	1	2
0	0	0	1	1
0	0	0	1	4
1	0	1	0	4
1	0	0	1	2
0	0	0	0	1

1	0	0	0	1
0	0	0	0	4
0	0	0	0	4
0	0	0	0	4
1	1	1	1	2
0	0	0	1	3
0	0	0	1	4
0	0	0	0	4
0	1	1	1	3

0	0	0	1	4
1	1	0	0	4
0	0	0	0	1
0	0	0	1	1
1	0	0	1	2
1	1	0	1	4
1	1	1	1	1
0	0	0	1	3
1	1	0	1	2

0	0	1	1	3
0	0	0	0	2
0	0	0	1	1
1	0	0	1	1
1	0	0	0	1
0	0	0	1	2
1	1	0	0	2
1	0	0	1	2
0	0	0	1	1

0	0	0	0	4
0	0	0	1	3
0	0	0	0	4
0	0	0	0	4
0	0	0	1	3
0	0	0	1	2
1	0	0	0	4
0	0	0	1	1
0	0	1	1	2

0	0	0	0	2
1	1	1	0	4
0	0	0	0	1
1	1	1	1	4
0	1	0	1	2
0	0	0	1	1
1	0	0	1	2
1	0	1	0	4
1	0	0	1	3

1	1	1	0	2
0	0	0	0	1
0	0	0	1	2
0	0	0	1	1
0	0	0	1	1
1	1	0	1	4
0	0	0	1	2
0	0	0	1	2
0	0	0	1	1

0	0	0	1	3
0	0	0	0	2
1	1	0	1	2
0	0	0	1	2
0	0	0	1	1
1	0	0	0	4
1	1	1	0	4
0	0	0	0	1
1	0	1	1	3

0	0	1	1	1
1	0	1	1	2
0	0	0	1	3
0	0	0	0	1
1	1	0	0	2
0	0	0	1	4
1	1	1	0	2
0	0	0	0	4
0	0	0	0	3

0	0	0	1	2
0	0	0	1	4
0	0	0	1	2
0	0	0	0	4
1	1	1	0	4
0	0	0	0	4
0	0	0	0	3
1	1	1	1	4
0	0	0	0	1

0	0	0	0	3
0	0	0	0	2
0	0	1	1	1
0	0	0	1	3
0	0	0	1	4
0	0	0	1	4
1	1	0	1	4
0	0	0	1	4
0	0	0	0	4

0	0	0	1	1
0	0	0	1	1
1	1	1	1	4
0	0	0	0	4
0	1	0	1	1
0	0	0	1	1
0	0	0	1	1
0	0	0	0	4
0	0	0	0	2

0	0	0	0	2
0	0	0	1	1
0	0	0	0	1
0	0	0	1	3
0	0	0	1	1
1	0	0	0	2
0	0	0	1	2
0	0	0	1	3
0	0	0	0	2

0	0	0	0	1
0	0	0	0	3
0	0	0	0	2
0	0	0	1	4
1	1	1	1	4
0	0	0	0	4
0	0	0	1	1
0	0	0	1	1
0	0	0	1	4

1	0	0	0	3
0	0	0	0	3
0	0	0	0	2
0	0	0	1	3
0	0	0	1	3
1	1	1	1	4
0	0	0	1	1
0	0	0	1	3
1	0	1	1	3

1	0	0	0	3
1	0	0	0	1
0	0	0	0	4
0	0	0	1	3
0	0	0	1	4
0	0	0	1	1
1	1	1	0	3
0	0	0	0	4
0	0	0	0	1

0	0	0	1	4
0	0	0	1	1
0	0	0	1	1
0	0	0	0	3
0	0	0	0	1
0	0	0	0	1
0	0	0	1	3
1	0	0	0	3
0	0	0	0	3

0	0	0	1	3
0	0	0	0	2
0	0	0	1	1
0	0	0	1	3
0	0	0	1	1
0	0	0	1	1
0	0	0	0	1
0	0	0	0	1
0	0	0	0	2

0	0	0	0	2
0	0	0	0	2
0	0	0	1	1
1	1	0	1	3
0	0	0	1	1
0	0	0	0	2
1	1	1	1	1
0	0	0	1	2
0	0	0	0	3

1	1	1	1	3
1	1	0	0	4
0	0	0	0	1
0	0	1	1	2
0	0	0	1	4
0	0	0	1	4
0	0	0	0	2
0	0	0	1	1
0	0	0	1	1

0	0	0	1	1
0	0	0	0	2
0	0	0	1	1
0	0	0	1	4
0	0	0	0	4
1	0	1	1	3
0	0	0	0	1
1	1	1	0	3
0	0	1	1	2

0	0	0	0	2
1	1	1	1	2

HAM_MITAD	HAM_TRES	OQ45_SD	OQ45_IR	OQ45_SR
2	3	53	21	21
2	3	61	17	12
1	1	35	15	8
2	3	64	11	16
1	2	54	18	9
2	3	47	28	7
1	2	0	20	7
2	3	62	20	7

1	1	58	21	19
1	2	55	15	5
1	2	55	26	15
1	2	0	17	17
1	1	44	0	10
1	1	0	10	14
2	3	53	17	7
1	1	57	22	2
1	1	55	23	14

2	2	57	23	19
2	2	78	28	20
1	1	37	7	3
2	3	73	31	7
1	2	47	14	12
1	1	59	26	19
2	2	63	20	8
1	1	15	4	0
1	1	40	0	7

2	3	63	22	9
2	3	71	15	12
1	1	46	13	12
2	3	67	22	19
1	2	55	23	14
1	1	44	10	5
1	1	42	9	11
1	1	70	30	6
1	2	52	19	14

1	1	46	7	11
1	1	39	16	14
1	1	61	19	9
1	1	44	13	15
1	1	35	9	3
1	1	35	10	9
1	2	45	0	11
1	1	0	0	12
1	2	64	0	15

2	3	69	0	20
1	1	58	0	16
2	3	74	30	23
2	2	0	21	10
1	1	63	22	19
2	2	71	29	17
1	2	59	31	21
1	2	54	18	19
1	2	61	12	16

1	2	63	25	19
1	2	71	21	18
1	2	35	10	7
1	2	0	5	8
1	2	71	18	22
1	2	54	13	19
1	2	37	18	9
1	2	37	0	1
1	2	83	28	15

1	2	0	20	18
1	1	30	14	6
1	2	49	18	0
1	1	35	17	6
1	2	40	14	6
1	1	79	19	23
1	1	48	10	10
1	1	76	26	19
1	1	36	11	9

1	1	55	25	15
1	1	52	0	10
2	3	37	0	6
1	2	32	16	15
1	1	52	30	26
2	3	92	30	26
2	2	80	22	22
2	2	35	11	10
1	2	66	22	0

1	2	66	22	13
2	3	57	17	17
2	2	0	27	14
1	1	35	11	7
2	3	73	9	14
2	3	0	13	13
1	1	36	0	2
2	3	75	27	18
2	3	83	22	16

1	2	48	0	23
2	3	70	20	6
2	3	61	21	16
2	2	59	26	4
2	3	75	22	18
2	3	43	16	6
2	3	73	11	21
1	2	47	9	9
2	3	78	18	22

2	2	53	13	11
2	3	88	24	16
1	2	0	17	10
1	1	46	14	0
2	3	69	18	9
2	2	89	23	22
1	1	0	0	4
1	1	45	0	10
2	3	90	40	20

1	1	29	5	2
1	1	21	7	9
1	2	50	12	19
2	3	76	7	16
1	1	49	0	12
2	3	61	25	20
1	1	40	10	10
2	3	74	21	9
2	3	58	15	14

2	3	61	20	26
2	2	38	7	13
2	3	0	25	16
2	3	75	15	18
1	2	45	18	2
1	1	47	20	8
2	3	66	22	10
1	1	48	13	2
1	1	34	7	6

2	3	68	15	17
2	3	0	24	20
1	1	65	30	19
2	3	74	32	20
2	2	46	3	5
2	2	74	28	10
1	2	47	15	2
2	2	63	5	18
1	1	28	2	4

1	2	40	9	7
2	2	50	16	8
1	1	55	15	12
2	3	0	21	6
2	3	72	16	25
1	1	61	15	19
1	1	26	5	6
1	1	48	10	16
1	1	0	0	9

2	2	64	14	20
2	2	0	14	16
2	3	60	22	17
1	1	41	12	6
1	1	38	0	4
1	2	57	20	21
2	2	0	25	14
2	2	67	17	23
1	2	86	24	23

1	1	58	21	19
2	3	46	15	10
1	1	44	16	23
1	1	38	10	20
2	2	60	14	9
1	2	48	7	3
2	3	55	10	9
1	2	31	2	6
2	3	87	33	19

2	3	81	24	25
2	2	45	24	17
2	3	50	20	17
2	3	49	27	15
2	3	46	15	17
2	2	30	19	11
2	3	55	16	5
1	2	68	27	16
2	2	52	22	21

1	2	39	11	17
1	1	26	8	2
2	3	57	17	19
2	2	69	21	4
2	3	34	10	13
1	2	50	11	6
2	2	53	9	17
1	2	60	18	21
1	2	51	26	24

2	3	75	27	12
1	2	50	18	15
1	1	0	21	15
2	2	0	25	18
1	1	0	14	9
1	2	41	13	14
1	1	38	8	9
2	3	84	23	20
1	1	41	9	24

1	2	59	21	13
2	2	72	13	6
1	1	49	10	7
1	1	62	23	12
1	2	36	17	10
1	1	59	10	5
1	1	54	12	7
2	2	84	12	23
2	2	72	18	23

2	3	94	26	25
2	2	51	19	12
1	1	0	20	16
1	1	0	17	19
1	1	0	18	7
1	1	34	16	16
1	1	24	11	1
2	3	57	12	22
1	1	36	0	8

1	1	29	5	0
1	1	30	16	14
1	1	0	22	23
1	1	6	10	1
2	3	71	28	10
2	3	57	16	10
2	3	74	28	18
2	3	73	20	23
1	2	58	16	3

1	1	34	17	7
1	1	16	14	3
2	3	0	0	0
2	3	75	25	0
2	2	47	18	2
1	2	0	0	3
1	1	44	0	11
2	2	72	28	16
2	3	79	26	12

1	1	53	10	11
2	2	48	24	12
1	1	0	18	12
1	1	0	20	13
2	2	59	15	19
1	1	62	19	21
2	3	66	17	11
2	3	88	29	10
1	2	55	10	10

1	1	64	16	5
2	2	43	13	4
1	1	47	31	14
1	2	54	24	23
2	3	68	18	21
2	3	0	23	19
1	1	49	15	14
1	2	57	21	0
1	1	64	0	15

2	2	51	22	15
1	1	42	8	15
2	3	0	24	19
2	3	76	26	20
1	1	50	27	23
1	2	51	13	22
1	2	30	7	9
1	1	57	26	16
2	3	44	9	9

1	1	42	13	19
2	3	38	8	14
2	3	23	4	11
1	1	54	14	14
1	1	44	17	12
2	3	47	13	18
2	3	68	20	11
1	2	72	0	7
1	1	46	8	16

1	1	42	15	9
2	3	58	30	21
2	3	64	26	23
2	3	80	17	31
1	1	37	25	9
2	2	41	17	7
2	3	55	9	13
2	3	80	8	4
2	3	70	0	27

2	3	0	12	16
2	3	0	28	16
1	1	38	0	12
1	1	29	14	10
1	2	0	12	0
2	3	0	26	15
1	1	36	21	5
2	2	0	25	10
1	2	49	11	7

2	2	69	17	14
1	1	58	22	15
1	1	46	8	6
1	1	41	16	4
1	1	36	12	5
1	2	64	4	13
1	2	68	16	19
1	2	78	22	16
1	1	1	2	2

2	3	77	38	24
2	3	73	15	17
2	3	59	21	17
2	3	66	21	22
2	3	45	20	14
1	2	39	8	8
2	3	87	26	22
1	1	53	15	17
1	2	50	18	13

1	2	38	19	6
2	3	57	17	20
1	1	69	14	16
2	3	74	18	21
1	2	47	20	2
1	1	57	11	10
1	2	44	18	11
2	3	64	17	20
2	2	59	17	20

1	2	58	31	19
1	1	49	14	9
1	2	50	11	5
1	1	45	7	7
1	1	41	7	6
2	3	73	14	10
1	2	51	18	0
1	1	36	7	7
1	1	34	11	7

2	2	70	4	6
1	2	60	25	3
1	2	59	10	15
1	2	36	14	19
1	1	28	18	9
2	3	87	15	22
2	3	87	34	18
1	1	32	8	10
2	2	74	26	19

1	1	60	18	5
1	1	37	15	8
2	2	43	23	8
1	1	0	21	6
1	2	47	22	6
2	3	54	15	10
1	2	52	27	8
2	3	69	30	12
2	2	57	16	17

1	2	63	25	17
2	3	47	9	14
1	2	65	18	0
2	3	0	0	0
2	3	74	28	12
2	3	83	27	12
2	2	57	21	19
2	3	87	25	13
1	1	61	20	13

2	2	0	22	13
1	2	42	9	12
1	1	25	11	3
2	3	59	15	4
2	3	69	14	14
2	3	81	0	12
2	3	48	26	14
2	3	73	12	24
2	3	75	14	22

1	1	0	14	6
1	1	40	11	9
2	3	64	19	8
2	3	80	0	16
1	1	0	10	8
1	1	16	16	4
1	1	11	2	9
2	3	80	33	14
1	2	53	24	30

1	2	76	22	23
1	1	45	8	2
1	1	60	11	17
2	2	65	23	18
1	1	29	12	4
1	1	36	18	11
1	2	38	17	14
2	3	74	21	17
1	2	53	36	20

1	1	62	18	9
2	2	68	25	18
1	1	63	19	8
2	3	76	25	19
2	3	80	23	29
2	3	71	0	23
1	1	43	0	9
1	1	10	0	0
2	3	87	19	18

2	3	65	28	6
2	2	62	32	29
1	2	74	28	29
2	2	43	20	16
2	2	63	26	14
2	3	72	29	21
1	1	37	18	9
2	2	62	16	14
2	3	55	31	20

2	2	0	0	0
1	1	24	15	6
2	3	79	29	28
2	2	42	27	16
2	3	0	14	11
1	1	41	14	10
2	2	65	22	17
2	3	93	25	20
1	1	0	19	12

2	3	22	12	1
1	1	32	4	15
1	1	48	20	16
2	3	78	26	26
1	1	55	19	11
1	1	30	9	9
2	2	64	27	19
2	2	65	26	19
2	2	71	8	12

2	3	57	19	16
1	2	79	19	19
1	1	0	1	6
2	3	70	26	24
1	1	57	20	14
1	1	37	10	6
1	1	47	14	10
1	1	56	0	12
1	2	56	20	12

1	2	64	18	21
1	2	69	29	18
1	1	7	5	4
2	2	81	20	9
1	1	53	19	11
1	2	65	17	10
1	1	41	18	2
1	2	63	23	19
2	2	60	20	13

2	2	77	28	18
2	3	75	22	14
1	1	19	4	4
1	2	76	20	18
2	3	59	25	22
2	3	66	7	28
1	1	77	3	27
1	1	42	26	14
1	1	38	8	9

1	1	30	20	4
1	2	63	24	13
1	1	28	4	2
2	3	57	19	11
2	3	82	13	15
2	2	73	23	20
1	1	36	17	22
2	2	66	19	15
1	2	0	0	13

1	2	0	0	0
1	2	82	0	15

ocupacion	vive_con
	3
13	1
	1
	5
8	5
4	3
5	5
13	4

3	3
5	4
9	4
11	1
5	1
1	4
3	4
3	4
8	3

8	4
1	4
1	4
11	1
1	4
5	1
8	4
3	3
1	4

3	4
5	4
1	4
8	4
1	1
5	4
11	4
	4
8	4

10	4
1	4
1	4
1	4
11	1
12	4
1	4
10	4
1	3

3	4
10	4
1	4
8	4
1	4
4	4
1	4
13	4
1	3

1	4
1	1
1	1
3	4
	4
	3
1	4
1	1
3	4

1	4
12	4
1	4
1	1
1	1
10	4
1	3
5	4
1	3

1	4
11	4
13	4
1	4
1	3
9	1
3	4
1	1
6	4

5	4
5	4
7	4
1	4
13	4
8	4
8	1
8	1
1	1

8	4
6	4
14	4
10	4
3	2
6	4
6	4
1	5
1	1

1	4
5	4
1	4
1	3
1	4
3	4
5	4
4	4
1	1

13	4
13	4
1	4
8	4
5	1
1	4
1	4
5	4
8	3

5	3
3	3
12	3
4	2
12	4
1	1
4	2
4	5
2	1

13	3
1	4
5	4
13	4
12	4
11	4
11	1
	4
8	4

1	1
1	4
1	4
4	4
6	4
	4
3	4
3	4
1	4

3	4
	4
1	4
11	4
10	5
1	4
1	5
1	4
8	4

1	4
1	4
1	4
8	4
1	5
13	3
1	3
4	3
8	4

1	4
10	4
13	1
12	4
1	4
6	4
1	1
11	4
8	5

6	4
10	4
13	4
3	4
1	5
4	4
5	4
1	
1	4

4	3
8	4
1	1
1	5
1	3
12	4
1	1
1	5
1	4

3	1
1	3
12	4
1	4
12	4
12	4
7	4
5	4
8	4

1	3
6	4
5	4
1	4
1	4
1	4
1	4
1	4
4	4

1	4
1	4
10	4
11	4
10	4
3	1
1	4
10	4
9	4

10	5
9	1
2	4
1	3
1	3
3	4
8	4
1	4

1	4
1	3
3	4
8	4
1	4
1	4
3	5
3	4
	4

7	4
12	4
7	4
1	4
6	4
	3
1	4
13	2
3	4

1	4
3	4
1	3
1	4
	5
2	4
14	4
10	4
8	4

8	4
8	4
3	4
12	3
14	4
1	4
1	4
1	4
3	4

1	4
4	4
12	1
6	4
1	1
1	4
1	5
1	4
10	4

1	5
1	4
1	4
9	4
1	4
5	4
5	4
4	4
1	4

1	4
10	2
1	3
1	1
3	4
9	4
1	4
1	4
11	4

2	4
1	4
13	4
13	4
8	4
3	1
5	4
1	4
1	4

10	2
1	4
6	4
1	4
4	4
14	4
1	4
1	4
1	4

10	4
1	4
4	4
9	3
7	5
5	4
9	4
1	5
1	4

5	4
5	1
1	4
3	4
9	1
6	1
4	4
6	1
8	4

4	4
13	4
3	4
13	2
13	4
1	4
1	3
3	2
2	4

13	1
3	4
14	4
4	4
1	3
1	4
4	4
10	2

1	4
13	2
1	4
1	3
1	5
1	4
1	4
1	4
11	3

1	3
6	3
1	4
10	4
9	1
1	5
12	4
10	2
2	1

13	4
1	4
12	4
13	4
1	4
1	4
8	2
1	4
1	4

4	4
3	4
13	4
1	4
1	4
5	4
1	1
1	4
1	4

13	1
1	4
1	4
1	3
9	1
1	4
1	4
1	4
5	4

8	4
1	4
3	4
4	1
5	5
1	3
8	1
1	4
3	4

9	1
9	3
8	1
1	4
1	4
10	4
8	4
8	1
1	4

1	3
1	4
1	3
3	3
10	4
1	3
10	4
1	4
13	4

3	4
4	4
5	5
5	1
6	4
1	4
5	4
13	4
10	5

1	4
6	4
3	4
1	4
1	4
11	4
12	4
12	1
1	5

1	1
3	3
1	4
9	3
3	1
8	5
3	4
3	4
1	4

13	5
